# Tricyclic Fused Lactams by Mukaiyama Cyclisation of Phthalimides and Evaluation of their Biological Activity

**DOI:** 10.3390/antibiotics12010009

**Published:** 2022-12-21

**Authors:** Lewis T. Ibbotson, Kirsten E. Christensen, Miroslav Genov, Alexander Pretsch, Dagmar Pretsch, Mark G. Moloney

**Affiliations:** 1 The Department of Chemistry, Chemistry Research Laboratory, University of Oxford, 12 Mansfield Road, Oxford OX1 3TA, UK; 2 Oxford Antibiotic Group, The Oxford Science Park, Magdalen Centre, Oxford OX4 4GA, UK

**Keywords:** pyrrolidinone, aldol, antibacterial

## Abstract

We report that phthalimides may be cyclized using a Mukaiyama-type aldol coupling to give variously substituted fused lactam (1,2,3,9b-tetrahydro-5*H*-pyrrolo[2,1-*a*]isoindol-5-one) systems. This novel process shows a high level of regioselectivity for *o*-substituted phthalimides, dictated by steric and electronic factors, but not for *m*-substituted phthalimides. The initial aldol adduct is prone to elimination, giving 2,3-dihydro-5*H*-pyrrolo[2,1-*a*]isoindol-5-ones, and the initial cyclisation can be conducted in such a way that aldol cyclisation-elimination is achievable in a one-pot approach. The 2,3-dihydro-5*H*-pyrrolo[2,1-*a*]isoindol-5-ones possess cross conjugation and steric effects which significantly influence the reactivity of several functional groups, but conditions suitable for epoxidation, ester hydrolysis and amide formation, and reduction, which provide for ring manipulation, were identified. Many of the derived lactam systems, and especially the eliminated systems, show low solubility, which compromises biological activity, although in some cases, antibacterial and cytotoxic activity was found, and this new class of small molecule provides a useful skeleton for further elaboration and study.

## 1. Introduction

The critical importance of natural products in the development of pharmaceutically active compounds has been thoroughly documented, and although popularity of this approach has waned in recent years in favour of combinatorial and rational design, there have been strong calls for its reinvigoration [1]. These calls are particularly relevant for antibacterial agents, for which there is a serious deficit of new candidates in the drug pipeline [2,3], at a time when there is considerable urgency to expand therapeutics as a result of the rapid emergence of resistant bacterial strains [4]. The challenges peculiar to antibacterial drug discovery [5,6,7,8] imply that natural products often provide biologically validated start points suitable for immediate elaboration in the quest for new pharmaceutically useful agents [9,10]. It has recently been recognised that existing strategies for the discovery of new antibacterials have not been effective [11], probably as a result at least in part of over-reliance of combinatorial approaches leading to structurally narrow libraries [12,13], and there is an urgent need for the identification of novel leads for expanding the antibacterial drug development pipeline [14,15]. The work of Waldmann [16,17] and Danishefsky [18] has reiterated the importance of natural products as a starting point for drug discovery, and our contribution to this area has been to show that chemical libraries modelled on natural products [19], including equisetin [20], reutericyclin [21], kibdelomycin [22], and streptolydigin [23], which all possess a core tetramate unit, or oxazolomycin [24] and pramanicin [25], which possess an α-hydroxypyroglutamate core, may exhibit significant antibacterial activity and provide useful opportunities for further optimisation. Critical to the success of this work has been the finding that C-acyl or C-carboxamide side chains may be introduced under mild conditions to tetramate and pyroglutamate skeletons [19] and that this leads to enhanced antibacterial activity. It would appear, therefore, that an α, α, α-tricarbonyl unit comprises, at least in part, the active pharmacophore, and this was corroborated by the finding that the core tetramate without an α, α, α-tricarbonyl unit had little intrinsic antibacterial activity [26].

The recent discovery of pyrrolizilactone [27], UCS1025A and B [28,29,30] and CJ-16264 [31], is of interest since all are comprised of a common lactam-lactone fused ring core and C-acyl decalin side chain. Studies of the biosynthesis [32], synthesis [33,34,35,36], and SAR [37] of UCS1025A suggest that the core skeleton might offer an opportunity for development, not least because of its similarity with bioactive tetramates, which are also appended with decalins [38]. Of significance is the antibacterial bioactivity of these systems, with MIC values of typically 1–15 ug/mL against Gram-positive MDR strains and some Gram-negative ones [31]. Limited SAR analysis with three CJ-16,264 stereoisomers shows MIC values of 2–16 ug/mL against MRSA, *E. faecelis*, and *E. faecium* [39]. As a result, the development of methodology for their total synthesis has attracted attention [40,41] and the total synthesis of myceliothermophins C, D, and E [42], a related structural type, has also recently been achieved. The synthesis of the azabicyclo[3.3.0]octane core provides a key background [43] and an unusual approach to the pyrrolizidine core from an 8-membered ring by transannular cyclisation has been reported [44]. Of particular interest was the elegant ring cyclisation methodology originally reported by Lambert [34] and developed later by both Hoye [35,45] and Christmann [33,46,47], since this provided rapid entry to the core lactam system from maleimides by an aldol-like ring closure, using in situ generated silyl enolates as nucleophiles. We have recently reported that this approach is suitable for substituted maleimides, and can be used to access a small library of novel pyrrolidinones [48]; of interest was their lack of antibacterial activity, but a similar phenomenon had been observed for unsubstituted tetramates [19]. We report here that the aldol cyclisation may be further extended to phthalimides, and that this gives rise to a range of functionalised systems whose biological activity has been assessed.

## 2. Results and Discussion

**Scheme 1:**
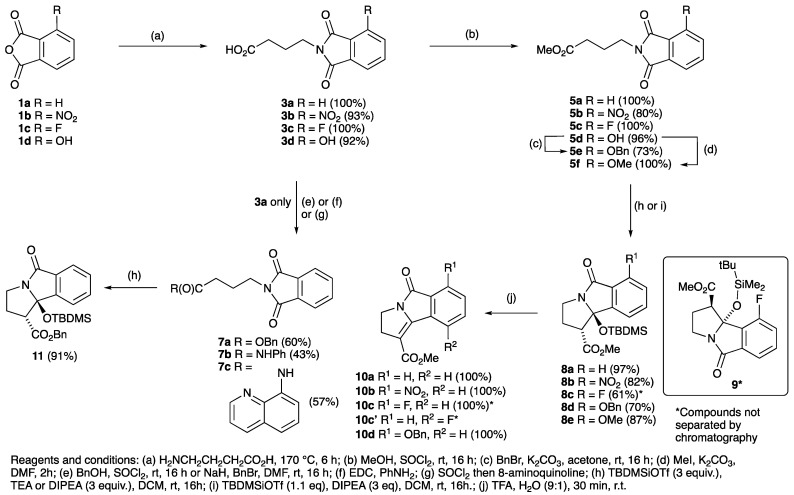
Synthesis and ring closure of substituted phthalimides.

**Scheme 2:**
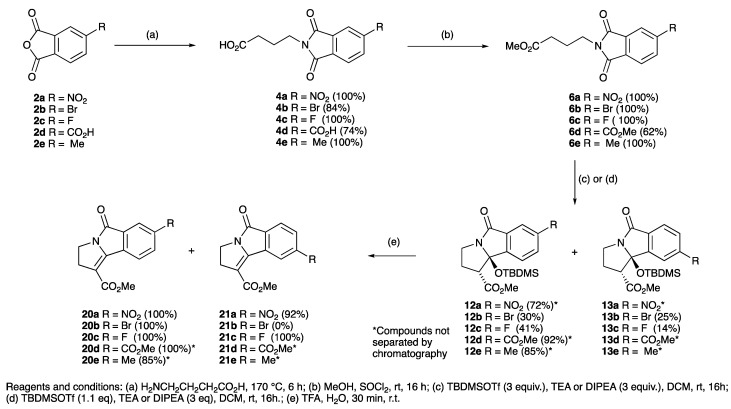
Synthesis and ring closure of substituted phthalimides.

Substituted phthalic anhydrides **1a**–**d** (Scheme 1) and **2a–e** (Scheme 2) and γ-aminobutyric acid (GABA) were condensed by heating without solvent to 170 °C for 6 h, during which the molten mixture slowly turned to a straw yellow colour, using the previously reported procedure [49,50,51,52,53], and successfully gave a range of substituted systems in excellent yields. Upon completion of the reaction, the cooled solid mass was dissolved in dichloromethane and washed using 0.5 N HCl, giving the desired products **3a–d** and **4a–e** in excellent yields (Scheme 1 and Scheme 2, and Table 1). Esterification of acids **3a–d** and **4a–e** to their corresponding methyl esters **5a–d** and **6a–e** using thionyl chloride and MeOH at rt over 16 h gave the products in quantitative yields in many cases (Table 1); however, this was ineffective for **4b** due to its unexpectedly low solubility, and synthesis of **6b** required direct condensation of methyl γ-aminobutanoate hydrochloride with the anhydride in toluene with DIPEA under reflux for 16 h, giving the desired product **6b** (quantitative yield), the structure of which was confirmed by single crystal X-ray diffraction (Figure S1, Supporting Information (SI)) [54,55,56,57]. Protection of the free hydroxyl group of hydroxyphthalimide **5d** as the OBn and OMe ethers **5e** and **5f** was achieved using standard procedures in excellent yields. Benzylation of **3a** using thionyl chloride/benzyl alcohol gave benzyl ester **7** in up to 60% yield (Scheme 1) and conversion to anilide **7b** and 8-amidoquinoline **7c** using the appropriate amine was similarly possible.

With the required phthalimides in hand, **5a** was treated with *N*,*N*-diisopropylethylamine (DIPEA) and *t*-butyldimethylsilyl triflate (TBDMSOTf) according to a modification of the literature’s procedure [34], and purification using flash column chromatography afforded the silyl containing tricyclic pyrrolizidinone **8a** with a good yield of 97% (Scheme 1 and Table 1). This material was readily characterised by standard spectroscopic techniques; of interest were the non-equivalent silyl dimethyl groups that had shifted upfield to −0.08 and −0.51 ppm due to the anisotropy of the adjacent aromatic ring, consistent with ring closure. The stereochemistry was confirmed by a combination of one and single crystal X-ray diffraction (Figure S1, ESI) [54,55,56,57]; the *trans*-relationship of the methyl ester and silyloxy moiety were evident, placing the methyl ester into a pseudoaxial position, and with one of the silyl methyl groups located over the aromatic ring, accounting for the shielding observed in the NMR spectrum. While the structure of **8a** was further confirmed by LRMS and HRMS, with the major mass ions being 362 [MH^+^] and 384 [MNa^+^] as expected, importantly these signals were accompanied by a mass ion of 132 less than the desired product at 230 [MH+]; this was consistent with in situ desilyloxylation giving **10a**. In fact, the cyclisation of **5a** was found to be unreliable, instead often giving **10a** directly and quantitively by in situ elimination. Synthesis of similar tetrahydro-1*H*-pyrrolo[2,1-a]isoindoles [58,59,60] and their unsaturated systems [61,62,63] has been reported. In order to understand the progress of this reaction, varying equivalents of TBDMSOTf were used with phthalimides **5a,b** and it was found that while the formation of the products **8a,b** was achievable in high yields with 1.1 equivalents of TBDMSOTf, nearly quantitative direct conversion of **8a** to unsaturated pyrrolizidinones **10a,b** could be achieved using 2.0 equivalents of TBDMSOTf (Table S1, ESI).

Application of these cyclisation conditions to phthalimides **5b–f** successfully gave cyclised products **8b–e** in good to excellent yield (Scheme 1 and Scheme 2 and Table 1). TLC and ^1^H NMR spectroscopic analysis indicated formation of only a single regioisomer, except for **5c** which gave an isomeric mixture of **8c** also containing **9** (ratio 7:1). Structural assignment was confirmed in the case of **8b,c, 9** and **12a** by single crystal X-ray diffraction (Figure S1, ESI) [54,55,56,57]. The mode of cyclisation appeared to be dictated by the sterically bulky substituents on the aromatic ring, but in the case of **5c** was biased by both the small size and electronegativity of the fluorine substituent which also gave the alternative isomer **9**. Substituted phthalimides **6a–e** were subjected to the same ring-closing conditions, giving good to excellent yields of products **12a–e** and **13a–e** (Scheme 1 and Scheme 2, and Table 1), usually as an approximately equal mixture of isomers, which proved to be difficult to separate by flash column chromatography, and arising by ring closure onto either phthalimide carbonyl group. While the cyclisations using methyl esters were very high yielding and reliable reactions, of interest was whether reactions of substrates with bulkier esters would be as effective; benzyl ester variant **7a** in fact cyclised to **11** with an excellent yield of 91% using the standard conditions (1.1 eq TBDMSOTf, 3 eq DIPEA) fully diastereoselectively, as the *trans*- isomer (Scheme 1), although both anilide **7b** and quinoline **7c** did not.

**Scheme 3:**
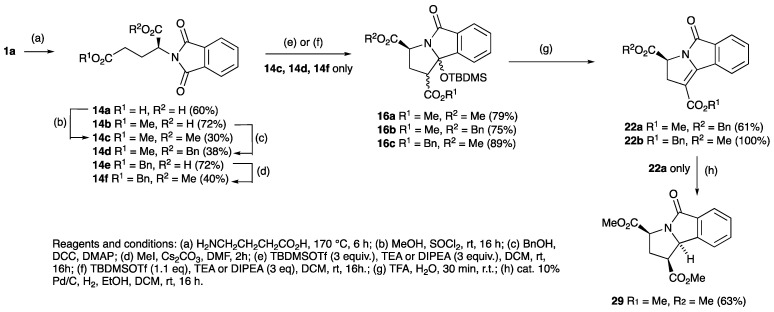
Synthesis and ring closure of glutamyl substituted phthalimides.

**Scheme 4:**
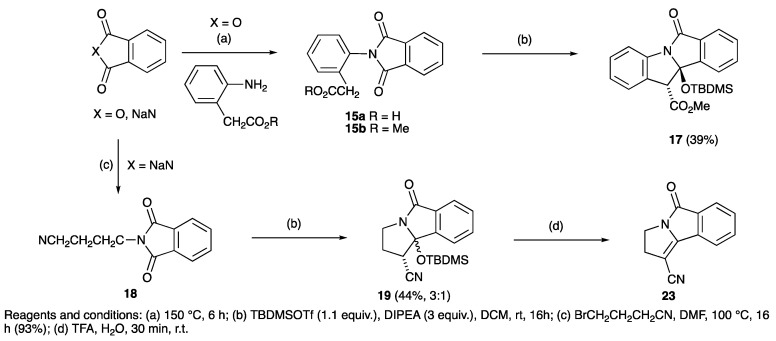
Synthesis and ring closure of substituted phthalimides.

The solventless phthalimide synthesis proved to be very effective with phthalic anhydride and glutamic acid, giving the desired diacid product **14a** in 60% yield (Scheme 3) [64,65,66]. However, this material was not easily soluble, but L-glutamic acid 5-methyl ester along with phthalic anhydride gave the desired and much more soluble product **14b** in 72% yield after heating at 175 °C for 6 h; this reaction remained effective on a multigram scale. In addition, L-glutamic dimethyl ester hydrochloride and phthalic anhydride under the same conditions gave the product **14c** in a yield of 30%; the same product could be prepared by esterification of diacid **14a** and with a similar yield (38%). This approach was similarly suitable for L-glutamic acid 5-benzyl ester, which gave the product **14d** with a yield of 38% [67,68]. The most effective method for the monomethyl esterification of benzyl glutamate **14e** used MeI, Cs_2_CO_3_, DMF, which gave the desired product **14f** in 40% yield. This approach could also be used for imide formation with phthalic anhydride and 2-aminophenylacetic acid via solventless conditions to give **15a**, followed by esterification which gave the desired ester **15b** with a yield of 38% (Scheme 4); however, a better alternative proved to be direct condensation of the methyl ester of aminophenylacetic acid to give **15b** and in quantitative yield. Nitrile **18**, was also readily available, prepared as shown (Scheme 4).

Cyclisation of these analogues was examined using the conditions optimised above. The L-dimethyl ester glutamic acid appended phthalimide **14c** cyclised in excellent yield of 79% to give **16a** as a diastereomeric mixture (Scheme 3); one of these was successfully crystallised and the structure for the major one determined by single crystal X-ray diffraction (Figure S1, ESI). This clearly shows that the two methyl esters are *cis*-related, with all substituents in a pseudoaxial-like arrangement [54,55,56,57]. The cyclisation of **14d** under the same conditions gave a single diastereomer of **16b**, most likely due to the greater steric hindrance of the two substituents, the structure of which was confirmed by single crystal X-ray diffraction (Figure S1, ESI) [54,55,56,57]. Benzyl glutamate **14f** was subjected to TBDMSOTf mediated cyclisations, but gave poor yields of **16c** of around 35% when using 1.1 eq of TBDMSOTf, although this improved to much higher yields (89%) with 3.0 eq of TBDMSOTf, as a mixture of inseparable diastereomers (*d.r* of 1:0.6), the major of which was assumed to have the same stereochemistry as **16a**, based on comparison to established NMR spectroscopic data. When compound **15b** (Scheme 4) was subjected to standard cyclisation conditions, product **17** was successfully obtained as a single stereoisomer in 39% yield. Of interest is that nitrile **18** also readily cyclised to the analogous product **19** as a mixture of diastereomers; a related material has previously been reported by photocyclisation [69].

**Scheme 5:**
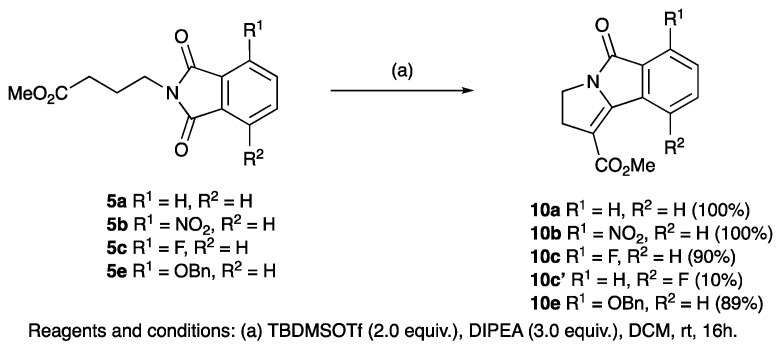
One-pot ring closure and elimination of substituted phthalimides.

Since TFA elimination reactions had been previously reported on silyloxyethers [60,70,71,72,73], similar reactions were then carried on **8a–d** and **12a–e, 13a–e**, giving quantitative conversions to the eliminated products **10a–d**, **20a–e**, **21a–e, 22a–b** (Scheme 1, Scheme 2, Scheme 3 and Scheme 4). When the diastereomeric mixture of **19** was stirred in TFA/H_2_O (9:1) for 30 min, only the *trans-* isomer reacted, leaving the *cis-*isomer unconverted, consistent with a fast antiperiplanar elimination; the structure of the unsaturated product **23** was confirmed by single crystal X-ray diffraction (Scheme 4 and Figure S2, (SI)) [54,55,56,57]. Moreover, it was found that the eliminated cyclised products could also be obtained directly by using TBDMSOTf (2 equiv.) for the ring closure reaction of both phthalimides **5a,b,c,e** and **14c,f** adducts and in excellent yield (Scheme 5).

**Figure 1:**
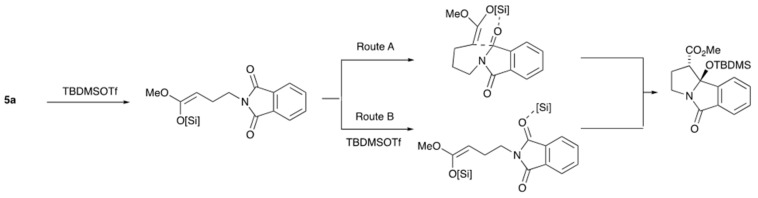
Possible mechanism for aldol ring closure.

Hoye described the mechanism of this ring-closing process as an intramolecular Mukaiyama-like addition in which formation of a silyl ketene acetal is followed by addition to one of the imide carbonyls via in situ silyl activation [45], and Christmann proposed the intermediacy of a *bis*-silylketene acetal formed in situ from the starting carboxylic acid [46]. Although the Mukaiyama aldol addition [74] is very well known [75,76,77,78,79,80], Mukaiyama-type additions to imides are not; however, a one-pot approach, in which silyl ketene acetals are intermediates, has been described for addition to imines [81]. We propose a similar mechanism for a Mukaiyama-imide aldol addition involving the formation of the silyl ketene acetal followed either by coordination of the imide carbonyl giving a 5,6-bicyclic transition state that undergoes aldol addition (Route A), or cyclisation involving separate imide activation by a second molecule of TBDMSOTf (Route B) (Figure 1).

**Scheme 6:**
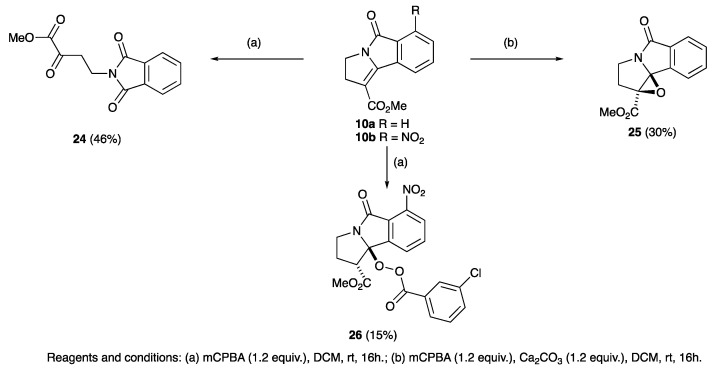
Elaboration of 2,3-dihydro-5*H*-pyrrolo[2,1-*a*]isoindol-5-ones.

**Scheme 7:**
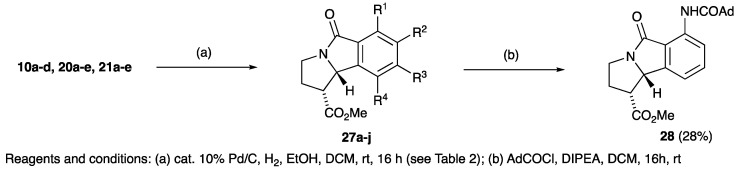
Reduction of 2,3-dihydro-5*H*-pyrrolo[2,1-*a*]isoindol-5-ones.

With the pyrrolidinones in hand, of interest was an examination of their further reactivity; it was expected that this might not be straightforward, since low solubility was found for many compounds, especially the planar derivatives such as **10, 20** and **21**. Additionally, the high level of cross-conjugation along with significant steric effects in these densely functionalised systems was expected to significantly modify their chemical behaviour. Functionalisation of the (electron deficient) carbon-carbon double bond of the unsaturated system of cyclised adducts, for which there was some precedent literature [82], was examined [83] using 35% aqueous hydrogen peroxide in the presence of 4-methyl morpholine. The unsubstituted variant **10a** proved to be unreactive under these conditions, although when dissolved in dichloromethane with *m*CPBA and left stirring for 16 h at room temperature, α-ketoester **24** was obtained in low yield (Scheme 6). Such a product would be expected to arise by initial epoxidation of the double bond, followed by a further attack by *m*CPBA leading to a ring opening. However, it was found that if this reaction was conducted in the presence of calcium carbonate, successful epoxidation was achieved, giving **25**. This approach proved not to be successful for **10b**, since 1.2 equivalents of *m*CPBA gave not the expected epoxide but adduct **26** (Scheme 6), whose structure was confirmed by careful NMR spectroscopic analysis. Catalytic hydrogenation gave highly efficient conversion of lactams **10a–d, 20a–e, 21a–e** to lactams **27a–j**, in a reaction in which the strong yellow colour of the starting material was fully discharged, consistent with the removal of the extended conjugation (Table 2 and Scheme 7). In the case of the nitro derivatives **10b, 20a,** and **21a**, concomitant reduction to the amine derivatives **27b–d** occurred. The structures of **27b** and **27f** were confirmed by single crystal X-ray diffraction (Figure S2, ESI) [54,55,56,57]. Reduction of **10d** also involved hydrogenolysis and afforded phenol **27h**. Glutamate derivatives **22a,b** were subjected to the same hydrogenation conditions and gave *cis-*dimethyl esters **29a,b** (Scheme 3), whose stereochemistry was shown from nOe analysis.

**Scheme 8:**
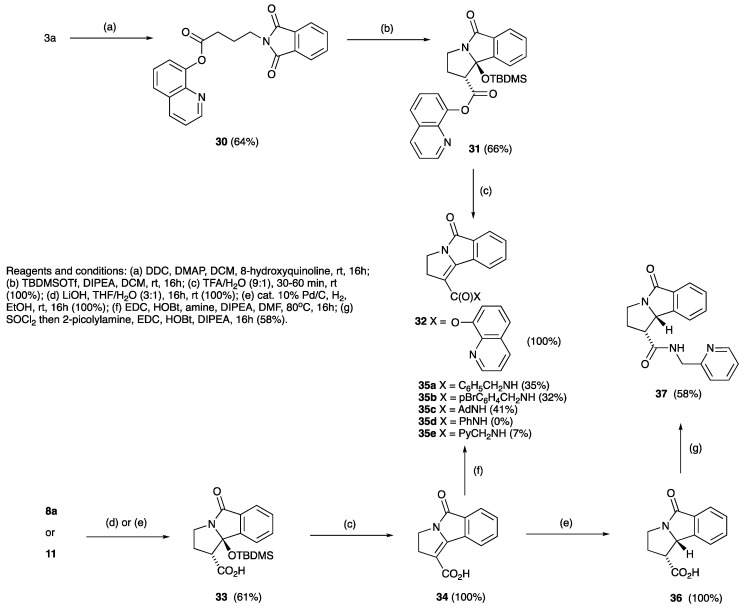
Elaboration of lactam systems.

With **27b** in hand, conversion to corresponding amide **28** using 1-adamantanecarbonyl chloride (Scheme 8) was made, as this group had given some of the highest levels of antibacterial activity seen for tetramates [19]; although this reaction proceeded successfully, the yield was low (28%), and this most likely arose by the combination of an electronically and sterically deactivated amine with a hindered acid chloride.

Of interest was whether this approach might be able to be adjusted to allow the ready introduction of ring substituents on the core skeleton, including C-H functionalisation, since related systems had been shown to be amenable to such manipulation [84]. Since the use of 8-aminoquinoline as a directing group for C-H activation is now well-known [85,86,87], 8-hydroxyquinoline ester **30** was prepared via *N*,*N’*-dicyclohexylcarbodiimide coupling with 8-hydroxyquinoline (64%), and although this could be effectively cyclised to **31** under standard conditions, in subsequent reactions **31** did not undergo remote C-H arylation. However, **31** when treated with TFA:H_2_O afforded the desired unsaturated system **32** quantitatively (Scheme 8). While ester hydrolysis of **8a** and **11** was found to be straightforward, giving acids **33** and **34**, the attempted DCC/DMAP coupling of **33** with 8-aminoquinoline proved to be unsuccessful, giving only the rearranged N-acylurea intermediate; 1-ethyl-3-(3-dimethylaminopropyl)carbodiimide (EDC) along with 1-hydroxybenzotriazole (HOBt) gave a similar outcome. It was also found that **10a** could be hydrolysed directly under basic conditions in excellent yield to give **33** directly, and that this, when treated with TFA, water, and methanol, gave acid **34** in quantitative yield (Scheme 8). With **34** in hand, amide formation was examined (Scheme 8) but the products **35a–c** could be obtained only in modest yield, and aniline was completely unreactive. This likely reflects the unusual electronic character of the extended conjugated push-pull system in the starting material. Alternatively, **36** could be obtained by direct reduction of acid **34** or by hydrolysis of **27a** in good yield (Scheme 8) and conversion to the picolyl amide **37** under a variety of conditions gave a modest yield of product.

## 3. Bioassays

The compounds were tested using a primary 96-well plate screening assay against MRSA (Gram+) and *E. coli* (Gram−) bacterial strains and MIC values and along with the calculated molecular weights, ClogP, tPSA along with HBD and HBA (Table S2, ESI). The only systems showing activity were **12e/13e, 31,** and **32**. This probably reflects the high level of hydrophobicity of the silyloxy ethers and the particularly low solubility of the unsaturated systems, even though their cheminformatic descriptors are broadly desirable. This outcome suggests that some fragments might be suitable for further elaboration to identify better antibacterial activity. Some compounds were also tested for cytotoxic activity against four different cell lines: HeLa, HEK 293, CaCo, and MDCK (Table S3 (SI)). Nearly all the compounds that were tested showed some weak activity, but **35c** was found to be moderately active against HeLa and HEK 293 with lesser activity against CaCo and MDCK. Once again, the low solubility of these compounds under assay conditions is likely to be an important limitation of this compound class.

## 4. Materials and Methods

Full experimental details are provided in the Supplementary Materials File S1.

## 5. Conclusions

We have shown that phthalimides may be effectively cyclized using a Mukaiyama-type aldol coupling leading to variously substituted fused lactam (1,2,3,9b-tetrahydro-5*H*-pyrrolo[2,1-*a*]isoindol-5-one) systems. This novel process shows a high level of regioselectivity for *o*-substituted phthalimides, dictated by steric and electronic factors, but not for *m*-substituted phthalimides. The initial aldol adduct is prone to elimination, and the cyclisation can be conducted in such a way that aldol cyclisation-elimination is achievable in one pot. The eliminated skeletal systems (2,3-dihydro-5*H*-pyrrolo[2,1-*a*]isoindol-5-one) possess cross-conjugation and steric effects which significantly influence the reactivity of several functional groups, but conditions suitable for epoxidation, ester hydrolysis and amide formation, and reduction, were identified. Many of the derived lactam systems, and especially the eliminated systems, show low solubility, which compromises biological activity, although in some cases, antibacterial and cytotoxic activity was found and this new class of small molecule provides a useful skeleton for further elaboration and study. We have earlier shown that a core bicyclic tetramate displays no intrinsic antibacterial activity [26], but that this can be restored after appropriate heterocyclic ring substitution [19]. The work herein shows that the core tetrahydro-5*H*-pyrrolo[2,1-*a*]isoindol-5-one system is now synthetically readily available, and further investigation is needed to develop the understanding of both its medicinal chemistry and biological activity.

## Figures and Tables

**Table 1:** Yields for Phthalimides **3,4**, Esters **5,6** and Lactams **8, 12** and **13** (Scheme 1 and Scheme 2).

R	Phthalimide	Temperature (°C)	Yield (%)	Ester	Yield (%)	Lactam	Yield (%)
H	**3a**	170	100	**5a**	100	**8a**	97
NO_2_	**3b**	175	93	**5b**	80	**8b**	82
F	**3c**	170	100	**5c**	100	**8c/9**	61 *
OH	**3d**	195	92	**5d**	96	-	-
OBn	-	-	-	**5e**	73	**8d**	70
OMe	-	-	-	**5f**	100	**8e**	87
NO_2_	**4a**	175	100	**6a**	100	**12a,13a**	72 *
Br	**4b**	170	84	**6b**	0 (100)	**12b,13b**	55 *
F	**4c**	170	100	**6c**	100	**12c,13c**	55 *
CO_2_H	**4d**	205	74	-	-	-	-
CO_2_Me	-	-	-	**6d**	62	**12d,13d**	92 *
CH_3_	**4e**	170	100	**6e**	100	**12e,13e**	85 *

* Yields are the total for both isomers.

**Table 2:** Reduction of unsaturated bicyclic lactams **10a**–**d**, **20a**–**e**, **21a**–**e**.

Substrate	Product	R^1^	R^2^	R^3^	R^4^	Yield (%)
**10a**	**27a**	H	H	H	H	100
**10b**	**27b**	NH_2_	H	H	H	100
**20a**	**27c**	H	NH_2_	H	H	100
**21a**	**27d**	H	H	NH_2_	H	100
**10c**	**27e**	F	H	H	H	50
**10c’**	**27f**	H	H	H	F	14
**20c**	**27g**	H	F	H	H	100
**10d**	**27h**	OH	H	H	H	86
**20e**	**27i**	H	Me	H	H	95
**21e**	**27j**	H	H	Me	H	

## Supplementary Materials

The following supporting information can be downloaded at: https://www.mdpi.com/article/10.3390/antibiotics12010009/s1. File S1: Supporting Information (SI). References [88,89,90,91,92,93,94,95,96] occur only in the supplementary materials.
