# Supplementary material for: Tricyclic Fused Lactams by Mukaiyama Cyclisation of Phthalimides and Evaluation of their Biological Activity
Source: Antibiotics (Basel). 2022 Dec 21;12(1):9. doi: 10.3390/antibiotics12010009 (PMC9854654; doi:10.3390/antibiotics12010009)
Supplement: Supplementary file 1 [file antibiotics-12-00009-s001.zip › antibiotics-2088954-supplementary.pdf]

Supporting Information

Tricyclic Fused Lactams by Mukaiyama Cyclisation of Phthalimides and Evaluation of their Biological Activity

Lewis T. Ibbotson,† Kirsten E. Christensen,† Miroslav Genov,‡ Alexander Pretsch,‡ Dagmar Pretsch,‡ and Mark G. Moloney*,†,#

†The Department of Chemistry, Chemistry Research Laboratory, University of Oxford, 12 Mansfield Road, Oxford. OX1 3TA

#Oxford Suzhou Centre for Advanced Research, Building A, 388 Ruo Shui Road, Suzhou Industrial Park, Jiangsu, 215123, P.R. China.

‡Oxford Antibiotic Group, The Oxford Science Park, Magdalen Centre, Oxford OX4 4GA, UK.

mark.moloney@chem.ox.ac.uk

Table of Contents

Figure S1: Single crystal X-ray structures **S-2**

Figure S2: Single crystal X-ray structures **S-3**

Table S1: Reaction Conditions for the cyclisation of Phthalimides 5a,b to Lactams 8a,b (Scheme 1). **S-4**

Table S2: MIC against against MRSA and *E. coli* and selected cheminformatic data. **S-4**

Table S3. Cytotoxicity of selected compounds against HeLa, HEK 293, CaCo, MDCK cell lines. **S-8**

Experimental procedures and data **S-9**

References **S-65**

NMR SPECTRA **S-66**

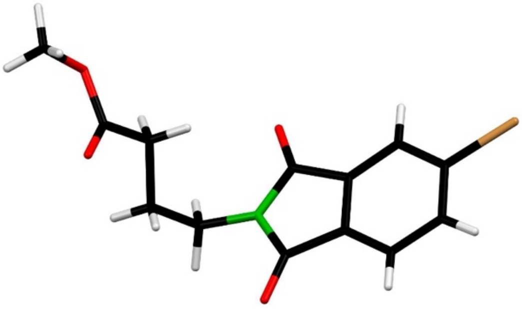

6b

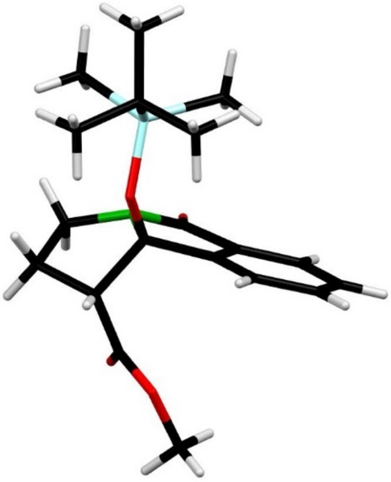

8a

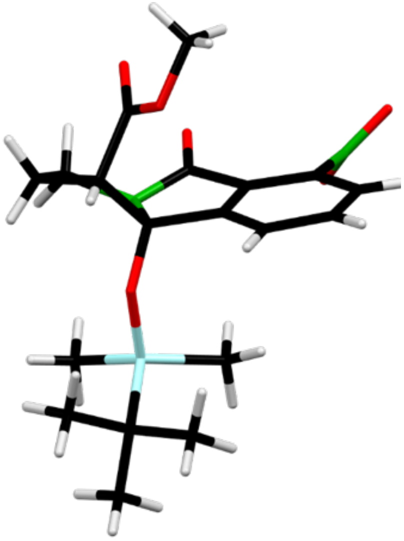

8b

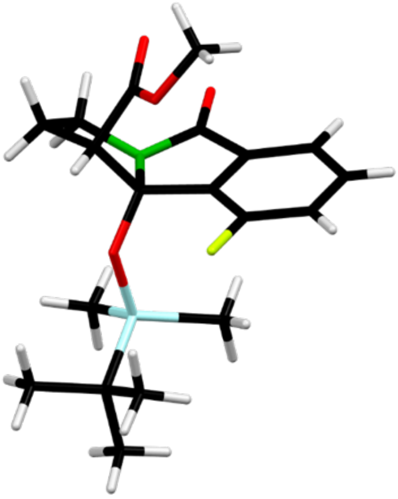

9

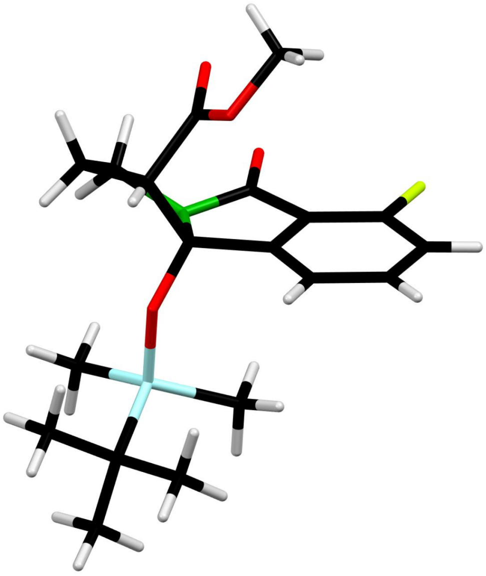

8c

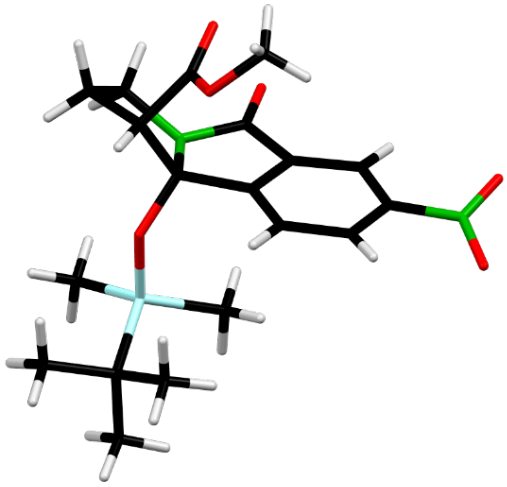

12a

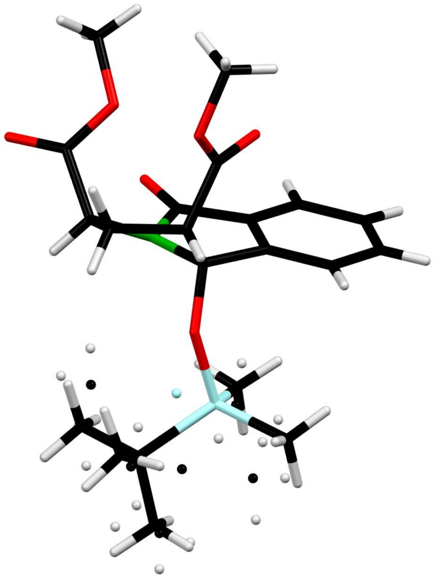

21

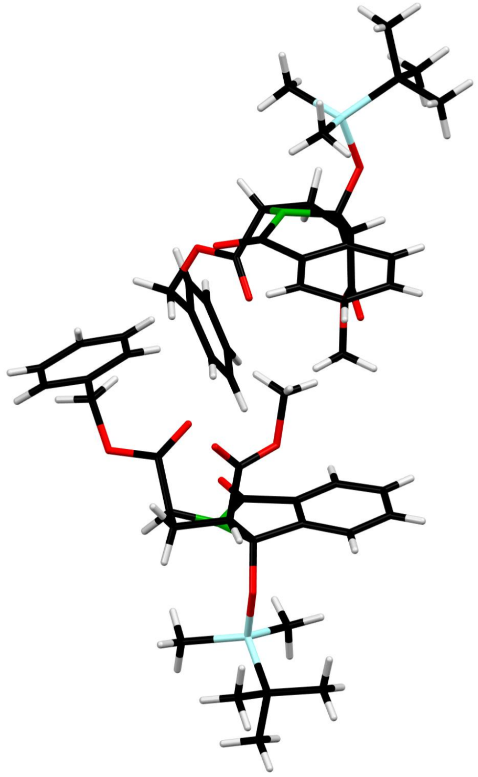

16b

Figure S1: Single crystal X-ray structures

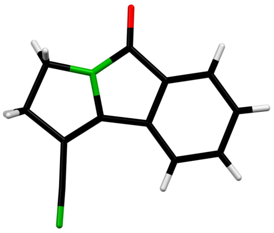

23

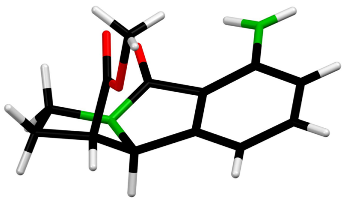

27b

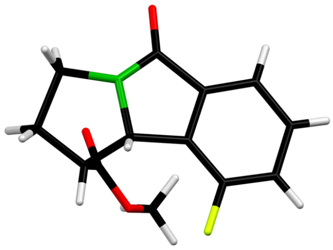

27f

Figure S2: Single crystal X-ray structures

Table S1: Reaction Conditions for the cyclisation of Phthalimides **5a,b** to Lactams **8a,b** (Scheme 1).

**Phthalimide**	**Conditions**	**Product Yield (%)**	
	**TBDMSOTf**(equiv.)	**8a or b**	**10a or b**
**5a**	1.0	78	0
**5a**	1.1	97	0
**5a**	2.0	0	100
**5b**	1.0	64	0
**5b**	1.1	82	0
**5b**	2.0	0	100

Table S2. MIC against against MRSA and *E. coli* and selected cheminformatic data.

	Compound	MRSA (μg/mL)	*E.coli* (μg/mL)	Mw	ClogP	tPSA	logS	HBD	HBA
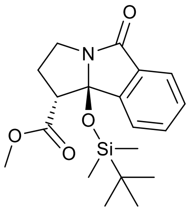	**8a**	n.a	n.a	361.1	3.401	55.84	-4.00	0	6
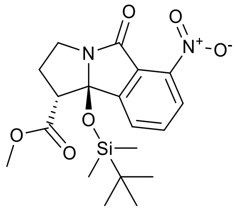	**8b**	n.a	n.a	406.1	3.306	107.65	-4.36	0	11
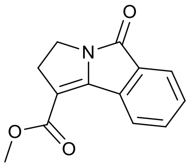	**10a**	n.a	n.a	229.0	0.775	46.61	-2.53	0	4
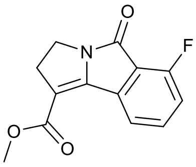	**10c**	n.a	n.a	247.0	0.994	46.61	-2.77	0	4
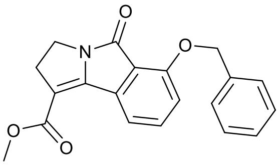	**10d**	n.a	n.a	335.1	2.763	55.84	-4.40	0	6

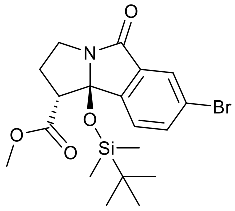	**12b**	n.a	n.a	439.0	4.339	55.84	-4.80	0	6
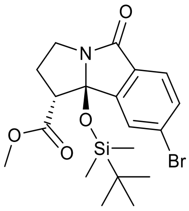	**13b**	n.a	n.a	439.0	4.339	55.84	-4.80	0	6
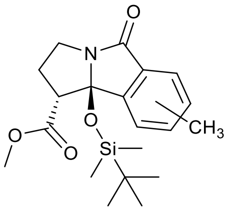	**12e,13e**	125	n.a	375.1	3.900	55.84	-4.35	0	6
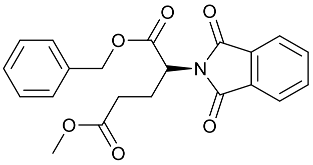	**14d**	n.a	n.a	381.1	2.780	89.98	4.27	0	8
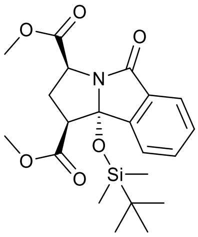	**16a**	n.a	n.a	419.1	3.428	82.14	-4.10	0	8
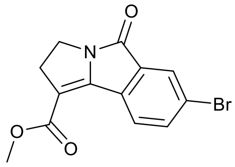	**20b**	n.a	n.a	306.9	1.714	46.61	-3.34	0	4
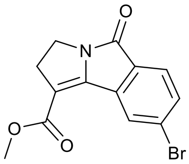	**21b**	n.a	n.a	306.9	1.714	46.61	-3.34	0	4
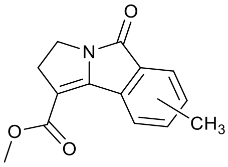	**20e, 21e**	n.a	n.a	243.0	1.274	46.61	-2.89	0	4
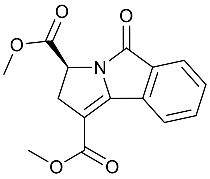	**22a**	n.a	n.a	287.0	0.853	72.91	-2.64	0	6

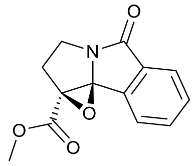	**25**	n.a	n.a	245.0	0.229	59.14	-1.72	0	6
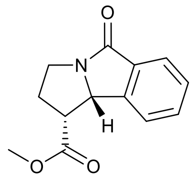	**27a**	n.a	n.a	231.0	0.975	46.61	-2.15	0	4
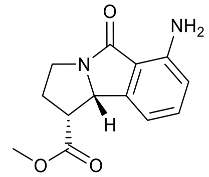	**27b**	n.a	n.a	246.1	0.258	72.63	-1.87	1	5
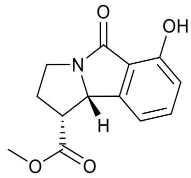	**27h**	n.a	n.a	247.0	0.849	66.84	-1.82	1	6
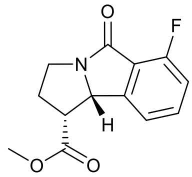	**27e**	n.a	n.a	249.0	1.194	46.61	-2.39	0	4
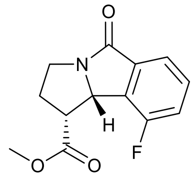	**27f**	n.a	n.a	249.0	1.194	46.61	-2.37	0	4
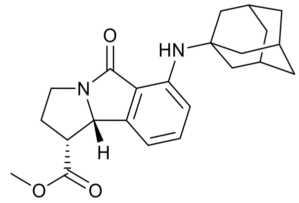	**28**	n.a	n.a	380.2	3.684	58.64	-4.87	1	5
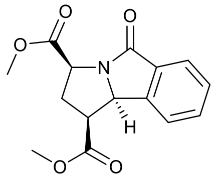	**29**	n.a	n.a	289.1	1.002	72.91	-2.26	0	6
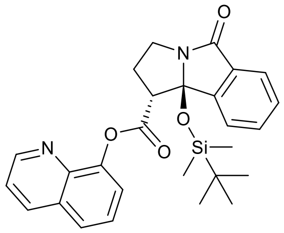	**31**	125	125	474.2	4.906	68.20	-6.36	0	7

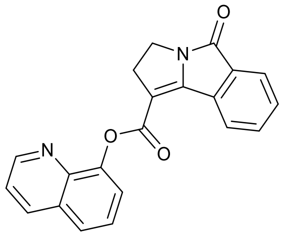	**32**	31.25	125	342.1	2.539	58.97	-4.92	0	5
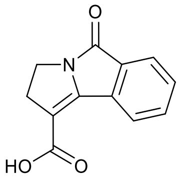	**34**	n.a	n.a	215.0	0.427	57.61	-2.51	1	6
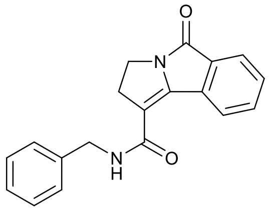	**35a**	n.a	n.a	304.1	1.989	49.41	-4.01	1	4
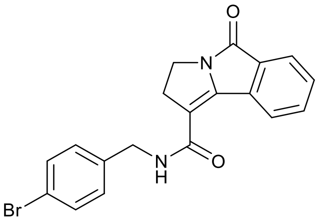	**35b**	n.a	n.a	382.0	2.852	49.41	-4.83	1	4
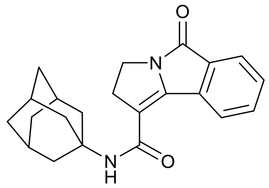	**35c**	n.a	n.a	348.1	2.926	49.41	-4.96	1	4
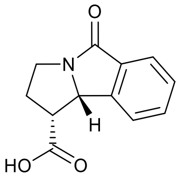	**36**	n.a	n.a	217.0	0.512	57.61	-1.77	1	6
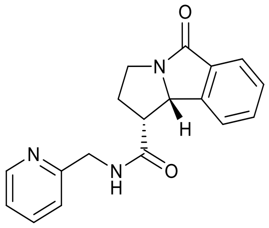	**37**	n.a	n.a	307.1	0.430	61.77	-2.91	1	5

n.a. = not active

Table S3. Cytotoxicity of selected compounds against HeLa, HEK 293, CaCo, MDCK cell lines.

**Compound Structure**	**Compound**	**HeLa**	**HEK 293**	**CaCo**	**MDCK**
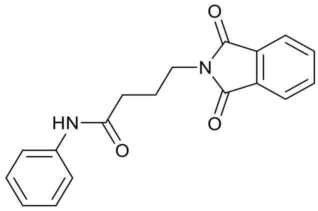	**7b**	125	62.5	125	125
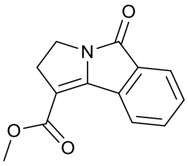	**10a**	62.5	62.5	62.5	62.5
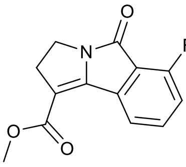	**10c**	62.5	31.2	62.5	62.5
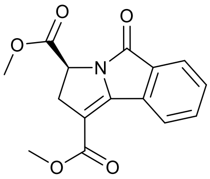	**22a**	62.5	62.5	125	125
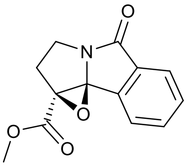	**25**	62.5	62.5	62.5	62.5
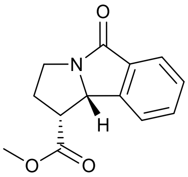	**27a**	62.5	62.5	62.5	62.5
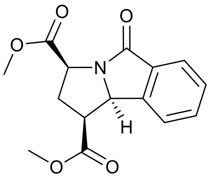	**29a**	62.5	62.5	62.5	62.5
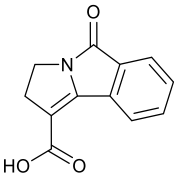	**34**	62.5	62.5	125	125

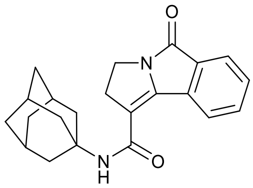	**35c**	15.6	15.6	31.2	31.2
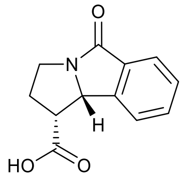	**36**	31.2	62.5	62.5	125

Materials and Methods
Experimental

General techniques

All reactions were performed in oven-dried glassware and under a N_2 atmosphere, unless non-anhydrous solvents were used. Anhydrous solvents were obtained from MBraun MB SPS5solvent purification system prior to use. Reactions that were concentrated, were done so with the water bath set to 40 °C while under reduced pressure using a Büchi R-114 rotatory evaporator that was attached to a Vacuubrand CVC2 pump with a pressure control system attached. When compounds were dried using reduced pressure, the water bath was set to 40 °C with the pump set to continuous, typically between 0-5 mb. Analytical thin layer chromatography (TLC) was conducted using Merck aluminium foil backed sheets coated with 0.2 mm Kielselgel 60 F_{254}. The eluent used is specified for each compound purified using flash column chromatography. TLC spots were visualised by UV irradiation (λ 254 and 366 nm) and where necessary, staining with ninhydrin or phosphomolybdic acid (PMA) solution followed by heating by using a heat gun. Iodine chamber was also used when applicable. Flash column chromatography was performed on Kielselgel 60 silica gel (230-400 mesh size). Optical rotations were recorded at 25 °C on a Perkin-Elmer 421 polarimeter using the D line of sodium (589) and a path length of 1 dm. Concentrations (c) are reported in g/100 mL and specific rotations ($[\alpha]_D^{25}$) are quoted in 10^{-1} deg cm^2 g^{-1}. Melting points (**Mp**) were recorded on a Stuart Scientific SMP1 melting point instrument and are uncorrected. Infrared spectra (**IR**) were recorded using a Bruker Tensor 27 FT-IR spectrometer equipped with an attached Pike Miracle attenuated total reflectance (ATR) module. Absorption maxima (**ν_{max}**) are reported in wavenumbers (cm^{-1}) with selected characteristic peaks assigned where possible. **^{1}H NMR** spectra were recorded on any of the following instruments; Brucker DPX200 (200 MHz), AVIII HD 400 (400 MHz), AVC 500 (500 MHz), AV600 (600 MHz) and AV700 (700 MHz). Deuterated solvents used for analysis were chloroform, methanol, dimethyl sulfoxide and water. Two-dimensional COSY and HSQC were recorded on a Bruker AVIII HD 400 (400 MHz) and AVC 500 (500 MHz). HMBC, NOESY and 1D nOe were recorded on AVC 500 (500 MHz). **^{13}C NMR** spectra were recorded in a Bruker AVIII HD400 at 101 MHz, AVC 500

at 125 MHz and AV600 at 150 MHz with a proton decoupling. Chemical shifts (δ_c) are reported in ppm downfield from TMS. Deuterated solvents used for analysis were chloroform, methanol, dimethyl sulfoxide and water. Assignments of the spectra were made with HSQC experiments, which was performed on a Brucker AVIII 400 HD. Low resolution mass spectra (***m/z***) were recorded on a Fison Platform spectrometer using electrospray ionisation (ESI) with both positive and negative ESI reported when possible. High resolution mass spectra (**HRMS**) were recorded on a Bruker microTOF (ESI), on an Agilent 7200 Q-TOF (CI) or on a Waters GCT (EI) with the predicted and observed mass ion reported to four decimal places. Crystals for x-ray crystallography were grown from slow vapour diffusion at room temperature of petroleum either 40:60 into a solution of the requisite compound in either $CDCl_3$ or DCM. Low temperature single crystal X-ray diffraction data were collected using a Rigaku Oxford SuperNova diffractometer. Raw frame data were reduced using CrysAlisPro. Full refinement details are given in the Supporting Information (CIF).

Bioassays

Screening of compounds was performed by Oxford Antibiotic Group, Austria. For MIC determination by broth dilution assay, the samples were tested in a primary 96 well plate screening assay. The compounds were diluted in Mueller Hinton Broth (MHB) for bacterial screening to a stock solution of 1.0 mg/mL, serial diluted and overlaid with a microbe solution in a concentration of 104 CFU/ml. The plates were incubated for 24 h at 35 °C, after which MIC values were read from the plates. For cytotoxicity testing, the synthesised compounds were tested against four different cell lines: HeLa, HEK 293, MDCK and CaCo. The cells were seeded in a 96 well plate and incubated until a confluence of 80% was achieved (under physiological conditions – 37 °C, 5% CO_2 and 95% humidity). The samples were tested by serial dilution in triplicates with starting concentration of 250 µg/mL. After 24 and 48 hours the survival of cells was evaluated by microscope and measured with Alamar blue. IC_{50} values were obtained from the calibration curves.

General Procedure A (Phthalimide formation)

Phthalic anhydride (1.0 eq) was added to 4-aminobutanoic acid (1.0 eq) and heated at 170 °C without solvent for 6 hours while stirring. The reaction was left to cool to room temperature with the resulting solid mass dissolved in DCM (10 mL). The organic layer was washed using 0.5 N HCl (2 x 10 mL) with the combined aqueous layers being back extracted with DCM (20 mL). The organic layers were then combined and concentrated by reduced pressure to afford the desired phthalimide.

General Procedure B (Esterification).

Starting material was dissolved in MeOH (10 mL) and cooled to 0 °C with an ice bath. Thionyl chloride (3.0 eq) was then added dropwise over 5 minutes and the mixture left stirring for 16 hours at room temperature. The solvent was removed by reduced pressure then azeotroped using DCM (3 x 10 mL) and purification *via* flash column chromatography afforded the desired ester.

General Procedure C (Lactamisation).

In an oven-dried round bottomed flask that had been purged with N_2, imide (1.0 eq) was dissolved in anhydrous DCM (5 mL) followed by the addition of DIPEA (3.0 eq) and left to stir at room temperature for 1 hour. The reaction mixture was cooled to 0 °C using an ice bath with TBDMSiOTf (1.1 eq) being added dropwise over 5 minutes. The ice bath was removed with the reaction mixture allowed to warm to room temperature and left stirring for 16 hours. The crude mixture was transferred to a separating funnel and diluted with DCM (10 mL) then washed using water (2 x 10 mL) with the aqueous layers being combined and back extracted using DCM (10 mL). The combined organic layers were then dried using $MgSO_4$ and concentrated by reduced pressure. The crude oil was then purified using flash column chromatography with the product containing fractions collected, combined and concentrated to afford the desired product.

General Procedure D (Elimination).

Silyl derivative was dissolved in a solution of TFA/H_2O (9:1, 1 mL) and left stirring at room temperature for 1 hour, during which the reaction mixture turned yellow. The solvent was removed using reduced pressure to afford the desired product without any additional purification.

General Procedure E (Hydrogenation)

In an oven-dried round bottomed flask that had been purged with N_2, compound was added followed by a catalytic quantity of 10% Pd/C and suspended in ethanol (15 mL), with DCM being added dropwise to encourage solubility of the starting material. The flask was sealed and purged using N_2 (3x) and charged with H_2 (2x). The reaction was then fitted with two balloons pressurised with H_2 with the reaction was left stirring at room temperature for 16 hours. The reaction flask was then degassed and purged using N_2. The solvent was then filtered through a pad of Kieselguhr under pressure. The pad of Kieselguhr was then washed using methanol (15 mL) with the combined organic solvents being concentration by reduced pressure to afford the desired product.

4-(6,13-Dioxoisoindolin-5-yl)butanoic acid 3a

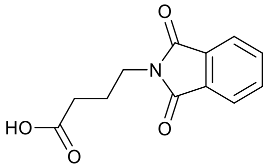

123a

3a was synthesised according to General Procedure **A** using phthalic anhydride (0.500 g, 3.37 mmol) and 4-aminobutanoic acid (0.435 g, 3.37 mmol) to afford the desired product as colourless solid (0.793 g, quantitative yield). **Mp**= 112-114 °C (lit.[1] 117 °C). **ν_{max} (KBr) cm^{-1}** 1765 (C=O), 1698 (N-C=O), 1467 (C=C), 1357. **^{1}H NMR** (400 MHz, Chloroform-*d*) δ 7.85 (dd, *J* = 5.4, 3.1 Hz, 2H, Ar-H), 7.72 (dd, *J* = 5.4, 3.0 Hz, 2H, Ar-H), 3.77 (t, *J* = 6.8 Hz, 2H, C(4)*H*), 2.42 (t, *J* = 7.4 Hz, 2H, C(2)*H*), 2.02 (m, 2H, C(3)*H*). **^{13}C NMR** (101 MHz, Chloroform-*d*) δ 177.4 (COOH), 168.4 (C=O), 134.0 (ArC),132.0 (ArC), 123.3 (C(7,12)ArC), 37.0 (C(4)), 31.1 (C(2)), 23.6 (C(3)). ***m/z***= 398 [2M-H] (ESI^{+}). **HMRS** calculate $C_{12}H_{10}NO_4$ requires 232.0615, found 232.0615 [MH^{-}] (ESI^{-}).

4-(8-Nitro-6,13-dioxoisoindolin-1-yl)butanoic acid 3b

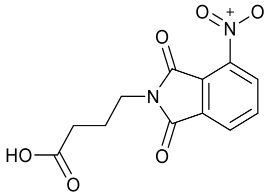

123b

3b was synthesised according to General Procedure **A** using 3-nitrophthalic anhydride (0.500 g, 2.58 mmol) and 4-aminobutanoic acid (0.267 g, 2.58 mmol) to afford the desired product as an colourless solid (0.692 g, 93%).**Mp**= 118-120 °C (Lit.[2] 132-133 °C). **ν_{max} (KBr) cm^{-1}** 1693 (N-C=O), 1539 (N=O), 1396, 1357 (N=O). **^{1}H NMR** (400 MHz, Chloroform-*d*) δ 8.21 – 8.08 (m, 2H, Ar-H), 7.99 (dd, *J* = 8.1, 7.5 Hz, 1H, Ar-H), 3.76 (t, *J* = 6.8 Hz, 2H, C(4)*H*, 2.38 (t, *J* = 7.2 Hz, 2H, C(2)*H*, 1.98 (m, 2H, C(3)*H*. **^{13}C NMR** (101 MHz, $CDCl_3$) δ 176.4 (COOH), 167.6 (C=O), 164.84 (C=O), 136.8 (**C**-NO_2), 135.4 (C(7,12)ArC), 129.3 (ArC), 127.6 (ArC), 38.9 (C(4)), 32.1 (C(2)), 24.6 (C(3)). ***m/z*** = 301 [MNa^{+}] (ESI^{+}) and 277 [MH^{-}] (ESI^{-}). **HRMS** calculated for $C_{12}H_{10}N_2NaO_6$ requires 301.0431, found 301.0430 [MNa^{+}] (ESI^{+}).

4-(8-Fluoro-6,13-dioxoisoindolin-1-yl)butanoic acid 3c[3]

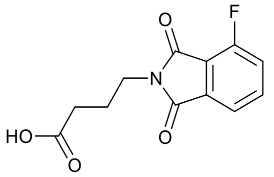

123f

3c was synthesised according to General Procedure **A** using 3-fluorophthalic anhydride (0.250 g, 1.50 mmol) and 4-aminobutanoic acid (0.155 g, 1.50 mmol) to afford the desired product as a light coloured brown solid (0.378 g, quantitive yield). **Mp**= 125-126 °C. **ν_{max} (KBr) cm^{-1}** 2980 (OH), 1708 (N-C=O). **^{1}H NMR** (400 MHz, Chloroform-*d*) δ 7.80 – 7.63 (m, 2H, Ar-H), 7.39 (ddd, *J* = 9.1, 7.6, 1.6 Hz, 1H, Ar-H), 3.77 (t, *J* = 6.8 Hz, 2H, C(4)*H*), 2.44 (t, *J* = 7.4 Hz, 2H, C(2)*H*), 2.02 (m, 2H, C(3)*H*). **^{13}C NMR** (101 MHz, Chloroform-*d*) δ 178.61 (COOH), 167.32 (d, *J* = 3.1 Hz, C=O), 165.17 (C=O), 157.48 (d, *J* = 264.8 Hz, Ar**C**-F), 136.78 (d, *J* = 7.8 Hz, ArC), 134.28 (ArC), 122.56 (d, *J* = 19.8 Hz, ArC), 119.68 (d, *J* = 3.5 Hz, ArC), 117.67 (d, *J* = 12.7 Hz, ArC), 37.34 (C(3)), 31.32 (C(1)), 23.57 (C(2)). ***m/z***= 250 [MH-] (ESI$^-$). **HRMS** calculated for $C_{12}H_{10}FNNaO_4$ requires 274.0486, found 274.0484 [MNa+] (ESI$^+$).

4-(8-Hydroxy-6,13-dioxoisoindolin-1-yl)butanoic acid 3d

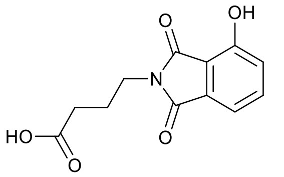

123d

3d was synthesised according to General Procedure **A** using 3-hydroxyphthalic anhydride (0.900 g, 5.48 mmol) and 4-aminobutanoic acid (0.565 g, 5.48 mmol) to afford the desired product as a brown coloured solid (1.452g, 92%). **Mp**= 189-191 °C. **ν_{max} (KBr) cm^{-1}** 1684 (N-C=O), 1614 (C=O). **^{1}H NMR** (400 MHz, Chloroform-d) δ 7.59 (dd, *J* = 8.4, 7.2 Hz, 1H, Ar-H), 7.31 (dd, *J* = 7.2, 0.8 Hz, 1H, Ar-H), 7.14 (dd, *J* = 8.4, 0.8 Hz, 1H, Ar-H), 3.68 (t, *J* = 6.8 Hz, 2H, C(4)*H*), 2.35 (t, *J* = 7.2 Hz, 2H, C(2)*H*), 1.94 (m, 2H, (C(3)*H*). **^{13}C NMR** (101 MHz, Methanol-d4) δ 176.52 (COOH), 169.79 (C=O), 169.48 (C=O), 156.38 (ArC), 137.02 (ArC), 134.80 (ArC), 124.11 (ArC), 115.64 (ArC), 37.96 (C(4)), 32.20 (C(2)), 24.91 (C(3)). ***m/z***= 272 [MNa+] (ESI$^+$). **HRMS** calculated for $C_{12}H_{11}NNaO_5$ requires 272.0529, found 272.0529 [MNa+] (ESI$^+$).

4-(9-Nitro-6,13-dioxoisoindolin-1-yl)butanoic acid 4a

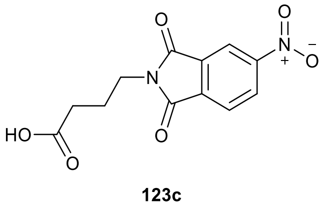

123c

4a was synthesised according to General Procedure **A** using 4-nitrophthalic anhydride (0.500 g, 2.58 mmol) and 4-aminobutanoic acid (0.267 g, 2.58 mmol) to afford the desired product as a beige coloured solid (0.698 g, quantitative yield). **Mp**= 164-166 °C (Lit.[4] 165-166 °C). **ν_{max} (KBr) cm^{-1}** 1700 (N-C=O), 1541 (N=O), 1396 (N=O). **^{1}H NMR** (500 MHz, Methanol-d4) δ 8.65 (dd, *J* = 8.1,

2.0 Hz, 1H, Ar-H), 8.61 (d, *J* = 2.0 Hz, 1H, Ar-H), 8.08 (d, *J* = 8.1 Hz, 1H, Ar-H), 3.79 (t, *J* = 6.8 Hz, 2H, C(4)*H*), 2.38 (t, *J* = 7.2 Hz, 2H, C(2)*H*), 1.99 (m, 2H, C(3)*H*). **^{13}C NMR** (126 MHz, Methanol-d4) δ 176.47 (COOH), 167.98 (C=O), 167.75 (C=O), 153.22 (Ar**C**-NO_2), 137.96 (ArC), 134.92 (ArC), 130.38 (ArC), 125.39 (ArC), 119.10 (ArC), 38.87 (C(4)), 32.18 (C(2)), 24.69 (C(3)). ***m/z***= 277 [MH-] (ESI^-). **HRMS** calculated for $C_{12}H_9N_2O_6$ requires 277.0466, found 277.0459 [MH-] (ESI^-).

4-(9-Bromo-6,13-dioxoisoindolin-1-yl)butanoic acid 4b

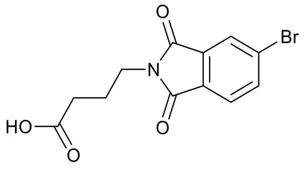

123e

4b was synthesised according to General Procedure **A** using 4-bromonphthalic anhydride (0.500 g, 2.21 mmol) and 4-aminobutanoic acid (0.228 g, 2.21 mmol) to afford the desired product as acolourless solid (0.58 g, 84%). **Mp**= 139-141 °C (Lit.[5] 103-106 °C). **ν_{max} (KBr) cm^{-1}** 1733 (C=O), 1685 (N-C=O). **^{1}H NMR** (400 MHz, DMSO-*d*$_6$) δ 8.02 – 7.93 (m, 2H, Ar-H), 7.78 – 7.70 (m, 1H, Ar-H), 3.58 (t, *J* = 6.8 Hz, 2H, C(4)*H)*, 2.26 (t, *J* = 7.2 Hz, 2H, C(2)*H*), 1.80 (m, 2H, C(3)*H*). **^{13}C NMR** (101 MHz, DMSO-*d*$_6$) δ 174.06 (COOH), 167.42 (C=O), 166.85 (C=O), 137.07 (ArC), 133.78 (ArC), 130.72 (ArC), 127.95 (Ar**C**-Br), 125.95 (ArC), 124.94 (ArC), 37.27 (C(4)), 31.07 (C(2)), 23.33 (C(3)). ***m/z***= 310 and 312 [MH-] (ESI^-). **HRMS** calculated for $C_{12}H_9BrNO_4$ requires 309.9720 and 311.9718, found 309.9718 and 311.9698 [MH-] (ESI^-).

4-(9-Fluoro-6,13-dioxoisoindolin-1-yl)butanoic acid 4c[2]

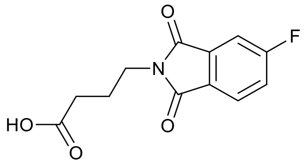

123g

4c was synthesised according to General Procedure **A** using 4-fluorophthalic anhydride (0.200 g, 1.20 mmol) and 4-aminobutanoic acid (0.124 g, 1.20 mmol) to afford the desired product as beige solid (0.302 g, Quantitive yield). **Mp**= 125-127 °C. **ν_{max} (KBr) cm^{-1}** 1699 (N-C=O), 1613 (C=O). **^{1}H NMR** (400 MHz, Chloroform-*d*) δ 7.84 (q, *J* = 8.2, 4.5 Hz, 1H, Ar-H), 7.50 (dd, *J* = 7.0, 2.3 Hz, 1H, Ar-H), 7.37 (td, *J* = 8.5, 2.3 Hz, 1H, Ar-H), 3.74 (t, *J* = 6.8 Hz, 2H, C(4)*H*), 2.40 (t, *J* = 7.4 Hz, 2H, C(2)*H*), 1.99 (m, 2H, C(3)*H*). **^{13}C NMR** (101 MHz, Chloroform-*d*) δ 178.71 (COOH), 167.42 (C=O), 167.08 (d, *J* = 3.1 Hz, C=O), 166.48 (d, *J* = 257.53 Hz, Ar**C**-F), 134.90 (d, *J*= 9.4 Hz, ArC), 127.86 (d, *J* = 2.6 Hz, ArC), 125.80 (d, *J* = 9.0 Hz, ArC), 121.11 (d, *J* = 23.8 Hz, ArC), 111.30 (d, *J* = 24.7

Hz, ArC), 37.43 (C(4)), 31.33 (C(2)), 23.61 (C(3)). ***m/z***= 250 [MH-] (ESI^-) and 274 [MNa+] (ESI^+). **HRMS** calculated for $C_{12}H_{10}FNNaO_4$ requires 274.0486, found 274.0484 [MNa+] (ESI^+).

4-(9-Carboxypropyl)-6,13-dioxoisoindoline-1-carboxylic acid 4d[6]

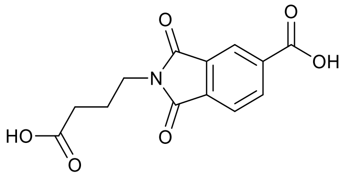

123h

4d was synthesised according to General Procedure **A** using trimellitic anhydride (0.500 g, 2.60 mmol) and 4-aminobutanoic acid (0.268 g, 2.60 mmol) at 215 °C with the crude material purified using flash column chromatography (5% Methanol in ethyl acetate with a drop of acetic acid) to afford the desired product as acolourless solid (0.537, 74%). ν_{max} **(KBr) cm^{-1}** 1774 (C=O), 1703 (N-C=O), 1486, 1397 (C=C). **^{1}H NMR** (400 MHz, Methanol-*d*4) δ 8.41 (dd, *J* = 7.7, 1.4 Hz, 1H, ArH), 8.36 (m, 1H, ArH), 7.95 – 7.90 (dd, *J*=7.2, 0.6 Hz, 1H, ArH), 3.75 (t, *J* = 6.8 Hz, 2H, C(4)*H*), 2.37 (t, *J* = 7.2 Hz, 2H, C(2)*H*), 1.99 (m, C(3)*H*). **^{13}C NMR** (101 MHz, Methanol-*d*4) δ 176.53 (C=O), 169.04 (C=O), 169.01 (C=O), 167.75 (C=O), 137.85 (Ar**C**-COOH), 136.63 (ArC), 136.55 (ArC), 133.68 (ArC), 124.85 (ArC), 124.19 (ArC), 38.53 (C(4)), 32.21 (C(2)), 24.77 (C3)). ***m/z***= 276 [MH-] (ESI^-). **HRMS** calculated for $C_{13}H_{10}NO_6$ requires 276.0503, found 276.0512 [MH-] (ESI^-).

4-(9-Methyl-6,13-dioxoisoindolin-5-yl)butanoic acid 4e[3]

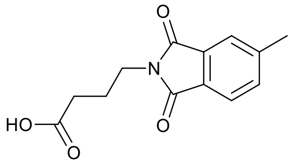

123i

4e was synthesised according to General Procedure **A** using 4-methylphthalic anhydride (0.786 g, 4.84 mmol) and 4-aminobutanoic acid (0.500 g, 4.84 mmol) to afford the desired product as a colourless solid (1.31 g, Quantitative yield). **MP**= 136-138 °C. ν_{max} **(KBr) cm^{-1}** 1767 (C=O), 1697 (N-C=O). **^{1}H NMR** (400 MHz, Chloroform-*d*) δ 7.70 (d, *J* = 7.7 Hz, 1H, Ar-H), 7.62 (dt, *J* = 1.5, 0.7 Hz, 1H, AR-H), 7.48 (ddq, *J* = 7.5, 1.4, 0.7 Hz, 1H, Ar-H), 3.73 (t, *J* = 6.8 Hz, 2H, C(4)*H*), 2.49 (d, *J* = 0.7 Hz, 3H, C(9a)Ar-CH_3), 2.40 (t, *J* = 7.5 Hz, 2H, C(2)*H*), 2.05 – 1.93 (q, *J*=7.0 Hz, 2H, C(3)*H*). **^{13}C NMR** (101 MHz, Chloroform-*d*) δ 178.37 (COOH), 168.69 (C=O), 168.58 (C=O), 145.41 (ArC), 134.64 (ArC), 132.51 (ArC), 129.52 (ArC), 123.98 (ArC), 123.34 (ArC), 37.13 (C(4)), 31.38 (C(2)), 23.80 (C(3)), 22.10 (**C**H_3). ***m/z***= 246 [MH-] (ESI^-), 270 [MNa+] (ESI^+). **HRMS** calculated for $C_{13}H_{13}NNaO_4$ requires 270.0731, found 270.0739 [MNa+] (ESI^+).

Methyl 1-(6,13-dioxoisoindolin-4-yl)butanoate 5a

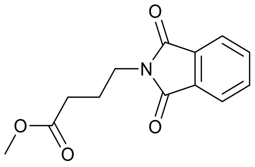

137a

5a was synthesised according to General Procedure **B** using **3a** (1.49 g, 6.02 mmol) to afford the desired product as a colourless solid (1.49 g, quantitative). **Mp**= 86-88 °C (Lit.[7] 89-90 °C). **ν_{max} (KBr) cm^{-1}** 1770, 1733 (N-C=O), 1707 (C=O). **^{1}H NMR** (400 MHz, Chloroform-*d*) δ 7.84 (dd, *J* = 5.4, 3.0 Hz, 2H, Ar-H), 7.72 (dd, *J* = 5.5, 3.0 Hz, 2H, Ar-H), 3.75 (t, *J* = 6.8 Hz, 2H, C(4)*H*), 3.65 (s, 3H, OMe), 2.38 (t, *J* = 7.5 Hz, 2H, C(2)*H*), 2.02 (m, 2H, C(3)*H*). **^{13}C NMR** (101 MHz, Chloroform-*d*) δ 173.48 (COOMe), 168.76 (CC=O), 134.41 (ArC), 132.46 (ArC), 123.68 (ArC), 52.13 (C(OMe)), 37.60 (C(4)), 31.74 (C2)), 24.31 (C3)). ***m/z***= 270 [MNa+] (ESI^{+}). **HRMS** calculated for $C_{13}H_{14}NO_4$ requires 248.0917, found 248.0917 [MH+] (ESI^{+}).

Methyl 1-(8-nitro-6,13-dioxoisoindolin-4-yl)butanoate 5b

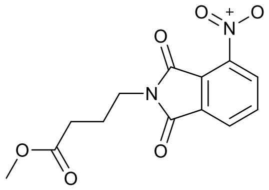

137b

5b was synthesised according to General Procedure B using **3b** (0.690 g, 2.48 mmol) with the crude material purified using flash column chromatography (40% ethyl acetate in pet-ether 40:60) to afford the desired product as a yellow coloured solid (0.584 g, 80%). **R_f**= 0.49 (40% ethyl acetate in pet-ether 40:60). **Mp**= 58-60 °C. **ν_{max} (KBr) cm^{-1}** 1778 (N-C=O), 1714 (C=O), 1540 (N-O, N=O). **^{1}H NMR** (400 MHz, Chloroform-d) δ 8.13 – 8.06 (m, 2H, Ar-H), 7.91 (dd, *J* = 8.2, 7.3 Hz, 1H, Ar-H), 3.77 (t, *J* = 6.9 Hz, 2H, C(4)*H*), 3.63 (s, 3H, OMe), 2.38 (t, *J* = 7.3 Hz, 2H, C(2)*H*), 2.02 (m, 2H, C(3)*H*). **^{13}C NMR** (101 MHz, Chloroform-*d*) δ 172.97 (COOMe), 165.90 (C=O), 163.01 (C=O), 145.21 (Ar**C**-NO_2), 135.49 (ArC), 134.20 (ArC), 128.62 (ArC), 127.10 (ArC), 123.85 (ArC), 51.82 (OMe), 38.09 (C(4)), 31.40 (C(2)), 23.68 (C(3)). ***m/z***= 315 [MNa+] (ESI^{+}). **HRMS** calculated for $C_{13}H_{12}NaN_2O_6$ requires 315.0587, found 315.0581 [MNa+] (ESI^{+}).

Methyl 1-(8-fluoro-6,13-dioxoisoindolin-4-yl)butanoate 5c

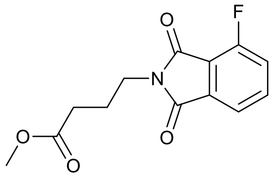

137f

5c was synthesised according to General Procedure B using **123f/3c** (0.370 g, 1.472 mmol) to afford the desired product as a brown coloured oil (0.393 g, quantitative yield). **ν_{max} (KBr) cm^{-1}** 1774 (N-C=O), 1708 (C=O), 1611 (N-C=O). **^{1}H NMR** (400 MHz, Chloroform-*d*) δ 7.72 (ddd, *J* = 8.1, 7.4, 4.3 Hz, 1H, Ar-H), 7.67 (d, *J* = 7.3 Hz, 1H, Ar-H), 7.38 (td, *J* = 8.5, 1.0 Hz, 1H, Ar-H), 3.75 (t, *J* = 6.8 Hz, 2H, C(4)*H*), 3.66 (s, 3H, OMe), 2.38 (t, *J* = 7.4 Hz, 2H, C(2)*H*), 2.02 (m, 2H, C(3)*H*). **^{13}C NMR** (101 MHz, Chloroform-*d*) δ 173.14 (COOMe), 156.35 (Ar**C**-F), 136.78 and 136.70 (d, *J*=19.9 Hz, (ArC), 122.66 and 122.46 (d, *J*=20 Hz, (ArC), 119.68 and 119.64 (d, *J*=4.2 Hz, ArC), 51.89 (OMe), 37.48 (C(4)), 31.40 (C(2)), 23.88 (C(3)). ***m/z***= 288 [MNa+] (ESI^{+}). **HRMS** calculate for $C_{13}H_{12}FNaNO_4$ requires 288.0642, found 288.0641 [MNa+] (ESI^{+}).

Methyl 1-(8-hydroxy-6,13-dioxoisoindolin-5-yl)butanoate 5d

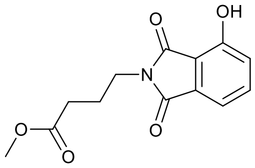

137d

5d was synthesised according to General Procedure B using **3d** (1.291 g, 5.17 mmol) to afford the desired product as a beige coloured solid (1.31 g, 96%). **Mp**= 121-123 °C. **ν_{max} (KBr) cm^{-1}** 31721 (N-C=O), 1686 (C=O), 1613, 1445 (C=C). **^{1}H NMR** (400 MHz, Chloroform-d) δ 7.65 – 7.54 (m, 2H, Ar-H), 7.38 (d, *J* = 7.2 Hz, 1H, Ar-H), 7.16 (d, *J* = 8.4 Hz, 1H, Ar-H), 3.71 (t, *J* = 6.9 Hz, 2H, (C(4)*H*), 3.66 (s, 3H, OMe), 2.38 (t, *J* = 7.4 Hz, 2H, (C(2)*H*), 2.02 (m, 2H, C(3)*H*). **^{13}C NMR** (101 MHz, Chloroform-*d*) δ 173.12 (COOMe), 170.36 (C=O), 167.98 (C=O), 154.70 (Ar**C**-OH), 136.47 (ArC), 132.12 (ArC), 122.80 (ArC), 116.02 (ArC), 114.59 (ArC), 51.83 (OMe), 37.07 (C(4)), 31.32 (C(2)), 23.92 (C(3)). ***m/z***= 262 [MH-] (ESI^{-}) and 286 [MNa+] (ESI^{+}). **HRMS** calculated for $C_{13}H_{14}NO_5$ requires 264.0867, found 264.0866 [MH+] (ESI^{+}).

Methyl 1-(8-(benzyloxy)-6,13-dioxoisoindolin-5-yl)butanoate 5e

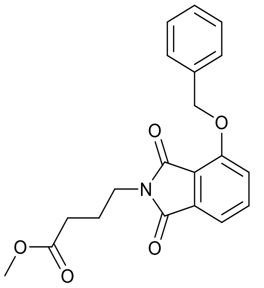

137j

Compound **5d** (0.200 g, 0.75 mmol) was dissolved in acetone (10 mL) followed by the addition of K_2CO_3 (0.126 g, 0.910 mmol) in one portion with the reaction mixture left to stir at room temperature for 5 minutes. Benzyl bromide (0.099 mL, 0.834 mmol) was then added dropwise over 5 minutes with the resulting mixture left stirring at room temperature for 16 hours. The volatiles were removed by reduced pressure then redissolved in DCM (20 mL). The organic layer was washed using water (2 x 20 mL) then back extracted using DCM (40 mL). The combined organic layers were washed with brine (40 mL) and dried over $MgSO_4$ and concentrated by reduced pressure. The crude was purified using flash column chromatography (using a gradient from 0-30% ethyl acetate in pet-ether 40:60) to afford the desired product as a colourless solid (0.196 g, 73%). **R_f**= 0.27 (30% ethyl acetate in pet-ether 40:60). **Mp**= 82-84 °C. **ν_{max} (KBr) cm^{-1}** 1730 (N-C=O), 1703 (C=O). **^{1}H NMR** (400 MHz, Chloroform-*d*) δ 7.58 (t, 1H, Ar-H), 7.51 – 7.45 (m, 2H, Ar-H), 7.44 – 7.35 (m, 3H, Ar-H), 7.35 – 7.28 (m, 1H, Ar-H), 7.19 (d, *J* = 8.4 Hz, 1H, Ar-H), 5.33 (s, 2H, C(8a) Ar-**CH_2**-O), 3.72 (t, *J* = 6.8 Hz, 2H, C(4)*H*), 3.64 (s, 3H, OMe), 2.37 (t, *J* = 7.5 Hz, 2H, C(2)*H*), 2.01 (m, 2H, C(3)*H*). **^{13}C NMR** (101 MHz, Chloroform-*d*) δ 173.22 (COOMe), 168.13 (C=O), 166.89 (C=O), 155.74 (ArC), 136.04 (ArC), 135.90 (Ar**C)**, 134.35 (ArC), 128.85 (ArC), 128.24 (ArC), 126.86 (ArC), 119.50 (ArC), 118.01 (ArC), 115.87 (ArC), 70.94 (**CH_2**-O), 51.78 (OMe), 37.12 (C(4)), 31.45 (C(2)), 24.00 (C(3)). ***m/z***= 376 [MNa+] (ESI$^+$). **HRMS** calculated for $C_{20}H_{19}NaNO_5$ requires 376.1155, found 376.1152 [MNa+] (ESI$^+$).

Methyl 1-(8-methoxy-6,13-dioxoisoindolin-5-yl)butanoate 5f

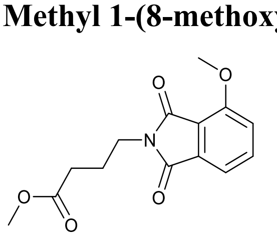

123k

Compound **5d** (0.200 g, 0.75 mmol) was dissolved in acetone (10 mL) followed by the addition of K_2CO_3 (0.126 g, 0.91 mmol) in one portion with the reaction mixture left to stir at room temperature for 5 minutes. Methyl iodide (0.052 mL, 0.83 mmol) was then added dropwise over 5 minutes with resulting mixture left stirring at room temperature for 16 hours. The volatiles were removed by

reduced pressure then redissolved in DCM (20 mL). The organic layer was washed using water (2 x 20 mL) then back extracted using DCM (40 mL). The combined organic layers were washed with brine (40 mL) then dried over $MgSO_4$ and concentrated by reduced pressure to afford the desired product as a yellow coloured solid (0.240 g, quantitative yield). **Mp** 76-80 °C. **ν_{max} (KBr) cm^{-1}** 1766, 1733 (N-C=O), 1704 (C=O). **^{1}H NMR** (400 MHz, Chloroform-*d*) δ 7.67 (dd, *J* = 8.4, 7.3 Hz, 1H, Ar-H), 7.44 (dd, *J* = 7.3, 0.8 Hz, 1H, Ar-H), 7.21 (dd, *J* = 8.5, 0.7 Hz, 1H, Ar-H), 4.03 (s, 3H, C(8a)*H*), 3.78 – 3.62 (m, 5H, C(4)*H*, C(OMe)), 2.38 (t, *J* = 7.4 Hz, 2H, C(2)*H*), 2.03 (m, C(3)*H*). **^{13}C NMR** (101 MHz, Chloroform-*d*) δ 173.55 (COOMe), 168.47 (C=O), 167.45 (C=O), 157.02 (Ar**C**-O), 136.57 (ArC), 134.59 (ArC), 117.86 (ArC), 117.67 (ArC), 115.86 (ArC), 56.75 (ArC-O**C**H_3), 52.10 (OMe), 37.40 (C(4)), 31.73 (C(2)), 24.29 (C(3)). ***m/z***= 300 [MNa+] (ESI$^+$). **HRMS** calculated for $C_{14}H_{15}NO_5$ requires 278.1023, found 278.1025 [MH+] (ESI$^+$).

Methyl 1-(9-nitro-1,3-dioxoisoindolin-4-yl)butanoate 6a

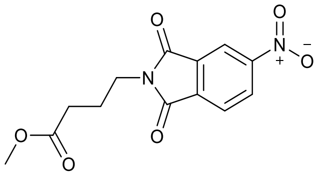

137c

6a was synthesised according to General Procedure B using **4a** (0.288 g, 1.03 mmol) to afford the desired product as a beige coloured solid (0.302 g, quantitative yield). **Mp**= 100-102 °C. **ν_{max} (KBr) cm^{-1}** 1725 (N-C=O), 1700 (C=O), 1540 (N-O, N=O). **^{1}H NMR** (400 MHz, Chloroform-*d*) δ 8.68 – 8.63 (m, 1H, Ar-H), 8.60 (d, *J* = 8.1 Hz, 1H, Ar-H), 8.04 (d, *J* = 7.8 Hz, 1H, Ar-H), 3.80 (t, *J* = 6.7 Hz, 2H, C(4)*H*), 3.64 (s, 3H, OMe), 2.39 (t, *J* = 7.1 Hz, 2H, C(2)*H*), 2.03 (m, 2H, C(3)*H*). **^{13}C NMR** (101 MHz, Chloroform-*d*) δ 172.99 (COOMe), 166.28 (C=O), 166.00 (C=O), 151.81 (Ar**C**-NO_2), 136.53 (ArC), 133.50 (ArC), 129.40 (ArC), 124.60 (ArC), 118.78 (ArC), 51.91 (OMe), 38.02 (C(4)), 31.30 (C(2)), 23.70 (C(3)). ***m/z***= 315 [MNa+] (ESI$^+$). **HRMS** calculated for $C_{13}H_{12}NaN_2O_6$ requires 315.0587, found 315.0589 [MNa+] (ESI$^+$).

Methyl 1-(9-bromo-6,13-dioxoisoindolin-1-yl)butanoate 6b

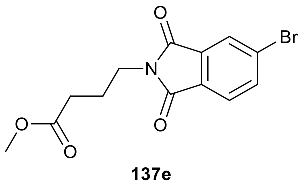

137e

Methyl γ-butyric acid (1.236 g, 9.23 mmol) was added to 4-bromophthalic anhydride (2.436 g, 9.32 mmol) and dissolved in toluene (30 mL) followed by the addition of DIPEA (4.84 mL, 27.87 mmol) and left to stir for 16 hours at reflux. The reaction was allowed to cool to room temperature with the

solvent being removed by reduced pressure to afford a yellow coloured oil. The impure oil was then triturated using MeOH and the product filtered under vacuum and washed using ice cold MeOH (20 mL) to afford the desired product as a while coloured solid (3.030 g, quantitative yield). **Mp**= 78-80 °C. **ν_{max} (KBr) cm^{-1}** 1774 (C=O), 1728 (C=O), 1696 (N-C=O). **^{1}H NMR** (400 MHz, Chloroform-d) δ 7.94 (dd, *J* = 1.7, 0.6 Hz, 1H, Ar-H), 7.83 (dd, *J* = 7.9, 1.7 Hz, 1H, Ar-H), 7.68 (dd, *J* = 7.9, 0.6 Hz, 1H, Ar-H), 3.72 (t, *J* = 6.9 Hz, 2H, C(4)*H*), 3.63 (s, 3H, OMe), 2.35 (t, *J* = 7.4 Hz, 2H, (C(2)*H*), 1.99 (m, 2H, C(3)*H*). **^{13}C NMR** (101 MHz, Chloroform-*d*) δ 173.06 (COOMe), 167.57 (C=O), 167.04 (C=O), 137.07 (ArC), 133.73 (ArC), 130.62 (ArC), 128.98 (Ar**C**-Br), 126.72 (ArC), 124.72 (ArC), 51.83 (OMe), 37.50 (C(4)), 31.32 (C(2)), 23.83 (C(3)). ***m/z***= 325.9 and 327.9 [MNa+] (ESI$^+$). **HRMS** calculated for $C_{13}H_{12}BrNNaO_4$ requires 326.0022 and 328.0002, found 326.0024 and 328.0004 [MNa+] (ESI$^+$).

Methyl 1-(9-fluoro-6,13-dioxoisoindolin-4-yl)butanoate 6c

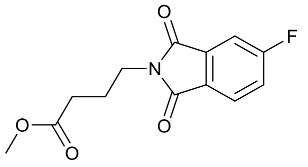

137g

6c was synthesised according to General Procedure B using **4c** (0.302 g, 1.20 mmol) to afford the desired product as a beige coloured solid (0.320 g, quantitative yield). **Mp**= 96-98 °C. **ν_{max} (KBr) cm^{-1}** 1679 (C=O). **^{1}H NMR** (400 MHz, Chloroform-d) δ 7.84 (dd, *J* = 8.2, 4.5 Hz, 1H, Ar-H), 7.51 (dd, *J* = 7.0, 2.3 Hz, 1H, Ar-H), 7.37 (td, 1H, Ar-H), 3.73 (t, *J* = 6.8 Hz, 2H, C(4)*H*), 3.64 (s, 3H, OMe), 2.37 (t, *J* = 7.4 Hz, 2H, C(2)*H*), 2.00 (m, 2H, C(3)*H*). **^{13}C NMR** (101 MHz, Chloroform-*d*) δ 173.11 (COOMe), 167.37 (C=O), 167.05 (d, *J* = 3.1 Hz, C=O), 166.46 (d, *J* = 256.5 Hz, Ar**C**-F), 134.94 (d, *J* = 9.5 Hz, ArC), 127.89 (d, *J* = 3.1 Hz, ArC), 125.73 (d, *J* = 9.5 Hz, ArC), 121.08 (d, *J* = 23.7 Hz, ArC), 111.25 (d, *J* = 24.8 Hz, ArC), 51.85 (OMe), 37.54 (C4)), 31.36 (C2)), 23.91 (C3)). ***m/z***= m/z=288 [MNa+] (ESI$^+$). **HRMS** calculated for $C_{13}H_{13}FNO_4$ requires 266.0823, found 266.0824 [MH+] (ESI$^+$).

Methyl 1-(9-methoxy-9-oxobutyl)-6,13-dioxoisoindoline-9-carboxylate 6d

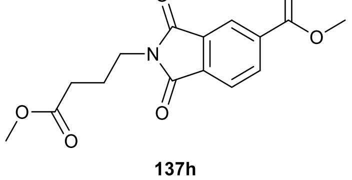

137h

6d was synthesised according to General Procedure B using **4d** (0.288 g, 1.03 mmol) with the crude material purified using flash column chromatography (20% ethyl acetate in pet-ether 40:60) to afford

the desired product as a colourless solid (0.198 g, 62%). **Mp**= 108-109°C. **ν_{max} (KBr) cm^{-1}** 2980, 1742 (N-C=O), 1702 (C=O). **^{1}H NMR** (400 MHz, Chloroform-*d*) δ 8.43 (d, *J* = 1.2 Hz, 1H, Ar-H), 8.37 (dd, *J* = 7.8, 1.4 Hz, 1H, Ar-H), 7.88 (d, *J* = 7.8 Hz, 1H, Ar-H), 3.95 (s, 3H, OMe), 3.74 (t, *J* = 6.9 Hz, 2H, C(4)*H*), 3.61 (s, 3H, OMe), 2.35 (t, *J* = 7.3 Hz, 2H, C(2)*H*), 2.00 (m, 2H, C(3)*H*). **^{13}C NMR** (101 MHz, Chloroform-*d*) δ 173.03 (COOMe), 167.43 and 167.39 (C=O), 165.23 (COOMe), 135.63 (ArC), 135.47 (ArC), 135.40 (Ar**C**-COOMe), 132.30 (ArC), 124.38 (ArC), 123.37 (ArC), 52.94 (OMe), 51.79 (OMe), 37.54 (C(4)), 31.32 (C2)), 23.82 C(3)). ***m/z***= 328 [MNa+] (ESI^{+}). **HRMS** calculated for $C_{15}H_{15}NaNO_6$ requires 328.0792, found 328.0790 [MNa+] (ESI^{+}).

Methyl 1-(9-methyl-6,13-dioxoisoindolin-5-yl)butanoate 6e

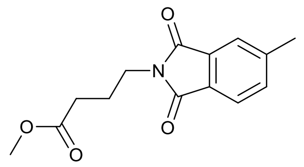

137i

6e was synthesised according to General Procedure B using **4e** (1.311 g, 5.30 mmol) to afford the desired product as a colourless solid (1.148 g, quantitative yield). **Mp**= 77-79 °C. **ν_{max} (KBr) cm^{-1}** 1768 (C=O), 1731 (N-C=O), 1703 (C=O). **^{1}H NMR** (400 MHz, Chloroform-*d*) δ 7.73 (d, *J* = 7.6 Hz, 1H, Ar-H), 7.68 – 7.63 (m, 1H, Ar-H), 7.51 (d, *J* = 7.2 Hz, 1H, Ar-H), 3.74 (t, *J* = 6.8 Hz, 2H, C(4)*H*), 3.66 (s, 3H, OMe), 2.52 (s, 3H, Ar-**CH_3**), 2.39 (t, *J* = 7.5 Hz, 2H, C(2)*H*), 2.03 (m, 2H, (C(3)*H*). **^{13}C NMR** (101 MHz, Chloroform-*d*) δ 173.19 (COOMe), 168.65 (C=O), 168.53 (C=O) 145.36 (Ar**C**-CH_3), 134.61 (ArC), 132.55 (ArC), 129.56 (ArC), 123.93 (ArC), 123.28 (ArC), 51.80 (OMe), 37.22 C(4)), 31.45 (C(2)), 24.04 (C(3)), 22.13 (C(9a)ArC-**C**H_3). ***m/z***= 284 [MNa+] (ESI^{+}).

Benzyl 1-(6,13-dioxoisoindolin-5-yl)butanoate 7a

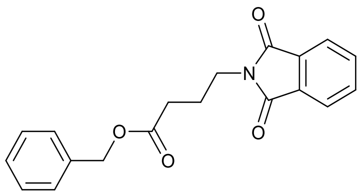

173

Compound **3a** was dissolved in thionyl chloride (1 mL) and left stirring at room temperature for 1 hour. The solvent was removed then redissolved in anhydrous DCM (10 mL). The reaction mixture was cooled to 0 °C using an ice bath with benzyl alcohol (0.390 mL, 3.77 mmol) added dropwise over 5 minutes and left stirring at room temperature for 16 hours. The reaction mixture was concentrated by reduced pressure and the crude material purified using flash column chromatography (DCM) to afford the desired product as a colourless oil (0.368 g, 60%). **R_f**= 0.59 (DCM) and 0.27 (20% ethyl acetate in pet-ether 40:60). **ν_{max} (KBr) cm^{-1}** 1770 (N-C=O), 1707 (C=O). **^{1}H NMR** (400

MHz, Chloroform-*d*) δ 7.88 – 7.78 (m, 2H, Ar-H), 7.75 – 7.66 (m, 2H, Ar-H), 7.39 – 7.27 (m, 5H, Bn), 5.09 (s, 2H, O-C**H₂**-Bn), 3.76 (t, *J* = 6.9 Hz, 2H, C(4)*H*), 2.43 (t, *J* = 7.5 Hz, 2H, C(2)*H*), 2.04 (m, 2H, C(3)*H*). **^{13}C NMR** (101 MHz, Chloroform-*d*) δ 172.52 (COOBn), 168.43 (C=O), 135.97 (ArC), 134.08 (ArC), 132.18 (ArC), 128.67 (ArC), 128.65 (ArC), 128.35 (ArC), 123.37 (ArC), 66.51 (O-**C**H$_2$-Bn), 37.31 (C(4)), 31.72 (C2)), 24.02 (C(3)). ***m/z***= 346 [MNa+] (ESI$^+$). **HRMS** calculated for $C_{19}H_{17}NaNO_4$ calculated for $C_{19}H_{17}NaNO_4$ requires 346.1049, found 346.1053 [MNa+] (ESI$^+$).

1-(6,13-Dioxoisoindolin-4-yl)-*N*-phenylbutanamide 7b

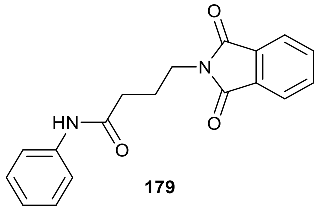

179

Compound **3a** (0.150 g, 0.64 mmol) was dissolved in DCM (5 mL) followed by the addition of EDC (0.228 g, 0.77 mmol) and stirred at room temperature for 1 hour. Aniline (0.16 mL, 0.77 mmol) was then added with the reaction left stirring at room temperature for an additional 16 hours. The reaction was then diluted using DCM (5 mL) and washed using water (10 mL) followed by an acid wash using 0.5 N HCl (10 mL). The organic layer was dried over $MgSO_4$ and concentrated by reduced pressure with the crude material being purified using flash column chromatography (40% ethyl acetate in pet-ether 40:60) to afford the desired product as a colourless solid (0.085 g, 43%). **R$_f$**= 0.28. **Mp**= 166-169 °C (Lit.[8] 155-175 °C). 3327 (N-H), 1770 (C=O), 1706 (N-C=O). **^{1}H NMR** (400 MHz, Chloroform-*d*) δ 7.92 – 7.80 (m, 2H, ArH), 7.73 (dt, *J* = 5.1, 3.5 Hz, 2H, ArH), 7.58 (d, *J* = 8.1 Hz, 2H, ArH), 7.33 (dd, *J* = 7.3, 1.8 Hz, 2H, ArH), 7.09 (t, *J* = 7.4 Hz, 1H, ArH), 3.88 – 3.75 (m, 2H, C(4)*H*), 2.44 – 2.31 (m, 2H, C(2)*H*), 2.12 (td, *J* = 11.6, 10.4, 5.5 Hz, 2H, C(3)*H*). ***m/z***= 618 [2MH+] (ESI$^+$). **HRMS** calculated for $C_{18}H_{17}N_2O_3$ requires 309.1234, found 309.1238 [MH+] (ESI$^+$).

1-(6,13-Dioxoisoindolin-4-yl)-*N*-(quinolin-8-yl)butanamide[9] 7c

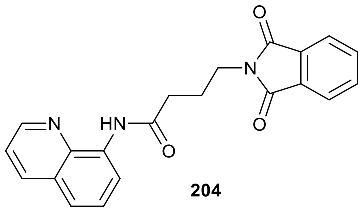

204

Compound **3a** (0.508 g, 2.17 mmol) was dissolved in an excess of $SOCl_2$ (4 mL) and left stirring at room temperature under N_2 for 16 hours. The solvent was removed using reduced pressure and azeotroped using DCM (3 x 10 mL). The flask containing the acid chloride was then purged using N_2 and dissolved in anhydrous DCM (5 mL). The acid chloride mixture was then slowly added to 8-aminoquinoline (0.298 g, 2.06 mmol) and TEA (0.364 mL, 2.71 mmol) dissolved in DCM (5 mL)

and stirred at room temperature while under an inert atmosphere of N_2 for 16 hours. The crude material was then washed using water (2 x 10 mL) and back extracted using DCM (20 mL) with the combined organic layers combined and concentrated by reduced pressure. The crude oil was purified using flash column chromatography (40% ethyl acetate in pet-ether 40:60) to afford the desired product as a light brown coloured solid (0.323 g, 57%). **R_f**= 0.40. **ν_{max} (KBr) cm^{-1}** 3345 (N-H), 1769 (N-C=O), 1699 (C=O), 1674 (N-C=O). **^{1}H NMR** (400 MHz, Chloroform-d) δ 9.77 (s, 1H, N-*H*), 8.78 (dd, *J* = 4.3, 1.7 Hz, 1H, ArC), 8.69 – 8.63 (m, 1H, ArC), 8.12 (dd, *J* = 8.3, 1.7 Hz, 1H, ArC), 7.77 (dd, *J* = 5.4, 3.0 Hz, 2H, ArC), 7.67 – 7.61 (m, 2H, ArC), 7.47 – 7.44 (m, 2H, ArC), 7.44 – 7.40 (m, 1H, ArC), 3.85 (t, *J* = 6.7 Hz, 2H, C(4)*H*), 2.63 (dd, *J* = 8.0, 7.0 Hz, 2H, C(2)*H*), 2.26 – 2.17 (m, 2H, C(3)*H*). **^{13}C NMR** (101 MHz, Chloroform-*d*) δ 170.46 (C=O), 168.52 (N-C=O), 148.20 (ArC), 138.38 (ArC), 136.39 (ArC), 134.49 (ArC), 133.96 (ArC), 132.16 (ArC), 127.97 (ArC), 127.44 (ArC), 123.28 (ArC), 121.66(ArC), 121.49(ArC), 116.53 (ArC), 37.58 (C(4)), 35.35 (C(2)), 24.60 (C3)). ***m/z***= 360 [MH-] (ESI^-). **HRMS** calculated for $C_{21}H_{16}N_3O_3$ requires 360.1343, found 360.1334 [MH-] (ESI^-).

Methyl (2*R*,13*R*)-13-((tert-butyldimethylsilyl)oxy)-6-oxo-3,4,6,13-1H-benzo[a]pyrrolizine-2-carboxylate 8a

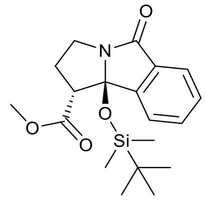

144a

8a was synthesised according to General Procedure C using **8a** (2.86 g, 11.57 mmol) with the crude material purified using flash column chromatography (30% ethyl acetate in pet-ether 40:60) to afford the desired product as a colourless solid (3.91 g, 97%). **R_f**= 0.31 (20% ethyl acetate in pet-ether 40:60). **Mp**= 86-88° C. **ν_{max} (KBr) cm^{-1}** 1737 (C=O), 1718 (N-C=O). **^{1}H NMR** (600 MHz, Chloroform-*d*) δ 7.66 (dd, *J* = 7.5, 1.0 Hz, 1H, Ar-H), 7.53 – 7.47 (m, 2H, Ar-H), 7.44 (ddd, *J* = 7.5, 6.9, 1.5 Hz, 1H, Ar-H), 3.95 (dt, *J* = 10.7, 8.5 Hz, 1H, C(4)H_A), 3.42 (ddd, *J* = 11.0, 9.5, 2.2 Hz, 1H, C(4)H_B), 3.30 (d, *J* = 6.8 Hz, 1H, C(2)*H*), 3.11 (s, 3H, OMe), 2.73 – 2.66 (m, 1H, C(3)H_A), 2.49 (dddd, *J* = 13.1, 8.5, 2.2, 1.0 Hz, 1H, C(3)H_B), 0.82 (s, 9H, $C(CH_3)_3$), -0.08 (s, 3H, Si-CH_3), -0.51 (s, 3H, Si-CH_3). **^{13}C NMR** (101 MHz, Chloroform-*d*) δ 171.60 (COOMe), 170.35 (C=O), 144.36 (ArC), 132.75 (ArC), 132.20 (ArC), 130.03 (ArC), 123.50 (ArC), 123.26 (ArC), 99.08 (C(13)), 53.05 C(2)), 51.41 (OMe), 42.11 (C(4)), 31.03 (C(3)), 25.48 (C(**C**H_3)$_3$), 17.80 (Si-**C**$(CH_3)_3$), -4.30 (Si-CH_3), -4.50 (Si-CH_3). ***m/z***= 384 [MNa+] (ESI^+). **HRMS** calculated for $C_{19}H_{27}NaNO_4Si$ requires 362.1782, found 362.1781 [MNa+] (ESI^+).

Methyl (2*R*,13*R*)-13-((tert-butyldimethylsilyl)oxy)-8-nitro-6-oxo-3,4,6,13-tetrahydro-1H-benzo[a]pyrrolizine-2-carboxylate 8b

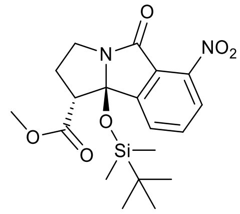

144b

8b was synthesised according to General Procedure C using **5b** (0.490 g, 1.67 mmol) with the crude material purified using flash column chromatography (30% ethyl acetate in pet-ether 40:60) to afford the desired product as a yellow coloured solid (0.433 g, 82%). $\mathbf{R_f}$= 0.51 (30% ethyl acetate in pet-ether 40:60). $\mathbf{\nu_{max}}$ **(KBr) cm^{-1}** 1726 (C=O), 1535 (N-O_2). **^{1}H NMR** (400 MHz, Chloroform-*d*) δ 7.85 (dd, *J* = 7.8, 1.1 Hz, 1H, Ar-H), 7.75 (dd, *J* = 7.7, 1.1 Hz, 1H, Ar-H), 7.69 (t, *J* = 7.6 Hz, 1H, Ar-H), 3.99 (dt, *J* = 11.5, 9.0 Hz, 1H, C(4)H_A), 3.46 (ddd, *J* = 11.4, 9.4, 2.1 Hz, 1H, C(4)H_B), 3.38 – 3.34 (d, *J*=6.7 Hz, 1H, C(2)*H*), 3.24 (s, 3H, OMe), 2.76 (dtd, *J* = 13.3, 9.4, 7.1 Hz, 1H, C(3)H_A), 2.53 (dddd, *J* = 13.4, 8.5, 2.2, 1.0 Hz, 1H, C(3)H_b), 0.85 (s, 9H, C(CH_3)$_3$), 0.03 (s, 3H, Si-CH_3), -0.44 (s, 3H, Si-CH_3). **^{13}C NMR** (126 MHz, Chloroform-*d*) δ 171.23 (COOMe), 164.88 (C=O), 147.29 (ArC), 145.56 (Ar**C**-NO_2), 133.15 (ArC), 127.44 (ArC), 125.37 (ArC), 124.77 (ArC), 97.83 (C(13)), 53.11 (C(2)), 51.89 (OMe), 42.94 (C(4)), 31.13 (C(3)), 25.64 (C(**C**H_3)$_3$, 17.89 (Si-**C**(CH_3)$_3$), -4.12 (Si-CH_3), -4.21 (Si-CH_3). ***m/z***= 407 [MH+] (ESI$^+$). **HRMS** calculated for $C_{19}H_{27}N_2O_6Si$ requires 407.1632, found 407.1634 [MH+] (ESI$^+$).

Methyl (2*R*,13*R*)-13-((tert-butyldimethylsilyl)oxy)-8-fluoro-6-oxo-3,4,6,13-tetrahydro-1H-benzo[a]pyrrolizine-2-carboxylate 8c and **Methyl (2*R*,13*R*)-13-((tert-butyldimethylsilyl)oxy)-11-fluoro-6-oxo-3,4,6,13-tetrahydro-1H-benzo[a]pyrrolizine-2-carboxylate 9**

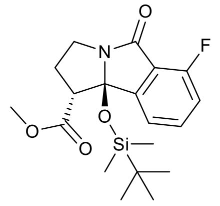

144e

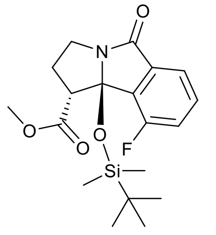

171

8c and **9** were synthesised according to General Procedure C using **5c** (0.389 g, 1.46 mmol) with the crude material purified using flash column chromatography (25% ethyl acetate in pet-ether 40:60) to afford the desired product as a mixture of isomers as a colourless solid (0.349 g, 61%). *d.r*= 1:0.3 **8a**:**9**. The structure of **9** was confirmed *via* x-ray analysis. The oil that remained was enriched in isomer **8c** (ratio now at 9:1) which was also able to be crystallised and analysed by crystallography.

R_f= 0.40. **ν_{max} (KBr) cm^{-1}** 1724 (N-C=O), 1625 (C=O). ***m/z***= 380 [MH+] (ESI$^+$). **HRMS** calculated for $C_{19}H_{27}FNO_4Si$ requires 380.1687, found at 380.1685 [MH+] (ESI$^+$).

8c: **^{1}H NMR** (500 MHz, Chloroform-*d*) δ 7.55 – 7.44 (m, 1H, Ar-H), 7.32 – 7.28 (d, *J*= 7.3 Hz, 1H, Ar-H), 7.14 – 7.08 (t, *J*=17.6, 8.2 Hz, 1H, ArH), 4.03 – 3.87 (m, 1H, C(4)H_A), 3.46 – 3.39 (m, 1H, C(4)H_B), 3.31 (dd, *J* = 7.0, 1.0 Hz, 1H, (C(2)*H*), 3.22 (s, 3H, OMe), 2.72 (dddd, *J* = 16.5, 13.3, 9.4, 7.1 Hz, 1H, C(3)H_A), 2.58 – 2.47 (m, 1H, C(3)H_B), 0.84 (s, 9H, Si-C(CH_3)$_3$), -0.03 (s, 3H, Si-CH_3), -0.43 (s, 3H, Si-CH_3). **^{13}C NMR** (126 MHz, Chloroform-*d*) δ 171.55 (COOMe), 167.13 (d, *J*=1.8 Hz, C=O), 157.84 (d, *J*= 262.4 Hz, Ar**C**-F), 147.18 (d, *J*= 2.2 Hz, ArC), 134.52 (d, *J*= 7.5 Hz, ArC), 119.70 (d, *J*= 4.0 Hz, ArC), 117.66 (d, *J*= 19.8 Hz, ArC), 98.67 (d, *J*= 1.8 Hz, C(13) , 53.24 (C(2)), 51.72 (OMe), 42.47 (C(4)), 31.10 (C(3)), 25.59 (Si-C(**C**H$_3$)$_3$), 17.92 (Si-**C**(CH_3)$_3$), -4.13 (Si-CH_3), -4.36 (Si-CH_3).

9: **^{1}H NMR** (500 MHz, Chloroform-*d*) δ 7.55 – 7.44 (m, 2H, Ar-H), 7.20 (ddd, *J* = 8.9, 7.9, 1.1 Hz, 1H), 4.03 – 3.87 (m, 1H, C(4)H_A), 3.46 – 3.39 (m, 1H, (C(4)H_B), 3.31 (dd, *J* = 7.0, 1.0 Hz, 1H, C(2)*H*), 3.21 (s, 3H), 2.72 (ddtd, *J* = 16.5, 13.3, 9.4, 7.1 Hz, 1H, C(3)H_A), 2.58 – 2.47 (m, 1H, C(3H_B), 0.84 (s, 9H, Si-C(CH_3)$_3$), -0.04 (s,3H, Si-CH_3), -0.42 (s, 3H, Si-CH_3). **^{13}C NMR** (126 MHz, Chloroform-*d*) δ 171.58 (COOMe), 168.76 (d, *J*= 2.3 Hz, C=O), 157.75 (d, *J*= 262.4 Hz, Ar**C**-F), 135.96 (d, *J*= 3.6 Hz, Ar), 132.69 (d, *J*= 6.6 Hz, ArC), 130.17 (d, *J*= 16.5 Hz, ArC), 119.86 (d, *J*= 4.7 Hz, ArC), 119.45 (d, *J*= 3.8 Hz, ArC), 97.70 (C(13)), 52.60 (C(2)), 51.72 (OMe), 41.88 (C(4)), 30.95 (C(3)), 25.49 (Si-C(**C**H$_3$)$_3$), 17.96 (Si-**C**(CH_3)$_3$), -4.17 (Si-CH_3), -4.81 (Si-CH_3).

Methyl (2*R*,13*R*)-8-(benzyloxy)-13-((tert-butyldimethylsilyl)oxy)-6-oxo-2,3,5,13-tetrahydro-1H-benzo[a]pyrrolizine-2-carboxylate 8d

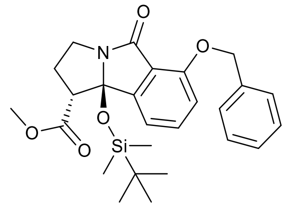

144j

8d was synthesised according to General Procedure C from **5e** (0.678 g, 1.92 mmol) with the crude material purified using flash column chromatography (30% ethyl acetate in pet-ether 40:60) to afford the desired product as a pale yellow coloured oil (0.632 g, 70%). R_f= 0.28. **ν_{max} (KBr) cm^{-1}** 1735 (C=O), 1713 (N-C=O). **^{1}H NMR** (400 MHz, Chloroform-*d*) δ 7.56 – 7.48 (m, 2H, ArH), 7.47 – 7.39 (m, 2H, ArH), 7.39 – 7.33 (m, 1H, ArH), 7.33 – 7.26 (m, 1H, ArH), 7.11 (dd, *J* = 7.5, 0.7 Hz, 1H, ArH), 6.95 (dd, *J* = 8.3, 0.7 Hz, 1H, ArH), 5.32 (s, 2H, OC**H$_2$**Ar), 4.03 (dt, *J* = 11.1, 8.9 Hz, 1H, C(4)H_A), 3.48 – 3.40 (m, 1H, C(4)H_B), 3.32 (dd, *J* = 7.1, 1.1 Hz, 1H, C(2)*H*), 3.22 (s, 3H, OMe), 2.81 – 2.64 (m, 1H, C(3)H_A), 2.52 (dddd, *J* = 13.1, 8.5, 2.3, 1.2 Hz, 1H, C(3)H_B), 0.87 (s, 9H, C(CH_3)$_3$),

0.00 (s, 3H, Si-CH_3), -0.43 (s, 3H, Si-CH_3). **^{13}C NMR** (101 MHz, Chloroform-*d*) δ 171.75 (COOMe), 169.14 (C=O), 155.57 (Ar**C**-OBn), 147.19 (ArC), 136.72 (Ar**C**CH_2O), 133.94 (ArC), 128.61 (ArC),127.82 (ArC), 126.95 (ArC), 120.63 (ArC), 116.40 (ArC), 115.43 (ArC), 98.37 (C(13)), 70.90 (O**C**H_2Ar), 53.51 (C(2)), 51.56 (OMe), 42.52 (C(4)), 30.96 (C(3)), 25.63 (C(**C**H_3)$_3$), 17.92 (**C**(CH_3)$_3$), -4.14 (Si-CH_3), -4.40 (Si-CH_3). ***m/z***= 347 [MH+] (ESI$^+$). **HRMS** calculated for $C_{26}H_{34}NO_5$ requires 368.2201, found 368.1299 [MH+] (ESI$^+$).

Methyl (2*R*,13*R*)-13-((tert-butyldimethylsilyl)oxy)-8-methoxy-6-oxo-3,4,6,13-tetrahydro-1H-benzo[a]pyrrolizine-2-carboxylate 8e

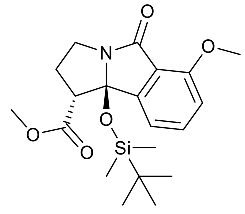

144k

8e was synthesised according to General Procedure C using **5f** (0.080 g, 0.28 mmol) with the crude material purified using flash column chromatography (30% ethyl acetate in pet-ether 40:60) to afford the desired product as a pale yellow coloured oil (0.98 g, 87%). **R_f**= 0.47. **ν_{max} (KBr) cm^{-1}** 1733 (C=O), 1715 (N-C=O). **^{1}H NMR** (400 MHz, Chloroform-*d*) δ 7.42 (dd, *J* = 8.2, 7.5 Hz, 1H, ArH), 7.28 (dd, *J* = 7.5, 0.8 Hz, 1H, ArH), 6.99 (dd, *J* = 8.2, 0.7 Hz, 1H, ArH), 3.94 – 3.84 (m, 4H, C(4)H_A and ArC-OC**H$_3$**), 3.46 – 3.36 (m, 2H, C(4)H_B and C(2)*H*), 3.17 (s, 3H, OMe), 2.74 (ddd, *J* = 13.1, 9.4, 7.0 Hz, 1H, C(3)H_A), 2.50 (dddd, *J* = 13.1, 8.6, 2.0, 0.8 Hz, 1H, C(3)H_B), 0.84 (s, 9H, C(CH_3)$_3$), -0.09 (s, 3H, Si-CH_3), -0.46 (s, 3H, Si-CH_3). **^{13}C NMR** (101 MHz, Chloroform-*d*) δ 171.73 (COOMe), 169.74 (C=O), 155.29 (Ar**C**OCH_3), 134.85 (ArC), 131.98 (ArC), 130.99 (ArC), 115.10 (ArC), 114.33 (ArC), 98.23 (C(13)), 55.36 (O**C**H_3), 52.18 (C(2)), 51.37 (OMe), 41.40 (C(4)), 31.04 (C(3)), 25.42 (C(**C**H_3)$_3$), 17.90 (**C**(CH_3)$_3$), -4.29 (Si-CH_3), -4.93 (Si-CH_3). ***m/z***= 414 [MNa+] (ESI$^+$). **HRMS** calculated for $C_{20}H_{30}NO_5Si$ requires 392.1888, found 392.1886 [MH+] (ESI$^+$).

Methyl 6-oxo-3,4-dihydro-1H-benzo[a]pyrrolizine-2-carboxylate 10a

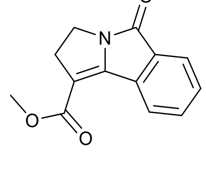

146a

Method 1) 10a was synthesised according to General Procedure C from **5a** (0.400 g, 1.61 mmol) and using 3.0 eq of TBDMSiOTf with the crude material purified using flash column chromatography

(40% ethyl acetate in DCM), alternatively, the crude oil can be triturated using EtOAc to afford the desired product as yellow coloured solid (0.372 g, quantitative yield).

Method 2) **10a** was synthesised according to General Procedure **D** using **8a** (4.31 g, 11.93 mmol) to afford the desired product as a bright yellow coloured solid (3.39 g, quantitative yield). $\mathbf{R_f}$= 0.41. **Mp**= 175-177 °C. $\mathbf{v_{max}}$ **(KBr) cm^{-1}** 1688 (N-C=O), 1641 (C=O). **^{1}H NMR** (400 MHz, Chloroform-*d*) δ 8.53 (d, 1H, Ar-H), 7.84 (ddd, *J* = 7.2, 1.5, 0.8 Hz, 1H, Ar-H), 7.68 – 7.57 (m, 2H, Ar-H), 3.97 (t, *J* = 7.9 Hz, 2H, C(4)*H*), 3.89 (s, 3H, OMe), 3.37 (t, *J* = 8.9 Hz, 2H, C(3)*H*). **^{13}C NMR** (126 MHz, Chloroform-*d*) δ 165.09 (COOMe), 164.05 (C=O), 149.17 (C=**C**), 136.26 (ArC), 132.26 (ArC), 131.55 (ArC), 129.62 (ArC), 126.77 (ArC), 123.52 (ArC), 110.31 (**C**=C), 51.95 (OMe), 40.09 (C(4)), 34.14 (C(3)). ***m/z***= 230 [MH+] (ESI$^+$) and 252 [MNa+] (ESI$^+$). **HRMS** calculated for $C_{13}H_{12}NO_3$ requires 230.0811, found 230.0812 [MH+] (ESI$^+$).

Methyl 8-nitro-6-oxo-3,4-dihydro-1H-benzo[a]pyrrolizine-2-carboxylate 10b

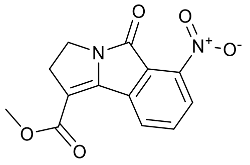

146b

Method 1) **10b** was synthesised according to General Procedure C from **5b** (0.300 g, 1.02mmol) using 3.0 eq of TBDMSiOTf with the crude oil triturated using EtOAc to afford the desired product as yellow coloured solid (0.283 g, quantitative yield).

Method 2) **10b** was synthesised according to General Procedure **D** using **8b** to afford the desired product as a bright yellow coloured solid (0.108 g, 98%).

Mp= 211-213 °C. $\mathbf{v_{max}}$ **(KBr) cm^{-1}** 1716 (N-C=O), 1694 (N-C=O), 1645 (C=O). **^{1}H NMR** (400 MHz, Chloroform-*d*) δ 8.87 (dd, *J* = 7.8, 1.0 Hz, 1H, Ar-H), 7.92 (dd, *J* = 8.1, 1.0 Hz, 1H, Ar-H), 7.78 (t, *J* = 7.9 Hz, 1H, Ar-H), 4.01 (t, *J* = 8.8 Hz, 2H, C(4)*H*), 3.90 (s, 3H, (OMe), 3.41 (t, *J* = 8.9 Hz, 2H, C(3)*H*). **^{13}C NMR** (101 MHz, Chloroform-*d*) δ 164.60 (COOMe), 158.79 (C=O), 146.41 (C=**C**), 145.79 (Ar**C**-NO_2), 133.06 (ArC), 131.48 (ArC), 130.60 (ArC), 127.38 (ArC), 125.88 (ArC), 113.26 (**C**=C), 52.30 (OMe), 40.63 (C(4)), 34.28 (C(3)). ***m/z***= 275 [MH+] (ESI$^+$) and 297 [MNa+] (ESI$^+$). **HRMS** calculated for $C_{13}H_{11}N_2O_5$ requires 275.0662, found 275.0661 [MH+] (ESI$^+$).

Methyl 8-fluoro-6-oxo-3,4-dihydro-1H-benzo[a]pyrrolizine-2-carboxylate 10c and **Methyl 9-fluoro-6-oxo-3,4-dihydro-1H-benzo[a]pyrrolizine-2-carboxylate 10c'**

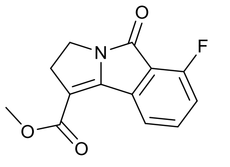

146f

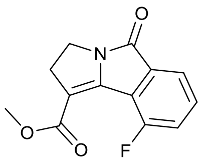

185

Method 1) **10c** and **10c'** were synthesised according to General Procedure C from **5c** (0.240 g, 0.95 mmol) using 3.0 eq of TBDMSiOTf with the crude oil triturated using pet-ether 40:60 (2 x 10 mL) followed by the addition of ethyl acetate (5 mL) resulting in precipitating to form a yellow-coloured solid. The solvent was removed and the remaining solid was triturated using ethyl acetate (20 mL) with the solid dried using reduced pressure to afford an inseparable mixture of regioisomers as a yellow coloured solid (0.364 g, quantitative yield). *r.r.* = 1.0: 0.1 for **10c:10c'**.

Method 2) **10c** and **10c'** were synthesised according to General Procedure **D** from a mixture of **8c** and **9** (0.349g, 0.92 mmol) to afford an inseparable mixture of products as a pale yellow coloured solid (0.347 g, quantitative yield). *r.r.* = 1.0: 0.33 for **10c** and **10c'. ν_{max} (KBr) cm^{-1}** 2951, 1693 (C=O), 1648 (N-C=O). ***m/z***= 248 [MH] (ESI$^+$) and 270 [MNa+] (ESI$^+$). **HRMS** calculated for $C_{13}H_{11}FNO_3$ requires 248.0726, found 248.0719 [MH+] (ESI$^+$).

10c ^{1}H NMR (400 MHz, Chloroform-*d*) δ 8.28 (d, *J* = 7.4 Hz, 1H, ArH), 7.63 – 7.54 (m, 1H, ArH), 7.20 (ddd, *J* = 9.1, 8.1, 0.7 Hz, 1H, ArH), 3.92 (t, *J* = 8.7 Hz, 2H, C(4)*H*), 3.84 (s, 3H, OMe), 3.32 (t, *J* = 8.7 Hz, 2H, C(3)*H*). **^{13}C NMR** (101 MHz, Chloroform-*d*) δ 164.70 (COOMe), 160.63 (C=O), 157.80 (d, *J* = 261.4 Hz, Ar**C**-F), 148.09 (d, *J* = 1.2 Hz, C=**C**), 134.17 (d, *J* = 7.9 Hz, ArC), 131.66 (d, *J* = 3.8 Hz, ArC) 122.90 (d, *J*= 3.5 Hz, ArC), 119.19 (d, *J* = 19.9 Hz, ArC), 110.88 (**C**=C), 51.83 (OMe), 40.06 (C(4)), 34.03 (C(3)).

10c and **10c'** ^{1}H NMR (400 MHz, Chloroform-*d*) δ 7.63 – 7.54 (m, 2H), 7.27 (ddd, *J* = 10.0, 8.1, 1.2 Hz, 1H), 3.92 (t, *J* = 8.7 Hz, 2H), 3.83 (s, 3H), 3.39 (t, *J* = 8.7 Hz, 1H, C(3)*H*).

Methyl 8-(benzyloxy)-6-oxo-3,4-dihydro-1H-benzo[a]pyrrolizine-2-carboxylate 10d

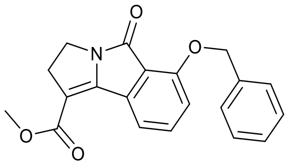

146j

Method 1) **10d** was synthesised according to General Procedure C from **5e** (0.180 g, 0.51 mmol) using 3.0 eq TBDMSiOTf with the crude oil triturated using pet-ether 40:60 (2 x 10 mL) followed by the addition of ethyl acetate (5 mL). The solvent was removed and the remaining solid was triturated using ethyl acetate (20 mL) to afford the desired product as a bright yellow coloured solid (0.089 g, 52%).

Method 2) **10d** was synthesised according to General Procedure D using **8d** (0.200 g, 0.42 mmol) to afford the desired product as a bright yellow coloured solid (0.149 g, quantitative yield).

Mp= 168-170 °C. **ν_{max} (KBr) cm^{-1}** 1678 (C=O), 1659 (N-C=O), 1256 (C-O). **^{1}H NMR** (500 MHz, Chloroform-*d*) δ 8.19 (dd, *J* = 7.6, 0.7 Hz, 1H, Ar-H), 7.56 – 7.50 (m, 3H, Ar-H), 7.42 – 7.37 (m, 3H, Ar-H), 7.34 – 7.30 (m, 1H, Ar-H), 7.10 (d, *J* = 8.3 Hz, 1H, Ar-H), 5.36 (s, 2H, C(8b)Ar-C**H**$_2$-O), 4.00 – 3.93 (m, 2H, C(4)*H*), 3.89 (s, 3H, OMe), 3.36 (t, *J* = 9.1 Hz, 2H, (C(3)*H*). **^{13}C NMR** (126 MHz, Chloroform-*d*) δ 165.18 (COOMe), 162.77 (C=O), 155.83 (Ar**C**-O-), 149.01 (C=**C**), 136.60 (CH$_2$-Ar**C**), 133.88 (ArC), 131.81 (ArC), 128.76 (BnC), 127.99 (ArC), 126.95 (BnC), 122.98 (ArC), 119.71 (ArC), 117.02 (ArC), 109.64 (C(2)**C**=C), 70.85 (O**C**H$_2$Bn), 51.83 (OMe), 40.10 (C(4)), 34.04 (C(3)). ***m/z***= 336 [MH+] (ESI$^+$). **HRMS** calculated for $C_{20}H_{18}NO_4$ requires 336.1230, found 336.1232 [MH+] (ESI$^+$).

Benzyl (2*R*,13*R*)-13-((tert-butyldimethylsilyl)oxy)-6-oxo-3,4,6,13-tetrahydro-1H-benzo[a]pyrrolizine-2-carboxylate 11

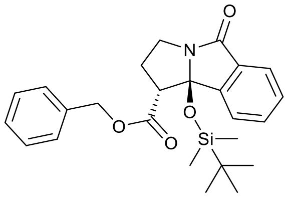

200

11 was synthesised according to General Procedure C using **7a** (0.368 g, 1.14 mmol) with the crude material purified using flash column chromatography (30% ethyl acetate in pet-ether 40:60) to afford the desired product as a colourless oil (0.418 g, 91%). **R_f**= 0.35 (30% ethyl acetate in pet-ether 40:60). **ν_{max} (KBr) cm^{-1}** 1716 (C=O), 1614 C=O). **^{1}H NMR** (400 MHz, Chloroform-*d*) δ 7.73 – 7.67 (m, 1H, Ar-H), 7.55 – 7.44 (m, 3H, Ar-H), 7.36 – 7.27 (m, 3H, Ar-H), 7.14 – 7.05 (m, 2H, Ar-H), 4.66 (d, *J* = 12.0 Hz, 1H, C(1a)H_A), 4.59 (d, *J* = 12.0 Hz, 1H, C(1a)H_B), 4.05 (dt, *J* = 11.0, 8.9 Hz, 1H, C(4)H_A), 3.51 (ddd, *J* = 11.3, 9.5, 2.1 Hz, 1H, C(4)H_B), 3.42 (dd, *J* = 6.9, 0.9 Hz, 1H, C(2)*H*), 2.79 (dtd, *J* = 13.1, 9.3, 6.9 Hz, 1H, C(3)H_a), 2.60 (dddd, *J* = 13.1, 8.6, 2.2, 0.9 Hz, 1H, C(3)H_B), 0.89 (s, 9H, (C(CH$_3$)$_3$), 0.00 (s, 3H, Si-CH$_3$), -0.45 (s, 3H, Si-CH$_3$). **^{13}C NMR** (101 MHz, Chloroform-*d*) δ 171.19 (COOMe), 170.37 (C=O), 144.33 (ArC), 135.12 (ArC), 132.96 (ArC), 132.27 (ArC), 130.16 (ArC), 128.76 (ArC), 128.59 (ArC), 128.40 (ArC), 123.69 (ArC), 123.43 (C(11)ArC), 99.18 (C(13)), 66.58 (C(1a)Bn-**C**H$_2$-), 53.04 (C(2)), 42.26 (C(4)), 31.34 (C(3)), 25.61 (C(**C**H$_3$)$_3$), 17.93 (Si-**C**(CH$_3$)$_3$), -4.20 (Si-CH$_3$), -4.40 (Si-CH$_3$). ***m/z***=460 (ESI$^+$) [MNa+]. **HRMS** calculated for $C_{25}H_{32}NO_4Si$ requires 438.2095, found 438.2096 (ESI$^+$) [MH+].

Methyl (2*R*,13*R*)-13-((tert-butyldimethylsilyl)oxy)-9-nitro-6-oxo-3,4,6,13-tetrahydro-1H-benzo[a]pyrrolizine-2-carboxylate 12a and **Methyl (2*R*,13*R*)-13-((tert-butyldimethylsilyl)oxy)-10-nitro-6-oxo-3,4,6,13-tetrahydro-1H-benzo[a]pyrrolizine-2-carboxylate 13a**

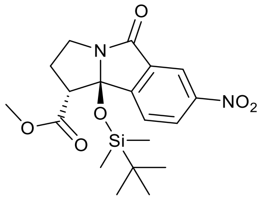

172a

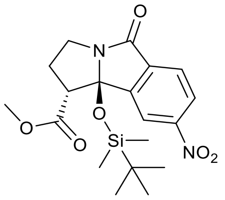

172b

12a and **13a** were synthesised according to General Procedure C using **6a** (0.100 g, 0.342 mmol) with the crude material purified using flash column chromatography (20% ethyl acetate in pet-ether 40:60). Only **12a** was cleanly isolated (0.015 g) but **13a** also contained **12a** (0.90 g, 72%).

12a R$_f$= 0.36. **Mp** 104-106 °C. **ν$_{max}$ (KBr) cm^{-1}** 1730 (C=O), 1714 (N-C=O). **^{1}H NMR** (500 MHz, Chloroform-*d*) δ 8.53 (d, *J* = 2.1 Hz, 1H, Ar-H), 8.43 (dd, *J* = 8.3, 2.1 Hz, 1H, Ar-H), 7.69 (d, *J* = 8.3 Hz, 1H, Ar-H), 3.99 (dt, *J* = 11.2, 8.8 Hz, 1H, C(4)H_A), 3.49 (ddd, *J* = 11.3, 9.4, 2.1 Hz, 1H, C(4)H_B), 3.40 – 3.34 (m, 1H, C(2)*H*), 3.22 (s, 3H, OMe), 2.76 (dtd, *J* = 13.2, 9.4, 7.1 Hz, 1H, C(3)H_A), 2.55 (ddt, *J* = 13.3, 8.2, 1.7 Hz, 1H, C(3)H_B), 0.85 (s, 9H, Si-C(C**H**$_3$)$_3$), -0.00 (s, 3H, Si-CH$_3$), -0.48 (s, 3H, Si-CH$_3$). **^{13}C NMR** (126 MHz, Chloroform-*d*) δ 171.23 (COOMe), 167.79 C=O), 150.29 (ArC), 149.71 (Ar**C**-NO$_2$), 134.70 (ArC), 127.41 (ArC), 124.61 (ArC), 119.01 (ArC), 98.57 (C(13)), 52.95 (C(2)), 51.91 (OMe), 42.63 (C(4)), 31.20 (C(3)), 25.51 (Si-C(**C**H$_3$)$_3$), 17.89 (Si-**C**(CH$_3$)$_3$), -4.11 (Si-CH$_3$), -4.13 (Si-CH$_3$). ***m/z***= 814 [2M+H+] (ESI$^+$) and 836 [2M+Na+] (ESI$^+$). **HRMS** calculated for $C_{19}H_{27}N_2O_6Si$ requires 407.1632, found 407.1629 [MH+] (ESI$^+$).

13a. **R$_f$**= 0.33. **ν$_{max}$ (KBr) cm^{-1}** 1726 (C=O), 1619 (N-C=O), 1535 (N-O$_2$). **^{1}H NMR** (400 MHz, Chloroform-*d*) δ 8.41 – 8.33 (m, 2H, Ar-H), 7.88 (d, *J* = 8.1 Hz, 1H, Ar-H), 4.05 – 3.92 (m, 1H, C(4)H_A), 3.50 (ddd, *J* = 11.3, 9.4, 2.1 Hz, 1H, C(4)H_B), 3.39 (t, *J* = 7.1 Hz, 1H, C(2)*H*), 3.23 (s, 3H, OMe), 2.78 (dtdd, *J* = 13.8, 9.3, 7.0, 4.6 Hz, 1H, (C(3)H_A), 2.61 – 2.51 (m, 1H, C(3)H_B), 0.86 (d, *J* = 2.3 Hz, 9H, C(CH$_3$)$_3$), -0.01 (d, *J* = 7.2 Hz, 3H, Si-CH$_3$), -0.48 (s, 3H, Si-CH$_3$). **^{13}C NMR** (101 MHz, Chloroform-*d*) δ 171.28 (COOMe), 167.82 (C=O), 150.57 (ArC) ,145.93 (Ar**C**-NO$_2$), 138.14 (ArC), 125.87 (ArC), 124.57 (ArC), 118.98 (ArC), 98.42 (C(13)), 52.72 (C(2)), 51.90 (OMe), 42.55 (C(4)), 31.25 (C(3)), 25.50 (C(**C**H$_3$)$_3$), 17.88 (Si-**C**(CH$_3$)$_3$), -4.12 (Si-CH$_3$), -4.14 (Si-CH$_3$). ***m/z***= 814 [2M+H+] (ESI$^+$) and 836 [2M+Na+] (ESI$^+$). **HRMS** calculated for $C_{19}H_{27}N_2O_6Si$ requires 407.1632, found 407.1628 [MH+] (ESI$^+$).

Methyl (2*R*,13*R*)-13-((tert-butyldimethylsilyl)oxy)-9-bromo-6-oxo-3,4,6,13-tetrahydro-1H-benzo[a]pyrrolizine-2-carboxylate 12b

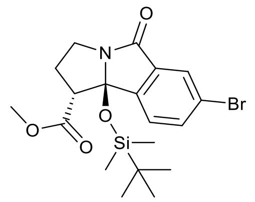

172c

12b was synthesised according to General Procedure C using **6b** (0.501 g, 1.54 mmol) with the crude material purified using flash column chromatography (20% ethyl acetate in pet-ether 40:60) to afford the desired products as a colourless solid (0.206 g, 30%). **R**$_f$= 0.43. **Mp**= 87-89 °C. **ν$_{max}$ (KBr) cm^{-1}** 1736 (C=O), 1722 (N-C=O). **^{1}H NMR** (400 MHz, Chloroform-*d*) δ 7.83 (d, *J* = 1.9 Hz, 1H, ArC), 7.67 (dd, *J* = 8.0, 1.8 Hz, 1H, ArC), 7.39 (d, *J* = 8.0 Hz, 1H, C(11)ArC), 3.96 (dt, *J* = 11.2, 8.8 Hz, 1H, C(4)H_A), 3.44 (ddd, *J* = 11.4, 9.5, 2.2 Hz, 1H, C(4)H_B), 3.31 (dd, *J* = 7.0, 1.0 Hz, 1H, C(2)*H*), 3.22 (s, 3H, OMe), 2.72 (dtd, *J* = 13.2, 9.3, 7.1 Hz, 1H, C(3)H_A), 2.52 (dddd, *J* = 13.2, 8.6, 2.3, 1.1 Hz, 1H, C(3)H_B), 0.85 (s, 9H, Si-C(CH_3)$_3$), -0.04 (s, 3H, Si-CH_3), -0.44 (s, 3H, Si-CH_3). **^{13}C NMR** (126 MHz, Chloroform-*d*) δ 171.56 (COOMe), 168.84 (C=O), 143.26 (Ar**C**-Br), 135.32 (ArC), 134.86 (ArC), 126.72 (ArC), 125.12 (ArC), 124.35 (ArC), 98.88 (C(13)), 53.02 (C(2)), 51.77 (OMe), 42.36 (C(4)), 31.21 (C(3)), 25.58 (Si-C(**C**H_3)$_3$), 17.93 (Si-**C**(CH_3)$_3$), -4.12 –(Si-CH_3), -4.16 (Si-CH_3). ***m/z***= 462 and 464 [MNa+] (ESI$^+$). **HRMS** calculated for $C_{19}H_{27}BrNO_4Si$ requires 440.0887 and 442.0867, found 440.0887 and 442.0866 [MH+] (ESI$^+$).

Methyl (2*R*,13*R*)-13-((tert-butyldimethylsilyl)oxy)-10-bromo-6-oxo-3,4,6,13-tetrahydro-1H-benzo[a]pyrrolizine-2-carboxylate 13b

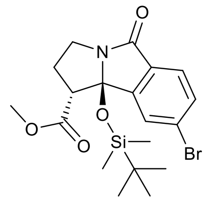

172d

13b was synthesised according to General Procedure C using **6b** (0.501 g, 1.54 mmol) with the crude material purified using flash column chromatography (20% ethyl acetate in pet-ether 40:60) to afford the desired products as a colourless solid (0.168 g, 25%). **R**$_f$= 0.34. **Mp**= 88-90 °C. **ν$_{max}$ (KBr) cm^{-1}** 1774 (C=O), 1715 (N-C=O). **^{1}H NMR** (500 MHz, Chloroform-*d*) δ 7.67 (d, *J* = 1.7 Hz, 1H, C(11)Ar-H), 7.62 (dd, *J* = 8.1, 1.7 Hz, 1H, Ar-H), 7.56 (d, *J* = 8.0 Hz, 1H, Ar-H), 3.95 (dt, *J* = 11.1, 8.8 Hz, 1H, C(4)H_A), 3.43 (ddd, *J* = 11.4, 9.5, 2.3 Hz, 1H, (C(4)H_B), 3.30 (dd, *J* = 7.0, 1.1 Hz, 1H, C(2)*H*), 3.22 (s, 3H, OMe), 2.71 (dtd, *J* = 13.2, 9.3, 7.1 Hz, 1H, C(3)H_A), 2.51 (dddd, *J* = 13.2, 8.5, 2.2, 1.1 Hz, 1H, C(3)H_B), 0.85 (s, 9H, Si-C(CH_3)$_3$), -0.04 (s, 3H, Si-CH_3), -0.44 (s, 3H, Si-CH_3). **^{13}C NMR** (126 MHz, Chloroform-*d*) δ 171.50 (COOMe), 169.46 (C=O), 146.36 (Ar**C**-Br), 133.56 (ArC),

131.83 ((ArC), 127.08 (C(11)ArC), 126.99 (ArC), 124.89 (ArC), 98.60 (C(13)), 53.04 (C(2)), 51.74 (OMe), 42.33 (C(4)), 31.11 (C(3)), 25.58 (Si-C(**C**H_3)$_3$), 17.92 (Si-**C**(CH_3)$_3$), -4.13 (Si-CH_3), -4.22 (Si-CH_3). ***m/z***= 462 and 464 [MNa+] (ESI$^+$). **HRMS** calculated for $C_{19}H_{27}BrNO_4Si$ requires 440.0887 and 442.0867, found 440.0888 and 442.0865 [MH+] (ESI$^+$).

Methyl (2*R*,13*R*)-13-((tert-butyldimethylsilyl)oxy)-9-fluoro-6-oxo-3,4,6,13-tetrahydro-1H-benzo[a]pyrrolizine-2-carboxylate 12c

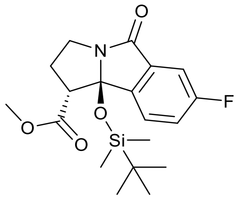

172e

12c was synthesised according to General Procedure C using **6c** (0.798 g, 3.01 mmol) with the crude material purified using flash column chromatography (25% ethyl acetate in pet-ether 40:60) to afford the desired product as a colourless solid (0.476 g, 41%). **R$_f$**= 0.61. **Mp**= 104-106 °C. **ν$_{max}$ (KBr) cm^{-1}** 1771 (C=O), 1719 (N-C=O). **^{1}H NMR** (500 MHz, Methanol-*d*$_4$) δ 7.63 (dd, *J* = 8.3, 4.5 Hz, 1H, C(11)ArC), 7.42 (ddd, *J* = 9.2, 8.3, 2.4 Hz, 1H, ArC), 7.36 (dd, *J* = 7.6, 2.4 Hz, 1H, ArC), 3.90 (dt, *J* = 11.1, 8.9 Hz, 1H, C(4)H_A), 3.46 (ddd, *J* = 11.4, 9.6, 2.1 Hz, 1H, C(4)H_B), 3.35 (d, *J* = 6.9 Hz, 1H, C(2)*H*), 3.20 (s, 3H, OMe), 2.77 (dtd, *J* = 13.3, 9.4, 7.0 Hz, 1H, C(3)H_A), 2.56 (dddd, *J* = 13.3, 8.5, 2.1, 0.9 Hz, 1H, C(3)H_B), 0.88 (s, 9H, C(CH_3)$_3$), -0.02 (s, 3H, Si-CH_3), -0.42 (s, 3H, Si-CH_3). **^{13}C NMR** (101 MHz, Methanol-*d*$_4$) δ 173.16 (COOMe), 170.95 (d, *J* = 3.2 Hz, C=O), 165.51 (d, *J* = 249.5 Hz, Ar**C**-F), 141.62 (d, *J* = 2.8 Hz, ArC), 136.29 (d, *J*= 8.7, ArC), 127.21 (d, *J* = 8.8 Hz, C(11)ArC), 121.05 (d, *J* = 23.8 Hz, ArC), 110.78 (d, *J* = 24.3 Hz, ArC), 100.19 (C(13)), 53.79 (C(2)), 52.04 (OMe), 43.22 (C(4)), 32.11 (C(3)), 25.96 (C(**C**H_3)$_3$), 18.68 (**C**(CH_3)), -3.99 (Si-CH_3), -4.02 (Si-CH_3). ***m/z***= 380 [MH+] (ESI$^+$). **HRMS** calculated for $C_{19}H_{27}FNO_4Si$ requires 380.1688, found 380.1687 [MH+] (ESI$^+$).

Methyl (2*R*,13*R*)-13-((tert-butyldimethylsilyl)oxy)-10-fluoro-6-oxo-3,4,6,13-tetrahydro-1H-benzo[a]pyrrolizine-2-carboxylate 13c

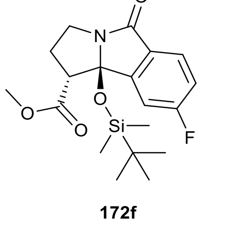

172f

13c was synthesised according to General Procedure C using **6c** (0.798 g, 3.01 mmol) with the crude material purified using flash column chromatography (25% ethyl acetate in pet-ether 40:60) to afford the desired product as a colourless solid (0.165 g, 14%). **R_f**= 0.48. **Mp**= 84-86 °C. **$ν_{max}$ (KBr) cm^{-1}** 1772 (C=O), 1718 (N-C=O). **^{1}H NMR** (400 MHz, Methanol-*d_4*) δ 7.73 – 7.66 (m, 1H, ArC), 7.38 – 7.28 (m, 2H, C(9,11)ArC), 3.89 (dt, *J* = 11.1, 8.5 Hz, 1H, C(4)*H_A*), 3.45 (ddd, *J* = 11.3, 9.5, 2.1 Hz, 1H, C(4)*H_B*), 3.36 (d, *J* = 6.9 Hz, 1H, C(2)*H*), 3.22 (s, 3H, OMe), 2.76 (dtd, *J* = 13.3, 9.4, 7.0 Hz, 1H, C(3)*H_A*), 2.55 (dddd, *J* = 13.3, 8.5, 2.1, 1.0 Hz, 1H, C(3)*H_B*), 0.89 (s, 9H, $C(CH_3)_3$), 0.00 (s, 3H, Si-CH_3), -0.42 (s, 3H, Si-CH_3). **^{13}C NMR** (101 MHz, Methanol-*d_4*) δ 173.02 (COOMe), 171.40 (C=O), 167.08 (d, *J* = 252.8 Hz, Ar**C**-F), 148.69 (d, *J* = 9.4 Hz, ArC), 130.04 (d, *J* = 2.4 Hz, ArC), 126.63 (d, *J* = 10.1 Hz, ArC), 118.98 (d, *J* = 23.8 Hz, ArC), 112.44 (d, *J* = 24.5 Hz, C(11)ArC), 99.87 (d, *J* = 2.6 Hz, C(13), 53.78 (C(2)), 52.09 (OMe), 43.33 (C(4)), 31.92 (C(3)), 25.94 (C(**C**H_3)$_3$), 18.66 (**C**(CH_3)$_3$), -4.01 (Si-CH_3), -4.16 (Si-CH_3). ***m/z***= 380 [MH+] (ESI^+). **HRMS** calculated for $C_{19}H_{27}FNO_4Si$ requires 380.1688, found 380.1688 [MH+] (ESI^+).

Dimethyl (2*R*,13*R*)-13-((tert-butyldimethylsilyl)oxy)-6-oxo-3,4,6,13-tetrahydro-1H-benzo[a]pyrrolizine-2,9-dicarboxylate 12d and **Dimethyl (2*R*,13*R*)-13-((tert-butyldimethylsilyl)oxy)-6-oxo-3,4,6,13-tetrahydro-1H-benzo[a]pyrrolizine-2,10-dicarboxylate 13d**

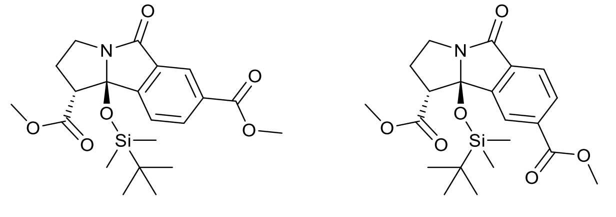

172g 172h

12d and **13d** were synthesised according to General Procedure C using **6d** (0.100 g, 0.33 mmol) with the crude material purified using flash column chromatography (20% ethyl acetate in pet-ether 40:60) to afford an inseparable mixture of the desired products as a colourless oil (0.132 g, 92%). **R_f**= 0.41 (ethyl acetate in pet-ether 40:60). **$ν_{max}$ (KBr) cm^{-1}** 1720 (N-C=O), 1624 (C=O). **^{1}H NMR** (500 MHz, Chloroform-*d*) δ 8.37 (dd, *J* = 1.5, 0.7 Hz, 1H, ArH), 8.26 (dd, *J* = 7.9, 1.5 Hz, 1H, ArH, 8.18 (d, *J* = 7.2 Hz, 2H, ArH), 7.80 – 7.73 (m, 1H, ArH), 7.60 (dd, *J* = 7.9, 0.7 Hz, 1H, ArH), 4.03 – 3.98 (m, 2H, C(4)*H_A*), 3.97 (s, ArC-COOMe), 3.95 (s, ArC-COOMe), 3.47 (ddd, *J* = 11.4, 9.4, 2.2 Hz, 2H, C(4)*H_B*), 3.36 (t, *J* = 6.6 Hz, 2H C(2)*H*), 3.17 (d, *J* = 0.8 Hz, 6H, OMe), 2.75 (dqd, *J* = 13.1, 9.1, 7.3 Hz, 2H, C(3)*H_A*), 2.53 (ddd, *J* = 13.3, 8.6, 2.1 Hz, 2H, C(3)*H_B*), 0.85 (d, *J* = 3.5 Hz, 18H, $C(CH_3)_3$), -0.05 (d, *J* = 10.8 Hz, 6H, Si-CH_3), -0.49 (d, *J* = 3.9 Hz, 6H, Si-CH_3). **^{13}C NMR** (126 MHz, Chloroform-*d*) δ 171.57 (C(O)OMe), 171.50 (C(O)OMe), 169.38 (C=O), 169.23 (C=O), 166.17 (OMe), 166.11 (OMe), 148.76 (ArC), 144.66 (ArC), 136.83 (ArC), 133.86 (ArC), 133.72 (ArC),

133.46 (ArC), 132.43 (ArC), 131.76 (ArC), 124.87 (ArC), 124.85 (ArC), 123.76 (ArC), 123.47 (ArC), 98.94 C(13)), 53.14 (OMe), 53.00 (OMe), 52.77 (OMe), 52.65 (OMe), 51.75 (C(2)), 51.72 (C(2)), 42.44 (C(4)), 42.36 (C(4)), 31.28 (C(3)), 31.15 (C(3)), 25.61 (C(**C**H_3)$_3$), 25.59 (C(**C**H_3)$_3$), 17.95 (Si-**C**(CH_3)$_3$), -4.10 (Si-CH_3), -4.15 (Si-CH_3), -4.23 (Si-CH_3). ***m/z***= 862 [2MNa+] (ESI$^+$). **HRMS** calculated for $C_{21}H_{30}NO_6Si$ requires 420.1836, found 420.1829 [MH+] (ESI$^+$).

Methyl (2*R*,13*R*)-13-((tert-butyldimethylsilyl)oxy)-9-methyl-6-oxo-3,4,6,13-tetrahydro-1H-benzo[a]pyrrolizine-2-carboxylate 12e and **Methyl (2*R*,13*R*)-13-((tert-butyldimethylsilyl)oxy)-10-methyl-6-oxo-3,4,6,13-tetrahydro-1H-benzo[a]pyrrolizine-2-carboxylate 13e**

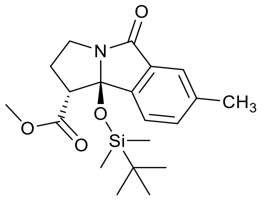

172i

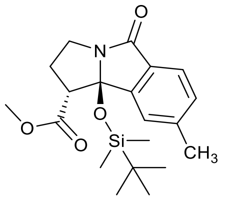

172j

12e and **13e** were synthesised according to General Procedure C using **6e** (0.500 g, 1.91 mmol) with the crude material purified using flash column chromatography (25% ethyl acetate in pet-ether 40:60) to give the product as a mixture of isomers (0.596 g, 85%). **R$_f$**= 0.54 (25% ethyl acetate in pet-ether 40:60) and 0.20 (DCM). ***m/z***= 376 [MH+] (ESI$^+$) and 398 [MNa+] (ESI$^+$). **HRMS** calculated for $C_{20}H_{30}NO_4Si$ requires 376.1938, found 376.1937 [MH+] (ESI$^+$).

12e ^{1}H NMR (400 MHz, Chloroform-*d*) δ 7.49 (dq, *J* = 1.7, 0.8 Hz, 1H), 7.45 – 7.36 (m, 1H, Ar-H), 7.35 – 7.29 (m, 1H, Ar-H), 7.26 (s, 1H, Ar-H), 3.98 – 3.90 (m, 1H (C(4)*H$_A$*), 3.42 (dddd, *J* = 11.7, 9.4, 2.3, 0.8 Hz, 1H, C(4)*H$_B$*), 3.36 – 3.27 (m, 1H, C(2)*H*), 3.16 (s, 3H, OMe), 2.70 (dtdd, *J* = 13.1, 9.3, 7.1, 1.3 Hz, 1H, C(3)*H$_A$*), 2.41 (s, 3H, C(C9)Ar-CH_3), 2.52 (m, 1H, C(3)*H$_B$*), 0.84 (d, *J* = 1.6 Hz, 9H, Si-C(CH_3)$_3$), -0.07 (d, *J* = 0.9 Hz, 3H, Si-CH_3), -0.48 (d, *J* = 1.2 Hz, 3H, Si-CH_3).

13e ^{1}H NMR (400 MHz, Chloroform-*d*) δ 8.39 – 8.33 (m, 1H, Ar-H), 7.56 (d, *J* = 7.7 Hz, 1H, Ar-H), 7.45 – 7.36 (m, 1H, Ar-H), 7.35 – 7.29 (m, 1H, Ar-H), 3.98 – 3.90 (m, 1H (C(4)*H$_A$*), 3.42 (dddd, *J* = 11.7, 9.4, 2.3, 0.8 Hz, 1H, C(4)*H$_B$*), 3.36 – 3.27 (m, 1H, C(2)*H*), 3.16 (d, *J* = 1.2 Hz, 3H, OMe), 2.70 (dtdd, *J* = 13.1, 9.3, 7.1, 1.3 Hz, 1H, C(3)*H$_A$*), 2.43 (d, *J* = 0.9 Hz, 2H, C(C10)Ar-CH_3), 2.52 (m, 2H, C(3)*H$_B$*), 0.84 (d, *J* = 1.6 Hz, 9H, Si-C(CH_3)$_3$), -0.07 (d, *J* = 0.9 Hz, 3H, Si-CH_3), -0.48 (d, *J* = 1.2 Hz, 3H, Si-CH_3).

***(S)*-4-(6,13-Dioxoisoindolin-5-yl)pentanedioic acid 14a**

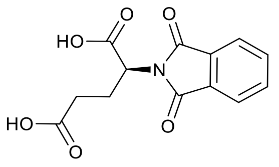

125L

Phthalic anhydride (1.00 g, 6.80 mmol) was added to L-glutamic acid (1.00 g, 6.80 mmol) and were heated to 170 °C for 6 hours while stirring. The reaction was left to cool to room temperature with the resulting solid mass dissolved in ethyl acetate (10 mL). The organic layer was washed using 0.5 N HCl (2 x 10 mL) with the combined aqueous layers being back extracted with ethyl acetate (20 mL). The organic layers were then combined and concentrated by reduced pressure to afford the desired product as a beige coloured solid (1.14 g, 60%). $[\alpha]_D^{25}$= -18.8 (*c* 1.0 in MeOH). **Mp**= 184-186 °C (lit.[10] 159-160 °C). **ν_{max} (KBr) cm^{-1}** 1773 (C=O), 1711 (C=O), 1654 (C=O). **^{1}H NMR** (400 MHz, Methanol-d_4) δ 7.88 – 7.84 (dd, *J* = 5.5, 2.7 Hz, 2H, ArH), 7.84 – 7.79 (dd, *J* = 5.4, 2.8 Hz, 2H, ArH), 4.93 (dd, *J* = 10.3, 4.8 Hz, 1H, C(4)*H*), 2.62 – 2.42 (m, 2H, C(2)*H*), 2.42 – 2.34 (m, 2H, C(3)*H*). **^{13}C NMR** (101 MHz, Methanol-d_4) δ 176.17 (C=O), 172.20 (C(4a)C=O), 169.20 (C=O), 135.65(ArC), 132.01 (C(7,12)ArC), 124.38 (ArC), 52.54 (C(4)), 31.56 (C2)), 25.24 (C3)). ***m/z***= 276 [MH-] (ESI$^-$) and 300 [MNa+] (ESI$^+$). **HRMS** calculated for $C_{13}H_{11}NNaO_6$ requires 300.0478, found 300.0479 [MNa+] (ESI$^+$).

(*S*)-4-(6,13-Dioxoisoindolin-5-yl)-4-methoxy-1-oxopentanoic acid 14b

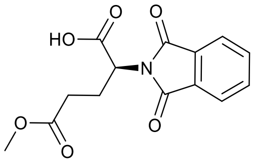

126L

14b was synthesised according to General Procedure C using phthalic anhydride (0.100 g, 0.67 mmol) and L-glutamic acid 5-methyl ester (0.108 g, 0.67 mmol) to afford the desired product as a pale-yellow coloured oil (0.141 g, 72%). $[\alpha]_D^{25}$= -33.0 (*c* 1.0 MeOH). **ν_{max} (KBr) cm^{-1}** 1774 (C=O), 1708 (N-C=O), 1438, 1387, 1263 (C=C), 1202 (C-O). **^{1}H NMR** (500 MHz, Chloroform-*d*) δ 7.89 (dd, 2H, Ar-H), 7.78 (dd, 2H, Ar-H), 5.01 (dd, *J* = 10.3, 5.0 Hz, 1H, C(2)*H*), 3.65 (s, 3H, OMe), 2.70 – 2.60 (m, 1H, C(4)H_A), 2.59 – 2.40 (m, 3H, C(4)H_B, C(3)*H*). **^{13}C NMR** (126 MHz, Chloroform-*d*) δ 173.42 (COOH), 172.63 (C(4a)COOH), 167.58 (), 167.48 (ArC), 134.42 (ArC), 131.73 (ArC), 123.77 (ArC), 51.86 (C(4)), 50.91 (C(1a)OMe), 30.64 (C(2)), 24.09 (C3)). ***m/z***= 314 [MNa+] (ESI$^+$), 290 [MH-] (ESI$^-$). **HRMS** calculated for $C_{14}H_{13}NaNO_4$ requires 314.0635, found 314.0631 [MNa+] (ESI$^+$).

Dimethyl (*S*)-4-(6,13-dioxoisoindolin-5-yl)pentanedioate 14c

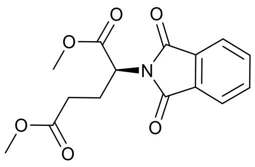

127L

Method 1) Phthalic anhydride (0.200 g, 1.32 mmol) was added to L-glutamic acid dimethyl ester (0.236 g, 1.32 mmol) and heated at 150 °C for 6 hours while stirring. The reaction was left to cool to room temperature with the resulting solid mass dissolved in DCM (10 mL). The organic layer was washed using 0.5N HCl (2x 10 mL) with the combined aqueous layers being back extracted with DCM (20 mL). The organic layers were then combined and concentrated by reduced pressure to afford the desired product as a colourless oil (0.166 g, 40%).

Method 2) Compound **14a** (0.936 g, 3.37 mmol) was dissolved in MeOH (10 mL) and cooled to 0 °C using an ice bath. $SOCl_2$ (2.026 mL, 10.11 mmol) was added dropwise over 5 minutes after which the reaction was allowed to warm to room temperature and left stirring for 16 hours to ensure completion. The solvent mixture was removed by reduced pressure then azeotroped using DCM (3 x 20 mL). The crude material was then purified *via* flash column chromatography (30% ethyl acetate in pet ether 40:60) with the product containing fractions being collected, combined and concentrated to afford the desired product as a colourless oil (0.312 g, 30%).

R_f= 0.40 (30% ethyl acetate in pet-ether 40:60). $[\boldsymbol{\alpha}]_{\mathbf{D}}^{\mathbf{25}}$= -35.1 (*c* 1.0 in DCM); {Lit.[11] $[\alpha]_D^{20}$= -58 (*c* 1.0 in DMF)}.[315] **ν_{max} (KBr) cm^{-1}** 1776 (C=O), 1736 (C=O), 1713 N-C=O). **^{1}H NMR** (400 MHz, Chloroform-*d*) δ 7.90 (dd, *J* = 5.4, 3.0 Hz, 2H, Ar-H), 7.78 (dd, *J* = 5.3, 2.9 Hz, 2H, AR-H), 4.96 (dd, *J* = 10.2, 5.0 Hz, 1H, C(2)*H*), 3.77 (s, 3H, OMe), 3.64 (s, 3H, OMe), 2.73 – 2.60 (m, 1H, C(4)H_A), 2.58 – 2.46 (m, 1H, C(4)H_B), 2.45 – 2.37 (m, 2H, C(3)*H*). **^{13}C NMR** (101 MHz, Chloroform-*d*) δ 172.72 (COOMe), 169.36 (C(4a)COOMe), 167.64 (C(9,16)C=O), 134.42 (ArC), 131.89 (C(7,12)ArC), 123.75 (ArC), 52.96 (OMe), 51.87 (OMe) , 51.32 (C(4)), 30.79 (C(2)), 24.44 (C(3)). ***m/z***= 328 [MNa+] (ESI^+). **HRMS** calculated for $C_{15}H_{16}NO_6$ requires 306.0972, found 306.0974 [MH+] (ESI^+).

4a-Benzyl 1-methyl (*S*)-4-(6,13-dioxoisoindolin-5-yl)pentanedioate 14d

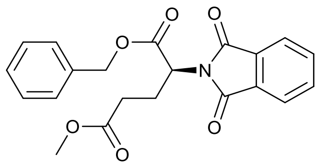

142

Compound **14b** (6.04 g, 20.75 mmol) was dissolved in anhydrous DCM (50 mL) followed by the addition of DCC (4.07 g, 22.82 mmol) and DMAP (0.253 g, 10% mol) with the resulting mixture left stirring for 1 hour at room temperature. Benzyl alcohol (2.35 mL, 22.82 mmol) was then added with

the reaction mixture left stirring at room temperature for an additional 16 hours. The crude mixture was diluted with DCM (20 mL) and washed with water (2 x 50 mL) with the organic layer dried over $MgSO_4$ and concentrated by reduced pressure. The crude mixture was then purified using flash column chromatography (0-30% ethyl acetate in pet-ether 40:60) to afford the desired product as a colourless oil (2.98 g, 38%).

$[\alpha]_D^{25}$ = -22.6 (*c* 1.0 in DCM). **Mp**= 103-105 °C. **R_f**= 0.15 (35% ethyl acetate in pet-ether 40:60). **ν_{max} (KBr) cm^{-1}** 1774 (C=O), 1716 (N-C=O). **^{1}H NMR** (400 MHz, Chloroform-*d*) δ 7.86 (ddd, *J* = 5.4, 3.0, 1.8 Hz, 2H, Ar-H), 7.75 (td, *J* = 5.5, 3.0 Hz, 3H, Ar-H), 7.37 – 7.27 (m, 4H, Ar-H), 5.19 (d, *J* = 5.5 Hz, 1H, PhC*H*), 5.18 (d, *J* = 5.5 Hz, 1H, PhC*H*), 4.98 (dd, *J* = 10.3, 5.1 Hz, 1H, C(4)*H*), 3.60 (s, 3H, OMe), 2.70 – 2.60 (m, 1H, C(2)H_A), 2.53 (dddd, *J* = 14.3, 10.3, 7.4, 6.1 Hz, 1H, C(2)H_B), 2.42 – 2.35 (m, 2H, C(3)*H*). **^{13}C NMR** (101 MHz, Chloroform-*d*) δ 172.71 (COOMe), 168.77 (C(4a)COOBn), 167.66 (C6,13)C=O), 135.26 (ArC)Bn), 134.40 (C(8,11)Ar), 131.85 (C(7,12)ArC), 128.66 (ArC)Bn), 128.47 (ArC)Bn), 128.23 (ArC)Bn), 123.72 (C(9,10)Ar), 67.74 (C(4a)Bn-**C**H_2-), 51.87 (C(4)), 51.59 (OMe), 30.80 (C(2)), 24.41 (C(3)). ***m/z***= 404 (ESI$^+$) [MNa+]. **HRMS** calculated for $C_{21}H_{19}NaNO_6$ requires 404.1104, found 404.1104 [MNa+] (ESI$^+$).

(*S*)-1-(Benzyloxy)-4-(6,13-dioxoisoindolin-5-yl)-4-oxopentanoic acid 14e[1213]

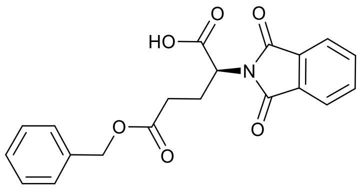

130

Phthalic anhydride (1.550 g, 10.47 mmol) was added to L-glutamic acid 5-benzyl ester (2.500 g, 10.47 mmol) and heated at 170 °C for 6 hours while stirring. The reaction was left to cool to room temperature with the resulting solid mass dissolved in DCM (10 mL). The organic layer was washed using 0.5 N HCl (2x 10 mL) with the combined aqueous layers being back extracted with DCM (20 mL). The organic layers were then combined and concentrated by reduced pressure to afford the desired product as a light brown coloured oil (2.78 g, 72%). ^{1}**H NMR** (400 MHz, Chloroform-*d*) δ 7.85 (dd, *J* = 5.5, 3.0 Hz, 2H, ArH), 7.73 (dd, *J* = 5.5, 3.1 Hz, 2H, ArH), 7.36 – 7.27 (m, 5H, ArH), 5.06 (s, 2H, C**H_2**O), 4.99 (ddd, *J* = 10.5, 5.0, 1.6 Hz, 1H, C(4)*H*), 2.72 – 2.59 (m, 1H, (C(4)H_A), 2.60 – 2.49 (m, 1H, C(4)H_B), 2.49 – 2.38 (m, 2H, C(3*)H). **m/z***= 366 [MH-] (ESI$^-$). **HRMS** calculated for $C_{20}H_{17}NaNO_6$ requires 390.0948, found 390.0944 [MNa+] (ESI$^+$).

1-Benzyl 4-methyl (*S*)-4-(6,13-dioxoisoindolin-5-yl)pentanedioate[14] 14f

Compound **14e** (3.70 g, 10.08 mmol) was suspended in DMF (20 mL) followed by the addition of Cs_2CO_3 (1.63 g, 5.04 mmol) with the reaction mixture left stirring at room temperature for 5 minutes resulting in the starting material dissolving. MeI (2.18 mL, 35.28 mmol) was slowly added over 5 minutes with the reaction mixture left stirring at room temperature for 2 hours. The reaction mixture was diluted with ethyl acetate (50 mL) and washed using warm water (40 °C, 5 x 50 mL) with the organic solvent concentrated by reduced pressure to afford the desired product as a colourless oil (1.64 g, 40%). **ν_{max} (KBr) cm^{-1}** 3033, 2954, 1775 (C=O), 1713 (N-C=O). **^{1}H NMR** (400 MHz, Chloroform-*d*) δ 7.90 – 7.82 (m, 2H, ArH), 7.77 – 7.71 (m, 2H, ArH), 7.37 – 7.27 (m, 5H, ArH), 5.05 (s, 2H, PhCH_2), 4.94 (dd, J = 10.0, 5.0 Hz, 1H, C(4)H), 3.73 (s, 3H, C(4a)OMe), 2.70 – 2.60 (m, 1H, C(4)H_A), 2.56 – 2.47 (m, 1H, C(4)H_B), 2.47 – 2.41 (m, 2H, C(4)H). **^{13}C NMR** (101 MHz, Chloroform-*d*) δ 172.10 (C(4a)COOMe), 169.31 (BnCH_2-**C**=O), 167.60 (C=O), 135.78 (C(1b)CH_2-**C**Ar), 134.39 (ArC) 131.82 (C(7,12)ArC), 128.63 (ArC), 128.37 (ArC), 123.71 (ArC), 66.61 (C(1b)ArC-**CH_2**-), 52.94 (C(4a)OMe), 51.26 (C(4)), 30.95 (C(2)), 24.35 (C(3)).

Methyl 2-(2-aminophenyl)acetate.[15]

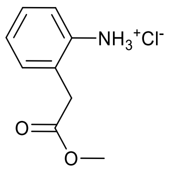

139

2-Aminophenylacetic acid (0.470 g, 3.11 mmol) was dissolved in MeOH (10 mL) in a round bottomed flask and cooled down to 0 °C using an ice bath. $SOCl_2$ (0.68 mL, 6.22 mmol) was added dropwise over 5 minutes after which the reaction was allowed to warm to room temperature and left stirring for 16 hours to ensure completion. The solvent mixture was removed by reduced pressure then azeotroped using DCM (3 x 20 mL) to afford the desired product as a beige coloured solid (0.536 g, quantitative yield). Mp= 110-112 °C (Lit. 105-108 °C). ν_{max} (KBr) cm^{-1} 2805 (NH_2 Salt), 1725 (C=O), 1622, 1565, 1519, 1493, 1472, 1458, 1439, 1342 (Ar-N), 1298, 1245, 1167 (C-O), 1116, 1005. ^{1}H NMR (400 MHz, Methanol-d_4) δ 7.46 (m, 4H, Ar-H), 3.92 (s, 2H, C(2)H), 3.75 (s, 3H, C(1)OMe). ^{13}C NMR (101 MHz, Methanol-d_4) δ 180.04 (C(1)COOMe), 133.58 (ArC-NH_3), 130.61 (ArC), 130.30 (ArC), 128.84 (ArC), 125.56 (ArC), 124.98 (ArC), 123.25 (ArC), 49.85 (C(1)OMe). *m/z*= 199 [MCl+] (ESI$^+$). HRMS calculated for $C_8H_{10}NO_2$ requires 166.0863, found 166.0864.

Methyl 1-(4-(6,13-dioxoisoindolin-4-yl)phenyl)acetate 15b[16]

Methyl 2-(2-aminophenyl)acetate (0.300 g, 1.81 mmol) was added to phthalic anhydride (0.269 g, 1.81 mmol) and heated at 170 °C for 6 hours while stirring. The reaction was left to cool to room temperature with the resulting solid mass dissolved in DCM (10 mL). The organic layer was washed

using 0.5 N HCl (2 x 10 mL) with the combined aqueous layers being back extracted with DCM (20 mL). The crude mixture was purified using flash column chromatography (20-40% ethyl acetate in pet-ether 40:60) to afford the desired product as a red/orange coloured solid (0.216 g, 44%). **R_f**= 0.37 (in 40% ethyl acetate in pet-ether 40:60). **ν_{max} (KBr) cm^{-1}** 1717 (C=O). **^{1}H NMR** (500 MHz, Chloroform-*d*) δ 7.99 (dd, *J* = 5.4, 3.1 Hz, 2H, Ar-H), 7.83 (dd, *J* = 5.4, 3.1 Hz, 2H, Ar-H), 7.52 – 7.43 (m, 3H, Ar-H), 7.28 – 7.24 (m, 1H (Ar-H), 3.62 (s, 2H, (C(2)*H*), 3.53 (s, 3H, OMe). **^{13}C NMR** (126 MHz, Chloroform-*d*) δ 170.69 (COOMe), 167.29 (C=O), 134.42 (ArC), 133.07 (C(4)Ar**C**-phthalimide), 131.95 (ArC), 131.73 (ArC), 130.76 (ArC), 129.76 (ArC), 129.40 (ArC), 128.58 (ArC), 123.82 (ArC), 52.11 (OMe), 38.21 (C(2)). ***m/z***= 294 [MH-] (ESI^-), 318 [MNa+] (ESI^+). **HRMS** calculated for $C_{17}H_{13}NaNO_4$ requires 318.0736, found 318.0734 [MNa+] (ESI^+).

2,4-Dimethyl (2*S*,4*S*,13*S*)-13-[(tert-butyldimethylsilyl)oxy]-6-oxo-3,4,6,13-tetrahydro-1H-benzo[a]pyrrolizine-2,4-dicarboxylate 16a and **2,4-Dimethyl (2*R*,4*S*,13*R*)-13-[(tert-butyldimethylsilyl)oxy]-6-oxo-3,4,6,13-tetrahydro-1H-benzo[a]pyrrolizine-2,4-dicarboxylate 16a'**

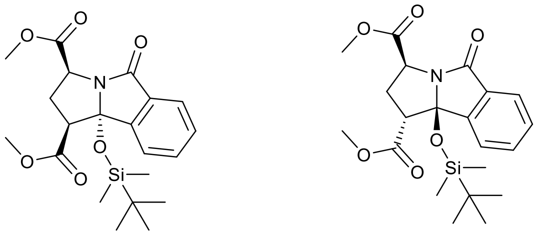

175La 175Lb

16a and **16a'** were synthesised according to General Procedure C using **14c** with the crude material purified using flash column chromatography (20% ethyl acetate in pet-ether 40:60) to afford an inseparable mixture of isomers with the desired products as a colourless solid that showed presence of SM remaining (0.068 g, 79%). *d.r* = 1: 0.29 for **16a:16a'**. **R_f**= 0.31 (20% ethyl acetate in pet-ether 40:60). ***m/z***= 420 [MH+] (ESI^+). **HRMS** calculated for $C_{21}H_{30}NO_6Si$ requires 420.1836, found 420.1826 [MH+] (ESI^+).

16a ^{1}H NMR (500 MHz, Chloroform-*d*) δ 7.71 (dt, *J* = 7.5, 1.0 Hz, 1H, ArH), 7.59 – 7.54 (m, 1H, ArH), 7.54 – 7.45 (m, 2H, ArH), 4.35 (dd, *J* = 8.9, 3.4 Hz, 1H, C(4)*H*), 3.77 (s, 3H, OMe), 3.43 (dd, *J* = 8.1, 2.2 Hz, 1H, C(2)*H*), 3.35 (s, 3H, C(4a)OMe), 3.10 – 3.03 (m, 1H, C(3)H_A), 2.92 (ddd, *J* = 13.6, 3.4, 2.3 Hz, 1H, C(3)H_B), 0.83 (d, *J* = 1.9 Hz, 9H, $C(CH_3)_3$), -0.07 (s, 3H, Si-CH_3), -0.55 (s, 3H, Si-CH_3). **^{13}C NMR** (126 MHz, Chloroform-*d*) δ 169.99 (COOMe), 169.95 (C(4a)COOMe), 167.54 (C=O), 145.55 (ArC), 132.79 (ArC), 132.47 (ArC), 129.79 (ArC), 123.90 (ArC), 123.78 (ArC), 99.29 (C(13)), 55.33 (C(4)), 52.69 (C(2)), 52.61 (OMe), 51.86 (C(4a)OMe), 36.07 (C(3)), 25.64 (C(**C**H_3)$_3$), 17.93 (**C**(CH_3)$_3$), -4.03 (Si-CH_3), -4.44 (Si-CH_3).

16a' ^{1}H NMR (500 MHz, Chloroform-*d*) δ 7.71 (dt, *J* = 7.4, 1.0 Hz, 1H, ArH), 7.61 – 7.55 (m, 1H, ArH), 7.54 – 7.45 (m, 2H, ArH), 4.86 (t, *J* = 9.0 Hz, 1H, C(4)*H*), 3.80 (s, 3H, OMe), 3.40 (d, *J* = 6.8 Hz, 1H, C(2)*H*), 3.14 (s, 3H, C(4a)OMe), 3.02 – 2.97 (m, 1H, C(3)H_A), 2.83 (dd, *J* = 13.4, 8.5 Hz, 1H, C(3)H_B), 0.83 (d, *J* = 1.9 Hz, 9H, $C(CH_3)_3$), -0.10 (s, 3H, Si-CH_3), -0.38 (s, 3H, Si-CH_3). **^{13}C NMR** (126 MHz, Chloroform-*d*) δ 171.76 (COOMe), 171.32 (C(4a)COOMe), 170.50 (C=O), 143.71 (ArC), 134.45 (ArC), 132.79 (ArC), 132.41 (ArC), 130.57 (ArC), 124.43 (ArC), 99.47 (C(13)), 53.89 (C(4)) 53.78 (C(2))), 52.61 (OMe), 51.73 (C(4a)OMe), 34.49 (C(3)), 25.73 (C(**C**H_3)$_3$), 18.13 (**C**(CH_3)$_3$), -3.41 (Si-CH_3), -4.16 (Si-CH_3).

4-Benzyl 2-methyl (2*S*,3*S*,13*S*)-13-((tert-butyldimethylsilyl)oxy)-6-oxo-3,4,6,13-tetrahydro-1H-benzo[a]pyrrolizine-2,4-dicarboxylate 16b

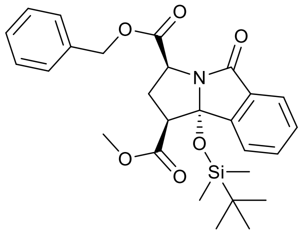

177

16b was synthesised according to General Procedure C using **14d** (0.100 g, 0.26 mmol) with 3.0 eq of TBDMSiOTf with the crude material purified using flash column chromatography (30% ethyl acetate in pet-ether 40:60) to afford the desired product as a pale yellow coloured solid (0.097 g, 75%). $[\alpha]_D^{25}$= -21.2 (*c* 1.0 in DCM). **R_f**= 0.29. **Mp**= 113-115 °C. **ν_{max} (KBr) cm^{-1}** 1719 (C=O). **^{1}H NMR** (400 MHz, Chloroform-*d*) δ 7.77 – 7.69 (m, 1H, ArH), 7.57 (td, *J* = 7.4, 1.1 Hz, 1H, ArH), 7.53 – 7.44 (m, 2H, ArH), 7.40 – 7.27 (m, 5H, ArH), 5.24 (d, *J* = 12.3 Hz, 1H, Ar-C**H**$_2$-), 5.15 (d, *J* = 12.3 Hz, 1H, Ar-C**H**$_2$-)), 4.40 (dd, *J* = 9.0, 2.9 Hz, 1H, C(4)*H*), 3.41 (dd, *J* = 8.0, 1.9 Hz, 1H, C(2)*H*), 3.18 (s, 3H, OMe), 3.09 (ddd, *J* = 13.6, 9.0, 8.0 Hz, 1H, C(3)H_A), 2.93 (dt, *J* = 13.6, 2.4 Hz, 1H, C(3)H_B), 0.82 (s, 9H, $C(CH_3)_3$), -0.08 (s, 3H, Si-CH_3), -0.55 (s, 3H, Si-CH_3). **^{13}C NMR** (101 MHz, Chloroform-*d*) δ 169.68 (COOMe), 169.32 (C(4a)COOMe), 167.55 (C=O), 145.68 (ArC), 135.58 (ArC), 134.39 (ArC), 132.86 (ArC), 132.42 (ArC), 129.71 (ArC), 128.59 (ArC), 128.39 (ArC), 124.02 (ArC), 123.75 (ArC), 123.68 (ArC), 99.34 (C(13)), 67.48 (Ar-**C**H_2-O), 55.42 (C(4)), 52.56 (C(2)), 51.66 (OMe), 35.97 (C(3)), 25.61 (C(**C**H_3)$_3$), 17.91 (**C**(CH_3)$_3$, -4.07 (Si-CH_3), -4.46 (Si-CH_3). ***m/z***= 496 [MH+] (ESI$^+$) and 518 [MNa+] (ESI$^+$). **HRMS** calculated for $C_{27}H_{34}NO_6Si$ requires 496.2149, found 496.2143 [MH+] (ESI$^+$).

2-Benzyl 4a-methyl (2*S*,4*S*,13*S*)-13-((tert-butyldimethylsilyl)oxy)-6-oxo-3,4,6,13-tetrahydro-1H-benzo[a]pyrrolizine-2,4-dicarboxylate 16c and **2-Benzyl 4a-methyl (2*R*,4*S*,13*R*)-13-((tert-**

butyldimethylsilyl)oxy)-6-oxo-3,4,6,13-tetrahydro-1H-benzo[a]pyrrolizine-2,4-dicarboxylate 16c'

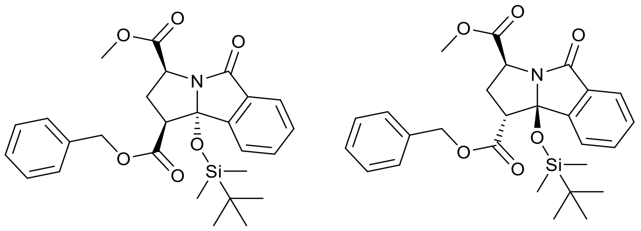

176a **176b**

16c and **16c'** were synthesised according to General Procedure C using **14f** (0.895 g, 2.34 mmol) with 3.0 eq of TBDMSiOTf with the crude material purified using flash column chromatography (30% ethyl acetate in pet-ether 40:60) to afford an inseparable mixture of isomers (1.038 g, 89%). *d.r*= 1: 0.6 for **16c:16c'**. **R_f**= 0.48. **ν_{max} (KBr) cm^{-1}** 1721 (C=O). ***m/z***= 496 [MH+] (ESI$^+$). **HRMS** calculated for $C_{27}H_{34}NO_6Si$ requires 496.2150, found at 496.2145 [MH+] (ESI$^+$).

16c ^{1}H NMR (400 MHz, Methanol-*d*$_4$) δ 7.57 (dt, *J* = 7.2, 1.2 Hz, 1H, ArH), 7.54 – 7.47 (m, 2H, ArH), 7.47 – 7.41 (m, 1H, ArH), 7.31 – 7.21 (m, 3H, ArH), 7.07 – 6.97 (m, 2H, ArH), 4.83 – 4.75 (m, 2H, ArC**H**$_2$-O), 4.37 (dd, *J* = 9.0, 3.1 Hz, 1H, C(4)*H*), 3.66 (s, 3H, OMe), 3.55 (dd, *J* = 8.0, 2.0 Hz, 1H, C(2)*H*), 3.15 (ddd, *J* = 13.7, 9.0, 7.9 Hz, 1H, C(3)H_A), 3.03 – 2.84 (m, 1H, C(3)H_B), 0.84 (s, 9H, C(CH_3)$_3$), -0.07 (s, 3H, Si-CH_3), -0.57 (s, 3H, Si-CH_3).**^{13}C NMR** (101 MHz, Methanol-*d*$_4$) δ 172.09 (C=O), 171.17 (COOBn), 170.70 (C(4a)COOMe), 146.92 (ArC), 136.55 (C(1c)OCH$_2$-**C**Ar), 133.96 (ArC), 133.76 (ArC), 130.95 (ArC), 129.55 (ArC), 129.51 (ArC), 129.40 (ArC), 125.65 (ArC), 124.22 (ArC), 100.77 (C(13), 67.87 (C(1b)ArC-**C**H$_2$-O), 56.42 (C(4)), 54.45 (C(2)), 52.87 (OMe), 37.09 (C(3), 25.98 (**C**(CH_3)$_3$), 18.64 (**C**(CH_3)$_3$), -3.96 (Si-CH_3), -4.28 (Si-CH_3).

16c' ^{1}H NMR (400 MHz, Methanol-*d*$_4$) δ 7.64 – 7.60 (m, 1H, ArH), 7.54 – 7.47 (m, 2H, ArH), 7.47 – 7.41 (m, 1H, ArH), 7.31 – 7.21 (m, 3H, ArH), 7.07 – 6.97 (m, 2H, ArH), 4.83 – 4.75 (m, 1H, C(4)*H*), 4.66 – 4.53 (dd, *J* = 15.2, 12.1 Hz, 2H, ArC**H**$_2$-O), 3.78 (s, 3H, OMe), 3.47 – 3.43 (m, 1H C(2)*H*), 3.03 – 2.84 (m, 2H, C(3)*H*), 0.84 (s, 9H, C(CH_3)$_3$), -0.11 (s, 3H, Si-CH_3), -0.35 (s, 3H, CH_3). **^{13}C NMR** (101 MHz, Methanol-*d*$_4$) δ 172.67 (C(4a)COOMe), 172.35 (C=O), 169.49 (COOBn), 144.69 (ArC), 136.37 (C(1c)OCH$_2$-**C**Ar), 134.03 (ArC), 133.42 (ArC), 131.96 (ArC), 129.71 (ArC), 129.35 (ArC), 129.18 (ArC), 126.03 (ArC), 124.42 (ArC), 100.79 (C(13)), 67.70 (C(1b)ArC-**C**H$_2$-O), 57.98 (C(4)), 54.79 (C(2)), 53.45 (C91)OMe), 35.81 (C(3)), 26.18 (C(**C**H$_3$)$_3$), 18.89 (**C**(CH_3)$_3$), -3.10 (Si-CH_3), -3.86 (Si-CH_3).

Methyl (2*R*,17*R*)-17-((tert-butyldimethylsilyl)oxy)-10-oxo-3,8-dihydro-1H-carboxylate[a]pyrrolizine-2-carboxylate 17

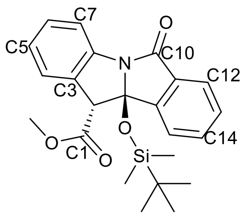

178

17 was synthesised according to General Procedure C using **15b** (0.181 g, 0.61 mmol) with the crude material purified using flash column chromatography (40% ethyl acetate in pet-ether 40:60) to afford the desired product as yellow coloured solid (0.098 g, 39%). **R$_f$**= 0.82. **ν$_{max}$ (KBr) cm^{-1}** 1728 (N-C=O). **^{1}H NMR** (400 MHz, Methanol-*d*$_4$) δ 7.81 (dt, *J* = 7.6, 1.0 Hz, 1H, Ar-H), 7.75 (td, *J* = 7.5, 1.2 Hz, 1H, Ar-H), 7.70 – 7.60 (m, 3H, Ar-H), 7.44 (td, *J* = 7.7, 1.3 Hz, 1H, Ar-H), 7.39 (ddt, *J* = 7.6, 1.4, 0.7 Hz, 1H, Ar-H), 7.22 (td, *J* = 7.5, 1.1 Hz, 1H, Ar-H), 4.37 (s, 1H, C(2)*H*), 3.18 (s, 3H, OMe), 0.74 (s, 9H, C(CH_3)$_3$), -0.28 (s, 3H, Si-CH_3), -0.38 (s, 3H, Si-CH_3). **^{13}C NMR** (101 MHz, Methanol-*d*$_4$) δ 170.87 (COOMe), 169.52 (C=O), 146.36 (ArC), 141.14 (ArC), 135.26 (ArC), 134.85 (ArC), 133.97 (ArC), 131.84 (ArC), 130.32 (ArC), 127.03 (ArC), 126.48 (ArC), 125.07 (ArC), 124.94 (ArC), 118.77 (ArC), 101.37 (C(17)), 60.41 (C(2)), 52.42 (OMe), 25.82 (Si-C(**C**H$_3$)$_3$, 18.55 (Si-**C**(CH_3)), -3.78 (Si-**C**H$_3$), -4.00 (Si-**C**H$_3$). ***m/z***= 408 [MH-] (ESI$^-$), 410 (MH+) (ESI$^+$), 432 [MNa+] (ESI$^+$) and 847 [2MNa+] (ESI$^+$). **HRMS** requires 410.1782, found 410.1774.

4-(1,3-Dioxoisoindolin-2-yl)butanenitrile 18

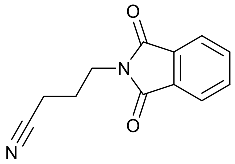

182

Potassium phthalimide (1.00 g, 5.40 mmol) and 4-bromobutyronitrile (0.75 mL, mmol) were dissolved in DMF (5 mL) and heated at 100 °C for 16 hours while stirring. The reaction was allowed to cool to room temperature then transferred to a separating funnel and diluted with DCM (15 mL). The organic layer was washed using warm water (3 x 15 mL) with the aqueous layer back extracted using DCM (20 mL). The combined organic layers were dried over Mg_2SO_4 then concentrated by reduced pressure. The crude organic was purified using flash column chromatography (a gradient from pet-ether 40:60 up to 50% ethyl acetate in pet-ether 40:60) with the product containing fractions being collected, combined and concentrated to afford the desired product as a white coloured solid (1.11 g, 97%). **R$_f$** = 0.5 (50% ethyl acetate in pet-ether 40:60). **ν$_{max}$ (KBr) cm^{-1}** 2247 (C≡N), 1772, 1705 (N-C=O), 1614, 1467 (C=C), 1436, 1396, 1376, 1358 (C-H), 1188, 1172, 1122, 1089, 1026. **^{1}H NMR** (400 MHz, Chloroform-*d*) δ 7.84 (dd, *J* = 5.5, 3.1 Hz, 2H, Ar-H), 7.73 (dd, *J* = 5.5, 3.1 Hz, 2H, Ar-H), 3.80 (t, *J* = 6.7 Hz, 2H, C(4)*H*), 2.42 (t, *J* = 7.3 Hz, 2H, (C(2)*H*), 2.06 (q, *J* = 14.0, 7.2,

6.9 Hz, 2H, C(3)*H*). **^{13}C NMR** (101 MHz, Chloroform-*d*) δ 168.30 (C(6,13)C=O), 134.32 (ArC), 134.30 (ArC), 131.96 (ArC), 123.54 (ArC), 118.83 (C(1)C≡N), 36.75 (C(4)), 24.87 (C(3)), 15.21 (C(2)). ***m/z***= 215 [MH+] (ESI$^+$).

(2*S*,13*R*)-13-((tert-Butyldimethylsilyl)oxy)-6-oxo-3,4,6,13-tetrahydro-1H-benzo[a]pyrrolizine-2-carbonitrile 19 and **(2*R*,13*R*)-13-((Tert-butyldimethylsilyl)oxy)-6-oxo-3,4,6,13-tetrahydro-benzo[a]pyrrolizine-2-carbonitrile 19'**

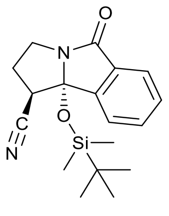

183a

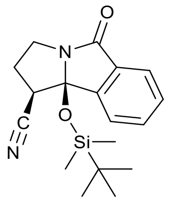

183b

Compound **18** (0.100 g, 0.46 mmol) was dissolved in anhydrous DCM (5 mL) followed by the addition of piperidine (0.13 mL, 1.38 mmol) and left stirring at room temperature for 30 minutes. The reaction was cooled to 0 °C with TBDMSiOTf (0.12 mL, 0.50 mmol) added dropwise and the resulting reaction left stirring at room temperature for 16 hours. The mixture was diluted with DCM (5 mL) then washed using water (2 x 10 mL) and back extracted with DCM (10 mL). The combined organic layers were combined, dried over $MgSO_4$ and concentrated by reduced pressure. The crude mixture was purified using flash column chromatography (30% ethyl acetate in pet-ether 40:60) to afford the product as a mixture of inseparable diastereomers (0.068 g, 44%). *d.r* = 3:1 for **19:19'**. **R_f**= 0.60. **v_{max} (KBr) cm^{-1}** 1773 (N-C=O), 1713 (N-C=O). ***m/z***= 329 [MH+] (ESI$^+$) and 351 [MNa+] (ESI$^+$). **HRMS** calculated for $C_{18}H_{25}N_2O_2Si$ requires 329.1680, found at 329.1680 [MH+] (ESI$^+$).

19 ^{1}H NMR (400 MHz, Chloroform-*d*) δ 7.80 – 7.75 (m, 1H, ArH), 7.66 – 7.61 (m, 1H, ArH), 7.59 – 7.53 (m, 2H, ArH), 4.00 – 3.87 (m, 1H, (C(4)H_A), 3.50 (ddd, *J* = 11.4, 8.9, 2.2 Hz, 1H, C(4)H_B), 3.38 (ddd, *J* = 6.8, 1.5, 0.6 Hz, 1H, (C(2)*H*), 2.89 (dddd, *J* = 13.2, 9.7, 8.9, 6.9 Hz, 1H, (C(3)H_A), 2.62 (dddd, *J* = 13.1, 7.9, 2.2, 1.5 Hz, 1H, C(3)H_B), 0.82 (s, 9H, C(CH_3)$_3$), -0.08 (s, 3H, Si-CH_3), -0.48 (s, 3H, Si-CH_3). **^{13}C NMR** (101 MHz, Chloroform-*d*) δ 170.07 (C=O), 144.20 (ArC), 133.30 (ArC), 132.31 (ArC), 130.99 (ArC), 124.08 (ArC), 121.67 (ArC), 117.31 (C-**C**≡N), 98.20 (C(13)), 41.15 (C(4)), 39.88 (C(2)), 32.57 (C(3)), 25.50 (C(**C**H$_3$)$_3$), 17.84 (**C**(CH_3)$_3$), -4.36 (Si-CH_3), -4.52 (Si-CH_3).

19' ^{1}H NMR (400 MHz, Chloroform-*d*) δ 7.84 (dd, *J* = 5.5, 3.0 Hz, 2H, ArH), 7.70 (dd, *J* = 5.5, 3.0 Hz, 2H, ArH), 4.00 – 3.87 (m, 1H, C(4)H_A), 3.84 – 3.75 (m, 1H, C(4)H_B), 2.03 – 1.84 (m, 3H, C(2)*H* and C(3)*H*), 0.96 (s, 9H, C(CH_3)$_3$), 0.17 (s, 3H, Si-CH_3), 0.09 (s, 3H, Si-CH_3). **^{13}C NMR** (101 MHz, Chloroform-*d*) δ 168.29 (C=O), 134.18 (ArC), 132.31 (ArC), 132.05 (ArC), 123.45 (ArC), 121.67 (C-**C**≡N), 98.20 (C(13)), 38.13, 26.80 (C(**C**H$_3$)$_3$), 26.36 (C(3)) 17.52 (**C**(CH_3)$_3$), 13.69 (C(2)), -7.03 (Si-CH_3), -7.14 (Si-CH_3).

Methyl 9-nitro-6-oxo-3,4-dihydro-1H-benzo[a]pyrrolizine-2-carboxylate 20a

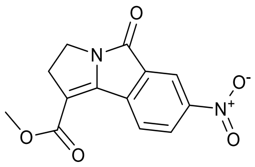

186a

20a was synthesised according to General Procedure **D** using **12a** (0.126 g, 0.31 mmol) to afford the desired product as a pale yellow coloured solid (0.109 g, quantitative yield). **Mp**= 206-208 °C. **ν_{max} (KBr) cm^{-1}** 1715 (N-C=O), 1695 (C=O), 1527 (-NO_2). **^{1}H NMR** (400 MHz, Chloroform-*d*) δ 8.74 (d, *J* = 8.4 Hz, 1H, Ar-H), 8.65 (d, *J* = 2.0 Hz, 1H, Ar-H), 8.51 (dd, *J* = 8.4, 2.1 Hz, 1H, Ar-H), 4.07 (t, *J* = 8.8 Hz, 2H, C(4)*H*), 3.93 (s, 3H, OMe), 3.45 (t, *J* = 8.8 Hz, 2H, C(3)*H*). **^{13}C NMR** (101 MHz, Chloroform-*d*) δ 164.33 (COOMe), 162.33 (C=O), 149.70 (Ar**C**-NO_2), 146.69 (C(13)C=**C**), 137.19 (ArC), 133.90 (ArC), 127.91 (C(11)ArC), 127.34 (ArC), 119.08 (ArC), 115.48 (C(2)**C**=C), 52.52 (OMe), 40.66 (C(4)), 34.51 (C(3)). ***m/z***= 275 [MH+] (ESI$^+$) and 297 [MNa+] (ESI$^+$). **HRMS** calculated for $C_{13}H_{11}N_2O_6$ requires 275.0662, found 275.0665 [MH+] (ESI$^+$).

Methyl 9-bromo-6-oxo-3,4-dihydro-1H-benzo[a]pyrrolizine-2-carboxylate 20b

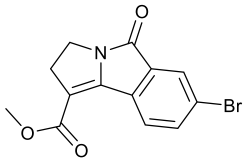

186c

20b was synthesised according to General Procedure D using **12c** (0.164 g, 0.40 mmol) to afford the desired product as a pale yellow coloured solid (0.143 g, quantitative yield). **Mp**= 197-199 °C. **ν_{max} (KBr) cm^{-1}** 1691 (C=O), 1647 (N-C=O). **^{1}H NMR** (400 MHz, Chloroform-*d*) δ 8.41 – 8.34 (m, 1H, Ar-H), 7.96 (dd, *J* = 1.9, 0.5 Hz, 1H, Ar-H), 7.77 (dd, *J* = 8.2, 1.8 Hz, 1H, Ar-H), 3.98 (t, *J* = 8.7 Hz, 2H, (C(4)*H*), 3.90 (s, 3H, OMe), 3.36 (t, *J* = 8.7 Hz, 2H, C(3)*H*). **^{13}C NMR** (101 MHz, Chloroform-*d*) δ 164.80 (COOMe), 163.15 (C=O), 147.92 (C(13)C=**C**), 137.56 (ArC), 135.50 (ArC), 128.16 (ArC), 128.02 (Ar**C**-Br), 126.95 (ArC), 126.30 (ArC), 112.43 (C(2)**C**=C), 52.20 OMe), 40.36 (C(4)), 34.27 (C(3)). ***m/z***= 309 and 311 [M+2H+] (ESI$^+$), 330 and 332 [MNa+] (ESI$^+$). **HRMS** calculated for $C_{13}H_{11}BrNO_3$ requires 307.9917 and 309.9896, found 307.9918 and 309.9897 [MH+] (ESI$^+$).

Methyl 9-fluoro-6-oxo-3,4-dihydro-1H-benzo[a]pyrrolizine-2-carboxylate 20c

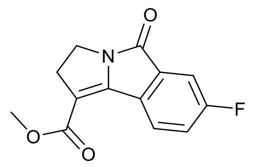

186e

20c was synthesised according to General Procedure D using **12c** (0.476 g, 1.25 mmol) to afford the desired product as a pale yellow coloured solid (0.317 g, quantitative yield). **Mp**= 201-203 °C. **ν_{max} (KBr) cm^{-1}** 1779, 1697 (C=O), 1646 (N-C=O). **^{1}H NMR** (400 MHz, Chloroform-*d*) δ 8.52 (dd, *J* = 8.5, 4.7 Hz, 1H, ArH), 7.50 (dd, *J* = 7.5, 2.4 Hz, 1H ArH), 7.33 (td, *J* = 8.7, 2.5 Hz, 1H, ArH), 3.98 (t, *J* = 8.7 Hz, 2H, C(4)*H*), 3.89 (s, 3H, OMe), 3.37 (t, *J* = 8.6 Hz, 2H, C(3)*H*). **^{13}C NMR** (101 MHz, Chloroform-*d*) δ 165.95 (COOMe), 164.83 (C=O), 158.59 (d, *J* = 41.8 Hz, Ar**C**-F), 147.70 (C(13)C=**C**), 138.41 (d, *J* = 8.9 Hz, ArC), 129.01 (d, *J* = 9.2 Hz, C(11)ArC), 125.42 (d, *J* = 2.7 Hz, ArC), 119.76 (d, *J* = 23.5 Hz, ArC), 111.75 (C(2)**C**=C), 111.05 (d, *J* = 24.4 Hz, ArC), 52.18 (OMe), 40.36 (C(4)), 34.13 (C(3)). ***m/z***= 248 [MH+] (ESI$^+$) and 270 [MNa+] (ESI$^+$). **HRMS** calculated for $C_{13}H_{11}FNO_3$ requires 248.0717, found 248.0719 [MH+] (ESI$^+$).

Methyl 10-nitro-6-oxo-3,4-dihydro-1H-benzo[a]pyrrolizine-2-carboxylate 21a

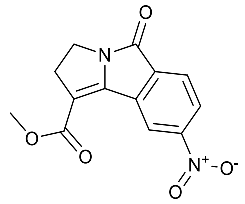

186b

21b was synthesised according to General Procedure **D** using **12b** (0.119 g, 0.29 mmol) with the ^{1}H NMR spectrum showed impurities from previous reaction so the crude material was purified using flash column chromatography (10% ethyl acetate in DCM) to afford the desired product as a yellow coloured solid (0.074 g, 92%). **R_f**= 0.48. **Mp**= 209-211 °C. **ν_{max} (KBr) cm^{-1}** 1715 (N-C=O), 1695 (N-C=O), 1648 (C=O), 1595, 1527 (-NO_2). **^{1}H NMR** (400 MHz, Chloroform-*d*) δ 9.33 (dd, *J* = 2.1, 0.6 Hz, 1H, Ar-H), 8.47 – 8.43 (m, 1H, Ar-H), 7.97 (dd, *J* = 8.3, 0.6 Hz, 1H, Ar-H), 4.03 (t, *J* = 7.9 Hz, 2H, C(4)*H*), 3.93 (s, 3H, OMe), 3.42 (t, *J* = 8.7 Hz, 2H, C(3)*H*). **^{13}C NMR** (101 MHz, Chloroform-*d*) δ 164.38 (COOMe), 161.45 (C=O), 150.49 (Ar**C**-NO_2), 146.85 (C(13)C=**C**), 140.81 (ArC), 130.35 (C(13)ArC), 126.53 (ArC), 124.36 (ArC), 122.10 (C(11)ArC), 113.05 (C(2)**C**=C), 52.34 (OMe), 40.50 (C(4)), 34.26 (C(3)). ***m/z***= 275 [MH+] (ESI$^+$) and 297 [MNa+] (ESI$^+$). **HRMS** calculated for $C_{13}H_{11}N_2O_6$ requires 275.0662, found 275.0664 [MH+] (ESI$^+$).

Methyl 10-fluoro-6-oxo-3,4-dihydro-1H-benzo[a]pyrrolizine-2-carboxylate 21c

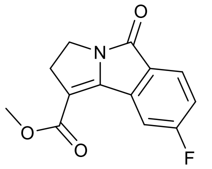

186f

21c was synthesised according to General Procedure D using **13c** (0.165 g, 0.435 mmol) to afford the desired product as a pale yellow coloured solid (0.110 g, quantitative yield). **Mp**= 179-181 °C. **ν$_{max}$ (KBr) cm^{-1}** 1712 (C=O), 1695 (N-C=O), 1649 (C=O). **^{1}H NMR** (400 MHz, Chloroform-*d*) δ 8.21 (dd, *J* = 8.7, 2.4 Hz, 1H, ArH), 7.79 (dd, *J* = 8.4, 4.9 Hz, 1H, ArH), 7.26 (m, 1H, ArH), 3.94 (t, *J* = 9.0 Hz, 2H, C(4)*H*), 3.88 (s, 3H, OMe), 3.34 (t, *J* = 9.1 Hz, 2H, C(3)*H*). **^{13}C NMR** (101 MHz, Chloroform-*d*) δ 167.57 (d, *J* = 41.7 Hz, (Ar**C**-F), 164.81 (COOMe), 162.95 (C=O), 148.14 (d, *J* = 3.2 Hz, C(13)C=**C**), 132.21 (d, *J* = 2.5 Hz, ArC), 131.64 (d, *J* = 11.2 Hz, ArC), 125.37 (d, *J* = 9.6 Hz, ArC), 118.79 (d, *J* = 23.8 Hz, C(11)ArC), 114.20 (d, *J* = 26.3 Hz, ArC), 111.10 (C(2)**C**=C), 52.00 (OMe), 40.24 (C(4)), 34.04 (C(3)). ***m/z***= 248 [MH+] (ESI^{+}) and 270 [MNa+] (ESI^{+}). **HRMS** calculated for $C_{13}H_{11}FNO_3$ requires 248.0717, found 248.0719 [MH+] (ESI^{+}).

Dimethyl 6-oxo-3,4-dihydro-1H-benzo[a]pyrrolizine-2,9-dicarboxylate 20d and **Dimethyl 6-oxo-3,4-dihydro-1H-benzo[a]pyrrolizine-2,10-dicarboxylate 21d**

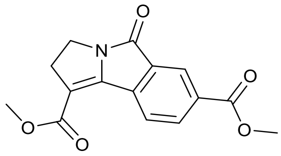

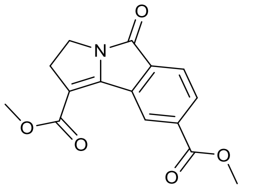

186g **186h**

20d and **21d** were synthesised according to General Procedure D using a mixture of **12d** and **13d** (0.130 g, 0.31 mmol) to afford the inseparable mixture of the desired products as an inseparable pale yellow coloured solid (0.090 g, quantitative yield). **ν$_{max}$ (KBr) cm^{-1}** 1694 (C=O), 1649 (N-C=O), 1618 (C=O). **^{1}H NMR** (400 MHz, Chloroform-*d*) δ 9.03 (d, *J* = 1.4 Hz, 1H, Ar-H), 8.51 (d, *J* = 8.1 Hz, 1H, Ar-H), 8.40 (d, *J* = 1.5 Hz, 1H, Ar-H), 8.25 (ddd, *J* = 10.7, 8.0, 1.5 Hz, 2H, Ar-H), 7.83 (d, *J* = 7.9 Hz, 1H, Ar-H), 3.95 (m, 10H, C(4)*H* and (OMe), 3.89 (m, 6H, C(9,10)OMe), 3.36 (t, *J* = 8.2 Hz, 4H, C(3)*H*). **^{13}C NMR** (101 MHz, Chloroform-*d*) δ 166.14 (C(O)OMe), 165.86 (C(O)OMe), 164.57 (C=O), 164.55 (C=O), 163.39 (OMe), 163.22 (OMe), 147.76 (C(13)C=**C**), 147.61 (C(13)C=**C**), 139.32 (ArC), 136.14 (ArC), 133.81 (ArC), 133.47 (ArC), 132.95 (ArC), 132.80 (ArC), 132.72 (ArC), 129.41 (ArC), 127.83 (ArC), 126.71 (ArC), 124.67 (ArC), 123.48 (ArC), 113.21 (C(2)**C**=C), 112.56 (C(2)**C**=C), 52.84 (OMe, 52.76 (OMe), 52.18 (OMe) 52.17 (OMe), 40.30 (C(4)), 40.28, (C(4)) 34.29 (C(3)), 34.24 (C(3)). ***m/z***= 576 [2MNa+] (ESI^{+}). **HRMS** calculated for $C_{15}H_{14}NO_5$ requires 288.0866, found 288.0866 [MH+] (ESI^{+}).

Methyl 9-methyl-6-oxo-3,4,6-benzo[a]pyrrolizine-2-carboxylate 20e and **Methyl 10-methyl-6-oxo-3,4,6-benzo[a]pyrrolizine-2-carboxylate 21e**

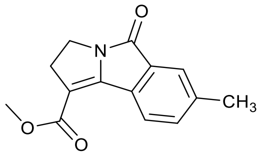

186i

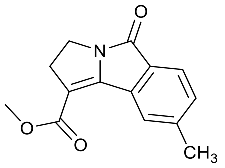

186j

20e and 21e were synthesised according to General Procedure D using a mixture of **12e** and **13e** (0.596 g, 1.58 mmol) with the crude material purified using flash column chromatography (20% ethyl acetate in DCM) to afford the desired products as in inseparable mixture of isomers as a yellow coloured solid (0.330 g, 85%). Note that the purification was to remove the starting material pre-cyclisation. $\mathbf{R_f}$= 0.54. **Mp**= 149-151 °C. $\boldsymbol{\nu_{max}}$ **(KBr) cm^{-1}** 1687 (C=O), 1643 (N-C=O). ***m/z***= 244 [MH+] (ESI$^+$) and 398 [MNa+] (ESI$^+$). **HRMS** calculated for $C_{14}H_{14}NO_3$ requires 244.0968, found 244.0968 [MH+] (ESI$^+$).

20e ^{1}H NMR (400 MHz, Chloroform-*d*) δ 8.34 (d, *J* = 7.9 Hz, 1H, ArH), 7.61 (dt, *J* = 1.6, 0.8 Hz, 1H, ArH), 7.41 (ddd, *J* = 7.9, 1.7, 0.8 Hz, 1H, ArH), 3.92 (t, *J* = 8.9 Hz, 2H, C(4)*H*), 3.87 (s, 3H, OMe), 3.32 (m, 2H), 2.49 (s, 3H, C(9a)ArC-CH_3). **^{13}C NMR** (101 MHz, Chloroform-*d*) δ 165.18 (COOMe), 164.05 (C=O), 149.33(C(12a)C=**C**), 142.39 (C(9/10)Ar**C**-CH_3), 136.55 (ArC), 132.93 (ArC), 127.04 (ArC, 126.47 (ArC), 123.88 (ArC), 109.12 (C(2)C=C), 51.79 (OMe, 40.01 (C(4)), 33.98 (C(3)), 22.01 C(9/10)Ar-**C**H_3).

21e ^{1}H NMR (400 MHz, Chloroform-*d*) δ 8.32 (dt, *J* = 1.6, 0.8 Hz, 1H, ArH), 7.71 – 7.66 (m, 1H ArH), 7.38 (ddd, *J* = 7.7, 1.5, 0.7 Hz, 1H, ArH), 3.92 (t, *J* = 8.9 Hz, 2H, C(4)*H*), 3.87 (s, 3H (OMe)), 3.32 (m, 2H), 2.47 (s, 3H, C(10a)ArC-CH_3). **^{13}C NMR** (101 MHz, Chloroform-*d*) δ 165.18 (COOMe), 164.05 (C=O), 149.65 (C(12a)C=**C**), 143.00 (C(9/10)Ar**C**-CH_3), 133.72 (ArC), 132.31 (ArC), 129.26 (ArC) 127.22 (ArC), 123.24 (ArC), 109.58 (**C**=C), 51.83 (OMe), 40.01 (C(4)), 34.01 (C(3)), 22.21 (C(9/10)Ar-**C**H_3).

1,4a-Dimethyl (4*S*)-6-oxo-3,4-dihydro-1H-benzo[a]pyrrolizine-2,4-dicarboxylate 22a

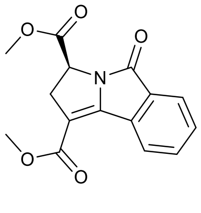

187L

Method 1) **22a** was synthesised according to General Procedure C using **14c** (0.095 g, 0.31 mmol) using 3.0 eq TBDMSiOTf with the crude oil purified using flash column chromatography (45% ethyl acetate in pet-ether 40:60) to afford the desired product as a yellow coloured solid (0.040 g, 45%).

Method 2) **22a** was synthesised according to General Procedure D from **16a** with the crude mixture purified using flash column chromatography (30% ethyl acetate in DCM) to afford the desired product as a yellow coloured solid (0.127 g, 61%).

$[\alpha]_D^{25}$= -12.9 (*c* 1.0 in DCM). **R**$_f$= 0.39 (30% ethyl acetate in DCM). **Mp**= 122-124 °C. **ν$_{max}$ (KBr) cm^{-1}** 1751 (C=O), 1693 (C=O), 1649 (N-C=O). **^{1}H NMR** (400 MHz, Chloroform-*d*) δ 8.54 – 8.47 (m, 1H, Ar-H), 7.85 (ddd, *J* = 7.3, 1.4, 0.7 Hz, 1H, Ar-H), 7.69 – 7.57 (m, 2H, Ar-H), 4.88 (dd, *J* = 11.0, 4.3 Hz, 1H, C(4)*H*), 3.86 (s, 3H, OMe), 3.79 (s, 3H, C(4a)OMe), 3.72 (m, 1H, C(3)H_A), 3.37 (dd, *J* = 17.6, 4.3 Hz, 1H, C(3)H_B). **^{13}C NMR** (101 MHz, Chloroform-*d*) δ 170.15 (COOMe), 164.45 (C(4a)COOMe), 163.44 (C=O), 148.96 (C(13)C=**C**), 135.79 (ArC, 132.63 (ArC), 131.80 (ArC), 129.59 (ArC, 126.94 (ArC), 123.93 (ArC), 107.46 C(2)**C**=C), 53.55 (OMe), 53.09 (C(4a)OMe), 51.96 (C(4)), 39.67 (C(3)). ***m/z***= 310 [MNa+] (ESI$^+$). **HRMS** calculated for $C_{15}H_{14}NO_5$ requires 288.0866, found 288.0867 [MH+] (ESI$^+$).

4-Benzyl 2-methyl (*S*)-6-oxo-3,4-dihydro-1H-benzo[a]pyrrolizine-2,4-dicarboxylate 22b

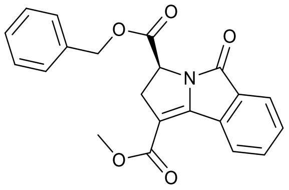

188

22b was synthesised according to General Procedure D using **16c** (0.150 g, 0.30 mmol) to afford the desired product as a yellow-coloured oil (0.110 g, quantitative yield). $[\alpha]_D^{25}$= -63.0 (*c* 1.0 in DCM). **ν$_{max}$ (KBr) cm^{-1}** 1697 (N-C=O), 1651 (C=O). **^{1}H NMR** (500 MHz, Chloroform-*d*) δ 8.52 (dt, *J* = 7.7, 1.0 Hz, 1H, ArH), 7.88 (dt, *J* = 7.3, 1.1 Hz, 1H, ArH), 7.74 – 7.60 (m, 3H, ArH), 7.38 – 7.32 (m, 4H, ArH), 5.23 (q, *J* = 12.2 Hz, 2H, C(4b)Ar-C**H**$_2$-O), 4.94 (dd, *J* = 11.0, 4.3 Hz, 1H, C(4)*H*), 3.87 (s, 3H, OMe), 3.77 – 3.69 (m, 1H, C(3)H_A), 3.36 (dd, *J* = 17.6, 4.3 Hz, 1H, C(3)H_B). **^{13}C NMR** (126 MHz, Chloroform-*d*) δ 168.77 (C(4a)COOMe), 164.46 (COOMe), 163.76 (C=O), 148.95 (C(13)C=**C**), 135.78 (ArC), 135.04 (ArC), 134.05 (ArC), 132.76 (ArC), 131.89 (ArC), 128.80 (ArC), 128.70 (ArC), 128.67 (ArC), 127.02 (ArC), 124.04 (ArC), 123.38 (ArC), 107.78 (C(2)**C**=C), 67.98 (C(4b)Ar-**C**H$_2$-O), 53.75 (OMe), 52.02 (C(4)), 39.64 (C(3)). ***m/z***= 368 [MNa+] (ESI$^+$). **HRMS** calculated for $C_{21}H_{18}NO_5$ requires 364.1179, found 364.1179 [MH+] (ESI$^+$).

6-Oxo-3,4H-dihydro-1H-benzo[a]pyrrolizine-2-carbonitrile 23

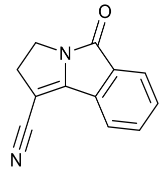

189

23 was synthesised according to General Procedure D from **19** (0.068 g, 0.20 mmol) to afford the desired product as pale yellow solid (0.059 g). **ν_{max} (KBr) cm^{-1}** 2221 (C≡N), 1773 (N-C=O), 1712 (N-C=O). **^{1}H NMR** (400 MHz, Chloroform-*d*) δ 7.98 – 7.93 (m, 1H, ArH), 7.86 – 7.81 (m, 1H, ArH), 7.71 (dd, *J* = 5.4, 3.1 Hz, 1H, ArH), 7.65 (ddd, *J* = 7.6, 5.5, 1.4 Hz, 1H, ArH), 4.02 (t, *J* = 8.9 Hz, 2H, C(4)*H*), 3.38 (t, *J* = 9.0 Hz, 2H, C(3)*H*). **^{13}C NMR** (101 MHz, Chloroform-*d*) δ 163.10 (C=O), 153.08 (C(13)C=**C**), 135.75 (ArC), 132.49 (ArC), 132.22 (ArC), 128.24 (ArC), 124.05 (ArC), 123.44 (ArC), 115.53 (C-**C**≡N), 85.29 (C(2)NC-**C**=C), 40.70 (C(4)), 35.22 (C(3)). **HRMS** calculated for $C_{12}C_9N_2O$ requires 197.0709, found 197.0715 [MH+] (ESI$^+$).

Methyl 1-(6,13-dioxoisoindolin-4-yl)-2-oxobutanoate 24

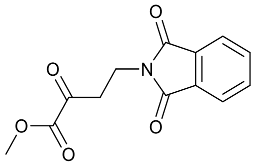

192

Compound **10a** (0.100 g, 0.43 mmol) was dissolved in DCM (3 mL) followed by the addition of *m*CPBA (0.09 g, 0.52 mmol) and left stirring at room temperature for 16 hours. The reaction mixture was diluted with DCM (5 mL) and washed using water (10 mL). The organic layer was concentrated by reduced pressure with the crude material purified using flash column chromatography (45% ethyl acetate in pet-ether) to afford the desired product as a colourless oil that solidified (0.052 g, 46%). **R_f**= 0.39. **^{1}H NMR** (400 MHz, Chloroform-*d*) δ 7.83 (dd, *J* = 5.4, 3.1 Hz, 2H, Ar-H), 7.72 (dd, 2H, Ar-H), 4.05 (t, *J* = 7.0 Hz, 2H, C(4)*H*), 3.88 (s, 3H, OMe), 3.25 (t, *J* = 6.9 Hz, 2H, (C(3)*H*). **^{13}C NMR** (101 MHz, Chloroform-*d*) δ 191.25 (C(2)C=O), 169.81 (COOMe), 168.14 (C=O), 134.28 (ArC), 132.05 (C(7,12)ArC), 123.55 (ArC), 53.31 (OMe), 38.31 (C(3)), 32.79 (C(4)). ***m/z***=284 [MNa+] (ESI$^+$). **HRMS** calculated for $C_{13}H_{12}NaNO_5$ requires 262.0710, found 262.0712 [MH+] (ESI$^+$).

Methyl (2*S*,13*S*)-6-oxo-3,4-dihydro-1H-12-oxa-1-azatricyclo-benzo[a]pyrrolizine-2-carboxylate 25

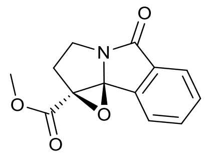

194

Compound **10a** (0.150 g, 0.65 mmol) was added to *m*CPBA (0.135 g, 0.78 mmol) and Ca_2CO_3 (0.083 g, 0.78 mmol) and dissolved in DCM (5 mL). The resulting mixture was then left stirring at room temperature for 16 hours. The reaction mixture was diluted with DCM (5 mL) and washed using water (2 x 10 mL) with the aqueous layers back extracted using DCM (10 mL). The organic layers were then combined and concentrated by reduced pressure to afford the desired product as a colourless solid (0.048 g, 30%). **Mp**= +240 °C. **ν_{max} (KBr) cm^{-1}** 2917, 1738 (C=O), 1700 (N-C=O). **^{1}H NMR** (400 MHz, Chloroform-*d*) δ 7.94 (d, *J* = 7.9 Hz, 1H, Ar-H), 7.81 (dd, *J* = 7.7, 1.3 Hz, 1H, Ar-H), 7.63 (td, *J* = 7.6, 1.3 Hz, 1H, Ar-H), 7.51 (td, *J* = 7.5, 0.8 Hz, 1H, Ar-H), 4.35 (dd, *J* = 12.5, 9.1 Hz, 1H, C(4)H_A), 3.93 (td, *J* = 12.0, 6.1 Hz, 1H, C(4)H_B), 3.46 (s, 3H, OMe), 2.55 (dd, *J* = 14.3, 6.0 Hz, 1H, C(3)H_A), 2.24 (ddd, *J* = 14.2, 11.7, 9.2 Hz, 1H, C(3)H_B). **^{13}C NMR** (101 MHz, Chloroform-*d*) δ 169.95 (COOMe), 169.46 (C=O), 140.81 (ArC), 134.19 (ArC), 131.81 (ArC), 129.75 (ArC), 127.59 (ArC), 124.50 (ArC), 65.83 (C(2)), 52.46 (COOMe), 42.11 (C(4)), 35.57 (C(3)). **HRMS** calculated for $C_{13}H_{12}NO_4$ requires 246.0764, found 246.0761 [MH+] (ESI$^+$).

Methyl (2*R*,13*R*)-13-(3-chlorobenzoylperoxy)-8-nitro-6-oxo-,3,4,6,13-tetrahydro-1H-benzo[a]pyrrolizine-2-carboxylate 26

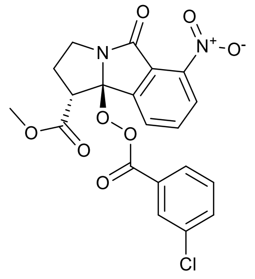

191

Compound **10b** (0.045 g, 0.16 mmol) was dissolved in DCM (10 mL) followed by the addition of *m*CPBA (0.034 g, 0.19 mmol) with the reaction mixture left stirring for 16 hours at room temperature. The crude mixture was washed using water (2 x 10 mL) and back extracted using DCM (10 mL) with the combined organic layers washed using brine (20 mL). The organic solvent was concentrated by reduced pressure with the crude material purified using flash column chromatography (40% ethyl acetate in pet-ether 60:60) to afford the product as a colourless oil (0.011 g, 15%). **R_f**= 0.45. **ν_{max} (KBr) cm^{-1}** 1776 (C=O), 1716 (N-C=O), 1541 (NO_2). **^{1}H NMR** (500 MHz, Chloroform-*d*) δ 8.06 (ddd, *J* = 15.2, 7.8, 0.9 Hz, 2H, Ar-H), 7.95 – 7.90 (m, 2H, Ar-H), 7.87 (t, *J* = 7.8 Hz, 1H, Ar-H), 7.52 (ddd, *J* = 8.0, 2.2, 1.1 Hz, 1H, PhtAr-H), 7.36 (t, *J* = 7.9 Hz, 1H, Phth-Ar-H), 5.32 (t, *J* = 6.8 Hz,

1H, C(2)*H*), 3.98 (td, *J* = 6.7, 1.9 Hz, 2H, C(4)*H*), 3.75 (s, 3H, OMe), 2.44 (q, *J* = 6.7 Hz, 2H, C(3)*H*). **^{13}C NMR** (126 MHz, Chloroform-*d*) δ 169.56 (COOMe), 165.66 (C=O), 164.60 (C(13)), 162.80 (C=O), 145.16 (ArC), 135.62 (ArC), 134.70 (ArC), 134.06 (Ar**C**-NO_2), 133.61 (ArC), 130.87 (ArC), 129.95 (ArC), 129.86 (ArC), 128.81 (Phth Ar-C), 128.15 (Phth Ar-C), 127.22 (Phth Ar-C), 123.78 (Ar**C**-Cl), 70.98 (C(2)), 52.87 (OMe), 35.30 (C(4)), 29.59 (C(3)). ***m/z***= 916 [2MH+] (ESI^+). **HRMS** calculated for $C_{20}H_{16}ClN_2O_8$ requires 447.0589 and 449.0560, found 447.0589 and 449.0559 [MH+] (ESI^+).

Methyl (1*R*,13*S*)-6-oxo-3,4,6,13-tetrahydro-1H-benzo[a]pyrrolizine-2-carboxylate 27a

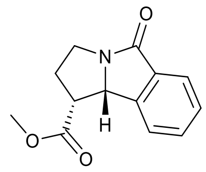

196a

27a was synthesised according to General Procedure E using **10a** (2.36 g, 10.29 mmol) to afford the desired product as a colourless solid (2.62 g, quantitative yield). **Mp**= 116-118 °C. **ν_{max} (KBr) cm^{-1}** 1729 (C=O), 1686 (N-C=O), 1615, 1468 (C=C). **^{1}H NMR** (500 MHz, Chloroform-*d*) δ 7.75 (dt, *J* = 7.5, 1.1 Hz, 1H, ArH), 7.50 (td, *J* = 7.4, 1.2 Hz, 1H, ArH), 7.47 – 7.38 (m, 2H, ArH), 4.97 (d, *J* = 6.9 Hz, 1H, C(12a)*H*), 3.98 (dt, *J* = 11.2, 8.5 Hz, 1H, C(4)H_A), 3.44 (dtd, *J*=17.4, 9.0, 2.5 Hz, 1H, (C(4)H_B), 3.34 (td, *J* = 7.0, 1.6 Hz, 1H, C(2)*H*), 3.14 (s, 3H, OMe), 2.60 (dddd, *J* = 13.2, 8.4, 3.0, 1.6 Hz, 1H, C(3)H_A), 2.49 (dddd, *J* = 13.5, 9.4, 8.6, 7.0 Hz, 1H, C(3)H_B). **^{13}C NMR** (126 MHz, Chloroform-*d*) δ 171.63 (COOMe), 171.60 (C=O), 142.57 (ArC), 134.51 (ArC), 131.53 (ArC), 128.96 (ArC), 123.87 (ArC), 123.55 (ArC), 66.31 (C(13)), 51.45 (OMe), 44.56 (C(4)), 41.79 (C(2)), 32.35 (C(3)). ***m/z***= 232 [MH+] (ESI^+) and 254 [MNa+] (ESI^+). **HRMS** calculated for $C_{13}H_{14}NO_3$ requires 232.0968, found 232.0966 [MH+] (ESI^+).

Methyl (2*R*,13*S*)-8-amino-6-oxo-3,4,6,13-tetrahydro-1H-benzo[a]pyrrolizine-2-carboxylate 27b

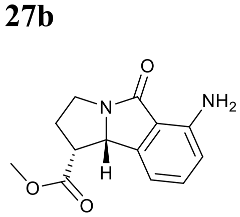

196b

27b was synthesised according to General Procedure E using **10b** (0.100 g, 0.36 mmol) to afford the desired product as a pale yellow coloured solid (0.097 g, quantitative yield). **Mp**= 153-155 °C. **ν_{max} (KBr) cm^{-1}** 3464 (N-H), 3362 (N-H), 1730 (C=O), 1672 (N-C=O). **^{1}H NMR** (500 MHz, Chloroform-

d) δ 7.21 (dd, *J* = 8.1, 7.4 Hz, 1H, Ar-H), 6.66 (dd, *J* = 7.4, 0.9 Hz, 1H, Ar-H), 6.55 (dd, *J* = 8.2, 0.8 Hz, 1H, Ar-H), 4.90 (d, *J* = 7.2 Hz, 1H, C(12a)*H*), 3.95 (dd, *J* = 11.2, 8.4 Hz, 1H, C(4)H_A), 3.37 (ddd, *J* = 11.2, 9.5, 3.2 Hz, 1H, C(4)H_B), 3.29 (dt, *J* = 7.0, 1.9 Hz, 1H, C(2)*H*), 3.25 (s, 3H, OMe), 2.55 (dddd, *J* = 13.5, 8.4, 3.2, 1.9 Hz, 1H, C(3)H_B), 2.44 (dddd, *J* = 13.5, 9.3, 8.4, 7.0 Hz, 1H, C(3)H_B). **^{13}C NMR** (126 MHz, Chloroform-*d*) δ 176.12 (C=O), 173.87 (COOMe), 145.93 (Ar**C**-NH_2), 143.84 (ArC), 133.04 (ArC), 116.60 (ArC), 114.54 (ArC), 111.97 (ArC), 66.33 (C(13)), 51.53 (OMe), 44.68 (C(2)), 41.79 (C(4)), 32.21 (C(3)). ***m/z***= 247 [MH+] (ESI$^+$). **HRMS** calculated for $C_{13}H_{15}N_2O_3$ requires 247.1077, found 247.1075 [MH+] (ESI$^+$).

Methyl (2*R*,13*S*)-9-amino-6-oxo-3,4,6,13-tetrahydro-1H-benzo[a]pyrrolizine-2-carboxylate 27c

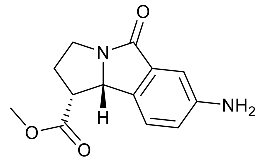

196c

27c was synthesised according to General Procedure E using **20a** (0.109 g, 0.39 mmol) to afford the desired product as a pale yellow coloured solid (0.104 g, quantitative yield). **ν_{max} (KBr) cm^{-1}** 3344 (N-H), 3025, 2948, 1729 (C=O), 1676 (N-C=O). **^{1}H NMR** (400 MHz, Chloroform-*d*) δ 7.17 (dd, *J* = 8.1, 2.7 Hz, 1H, ArH), 6.94 (d, *J* = 2.3 Hz, 1H, ArH), 6.74 (dd, *J* = 8.2, 2.3 Hz, 1H, ArH), 4.87 (d, *J* = 6.9 Hz, 1H, C(13)*H*), 3.94 (dtd, *J* = 11.4, 8.5, 2.7 Hz, 1H, C(4)H_A), 3.39 (ddd, *J* = 11.6, 9.3, 2.9 Hz, 1H, C(4)H_B), 3.25 (td, *J* = 6.9, 1.5 Hz, 1H, C(2)*H*), 3.20 (s, 3H, OMe), 2.56 (dddd, *J* = 13.1, 8.5, 3.0, 1.6 Hz, 1H, C(3)H_A), 2.50 – 2.38 (m, 1H, (C(3)H_B). **^{13}C NMR** (101 MHz, Chloroform-*d*) δ 172.25 (COOMe), 171.94 (C=O), 149.00 (Ar**C**-NH_2), 135.63 (ArC), 131.15 (ArC), 124.05 (ArC), 117.43 (ArC), 106.12 (ArC), 66.03 (C(13)), 51.48 (OMe), 44.68 (C(2)), 41.69 (C(4)), 32.21 (C(3)). ***m/z***=247 [MH+] (ESI$^+$). **HRMS** calculated for $C_{13}H_{15}N_2O_3$ requires 247.1077, found 247.1075 [MH+] (ESI$^+$).

Methyl (2*R*,13*S*)-10-amino-6-oxo-3,4,6,13-tetrahydro-1H-benzo[a]pyrrolizine-2-carboxylate 27d

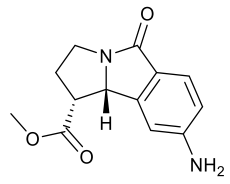

196d

27d was synthesised according to General Procedure E using **21a** (0.045 g, 0.16 mmol) to afford the desired product as a pale yellow coloured solid (0.062 g, quantitative yield). **ν_{max} (KBr) cm^{-1}** 3346 (N-H), 1728 (C=O), 1670 (N-C=O), 1609 (C=O). **^{1}H NMR** (400 MHz, Chloroform-*d*) δ 7.52 (d, *J* =

8.2 Hz, 1H, ArH), 6.69 – 6.62 (m, 2H, ArH), 4.85 (d, *J* = 7.0 Hz, 1H, C(13)*H*), 3.94 (dt, *J* = 11.0, 8.4 Hz, 1H(C(4)H_A), 3.38 (ddd, *J* = 11.6, 9.3, 3.0 Hz, 1H (C(4)H_B), 3.30 – 3.23 (m, 4H, C(2)*H* and OMe), 2.55 (ddt, *J* = 13.2, 8.3, 2.6 Hz, 1H, C(3)H_A), 2.44 (dtd, *J* = 13.5, 8.9, 7.1 Hz, 1H, C(3)H_B). **^{13}C NMR** (101 MHz, Chloroform-*d*) δ 172.44 (COOMe), 171.84 (C=O), 150.23 (Ar**C**-NH_2), 145.18 (ArC), 125.25 (ArC), 124.39 (ArC), 115.49 (ArC), 108.62 (ArC), 65.97 (C(4)), 51.56 (OMe), 44.86 (C(2)), 41.96 (C(4)), 32.27 (C(3)). ***m/z***= 247 [MH+] (ESI$^+$) and 269 [MH+] (ESI$^+$). **HRMS** calculated for $C_{13}H_{15}N_2O_3$ requires 247.1077, found 247.1075 [MH+] (ESI$^+$).

Methyl (2*R*,13*S*)-8-fluoro-6-oxo-3,4,6,13-tetrahydro-1H-benzo[a]pyrrolizine-2-carboxylate 27e and **Methyl (2*R*,13*S*)-11-fluoro-6-oxo-3,4,6,13-tetrahydro-1H-benzo[a]pyrrolizine-2-carboxylate 27f**

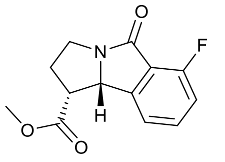

196e

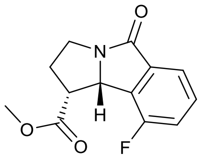

196f

27e and **27f** were synthesised according to General Procedure E using a mixture of **10c** and **10c'** (0.250 g, 1.01 mmol) with the products separated by flash column chromatography (25% ethyl acetate in pet-ether 40:60) to afford the desired products as yellow-coloured solids.

27e (0.125 g, 50%). **R$_f$**= 0.16. **Mp**= 143-145 °C. **ν$_{max}$ (KBr) cm^{-1}** 1730 (C=O), 1694 (N-C=O), 1626 (C=O). **^{1}H NMR** (500 MHz, Chloroform-*d*) δ 7.48 (ddd, *J* = 8.4, 7.6, 4.7 Hz, 1H, ArH), 7.20 (d, *J* = 7.5 Hz, 1H, ArH), 7.07 (t, *J* = 17.6, 8.8 Hz, 1H, ArH), 4.97 (d, *J* = 6.9 Hz, 1H, C(13)*H*), 3.98 (dt, *J* = 11.3, 8.5 Hz, 1H, (C(4)H_A), 3.41 (ddd, *J* = 11.9, 9.3, 3.0 Hz, 1H, C(4)H_B), 3.34 (td, *J* = 7.0, 1.6 Hz, 1H, C(2)*H*), 3.23 (s, 3H, OMe), 2.59 (dddd, *J* = 13.2, 8.5, 3.0, 1.6 Hz, 1H, C(3)H_A), 2.54 – 2.45 (m, 1H, C(3)H_B). **^{13}C NMR** (126 MHz, Chloroform-*d*) δ 171.50 (COOMe), 168.40 (d, *J* = 2.3 Hz, C=O), 158.50 (d, *J* = 260.7 Hz, Ar**C**-F), 145.30 (d, *J* = 3.2 Hz, ArC), 133.64 (d, *J* = 7.5 Hz, ArC), 121.65 (d, *J* = 13.5 Hz, ArC), 119.60 (d, *J* = 4.1 Hz, C(11)ArC), 116.37 (d, *J* = 19.5 Hz, ArC) , 66.00 (C(13)), 51.66 (OMe), 44.59 (C(2)), 41.98 (C(4)), 32.37 (C(3)). ***m/z***= 248 [MH-] (ESI$^-$). **HRMS** calculated for $C_{13}H_{13}FNO_3$ requires 250.0874, found 250.0875 [MH+] (ESI$^+$).

27f (0.035 g, 14%). **R$_f$**= 0.20. **Mp**= 144-146 °C. **ν$_{max}$ (KBr) cm^{-1}** 1731 (C=O), 1698 (N-C=O). **^{1}H NMR** (500 MHz, Chloroform-*d*) δ 7.56 (dt, *J* = 7.5, 0.8 Hz, 1H, ArH), 7.45 (dddd, *J* = 8.1, 7.4, 4.6, 0.6 Hz, 1H, ArH), 7.20 (ddd, *J* = 8.8, 8.2, 0.8 Hz, 1H, ArH), 5.04 (d, *J* = 6.4 Hz, 1H, C(13)*H*), 4.00 – 3.91 (m, 1H, C(4)H_A), 3.48 – 3.44 (m, 1H, C(4)H_B), 3.44 – 3.40 (m, 1H, (C(2)*H*), 3.20 (s, 3H, OMe), 2.64 (dddd, *J* = 13.6, 8.4, 2.4, 1.1 Hz, 1H, (C(3)H_A), 2.53 (dtd, *J* = 13.6, 9.3, 6.8 Hz, 1H, C(3)H_B). **^{13}C NMR** (126 MHz, Chloroform-*d*) δ 171.30 (COOMe), 170.27 (d, *J* = 2.4 Hz, C=O),

157.84 (d, J = 251.5 Hz, C(11)Ar**C**-F), 137.43 (d, J =4.4 Hz, ArC), 131.40 (d, J = 6.5 Hz, ArC), 128.68 (d, J = 18.3 Hz, ArC), 119.90 (d, J = 3.6 Hz, ArC), 118.42 (d, J = 19.6 Hz, ArC), 63.87 (C(13)), 51.68 (OMe), 44.09 (C(2)), 41.65 (C(4)), 32.39 (C(3)). ***m/z***= 248 [MH-] (ESI^-). **HRMS** calculated for $C_{13}H_{13}FNO_3$ requires 250.0874, found 250.0874 [MH+] (ESI^+).

Methyl (2*R*,13*S*)-9-fluoro-6-oxo-3,4,6,13-tetrahydro-1H-benzo[a]pyrrolizine-2-carboxylate 27g

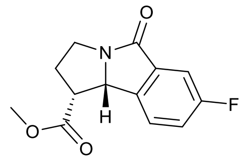

196g

27g was synthesised according to General Procedure E using **20c** (0.317 g, 1.28 mmol) to afford the desired product as a colourless solid (0.331 g, quantitative yield). **Mp**= 139-141 °C. **ν_{max} (KBr) cm^{-1}** 1729 (C=O), 1690 (N-C=O). **^{1}H NMR** (400 MHz, Chloroform-*d*) δ 7.45 – 7.36 (m, 2H, ArH), 7.20 (ddd, J = 9.0, 8.3, 2.5 Hz, 1H, ArH), 4.95 (d, J = 6.9 Hz, 1H, C(13)*H*), 3.97 (dt, J = 11.4, 8.5 Hz, 1H, C(4)H_A), 3.44 (dddd, J = 11.3, 9.4, 2.9, 0.7 Hz, 1H, C(4)H_B), 3.33 (td, J = 6.9, 1.6 Hz, 1H, C(2)*H*), 3.19 (s, 3H, (OMe), 2.61 (dddd, J = 13.0, 8.4, 2.9, 1.5 Hz, 1H, C(3)H_A), 2.50 (dddd, J = 13.6, 9.4, 8.6, 6.9 Hz, 1H, C(3)H_B). **^{13}C NMR** (101 MHz, Chloroform-*d*) δ 171.47, 170.28, 163.34 (d, J = 248.5 Hz, Ar**C**-F), 138.00 (d, J = 3.0 Hz, ArC), 136.81 (d, J = 8.0 Hz, ArC), 125.09 (d, J = 8.6 Hz, C(11)ArC), 119.00 (d, J = 23.8 Hz, ArC), 110.74 (d, J = 23.5 Hz, ArC), 65.90 (C(13)), 51.58 (OMe), 44.49 (C(2)), 41.89 (C(4)), 32.36 (C(3)). ***m/z***= 248 [MH-] (ESI^-). **HRMS** calculated for $C_{13}H_{13}FNO_3$ requires 250.0874, found 250.0872 [MH+] (ESI^+).

Methyl (2*R*,13*S*)-8-hydroxy-6-oxo-3,4,6,13-tetrahydro-1H-benzo[a]pyrrolizine-2-carboxylate 27h

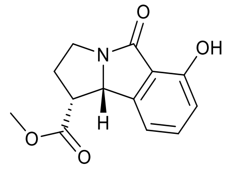

196i

27h was synthesised according to General Procedure E using **10d** (0.400 g, 1.19 mmol) to afford the desired product as a colourlesssolid (0.252 g, 86%). **ν_{max} (KBr) cm^{-1}** 1731 (C=O), 1672 (N-C=O). **^{1}H NMR** (400 MHz, Chloroform-*d*) δ 7.37 (dd, J = 8.3, 7.4 Hz, 1H, ArH), 6.91 (dt, J = 7.4, 0.8 Hz, 1H, ArH), 6.85 (d, J = 8.2 Hz, 1H, ArH), 4.98 (d, J = 6.9 Hz, 1H, C(13)*H*), 3.93 (dt, J = 11.1, 8.5 Hz, 1H, C(4)H_A), 3.40 (dtd, J= 17.9, 8.9, 2.9 Hz, 1H, C(4)H_B), 3.32 (td, J = 6.9, 1.6 Hz, 1H, C(2)*H*), 3.23 (s, 3H, OMe), 2.60 (dddd, J = 13.2, 8.4, 3.0, 1.6 Hz, 1H, C(3)H_A), 2.49 (dddd, J = 13.6, 9.4, 8.6, 6.9

Hz, 1H, C(3)H_B). **^{13}C NMR** (101 MHz, Chloroform-*d*) δ 173.27 (COOMe), 171.61 (C=O), 155.56 (Ar**C**-OH), 142.88 (ArC), 134.01 (ArC), 118.57 (ArC), 115.60 (C(11)ArC), 115.04 (ArC), 67.08 (C(13)), 51.59 (OMe), 44.23 (C(2)), 41.34 (C(4)), 32.46 (C(3)). ***m/z***= 246 [MH-] (ESI$^-$), 248 [MH+] (ESI$^+$) and 270 [MNa+] (ESI$^+$). **HRMS** calculated for $C_{13}H_{14}NO_4$ requires 248.0917, found 248.0916 [MH+] (ESI$^+$).

Methyl (2*R*,13S)-9-methyl-6-oxo-3,4,6,13-tetrahydro-1H-benzo[a]pyrrolizine-2-carboxylate 27i and Methyl (2*R*,13S)-10-methyl-6-oxo-3,4,6,13-tetrahydro-1H-benzo[a]pyrrolizine-2-carboxylate 27j

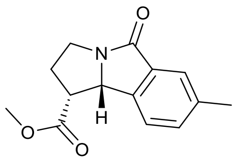

196j

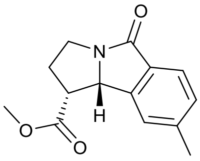

196k

27i and **27j** were synthesised according to General Procedure E using a mixture of **20e** and **21e** (0.300 g, 1.35 mmol) to afford the desired products as an inseparable colourless solid (0.318 g, 95%). **ν_{max} (KBr) cm^{-1}** 1769 (C=O), 1731 (C=O), 1683 (N-C=O). ***m/z***= 246 [MH+] (ESI$^+$) and 268 [MNa+] (ESI$^+$). **HRMS** calculated for $C_{14}H_{16}NO_3$ requires 246.1125, found at 246.1125 [MH+] (ESI$^+$).

27i ^{1}H NMR (400 MHz, Chloroform-*d*) δ 7.56 (q, *J* = 1.0 Hz, 1H, ArH), 7.30 (d, *J* = 1.2 Hz, 1H, ArH), 7.26 – 7.20 (m, 1H, ArH), 4.93 (dd, *J* = 6.9, 4.4 Hz, 1H, C(13)*H*), 4.01 – 3.91 (m, 1H, C(4)H_A), 3.45 – 3.38 (m, 1H, (C(4)H_B), 3.31 (td, *J* = 7.0, 1.6 Hz, 1H, (C(2)*H*), 3.17 (d, *J* = 2.0 Hz, 3H, (OMe), 2.58 (dddd, *J* = 13.2, 8.4, 3.0, 1.6 Hz, 1H, C(3)H_A), 2.53 – 2.44 (m, 1H, C(3)H_B), 2.39 (d, *J* = 0.7 Hz, 3H, ArC-C**H**$_3$). **^{13}C NMR** (101 MHz, Chloroform-*d*) δ 171.82 (C=O), 171.73 (COOMe), 142.27 (ArC), 139.08 (Ar**C**-CH$_3$), 134.62 (ArC), 132.54 (ArC), 124.15 (ArC), 123.23 (ArC), 66.17 (C(13)), 51.48 (OMe), 44.55 (C(2)), 41.77 (C(4)), 32.36 (C(3)), 21.49 (ArC-**C**H$_3$).

27j ^{1}H NMR (400 MHz, Chloroform-*d*) δ 7.66 – 7.61 (m, 1H, ArH), 7.30 (d, *J* = 1.2 Hz, 1H, ArH), 7.26 – 7.20 (m, 1H, ArH), 4.93 (dd, *J* = 6.9, 4.4 Hz, 1H, C(13)*H*), 4.01 – 3.91 (m, 1H, (C(4)H_A), 3.45 – 3.38 (m, 1H, C(C4)H_B), 3.31 (td, *J* = 7.0, 1.6 Hz, 1H, (C(2)*H*), 3.17 (d, *J* = 2.0 Hz, 3H, (OMe), 2.58 (dddd, *J* = 13.2, 8.4, 3.0, 1.6 Hz, 1H, C(3)H_A), 2.53 – 2.44 (m, 1H, C(3)H_B), 2.42 (s, 3H, ArC-C**H**$_3$). ^{13}C NMR (101 MHz, Chloroform-*d*) δ 171.88 (C=O), 171.67 (COOMe), 142.99 (ArC), 139.77 (Ar**C**-CH$_3$), 131.92 (ArC), 129.95 (ArC), 124.01 (ArC), 123.66 (ArC), 66.17 (C(13)), 51.46 (OMe), 44.63 (C(2)), 41.79 (C(4)), 32.36 (C(3)), 21.96 (ArC-**C**H$_3$).

Methyl (2*R*,13*R*)-8-(adamantane-1-amido)-13-[(tert-butyldimethylsilyl)oxy]-6-oxo-3,4,6,13-tetrahydro-1H-benzo[a]pyrrolizine-2-carboxylate 28

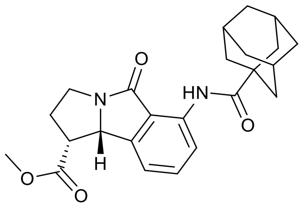

197

Compound **27b** (0.050 g, 0.20 mmol) and 1-adamantanecarbonyl chloride (0.048 g, 0.24 mmol) and dissolved in DCM (5 mL) followed by the addition of DIPEA (0.042 mL), 0.24 mmol) with the reaction mixture left stirring at room temperature for 16 hours. The crude mixture was transferred to a separating funnel and diluted with DCM (5 mL) and washed using water (2 x 10 mL) then back extracted using DCM (10 mL). The combined organic layers were combined and dried over $MgSO_4$ and concentrated by reduced pressure. The crude material was then purified using flash column chromatography (20-40% ethyl acetate in pet-ether 40:60) with the product containing fractions collected, combined and concentrated to afford the desired product as a pale-yellow coloured oil (0.023 g, 28%). **ν_{max} (KBr) cm^{-1}** 1734 (C=O), 1677 (N-C=O), 1621 (N-C=O). **^{1}H NMR** (400 MHz, Chloroform-*d*) δ 10.51 (s, 1H, N(8a)*H*), 8.53 (d, *J* = 8.3 Hz, 1H, Ar-H), 7.49 – 7.42 (t, *J*= 8.2 Hz, 1H, Ar-H), 7.06 (dt, *J* = 7.5, 0.8 Hz, 1H, Ar-H), 4.97 (d, *J* = 7.0 Hz, 1H, C(12a)*H*), 3.97 (dt, *J* = 11.1, 8.5 Hz, 1H, C(4)H_A), 3.44 (dddd, *J* = 11.1, 9.4, 2.8, 0.7 Hz, 1H, C(4)H_B), 3.35 (td, *J* = 6.8, 1.5 Hz, 1H, C(2)*H*), 3.24 (s, 3H, OMe), 2.63 (dddd, *J* = 13.6, 8.4, 2.8, 1.5 Hz, 1H, C(3)H_A), 2.57 – 2.45 (m, 1H, C(3)H_B), 2.02 (d, *J* = 3.1 Hz, 7H, C(Adamantane)*H*), 1.75 (t, *J* = 3.2 Hz, 7H, C(adamantane)*H*). **^{13}C NMR** (101 MHz, Chloroform-*d*) δ 177.70 (COOMe), 172.93 C(C6)C=O), 171.66 (C(8b)C=O), 142.54 ArC), 138.15 (Ar**C**-N), 133.46 (ArC), 119.91 (ArC), 118.86 (ArC), 117.59 (ArC), 66.45 (C(13)), 51.70 (OMe), 44.09 (C(2)), 42.16 (Adamantane C), 41.51 (C(4)), 39.16 (Adamantane C), 36.58 (Adamantane C), 32.40 (C(3)), 28.28 (Adamantane C). ***m/z***= 409 [MH+] (ESI$^+$). **HRMS** calculated for $C_{24}H_{29}N_2O_4$ requires 409.2121, found 409.2118 [MH+] (ESI$^+$).

Dimethyl (1*S*,4a*S*,13*R*)-6-oxo-3,4,6,13-tetrahydro-1H-benzo[a]pyrrolizine-2,4-dicarboxylate 29a

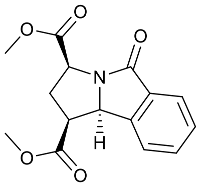

198L

29a was synthesised according to General Procedure E using **22a** using (0.104 g, 0.36 mmol) to afford the desired product as a yellow-coloured solid (0.063 g, 63%). **Mp**= 106-108 °C. **ν_{max} (KBr) cm^{-1}** 1738 (C=O), 1692 (N-C=O), 1616 (C=O). **^{1}H NMR** (500 MHz, Chloroform-*d*) δ 7.77 (d, *J* = 7.6, 1.1

Hz, 1H, Ar-H), 7.54 (td, *J* = 7.5, 1.3 Hz, 1H, Ar-H), 7.47 – 7.41 (m, 2H, Ar-H), 5.03 (d, *J* = 7.2 Hz, 1H, C(4)*H*), 4.40 (dd, *J* = 9.0, 3.2 Hz, 1H (C(13)*H*), 3.76 (s, 3H, OMe), 3.42 (td, *J* = 7.6, 2.2 Hz, 1H, C(2)*H*), 3.35 (s, 3H C(4a)OMe), 3.01 (ddd, *J* = 14.0, 3.2, 2.2 Hz, 1H, C(3)H_A), 2.83 (ddd, *J* = 13.9, 9.0, 7.8 Hz, 1H, C(3)H_B). **^{13}C NMR** (126 MHz, Chloroform-*d*) δ 170.40 (COOMe), 169.92 (C(4a)COOMe), 168.32 (C=O), 143.35 (ArC), 134.47 (ArC), 131.67 (ArC), 128.40 (ArC), 124.20 (ArC), 123.50 (ArC), 66.09 (C(4)), 55.11 (C(2)), 52.60 (OMe), 51.83 (C(4a)OMe), 43.21 (C(2)), 37.19 (C(3)). ***m/z***= 290 [MH+] (ESI$^+$) and 312 [MNa+] (ESI$^+$). **HRMS** calculated for $C_{15}H_{16}NO_5$ requires 290.1023, found 290.1021 [MH+] (ESI$^+$).

Quinolin-8-yl 1-(6,13-dioxoisoindolin-5-yl)butanoate 30

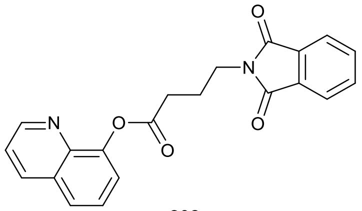

208

Compound **3a** (0.500 g, 2.14 mmol) was dissolved in DCM (5 mL) followed by the addition of DCC (0.486 g, 2.35 mmol) and a catalytic amount of DMAP and left stirring at room temperature for 5 minutes. 8-Hydroxyquinoline (0.920 g, 6.42 mmol) was added to the reaction mixture and left stirring at room temperature for 16 hours. The crude mixture was diluted with DCM (10 mL) then transferred to a separating funnel and washed using water (2 x 10 mL) and back extracted using DCM (20 mL) with the combined organic layers were combined, dried over $MgSO_4$ and concentrated by reduced pressure. The crude mix was purified using flash column chromatography (30-50% ethyl acetate in pet-ether 40:60) with the product containing fractions collected, combined and concentrated by reduced pressure to afford the desired product as a pale yellow coloured solid (0.498 g, 64%). **Mp**= 136-138 °C. **ν_{max} (KBr) cm^{-1}** 1759 (C=O), 1706 (N-C=O). **^{1}H NMR** (400 MHz, Chloroform-*d*) δ 8.90 (dd, *J* = 4.2, 1.7 Hz, 1H, ArH), 8.15 (dd, *J* = 8.3, 1.7 Hz, 1H, ArH), 7.88 – 7.83 (m, 2H, ArH), 7.74 – 7.67 (m, 3H, ArH), 7.52 (t, *J* = 7.8 Hz, 1H, ArH), 7.47 (dd, *J* = 7.5, 1.6 Hz, 1H), 7.41 (dd, *J* = 8.3, 4.2 Hz, 1H, ArH), 3.92 (t, *J* = 6.9 Hz, 2H, C(4)*H*), 2.89 (t, *J* = 7.5 Hz, 2H, C(2)*H*), 2.27 (m, 2H, C(3)*H*). **^{13}C NMR** (101 MHz, Chloroform-*d*) δ 171.59 (ArO-**C**=O), 168.50 (C=O), 150.58 (ArC), 147.53 (ArC), 141.30 (ArC), 136.05 (ArC), 134.07 (ArC), 132.27 (ArC), 129.61 (ArC), 126.32 (ArC), 125.96 (ArC), 123.39 (ArC), 121.82 (ArC), 121.61 (ArC), 37.37 (C(4)), 31.75 (C(2)), 24.21 (C(3)). ***m/z***= 361 [MH+] (ESI$^+$) and 383 [MNa+] (ESI$^+$). **HMRS** calculated for $C_{21}H_{17}N_2O_4$ requires 361.1183, found 361.1182 [MH+] (ESI$^+$).

Quinolin-8-yl (2*R*,13*R*)-13-((tert-butyldimethylsilyl)oxy)-6-oxo-3,4,6,13-tetrahydro-1H-benzo[a]pyrrolizine-2-carboxylate 31

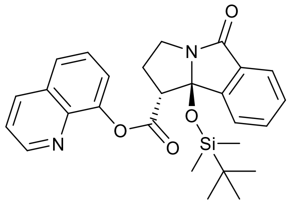

209

31 was synthesised according to General Procedure C using **29** (0.232 g, 0.64 mmol) with the crude material purified using flash column chromatography (30% ethyl acetate in pet-ether 40:60) to afford the desired product as a colourless solid (0.204 g, 66%). $\mathbf{R_f}$= 0.20 (30% ethyl acetate in pet-ether 40:60). **Mp**= 193-195 °C. $\mathbf{\nu_{max}}$ **(KBr) cm^{-1}** 1757 (C=O), 1715 (N-C=O). **^{1}H NMR** (400 MHz, Chloroform-*d*) δ 8.85 (dd, *J* = 4.2, 1.7 Hz, 1H, ArH), 8.12 (dd, *J* = 8.3, 1.7 Hz, 1H, ArH), 7.88 – 7.82 (m, 1H, ArH), 7.80 (dt, *J* = 7.5, 1.0 Hz, 1H, ArH), 7.67 (td, *J* = 7.4, 1.4 Hz, 1H, ArH), 7.62 (ddd, *J* = 8.4, 5.6, 1.4 Hz, 2H, ArH), 7.40 (dd, *J* = 8.3, 4.2 Hz, 1H, ArH), 7.32 – 7.27 (m, 1H, ArH), 6.22 (dd, *J* = 7.5, 1.3 Hz, 1H, ArH), 4.14 (dt, *J* = 11.0, 8.9 Hz, 1H (C(4)H_A), 3.89 (dd, *J* = 6.7, 0.9 Hz, 1H, (C(2)*H*), 3.61 (ddd, *J* = 11.3, 9.3, 2.1 Hz, 1H, C(4)H_B), 3.13 – 3.04 (m, 1H, (C(3)H_A), 3.03 – 2.95 (m, 1H, C(3)H_B), 0.94 (s, 9H, (C(CH_3)$_3$), 0.07 (s, 3H, Si-CH_3), -0.38 (s, 3H, Si-CH_3). **^{13}C NMR** (101 MHz, Chloroform-*d*) δ 170.34 (COOMe), 169.82 (C=O), 150.49 (C(7,12)ArC), 146.44 (ArC), 144.87 (ArC), 141.00 (ArC), 135.92 (ArC), 133.46 (ArC), 132.51 (ArC), 130.27 (ArC), 129.39 (ArC), 126.04 (ArC), 125.92 (ArC), 124.30 (ArC), 123.58 (ArC), 121.76 (ArC), 120.79 (ArC), 99.34 (C(13)), 53.15 (C(2)), 42.35 (C(4)), 31.67 (C(3)), 25.66 (C(**C**H_3)$_3$), 17.99 (Si-**C**(CH_3)$_3$), -4.12 (Si-CH_3), -4.32 (Si-CH_3). ***m/z***= 475 [MH+] (ESI^+) and 497 [MNa+] (ESI^+). **HRMS** calculated for $C_{27}H_{30}NaN_2O_4Si$ requires 475.2023, found 475.2023 [MNa+] (ESI^+).

Quinolin-8-yl 6-oxo-3,4-dihydro-1H-[a]pyrrolizine-2-carboxylate 32

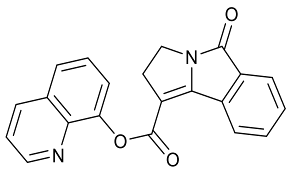

211

32 was synthesised according to General Procedure D using **31** (0.100 g, 0.21 mmol) to afford the desired product as a pale orange coloured solid (0.091 g, quantitative yield). **Mp**= +240 °C. $\mathbf{\nu_{max}}$ **(KBr) cm^{-1}** 1703 (N-C=O), 1641 (C=O). **^{1}H NMR** (500 MHz, Chloroform-*d*) δ 9.55 (d, *J* = 5.0 Hz, 1H, ArH), 8.86 (d, *J* = 8.3 Hz, 1H, ArH), 8.43 (d, *J* = 7.7 Hz, 1H, ArH), 8.07 (dd, *J* = 8.2, 1.3 Hz, 1H, ArH), 7.99 – 7.91 (m, 2H, ArH), 7.87 (t, *J* = 7.9, 1.1 Hz, 2H, ArH), 7.58 (dtd, *J* = 20.0, 7.5, 1.2 Hz, 2H, ArH), 4.10 (t, *J* = 8.0 Hz, 2H, C(4)*H*), 3.77 (t, *J* = 8.3 Hz, 2H, C(3)*H*). **^{13}C NMR** (126 MHz,

Chloroform-*d*) δ 164.66 (COOAr), 162.84 (C=O), 152.89 (C(1b)Ar**C**), 147.10 (ArC), 144.66 (C(13)C=**C**), 142.71 (ArC), 135.90 (ArC), 134.13 (ArC), 132.57 (ArC), 132.11 (ArC), 130.24 (ArC), 129.74 (ArC), 129.25 (ArC), 127.15 (ArC), 127.04 (ArC), 126.65 (ArC), 123.78 (ArC), 122.42 (ArC), 109.04 (C(2)**C**=C), 40.52 (C(4)), 33.76 (C(3)). **HRMS** calculated for $C_{21}H_{15}N_2O_3$ requires 343.1077, found 343.1077 [MH+] (ESI^+).

(1*R*,13*R*)-13-[(tert-Butyldimethylsilyl)oxy]-6-oxo-3,4,6,13-tetrahydro-1H-benzo[a]pyrrolizine-2-carboxylic acid 33

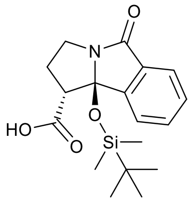

212

Method 1) Compound **8a** (0.330 g, 0.91 mmol) dissolved in a solution of THF in water (3:1, 5 mL) followed by the addition of NaOH (0.073 g, 1.82 mmol) in one portion with the reaction mixture left stirring at room temperature for 16 hours. The reaction mixture was acidified using 0.5 N HCl to pH ~3, that caused the product to precipitate out of solution. The mixture was transferred to a separating funnel and extracted using ethyl acetate (2 x 10 mL) with the combined organic layers being dried over $MgSO_4$ and concentrated by reduced pressure to afford the desired product as a colourless solid (0.317 g, quantitative yield).

Method 2) Compound **11** (0.418 g, 0.96 mmol) was added followed by a catalytic quantity of 10% Pd/C and suspended in ethanol (15 mL), with DCM being added dropwise to encourage solubility of the starting material. The flask was sealed and purged using N_2 (3x) and charged with H_2 (2x). The reaction was then fitted with two balloons pressurised with H_2 with the reaction was left stirring at room temperature for 16 hours. The reaction flask was then degassed and purged using N_2. The solvent was then filtered through a pad of Kieselguhr under pressure. The pad of Kieselguhr was then washed using methanol (15 mL) with the combined organic solvents being concentration by reduced pressure to afford the desired product as a colourless solid (0.334 g, quantitative yield). **Mp**= 186-188 °C. **ν_{max} (KBr) cm^{-1}** 1736 (C=O), 1666 (N-C=O), 1469 (C=C). **^{1}H NMR** (400 MHz, Methanol-*d*$_4$) δ 7.70 – 7.59 (m, 3H, ArH), 7.58 – 7.52 (m, 1H, ArH), 3.90 (ddd, *J* = 11.0, 9.4, 8.5 Hz, 1H, C(4)H_A), 3.45 (ddd, *J* = 11.2, 9.5, 2.0 Hz, 1H, C(4)H_B), 3.27 (d, *J* = 6.8 Hz, 1H, C(2)*H*), 2.89 – 2.72 (m, 1H, C(3)H_A), 2.59 – 2.50 (m, 1H, C(3)H_B), 0.87 (d, *J* = 8.5 Hz, 9H, C(CH_3)), -0.04 (d, *J* = 1.3 Hz, 3H, Si-CH_3), -0.46 (s, 3H, Si-CH_3). **^{13}C NMR** (101 MHz, Methanol-*d*$_4$) δ 174.90 (C=O), 172.71 (COOH), 146.18 (ArC), 134.21 (ArC), 133.86 (ArC), 131.34 (ArC), 125.20 (ArC), 123.98 (ArC), 100.71 (C(13)), 53.94 (C(2)), 43.09 (C(4)), 32.42 (C(3)), 26.00 (C(**C**H_3)$_3$, 18.70 (Si-**C**(CH_3)$_3$), -4.01

(Si-CH_3), -4.13 (Si-CH_3). ***m/z***= 370 [MNa+] (ESI^+). **HRMS** calculated for $C_{18}H_{25}NaNO_4Si$ requires 370.1445, found 370.1447 [MNa+] (ESI^+).

5-Oxo-3,4-dihydro-1H-benzo[a]pyrrolizine-2-carboxylic acid 34

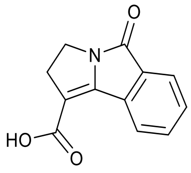

160a

Compound **33** (0.334 g, 0.96 mmol) was dissolved in a solution of TFA/H_2O (9:1, 1 mL) with methanol added to help solubility and left stirring at room temperature for 1 hour, in which the reaction mixture turned yellow. The stirrer bar was removed and washed using water (2 mL) resulting in the mixture to turn cloudy. The solvent was removed to afford the desired product as a yellow coloured solid (0.222 g, quantitative yield). **Mp**= 234-236 °C (Lit.[17] 229-231 °C). **ν_{max} (KBr) cm^{-1}** 1724 (C=O), 1646 (N-C=O), 1621 (C=O). **^{1}H NMR** (500 MHz, DMSO-d_6) δ 12.91 (s, 1H, COO-H), 8.46 (d, J = 7.7 Hz, 1H, Ar-H), 7.80 – 7.70 (m, 2H, Ar-H), 7.66 (t, J = 7.4 Hz, 1H, Ar-H), 3.85 (t, J = 8.2 Hz, 2H, C(4)*H*), 3.23 (t, J = 8.2 Hz, 2H, C(3)*H*). **^{13}C NMR** (126 MHz, DMSO-d_6) δ 165.58 (COOH), 162.48 (C6)C=O), 147.23 (C(13)C=**C**), 135.73 (ArC), 132.12 (ArC), 131.42 (ArC), 129.14 (ArC), 126.23 (ArC), 122.86 (ArC), 111.60 (C(2)**C**=C), 34.01 (C(3)). C4 resides somewhere around 39.95ppm but is hidden under DMSO, confirmed *via* HSQC NMR data. ***m/z***= 427 [2MH+] (ESI^+). **HRMS** calculated for $C_{12}H_8NO_3$ requires 214.0510, found 214.0509 [MH-] (ESI^-).

***N*-Benzyl-6-oxo-3,5-dihydro-1H-benzo[a]pyrrolizine-2-carboxamide 35a**

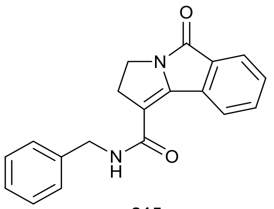

215a

Compound **34** (0.125 g, 0.58 mmol) was suspended in DMF (3 mL) followed by the addition of EDC (0.10 mL, 0.69 mmol) and DIPEA (0.20 mL, 1.16 mmol) and left stirring for 1 hour at room temperature. HOBt (0.078 g, 0.69 mmol) was added followed by the addition of benzyl amine (0.08 mL, 0.69) with the resulting reaction mixture left stirring at room temperature for 16 hours. The crude mixture was diluted with DCM (10 mL) and washed using warm water (40 °C, 5 x 10 mL) with the organic layer purified using flash column chromatography (40% ethyl acetate in DCM) to afford the desired product as a colourless solid (0.0.68 g, 35%). **R_f**= 0.39. **Mp**= 201-203 °C. **ν_{max} (KBr) cm^{-1}** 3328 (N-H), 1690 (N-C=O), 1654 (C=O) 1625 (N-C=O). **^{1}H NMR** (400 MHz, Chloroform-d) δ 8.76

(dt, J = 7.8, 1.0 Hz, 1H, ArH), 7.78 (td, 1H, ArH), 7.58 (dtd, 2H, ArH), 7.36 – 7.26 (m, 5H, ArH), 6.08 (t, J = 5.8 Hz, 1H, ArH), 4.60 (d, J = 5.8 Hz, 2H, C(1c)*H*), 3.95 (t, 2H, C(4)*H*), 3.28 (t, 2H, C(3)*H*). **^{13}C NMR** (101 MHz, Chloroform-*d*) δ 163.83 (N-C=O), 163.78 (N-C=O), 147.64 (C(13)C=**C**), 138.12 (ArC), 136.13 (ArC), 132.13 (ArC), 131.14 (ArC), 129.94 (ArC), 128.93 (ArC), 127.96 (ArC), 127.81 (ArC), 127.49 (ArC), 123.14 (ArC), 112.40 (C(2)**C**=C), 43.81 (C(1b)N-**C**H$_2$-Ar), 39.91 (C(4)), 33.77 (C(3)). ***m/z***= 305 [MH+] (ESI$^+$) and [327] MNa+] (ESI$^+$). **HMRS** calculated for $C_{19}H_{17}N_2O_2$ requires 305.1285, found 305.1285 [MH+] (ESI$^+$).

***N*-(4-Bromobenzyl)-6-oxo-3,4-dihydro-1H-benzo[a]pyrrolizine-2-carboxamide 35b**

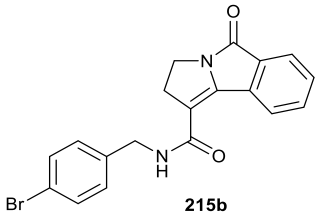

215b

Compound **34** (0.098 g, 0.45 mmol) was suspended in DMF (3 mL) followed by the addition of EDC (0.097 mL, 0.54 mmol) and DIPEA (0.16 mL, 0.9 mmol) and left stirring for 1 hour at room temperature in which all the starting material had dissolved. HOBt (0.078 g, 0.69 mmol) was added followed by the addition of 4-bromobenzylamine (0.101 g, 0.54 mmol) with the resulting reaction mixture left stirring at room temperature for 16 hours. The crude mixture was diluted with DCM (10 mL) and washed using warm water (40 °C, 5 x 10 mL) with the organic layer purified using flash column chromatography (gradient from 50% ethyl acetate in pet-ether 40:60 to 100% ethyl acetate) to afford the desired product as a colourless solid (0.068 g, 44%). **R$_f$**= 0.37 (100% ethyl acetate). **Mp**= 208-210 °C. **ν_{max} (KBr) cm^{-1}** 3333 (N-H), 1692 (N-C=O), 1655 (C=O), 1627 (C=O). **^{1}H NMR** (400 MHz, Chloroform-*d*) δ 8.80 – 8.72 (m, 1H, ArH), 7.79 (d, J = 8.0, 1.4 Hz, 1H, ArH), 7.60 (dt, 2H, ArH), 7.45 (dd, J = 8.4, 2.5 Hz, 2H, ArH), 7.21 (dd, J = 8.4, 2.0 Hz, 2H, ArH), 6.04 (s, 1H, N(1a)*H*), 4.56 (d, J = 5.9, 1.7 Hz, 2H, C(1b)*H*), 3.97 (td, J = 8.5, 4.0 Hz, 2H, C(4)*H*), 3.30 (t, J = 9.0, 7.4 Hz, 2H, C(3)*H*). **^{13}C NMR** (101 MHz, Chloroform-*d*) δ 163.85 (C=O), 163.83 (N-C=O), 148.00 (C(13)C=**C**, 137.27 (ArC), 136.16 (ArC), 132.20 (ArC), 132.01 (ArC), 131.27 (ArC), 129.92 (ArC), 129.65 (ArC), 127.53 (ArC), 123.20 (ArC), 121.71 (Ar**C**-Br), 111.97 (C(2)**C**=C), 43.16 (C(1b)ArC-**C**H$_2$-, 39.95 (C(4)) 33.76 (C(3)). ***m/z***= 789 and 791 [2M+Na+] (ESI$^+$). **HRMS** calculated for $C_{19}H_{16}BrN_2O_2$ calculated for 383.0390 and 385.0396, found 383.0392 and 385.0370 [MH+] (ESI$^+$).

***N*-(-Adamantan-1-yl)-6-oxo-3,4-dihydro-1H-benzo[a]pyrrolizine-2-carboxamide 35c**

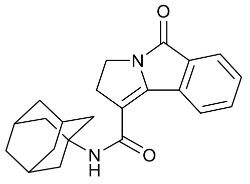

215c

Compound **34** (0.050 g, 0.23 mmol) was dissolved in DCM (5 mL) followed by the addition of EDC (0.043 g, 0.27 mmol) and left stirring at room temperature for 1 hour. 1-Adamantylamine hydrochloride (0.052 g 0.27 mmol) and DIPEA (0.049 mL, 0.27 mmol) were added and the reaction was left stirring at room temperature for 16 hours. The mixture was then diluted with DCM (5mL) and washed using water (2 x 10 mL) and dried over $MgSO_4$ and concentrated by reduced pressure. The crude material was then purified using flash column chromatography (30% ethyl acetate in DCM) to afford the desired product as a pale-yellow coloured oil (0.034 g, 41%). **R_f**= 0.34. **ν_{max} (KBr) cm^{-1}** 3321 (N-H), 1698 (N-C=O), 1655 (C=O), 1628 (N-C=O). **^{1}H NMR** (500 MHz, Chloroform-*d*) δ 8.81 (dd, *J* = 7.5, 1.2 Hz, 1H, Ar-H), 7.84 (dt, *J* = 7.3, 0.9 Hz, 1H, Ar-H), 7.63 (td, *J* = 7.5, 1.3 Hz, 1H, Ar-H), 7.58 (td, *J* = 7.4, 1.2 Hz, 1H, Ar-H), 5.94 (d, *J*= 8.0 Hz, 1H, N(1a)N*H*), 4.06 (t, *J* = 8.9, 7.5 Hz, 2H, C(4)*H*), 3.38 (t, 2H, C(3)*H*), 2.09 – 2.03 (m, 2H, C(Adamantane)*H*), 1.98 – 1.56 (m, 15H, C(Adamantante)*H*). **^{13}C NMR** (126 MHz, Chloroform-*d*) δ 163.88 (C=O), 163.07 (N-C=O), 147.34 (C(13)C=**C**), 136.18 (ArC, 132.10 (ArC), 131.08 (ArC), 130.04 (ArC, 127.56 (ArC), 123.18 (ArC), 112.83 (C(2)**C**=C), 39.91 C(4)), 37.56 (C(adamantane)), 37.24 (C(adamantane)), 33.87 (C(3)), 32.27 (C(adamantane)), 32.14 (C(adamantane)), 27.31 (C(adamantane)), 27.27 (C(adamantane)). ***m/z***= 349 [MH+] (ESI$^+$).

6-Oxo-*N*-(pyridin-2-ylmethyl)-3,4-dihydro-1H-pyrrolo[2,1-a]isoindole-2-carboxamide 35e

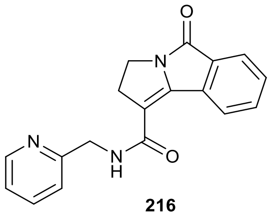

216

Compound **34** (0.200 g, 0.93 mmol) was suspended in DMF (3 mL) followed by the addition of EDC (0.20 mL, 1.11 mmol) and DIPEA (0.19 mL, 1.11 mmol) and left stirring for 1 hour at room temperature in which all the starting material had dissolved. HOBt (0.150 g, 1.11 mmol) was added followed by the addition of 2-picolylamine (0.12 mL, 1.11 mmol) with the resulting reaction mixture left stirring at room temperature for 16 hours. The crude mixture was diluted with DCM (10 mL) and washed using warm water (40 °C, 5 x 10 mL) with the organic layer purified using flash column chromatography (40% ethyl acetate in pet-ether 40:60) with the desired fractions collected, combined and concentrated by reduced pressure to afford the desired product as a light blue coloured solid

(0.020 g, 7%). **R$_f$**= 0.19. **^{1}H NMR** (400 MHz, Chloroform-*d*) δ 8.46 – 8.41 (m, 1H, ArH), 7.84 – 7.79 (m, 1H, ArH), 7.61 – 7.51 (m, 2H, ArH), 7.49 – 7.35 (m, 4H, ArH), 5.33 (s, 2H, C(1b)ArC-C**H**$_2$-N), 3.96 (dd, *J* = 9.1, 7.7 Hz, 2H, (C(4)*H*), 3.39 (dd, *J* = 9.1, 7.7 Hz, 2H, (C(3)*H*). ***m/z***= 306 [MH+] (ESI$^+$) and 328 [MNa+] (ESI$^+$). **HRMS** calculated for $C_{18}H_{16}N_3O_2$ requires 306.1237, found 306.1125 [MH+] (ESI$^+$).

(2*R*,13*S*)-6-Oxo-3,4,6,12-tetrahydro-1H-benzo[a]pyrrolizine-2-carboxylic acid 36

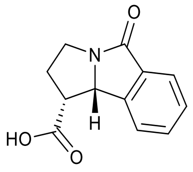

217

Method 1) Compound **27a** (0.051 g, 0.22 mmol) was suspended in water (5 mL) with NaOH (0.017g, 0.44 mmol) being added in one portion. The reaction mixture was then left stirring for 16 hours at room temperature. The reaction mixture was then acidified to pH ~3 using 0.5 N HCl with a small quantity of precipitate forming. The aqueous layer was then extracted using ethyl acetate (3 x 5 mL) with the combined organic layers being combined and concentrated by reduced pressure to afford the desired product as a a colourless solid (0.032 g, 72%).

Method 2) **36** was synthesised according to General Procedure E using **34** (0.500 g, 2.18 mmol) to afford the desired product as a colourless solid (0.481 g, quantitative yield). **ν$_{max}$ (KBr) cm^{-1}** 1721 (C=O), 1644 (C-N=O), 1614 (C=O). **^{1}H NMR** (400 MHz, Methanol-*d*$_4$) δ 7.68 (d, *J* = 7.5 Hz, 1H, Ar-H), 7.59 (d, 2H, Ar-H), 7.49 (dt, *J* = 8.1, 4.1 Hz, 1H, Ar-H), 5.14 (d, *J* = 6.7 Hz, 1H, C(13)*H*), 3.87 (dt, *J* = 11.2, 8.7 Hz, 1H, C(4)*H$_A$*), 3.45 (ddd, *J* = 11.4, 8.1, 3.9 Hz, 1H, C(4)*H$_B$*), 3.37 (td, *J* = 6.3, 2.3 Hz, 1H, C(2)*H*), 2.63 – 2.56 (m, 2H, C(3)*H*). **^{13}C NMR** (101 MHz, Methanol-*d*$_4$) δ 174.79 (COOH), 173.61 (C=O), 144.75 (ArC), 135.60 (ArC), 132.96 (ArC), 129.85 (ArC), 125.16 (ArC), 124.25 (ArC), 67.95 (C(13)), 42.47 (C(2)), 42.44 (C(4)), 33.52 (C(3)). ***m/z***= 435 [2MNa+] (ESI$^+$). **HRMS** calculated $C_{12}H_{12}NO_3$ requires 218.0812, found 218.0810 [MH+] (ESI$^+$).

(1*R*,13*S*)-6-Oxo-*N*-(pyridin-2-ylmethyl)-3,4,6,13-tetrahydro-1H-benzo[a]pyrrolizine-2-carboxamide 37

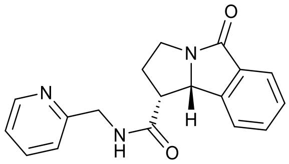

218

Method 1) In an oven dried flask that had been purged with N_2, compound **36** (0.100 g, 0.46 mmol) was suspended in $SOCl_2$ (0.33 mL, 4.60 mmol) with a few drops of anhydrous DCM to assist with

solubility and left stirring under an inert atmosphere of N_2 at room temperature for 2 hours. The solvent was then removed and azeotroped with anhydrous DCM (3 x 5 mL) by reduced pressure. The oil was then redissolved in anhydrous DCM with the flask purged with N_2, followed by the addition of 2-picolylamine (0.095 mL, 0.92 mmol) added dropwise over 5 minutes. The resulting reaction mixture was then left stirring at room temperature for 16 hours. The crude mixture was diluted with DCM (10 mL) and washed using warm water (40 °C, 5 x 10 mL) with the organic layer purified using flash column chromatography (silica pre-doped using 2.0% TEA, with the solvent being using to run the column 2.0% TEA, 2.5% MeOH in DCM) with the desired fractions collected, combined and concentrated by reduced pressure to afford the desired product as a pale-yellow coloured oil (0.074 g, 52%).

Method 2) Compound **36** (0.100 g, 0.46 mmol) was suspended in DMF (3 mL) followed by the addition of EDC (0.10 mL, 0.55 mmol) and DIPEA (0.24 mL, 1.11 mmol) and left stirring for 1 hour at room temperature in which all the starting material had dissolved. HOBt (0.85 g, 0.55 mmol) was added followed by the addition of 2-picolylamine (0.056 mL, 0.55 mmol) with the resulting reaction mixture left stirring at room temperature for 16 hours. The crude mixture was diluted with DCM (10 mL) and washed using warm water (40 °C, 5 x 10 mL) with the organic layer purified using flash column chromatography (silica pre-doped using 2.0% TEA, with the solvent being using to run the column 2.0% TEA, 2.5% MeOH in DCM) with the desired fractions collected, combined and concentrated by reduced pressure to afford the desired product pale yellow coloured oil (0.083g, 58%).

R_f = 0.51 (2.0% TEA, 2.5% MeOH in DCM). **ν_{max} (KBr) cm^{-1}** 3292 (N-H), 1657 (N-C=O), 1591 (N-H). **^{1}H NMR** (400 MHz, Chloroform-*d*) δ 8.51 (dt, *J* = 4.9, 1.4 Hz, 1H, ArH), 7.81 – 7.73 (m, 1H, ArH), 7.69 (td, *J* = 7.7, 1.8 Hz, 1H, ArH), 7.50 – 7.41 (m, 3H, ArH), 7.30 (d, *J* = 7.8 Hz, 1H, ArH), 7.22 (ddd, *J* = 7.6, 4.8, 1.1 Hz, 1H, ArH), 4.99 (d, *J* = 9.7 Hz, 1H, C(13)*H*), 4.70 (dd, *J* = 16.4, 5.0 Hz, 1H, C(1b)ArCC**H_2**N), 4.61 (dd, *J* = 16.4, 4.7 Hz, 1H, C(1b)ArCC**H_2**N), 3.77 (dt, *J* = 11.6, 8.7 Hz, 1H, C(4)H_A), 3.55 (ddd, *J* = 11.8, 9.5, 2.6 Hz, 1H, C(3)H_B), 2.72 (ddt, *J* = 12.9, 11.3, 9.3 Hz, 1H, C(3)H_A, 2.65 – 2.57 (m, 1H, C(3)H_B), 2.31 (ddd, *J* = 11.2, 9.7, 7.5 Hz, 1H, (C(2)*H*). **^{13}C NMR** (101 MHz, Chloroform-*d*) δ 171.46 (N-C=O), 170.93 (C=O), 155.77 (C(1c)CH_2-**C**Ar), 149.11 (ArC), 145.13 (ArC), 137.04 (ArC), 133.48 (ArC), 131.94 (ArC) 128.89 (ArC), 124.08 (ArC), 123.38 (ArC), 122.72 (ArC), 122.28 (ArC), 66.77 (C(13)), 49.25 (C(1b)ArC-**C**H_2-N), 44.58 (C(2)), 41.64 (C(4)), 33.86 (C(3)). ***m/z***= 306 [MH-] (ESI^-) and 308 [MH+] (ESI^+). **HRMS** calculated for $C_{18}H_{18}N_3O_2$ requires 308.1394, found 308.1393 [MH+] (ESI^+).

References

[1] E. Guénin, M. Monteil, N. Bouchemal, T. Prangé and M. Lecouvey, *Eur. J. Org. Chem.*, 2007, **20**, 3380–3391.
[2] K. S. Ko, G. Park, Y. Yu and N. L. Pohl, *Org. Lett.*, 2008, **10**, 5381–5384.
[3] H. Wu, J. Wu, W. Zhang, Z. Li, J. Fang, X. Lian, T. Qin, J. Hao, Q. Zhou and S. Wu, *Bioorg. Med. Chem. Lett.*, 2019, **29**, 870–872
[4] L. R. Caswell and K. C. C. Yang, *J. Chem. Eng. Data*, 1968, **13**, 291–292.
[5] T. J. M. Kaml, S. A. Khan, *Indian J. Hetrocyclic Chem.*, 2010, **19**, 321–324.
[6] A. Staubli, E. Ron and R. Langer, *J. Am. Chem. Soc.*, 1990, **112**, 4419–4424
[7] J. C. Richards and I. D. Spenser, *Can. J. Chem.*, 1982, **60**, 2810–2820.
[8] P. Beringer and M. E. Winter, *Basic Clin. Pharmacokinet. Fifth Ed.*, 2011, **5**, 3–22
[9] R. Tomar, D. Bhattacharya and S. A. Babu, *Tetrahedron*, 2019, **75**, 2447–2465
[10] Y. Kosdo, *Biopolymers*, 1970, **9**, 41–52.
[11] G. G. Vatulina, T. N. Tuzhilkova, T. V. Matveeva, V. P. Krasnov, N. L. Burde and L. V. Alekseeva, *Pharm. Chem. J.*, 1986, **20**, 647–653
[12] M. H. Chen, O. P. Goel, J. Magano, J. R. Rubin, W. Company and A. Arbor, *Bioorg. Med. Chem. Lett.*, 1999, **9**, 1587–1592.
[13] F. E. King, J. W. Clark-Lewis, R. Wade and W. A. Swindin, *J. Chem. Soc.*, 1957, **166**, 873–880.
[14] J. A. Robl, *Tetrahedron Lett.*, 1994, **35**, 393–396.
[15] T. H. Fife and N. W. Duddy, *J. Am. Chem. Soc.*, 1983, **105**, 74–79.
[16] G. Kim and G. Keum,, *Heterocycles*, 1997, **45**, 1979–1988.
[17] R. M. De Figueiredo, R. Fröhlich and M. Christmann, *Angew. Chemie - Int. Ed.*, 2007, **46**, 2883–2886

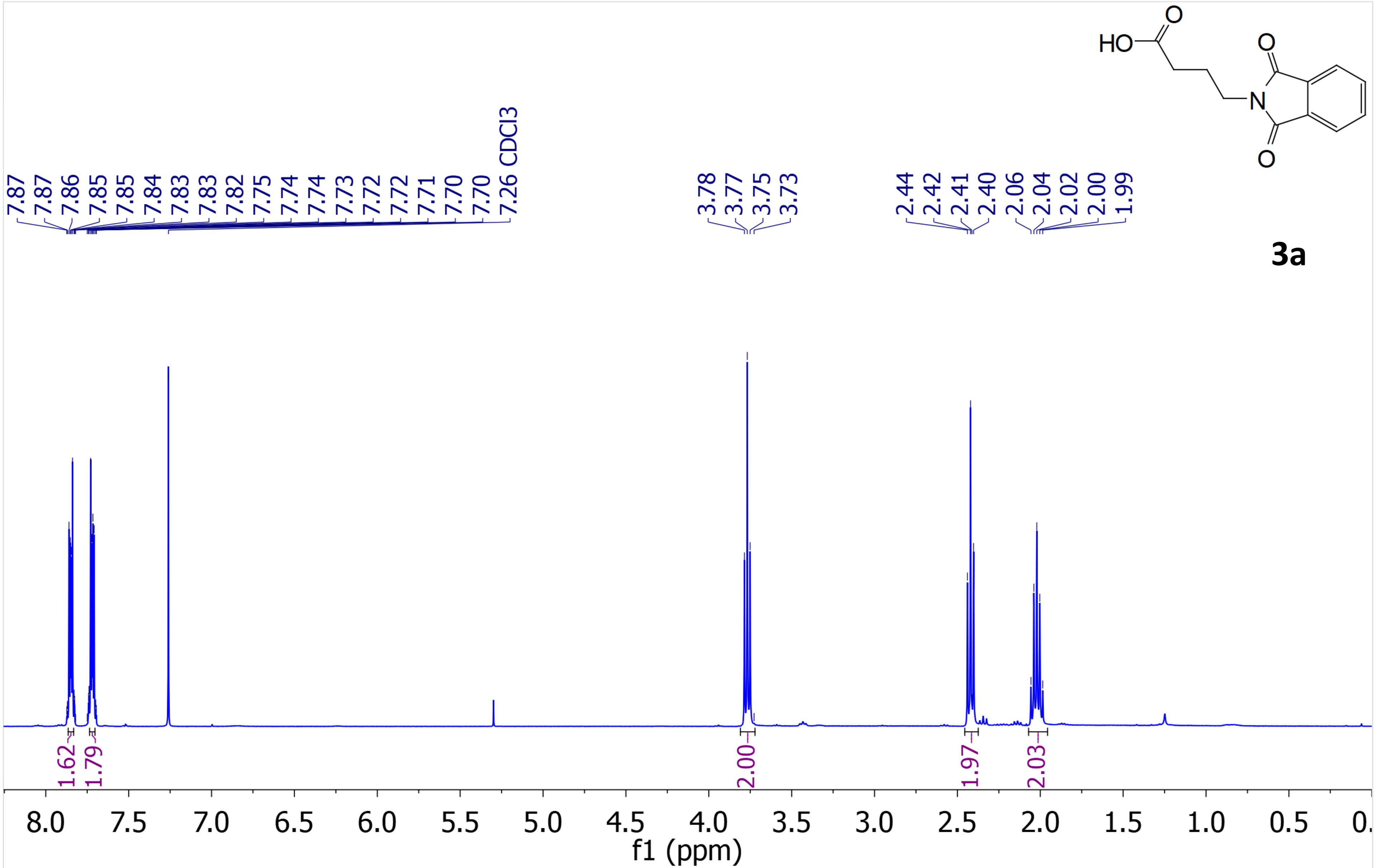

^{1}H NMR (400 MHz, Chloroform-*d*) δ 7.85 (dd, *J* = 5.4, 3.1 Hz, 2H), 7.72 (dd, *J* = 5.4, 3.0 Hz, 2H), 3.77 (t, *J* = 6.8 Hz, 2H), 2.42 (t, *J* = 7.4 Hz, 2H), 2.02 (p, *J* = 7.1 Hz, 2H).

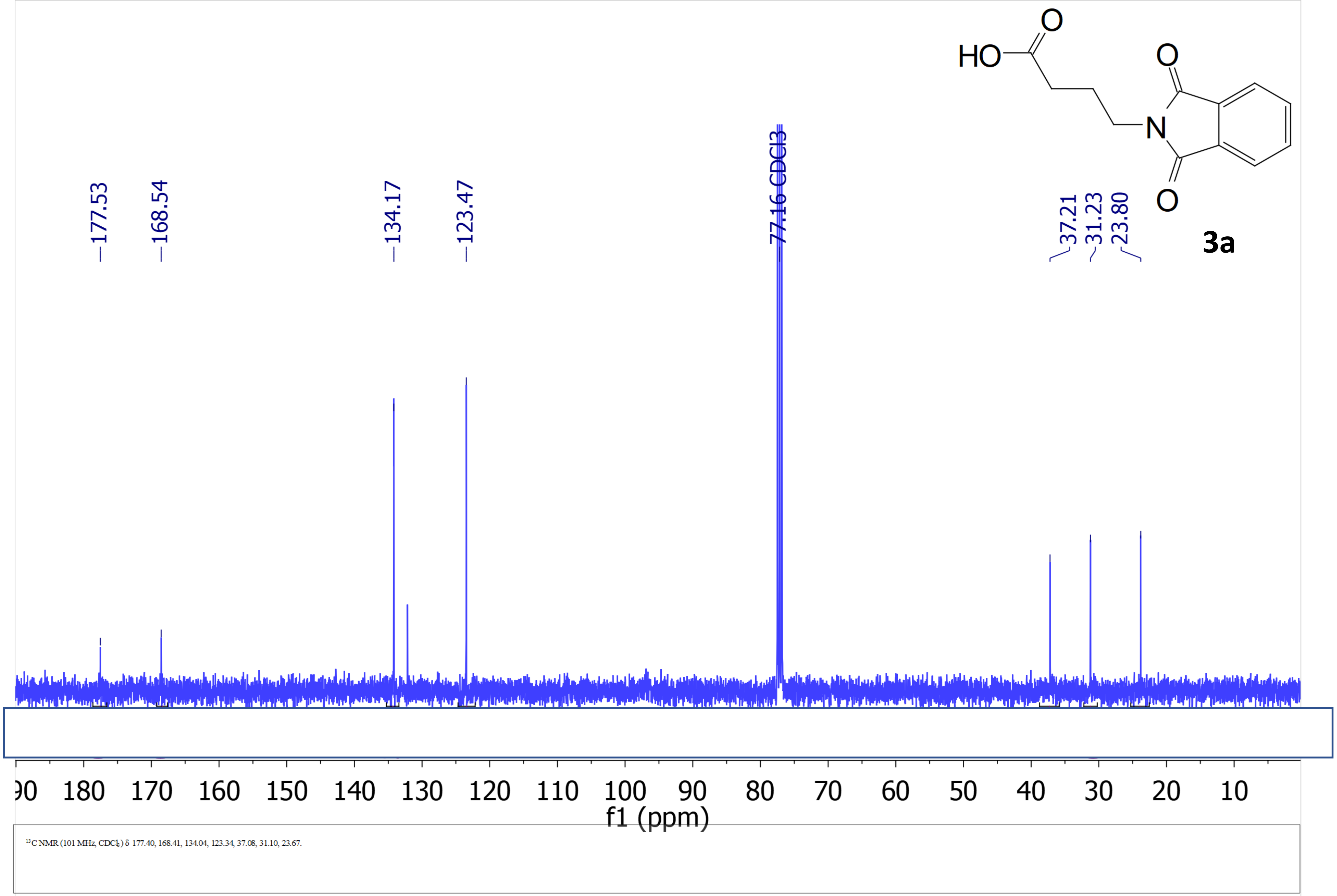

^{13}C NMR (101 MHz, $CDCl_3$) δ 177.40, 168.41, 134.04, 123.34, 37.08, 31.10, 23.67.

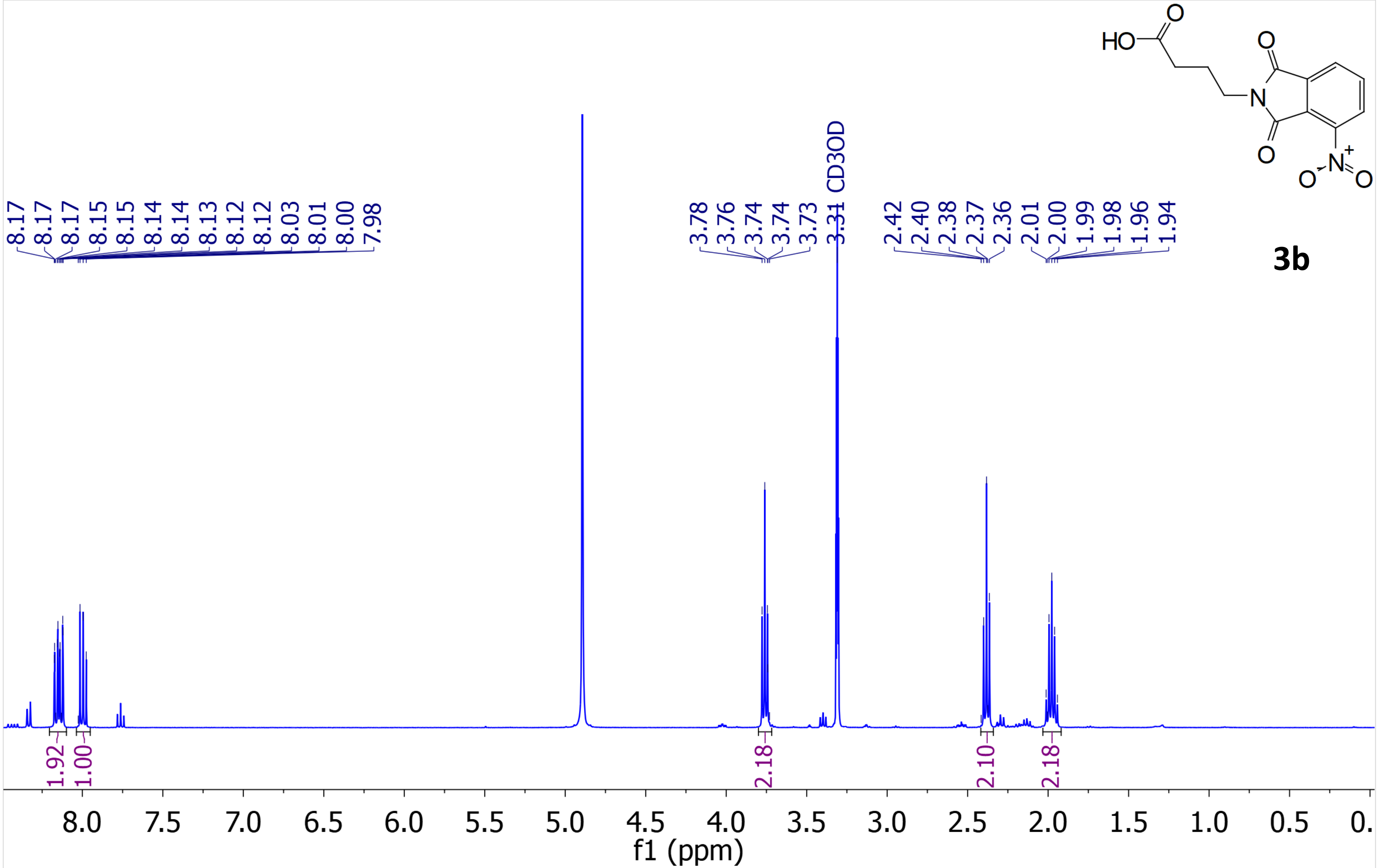

3b

^{1}H NMR (400 MHz, Methanol-d_4) δ 8.15 (ddd, J = 12.8, 7.8, 0.9 Hz, 2H), 8.04 – 7.95 (m, 1H), 3.76 (t, J = 6.8 Hz, 2H), 2.38 (t, J = 7.2 Hz, 2H), 1.98 (p, J = 7.0 Hz, 2H).

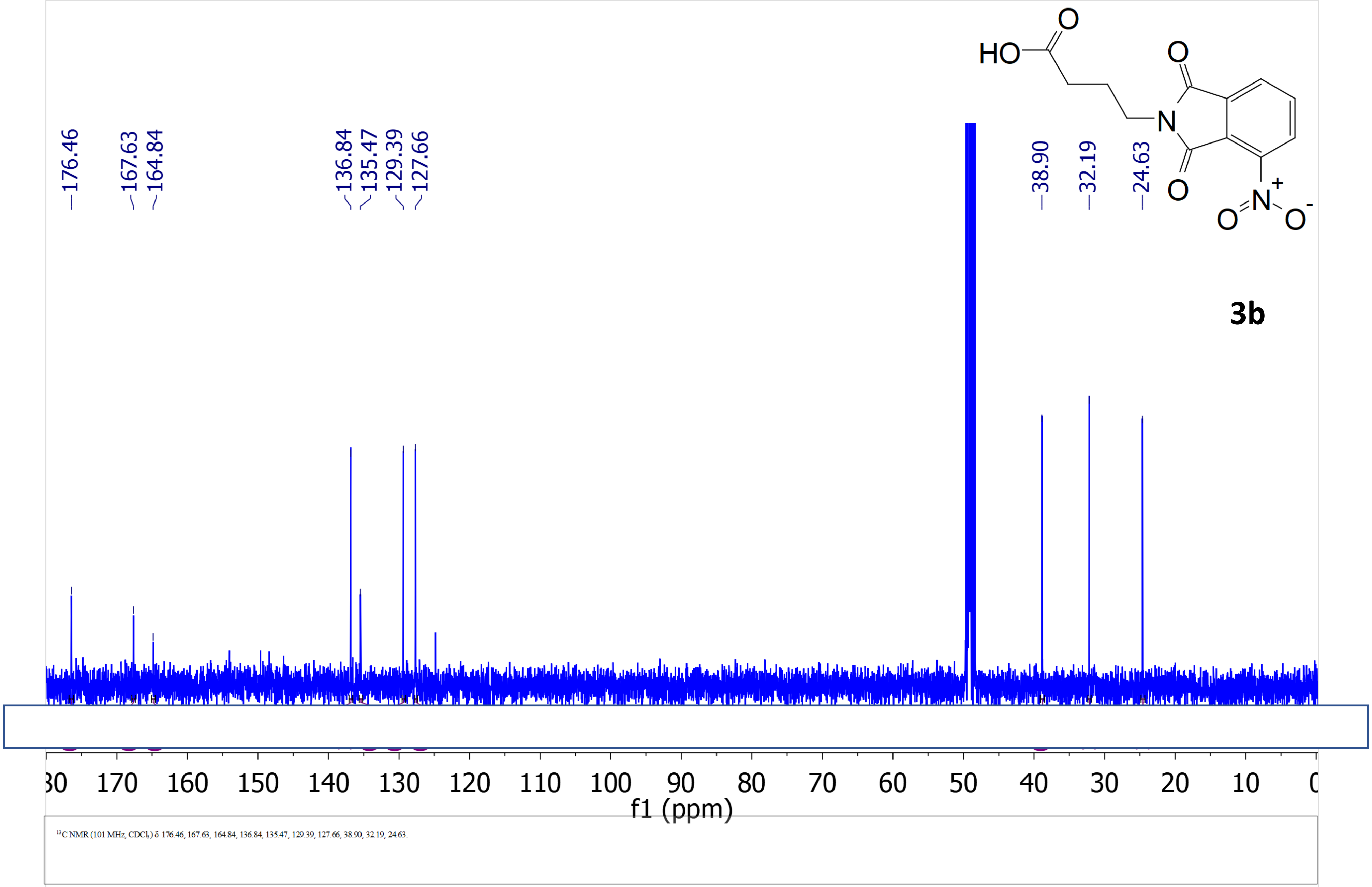

^{13}C NMR (101 MHz, $CDCl_3$) δ 176.46, 167.63, 164.84, 136.84, 135.47, 129.39, 127.66, 38.90, 32.19, 24.63.

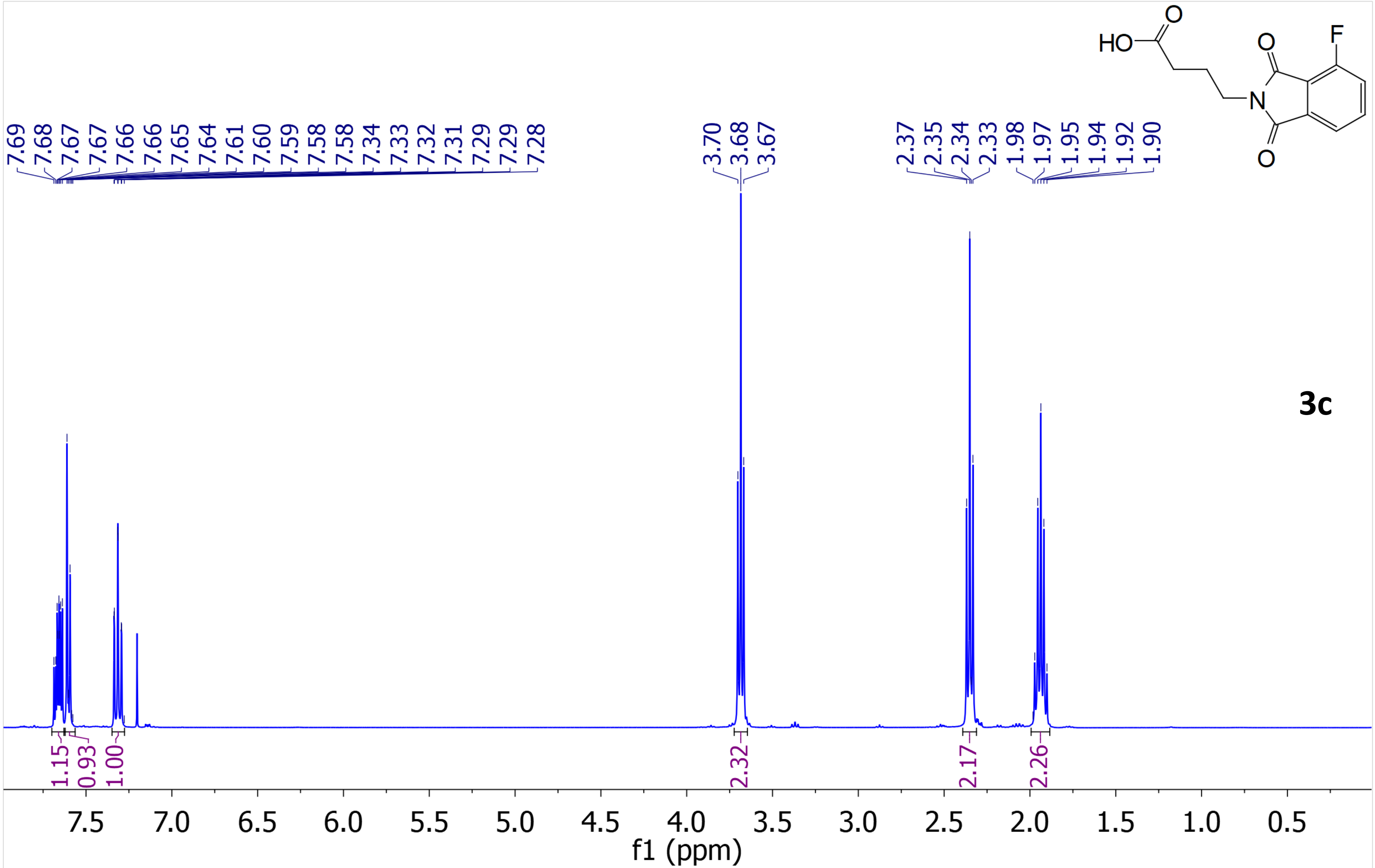

3c

^{1}H NMR (400 MHz, Chloroform-*d*) δ 7.66 (ddd, *J* = 8.2, 7.3, 4.3 Hz, 1H), 7.60 (d, *J* = 7.1 Hz, 1H), 7.31 (td, *J* = 8.5, 0.9 Hz, 1H), 3.68 (t, *J* = 6.8 Hz, 2H), 2.35 (t, *J* = 7.4 Hz, 2H), 1.94 (p, *J* = 7.1 Hz, 2H).

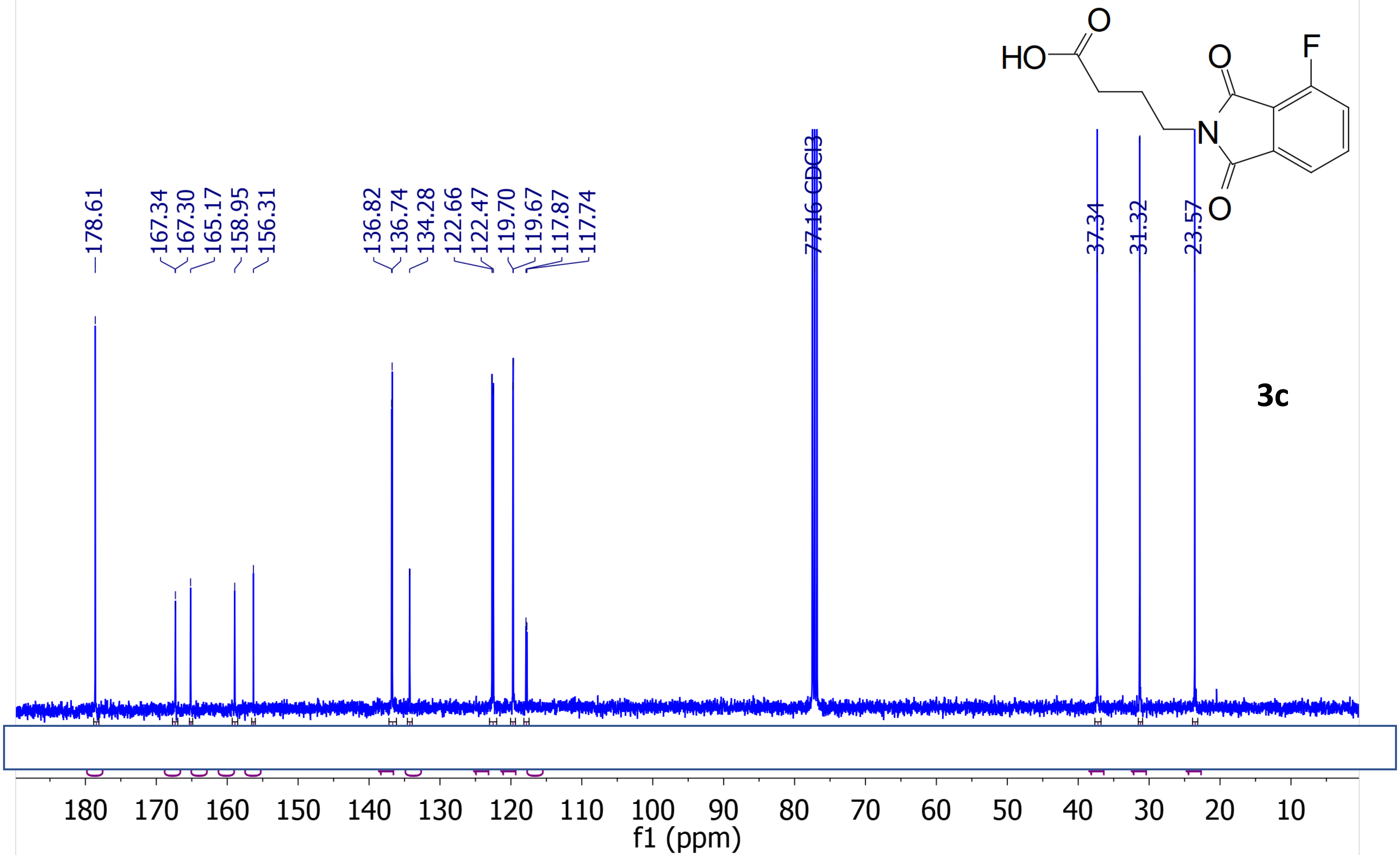

^{13}C NMR (101 MHz, Chloroform-*d*) δ 178.61, 167.32 (d, *J* = 3.1 Hz), 165.17, 158.95, 156.31, 136.78 (t, *J* = 7.8 Hz), 134.28, 122.56 (t, *J* = 19.8 Hz), 119.69 (t, *J* = 3.5, 3.1 Hz), 117.81 (t, *J* = 12.7 Hz), 37.34, 31.32, 23.57 (d, *J* = 3.0 Hz).

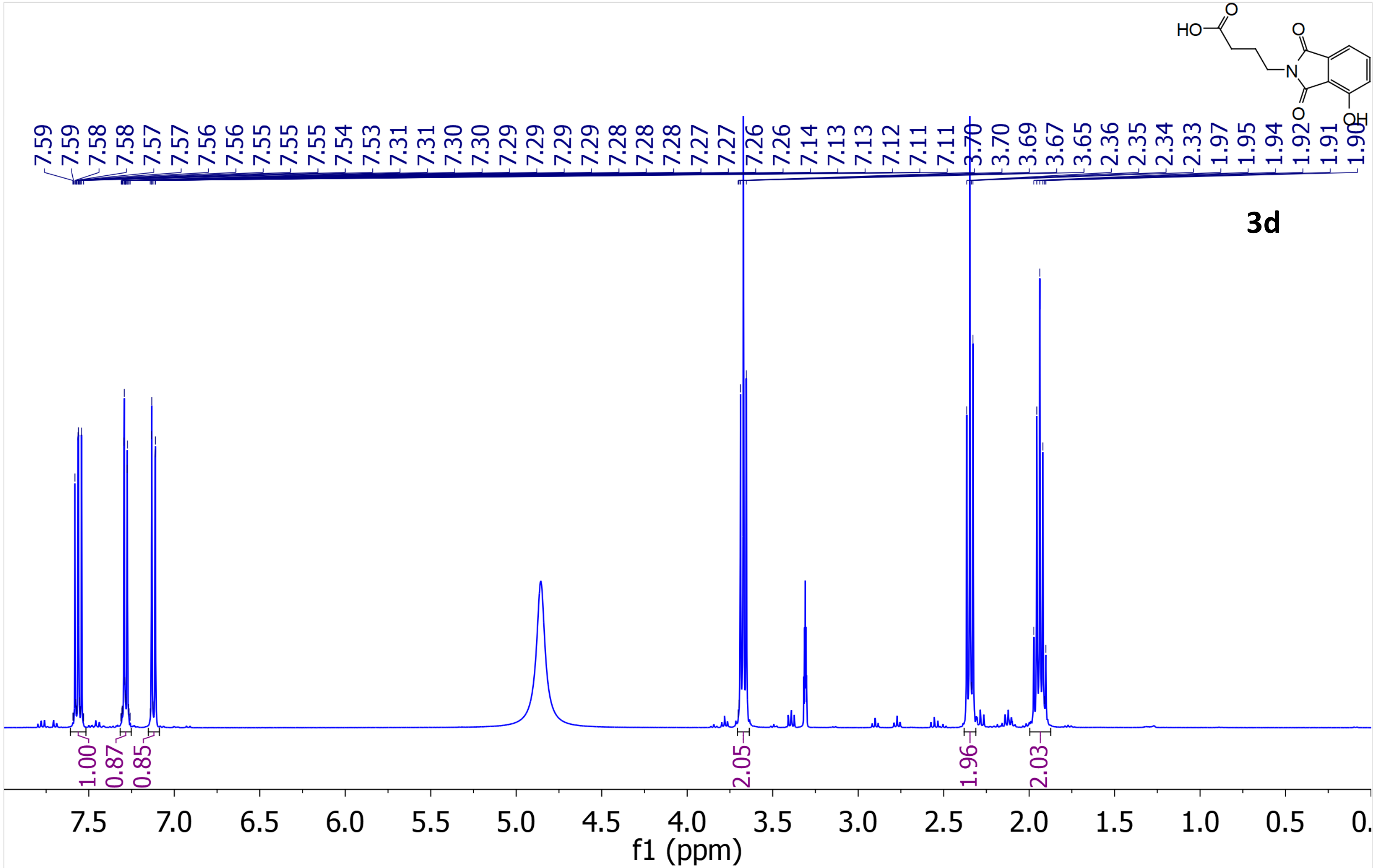

^{1}H NMR (400 MHz, Methanol-d_4) δ 7.56 (dd, J = 8.4, 7.2 Hz, 1H), 7.28 (dd, J = 7.2, 0.7 Hz, 1H), 7.12 (dd, J = 8.4, 0.7 Hz, 1H), 3.67 (t, J = 6.8 Hz, 2H), 2.35 (t, J = 7.3 Hz, 2H), 1.94 (p, J = 7.0 Hz, 2H).

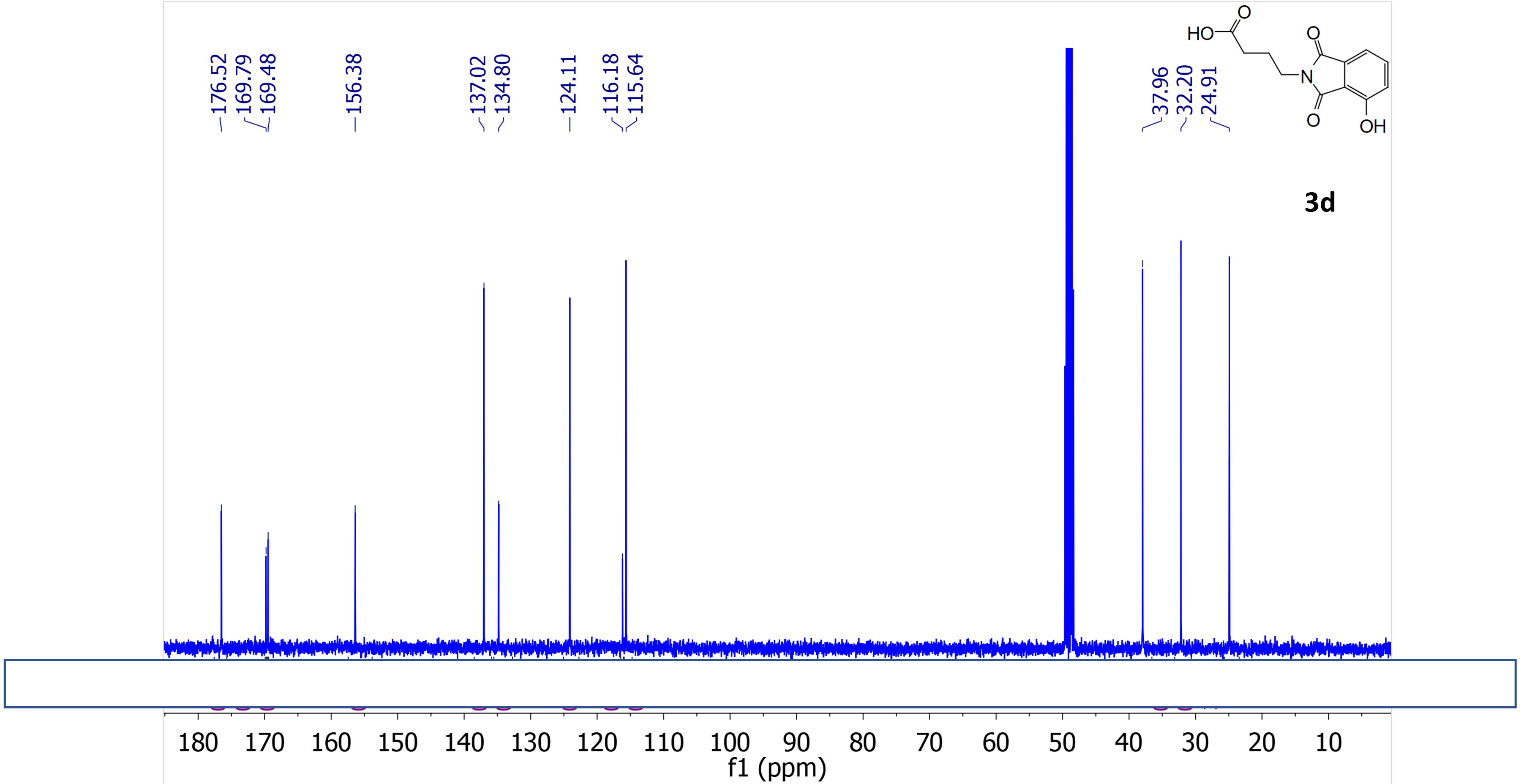

^{13}C NMR (101 MHz, MeOD) δ 176.52, 169.79, 169.48, 156.38, 137.02, 134.80, 124.11, 116.18, 115.64, 37.96, 32.20, 24.91.

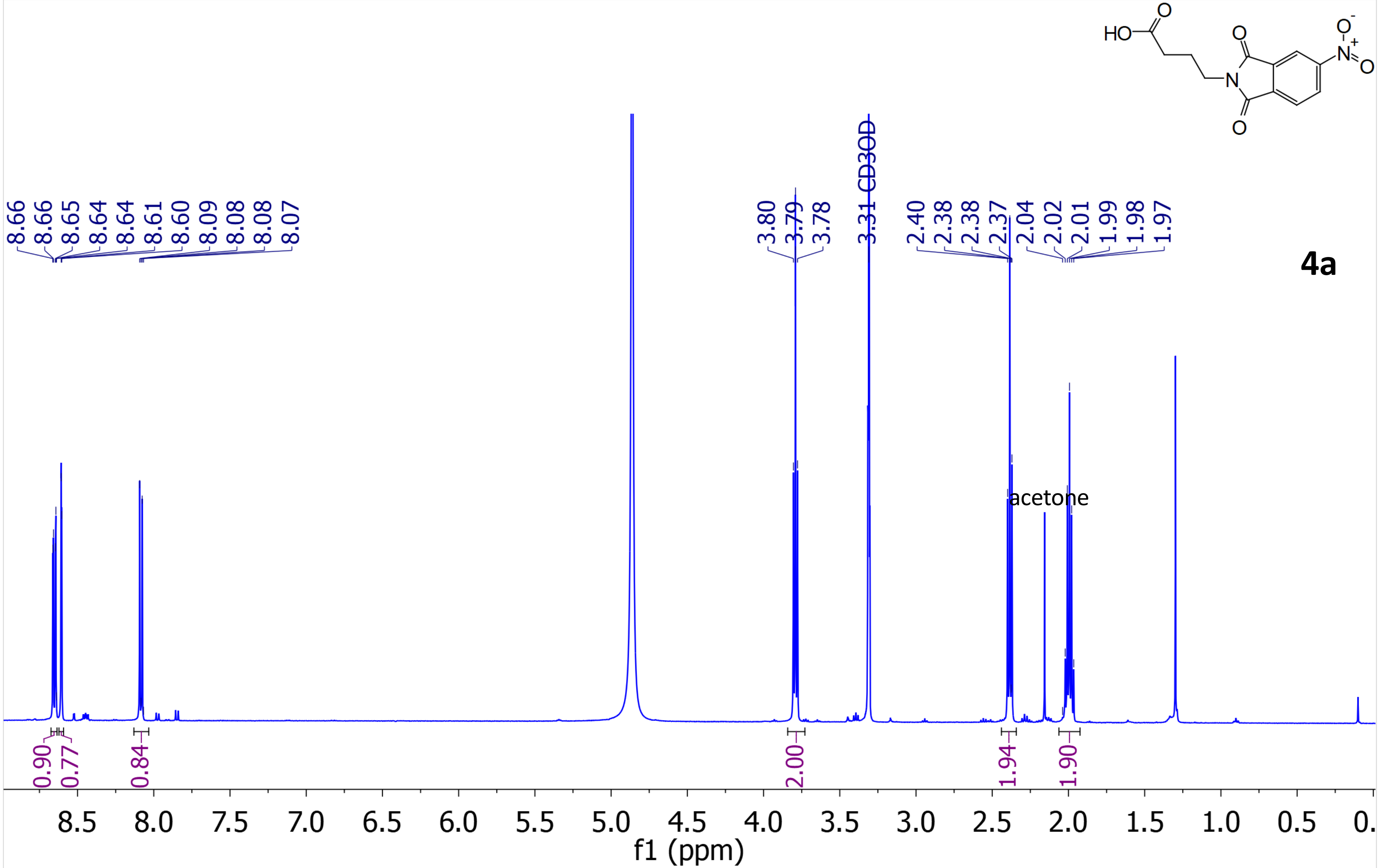

^{1}H NMR (500 MHz, Methanol-d_4) δ 8.65 (dd, J = 8.1, 2.0 Hz, 1H), 8.61 (d, J = 2.0 Hz, 1H), 8.08 (d, J = 8.1 Hz, 1H), 3.79 (t, J = 6.8 Hz, 2H), 2.38 (t, J = 7.2 Hz, 2H), 1.99 (p, J = 6.9 Hz, 2H).

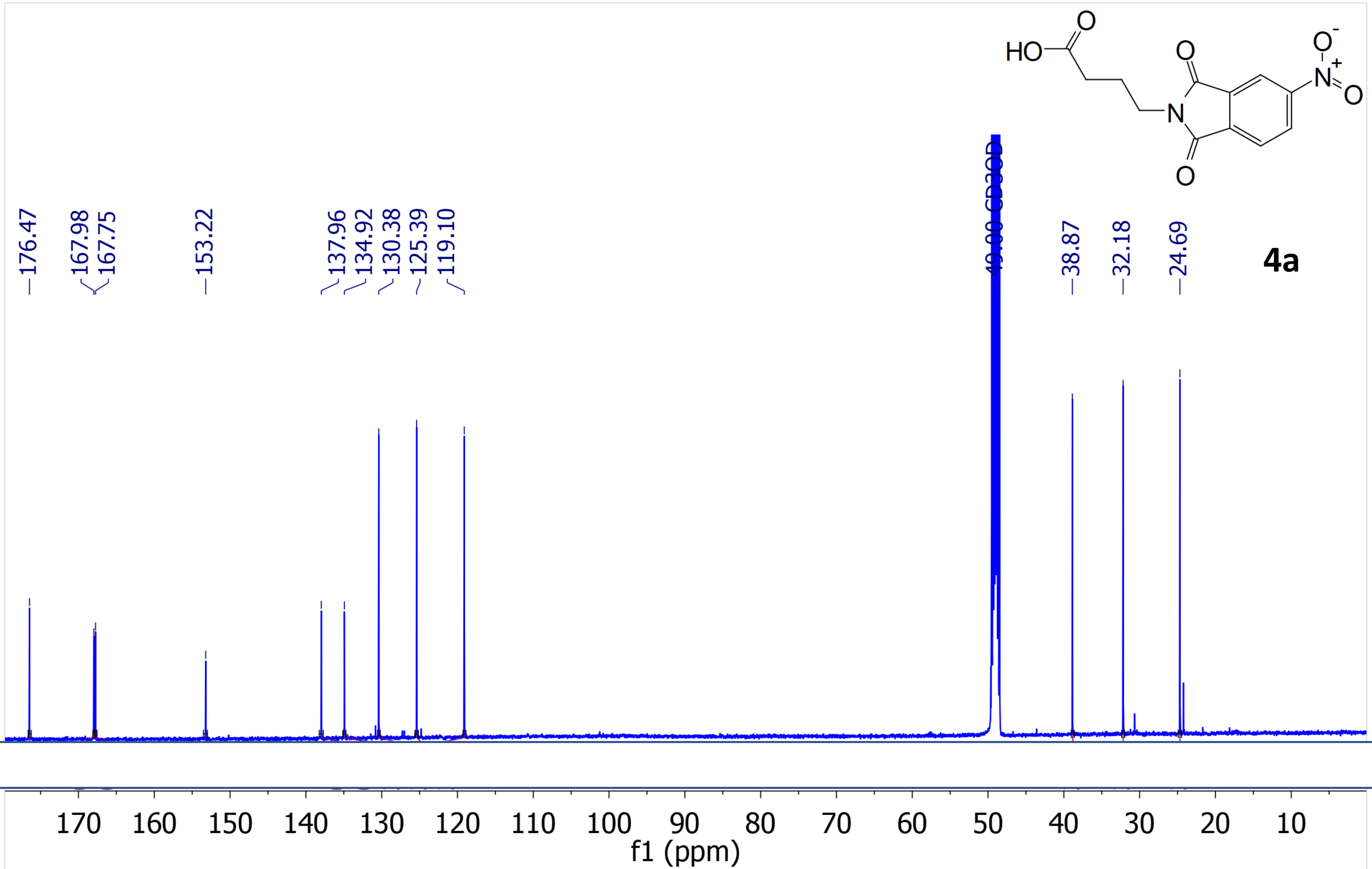

4a

^{13}C NMR (126 MHz, MeOD) δ 176.47, 167.98, 167.75, 153.22, 137.96, 134.92, 130.38, 125.39, 119.10, 38.87, 32.18, 24.69.

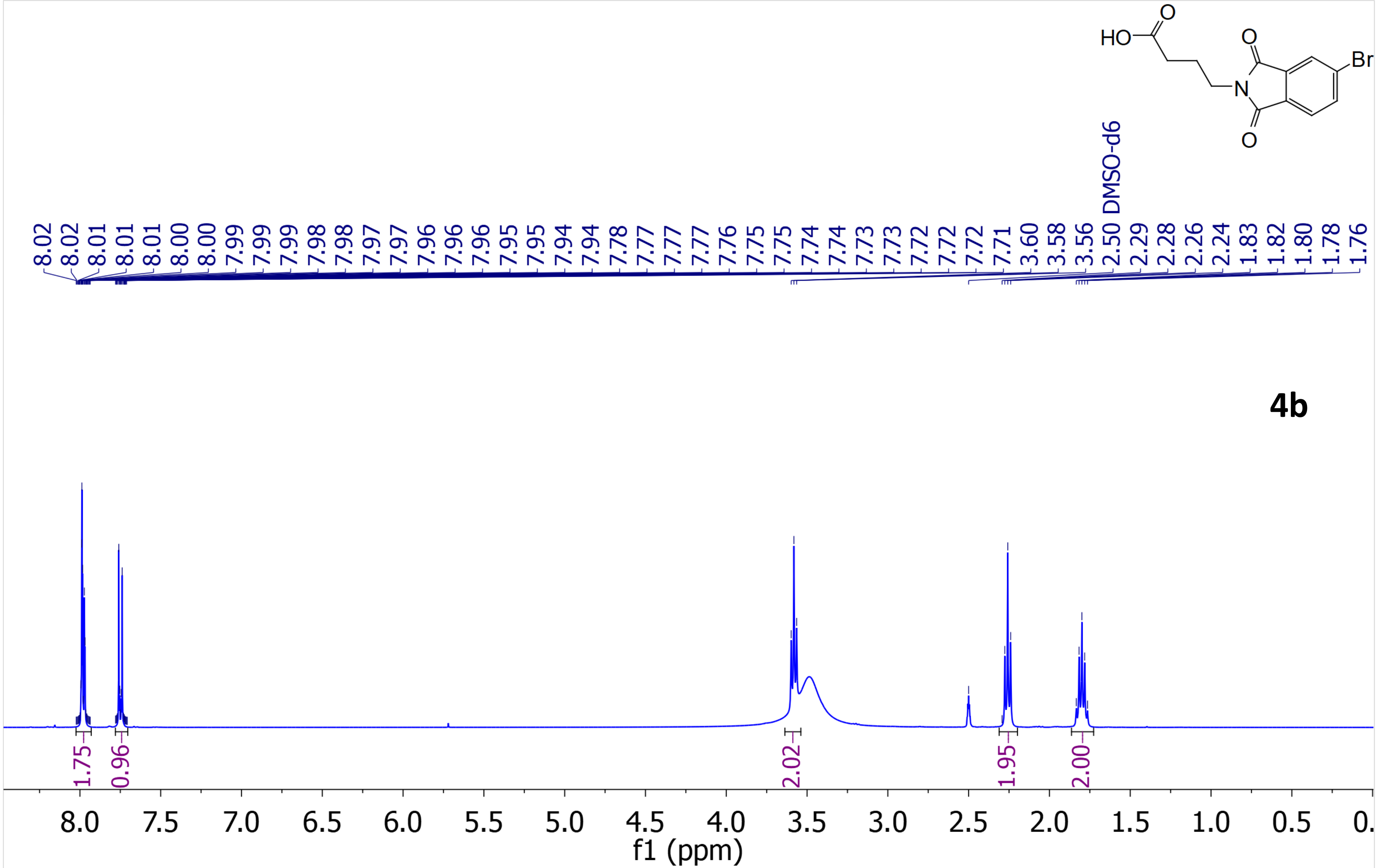

4b

^{1}H NMR (400 MHz, DMSO-d_6) δ 8.02 – 7.93 (m, 2H), 7.78 – 7.70 (m, 1H), 3.58 (t, J = 6.8 Hz, 2H), 2.26 (t, J = 7.2 Hz, 2H), 1.80 (p, J = 7.0 Hz, 2H).

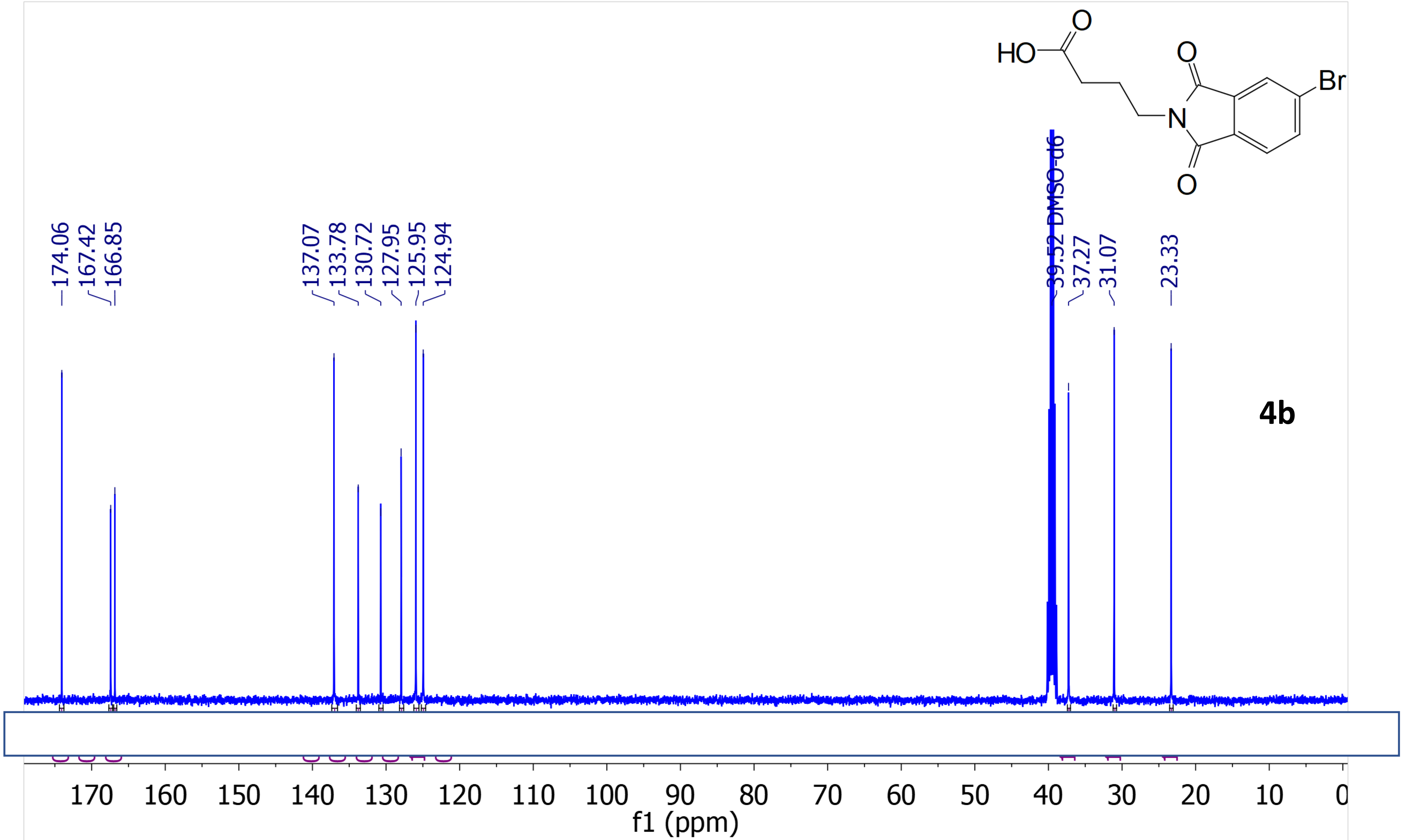

^{13}C NMR (101 MHz, DMSO) δ 174.06, 167.42, 166.85, 137.07, 133.78, 130.72, 127.95, 125.95, 124.94, 37.27, 31.07, 23.33.

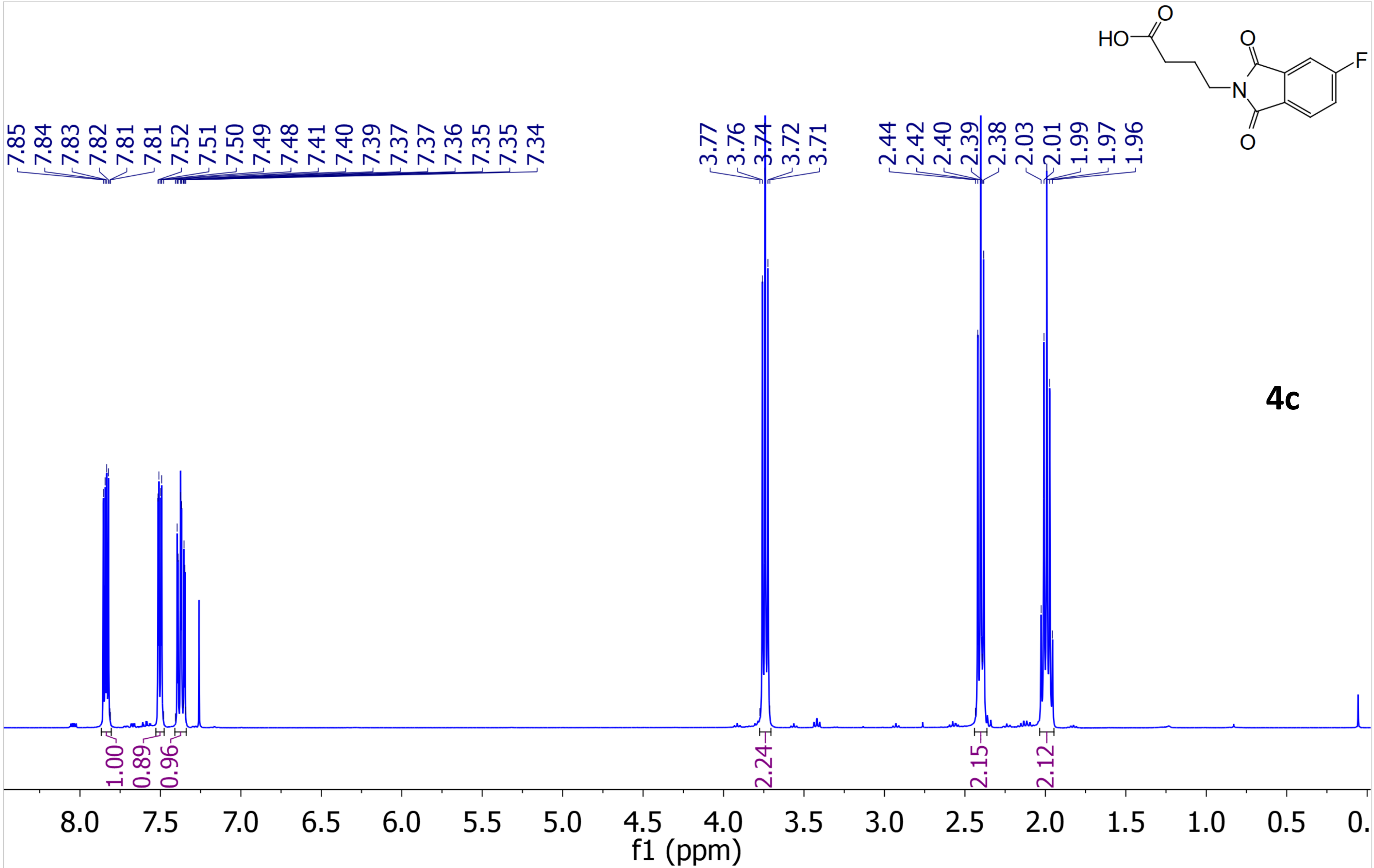

^{1}H NMR (400 MHz, Chloroform-*d*) δ 7.84 (q, *J* = 8.2, 4.5 Hz, 1H), 7.50 (dd, *J* = 7.0, 2.3 Hz, 1H), 7.37 (td, *J* = 8.5, 2.3 Hz, 1H), 3.74 (t, *J* = 6.8 Hz, 2H), 2.40 (t, *J* = 7.4 Hz, 2H), 1.99 (p, *J* = 7.1 Hz, 2H).

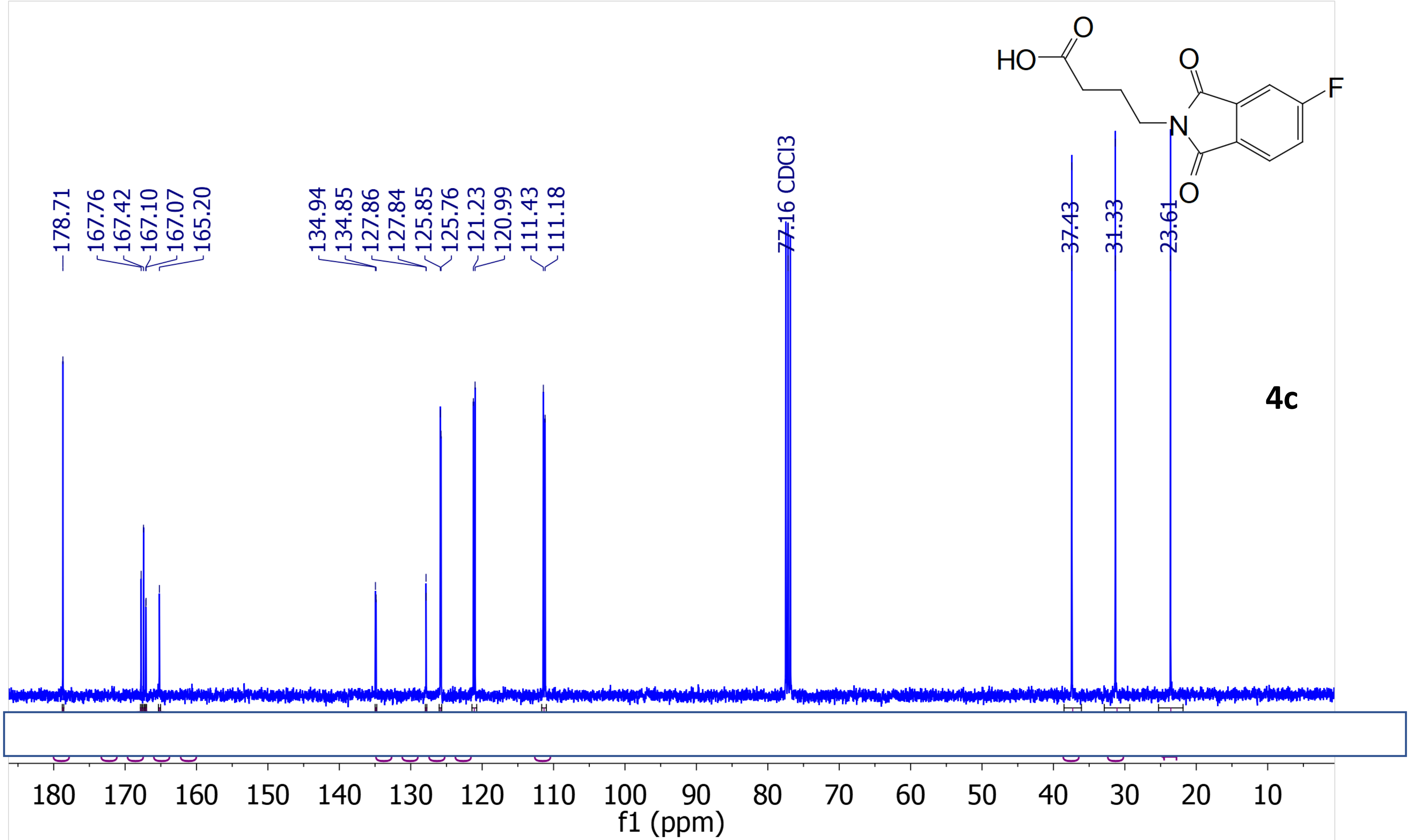

^{13}C NMR (101 MHz, $CDCl_3$) δ 178.71, 167.76, 167.42, 167.10, 167.07, 165.20, 134.94, 134.85, 127.86, 127.84, 125.85, 125.76, 121.23, 120.99, 111.43, 111.18, 37.43, 31.33, 23.61.

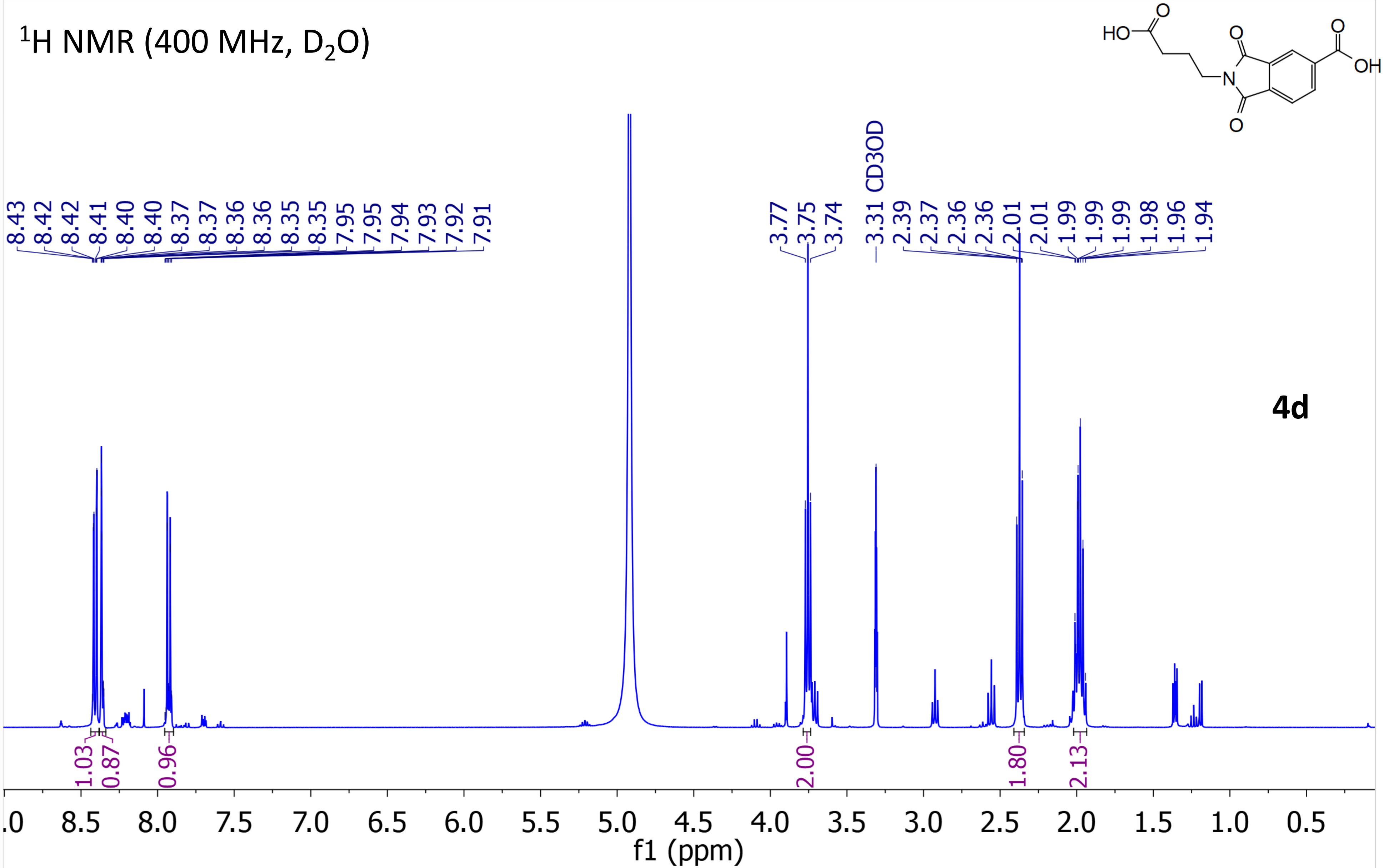

^{1}H NMR (400 MHz, Methanol-d_4) δ 8.41 (dd, J = 7.7, 1.4 Hz, 1H), 8.36 (m, 1H), 7.95 – 7.90 (m, 1H), 3.75 (t, J = 6.8 Hz, 2H), 2.37 (t, J = 7.2 Hz, 2H), 1.99 (p, 2H).

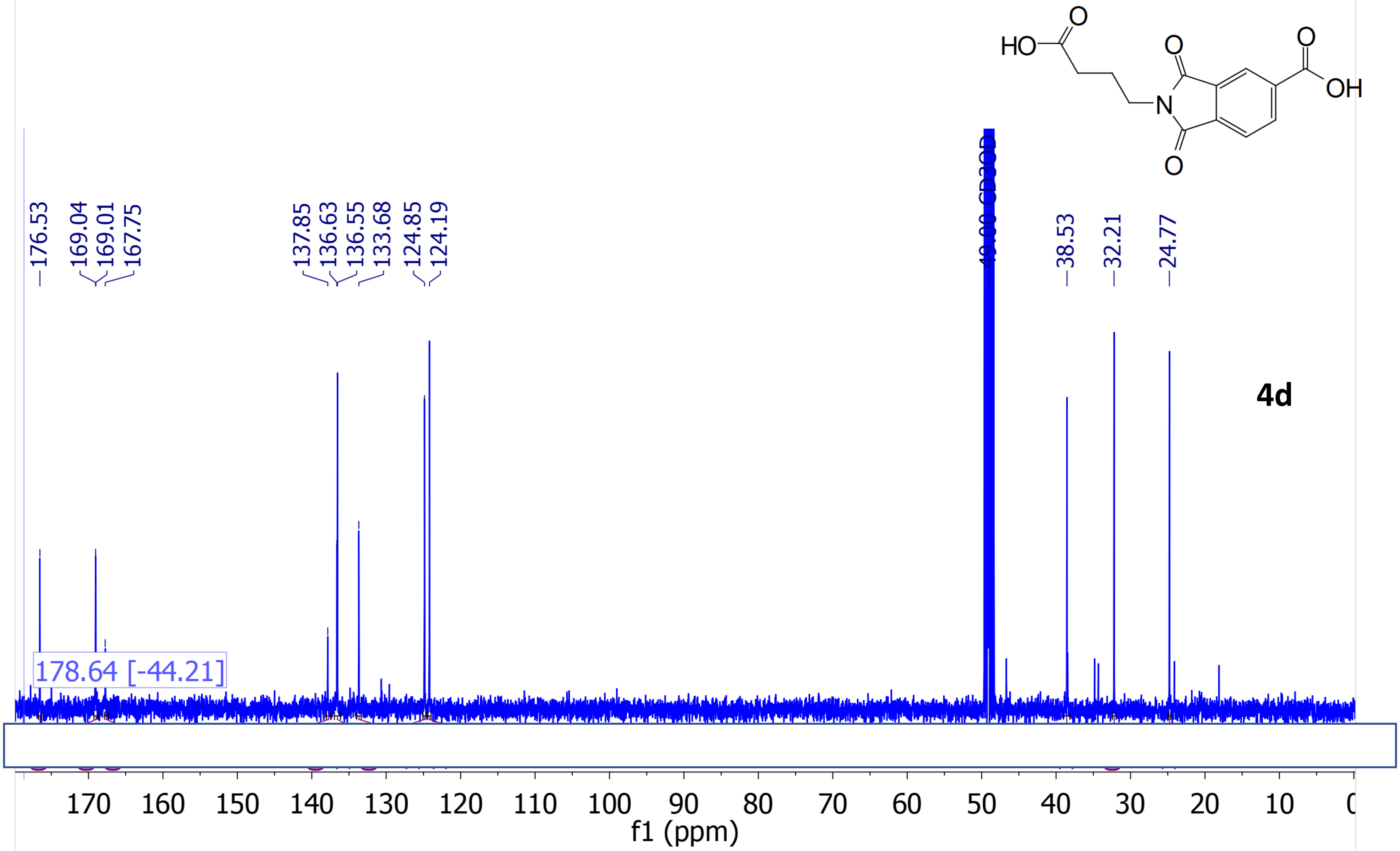

^{13}C NMR (101 MHz, MeOD) δ 176.53, 169.04, 169.01, 167.75, 137.85, 136.63, 136.55, 133.68, 124.85, 124.19, 38.53, 32.21, 24.77.

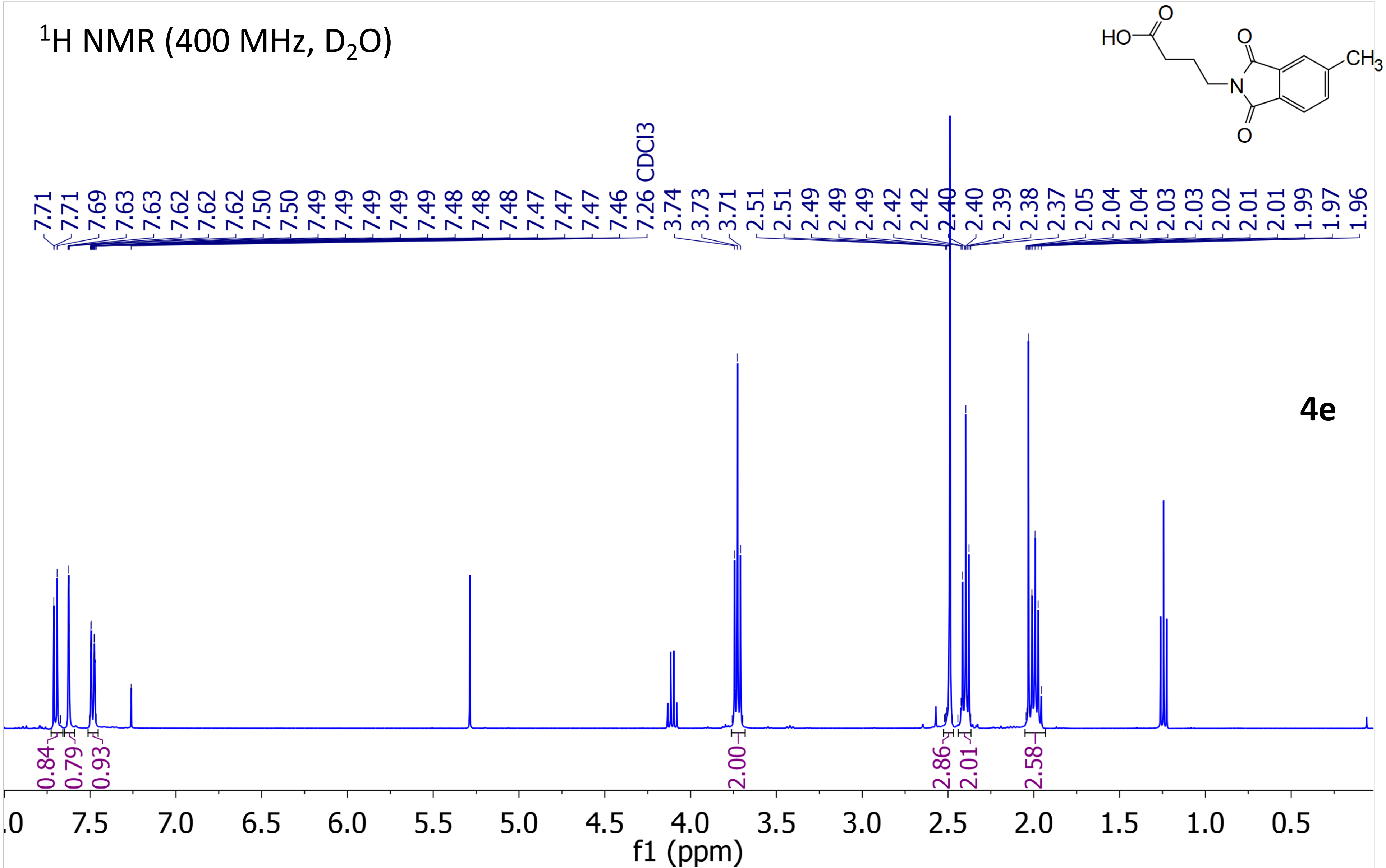

^{1}H NMR (400 MHz, Chloroform-*d*) δ 7.70 (d, $J = 7.7$ Hz, 1H), 7.62 (dt, $J = 1.5, 0.7$ Hz, 1H), 7.48 (ddq, $J = 7.5, 1.4, 0.7$ Hz, 1H), 3.73 (t, $J = 6.8$ Hz, 2H), 2.49 (d, $J = 0.7$ Hz, 3H), 2.40 (t, $J = 7.5$ Hz, 2H), 2.05 – 1.93 (m, 3H).

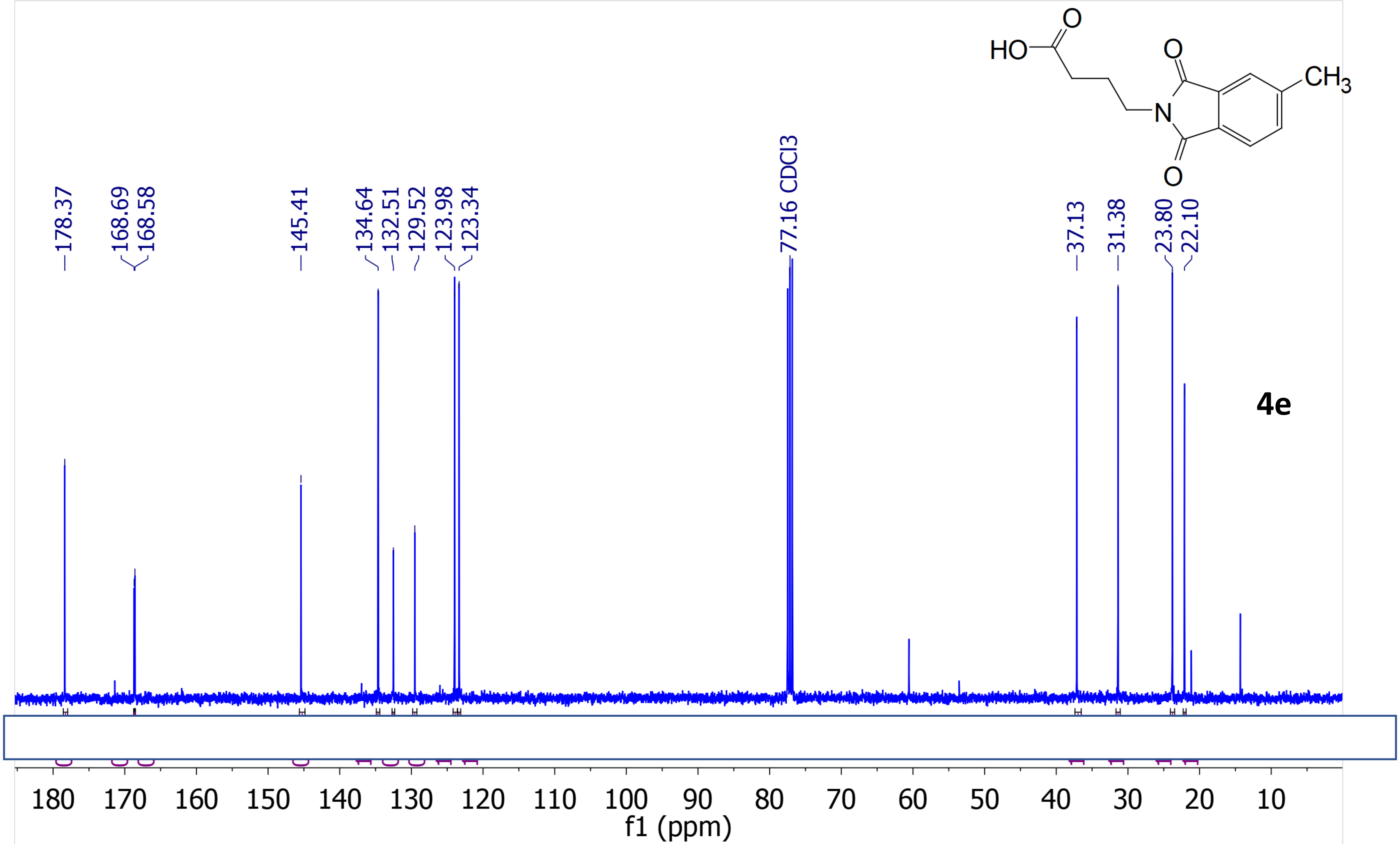

^{13}C NMR (101 MHz, $CDCl_3$) δ 178.37, 168.69, 168.58, 145.41, 134.64, 132.51, 129.52, 123.98, 123.34, 37.13, 31.38, 23.80, 22.10.

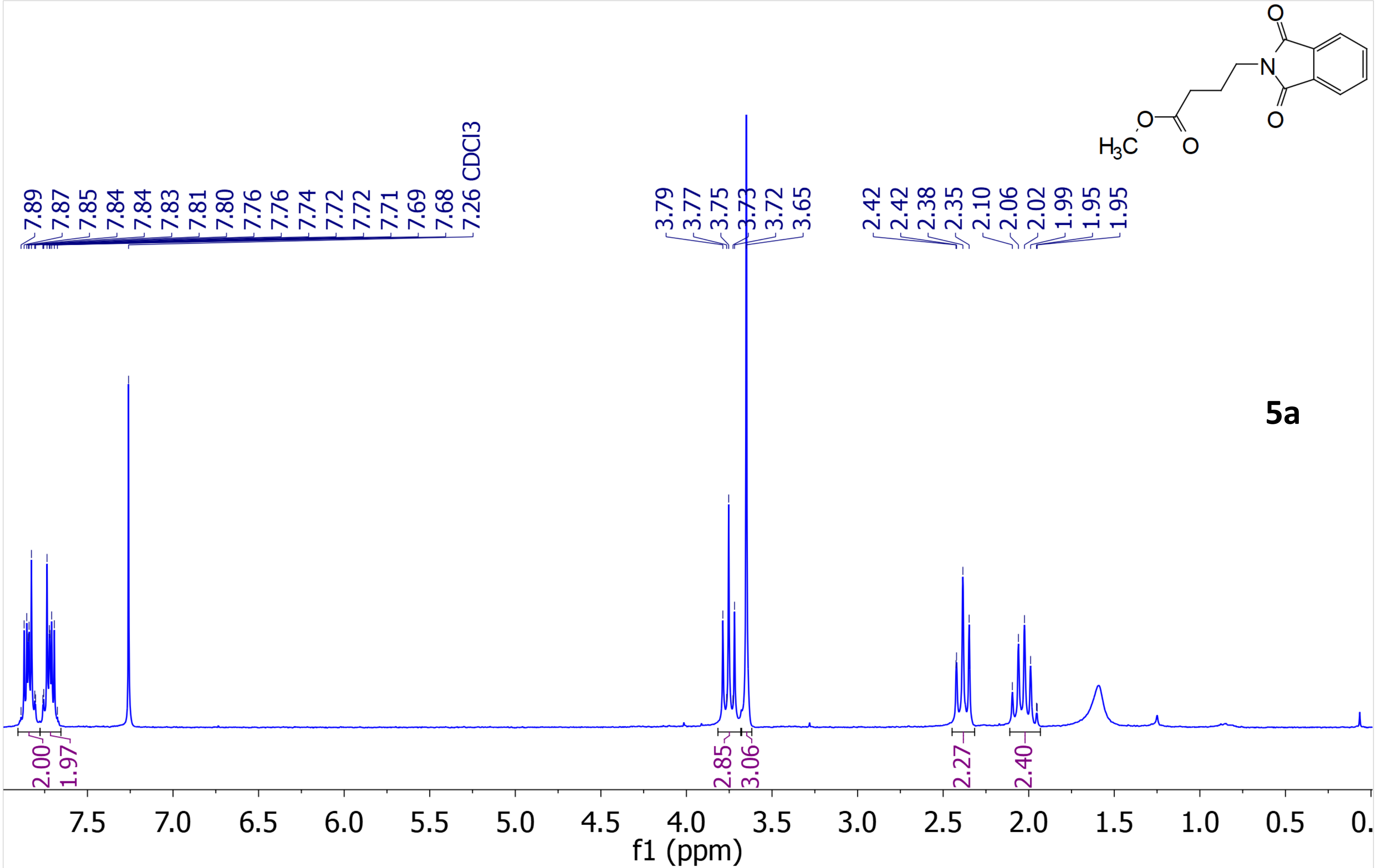

^{1}H NMR (200 MHz, Chloroform-*d*) δ 7.91 – 7.79 (m, 2H), 7.79 – 7.66 (m, 2H), 3.76 (t, J = 6.8 Hz, 2H), 3.66 (s, 3H), 2.39 (t, J = 7.4 Hz, 2H), 2.03 (p, J = 7.0 Hz, 2H).

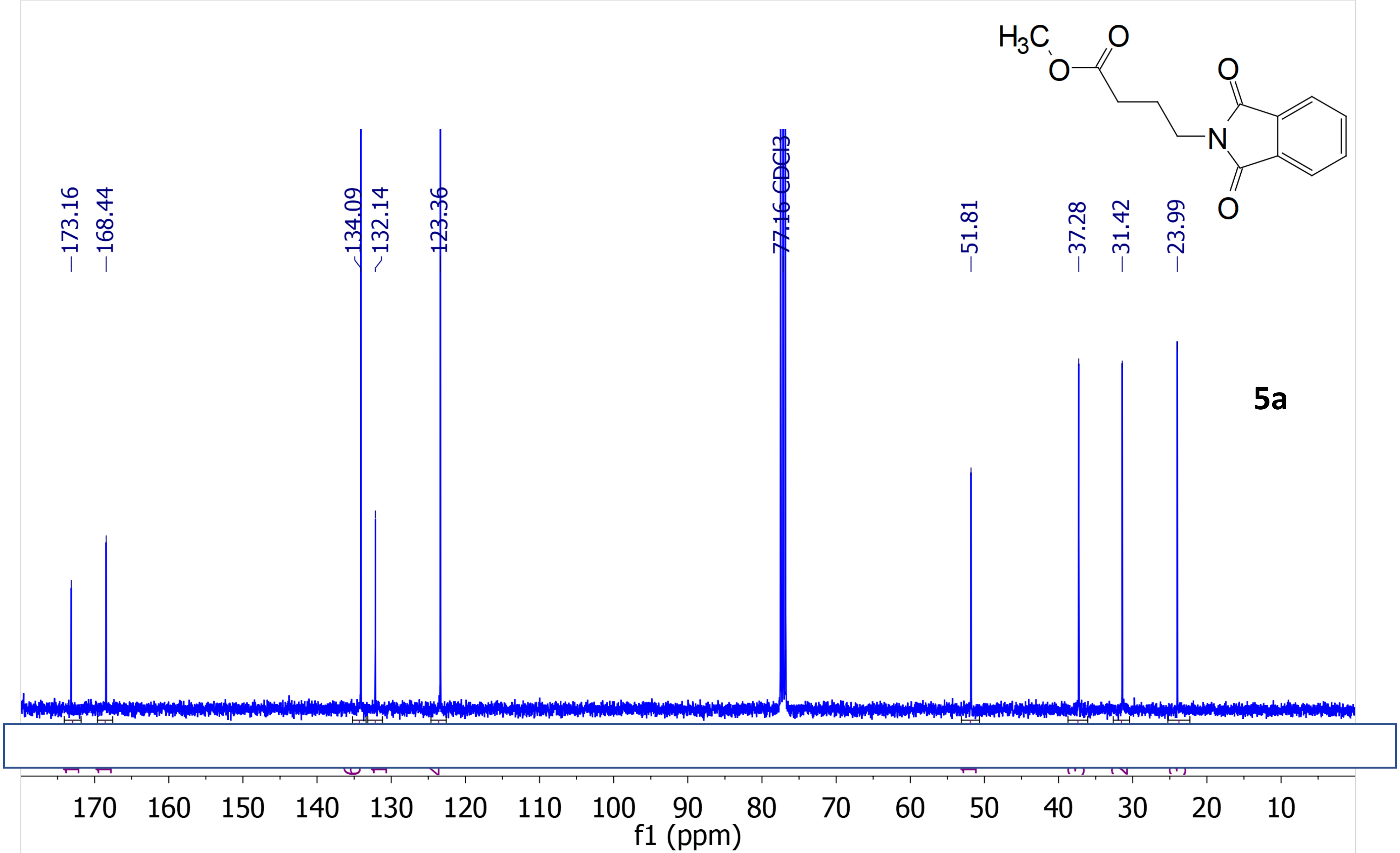

^{13}C NMR (101 MHz, $CDCl_3$) δ 173.48, 168.76, 134.41, 132.46, 123.68, 52.13, 37.60, 31.74, 24.31.

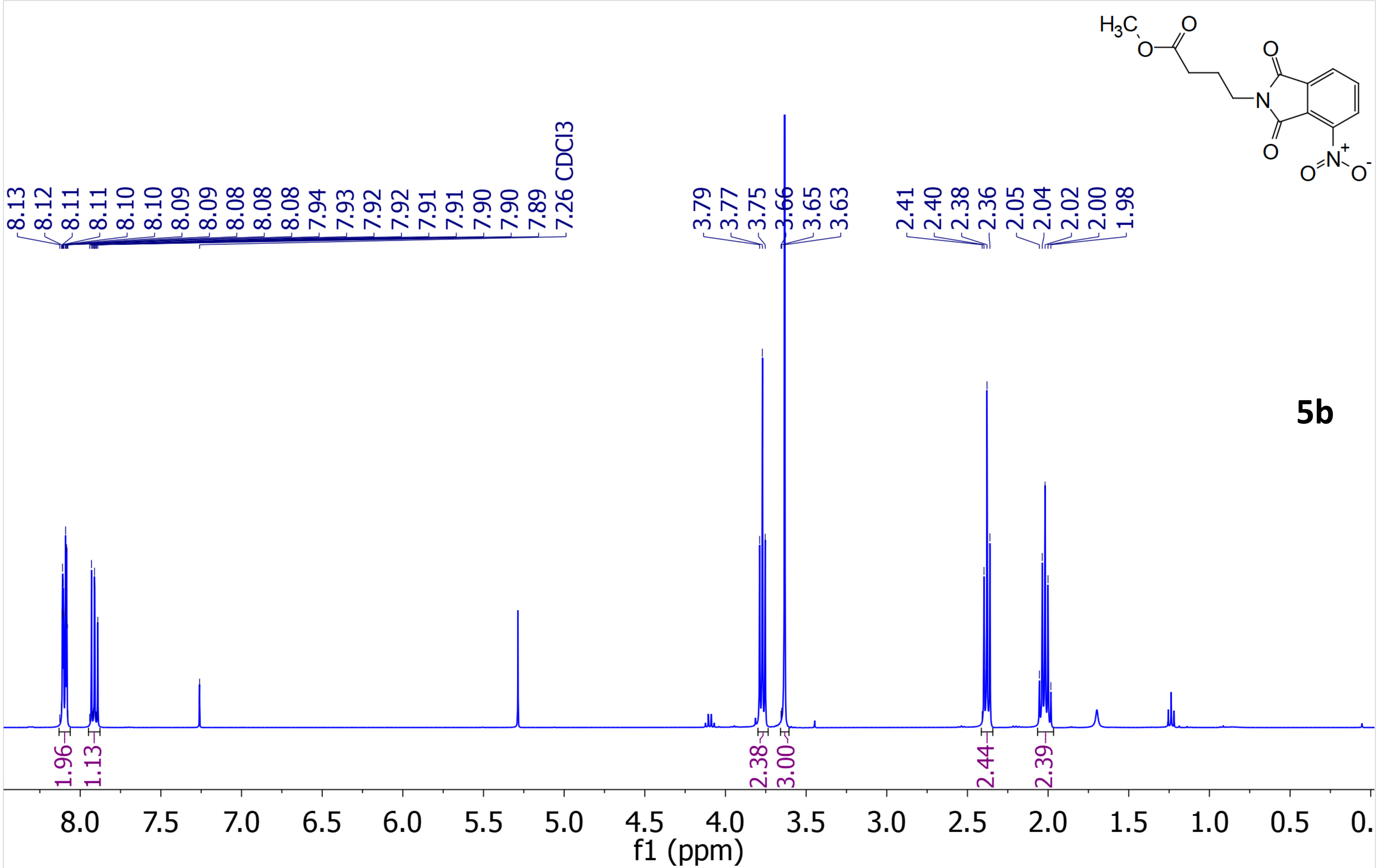

^{1}H NMR (400 MHz, Chloroform-*d*) δ 8.13 – 8.06 (m, 2H), 7.91 (dd, *J* = 8.2, 7.3 Hz, 1H), 3.77 (t, *J* = 6.9 Hz, 2H), 3.63 (s, 3H), 2.38 (t, *J* = 7.3 Hz, 2H), 2.02 (p, *J* = 7.1 Hz, 2H).

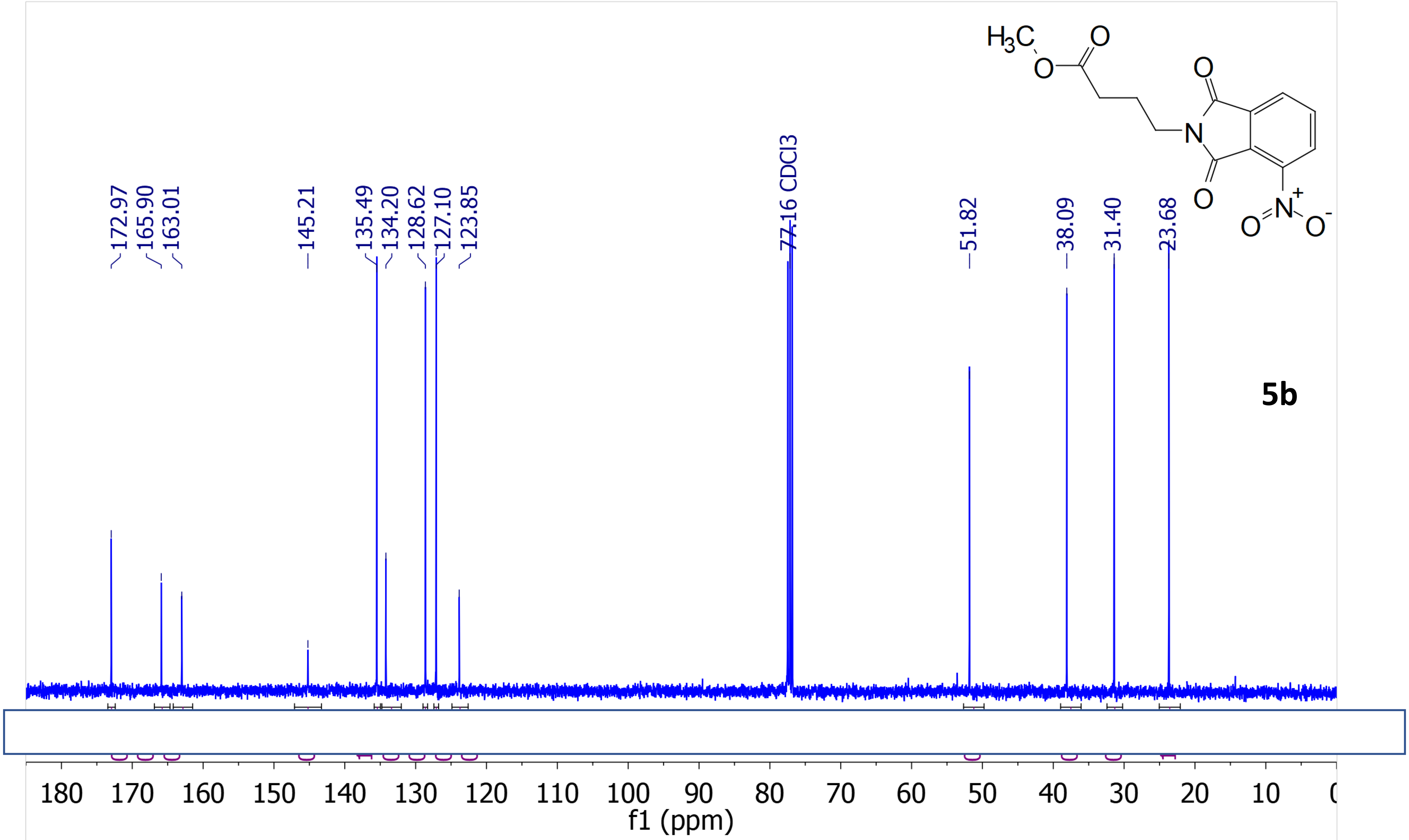

^{13}C NMR (101 MHz, $CDCl_3$) δ 172.97, 165.90, 163.01, 145.21, 135.49, 134.20, 128.62, 127.10, 123.85, 51.82, 38.09, 31.40, 23.68.

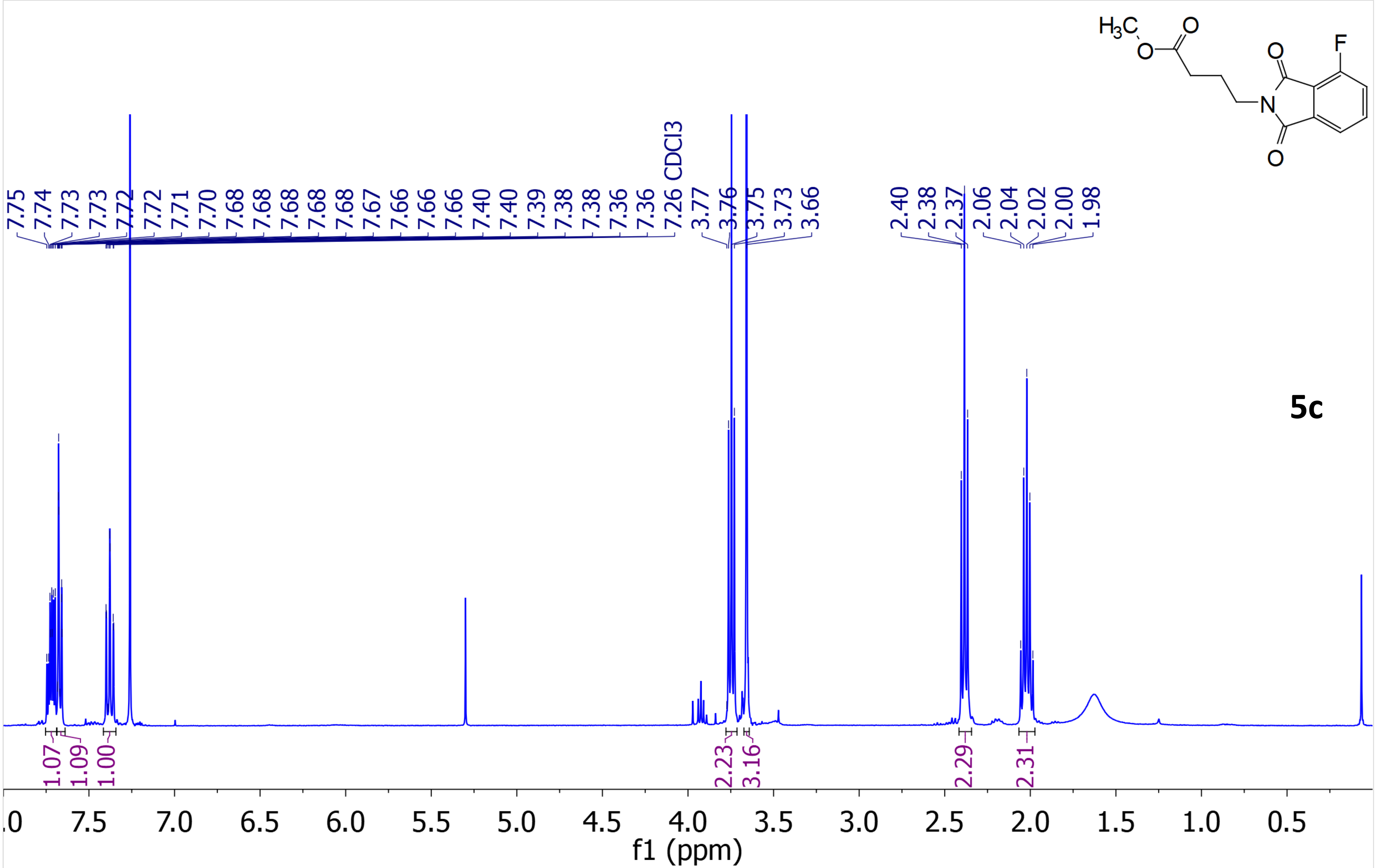

^{1}H NMR (400 MHz, Chloroform-*d*) δ 7.72 (ddd, *J* = 8.1, 7.4, 4.3 Hz, 1H), 7.67 (d, *J* = 7.3 Hz, 1H), 7.38 (td, *J* = 8.5, 1.0 Hz, 1H), 3.75 (t, *J* = 6.8 Hz, 2H), 3.66 (s, 3H), 2.38 (t, *J* = 7.4 Hz, 2H), 2.02 (p, *J* = 7.1 Hz, 2H).

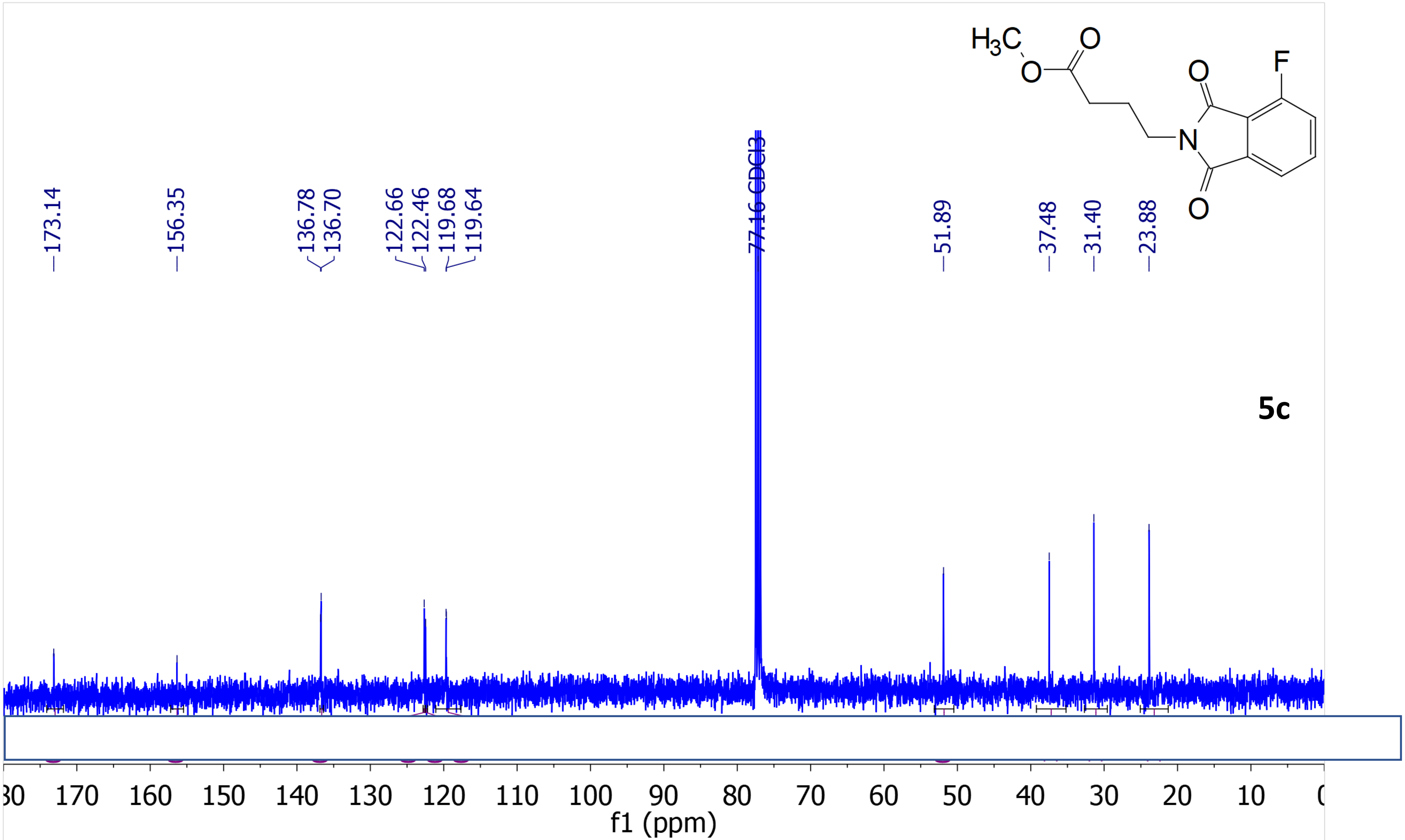

^{13}C NMR (101 MHz, $CDCl_3$) δ 173.14, 156.35, 136.78, 136.70, 122.66, 122.46, 119.68, 119.64, 51.89, 37.48, 31.40, 23.88.

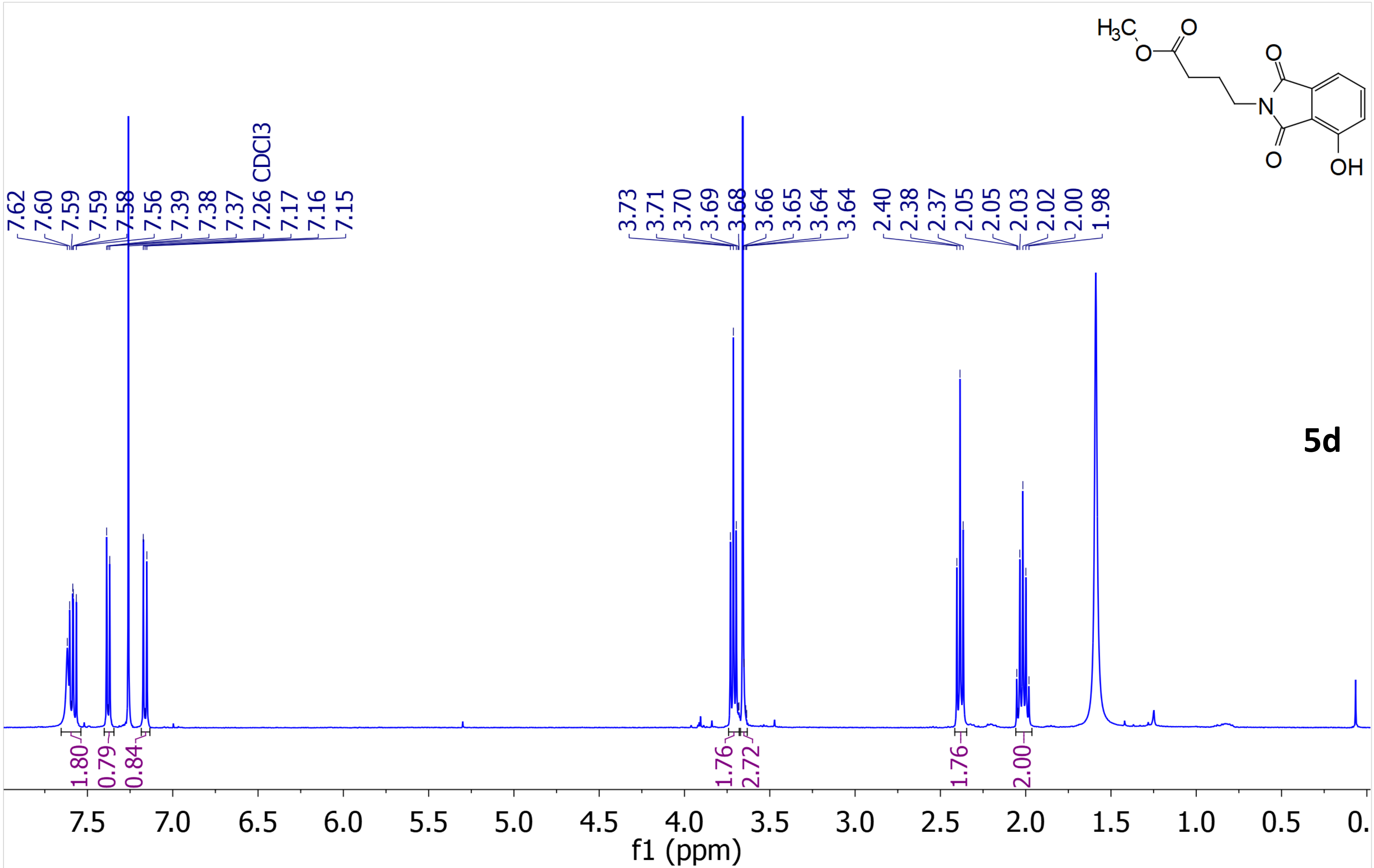

^{1}H NMR (400 MHz, Chloroform-*d*) δ 7.65 – 7.54 (m, 2H), 7.38 (d, *J* = 7.2 Hz, 1H), 7.16 (d, *J* = 8.4 Hz, 1H), 3.71 (t, *J* = 6.9 Hz, 2H), 3.66 (s, 3H), 2.38 (t, *J* = 7.4 Hz, 2H), 2.02 (p , *J* = 7.1 Hz, 2H).

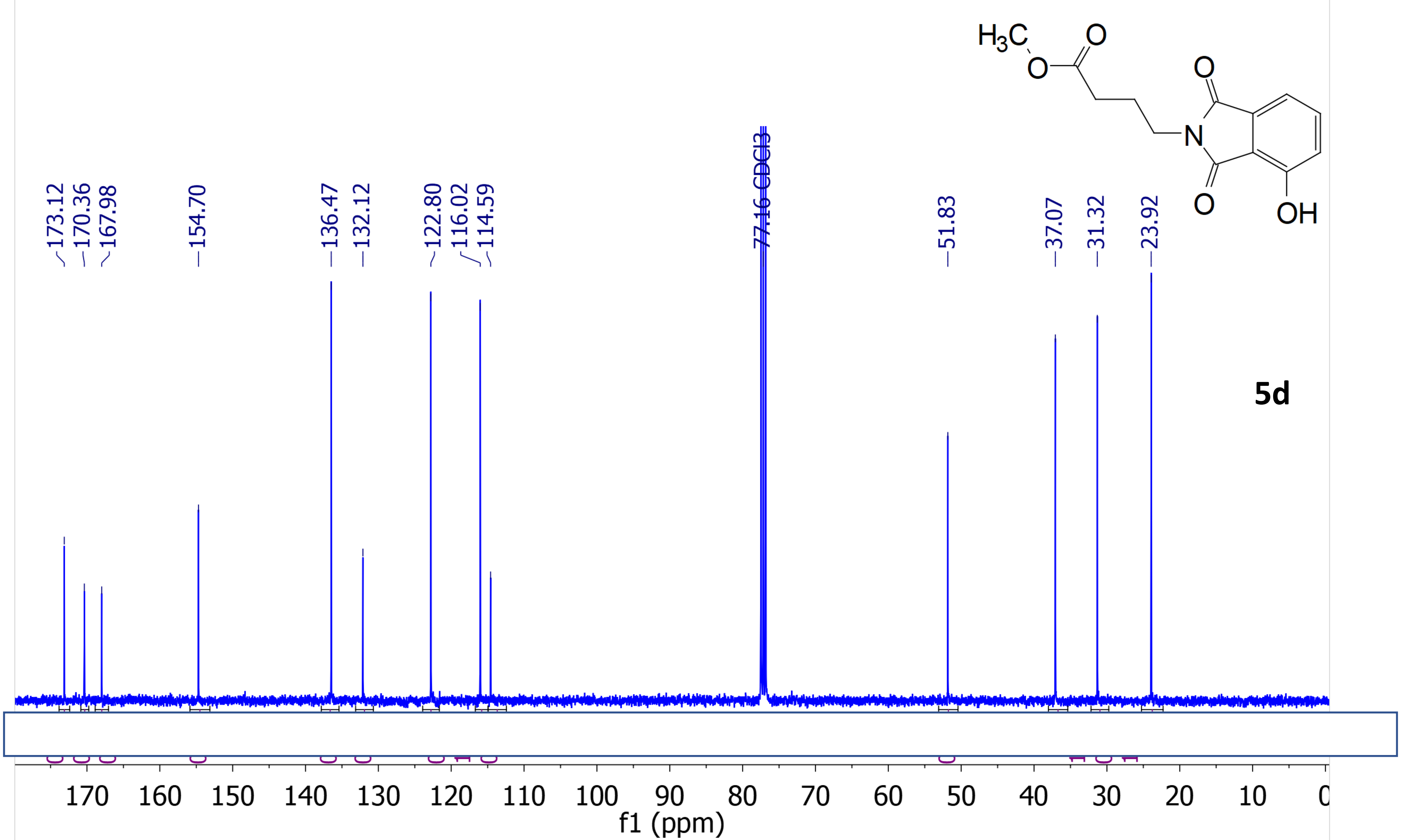

^{13}C NMR (101 MHz, $CDCl_3$) δ 173.12, 170.36, 167.98, 154.70, 136.47, 132.12, 122.80, 116.02, 114.59, 51.83, 37.07, 31.32, 23.92.

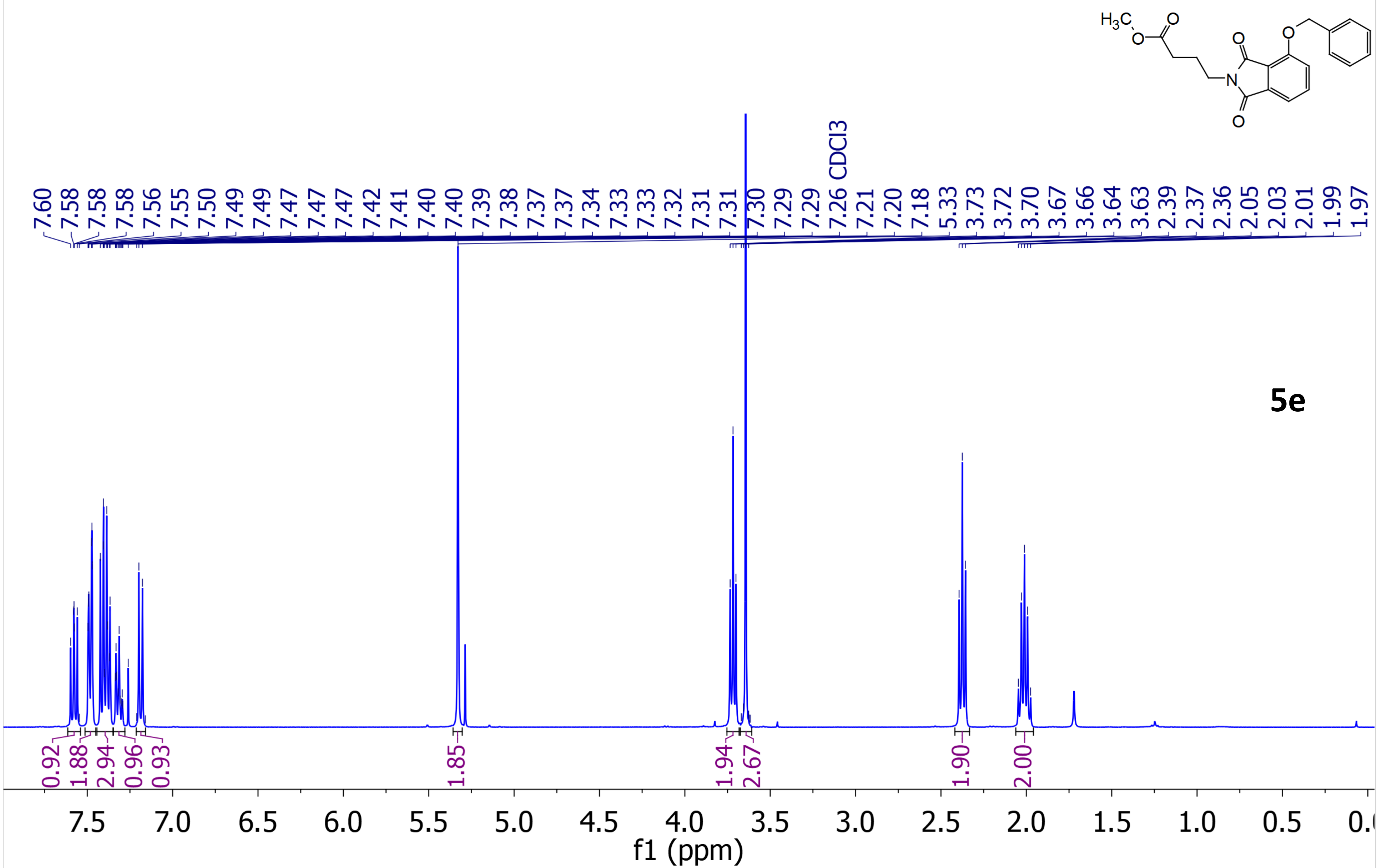

^{1}H NMR (400 MHz, Chloroform-*d*) δ 7.58 (t, 1H), 7.51 – 7.45 (m, 2H), 7.44 – 7.35 (m, 3H), 7.35 – 7.28 (m, 1H), 7.19 (d, J = 8.4 Hz, 1H), 5.33 (s, 2H), 3.72 (t, J = 6.8 Hz, 2H), 3.64 (s, 3H), 2.37 (t, J = 7.5 Hz, 2H), 2.01 (p, J = 7.1 Hz, 2H).

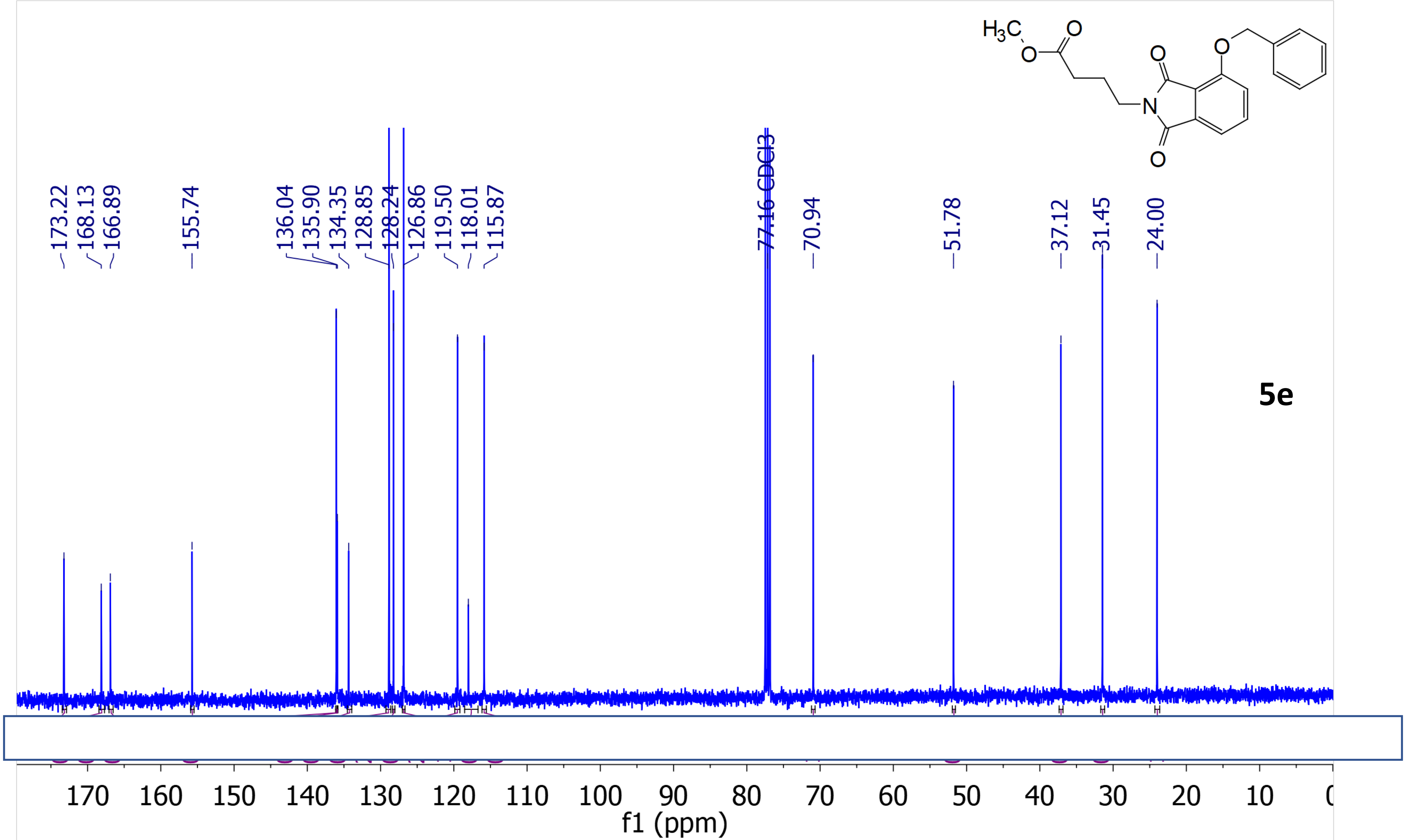

^{13}C NMR (101 MHz, $CDCl_3$) δ 173.22, 168.13, 166.89, 155.74, 136.04, 135.90, 134.35, 128.85, 128.24, 126.86, 119.50, 118.01, 115.87, 70.94, 51.78, 37.12, 31.45, 24.00.

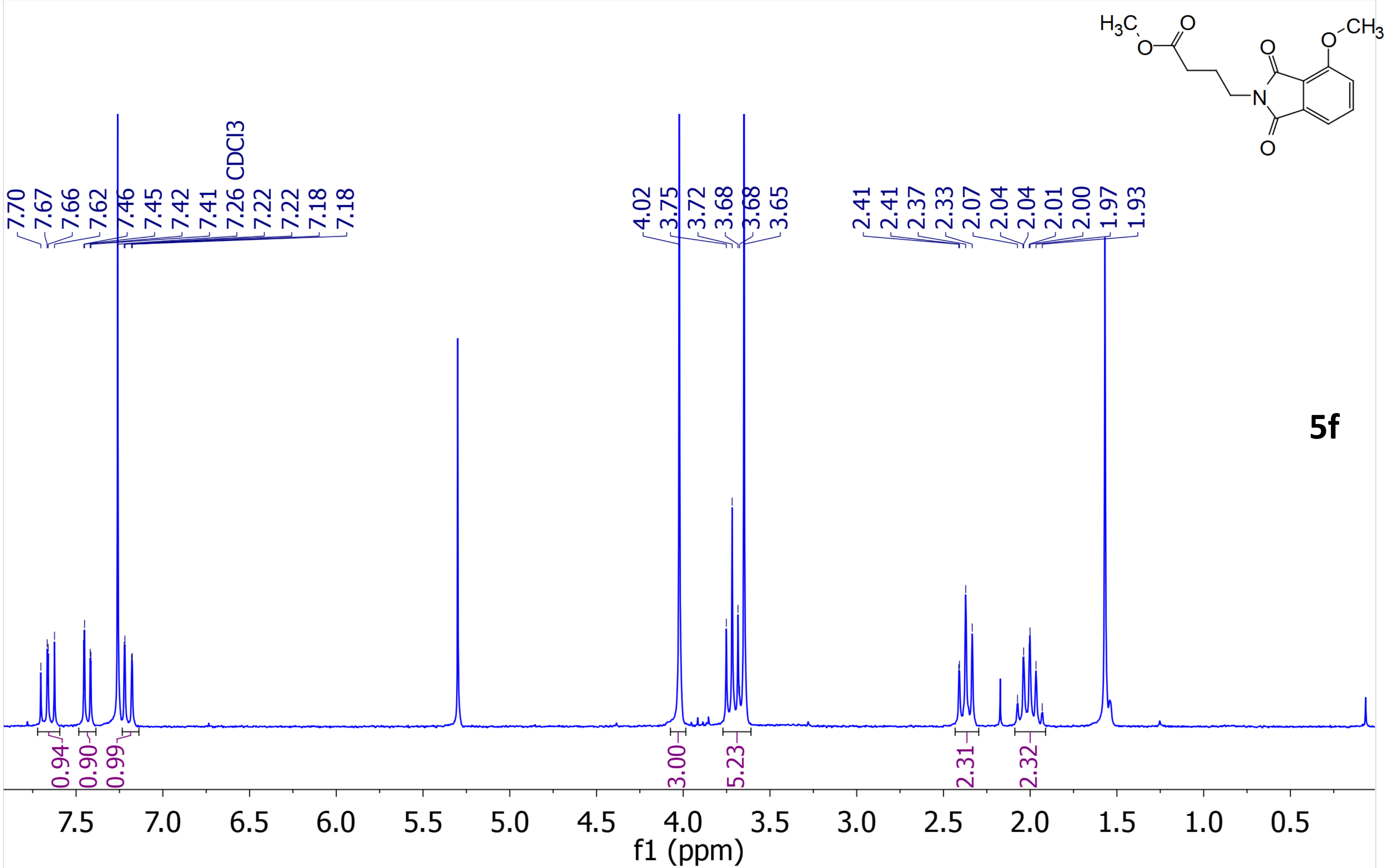

^{1}H NMR (200 MHz, Chloroform-*d*) δ 7.67 (dd, *J* = 8.4, 7.3 Hz, 1H), 7.44 (dd, *J* = 7.3, 0.8 Hz, 1H), 7.21 (dd, *J* = 8.5, 0.7 Hz, 1H), 4.03 (s, 3H), 3.78 – 3.62 (m, 5H), 2.38 (t, *J* = 7.4 Hz, 2H), 2.03 (p, 2H).

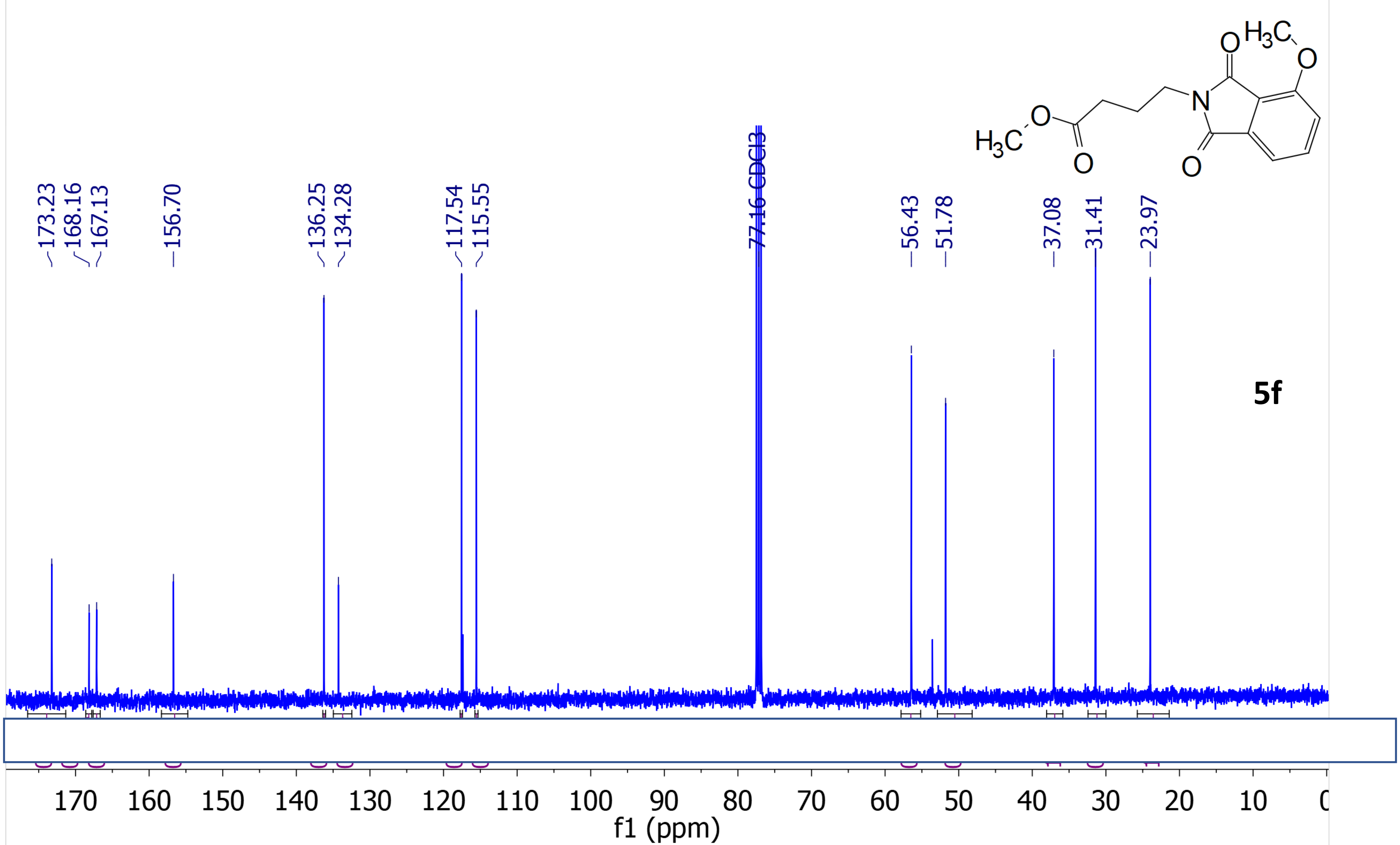

^{13}C NMR (101 MHz, $CDCl_3$) δ 173.55, 168.47, 167.45, 157.02, 136.57, 134.59, 117.86, 115.86, 56.75, 52.10, 37.40, 31.73, 24.29.

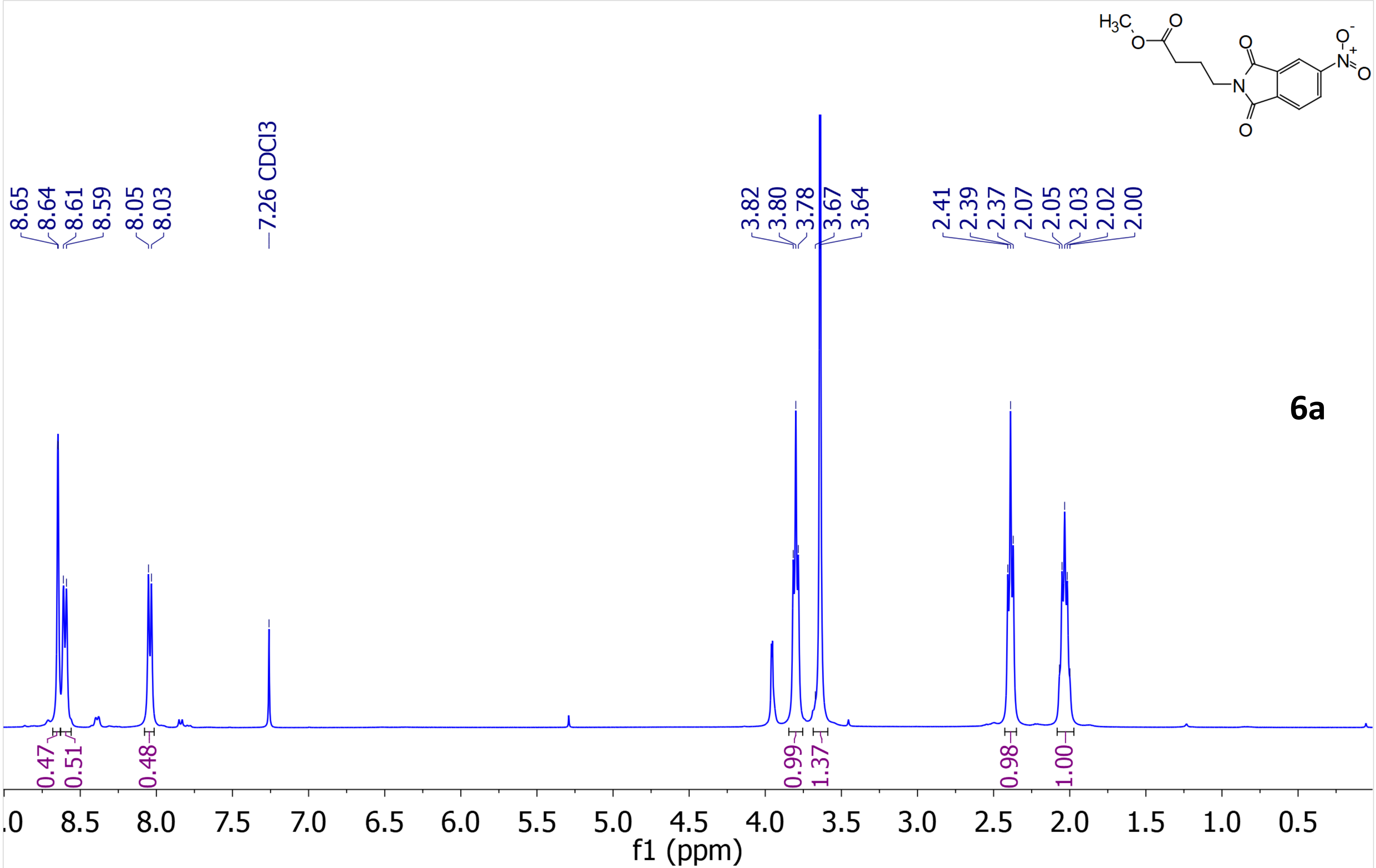

^{1}H NMR (400 MHz, Chloroform-*d*) δ 8.68 – 8.63 (m, 0H), 8.60 (d, *J* = 8.1 Hz, 1H), 8.04 (d, *J* = 7.8 Hz, 0H), 3.80 (t, *J* = 6.7 Hz, 1H), 3.64 (s, 1H), 2.39 (t, *J* = 7.1 Hz, 1H), 2.03 (p, *J* = 6.8 Hz, 1H).

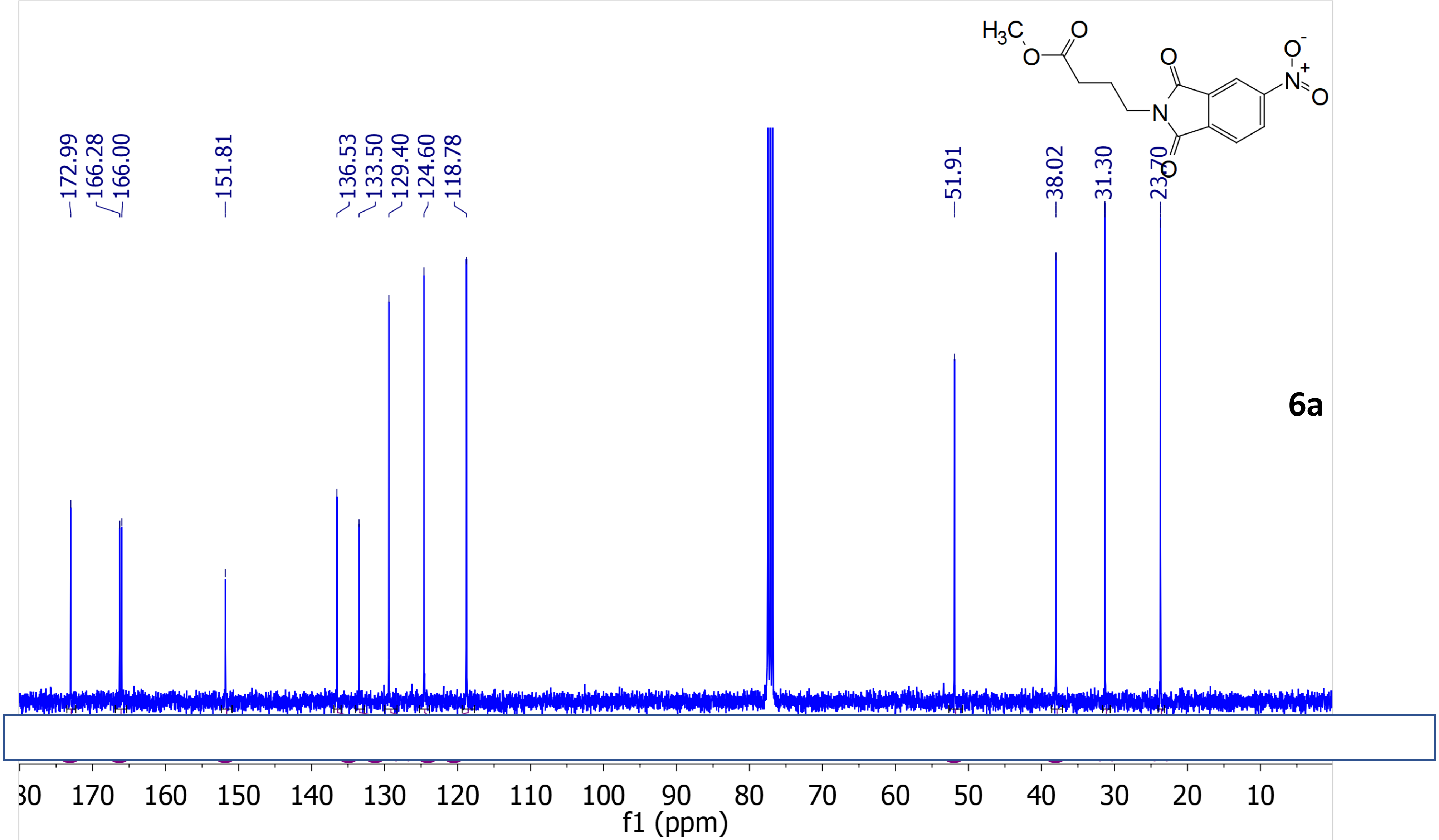

^{13}C NMR (101 MHz, $CDCl_3$) δ 172.99, 166.28, 166.00, 151.81, 136.53, 133.50, 129.40, 124.60, 118.78, 51.91, 38.02, 31.30, 23.70.

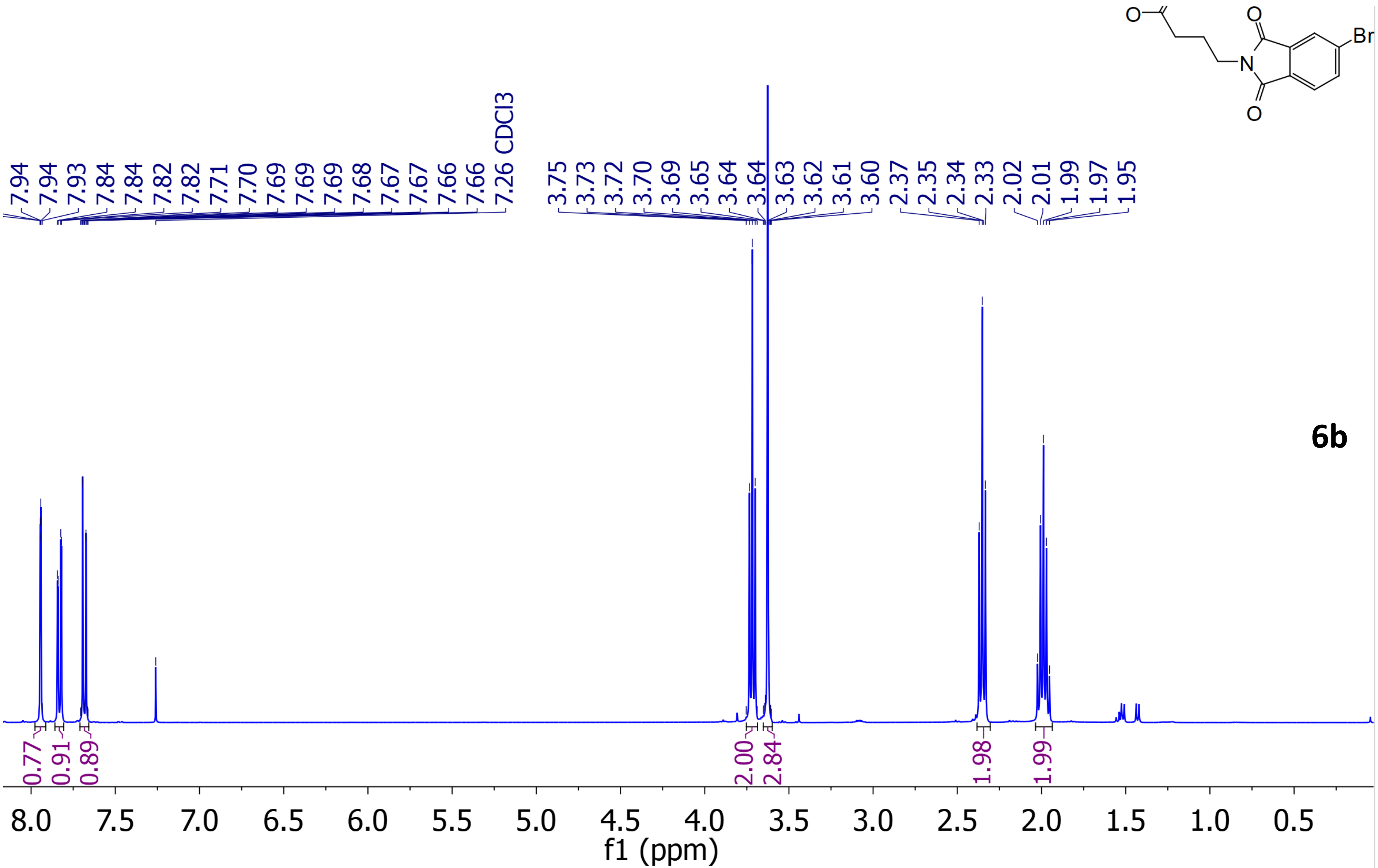

ʀ (400 MHz, Chloroform-*d*) δ 7.94 (dd, *J* = 1.7, 0.6 Hz, 1H), 7.83 (dd, *J* = 7.9, 1.7 Hz, 1H), 7.68 (dd, *J* = 7.9, 0.6 Hz, 1H), 3.72 (t, *J* = 6.9 Hz, 2H), 3.63 (s, 3H), 2.35 (t, *J* = 7.4 Hz, 2H), 1.99 (p, *J* = 7.1 Hz, 2H).

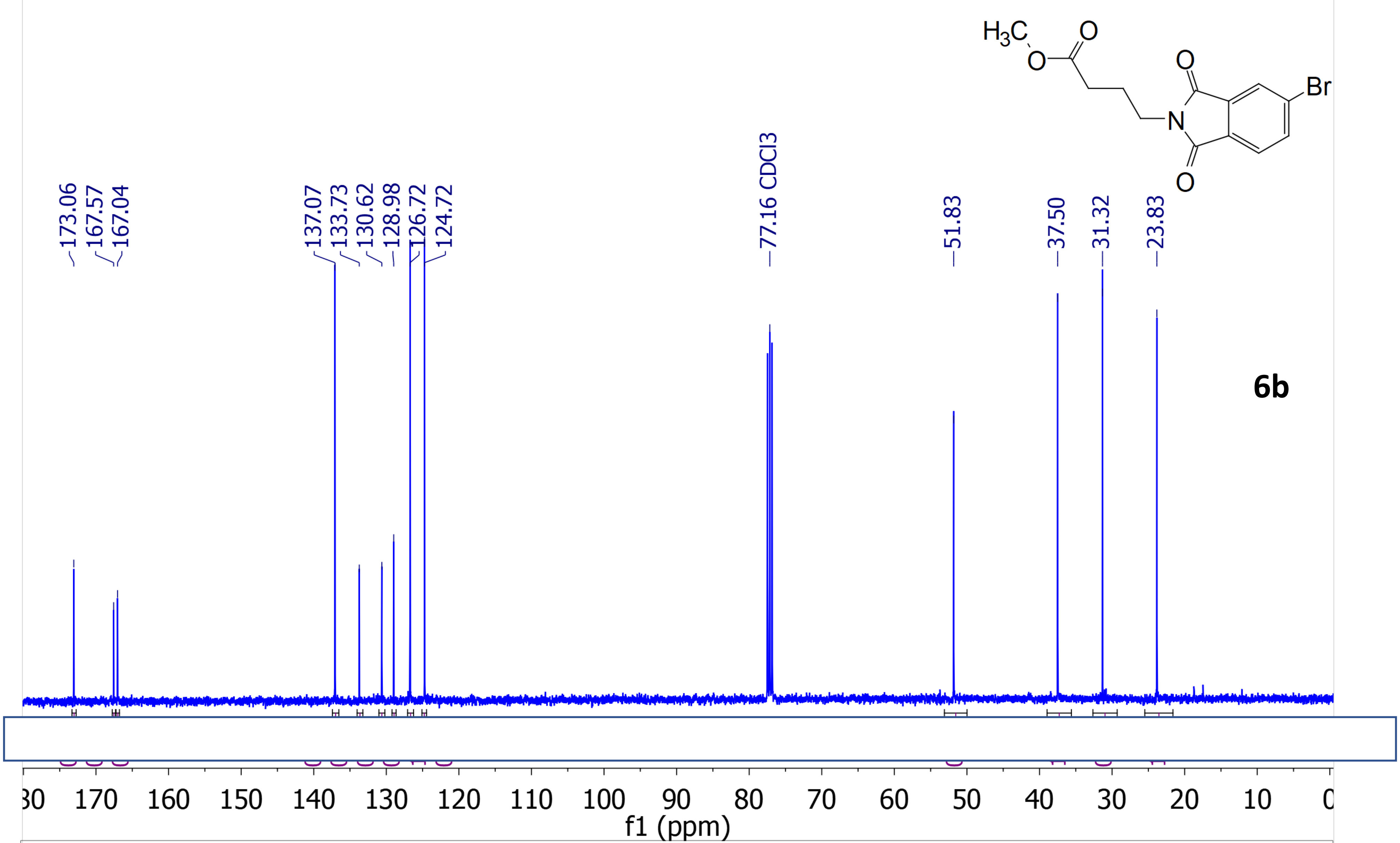

6b

^{13}C NMR (101 MHz, $CDCl_3$) δ 173.06, 167.57, 167.04, 137.07, 133.73, 130.62, 128.98, 126.72, 124.72, 51.83, 37.50, 31.32, 23.83.

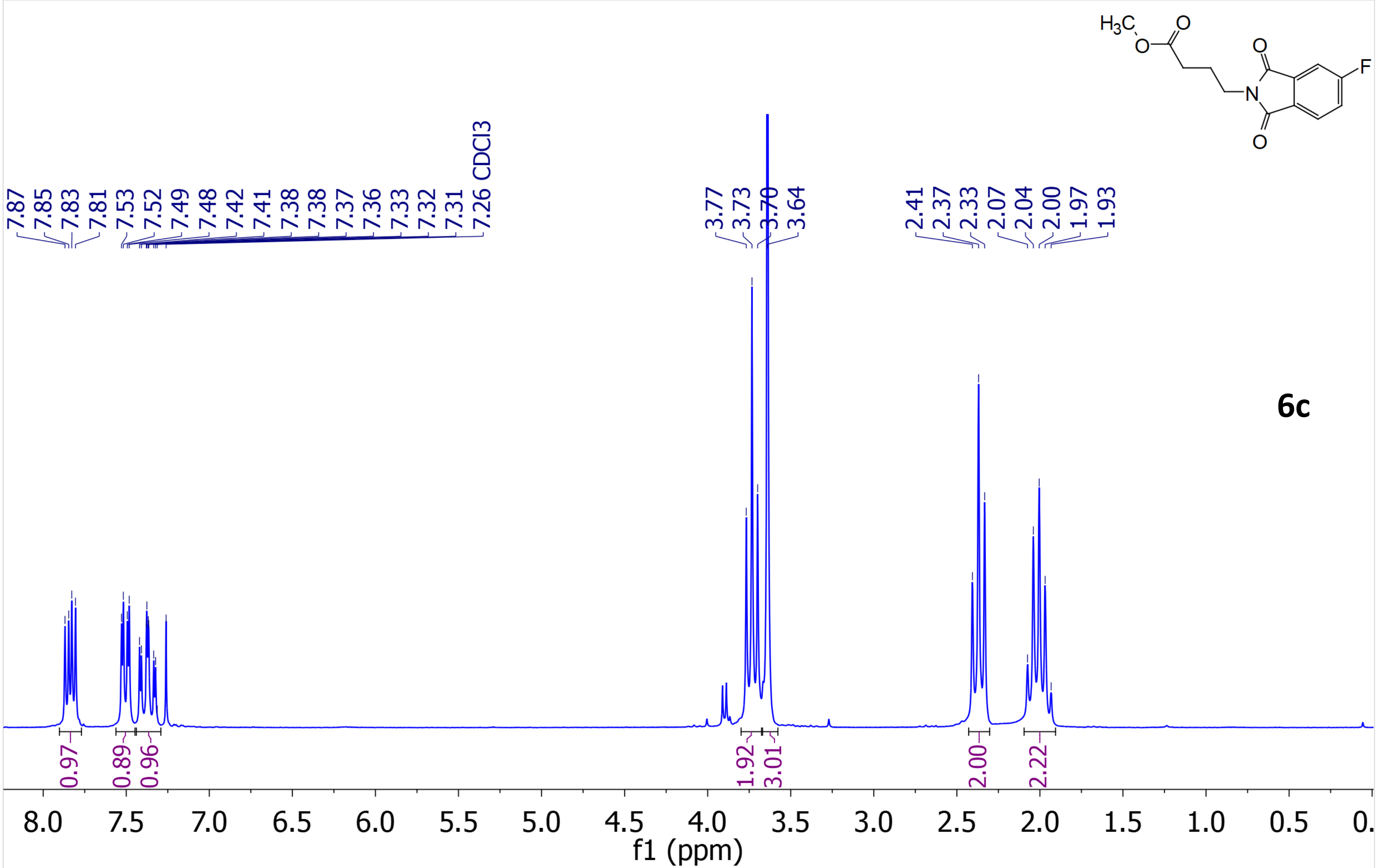

^{1}H NMR (200 MHz, Chloroform-*d*) δ 7.84 (dd, *J* = 8.2, 4.5 Hz, 1H), 7.51 (dd, *J* = 7.0, 2.3 Hz, 1H), 7.37 (td, 1H), 3.73 (t, *J* = 6.8 Hz, 2H), 3.64 (s, 3H), 2.37 (t, *J* = 7.4 Hz, 2H), 2.00 (p, *J* = 7.1 Hz, 2H).

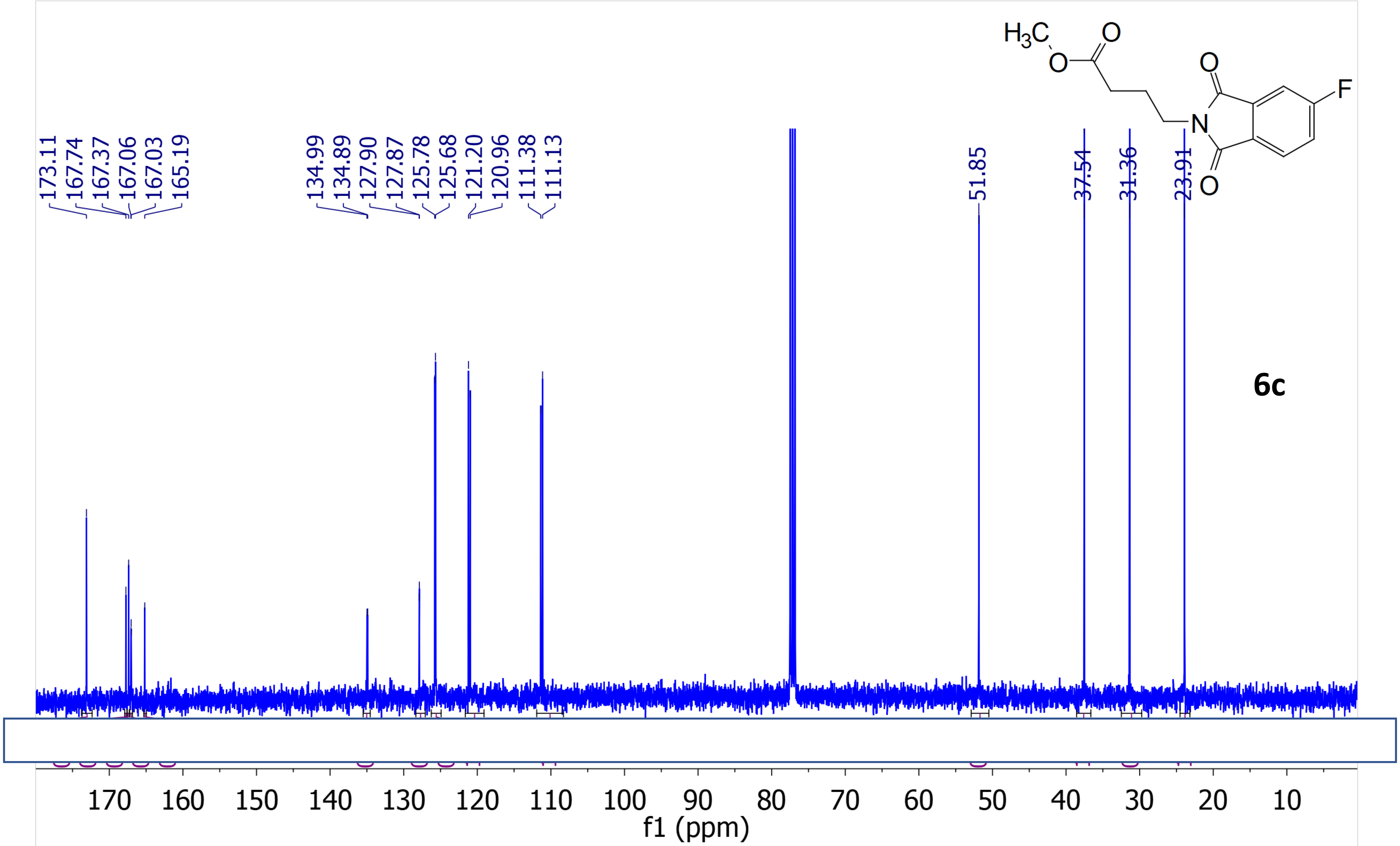

^{13}C NMR (101 MHz, Chloroform-*d*) δ 173.11, 167.74 (d, J = 256.5 Hz), 167.37, 167.05 (d, J = 3.1 Hz), 165.19, 134.94 (d, J = 9.5 Hz), 127.89 (d, J = 3.1 Hz), 125.73 (d, J = 9.5 Hz), 121.08 (d, J = 23.7 Hz), 111.25 (d, J = 24.8 Hz), 51.85, 37.54, 31.36, 23.91.

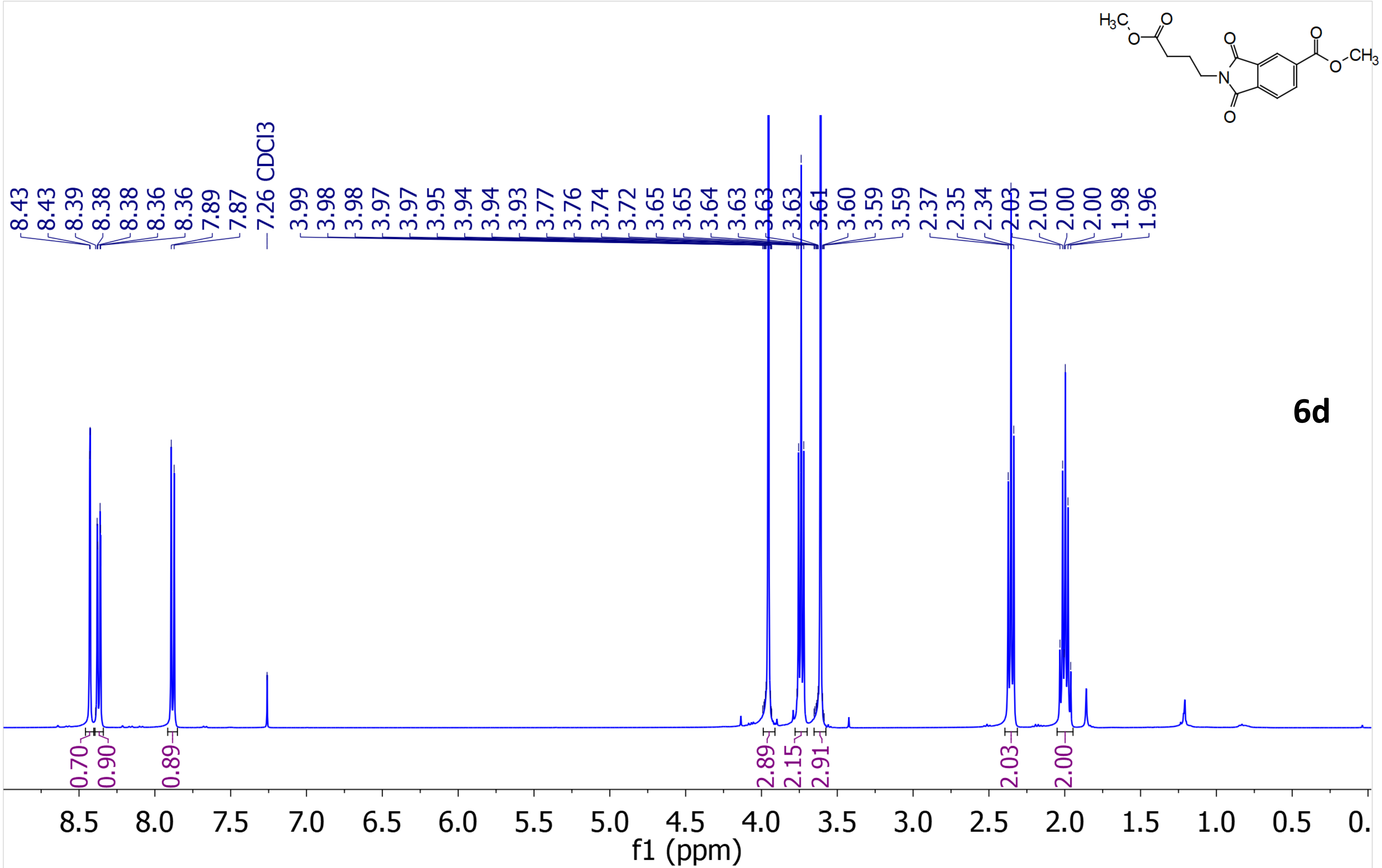

^{1}H NMR (400 MHz, Chloroform-*d*) δ 8.43 (d, *J* = 1.2 Hz, 1H), 8.37 (dd, *J* = 7.8, 1.4 Hz, 1H), 7.88 (d, *J* = 7.8 Hz, 1H), 3.95 (s, 3H), 3.74 (t, *J* = 6.9 Hz, 2H), 3.61 (s, 3H), 2.35 (t, *J* = 7.3 Hz, 2H), 2.00 (p, *J* = 7.1 Hz, 2H).

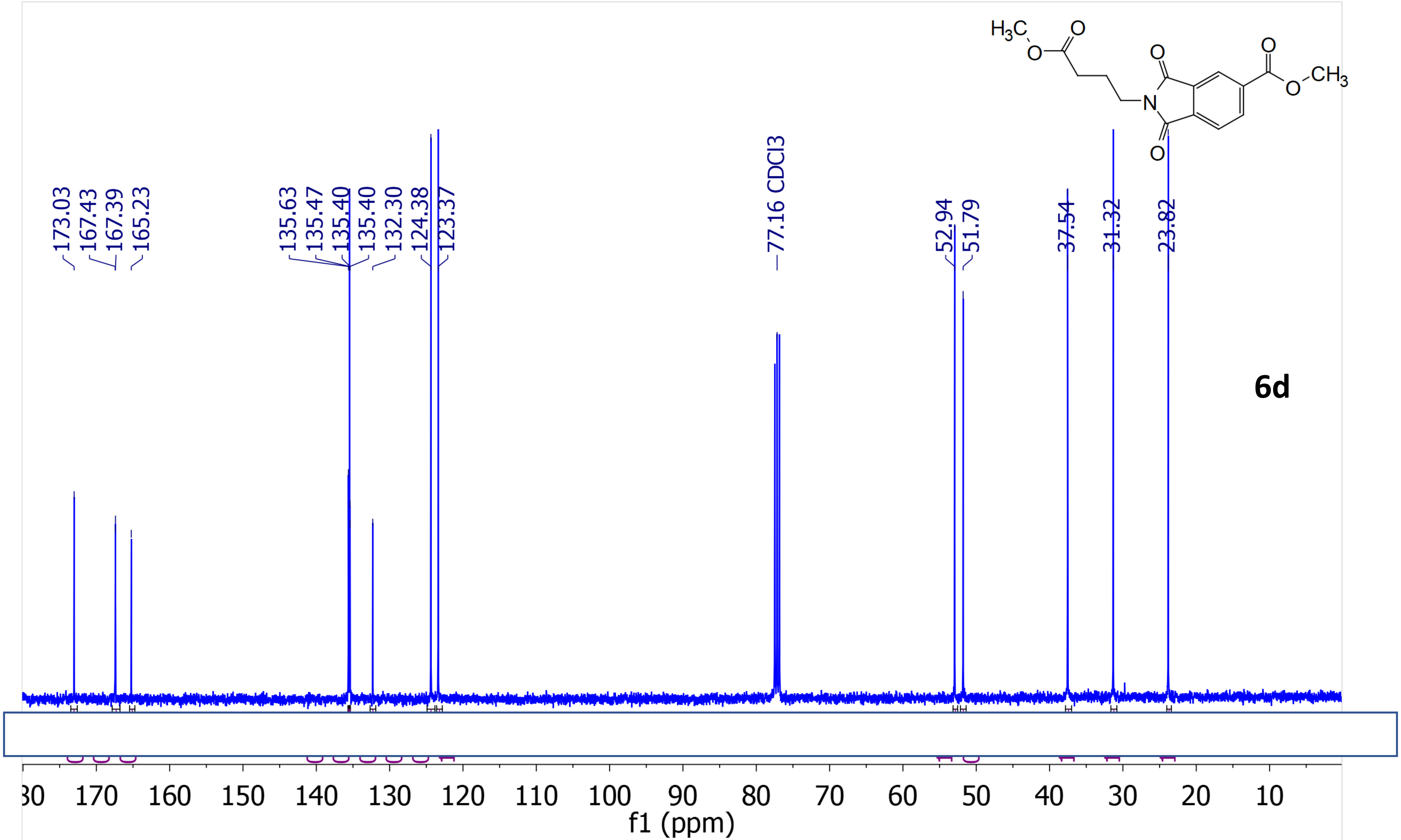

^{13}C NMR (101 MHz, $CDCl_3$) δ 173.03, 167.43, 167.39, 165.23, 135.63, 135.47, 135.40, 135.40, 132.30, 124.38, 123.37, 52.94, 51.79, 37.54, 31.32, 23.82.

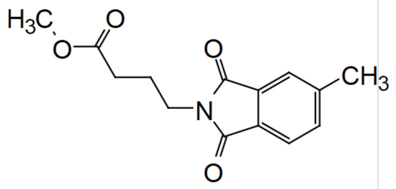

6e

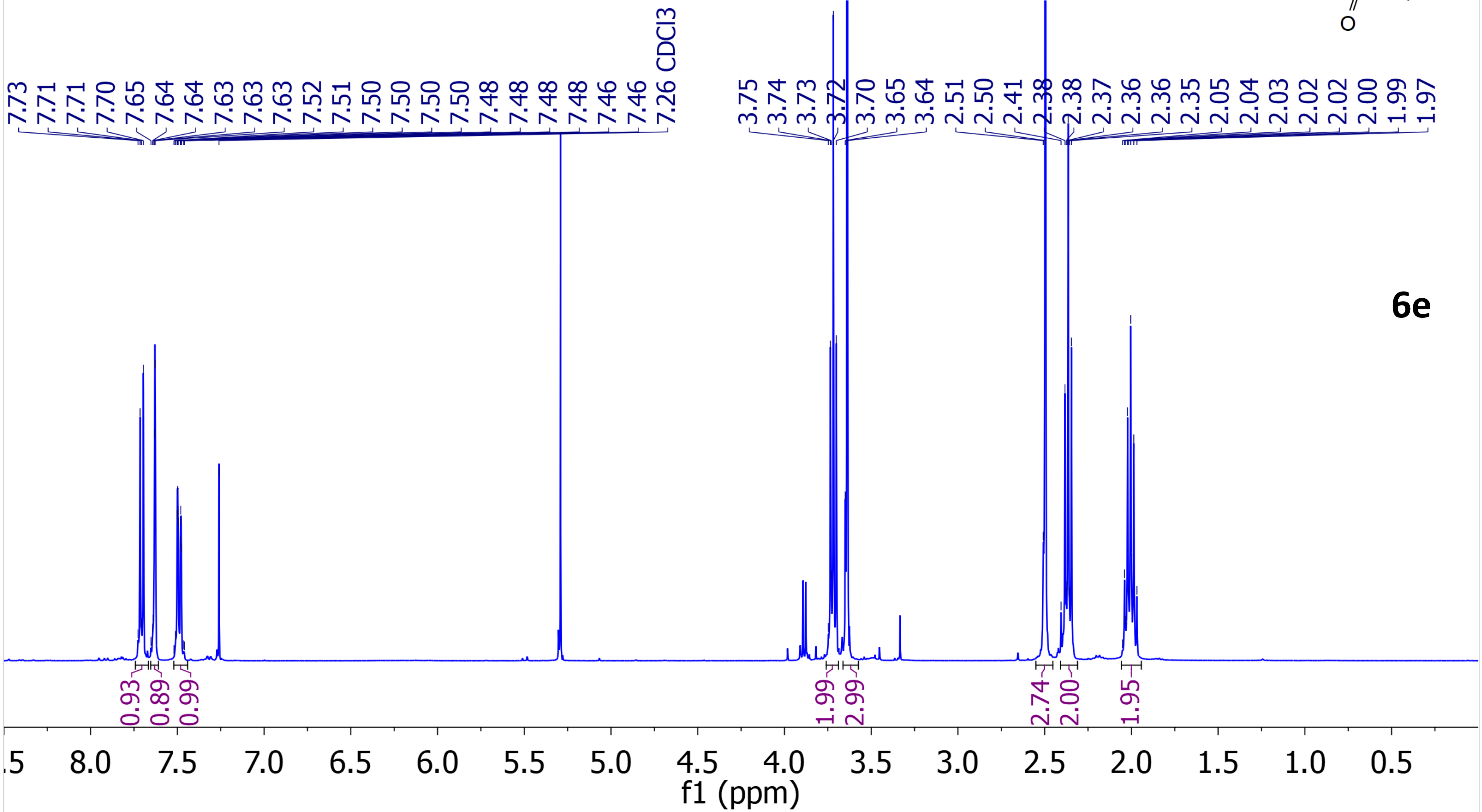

^{1}H NMR (400 MHz, Chloroform-*d*) δ 7.73 (d, *J* = 7.6 Hz, 1H), 7.68 – 7.63 (m, 1H), 7.51 (d, *J* = 7.2 Hz, 1H), 3.74 (t, *J* = 6.8 Hz, 2H), 3.66 (s, 3H), 2.52 (s, 3H), 2.39 (t, *J* = 7.5 Hz, 2H), 2.03 (p, *J* = 7.1 Hz, 2H).

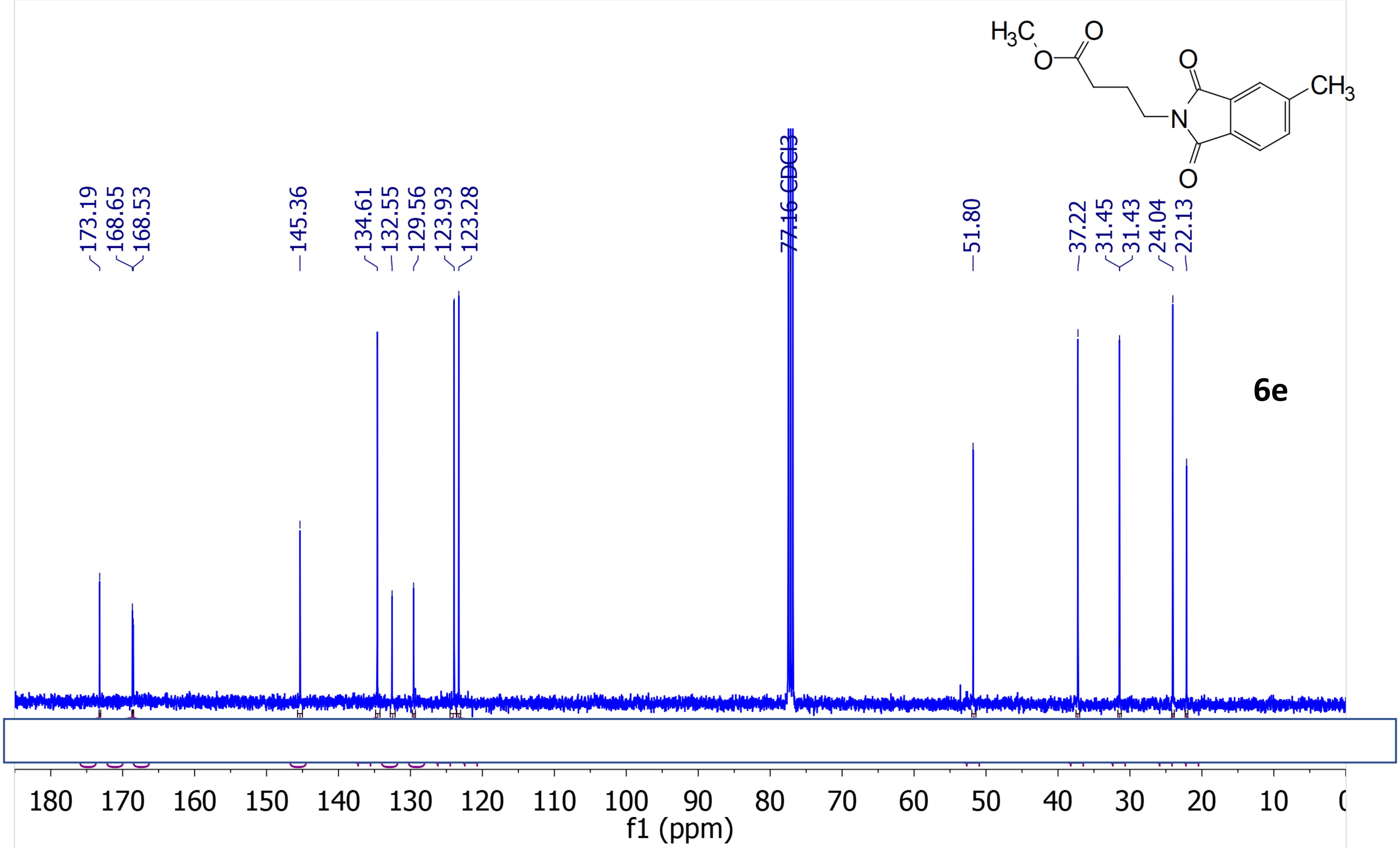

^{13}C NMR (101 MHz, $CDCl_3$) δ 173.19, 168.65, 168.53, 145.36, 134.61, 132.55, 129.56, 123.93, 123.28, 51.80, 37.22, 31.45, 31.43, 24.04, 22.13.

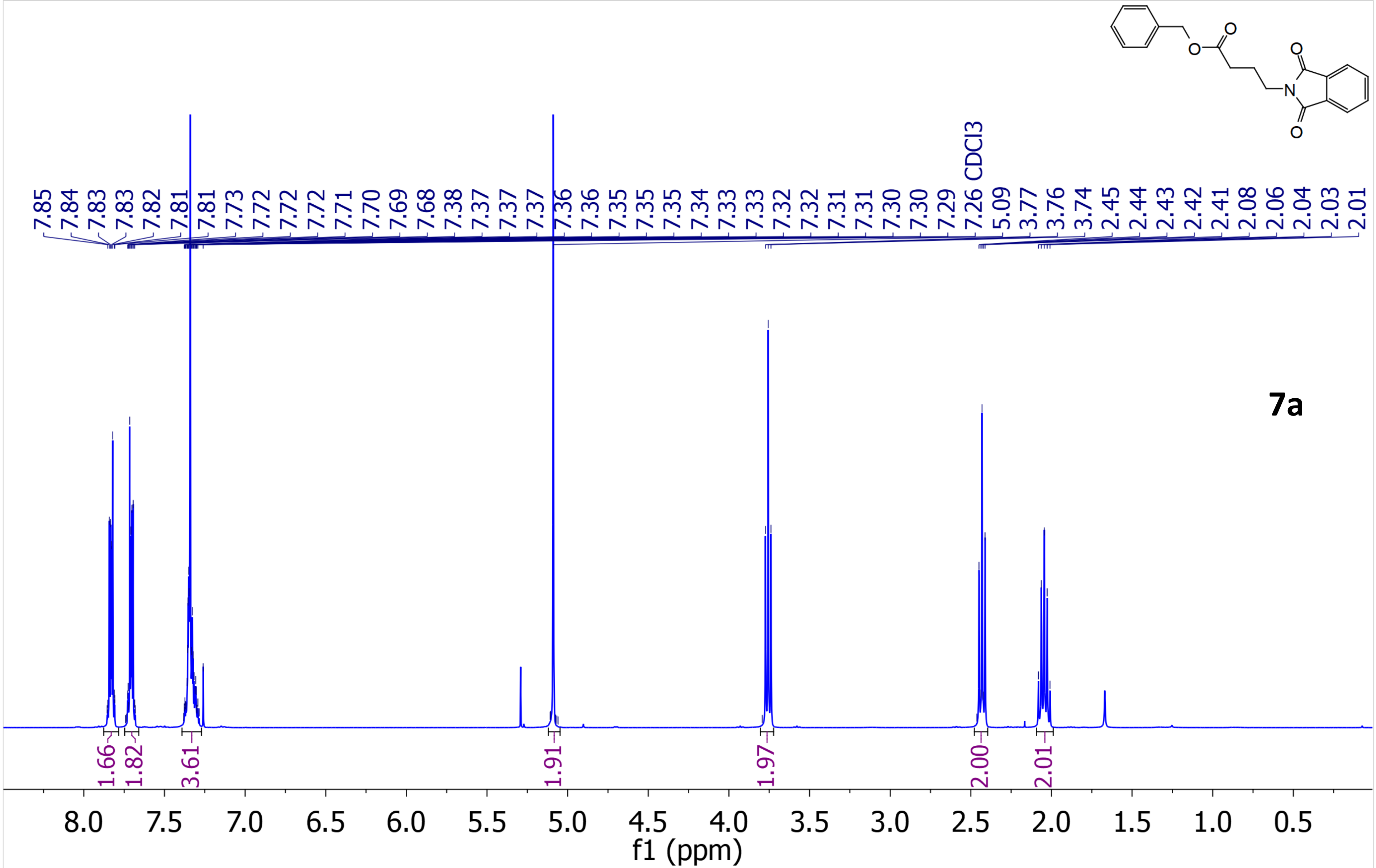

^1H NMR (400 MHz, Chloroform-*d*) δ 7.88 – 7.78 (m, 2H), 7.75 – 7.66 (m, 2H), 7.39 – 7.27 (m, 5H), 5.09 (s, 2H), 3.76 (t, *J* = 6.9 Hz, 2H), 2.43 (t, *J* = 7.5 Hz, 2H), 2.04 (p, *J* = 7.1 Hz, 2H).

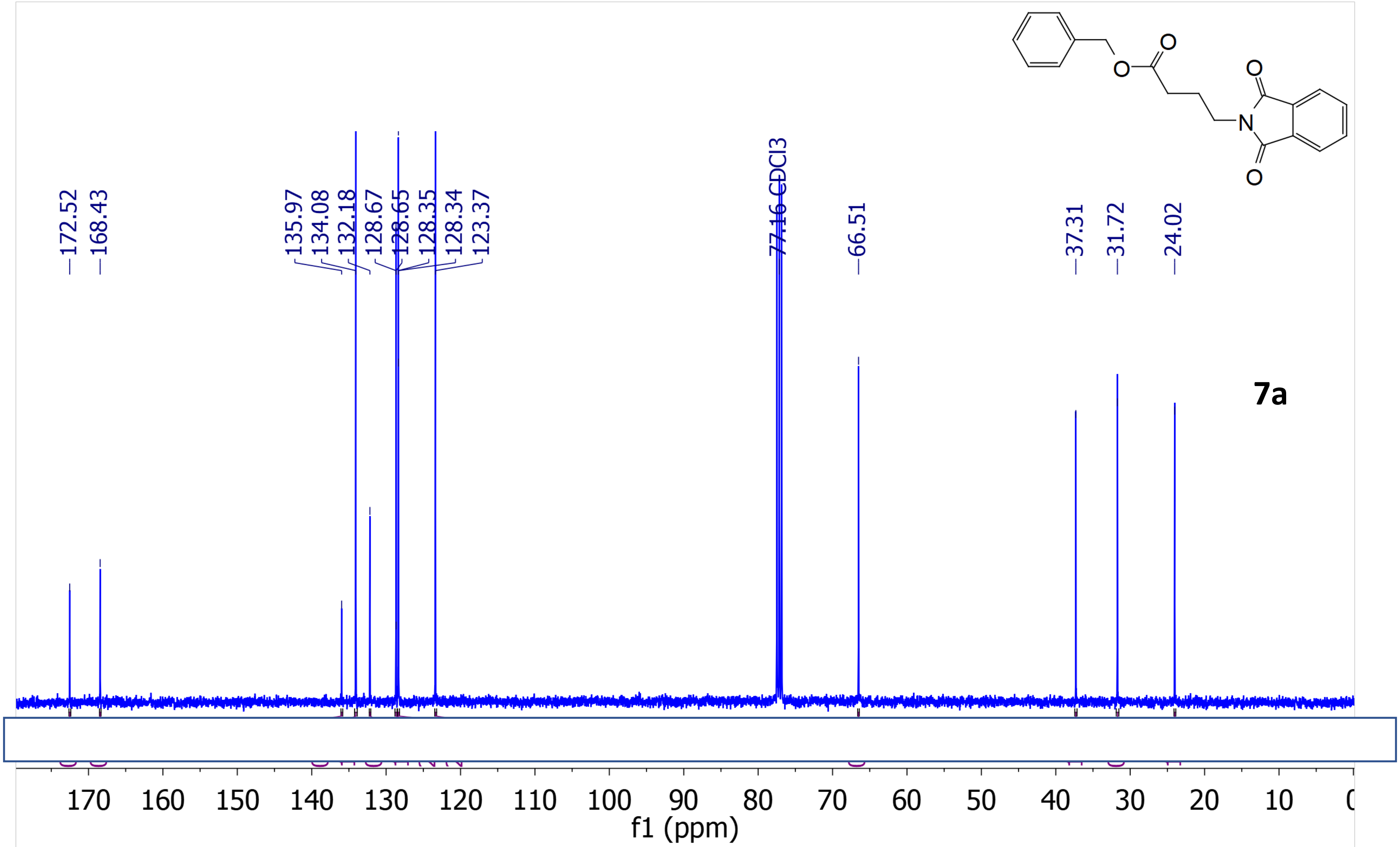

^{13}C NMR (101 MHz, Chloroform-*d*) δ 172.52, 168.43, 135.97, 134.08, 132.18, 128.67, 128.65, 128.35, 128.34, 123.37, 66.51, 37.31, 31.72, 24.02.

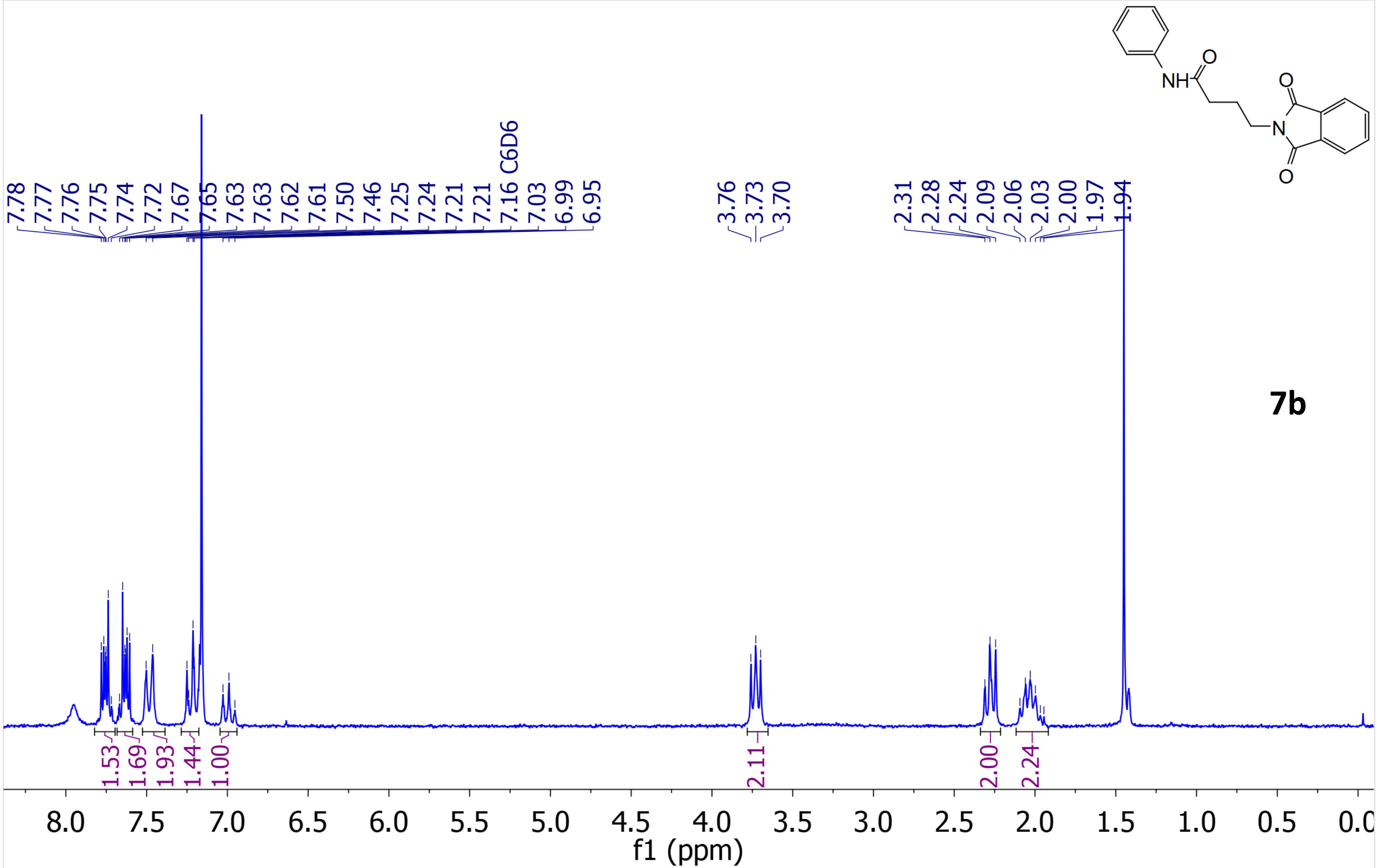

^{1}H NMR (200 MHz, Chloroform-*d*) δ 7.92 – 7.80 (m, 2H), 7.73 (dt, *J* = 5.1, 3.5 Hz, 2H), 7.58 (d, *J* = 8.1 Hz, 2H), 7.33 (dd, *J* = 7.3, 1.8 Hz, 2H), 7.09 (t, *J* = 7.4 Hz, 1H), 3.88 – 3.75 (m, 2H), 2.44 – 2.31 (m, 2H), 2.12 (td, *J* = 11.6, 10.4, 5.5 Hz, 2H).

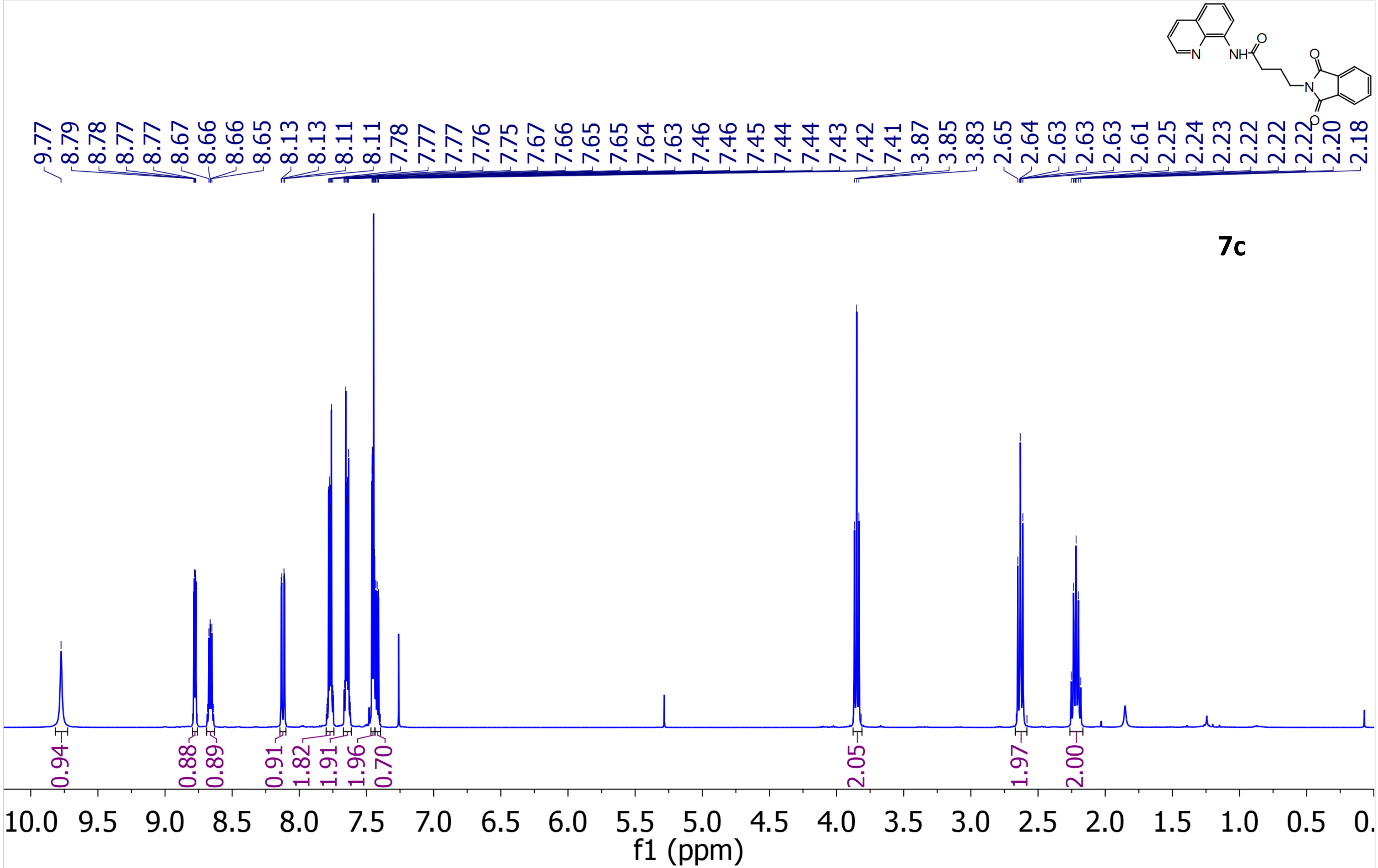

^{1}H NMR (400 MHz, Chloroform-*d*) δ 9.77 (s, 1H), 8.78 (dd, *J* = 4.3, 1.7 Hz, 1H), 8.69 – 8.63 (m, 1H), 8.12 (dd, *J* = 8.3, 1.7 Hz, 1H), 7.77 (dd, *J* = 5.4, 3.0 Hz, 2H), 7.67 – 7.61 (m, 2H), 7.47 – 7.44 (m, 2H), 7.44 – 7.40 (m, 1H), 3.85 (t, *J* = 6.7 Hz, 2H), 2.63 (dd, *J* = 8.0, 7.0 Hz, 2H), 2.26 – 2.17 (m, 2H). product contains 1% DCM by weight

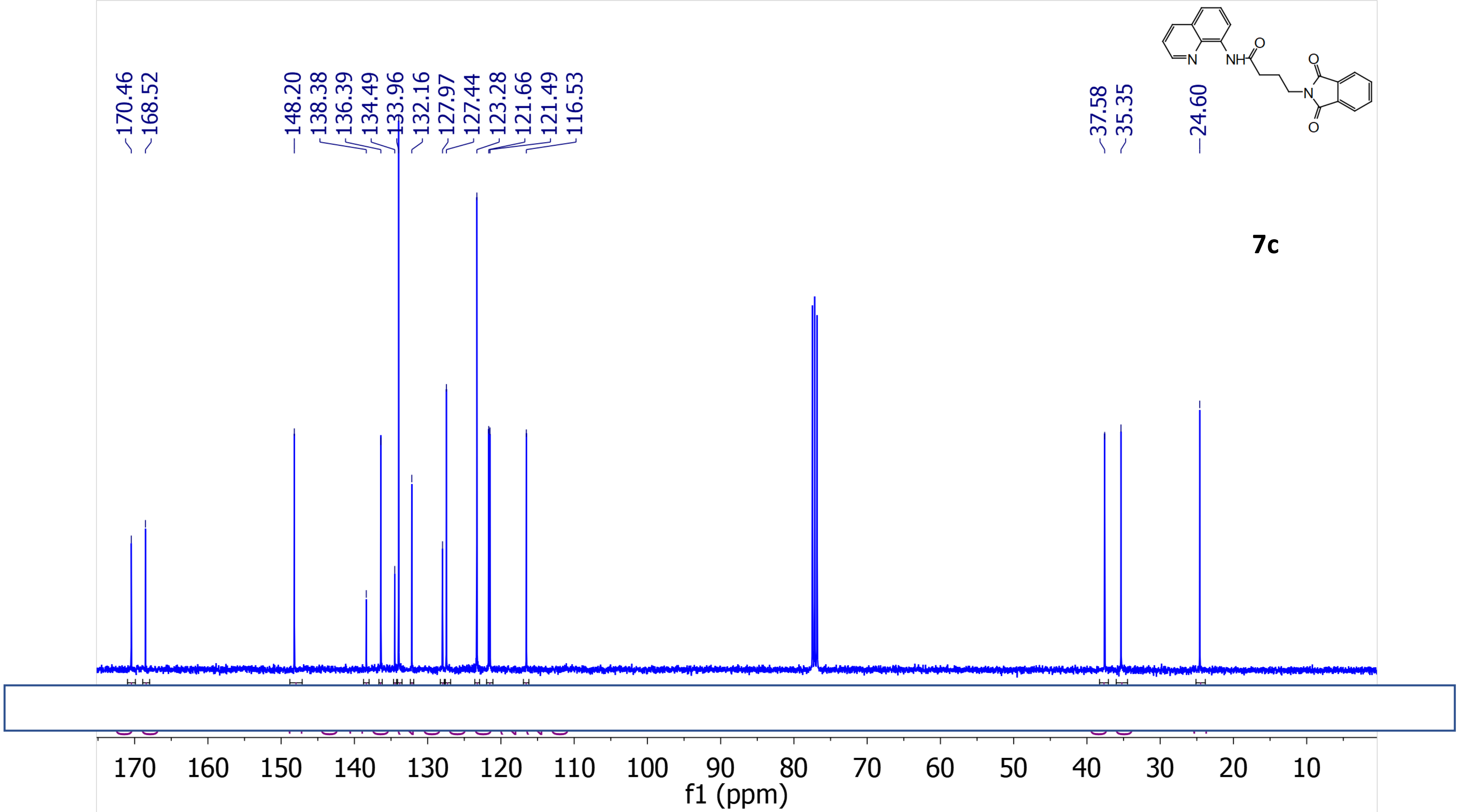

^{13}C NMR (101 MHz, $CDCl_3$) δ 170.46, 168.52, 148.20, 138.38, 136.39, 134.49, 133.96, 132.16, 127.97, 127.44, 123.28, 121.66, 121.49, 116.53, 37.58, 35.35, 24.60.

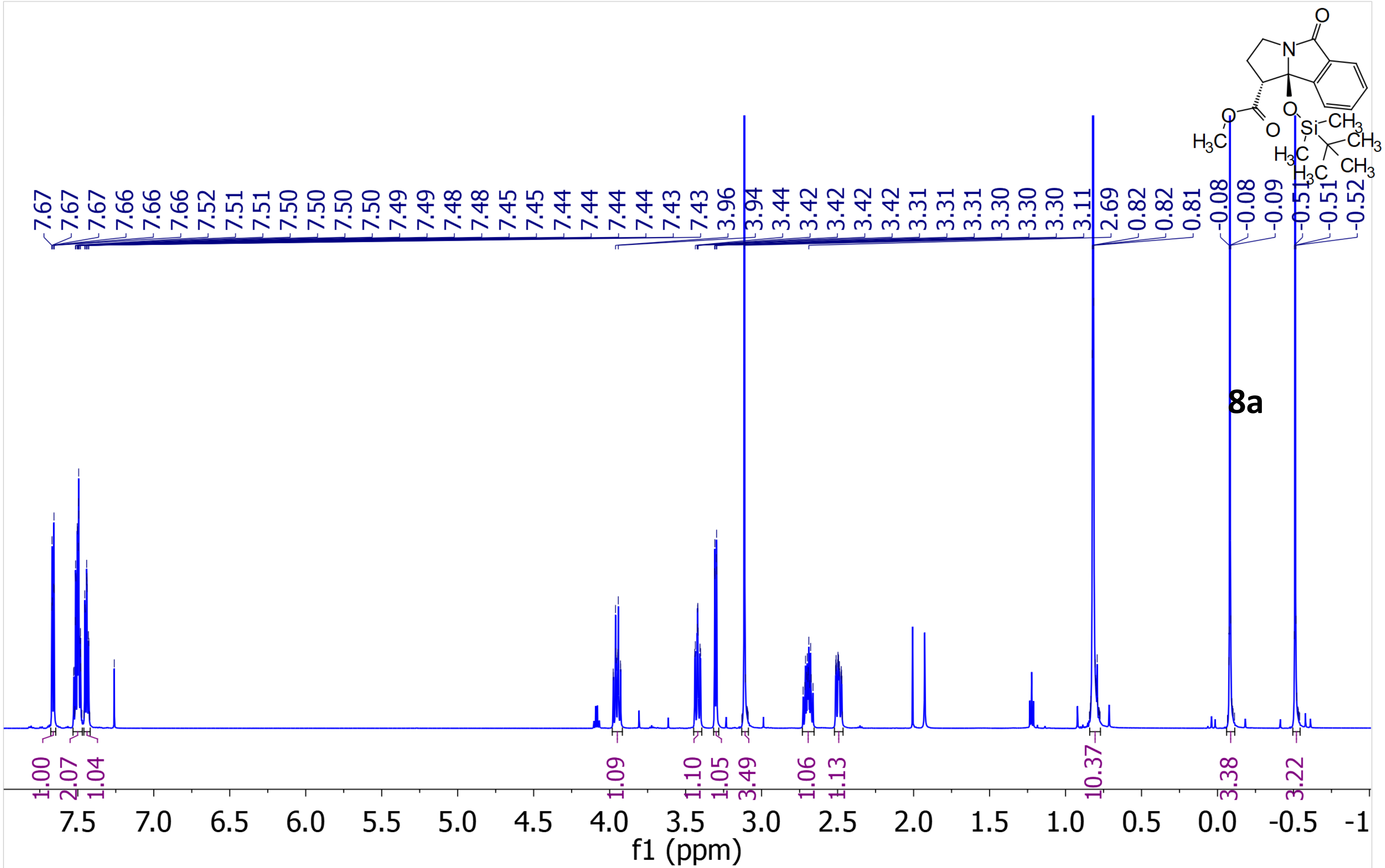

^{1}H NMR (600 MHz, Chloroform-*d*) δ 7.66 (dd, *J* = 7.5, 1.0 Hz, 1H), 7.53 – 7.47 (m, 2H), 7.44 (ddd, *J* = 7.5, 6.9, 1.5 Hz, 1H), 3.95 (dt, *J* = 10.7, 8.5 Hz, 1H), 3.42 (ddd, *J* = 11.0, 9.5, 2.2 Hz, 1H), 3.30 (d, *J* = 6.8 Hz, 1H), 3.11 (s, 3H), 2.73 – 2.66 (m, 1H), 2.49 (dddd, *J* = 13.1, 8.5, 2.2, 1.0 Hz, 1H), 0.82 (s, 9H), -0.08 (s, 3H), -0.51 (s, 3H).

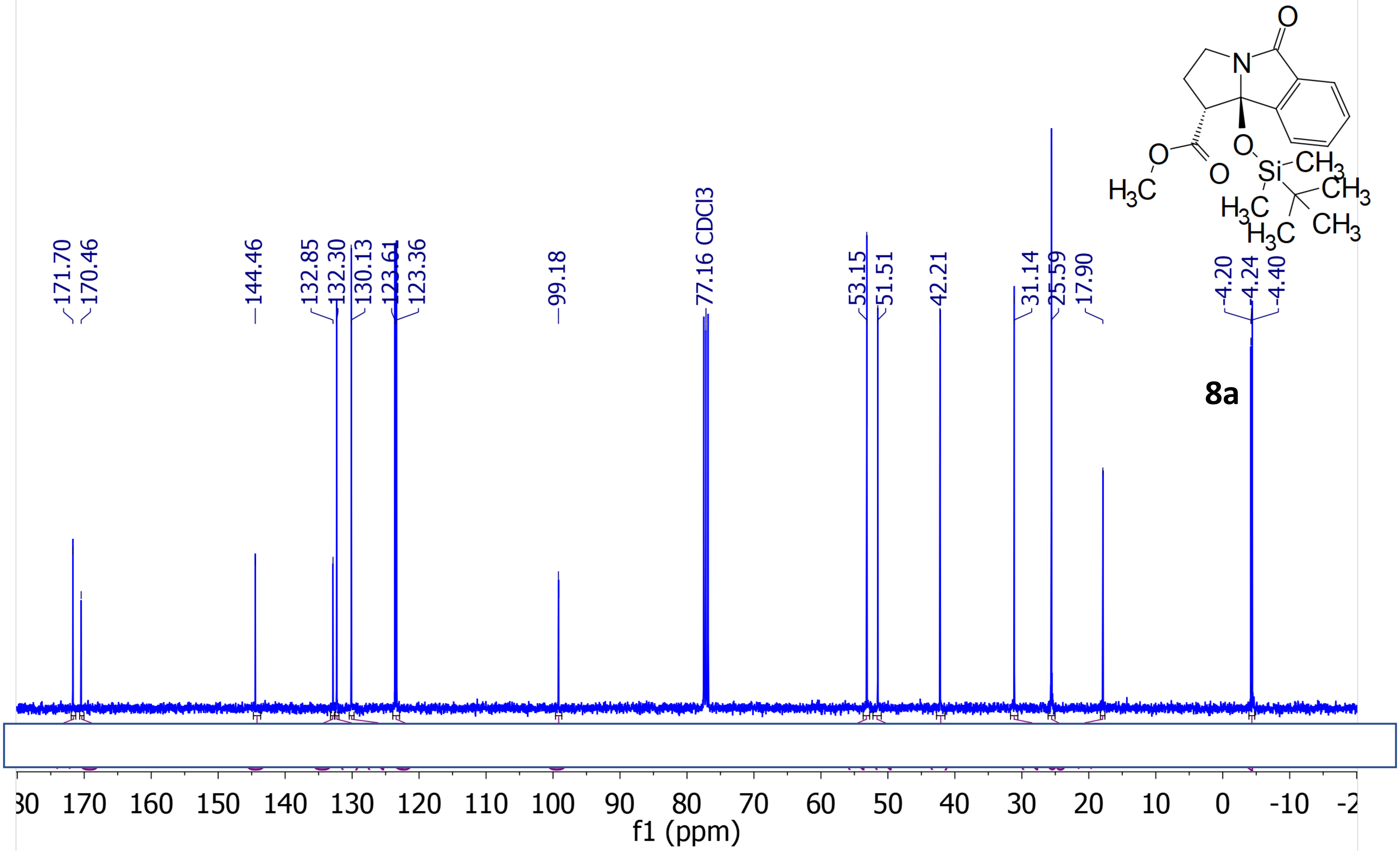

^{13}C NMR (101 MHz, $CDCl_3$) δ 171.60, 170.35, 144.36, 132.75, 132.20, 130.03, 123.50, 123.26, 99.08, 53.05, 51.41, 42.11, 31.03, 25.48, 17.80, -4.30, -4.34, -4.50.

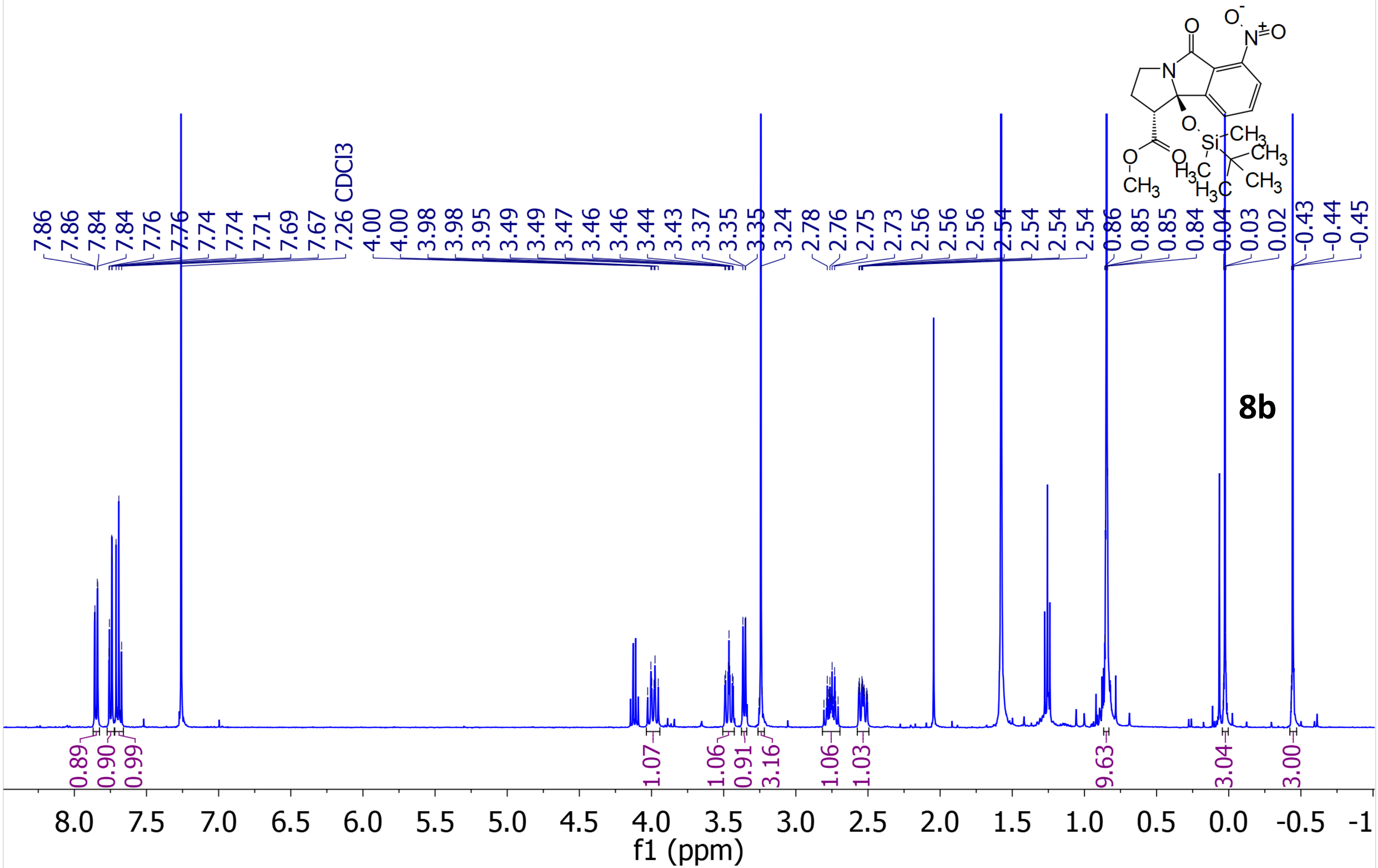

^{1}H NMR (400 MHz, Chloroform-*d*) δ 7.85 (dd, *J* = 7.8, 1.1 Hz, 1H), 7.75 (dd, *J* = 7.7, 1.1 Hz, 1H), 7.69 (t, *J* = 7.6 Hz, 1H), 3.99 (dt, *J* = 11.5, 9.0 Hz, 1H), 3.46 (ddd, *J* = 11.4, 9.4, 2.1 Hz, 1H), 3.38 – 3.34 (m, 1H), 3.24 (s, 3H), 2.76 (dtd, *J* = 13.3, 9.4, 7.1 Hz, 1H), 2.53 (dddd, *J* = 13.4, 8.5, 2.2, 1.0 Hz, 1H), 0.85 (s, 9H), 0.03 (s, 3H), -0.44 (s, 3H).

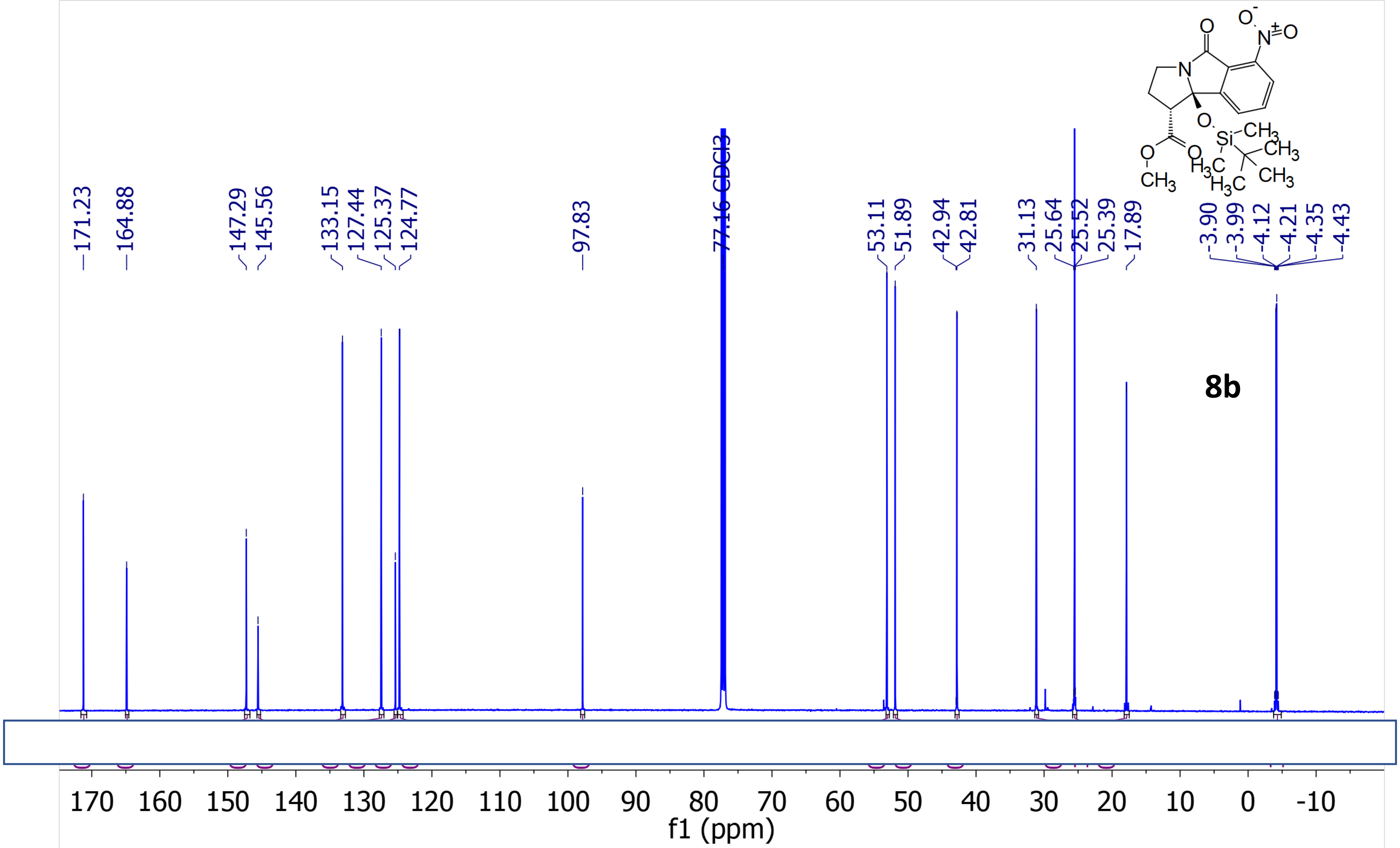

^{13}C NMR (126 MHz, $CDCl_3$) δ 171.23, 164.88, 147.29, 145.56, 133.15, 127.44, 125.37, 124.77, 97.83, 53.11, 51.89, 42.94, 42.81, 31.13, 25.64, 25.52, 25.39, 17.89, -3.90, -3.99, -4.12, -4.21, -4.35, -4.43.

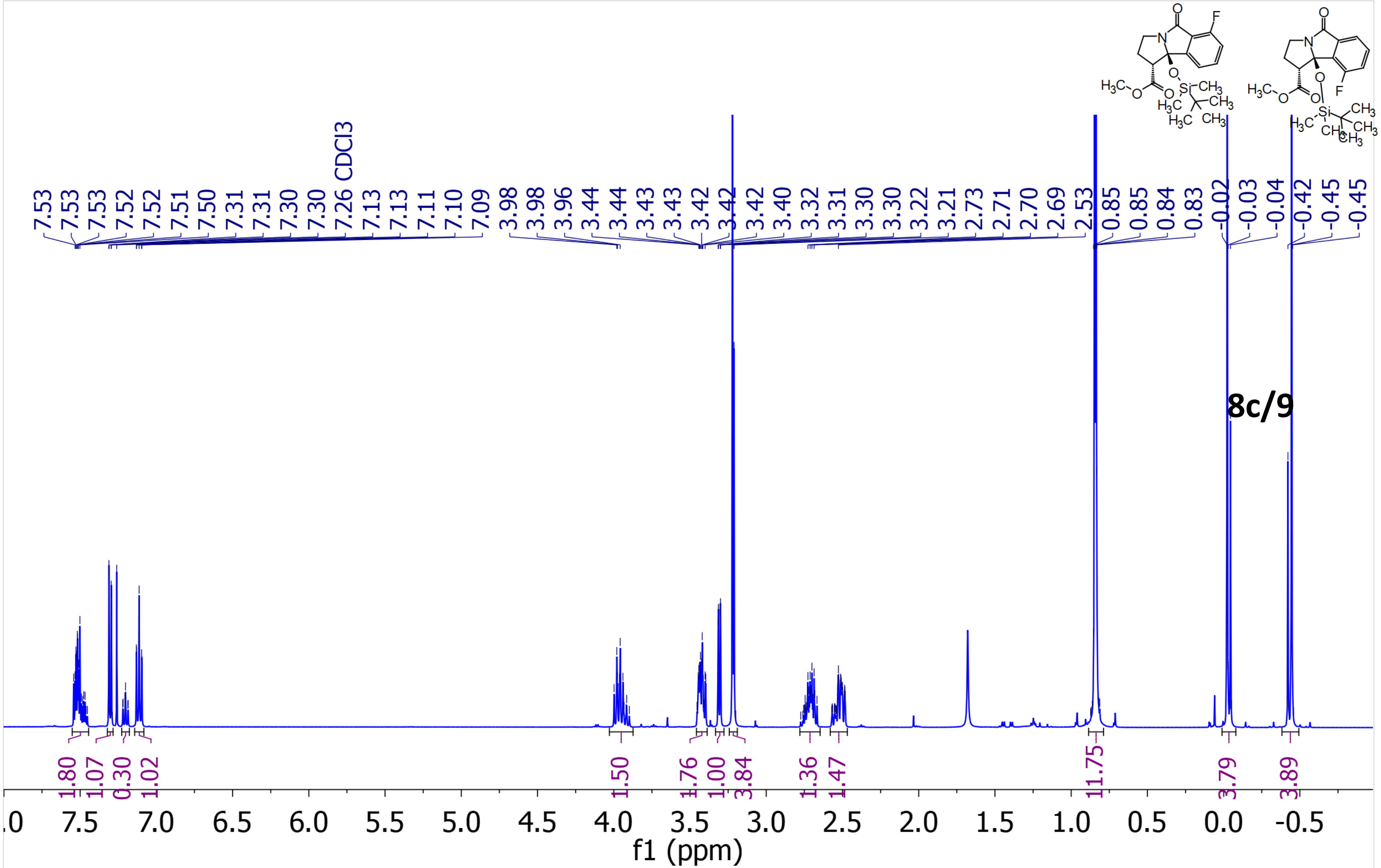

^{1}H NMR (500 MHz, Chloroform-*d*) δ 7.55 – 7.44 (m, 2H), 7.32 – 7.28 (m, 1H), 7.20 (ddd, *J* = 8.9, 7.9, 1.1 Hz, 0H), 7.14 – 7.08 (m, 1H), 4.03 – 3.87 (m, 2H), 3.46 – 3.39 (m, 2H), 3.31 (dd, *J* = 7.0, 1.0 Hz, 1H), 3.22 (d, *J* = 5.1 Hz, 4H), 2.72 (ddtd, *J* = 16.5, 13.3, 9.4, 7.1 Hz, 1H), 2.58 – 2.47 (m, 1H), 0.84 (d, *J* = 4.4 Hz, 12H), -0.03 (d, *J* = 10.8 Hz, 4H), -0.43 (d, *J* = 12.0 Hz, 4H).

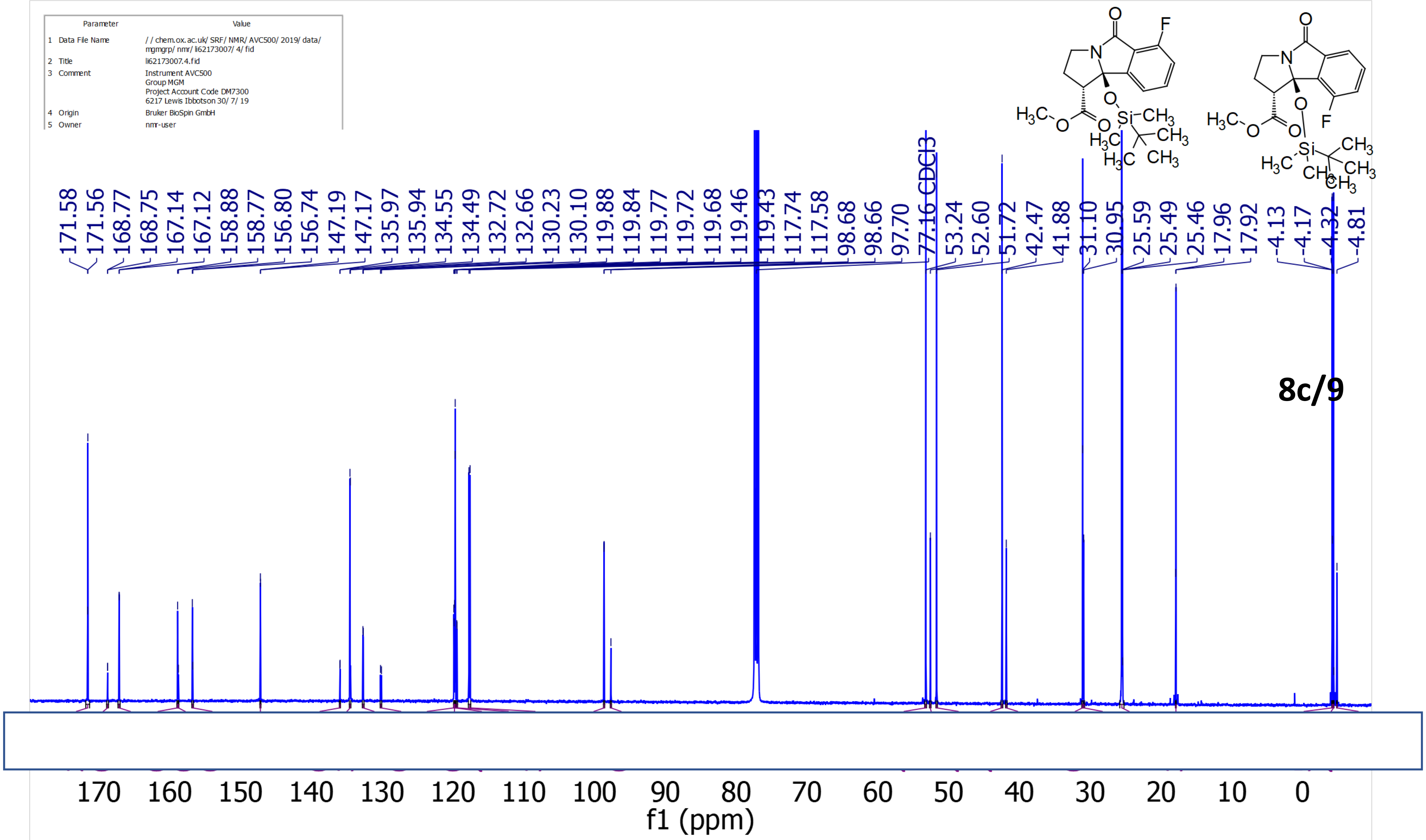

^{13}C NMR (126 MHz, $CDCl_3$) δ 171.58, 171.56, 168.77, 168.75, 167.14, 167.12, 158.88, 158.77, 156.80, 156.74, 147.19, 147.17, 135.97, 135.94, 134.55, 134.49, 132.72, 132.66, 130.23, 130.10, 119.88, 119.84, 119.77, 119.72, 119.68, 119.46, 119.43, 117.74, 117.58, 98.68, 98.66, 97.70, 53.24, 52.60, 51.72, 42.47, 41.88, 31.10, 30.95, 25.59, 25.49, 25.46, 17.96, 17.92, -4.13, -4.17, -4.32, -4.36, -4.81.

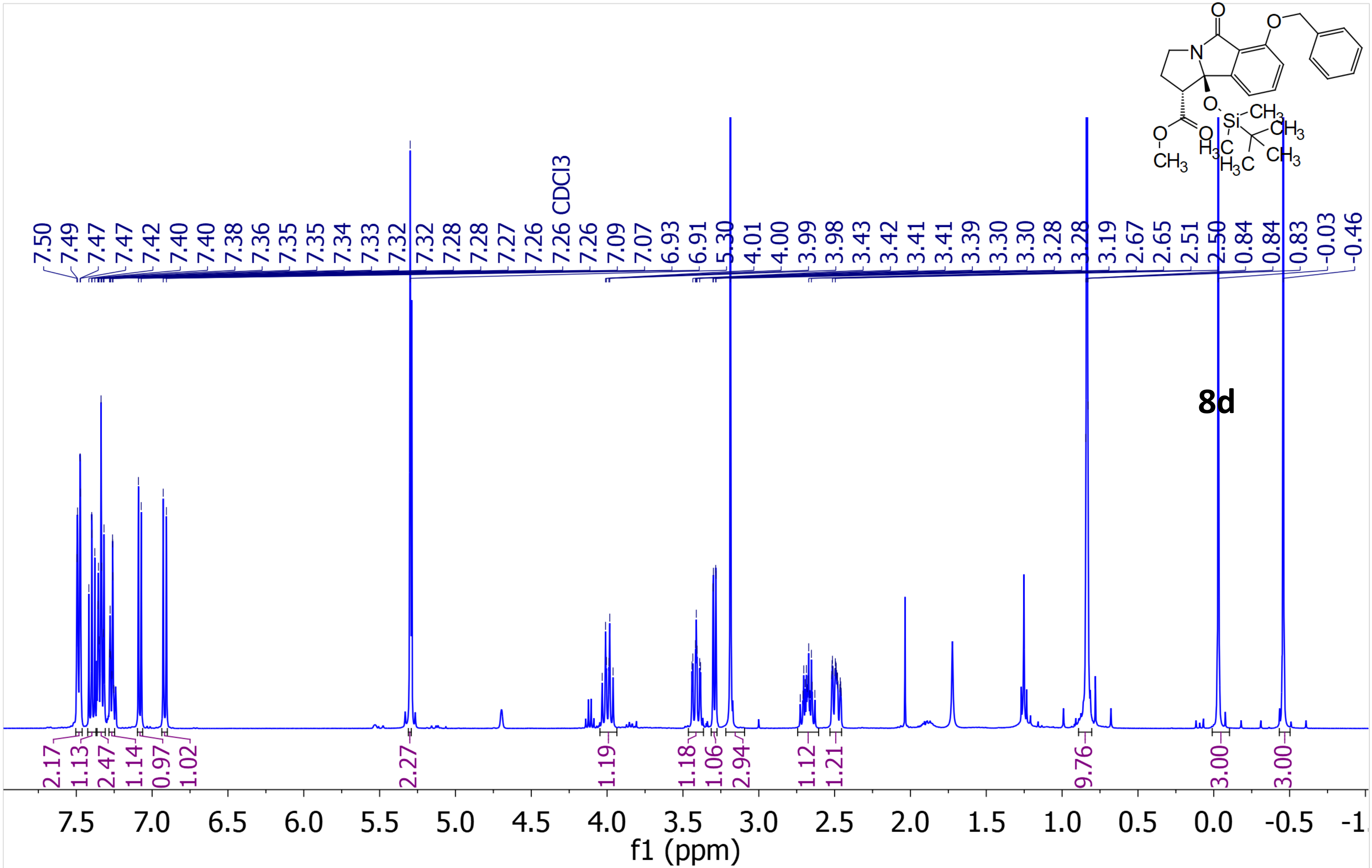

^{1}H NMR (400 MHz, Chloroform-*d*) δ 7.52 – 7.48 (m, 2H), 7.42 (dd, *J* = 8.3, 7.5 Hz, 1H), 7.38 – 7.33 (m, 2H), 7.30 – 7.27 (m, 1H), 7.10 (d, *J* = 7.4 Hz, 1H), 6.94 (d, *J* = 8.3 Hz, 1H), 5.32 (s, 2H), 4.02 (dt, *J* = 11.0, 8.8 Hz, 1H), 3.43 (ddd, *J* = 11.3, 9.4, 2.3 Hz, 1H), 3.31 (d, *J* = 7.1 Hz, 1H), 3.21 (s, 3H), 2.70 (dtd, *J* = 13.1, 9.2, 7.1 Hz, 1H), 2.51 (dddd, *J* = 13.1, 8.5, 2.3, 1.2 Hz, 1H), 0.86 (s, 9H), -0.01 (s, 3H), -0.44 (s, 3H).

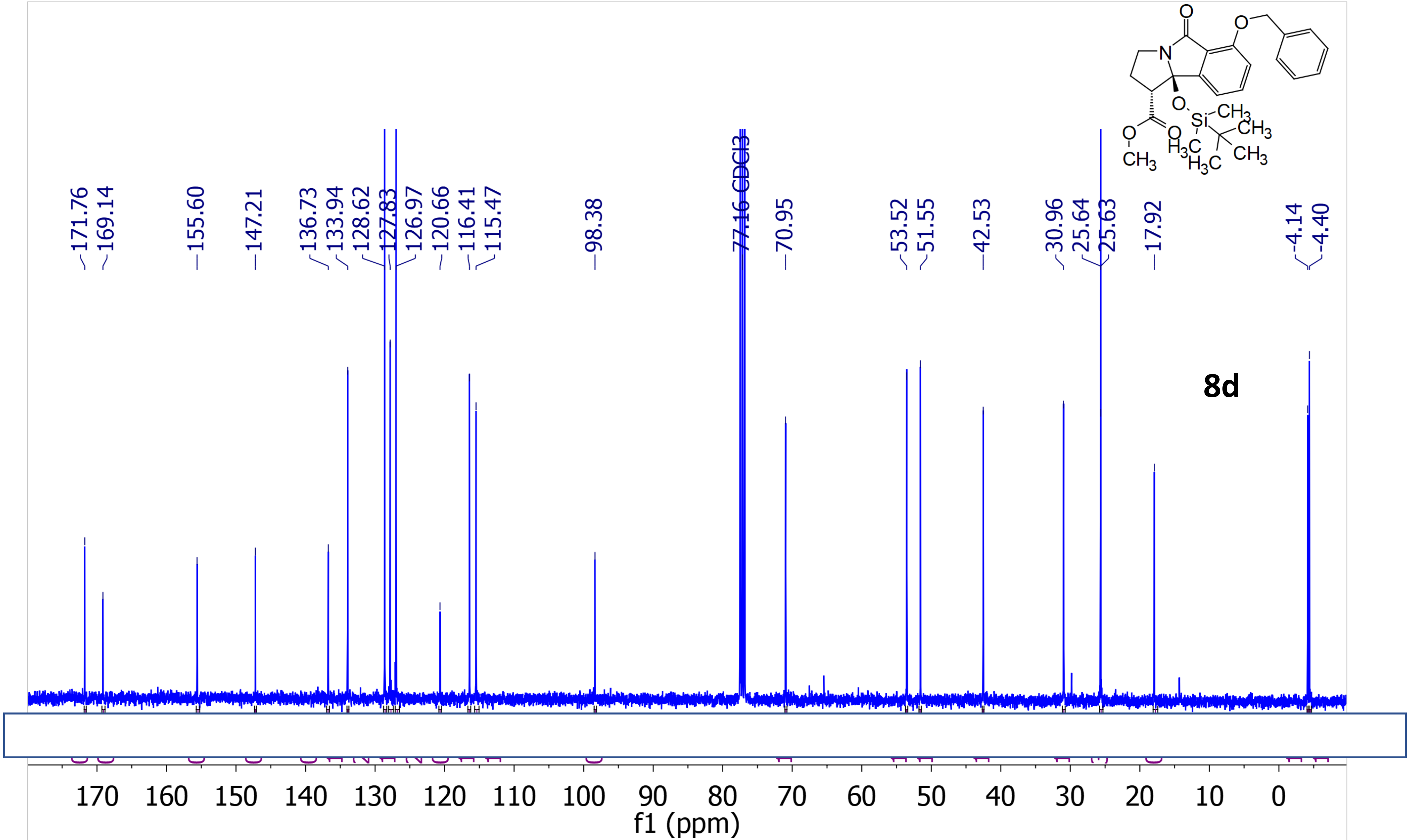

^{13}C NMR (101 MHz, $CDCl_3$) δ 171.76, 169.14, 155.60, 147.21, 136.73, 133.94, 128.62, 127.83, 126.97, 120.66, 116.41, 115.47, 98.38, 70.95, 53.52, 51.55, 42.53, 30.96, 25.64, 25.63, 17.92, -4.14, -4.40.

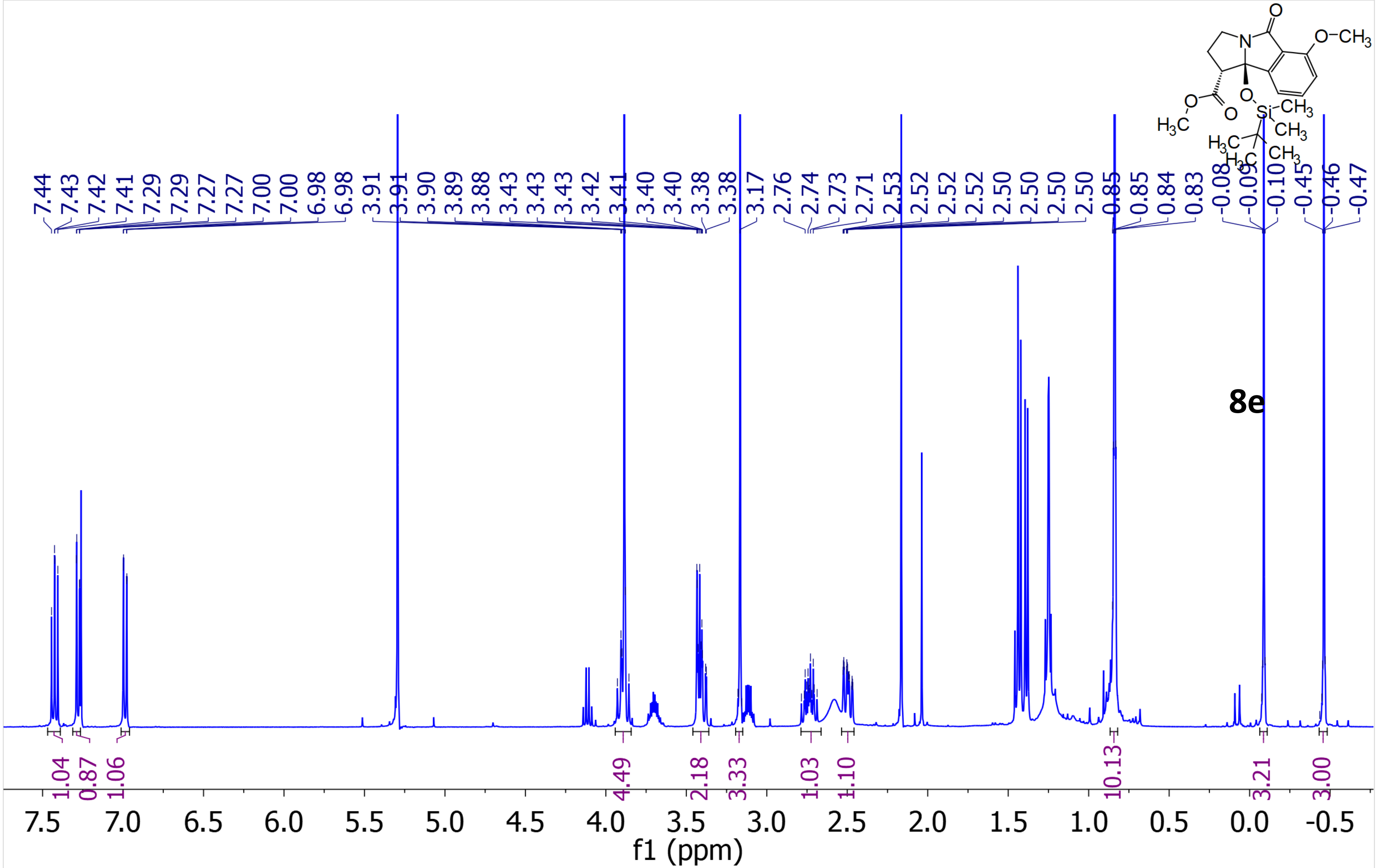

^{1}H NMR (400 MHz, Chloroform-*d*) δ 7.42 (dd, *J* = 8.2, 7.5 Hz, 1H), 7.28 (dd, *J* = 7.5, 0.8 Hz, 1H), 6.99 (dd, *J* = 8.2, 0.7 Hz, 1H), 3.94 – 3.84 (m, 4H), 3.46 – 3.36 (m, 2H), 3.17 (s, 3H), 2.74 (dtd, *J* = 13.1, 9.4, 7.0 Hz, 1H), 2.50 (dddd, *J* = 13.1, 8.6, 2.0, 0.8 Hz, 1H), 0.84 (s, 9H), -0.09 (s, 3H), -0.46 (s, 3H).

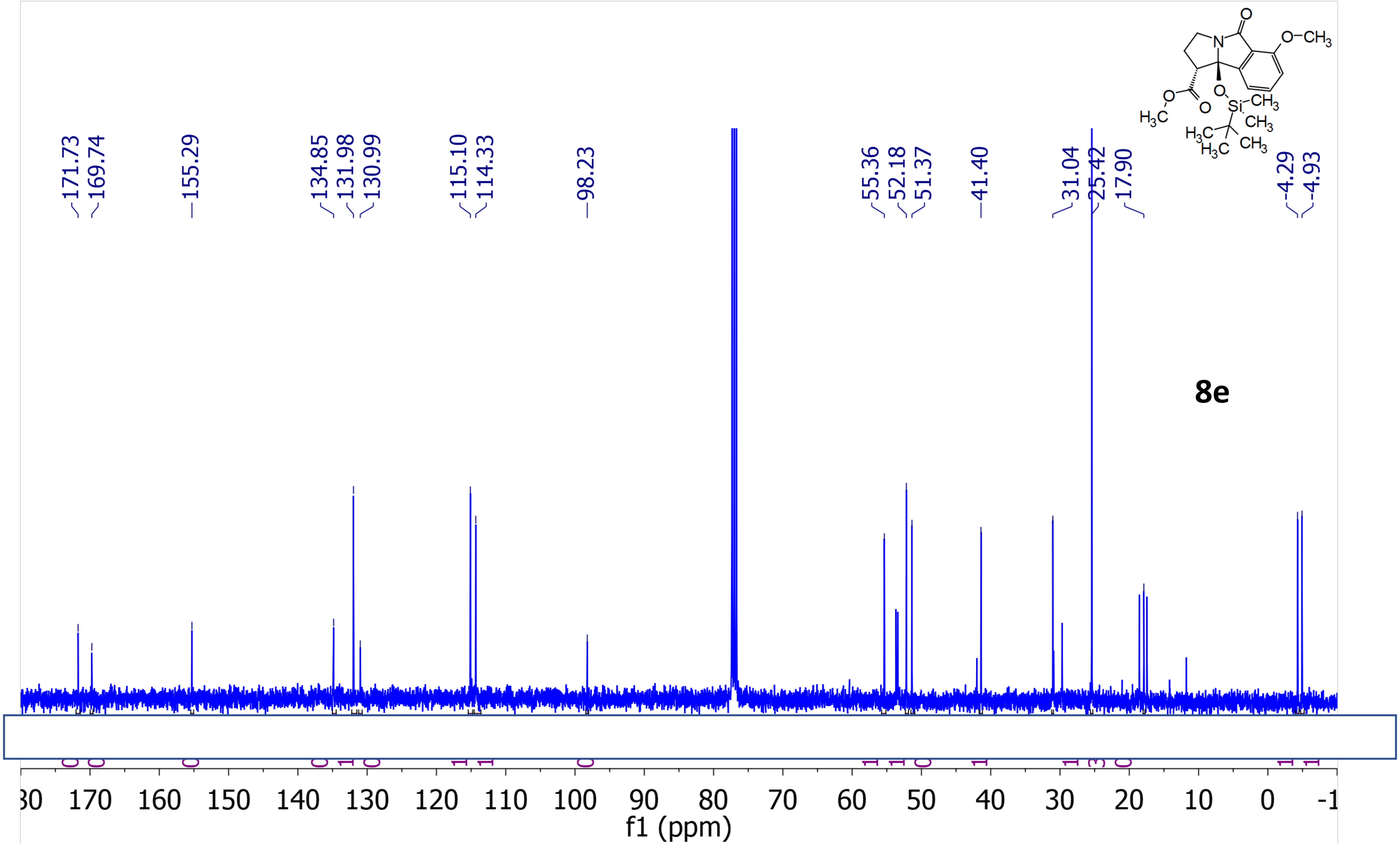

^{13}C NMR (101 MHz, $CDCl_3$) δ 171.73, 169.74, 155.29, 134.85, 131.98, 130.99, 115.10, 114.33, 98.23, 55.36, 52.18, 51.37, 41.40, 31.04, 25.42, 17.90, -4.29, -4.93.

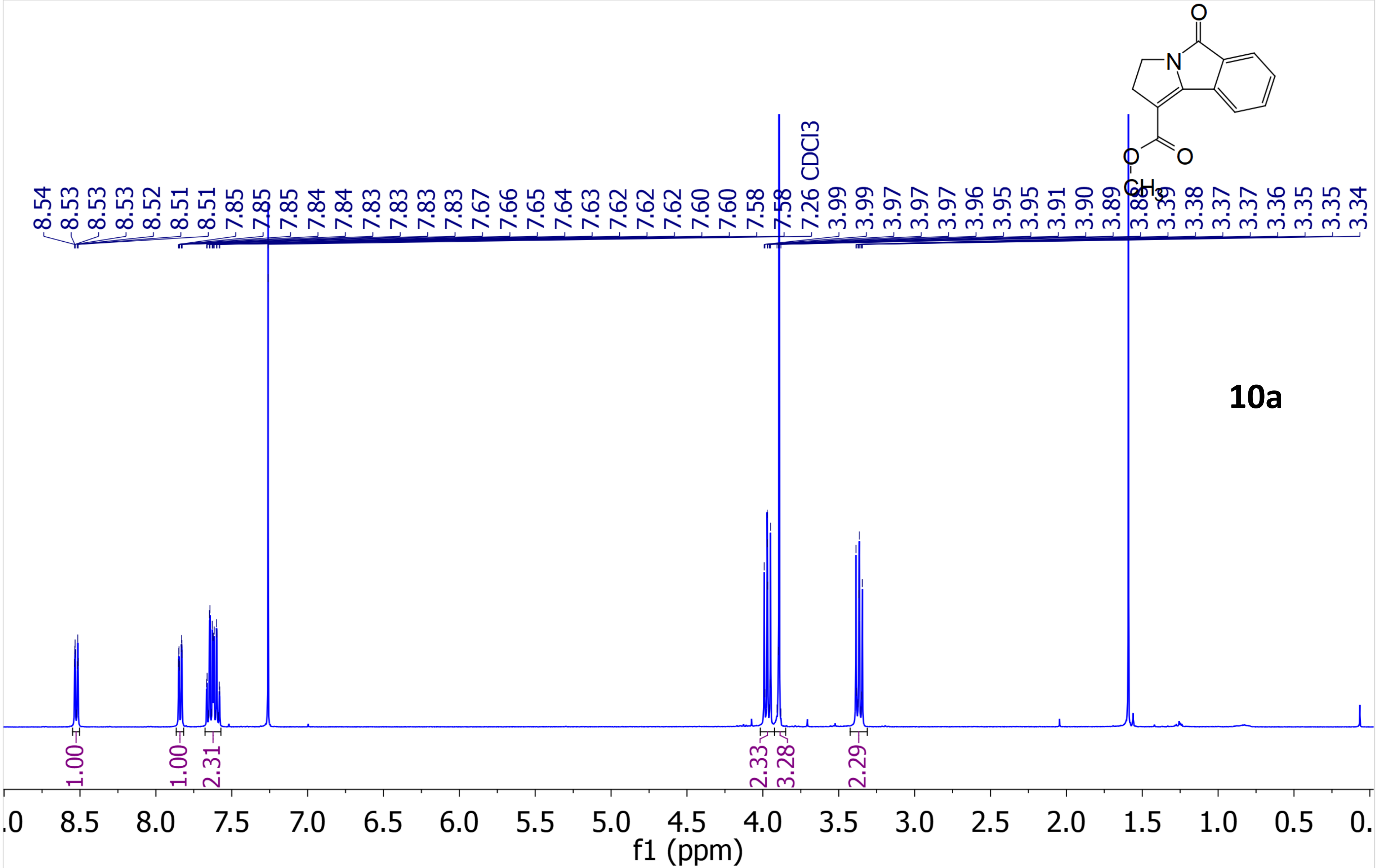

^{1}H NMR (400 MHz, Chloroform-*d*) δ 8.53 (d, 1H), 7.84 (ddd, J = 7.2, 1.5, 0.8 Hz, 1H), 7.68 – 7.57 (m, 2H), 3.97 (t, 2H), 3.89 (s, 3H), 3.37 (t, J = 9.1, 7.7 Hz, 2H).

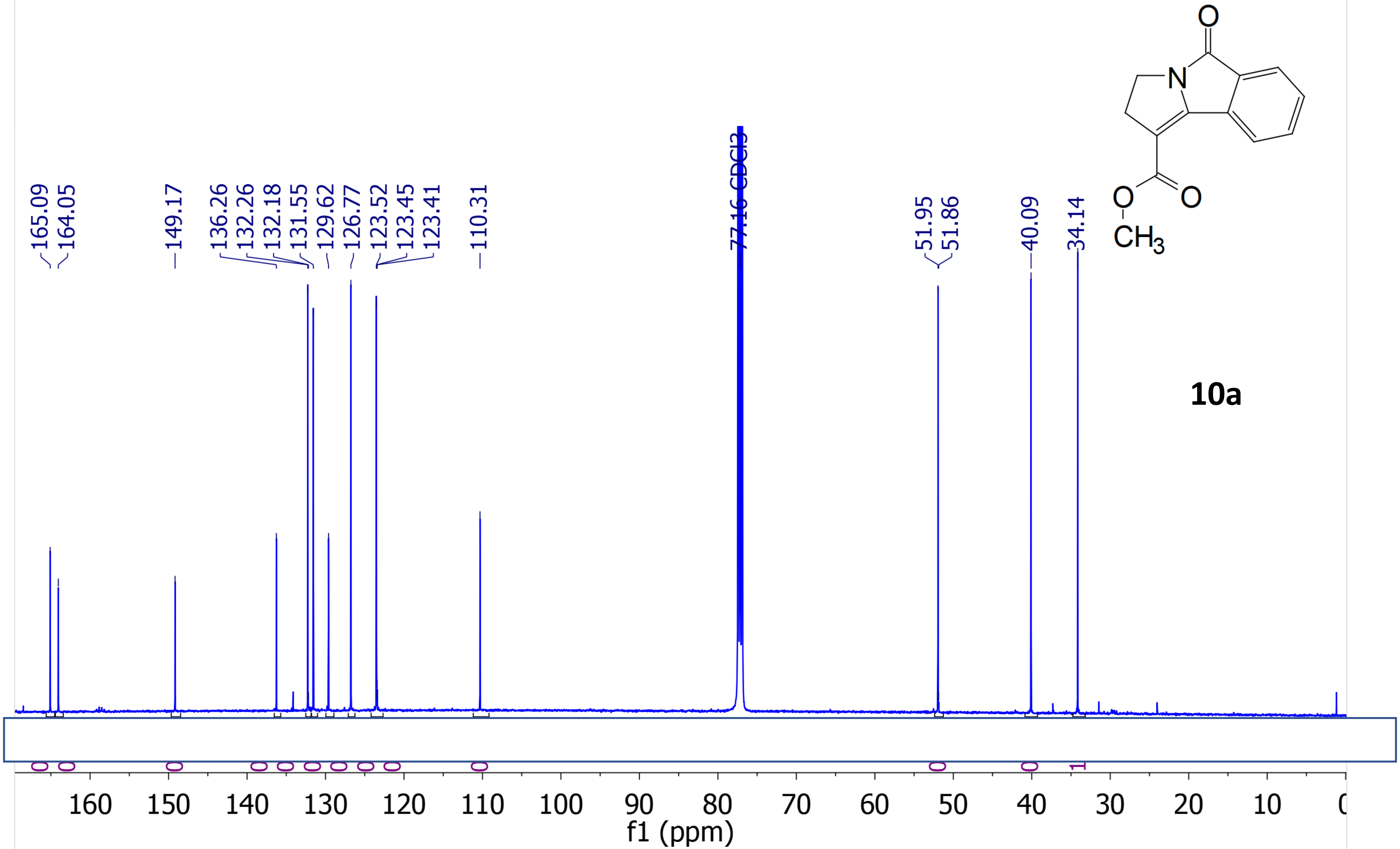

10a

^{13}C NMR (126 MHz, $CDCl_3$) δ 165.09, 164.05, 149.17, 136.26, 132.26, 132.18, 131.55, 129.62, 126.77, 123.52, 123.45, 123.41, 110.31, 51.95, 51.86, 40.09, 34.14.

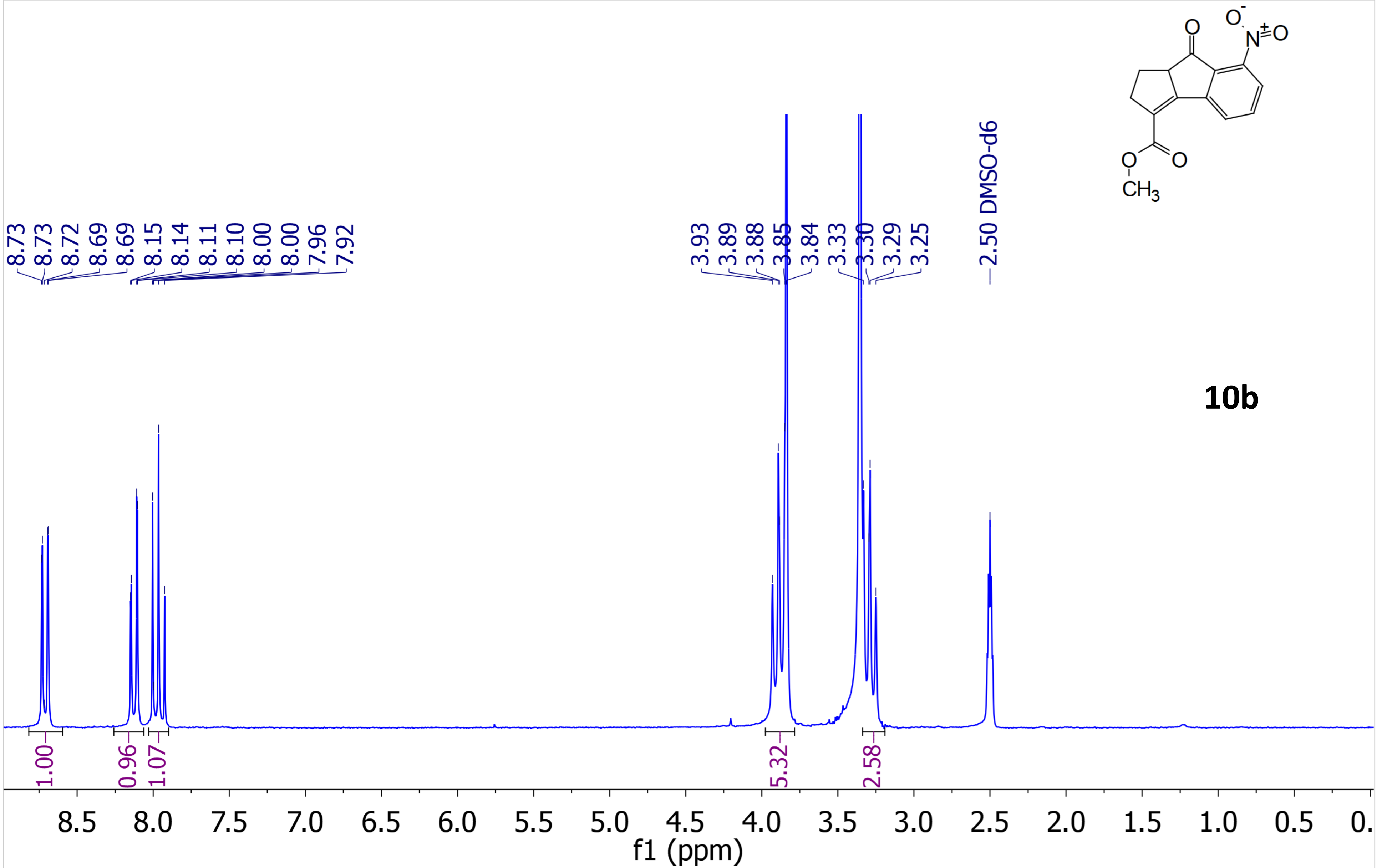

10b

^{1}H NMR (200 MHz, DMSO-d_6) δ 8.71 (dd, J = 7.7, 1.0 Hz, 1H), 8.13 (dd, J = 8.1, 1.0 Hz, 1H), 7.96 (t, J = 7.9 Hz, 1H), 3.98 – 3.78 (m, 5H), 3.34 – 3.19 (m, 3H).

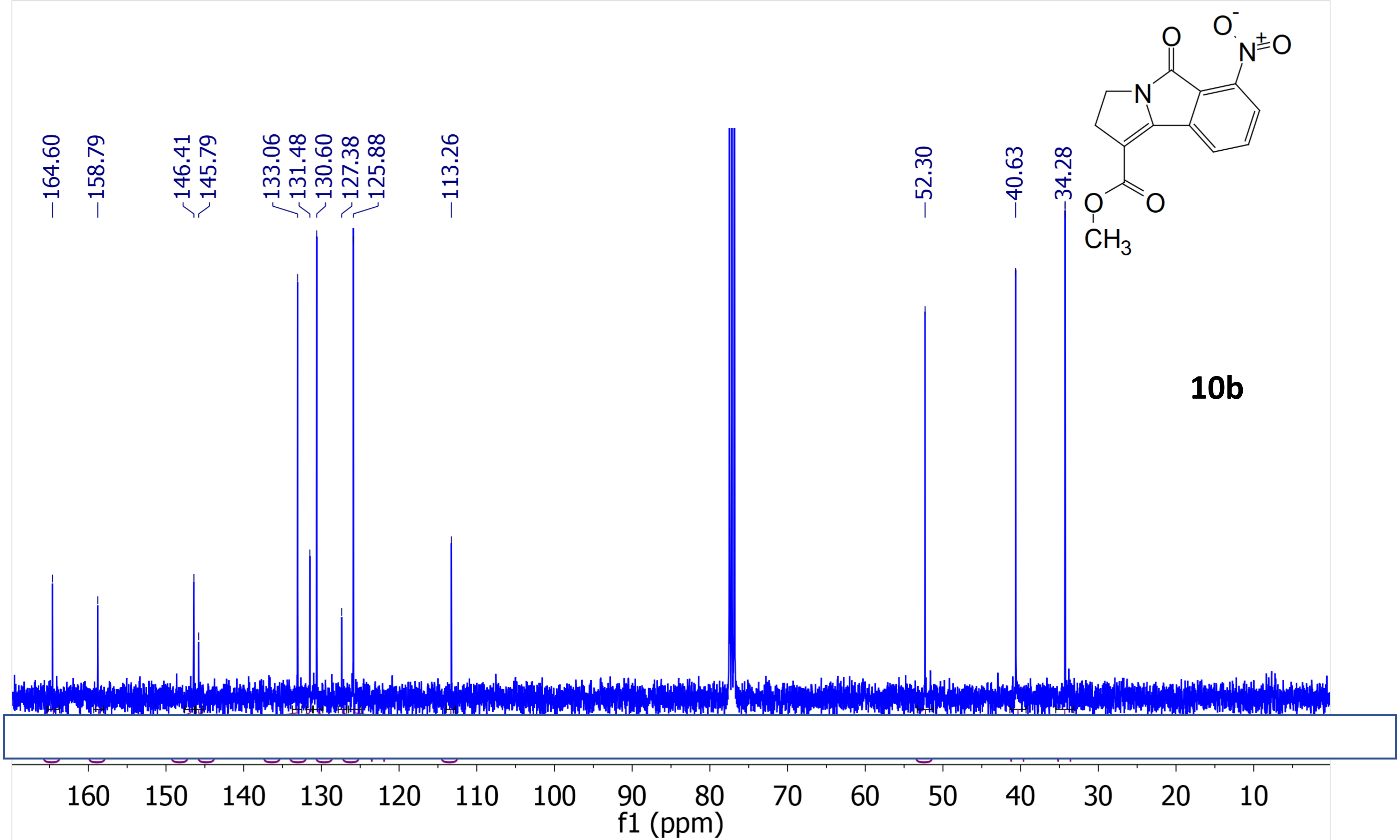

^{13}C NMR (101 MHz, $CDCl_3$) δ 164.60, 158.79, 146.41, 145.79, 133.06, 131.48, 130.60, 127.38, 125.88, 113.26, 52.30, 40.63, 34.28.

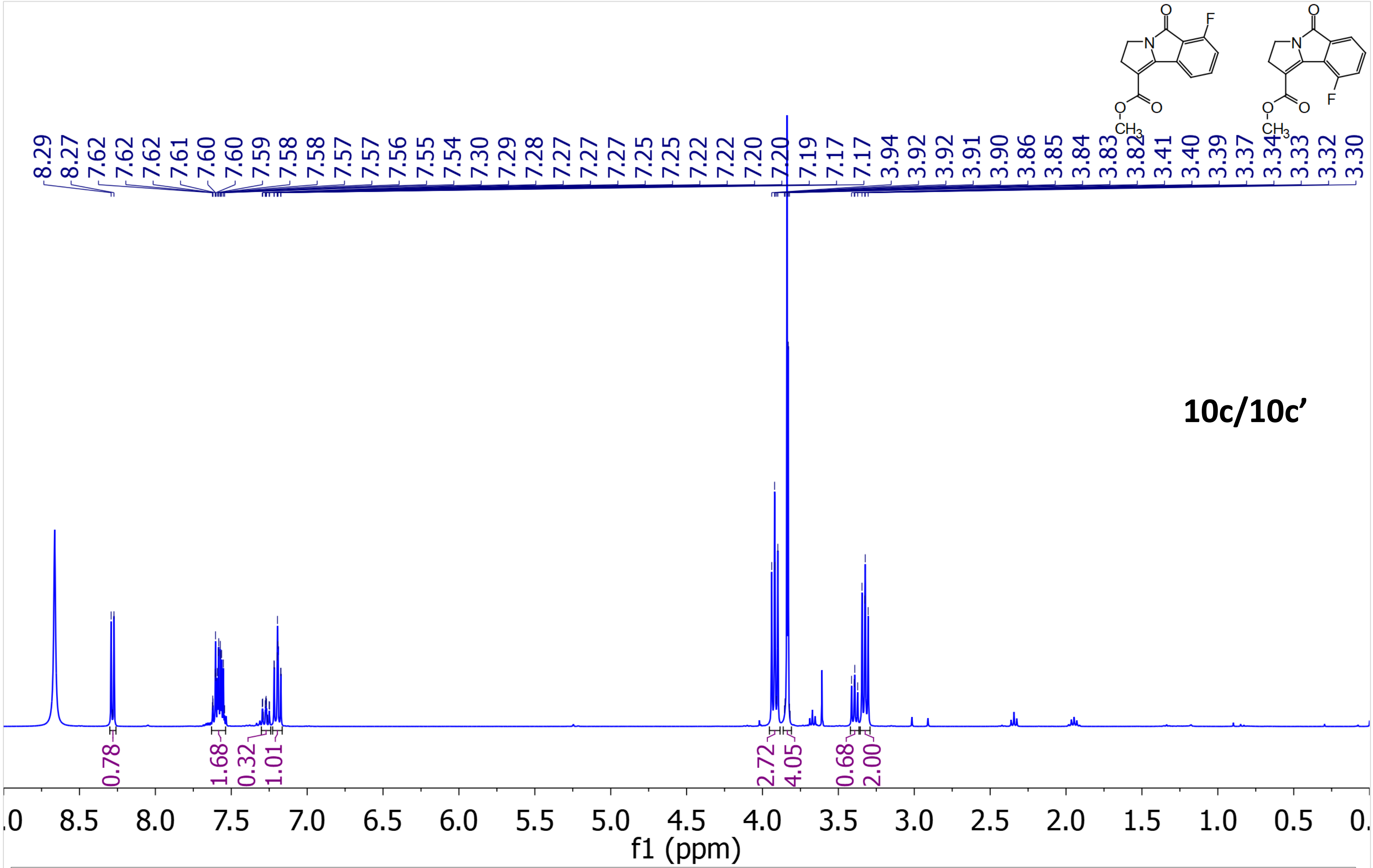

^{1}H NMR (400 MHz, Chloroform-*d*) δ 8.28 (d, *J* = 7.4 Hz, 1H), 7.63 – 7.54 (m, 2H), 7.27 (ddd, *J* = 10.0, 8.1, 1.2 Hz, 0H), 7.20 (ddd, *J* = 9.1, 8.1, 0.7 Hz, 1H), 3.92 (t, *J* = 8.7, 7.5 Hz, 3H), 3.86 – 3.81 (m, 4H), 3.39 (t, *J* = 8.7, 7.4 Hz, 1H), 3.32 (t, *J* = 8.7, 7.5 Hz, 2H).

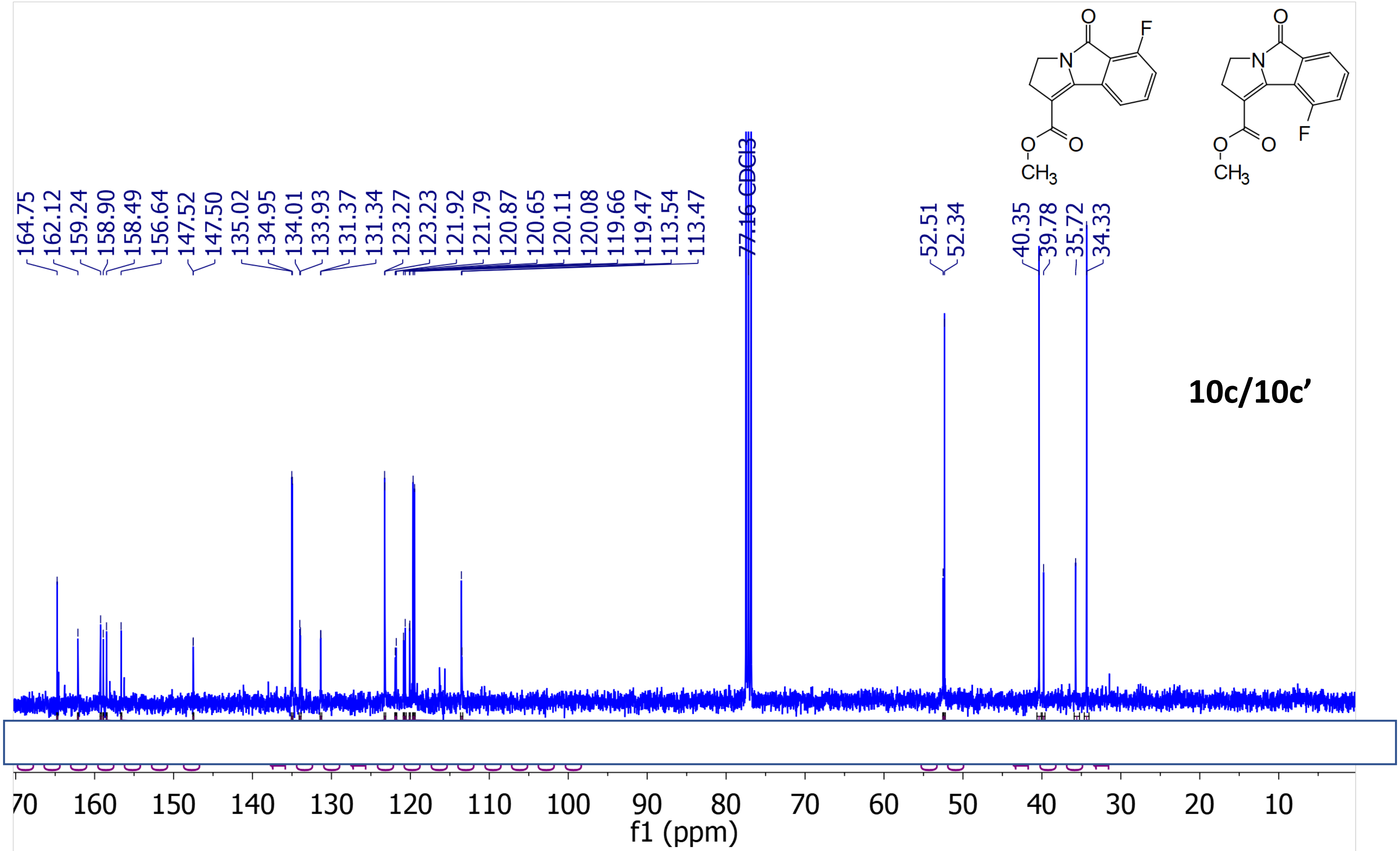

^{13}C NMR (101 MHz, $CDCl_3$) δ 164.75, 162.12, 159.24, 158.90, 158.49, 156.64, 147.52, 147.50, 135.02, 134.95, 134.01, 133.93, 131.37, 131.34, 123.27, 123.23, 121.92, 121.79, 120.87, 120.65, 120.11, 120.08, 119.66, 119.47, 113.54, 113.47, 52.51, 52.34, 40.35, 39.78, 35.72, 34.33.

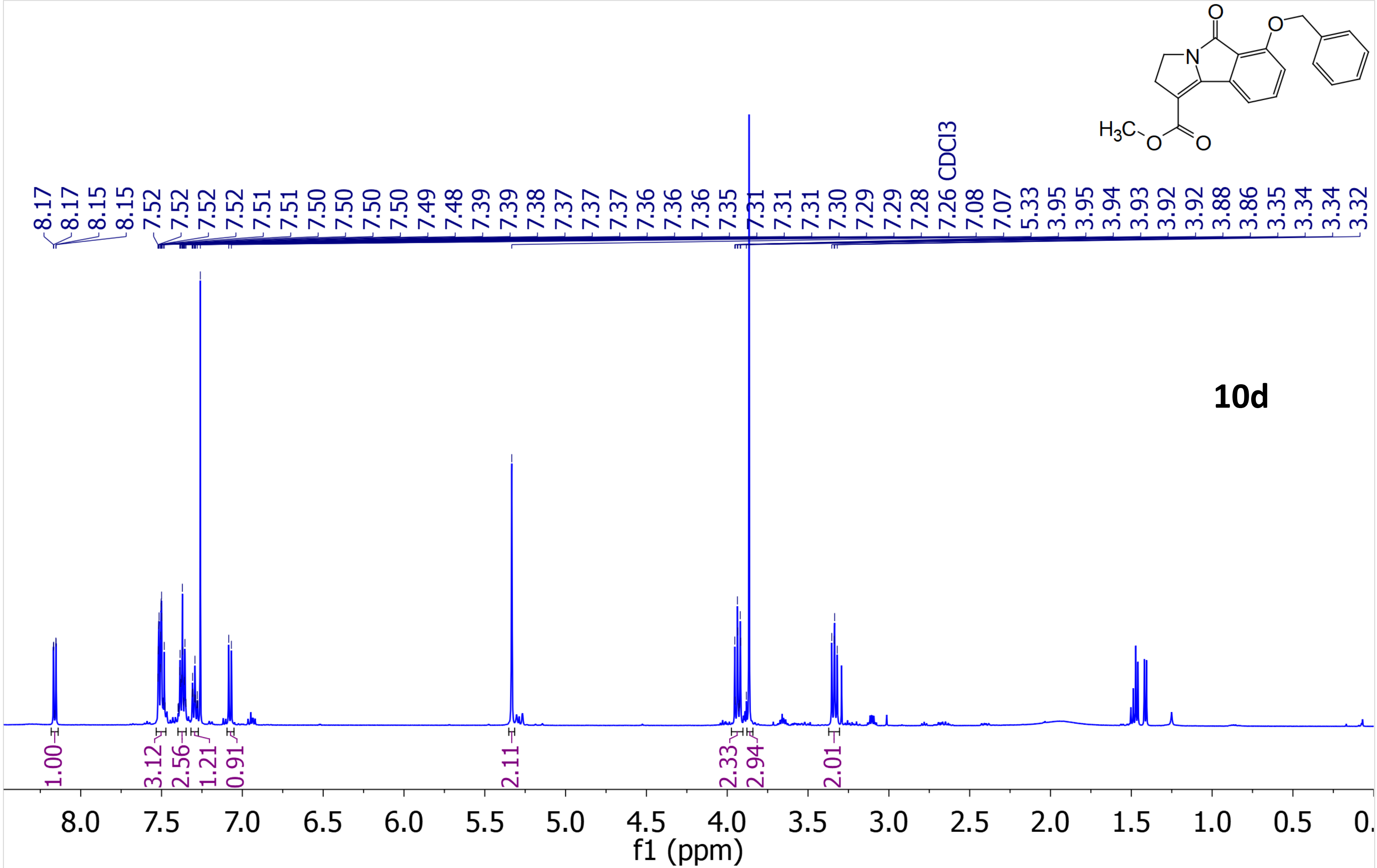

10d

^{1}H NMR (500 MHz, Chloroform-*d*) δ 8.19 (dd, *J* = 7.6, 0.7 Hz, 1H), 7.56 – 7.50 (m, 3H), 7.42 – 7.37 (m, 3H), 7.34 – 7.30 (m, 1H), 7.10 (d, *J* = 8.3 Hz, 1H), 5.36 (s, 2H), 4.00 – 3.93 (m, 2H), 3.89 (s, 3H), 3.36 (dd, *J* = 9.1, 7.8 Hz, 2H).

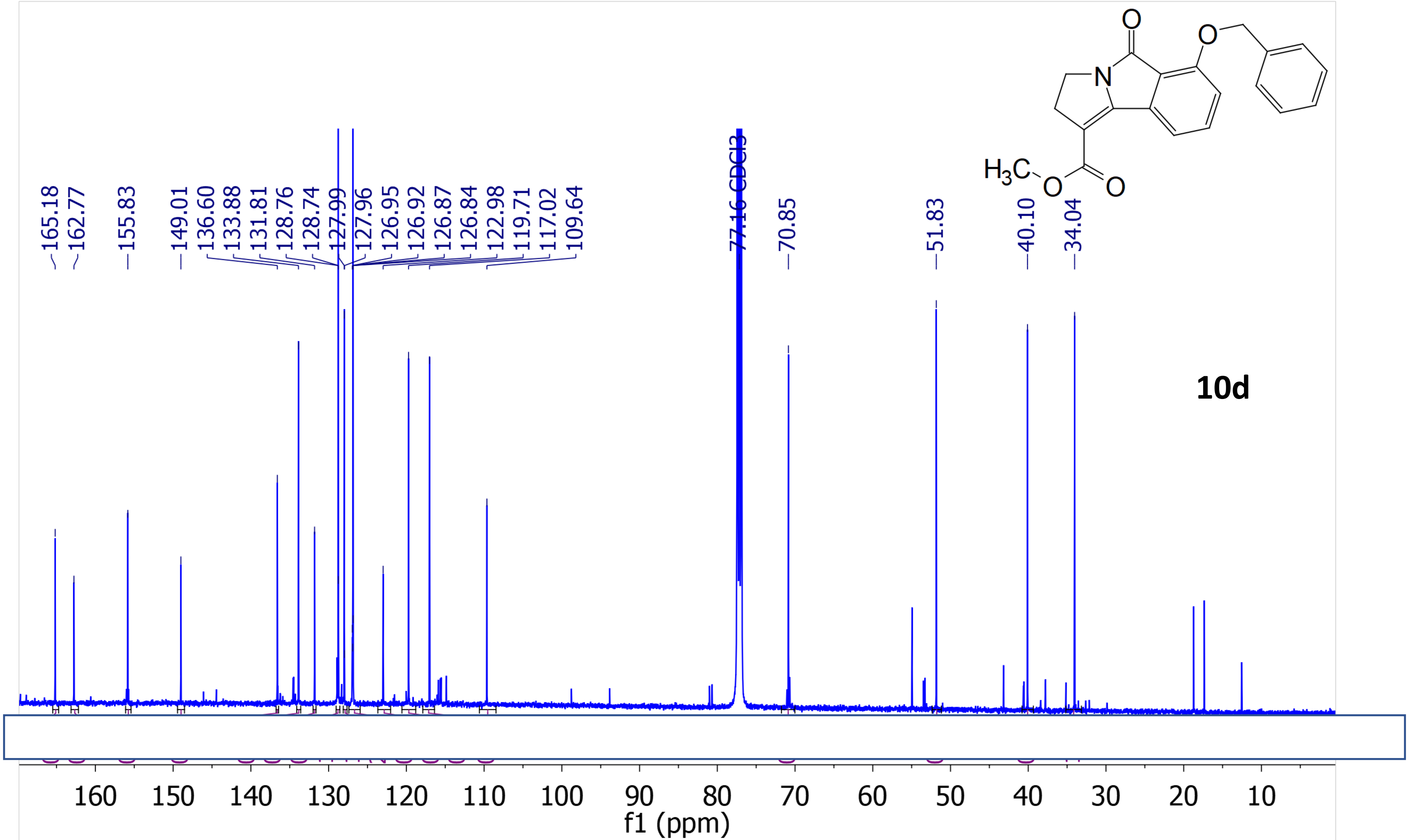

^{13}C NMR (126 MHz, $CDCl_3$) δ 165.18, 162.77, 155.83, 149.01, 136.60, 133.88, 131.81, 128.76, 128.74, 127.99, 127.96, 126.95, 126.92, 126.87, 126.84, 122.98, 119.71, 117.02, 109.64, 70.85, 51.83, 40.10, 34.04.

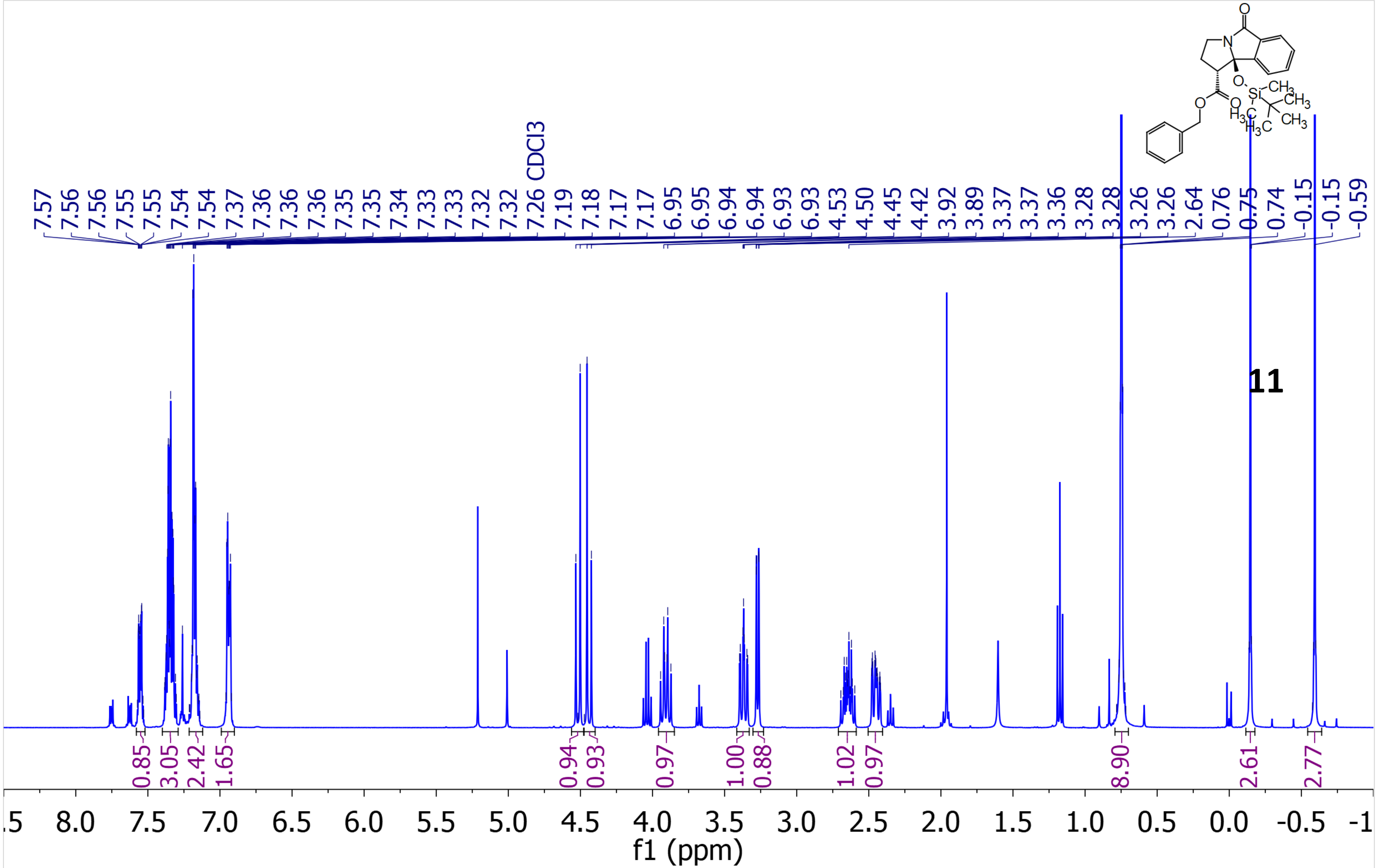

δ H (400 MHz, Chloroform-*d*) 7.58 – 7.52 (1 H, m), 7.40 – 7.29 (3 H, m), 7.21 – 7.12 (3 H, m), 6.99 – 6.90 (2 H, m), 4.52 (1 H, d, *J* 12.0), 4.44 (1 H, d, *J* 12.0), 3.91 (1 H, dt, *J* 11.0, 8.9), 3.37 (1 H, ddd, *J* 11.3, 9.5, 2.1), 3.27 (1 H, dd, *J* 6.9, 0.9), 2.65 (1 H, dtd, *J* 13.1, 9.3, 6.9), 2.45 (1 H, dddd, *J* 13.1, 8.6, 2.2, 0.9), 0.75 (9 H, s), -0.15 (3 H, s), -0.59 (3 H, s).

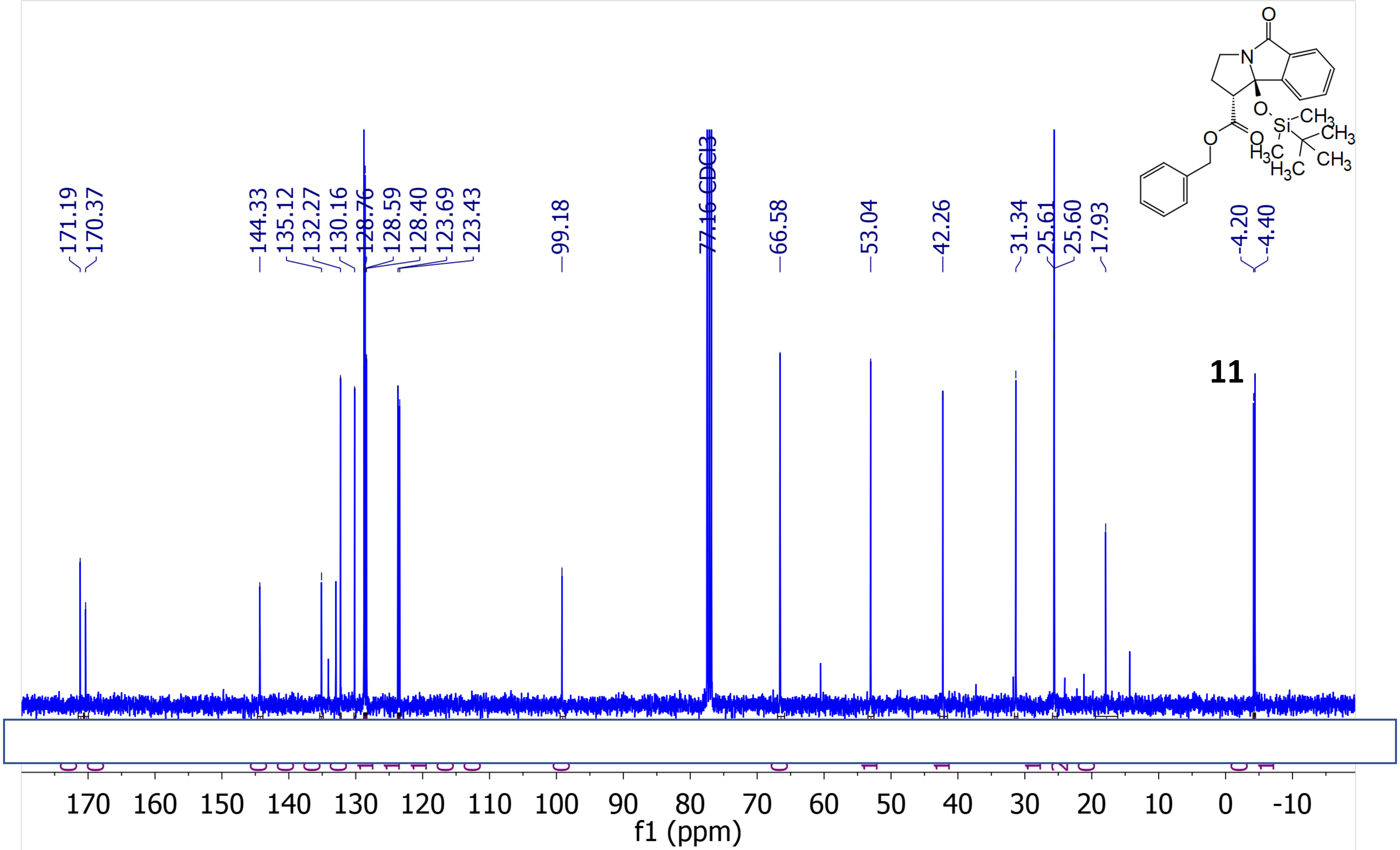

^{13}C NMR (101 MHz, Chloroform-*d*) δ 171.19, 170.37, 144.33, 135.12, 132.27, 130.16, 128.76, 128.59, 128.40, 123.69, 123.43, 99.18, 66.58, 53.04, 42.26, 31.34, 25.61, 25.60, 17.93, -4.20, -4.40.

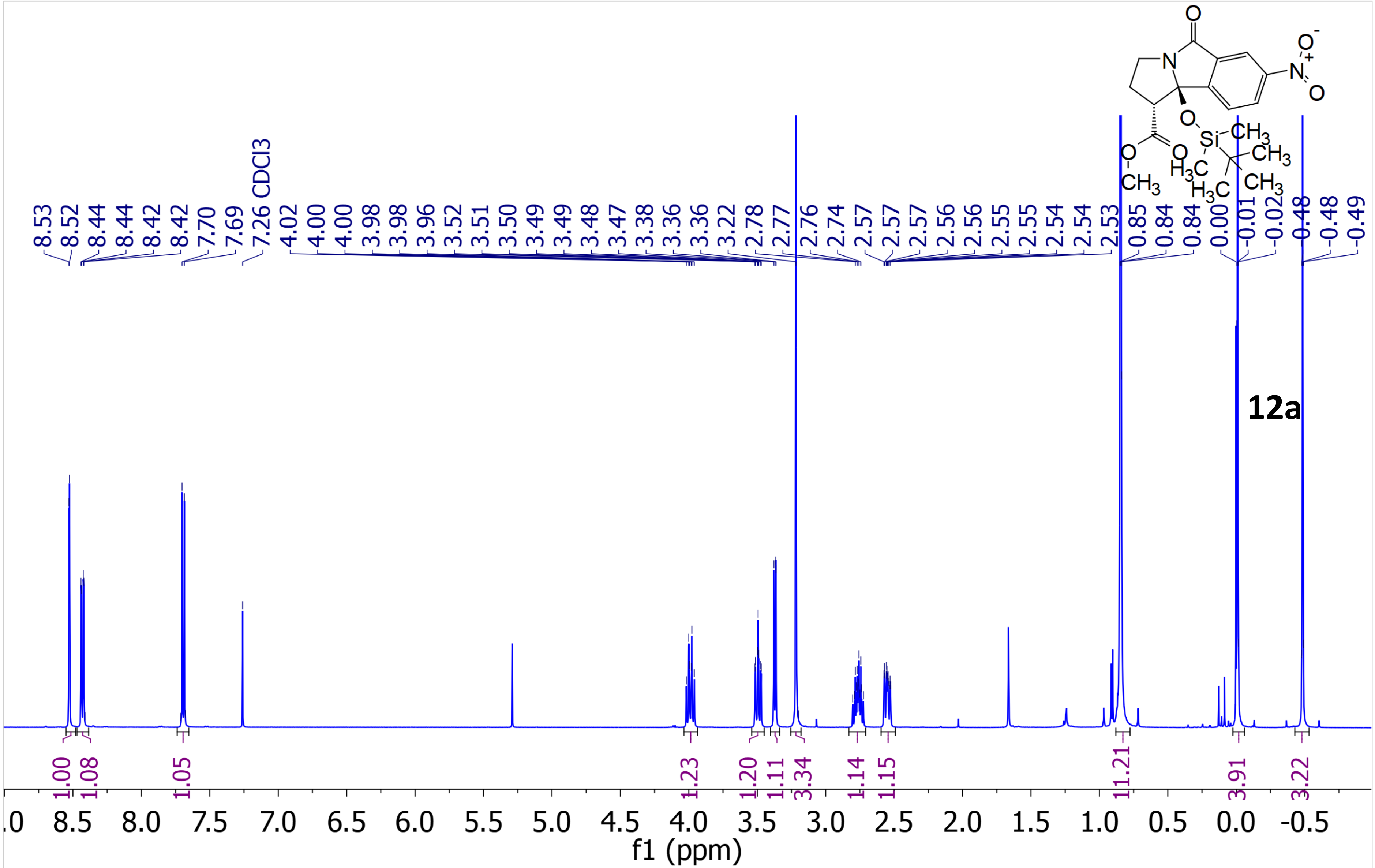

^{1}H NMR (500 MHz, Chloroform-*d*) δ 8.53 (d, *J* = 2.1 Hz, 1H), 8.43 (dd, *J* = 8.3, 2.1 Hz, 1H), 7.69 (d, *J* = 8.3 Hz, 1H), 3.99 (dt, *J* = 11.2, 8.8 Hz, 1H), 3.49 (ddd, *J* = 11.3, 9.4, 2.1 Hz, 1H), 3.40 – 3.34 (m, 1H), 3.22 (s, 3H), 2.76 (dtd, *J* = 13.2, 9.4, 7.1 Hz, 1H), 2.55 (ddt, *J* = 13.3, 8.2, 1.7 Hz, 1H), 0.85 (s, 9H), -0.00 (s, 3H), -0.48 (s, 3H).

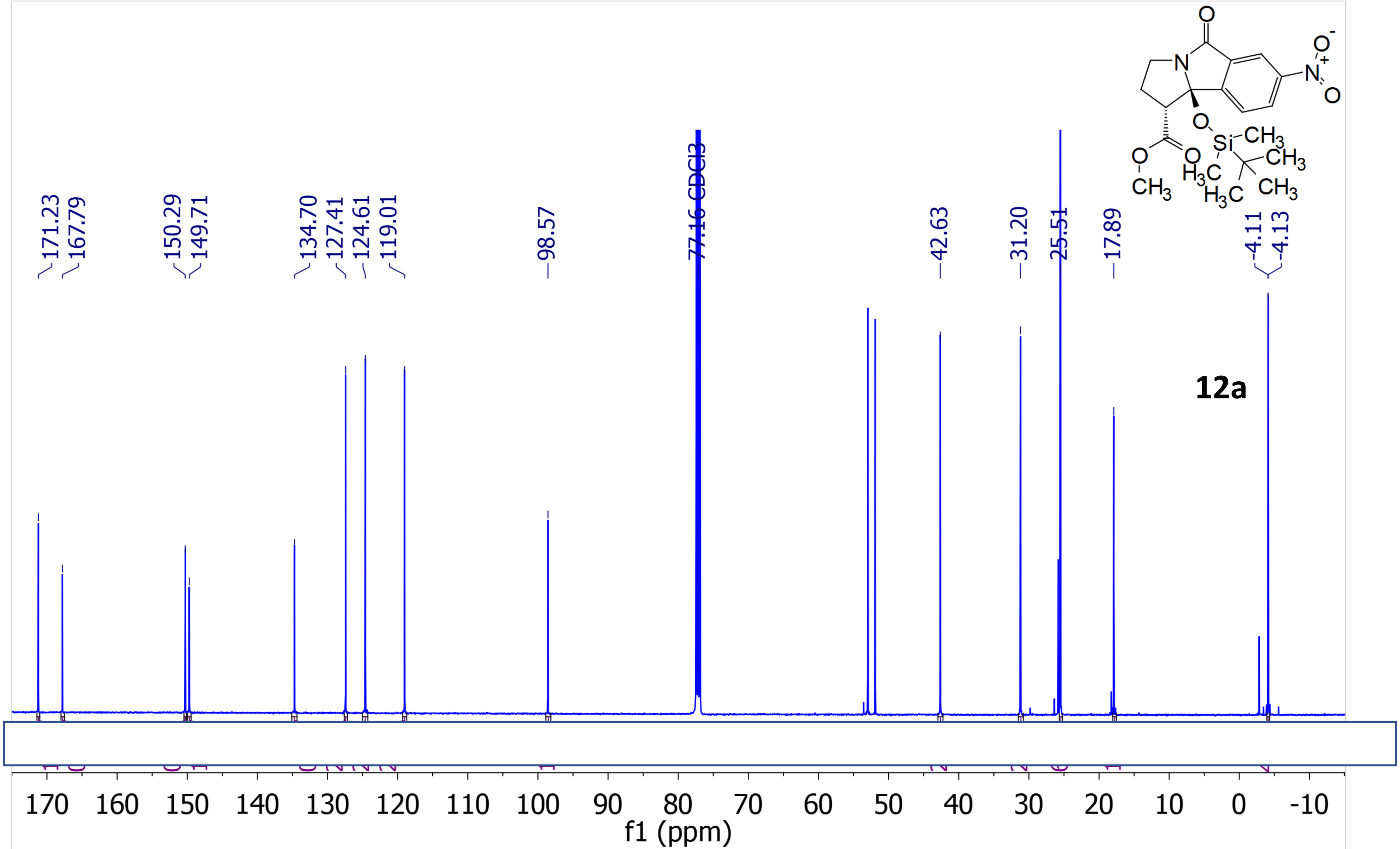

^{13}C NMR (126 MHz, $CDCl_3$) δ 171.23, 167.79, 150.29, 149.71, 134.70, 127.41, 124.61, 119.01, 98.57, 42.63, 31.20, 25.51, 17.89, -4.11, -4.13.

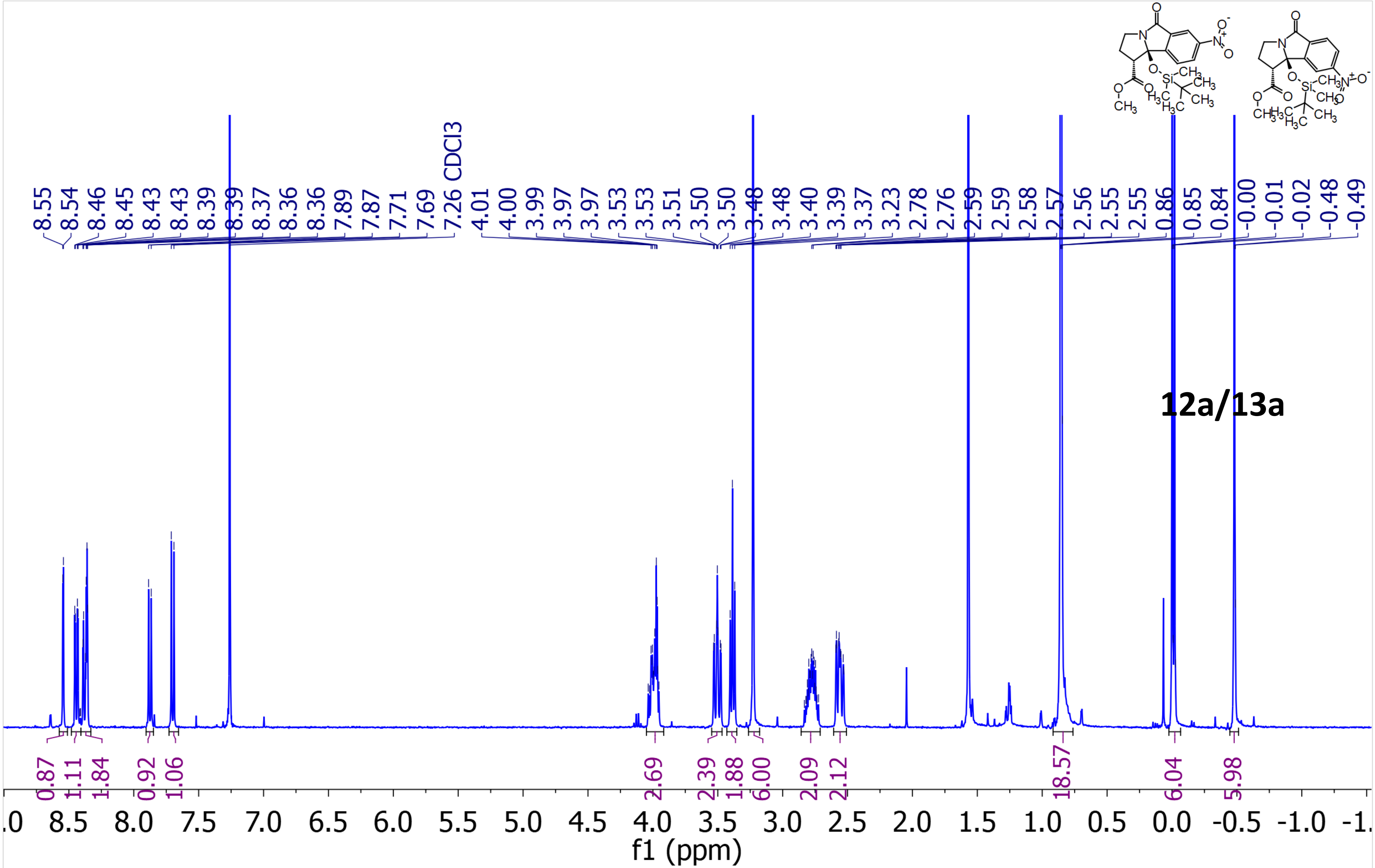

^{1}H NMR (400 MHz, Chloroform-*d*) δ 8.54 (d, *J* = 2.0 Hz, 1H), 8.44 (dd, *J* = 8.3, 2.1 Hz, 1H), 8.41 – 8.33 (m, 2H), 7.88 (d, *J* = 8.1 Hz, 1H), 7.70 (d, *J* = 8.2 Hz, 1H), 4.05 – 3.92 (m, 3H), 3.50 (ddd, *J* = 11.3, 9.4, 2.1 Hz, 2H), 3.39 (t, *J* = 7.1 Hz, 2H), 3.23 (s, 6H), 2.78 (dtdd, *J* = 13.8, 9.3, 7.0, 4.6 Hz, 2H), 2.61 – 2.51 (m, 2H), 0.86 (d, *J* = 2.3 Hz, 18H), -0.01 (d, *J* = 7.2 Hz, 6H), -0.48 (s, 6H).

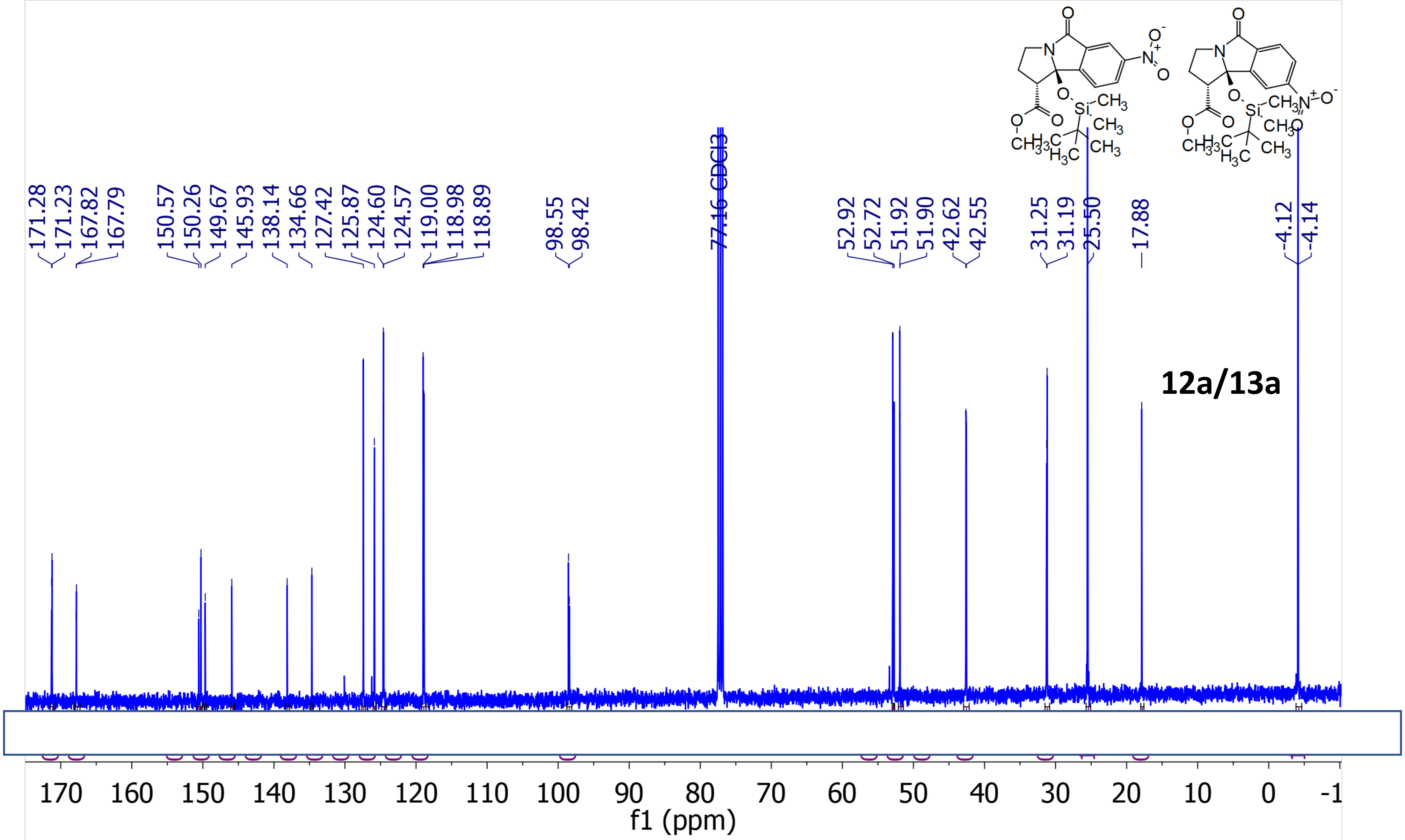

^{13}C NMR (101 MHz, $CDCl_3$) δ 171.28, 171.23, 167.82, 167.79, 150.57, 150.26, 149.67, 145.93, 138.14, 134.66, 127.42, 125.87, 124.60, 124.57, 119.00, 118.98, 118.89, 98.55, 98.42, 52.92, 52.72, 51.92, 51.90, 42.62, 42.55, 31.25, 31.19, 25.50, 17.88, -4.12, -4.14.

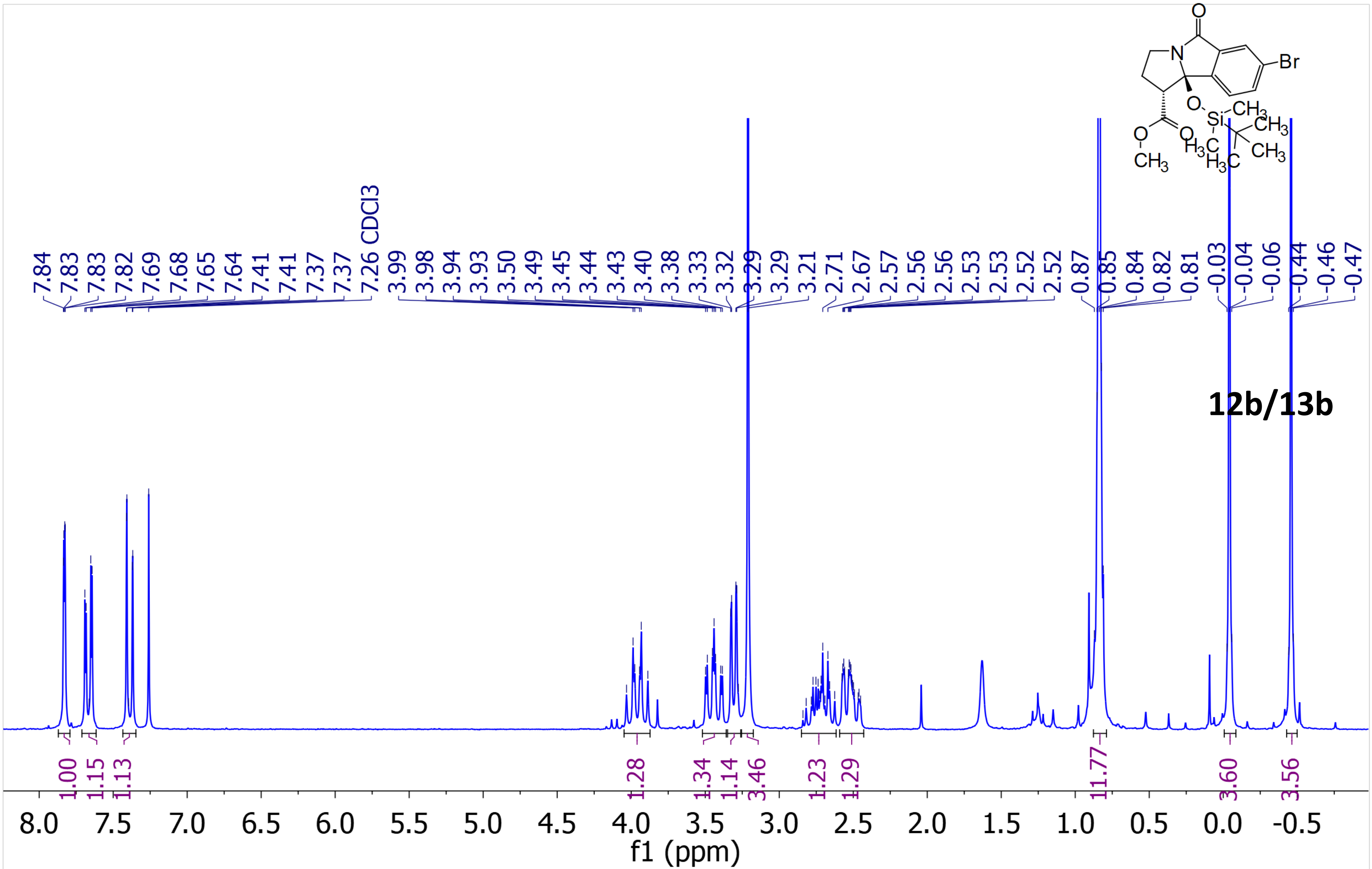

^{1}H NMR (200 MHz, Chloroform-*d*) δ 7.83 (dd, *J* = 1.8, 0.5 Hz, 1H), 7.67 (dd, *J* = 8.0, 1.8 Hz, 1H), 7.39 (dd, *J* = 8.0, 0.6 Hz, 1H), 3.96 (dt, *J* = 11.1, 8.8 Hz, 1H), 3.44 (ddd, *J* = 11.3, 9.3, 2.3 Hz, 1H), 3.31 (dd, *J* = 6.9, 1.1 Hz, 1H), 3.21 (s, 3H), 2.72 (dtd, *J* = 13.2, 9.2, 6.9 Hz, 1H), 2.52 (dddd, *J* = 13.0, 9.6, 2.9, 1.6 Hz, 1H), 0.84 (s, 9H), -0.04 (s, 3H), -0.46 (s, 3H).

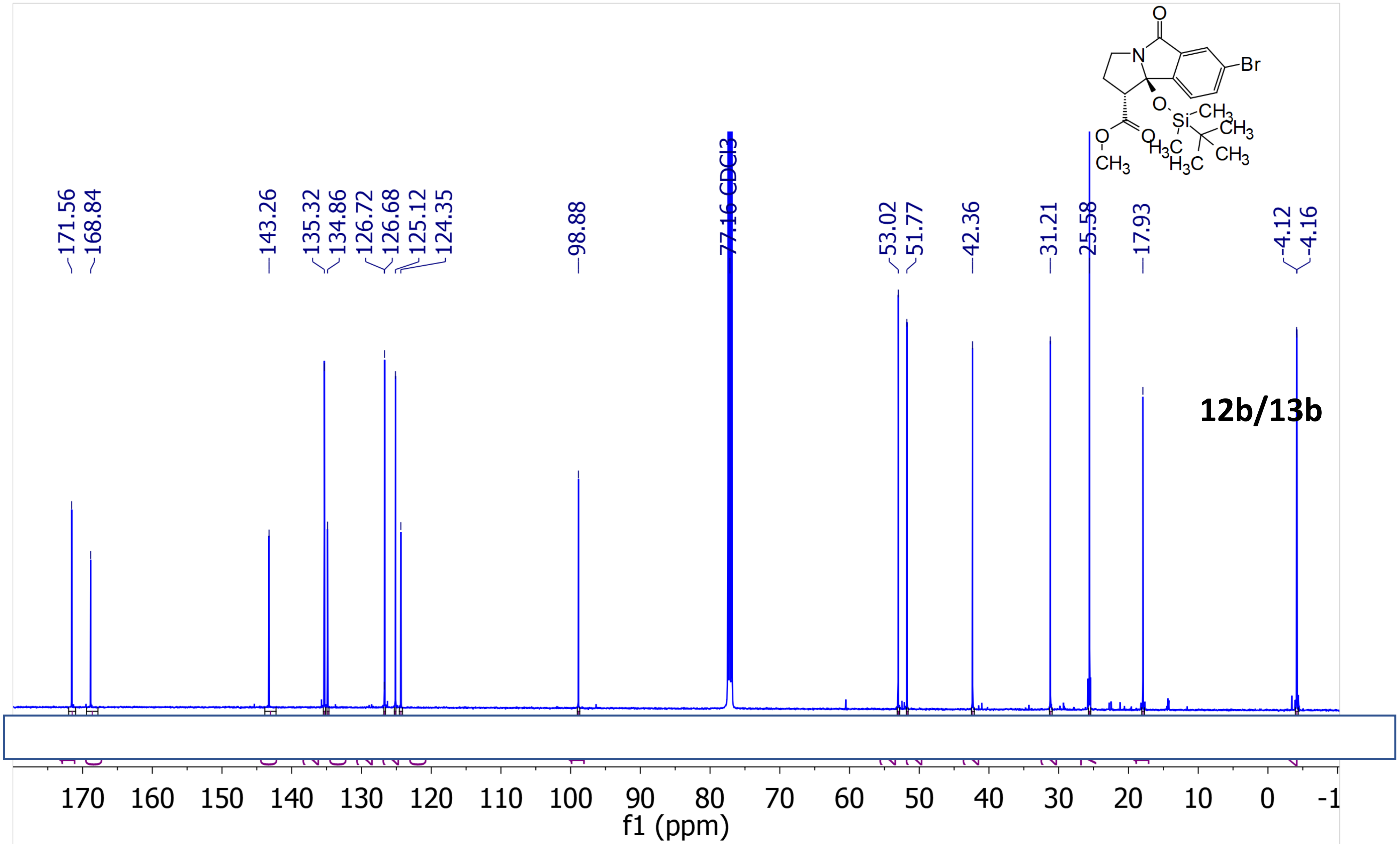

^{13}C NMR (126 MHz, $CDCl_3$) δ 171.56, 168.84, 143.26, 135.32, 134.86, 126.72, 126.68, 125.12, 124.35, 98.88, 53.02, 51.77, 42.36, 31.21, 25.58, 17.93, -4.12, -4.16.

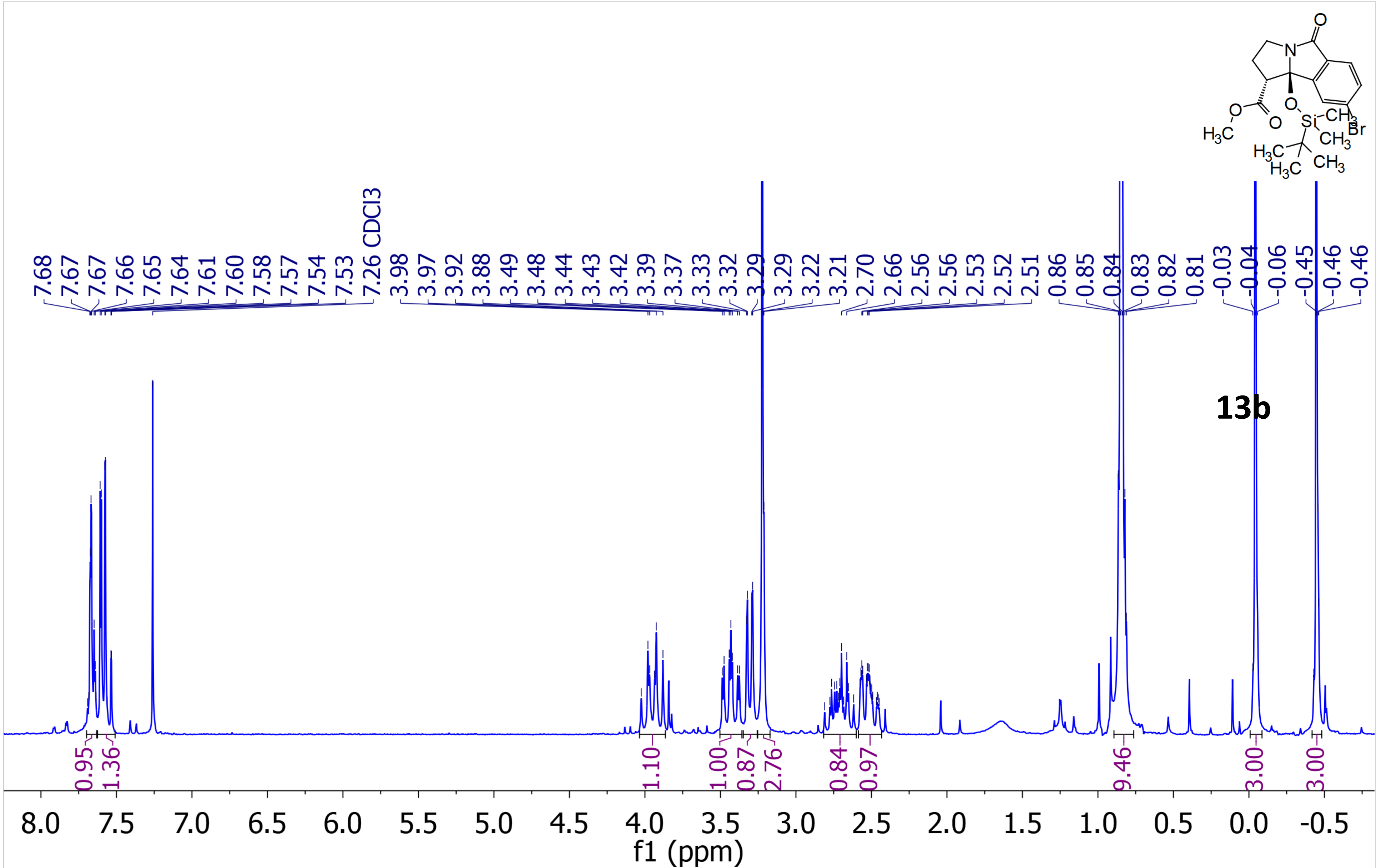

^{1}H NMR (200 MHz, Chloroform-*d*) δ 7.70 – 7.63 (m, 1H), 7.63 – 7.51 (m, 2H), 3.95 (dt, *J* = 11.2, 8.8 Hz, 1H), 3.43 (ddd, *J* = 11.3, 9.2, 2.4 Hz, 1H), 3.31 (dd, *J* = 6.9, 1.2 Hz, 1H), 3.22 (s, 3H), 2.71 (dtd, *J* = 13.2, 9.2, 7.0 Hz, 1H), 2.51 (dddd, *J* = 13.2, 8.6, 2.4, 1.2 Hz, 1H), 0.84 (s, 9H), -0.04 (s, 3H), -0.45 (s, 3H).

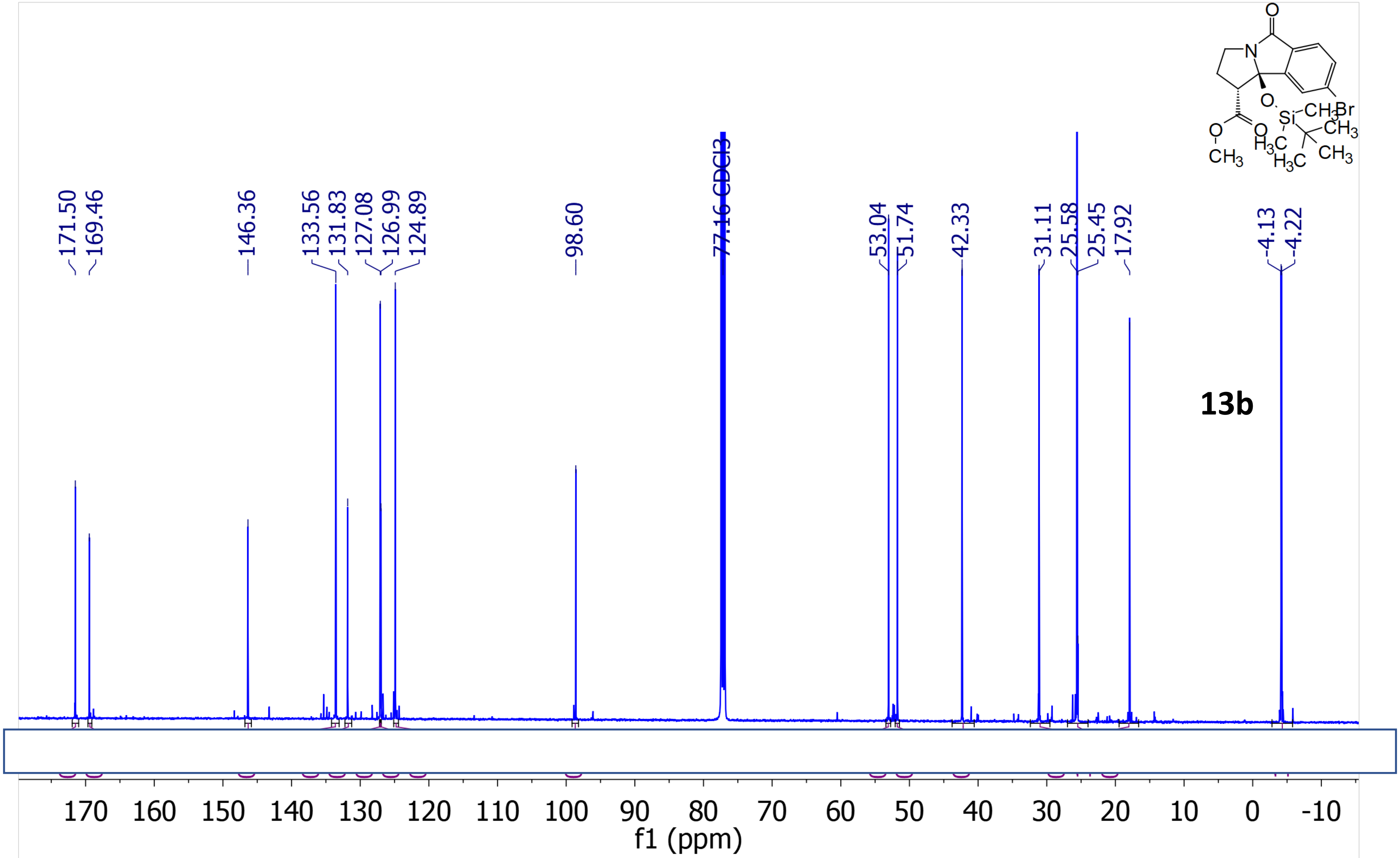

^{13}C NMR (126 MHz, $CDCl_3$) δ 171.50, 169.46, 146.36, 133.56, 131.83, 127.08, 126.99, 124.89, 98.60, 53.04, 51.74, 42.33, 31.11, 25.58, 25.45, 17.92, -4.13, -4.22.

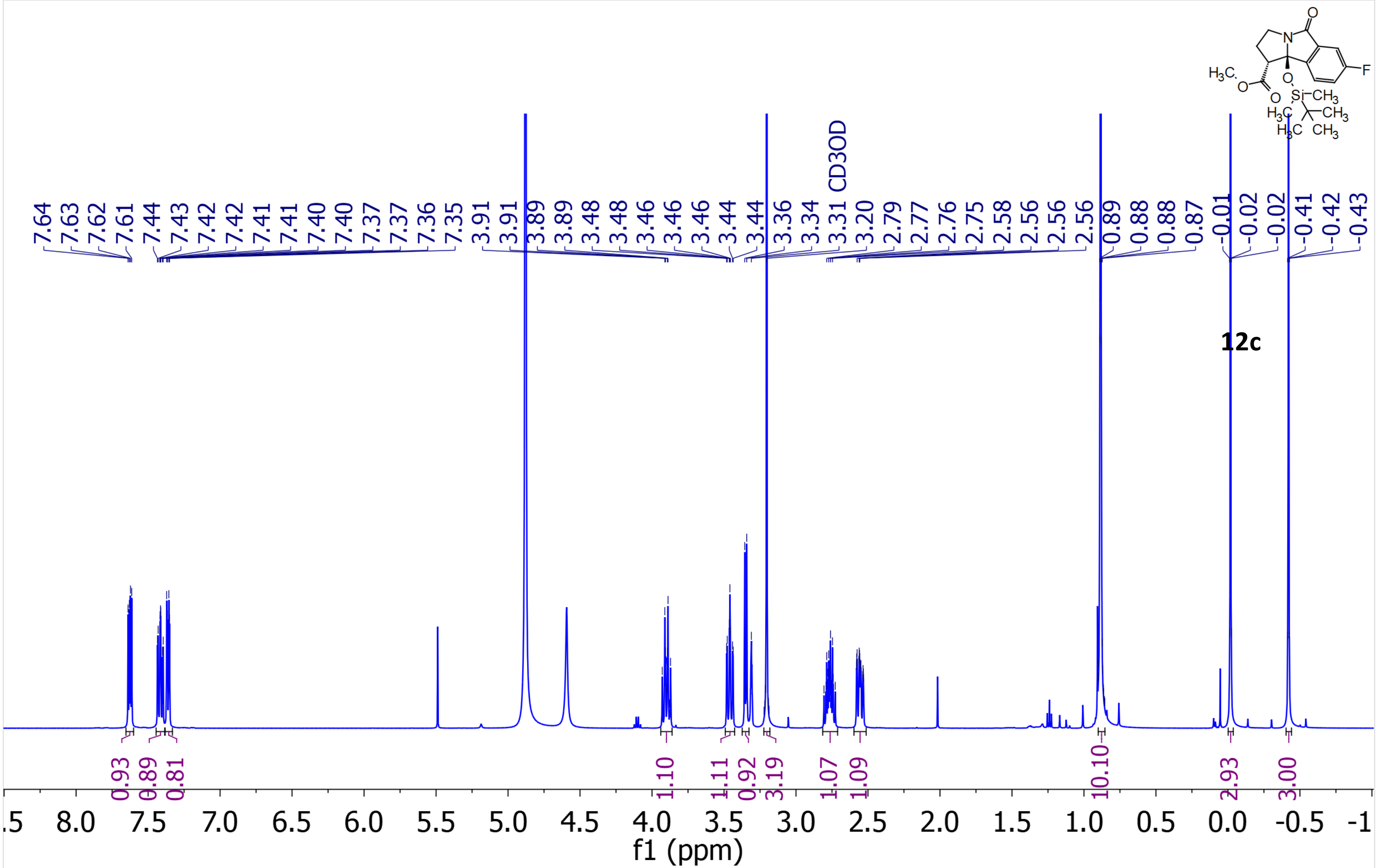

^{1}H NMR (500 MHz, Methanol-d_4) δ 7.63 (dd, J = 8.3, 4.5 Hz, 1H), 7.42 (ddd, J = 9.2, 8.3, 2.4 Hz, 1H), 7.36 (dd, J = 7.6, 2.4 Hz, 1H), 3.90 (dt, J = 11.1, 8.9 Hz, 1H), 3.46 (ddd, J = 11.4, 9.6, 2.1 Hz, 1H), 3.35 (d, J = 6.9 Hz, 1H), 3.20 (s, 3H), 2.77 (dtd, J = 13.3, 9.4, 7.0 Hz, 1H), 2.56 (dddd, J = 13.3, 8.5, 2.1, 0.9 Hz, 1H), 0.88 (s, 10H), -0.02 (s, 3H), -0.42 (s, 3H).

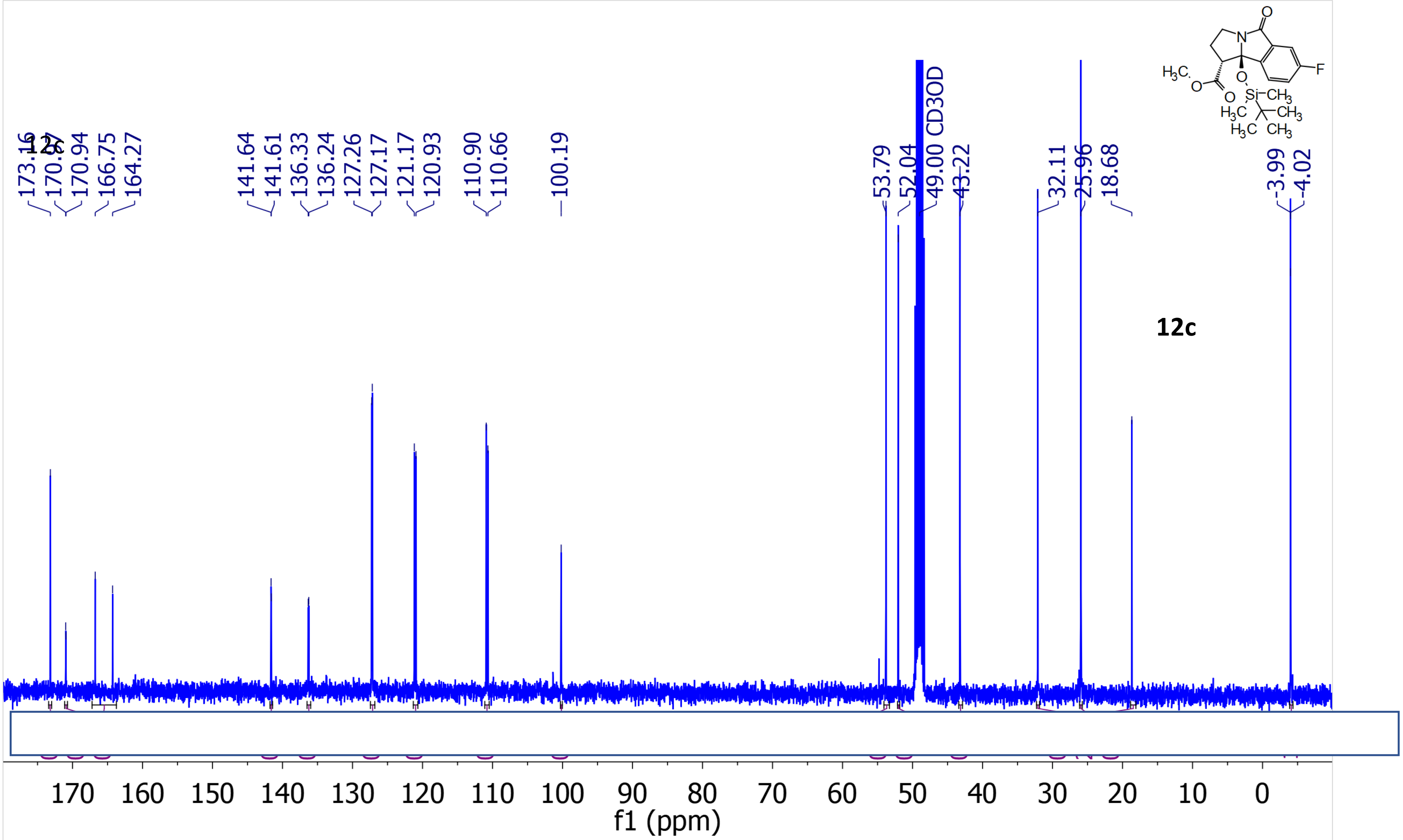

^{13}C NMR (101 MHz, MeOD) δ 173.16, 170.97, 170.94, 166.75, 164.27, 141.64, 141.61, 136.33, 136.24, 127.26, 127.17, 121.17, 120.93, 110.90, 110.66, 100.19, 53.79, 52.04, 49.00, 43.22, 32.11, 25.96, 18.68, -3.99, -4.02.

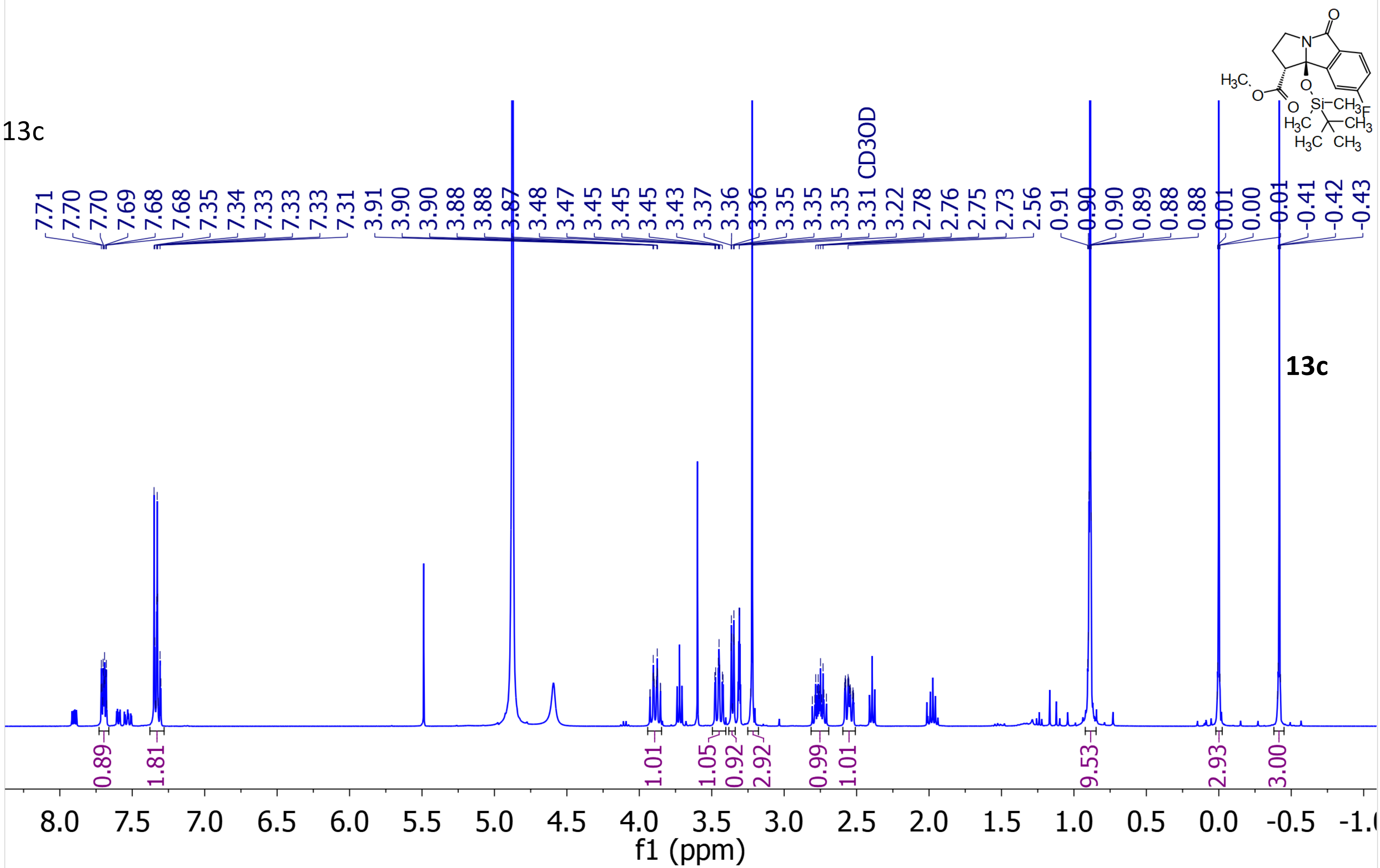

^{1}H NMR (400 MHz, Methanol-d_4) δ 7.73 – 7.66 (m, 1H), 7.38 – 7.28 (m, 2H), 3.89 (dt, J = 11.1, 8.5 Hz, 1H), 3.45 (ddd, J = 11.3, 9.5, 2.1 Hz, 1H), 3.36 (d, J = 6.9 Hz, 1H), 3.22 (s, 3H), 2.76 (dtd, J = 13.3, 9.4, 7.0 Hz, 1H), 2.55 (dddd, J = 13.3, 8.5, 2.1, 1.0 Hz, 1H), 0.89 (s, 10H), 0.00 (s, 3H), -0.42 (s, 3H).

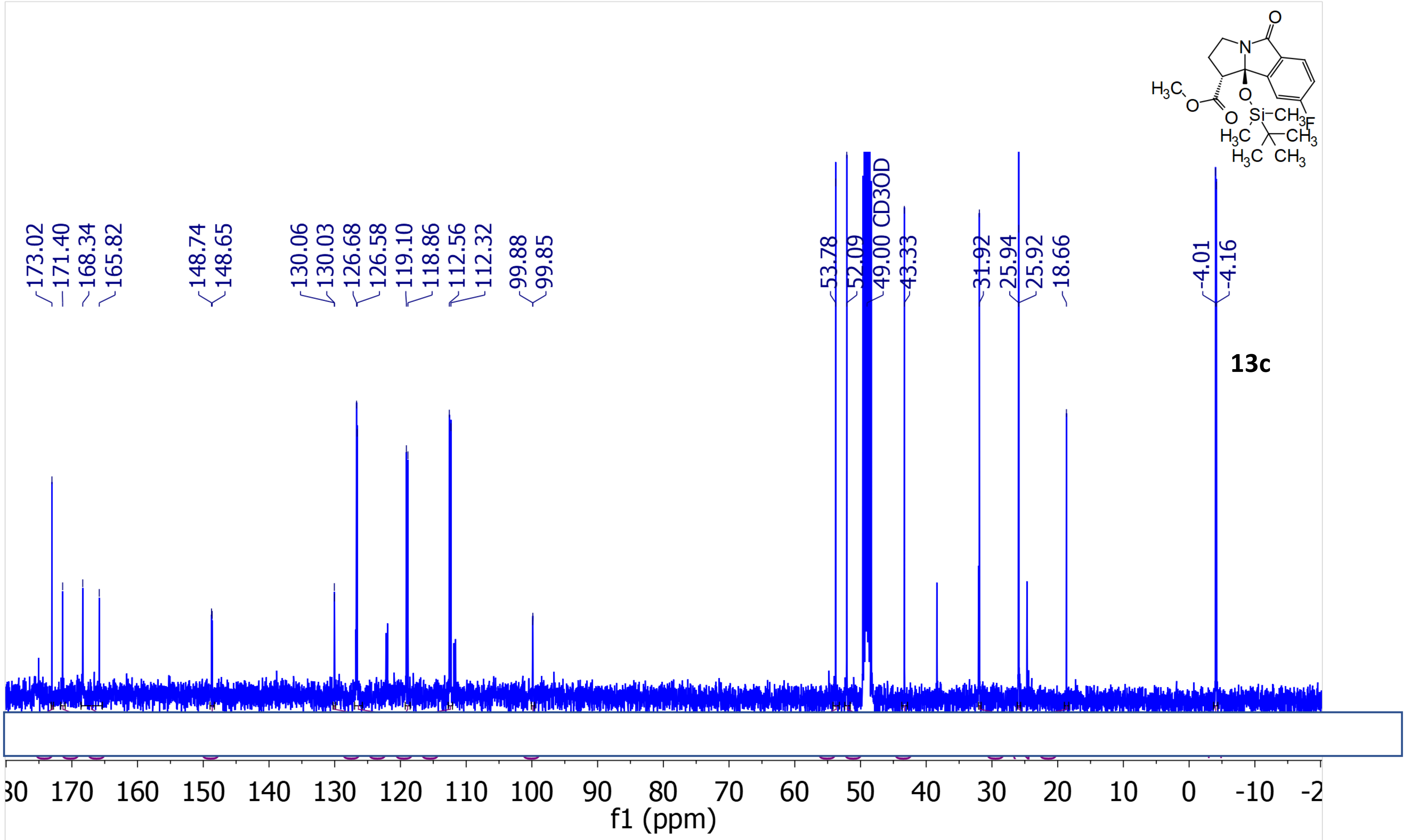

^{13}C NMR (101 MHz, MeOD) δ 173.02, 171.40, 168.34, 165.82, 148.74, 148.65, 130.06, 130.03, 126.68, 126.58, 119.10, 118.86, 112.56, 112.32, 99.88, 99.85, 53.78, 52.09, 49.00, 43.33, 31.92, 25.94, 25.92, 18.66, -4.01, -4.16.

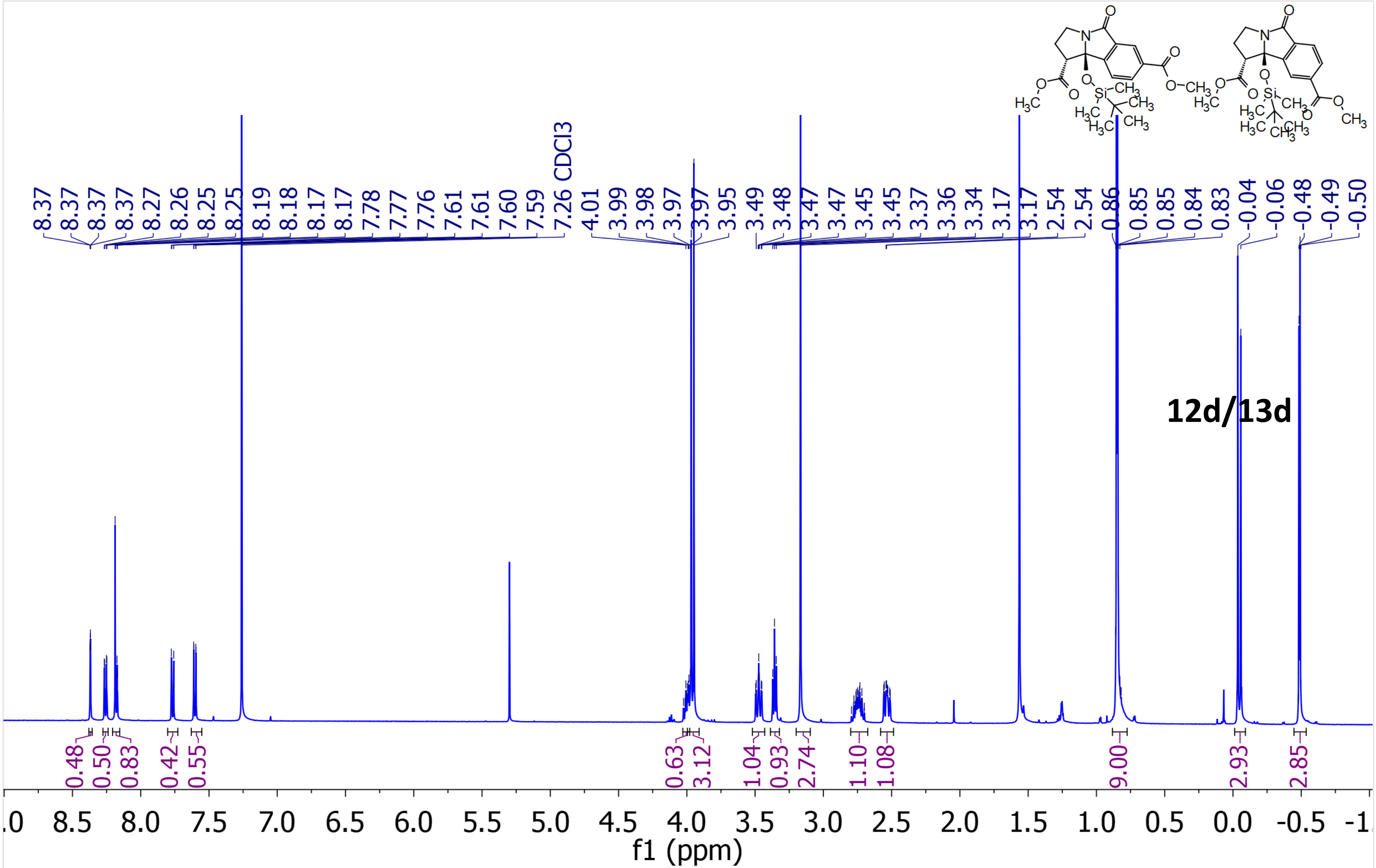

^{1}H NMR (500 MHz, Chloroform-*d*) δ 8.37 (dd, *J* = 1.5, 0.7 Hz, 0H), 8.26 (dd, *J* = 7.9, 1.5 Hz, 1H), 8.18 (d, *J* = 7.2 Hz, 1H), 7.80 – 7.73 (m, 0H), 7.60 (dd, *J* = 7.9, 0.7 Hz, 1H), 4.03 – 3.98 (m, 1H), 3.96 (d, *J* = 9.9 Hz, 3H), 3.47 (ddd, *J* = 11.4, 9.4, 2.2 Hz, 1H), 3.36 (t, *J* = 6.6 Hz, 1H), 3.17 (d, *J* = 0.8 Hz, 3H), 2.75 (dqd, *J* = 13.1, 9.1, 7.3 Hz, 1H), 2.53 (ddd, *J* = 13.3, 8.6, 2.1 Hz, 1H), 0.85 (d, *J* = 3.5 Hz, 9H), -0.05 (d, *J* = 10.8 Hz, 3H), -0.49 (d, *J* = 3.9 Hz, 3H).

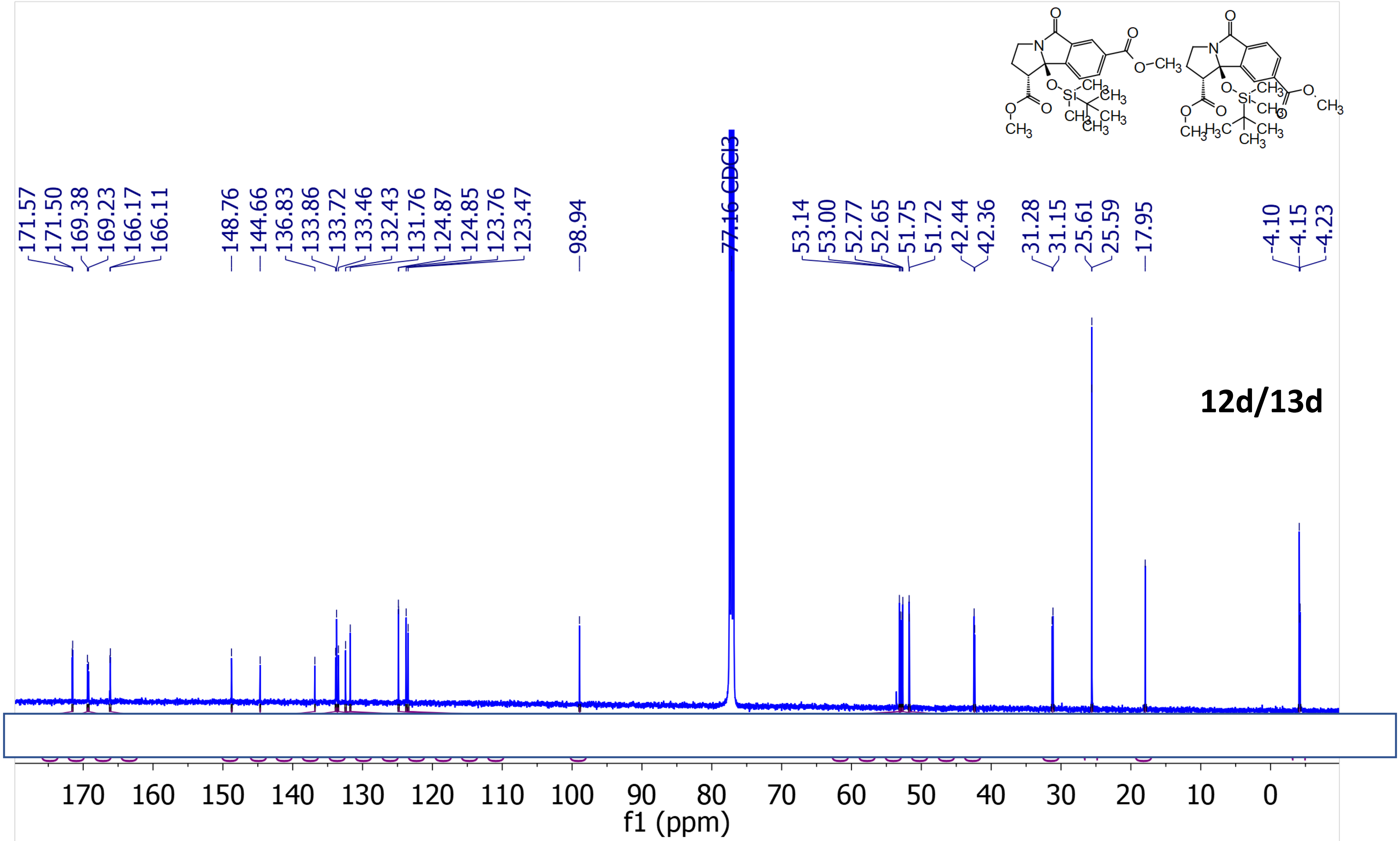

^{13}C NMR (126 MHz, $CDCl_3$) δ 171.57, 171.50, 169.38, 169.23, 166.17, 166.11, 148.76, 144.66, 136.83, 133.86, 133.72, 133.46, 132.43, 131.76, 124.87, 124.85, 123.76, 123.47, 98.94, 53.14, 53.00, 52.77, 52.65, 51.75, 51.72, 42.44, 42.36, 31.28, 31.15, 25.61, 25.59, 17.95, -4.10, -4.15, -4.23.

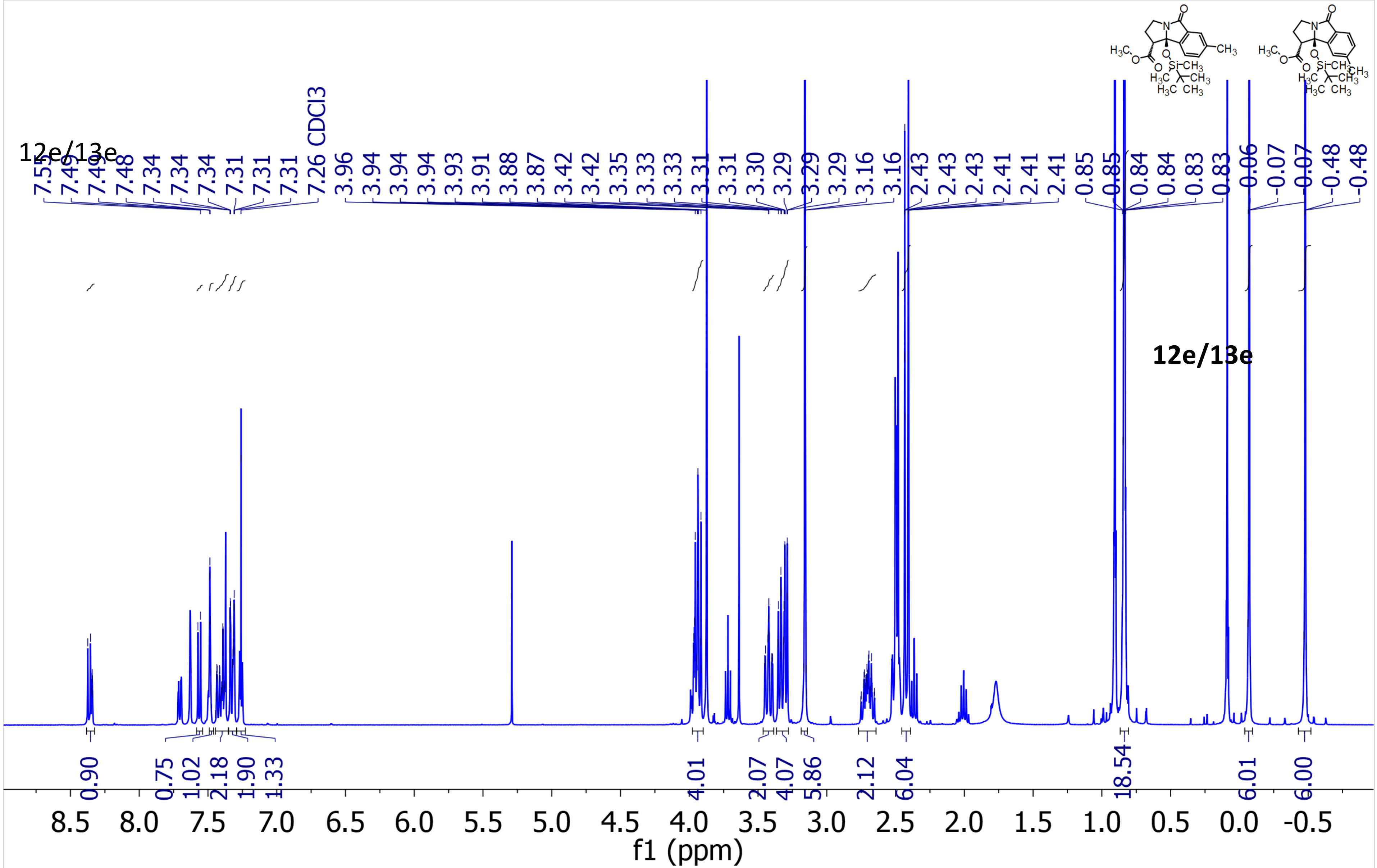

^{1}H NMR (400 MHz, Chloroform-*d*) δ 8.39 – 8.33 (m, 1H), 7.56 (d, *J* = 7.7 Hz, 1H), 7.49 (dq, *J* = 1.7, 0.8 Hz, 1H), 7.45 – 7.36 (m, 1H), 7.35 – 7.29 (m, 1H), 7.26 (s, 1H), 3.98 – 3.90 (m, 2H), 3.42 (dddd, *J* = 11.7, 9.4, 2.3, 0.8 Hz, 2H), 3.36 – 3.27 (m, 4H), 3.16 (d, *J* = 1.2 Hz, 6H), 2.70 (dtdd, *J* = 13.1, 9.3, 7.1, 1.3 Hz, 2H), 2.43 (d, *J* = 0.9 Hz, 3H), 2.41 (d, *J* = 0.7 Hz, 3H), 0.84 (d, *J* = 1.6 Hz, 18H), -0.07 (d, *J* = 0.9 Hz, 6H), -0.48 (d, *J* = 1.2 Hz, 6H).

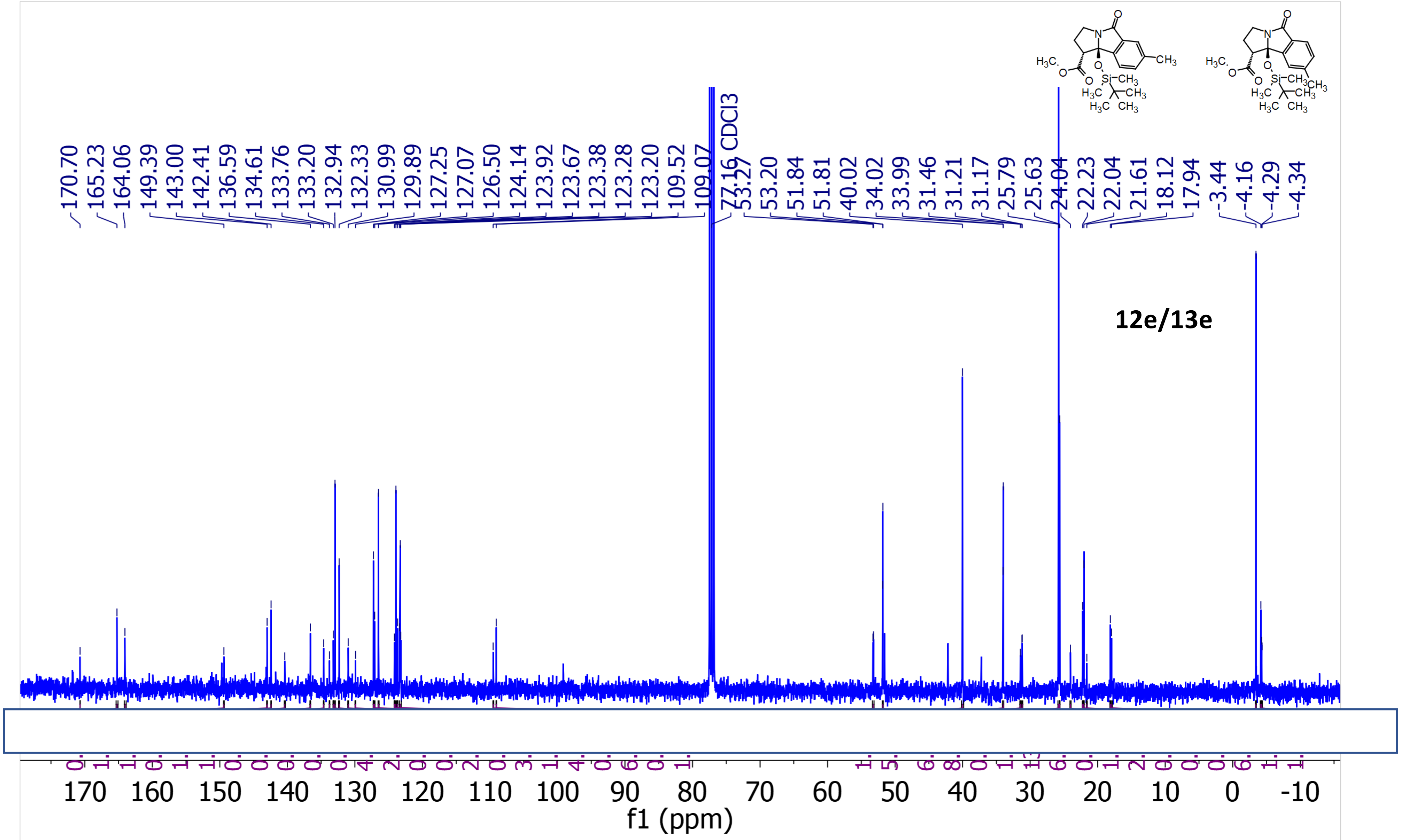

^{13}C NMR (101 MHz, $CDCl_3$) δ 170.70, 165.23, 164.06, 149.39, 143.00, 142.41, 140.38, 136.59, 134.61, 133.76, 133.20, 132.94, 132.33, 130.99, 129.89, 127.25, 127.07, 126.50, 124.14, 123.92, 123.67, 123.38, 123.28, 123.20, 109.52, 109.07, 77.16, 53.27, 53.20, 51.84, 51.81, 40.02, 34.02, 33.99, 31.46, 31.21, 31.17, 25.79, 25.63, 24.04, 22.23, 22.04, 21.61, 18.12, 17.94, -3.44, -4.16, -4.29, -4.34.

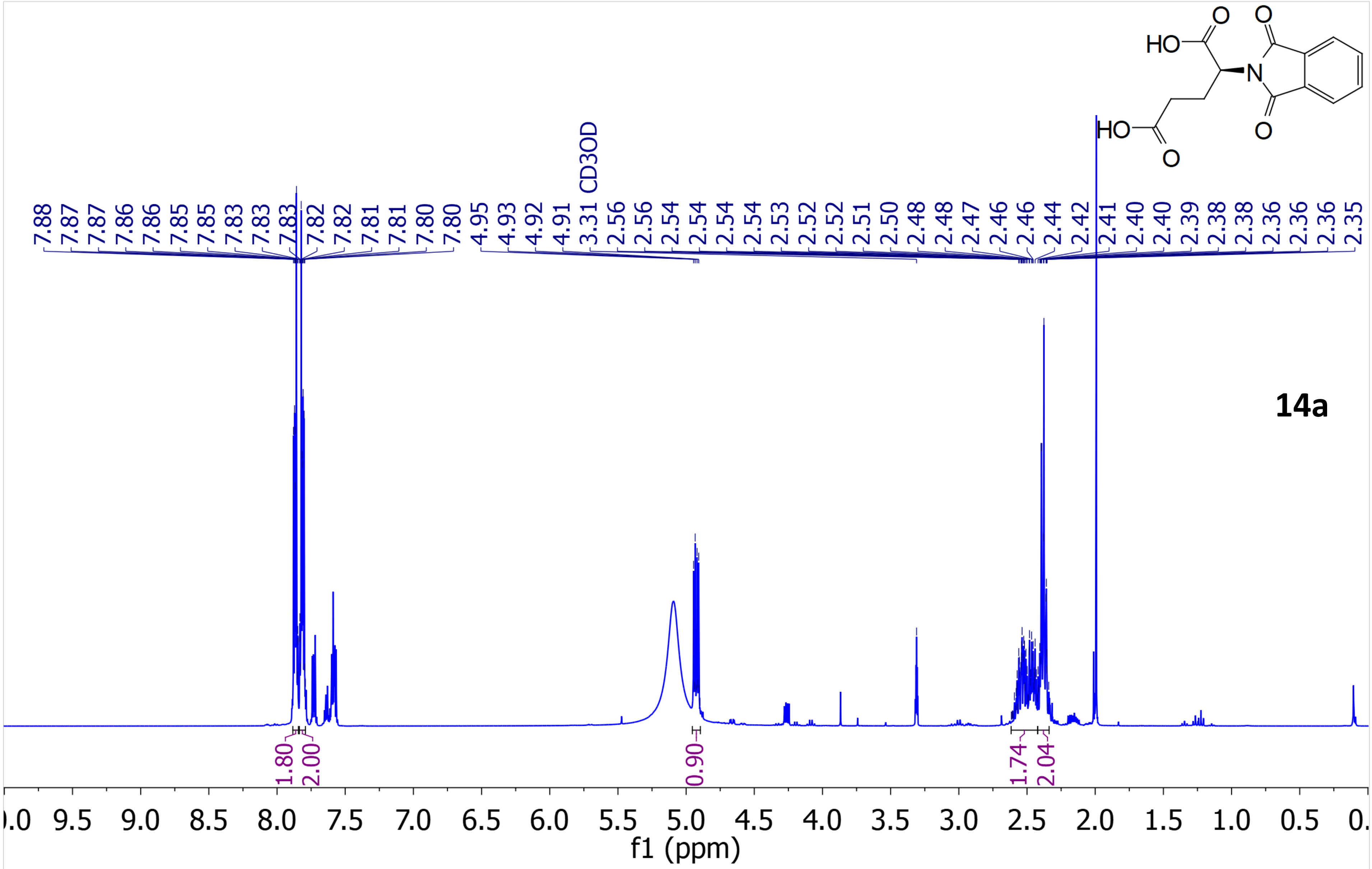

^{1}H NMR (400 MHz, Methanol-d_4) δ 7.86 (dd, J = 8.5, 5.7 Hz, 2H), 7.81 (dd, J = 8.5, 5.3 Hz, 2H), 4.93 (dd, J = 10.3, 4.8 Hz, 1H), 2.62 – 2.42 (m, 2H), 2.42 – 2.34 (m, 2H).

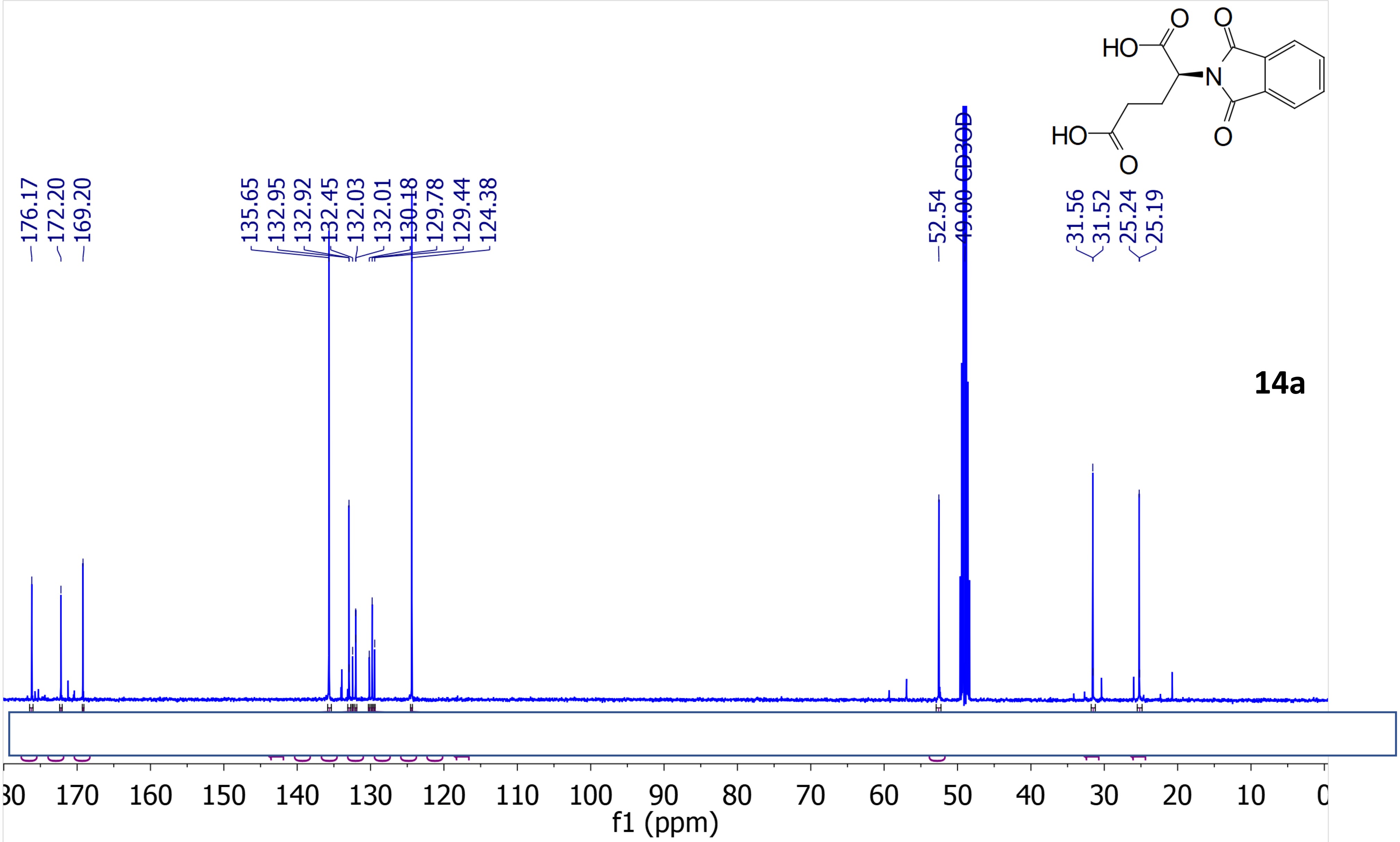

^{13}C NMR (101 MHz, MeOD) δ 176.17, 172.20, 169.20, 135.65, 132.95, 132.92, 132.45, 132.03, 132.01, 130.18, 129.78, 129.44, 124.38, 52.54, 31.56, 31.52, 25.24, 25.19.

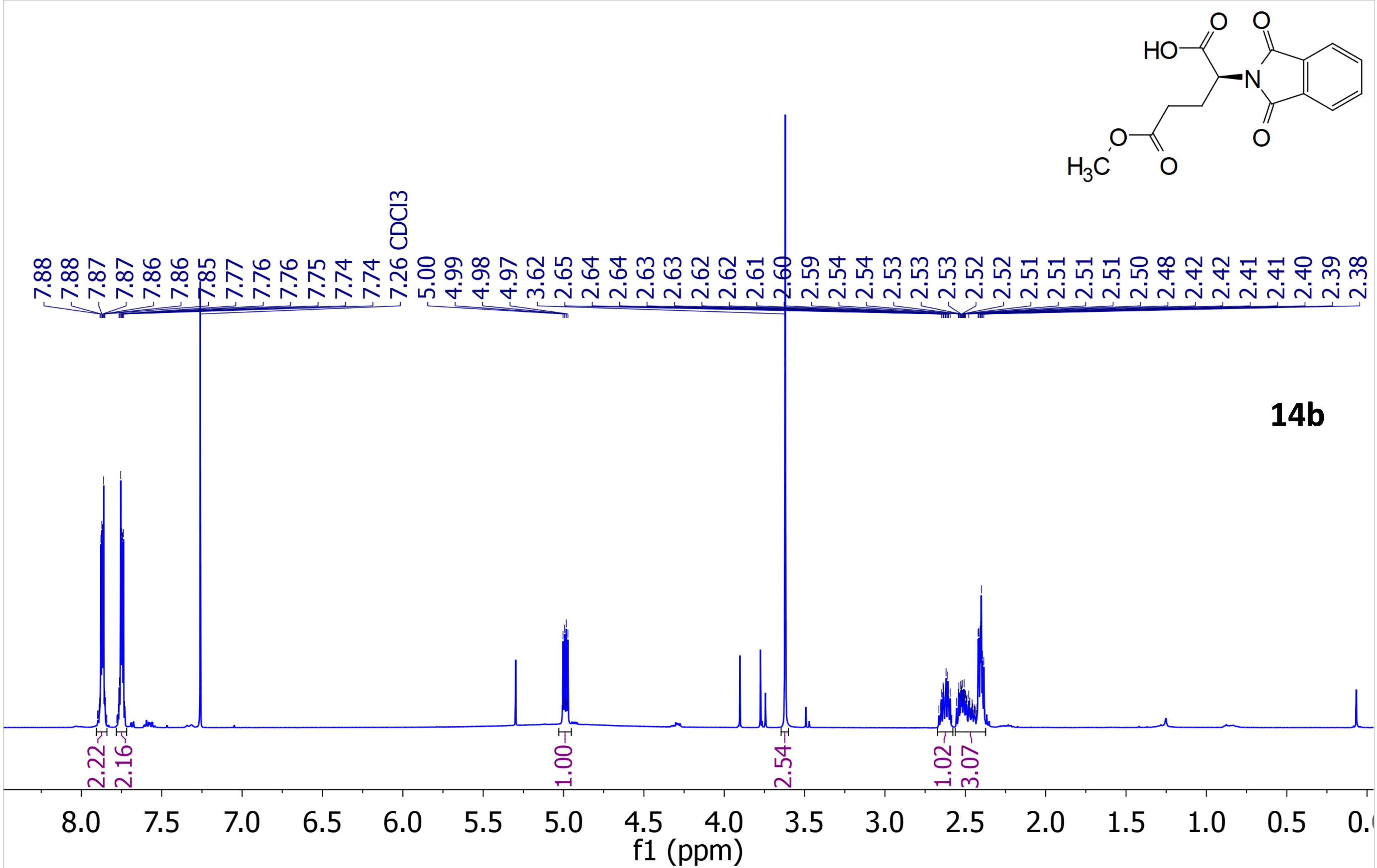

14b

^{1}H NMR (500 MHz, Chloroform-*d*) δ 7.89 (dd, 2H), 7.78 (dd, 2H), 5.01 (dd, *J* = 10.3, 5.0 Hz, 1H), 3.65 (s, 3H), 2.70 – 2.60 (m, 1H), 2.59 – 2.40 (m, 3H).

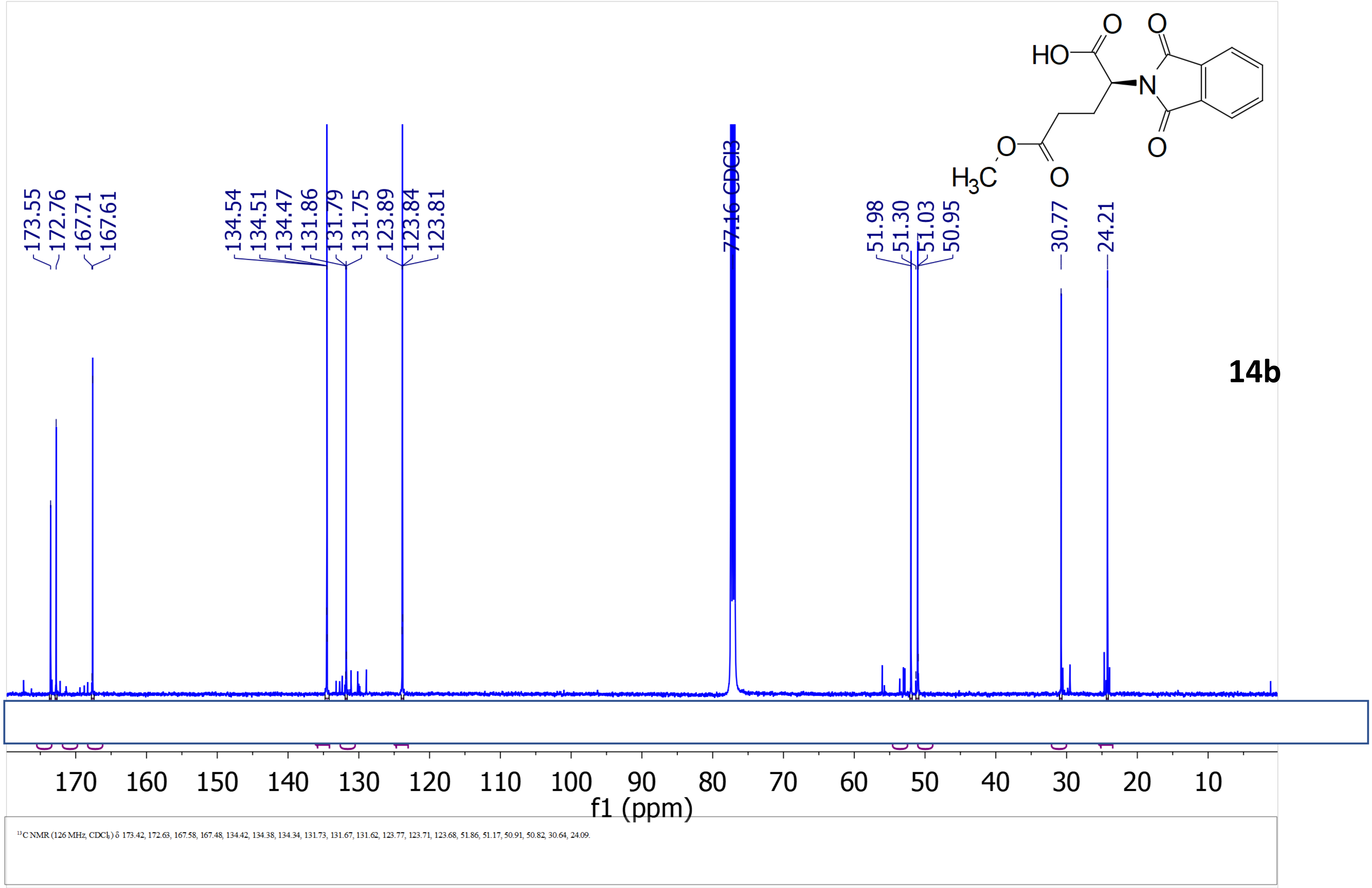

14b

^{13}C NMR (126 MHz, $CDCl_3$) δ 173.42, 172.63, 167.58, 167.48, 134.42, 134.38, 134.34, 131.73, 131.67, 131.62, 123.77, 123.71, 123.68, 51.86, 51.17, 50.91, 50.82, 30.64, 24.09.

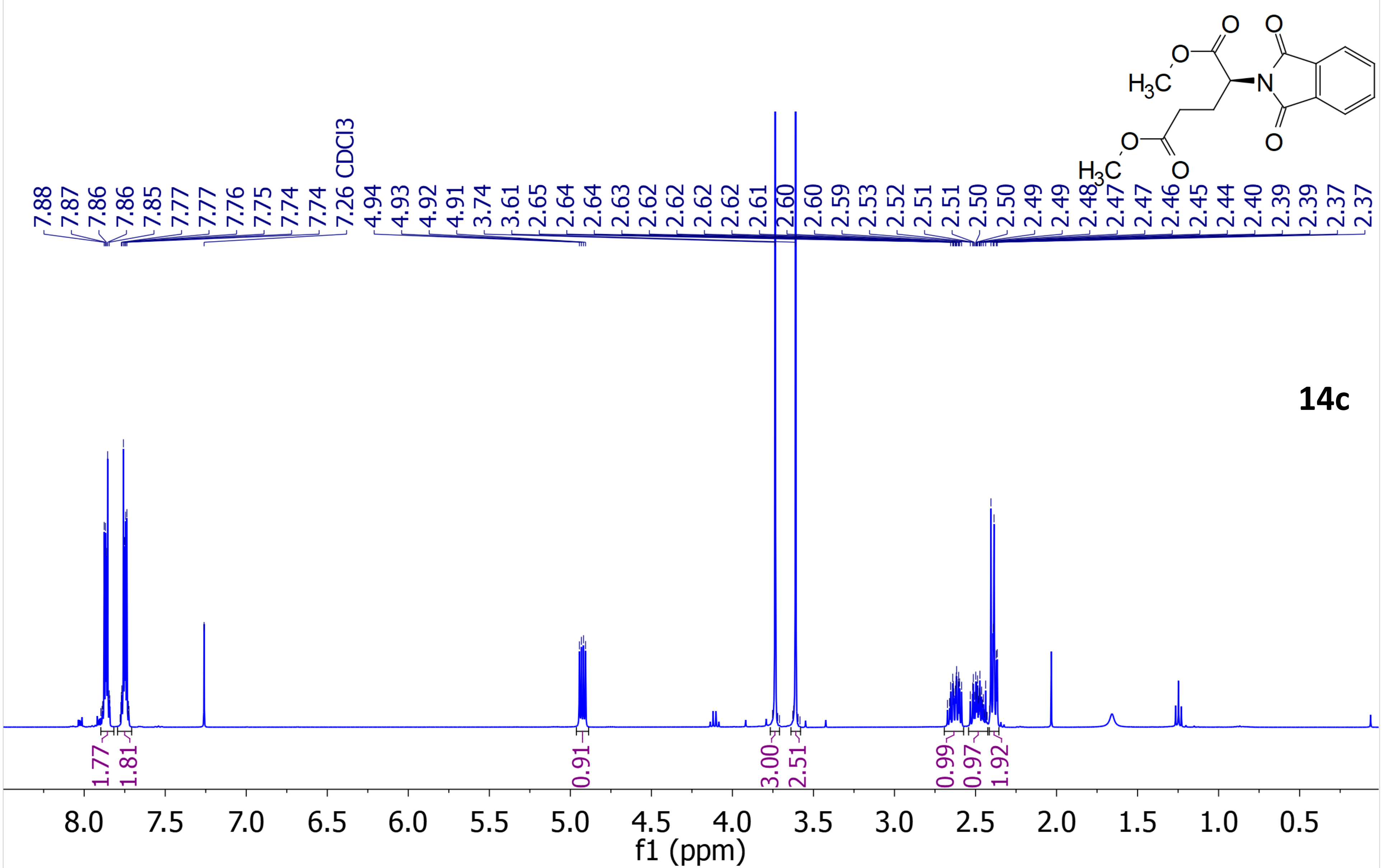

^{1}H NMR (400 MHz, Chloroform-*d*) δ 7.87 (dd, *J* = 5.4,3.1 Hz, 2H), 7.75 (dd, *J* = 5.3, 3.1 Hz, 2H), 4.93 (dd, *J* = 10.2, 5.0 Hz, 1H), 3.74 (s, 3H), 3.61 (s, 3H), 2.69 – 2.57 (m, 1H), 2.48 (dddd, *J* = 14.1, 10.0, 7.6, 5.8 Hz, 1H), 2.42 – 2.36 (m, 2H).

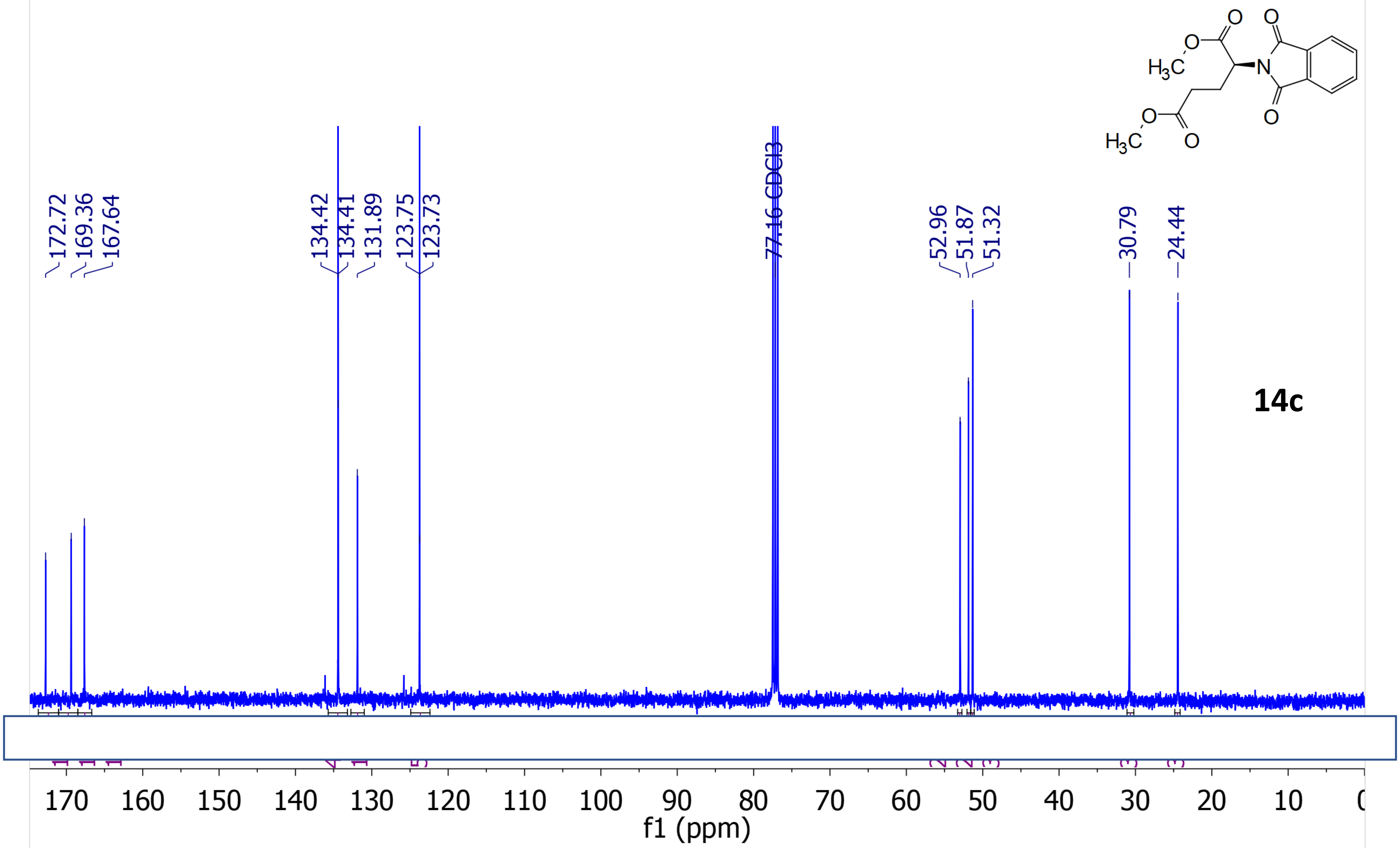

^{13}C NMR (101 MHz, $CDCl_3$) δ 172.72, 169.36, 167.64, 134.42, 134.41, 131.89, 123.75, 123.73, 52.96, 51.87, 51.32, 30.79, 24.44.

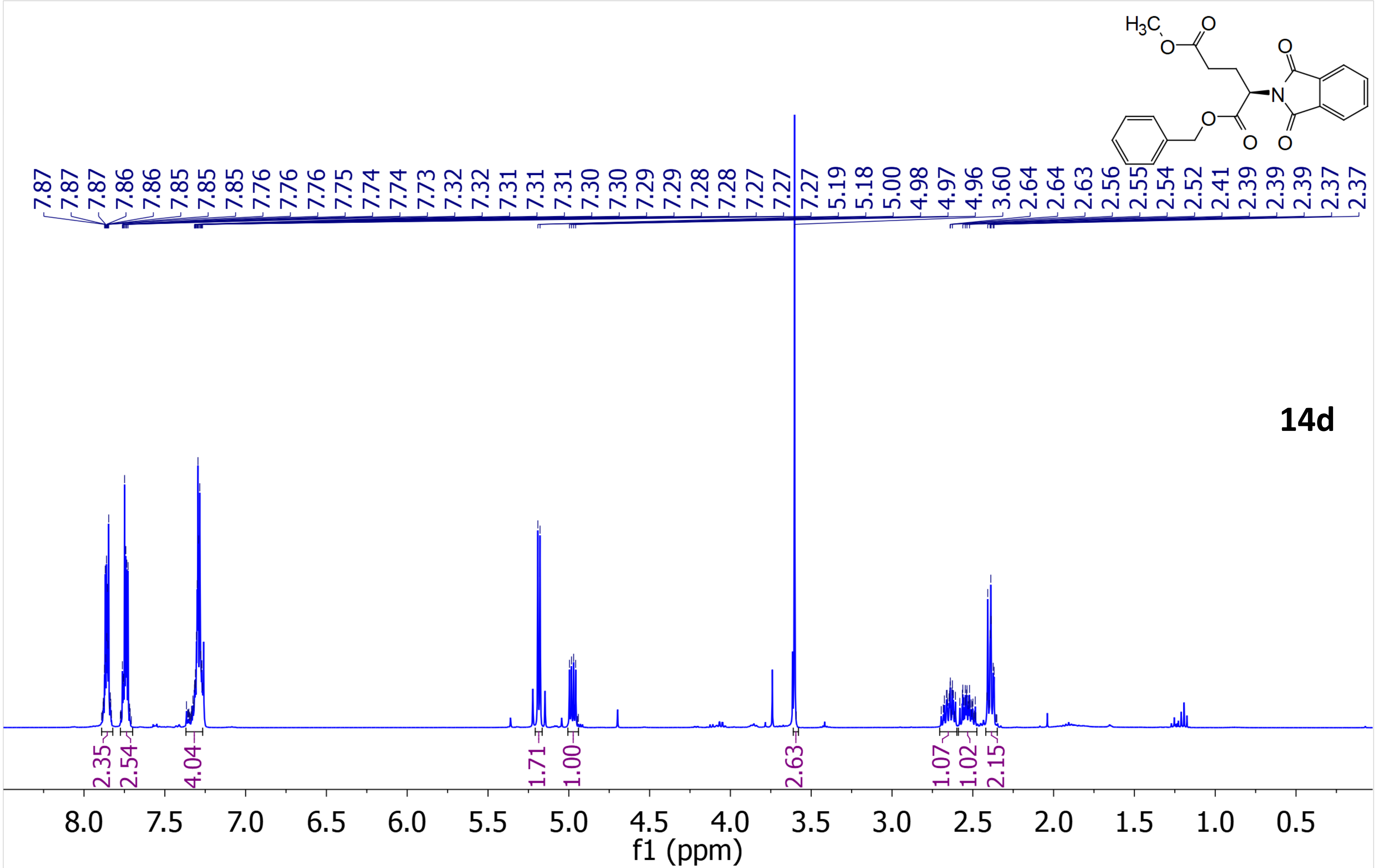

14d

^{1}H NMR (400 MHz, Chloroform-*d*) δ 7.86 (ddd, *J* = 5.4, 3.0, 1.8 Hz, 2H), 7.75 (td, *J* = 5.5, 3.0 Hz, 3H), 7.37 – 7.27 (m, 4H), 5.18 (d, *J* = 5.5 Hz, 2H), 4.98 (dd, *J* = 10.3, 5.1 Hz, 1H), 3.60 (s, 3H), 2.70 – 2.60 (m, 1H), 2.53 (dddd, *J* = 14.3, 10.3, 7.4, 6.1 Hz, 1H), 2.42 – 2.35 (m, 2H).

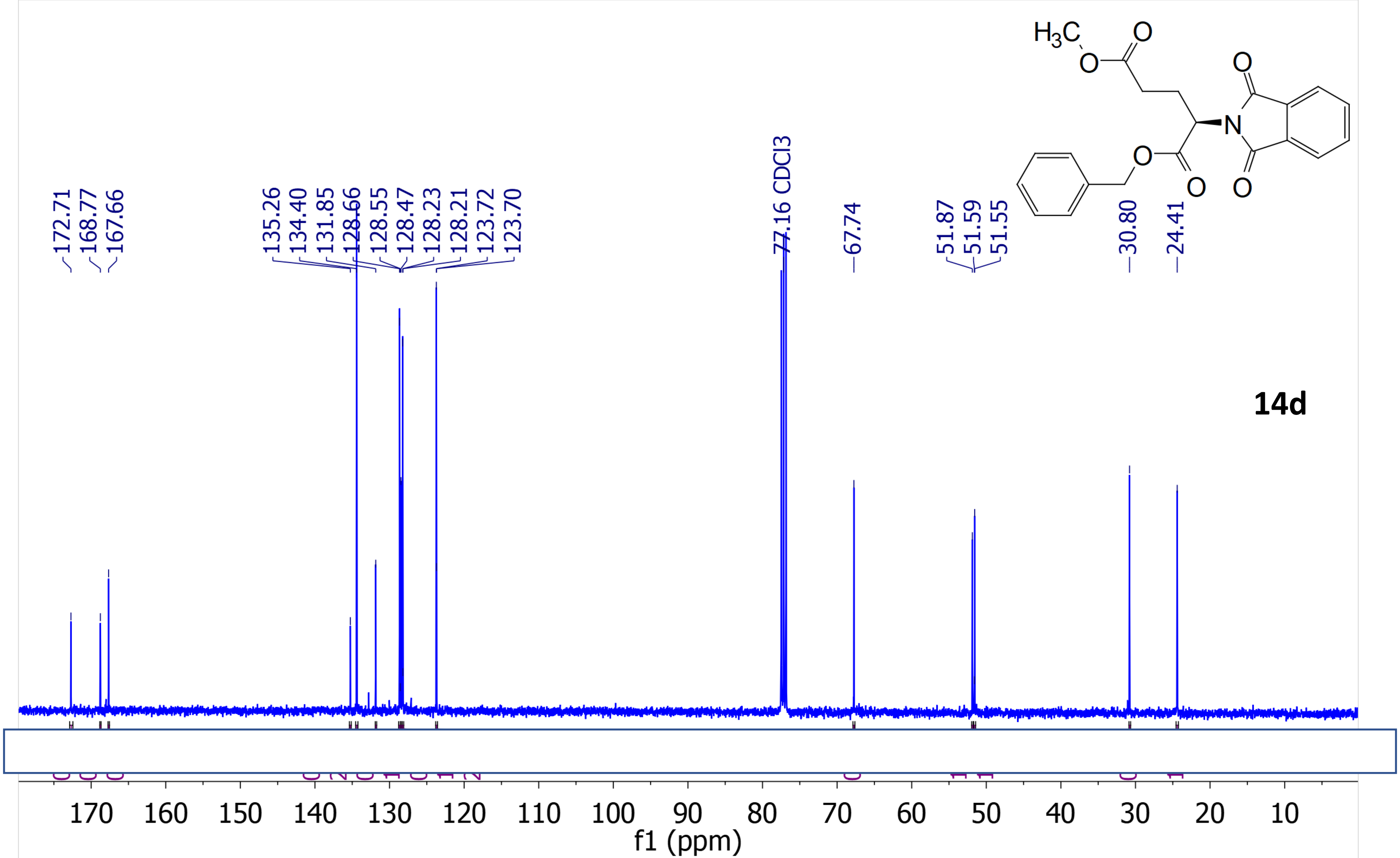

^{13}C NMR (101 MHz, Chloroform-*d*) δ 172.71, 168.77, 167.66, 135.26, 134.40, 131.85, 128.66, 128.55, 128.47, 128.23, 128.21, 123.72, 123.70, 67.74, 51.87, 51.59, 51.55, 30.80, 24.41.

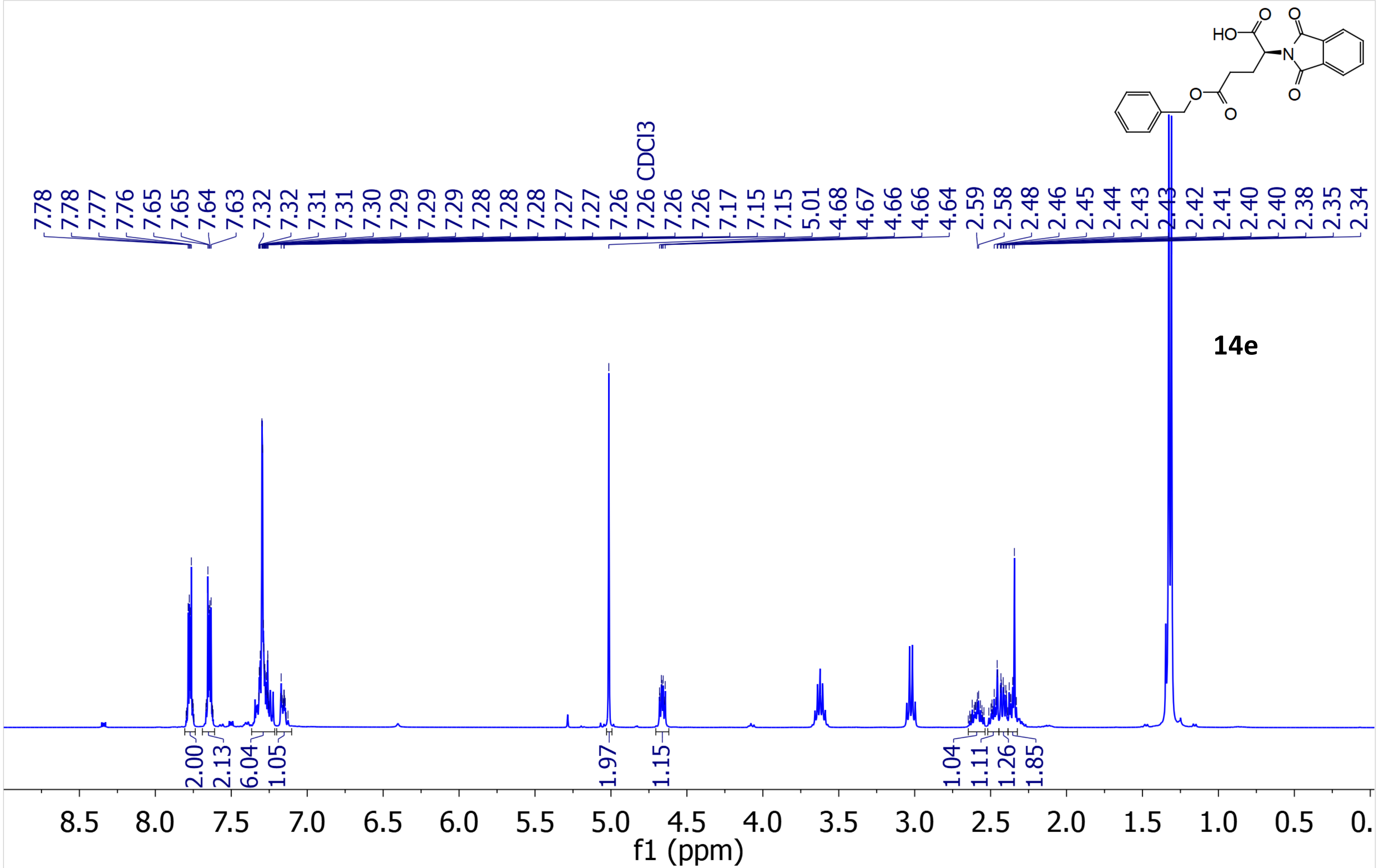

^{1}H NMR (400 MHz, Chloroform-*d*) δ 7.82 – 7.72 (m, 2H), 7.69 – 7.61 (m, 2H), 7.29 (dq, *J* = 10.4, 3.9, 3.5 Hz, 4H), 7.16 (dt, *J* = 8.0, 2.2 Hz, 1H), 5.01 (s, 2H), 4.69 – 4.63 (m, 1H), 2.65 – 2.54 (m, 1H), 2.52 – 2.45 (m, 1H), 2.45 – 2.33 (m, 2H).

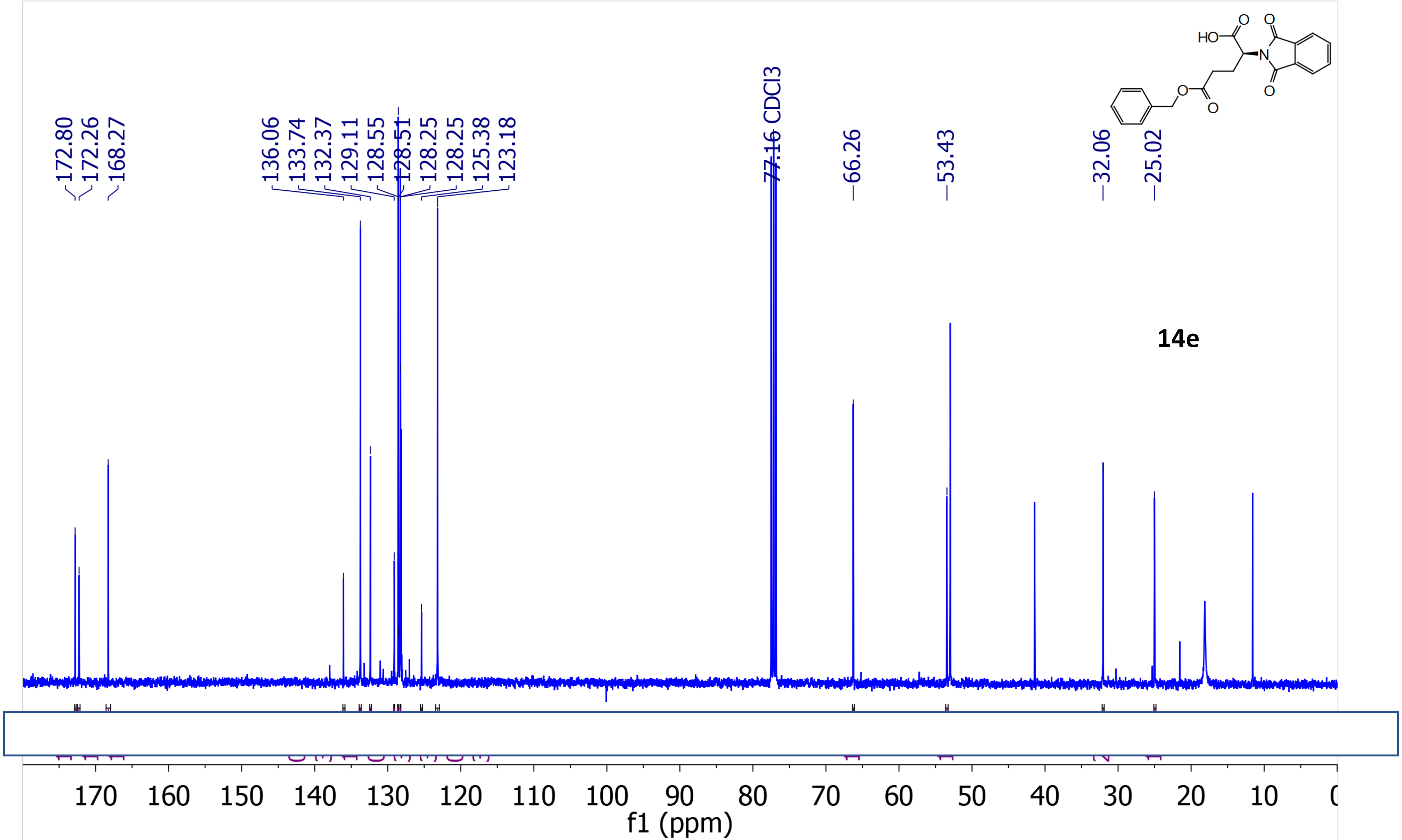

^{13}C NMR (101 MHz, $CDCl_3$) δ 172.80, 172.26, 168.27, 136.06, 133.74, 132.37, 129.11, 128.55, 128.51, 128.25, 128.25, 125.38, 123.18, 77.16, 66.26, 53.43, 32.06, 25.02.

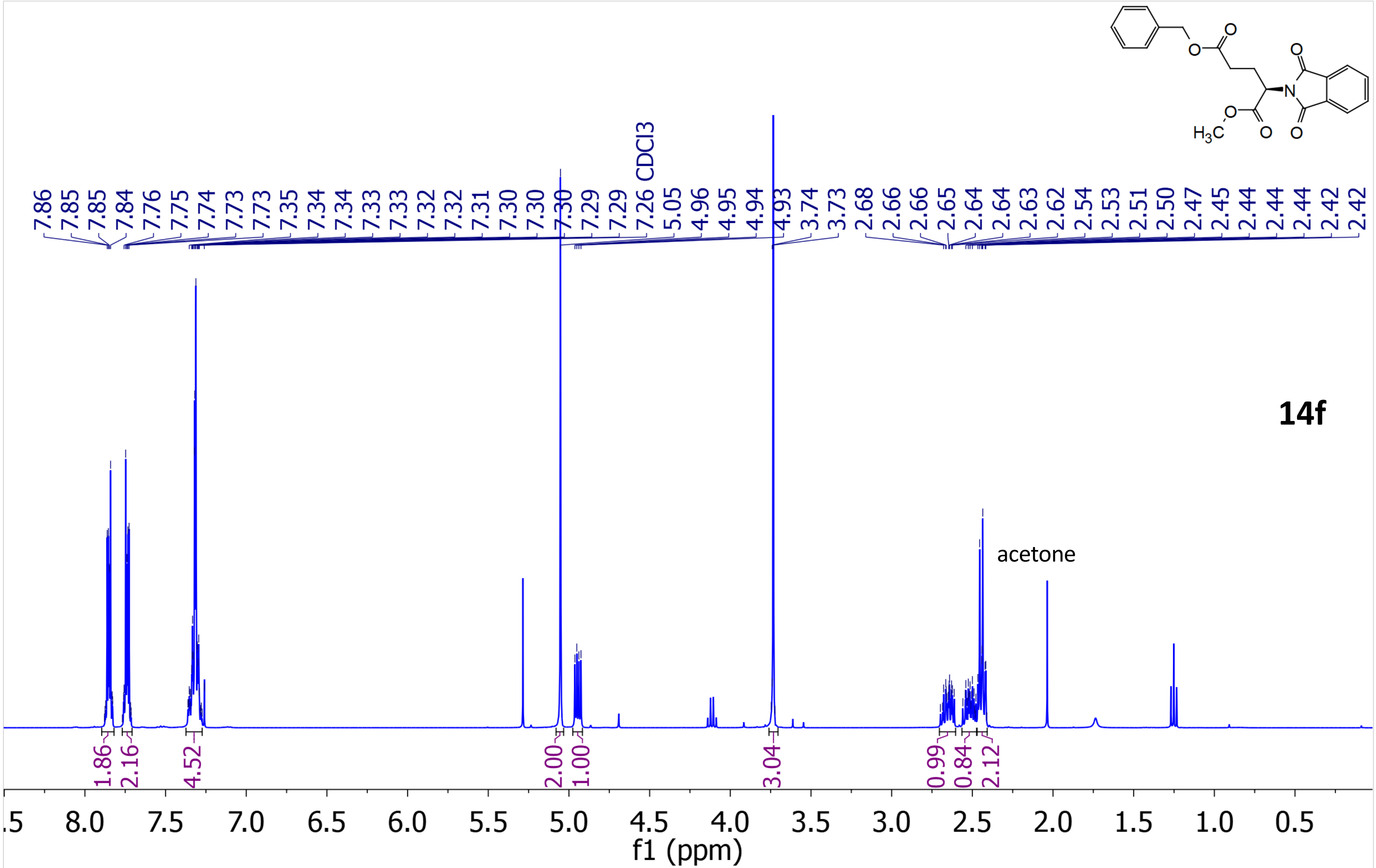

^{1}H NMR (400 MHz, Chloroform-*d*) δ 7.90 – 7.82 (m, 2H), 7.77 – 7.71 (m, 2H), 7.37 – 7.27 (m, 5H), 5.05 (s, 2H), 4.94 (dd, J = 10.0, 5.0 Hz, 1H), 3.73 (s, 3H), 2.70 – 2.60 (m, 1H), 2.56 – 2.47 (m, 1H), 2.47 – 2.41 (m, 2H).

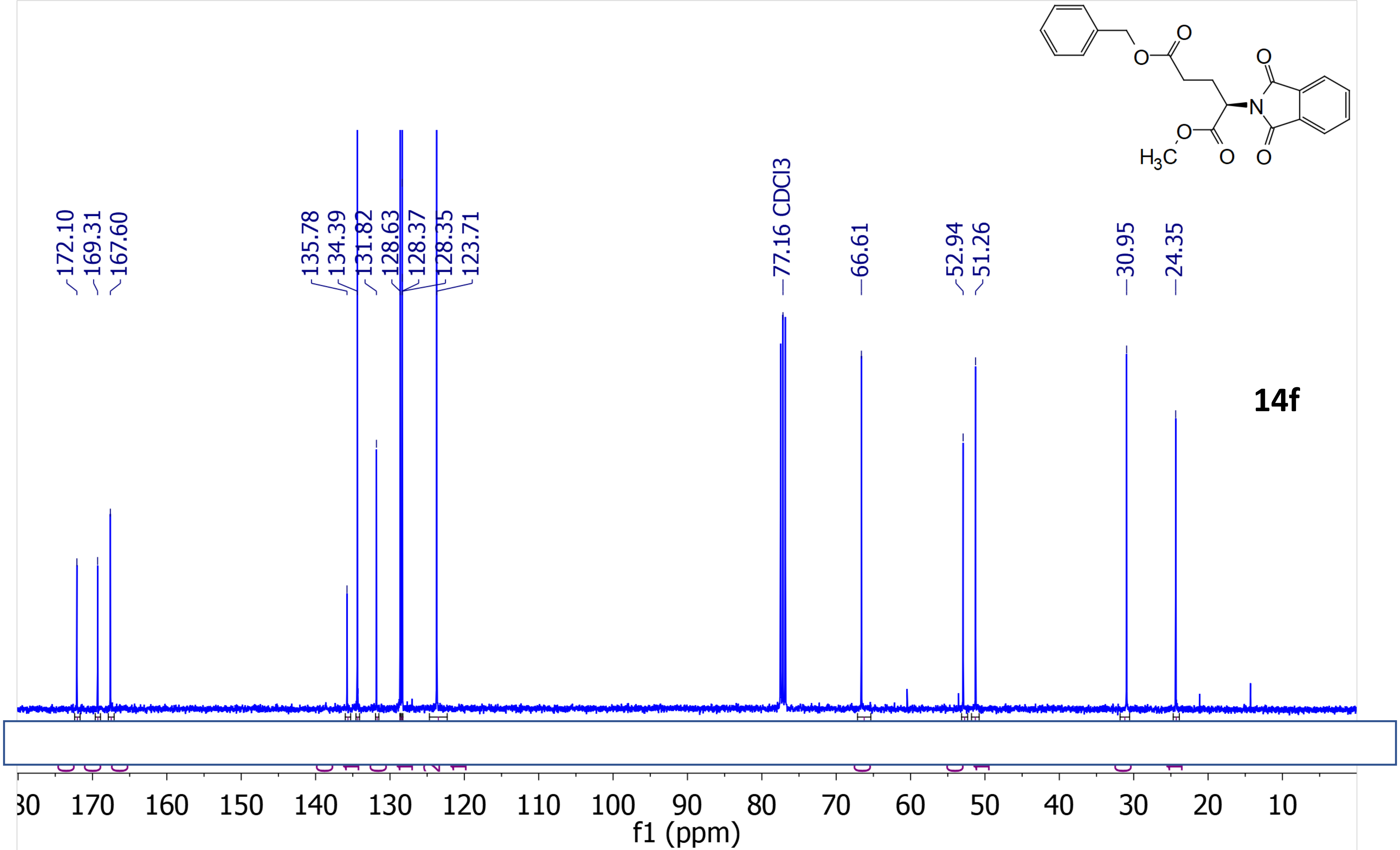

^{13}C NMR (101 MHz, $CDCl_3$) δ 172.10, 169.31, 167.60, 135.78, 134.39, 131.82, 128.63, 128.37, 128.35, 123.71, 66.61, 52.94, 51.26, 30.95, 24.35.

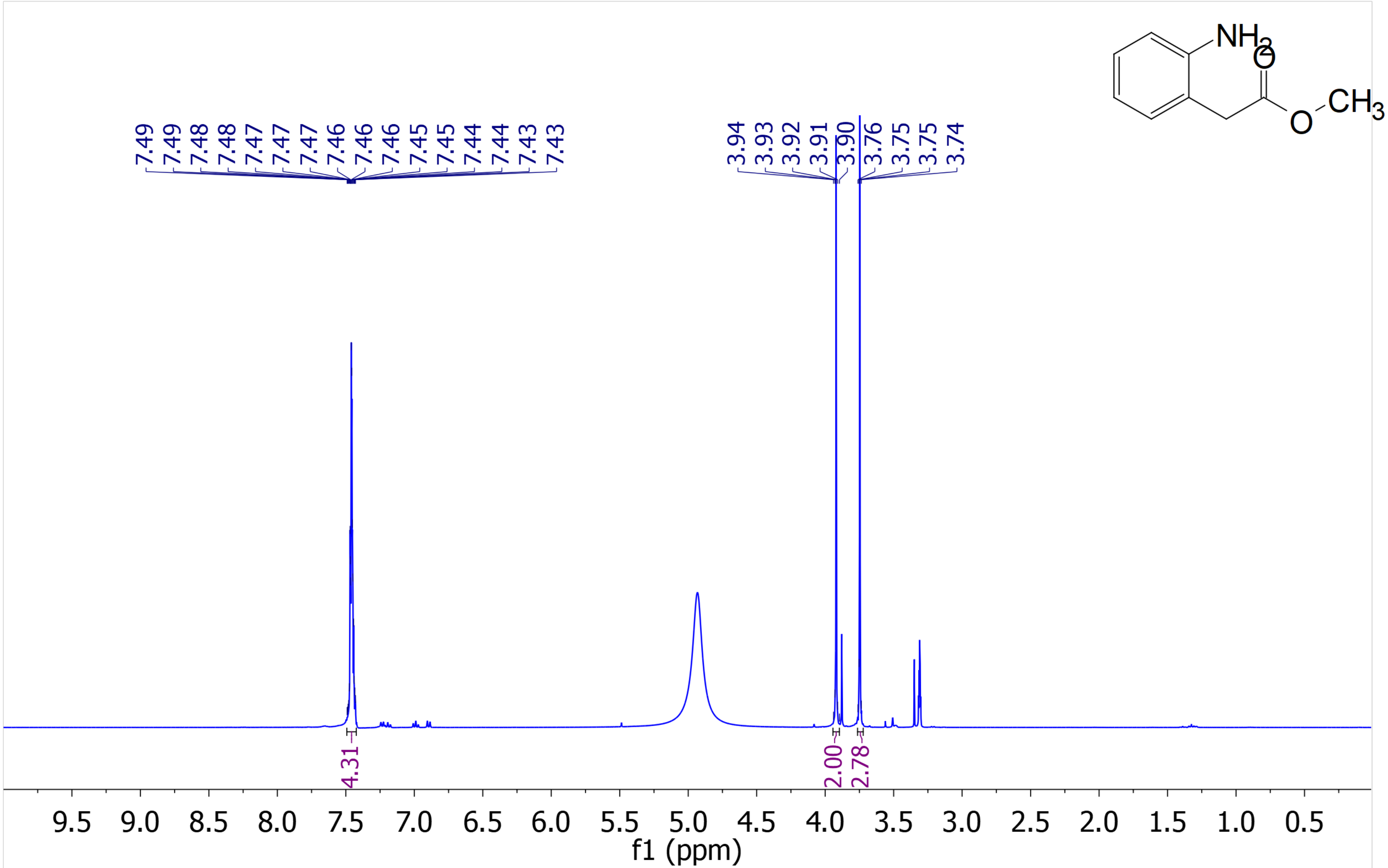

^{1}H NMR (400 MHz, Methanol-d_4) δ 7.46 (tt, J = 3.8, 1.7 Hz, 4H), 3.92 (s, 2H), 3.75 (s, 3H).

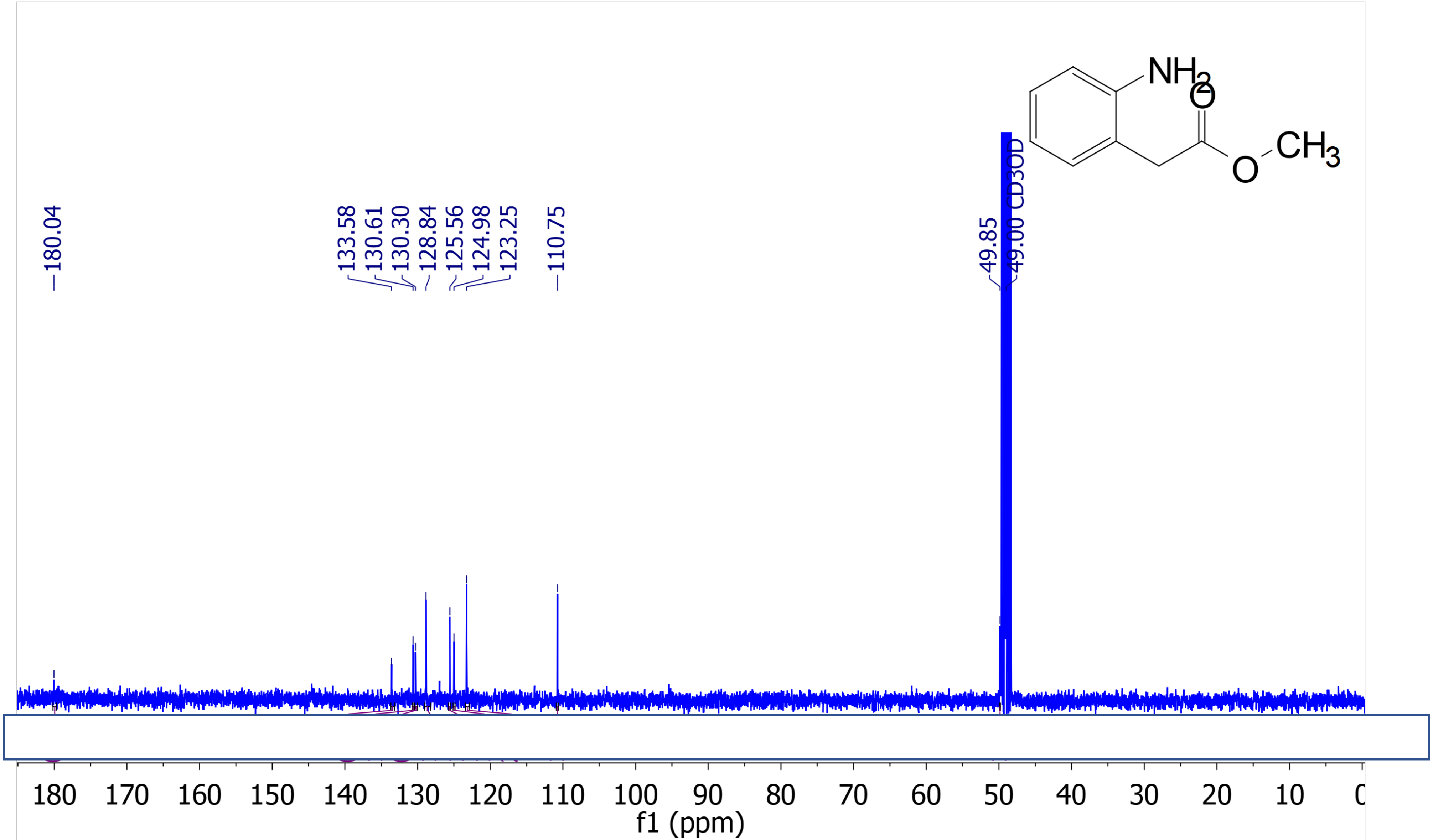

^{13}C NMR (101 MHz, MeOD) δ 180.04, 133.58, 130.61, 130.30, 128.84, 125.56, 124.98, 123.25, 110.75, 49.85, 49.00.

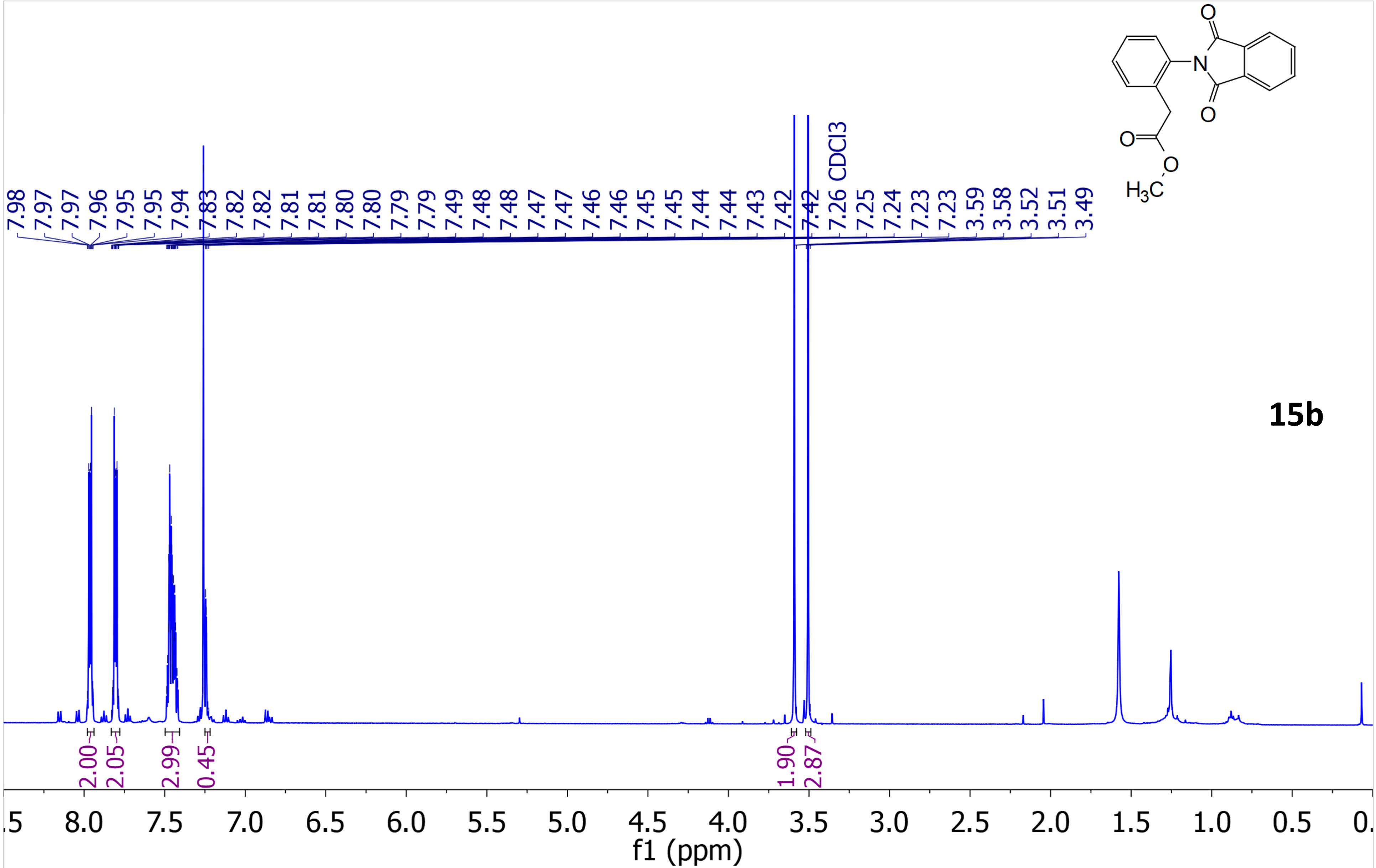

^{1}H NMR (500 MHz, Chloroform-*d*) δ 7.99 (dd, *J* = 5.4, 3.1 Hz, 2H), 7.83 (dd, *J* = 5.4, 3.1 Hz, 2H), 7.52 – 7.43 (m, 3H), 7.28 – 7.24 (m, 1H), 3.62 (s, 2H), 3.53 (s, 3H).

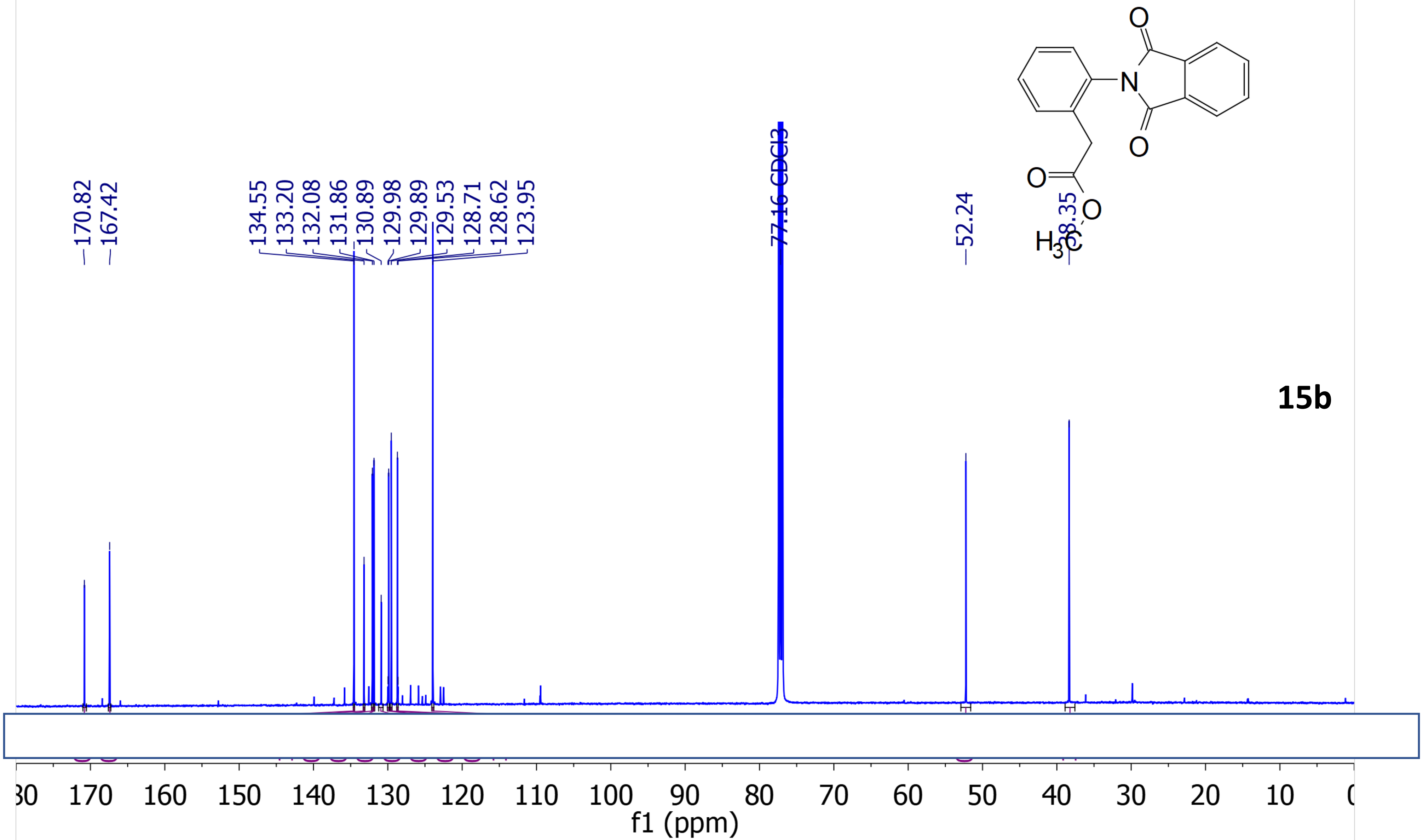

15b

^{13}C NMR (126 MHz, $CDCl_3$) δ 134.42, 133.07, 131.95, 131.73, 129.85, 129.76, 129.40, 128.58, 128.49, 123.82, 52.11, 38.21.

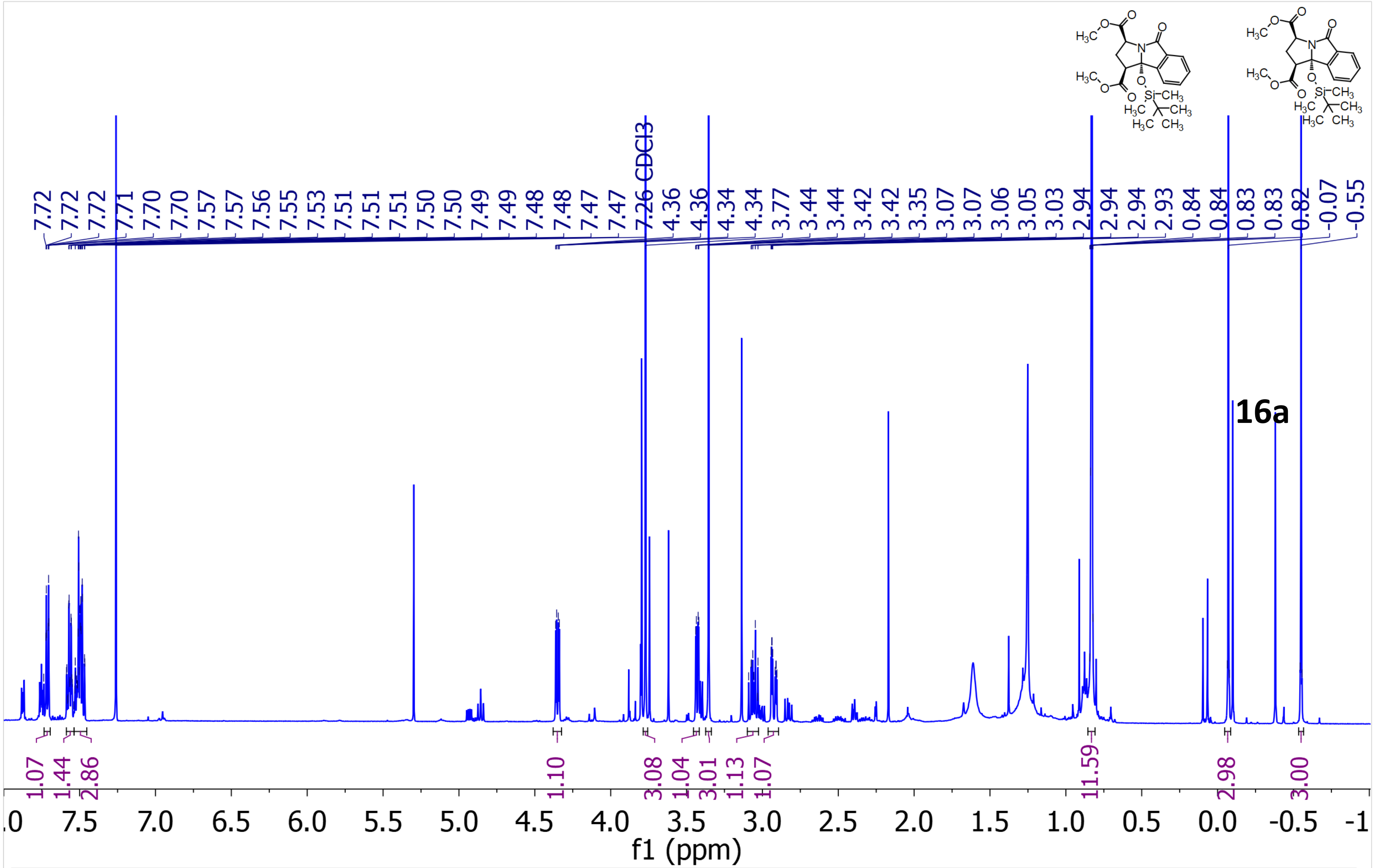

^{1}H NMR (500 MHz, Chloroform-*d*) δ 7.71 (dt, *J* = 7.5, 1.0 Hz, 1H), 7.59 – 7.54 (m, 1H), 7.54 – 7.45 (m, 3H), 4.35 (dd, *J* = 8.9, 3.4 Hz, 1H), 3.77 (s, 3H), 3.43 (dd, *J* = 8.1, 2.2 Hz, 1H), 3.35 (s, 3H), 3.10 – 3.03 (m, 1H), 2.92 (ddd, *J* = 13.6, 3.4, 2.3 Hz, 1H), 0.83 (d, *J* = 1.9 Hz, 12H), -0.07 (s, 3H), -0.55 (s, 3H).

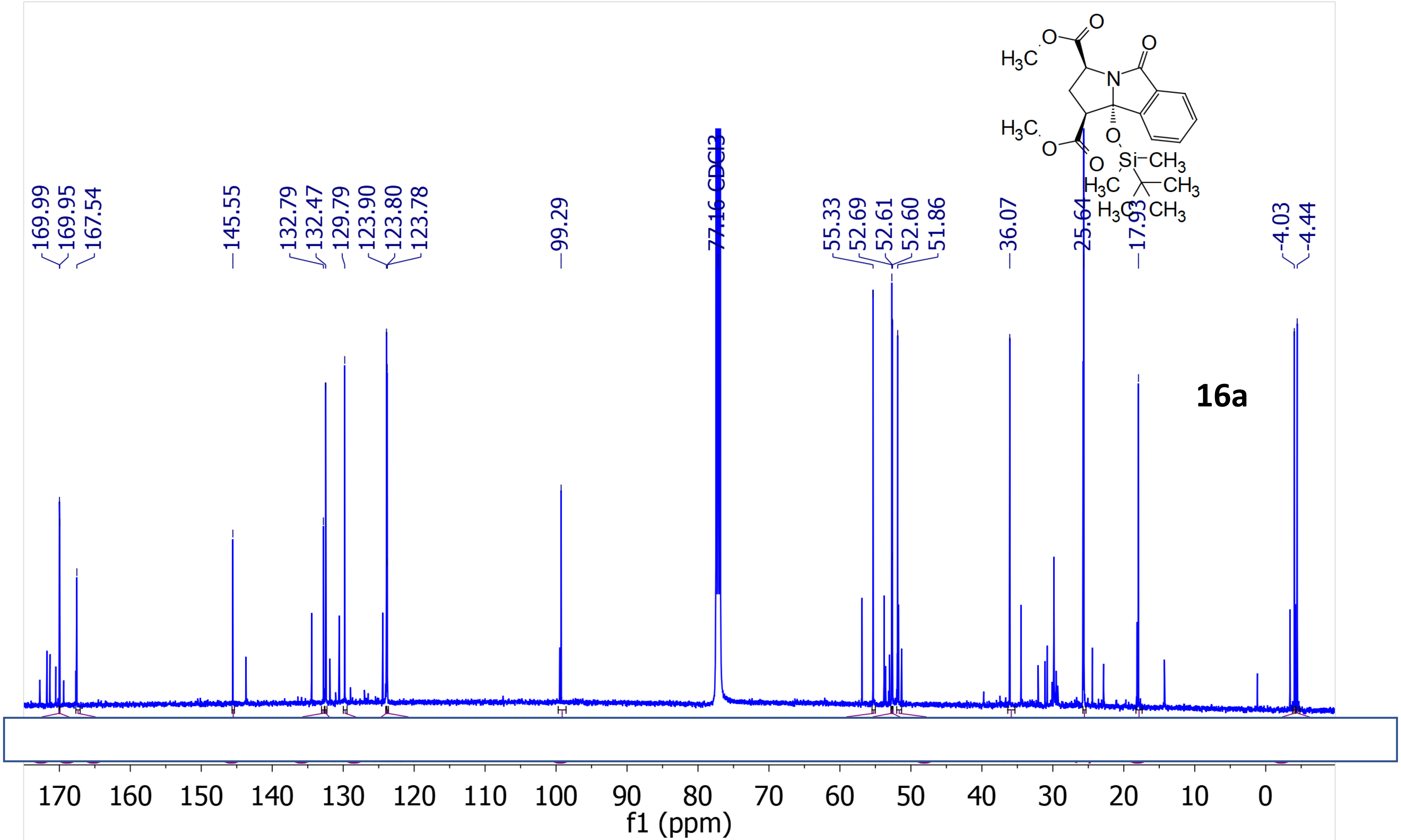

^{13}C NMR (126 MHz, $CDCl_3$) δ 169.99, 169.95, 167.54, 145.55, 132.79, 132.47, 129.79, 123.90, 123.80, 123.78, 99.29, 77.16, 55.33, 52.69, 52.61, 52.60, 51.86, 36.07, 25.64, 17.93, -4.03, -4.44.

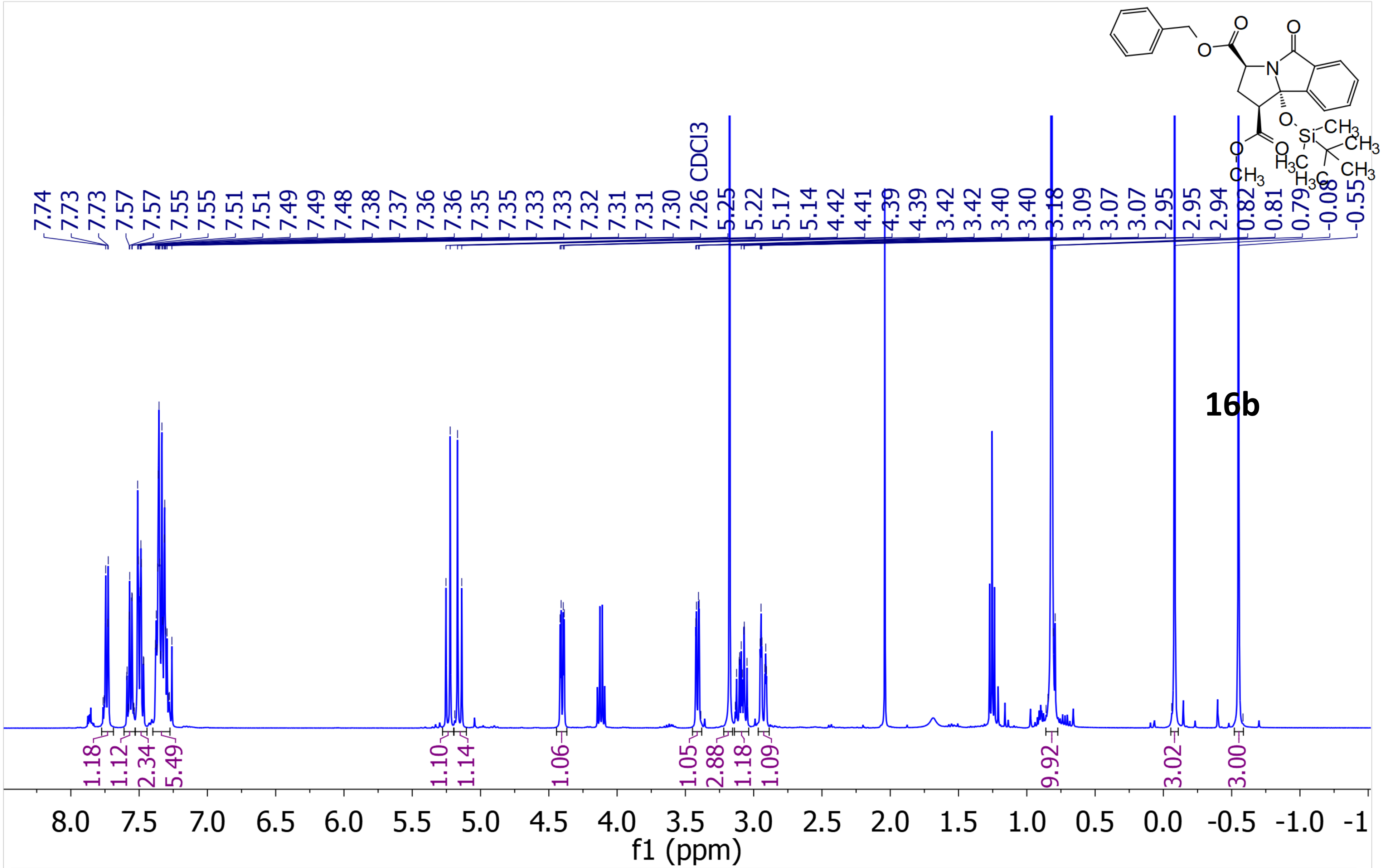

^{1}H NMR (400 MHz, Chloroform-*d*) δ 7.77 – 7.69 (m, 1H), 7.57 (td, *J* = 7.4, 1.1 Hz, 1H), 7.53 – 7.44 (m, 2H), 7.40 – 7.27 (m, 5H), 5.24 (d, *J* = 12.3 Hz, 1H), 5.15 (d, *J* = 12.3 Hz, 1H), 4.40 (dd, *J* = 9.0, 2.9 Hz, 1H), 3.41 (dd, *J* = 8.0, 1.9 Hz, 1H), 3.18 (s, 3H), 3.09 (ddd, *J* = 13.6, 9.0, 8.0 Hz, 1H), 2.93 (dt, *J* = 13.6, 2.4 Hz, 1H), 0.82 (s, 9H), -0.08 (s, 3H), -0.55 (s, 3H).

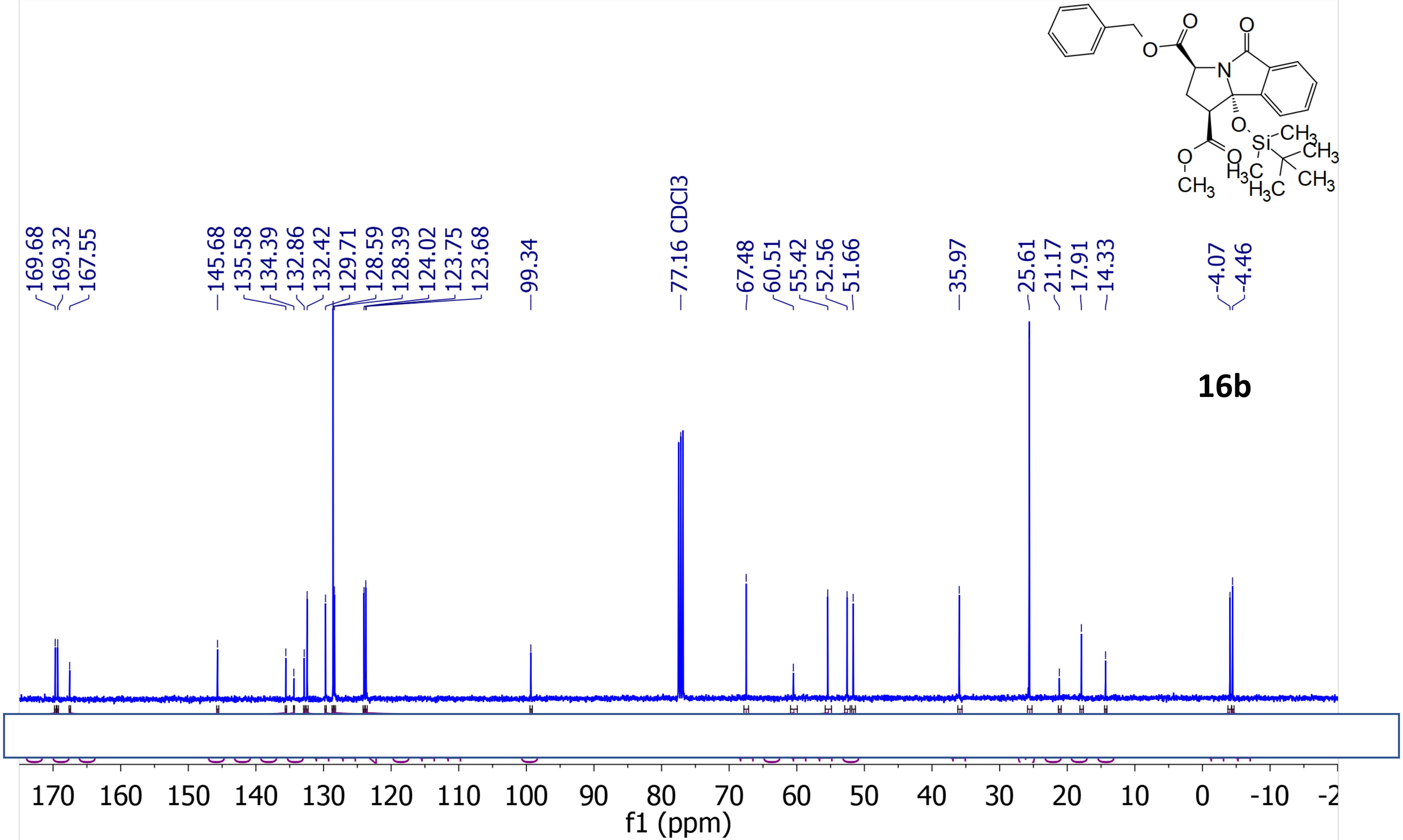

^{13}C NMR (101 MHz, Chloroform-*d*) δ 169.68, 169.32, 167.55, 145.68, 135.58, 134.39, 132.86, 132.42, 129.71, 128.59, 128.39, 124.02, 123.75, 123.68, 99.34, 67.48, 60.51, 55.42, 52.56, 51.66, 35.97, 25.61, 21.17, 17.91, 14.33, -4.07, -4.46.

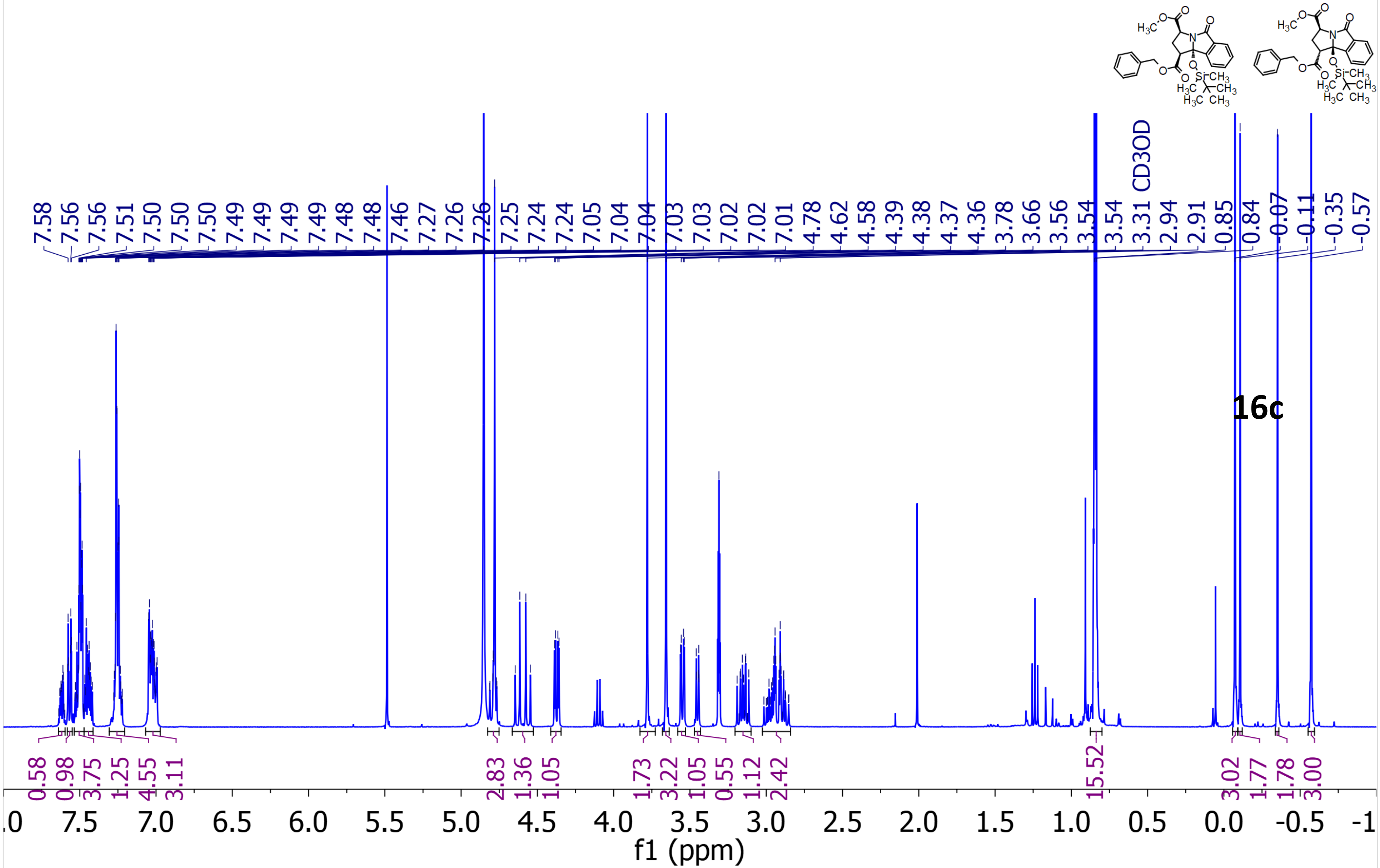

^{1}H NMR (400 MHz, Methanol-d_4) δ 7.64 – 7.60 (m, 1H), 7.57 (dt, J = 7.2, 1.2 Hz, 1H), 7.54 – 7.47 (m, 4H), 7.47 – 7.41 (m, 1H), 7.31 – 7.21 (m, 5H), 7.07 – 6.97 (m, 3H), 4.83 – 4.75 (m, 3H), 4.66 – 4.53 (m, 2H), 4.37 (dd, J = 9.0, 3.1 Hz, 1H), 3.78 (s, 3H), 3.66 (s, 3H), 3.55 (dd, J = 8.0, 2.0 Hz, 1H), 3.47 – 3.43 (m, 1H), 3.15 (ddd, J = 13.7, 9.0, 7.9 Hz, 1H), 3.03 – 2.84 (m, 3H), 0.84 (d, J = 4.3 Hz, 18H), -0.07 (s, 3H), -0.11 (s, 3H), -0.35 (s, 3H), -0.57 (s, 3H).

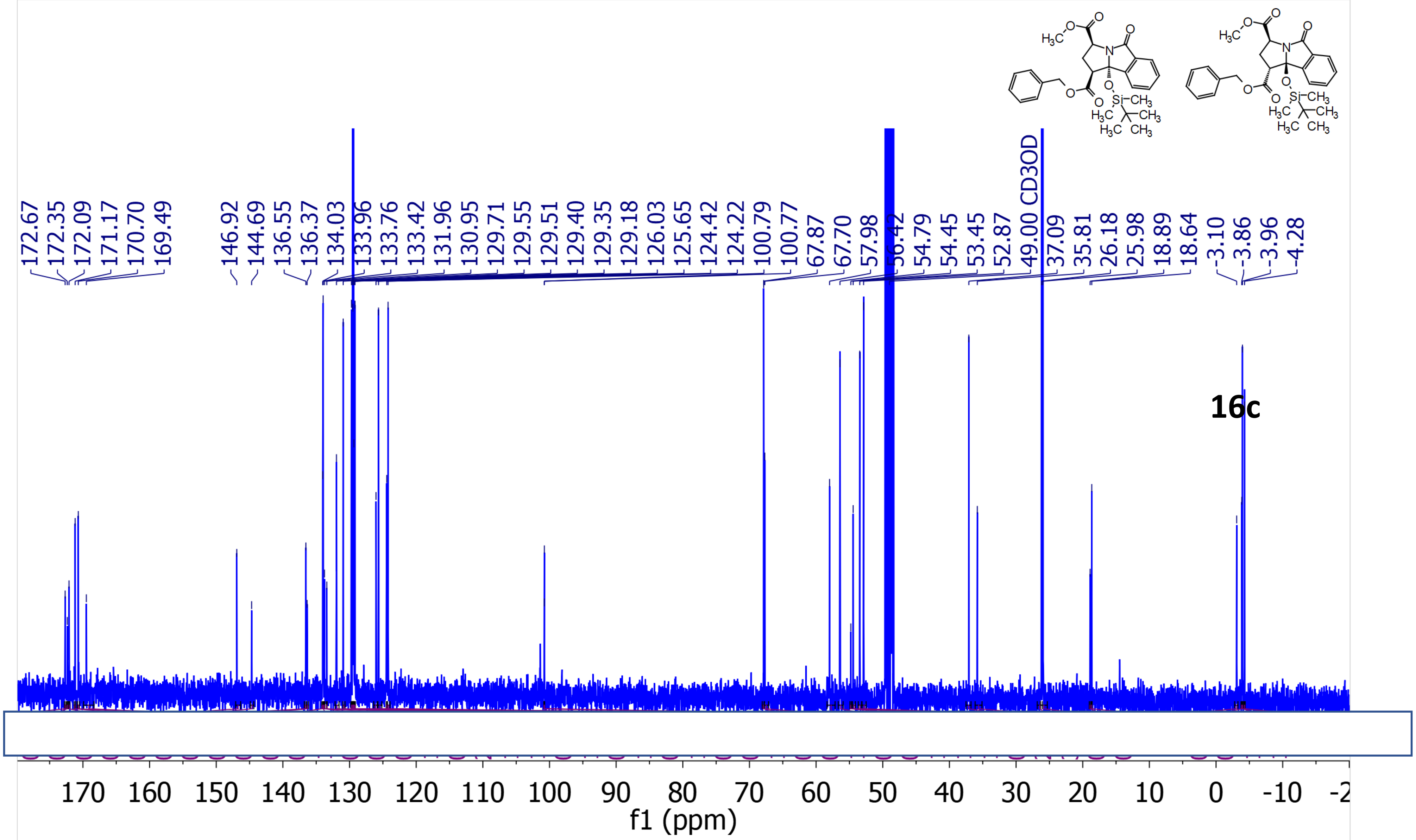

^{13}C NMR (101 MHz, MeOD) δ 172.67, 172.35, 172.09, 171.17, 170.70, 169.49, 146.92, 144.69, 136.55, 136.37, 134.03, 133.96, 133.76, 133.42, 131.96, 130.95, 129.71, 129.55, 129.51, 129.40, 129.35, 129.18, 126.03, 125.65, 124.42, 124.22, 100.79, 100.77, 67.87, 67.70, 57.98, 56.42, 54.79, 54.45, 53.45, 52.87, 49.00, 37.09, 35.81, 26.18, 25.98, 18.89, 18.64, -3.10, -3.86, -3.96, -4.28.

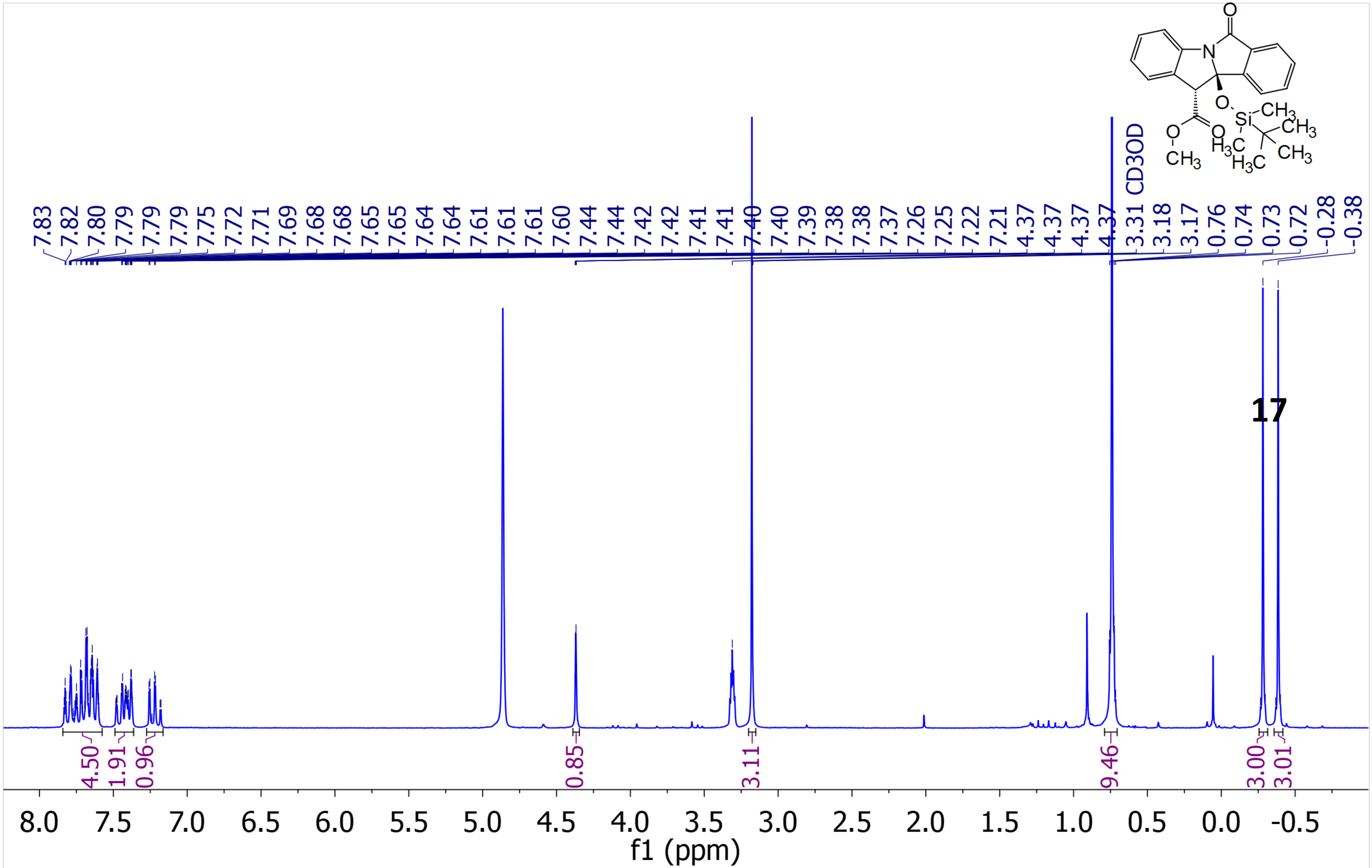

^{1}H NMR (200 MHz, Methanol-d_4) δ 7.84 – 7.58 (m, 5H), 7.49 – 7.36 (m, 2H), 7.22 (td, J = 7.5, 1.2 Hz, 1H), 4.37 (t, J = 0.6 Hz, 1H), 3.18 (s, 3H), 0.74 (s, 9H), -0.28 (s, 3H), -0.38 (s, 3H).

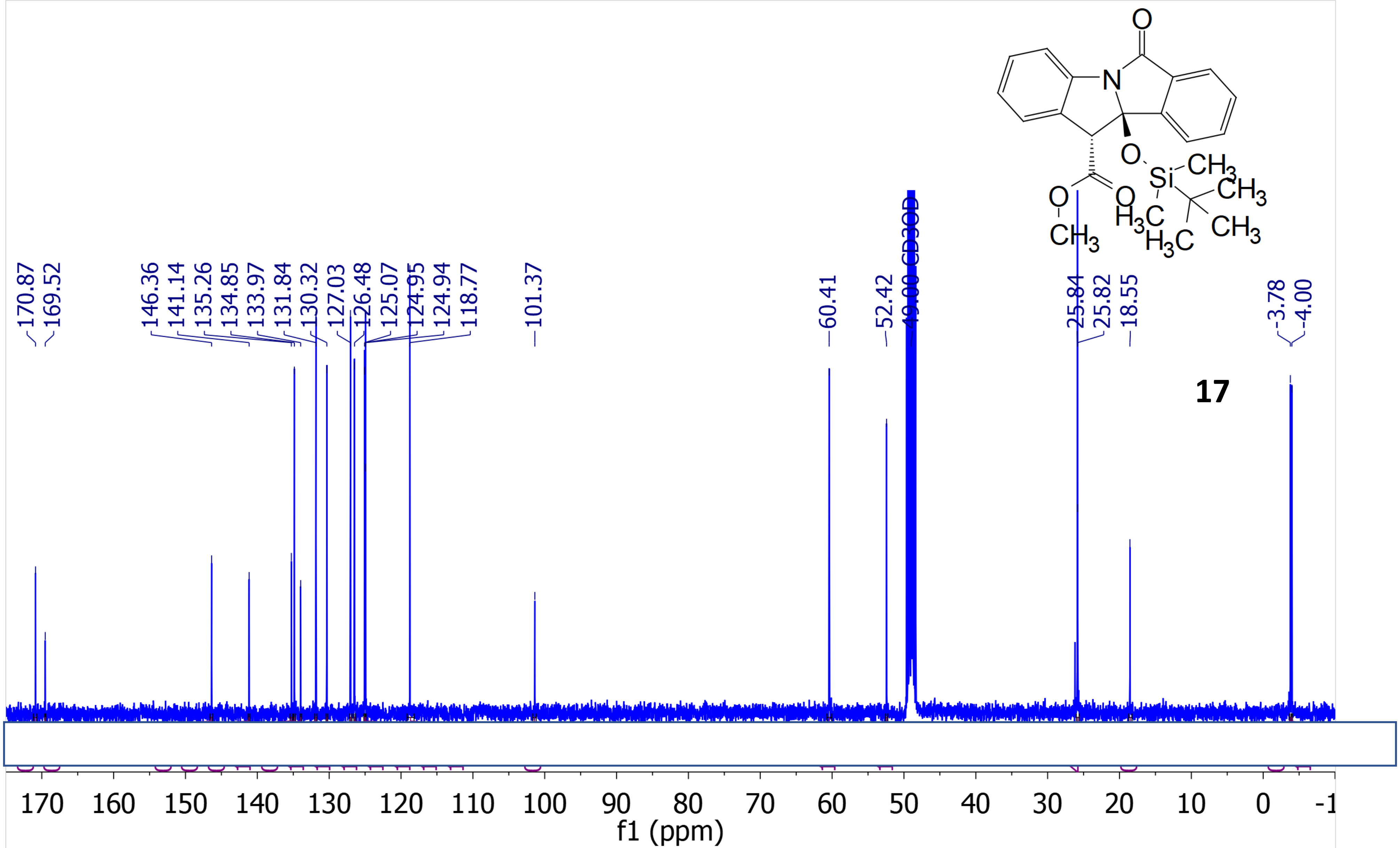

^{13}C NMR (101 MHz, MeOD) δ 170.87, 169.52, 146.36, 141.14, 135.26, 134.85, 133.97, 131.84, 130.32, 127.03, 126.48, 125.07, 124.95, 124.94, 118.77, 101.37, 60.41, 52.42, 25.84, 25.82, 18.55, -3.78, -4.00.

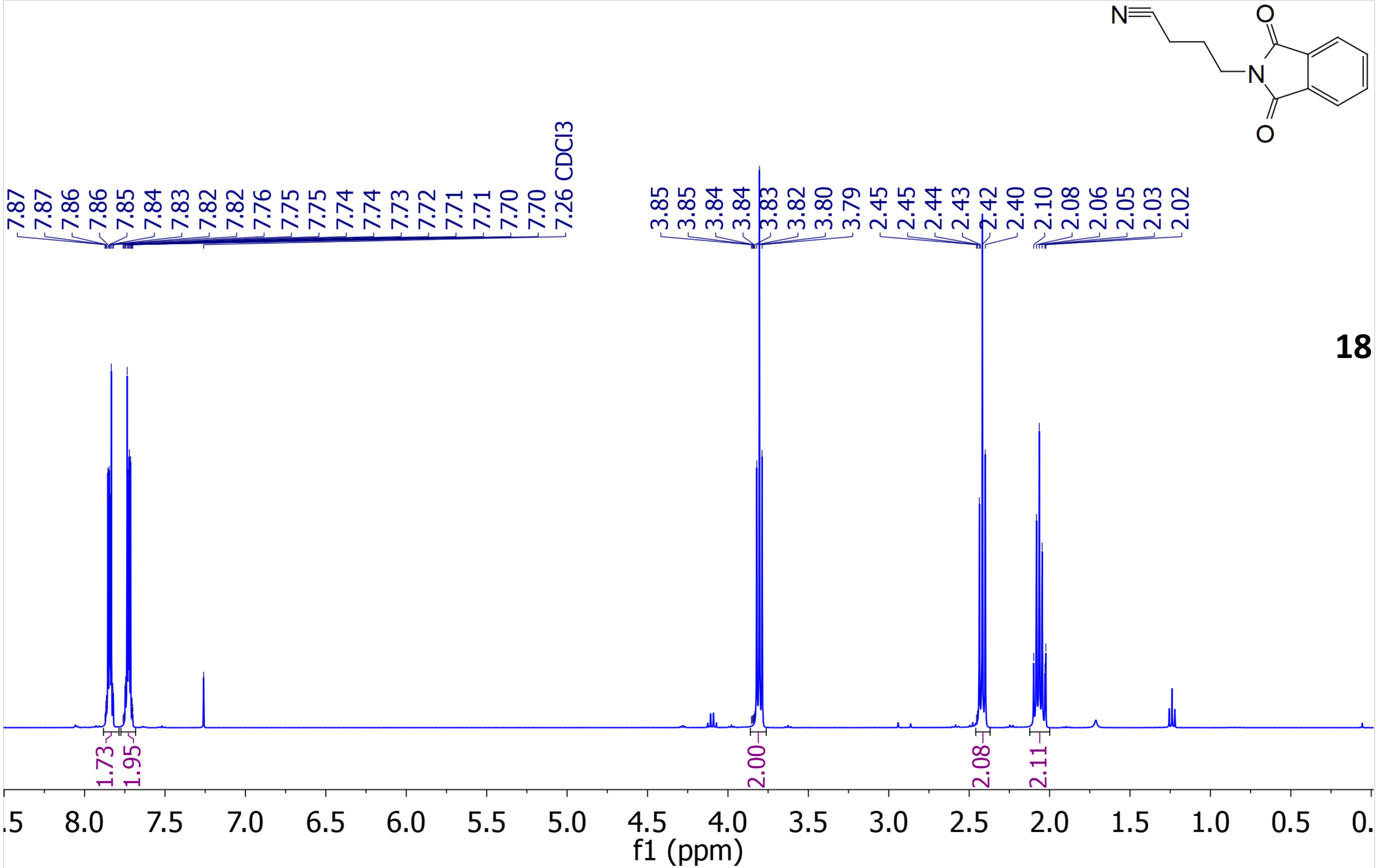

18

^{1}H NMR (400 MHz, Chloroform-*d*) δ 7.84 (dd, *J* = 5.5, 3.1 Hz, 2H), 7.73 (dd, *J* = 5.5, 3.1 Hz, 2H), 3.80 (t, *J* = 6.7 Hz, 2H), 2.42 (t, *J* = 7.3 Hz, 2H), 2.06 (dt, *J* = 14.0, 7.2, 6.9 Hz, 2H).

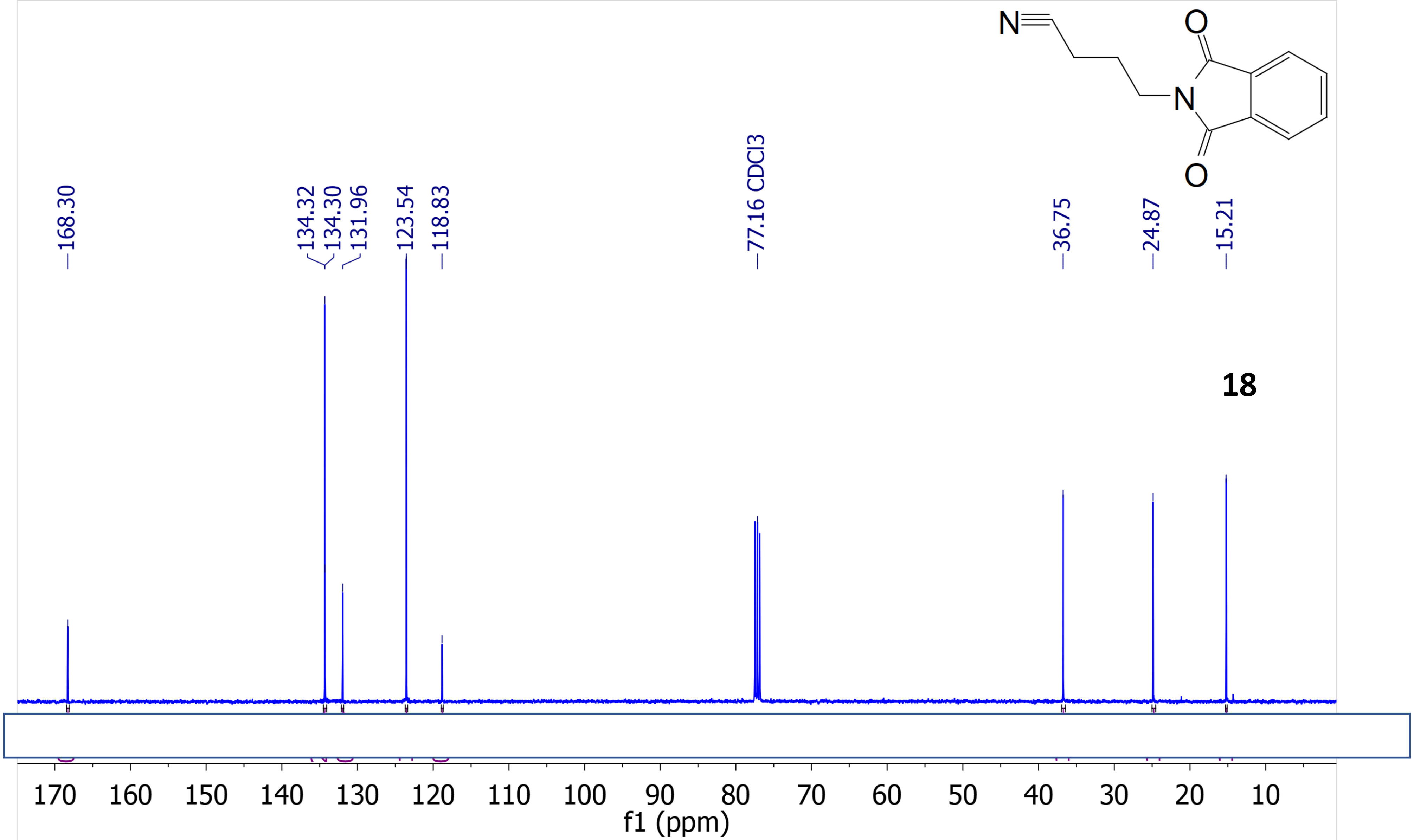

18

^{13}C NMR (101 MHz, $CDCl_3$) δ 168.30, 134.32, 134.30, 131.96, 123.54, 118.83, 36.75, 24.87, 15.21.

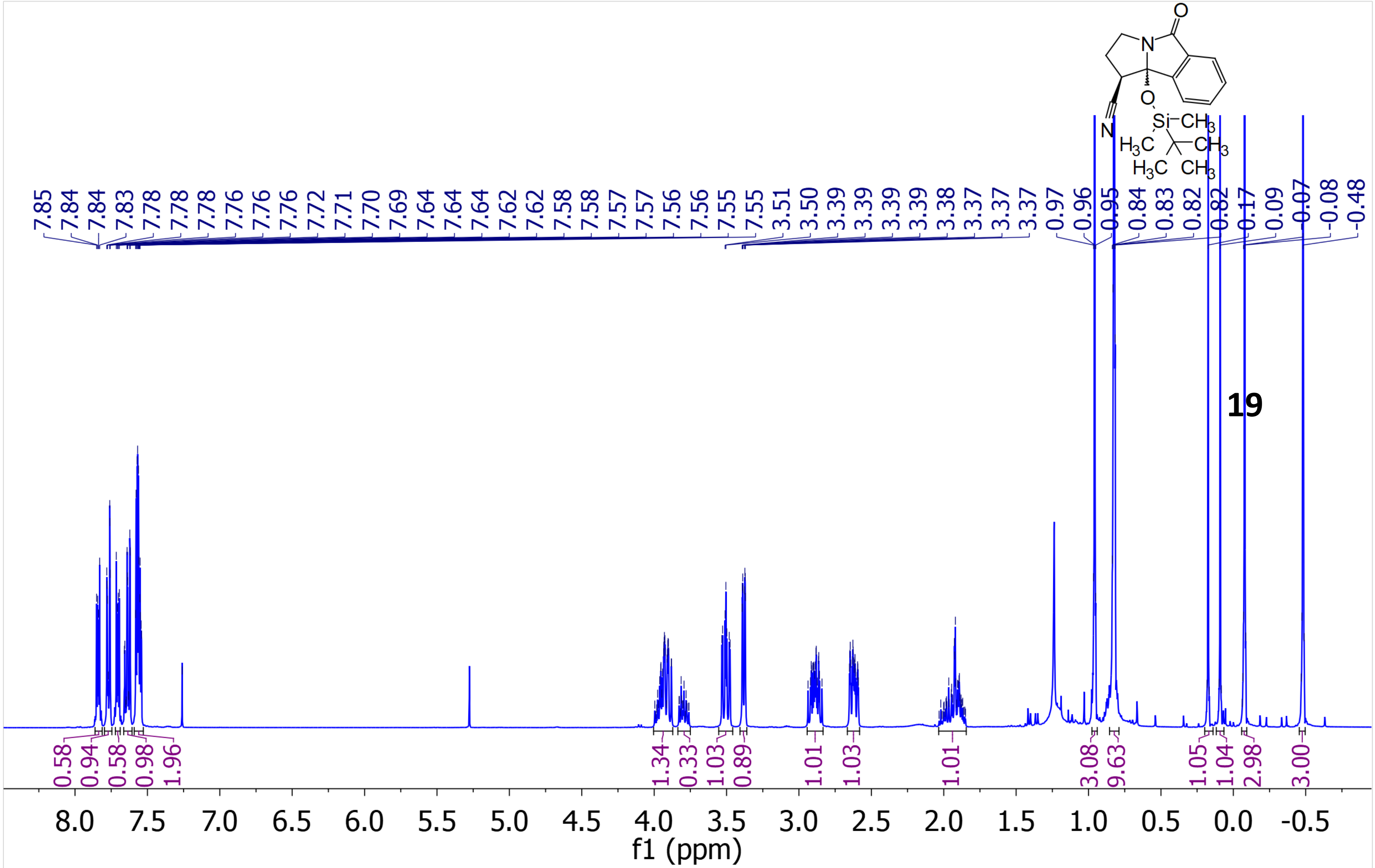

^{1}H NMR (400 MHz, Chloroform-*d*) δ 7.84 (dd, *J* = 5.5, 3.0 Hz, 1H), 7.80 – 7.75 (m, 1H), 7.70 (dd, *J* = 5.5, 3.0 Hz, 1H), 7.66 – 7.61 (m, 1H), 7.59 – 7.53 (m, 2H), 4.00 – 3.87 (m, 1H), 3.84 – 3.75 (m, 0H), 3.50 (ddd, *J* = 11.4, 8.9, 2.2 Hz, 1H), 3.38 (ddd, *J* = 6.8, 1.5, 0.6 Hz, 1H), 2.89 (dddd, *J* = 13.2, 9.7, 8.9, 6.9 Hz, 1H), 2.62 (dddd, *J* = 13.1, 7.9, 2.2, 1.5 Hz, 1H), 2.03 – 1.84 (m, 1H), 0.96 (s, 3H), 0.82 (s, 10H), 0.17 (s, 1H), 0.09 (s, 1H), -0.08 (s, 3H), -0.48 (s, 3H).

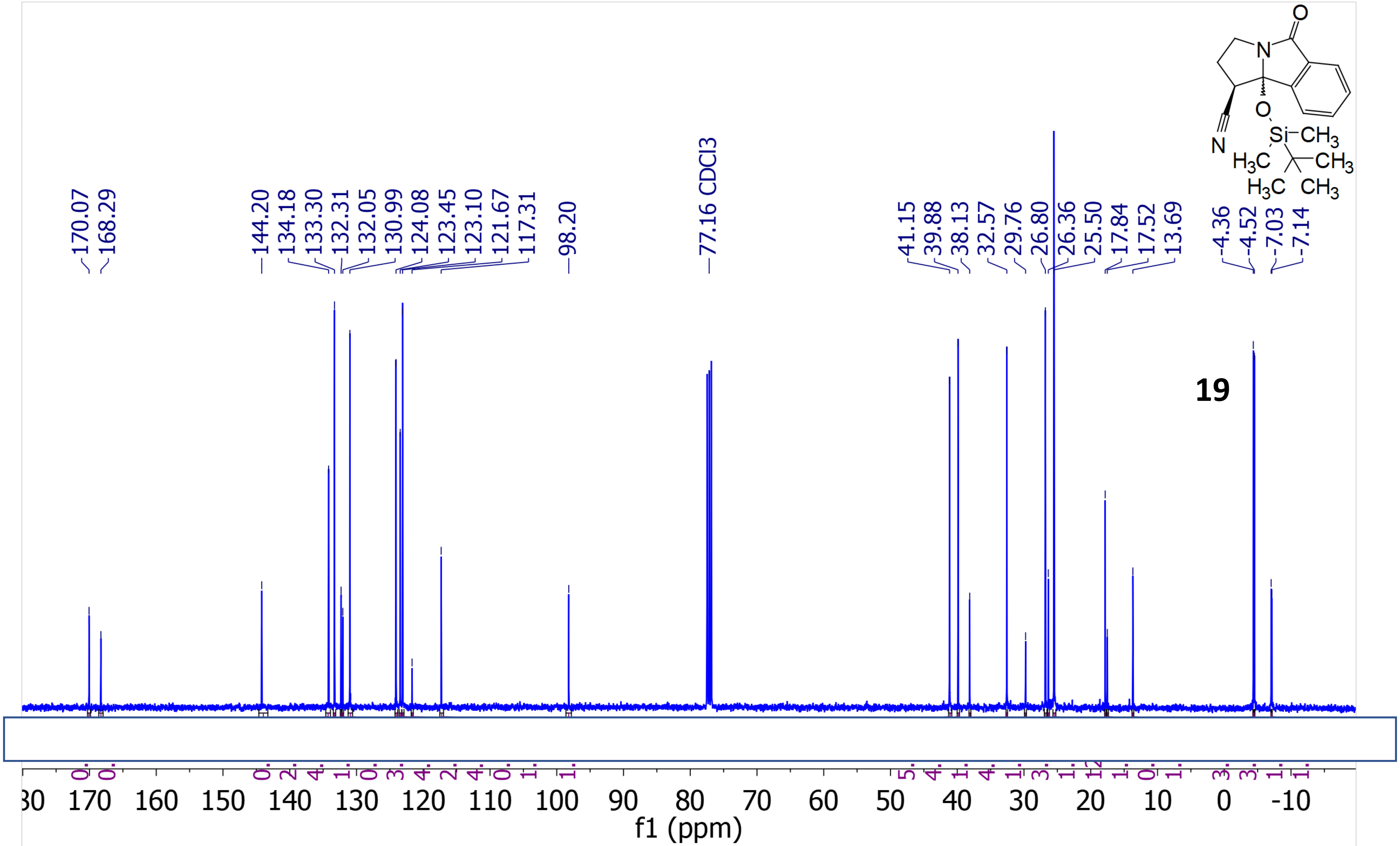

^{13}C NMR (101 MHz, $CDCl_3$) δ 170.07, 168.29, 144.20, 134.18, 133.30, 132.31, 132.05, 130.99, 124.08, 123.45, 123.10, 121.67, 117.31, 98.20, 41.15, 39.88, 38.13, 32.57, 29.76, 26.80, 26.36, 25.50, 17.84, 17.52, 13.69, -4.36, -4.52, -7.03, -7.14.

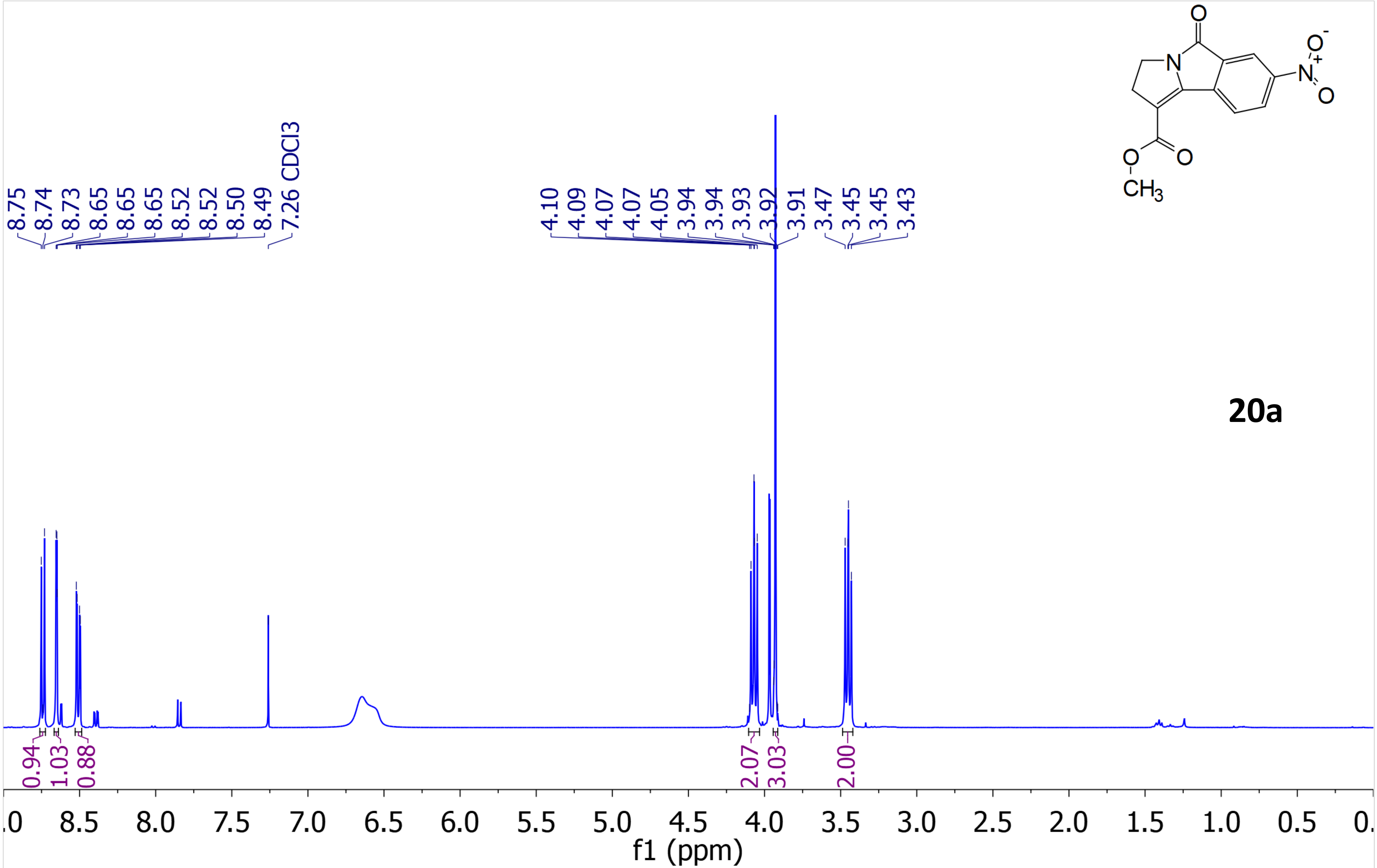

20a

^{1}H NMR (400 MHz, Chloroform-*d*) δ 8.74 (d, *J* = 8.4 Hz, 1H), 8.65 (d, *J* = 2.0 Hz, 1H), 8.51 (dd, *J* = 8.4, 2.1 Hz, 1H), 4.07 (t, *J* = 8.8, 7.6 Hz, 2H), 3.93 (s, 3H), 3.45 (t, *J* = 8.8, 7.5 Hz, 2H).

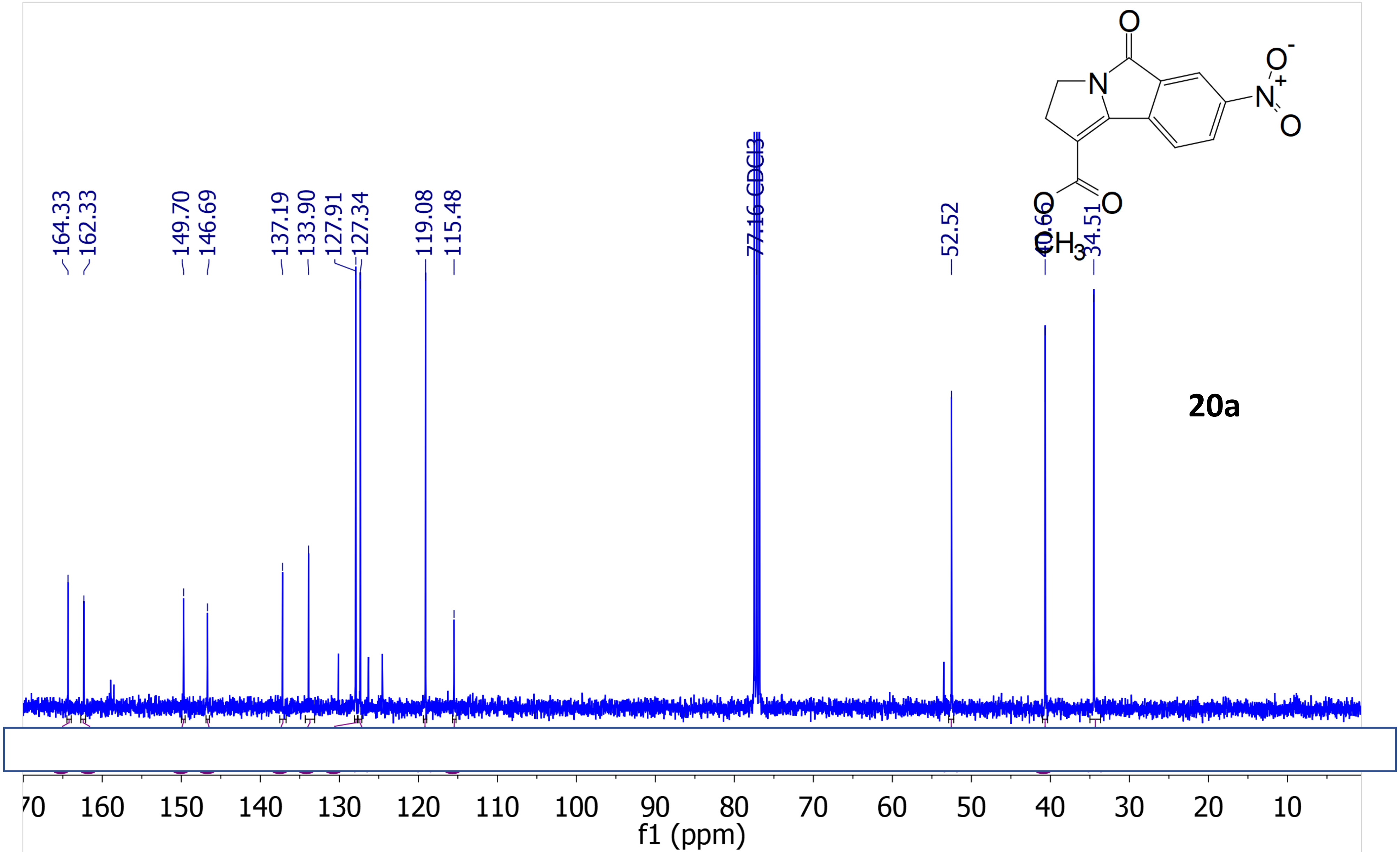

^{13}C NMR (101 MHz, $CDCl_3$) δ 164.33, 162.33, 149.70, 146.69, 137.19, 133.90, 127.91, 127.34, 119.08, 115.48, 52.52, 40.66, 34.51.

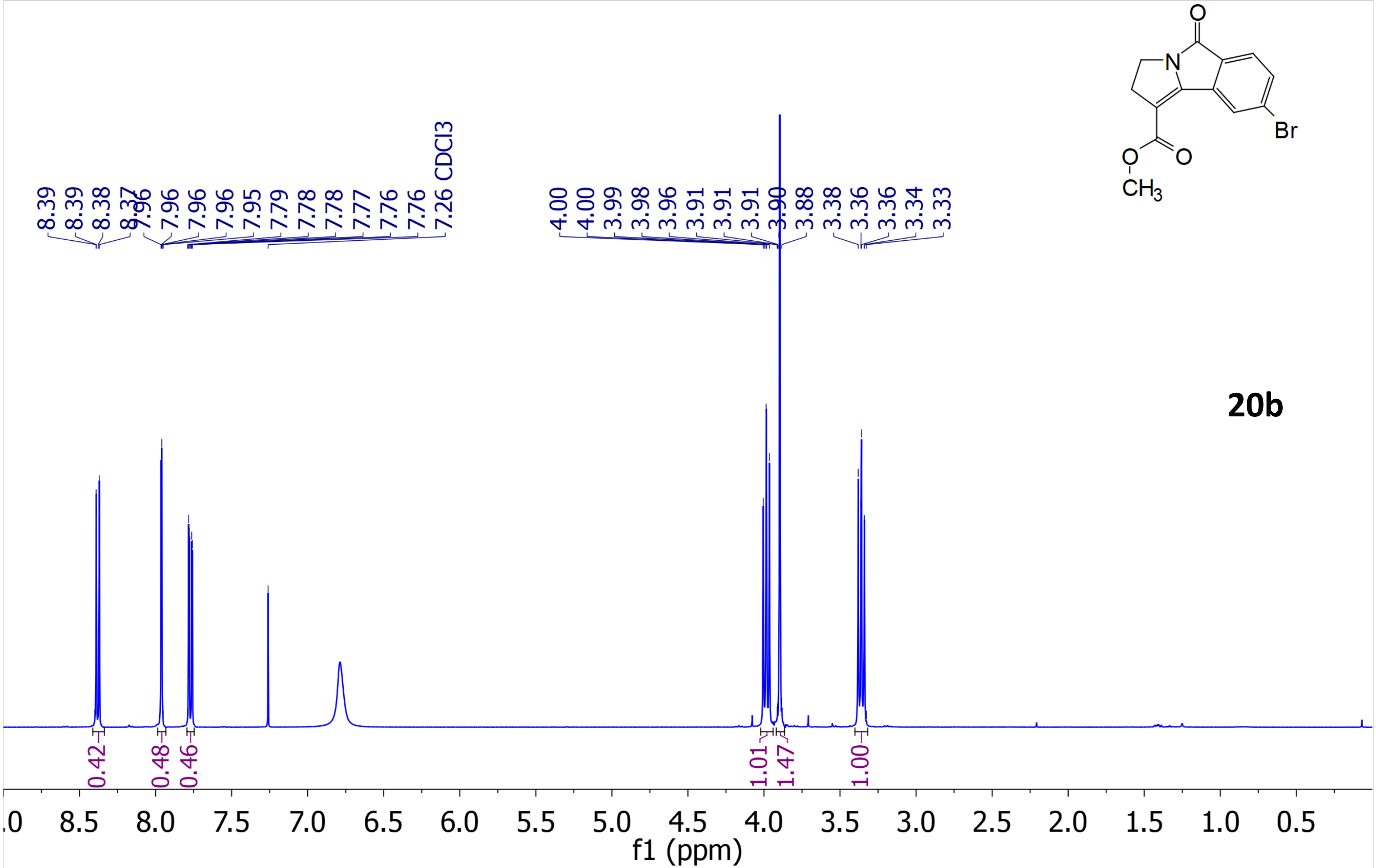

^{1}H NMR (400 MHz, Chloroform-*d*) δ 8.41 – 8.34 (m, 1H), 7.96 (dd, *J* = 1.9, 0.5 Hz, 1H), 7.77 (dd, *J* = 8.2, 1.8 Hz, 1H), 3.98 (dd, *J* = 8.7, 7.5 Hz, 2H), 3.90 (s, 3H), 3.36 (dd, *J* = 8.7, 7.4 Hz, 2H).

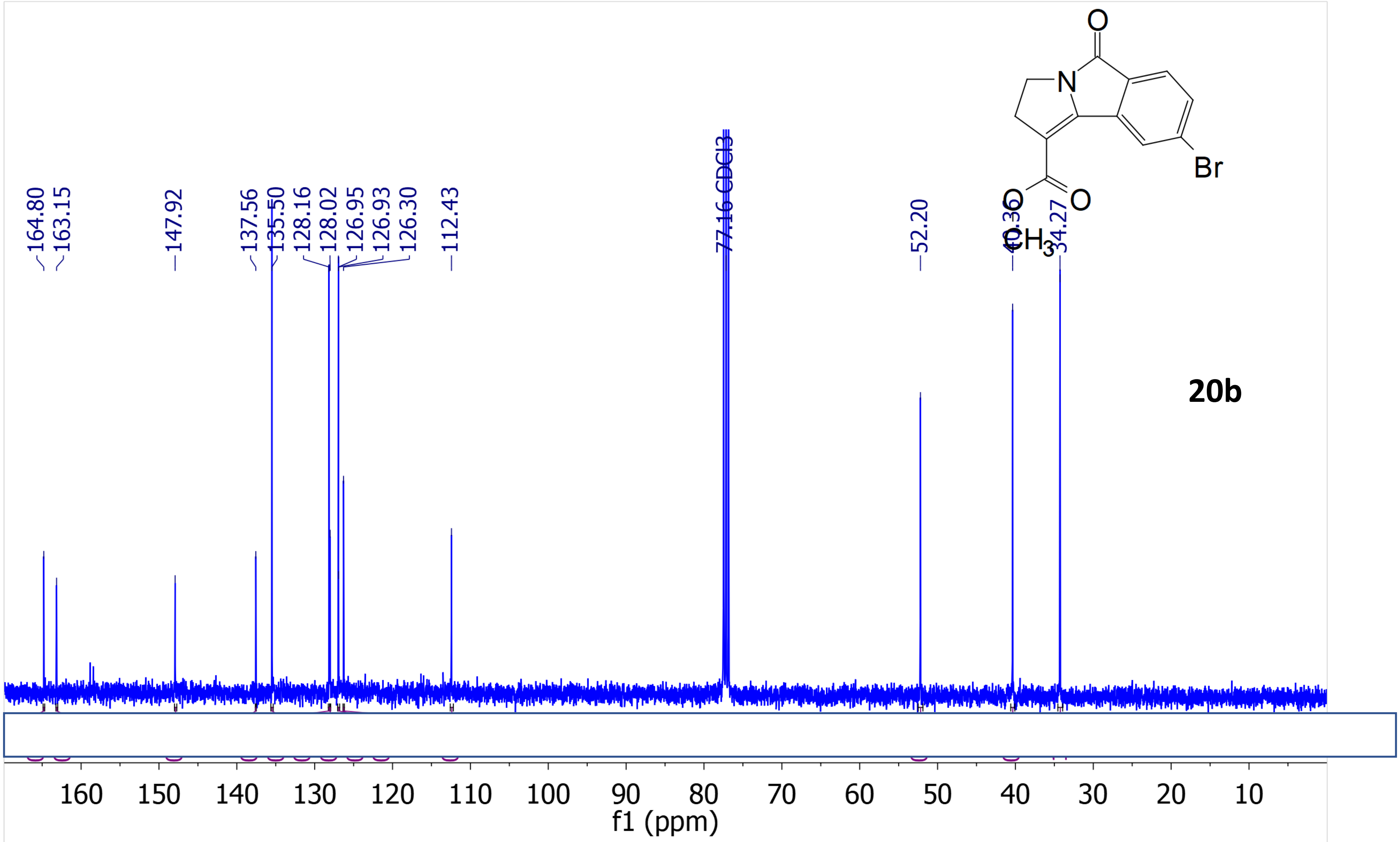

^{13}C NMR (101 MHz, $CDCl_3$) δ 164.80, 163.15, 147.92, 137.56, 135.50, 128.16, 128.02, 126.95, 126.93, 126.30, 112.43, 52.20, 40.36, 34.27.

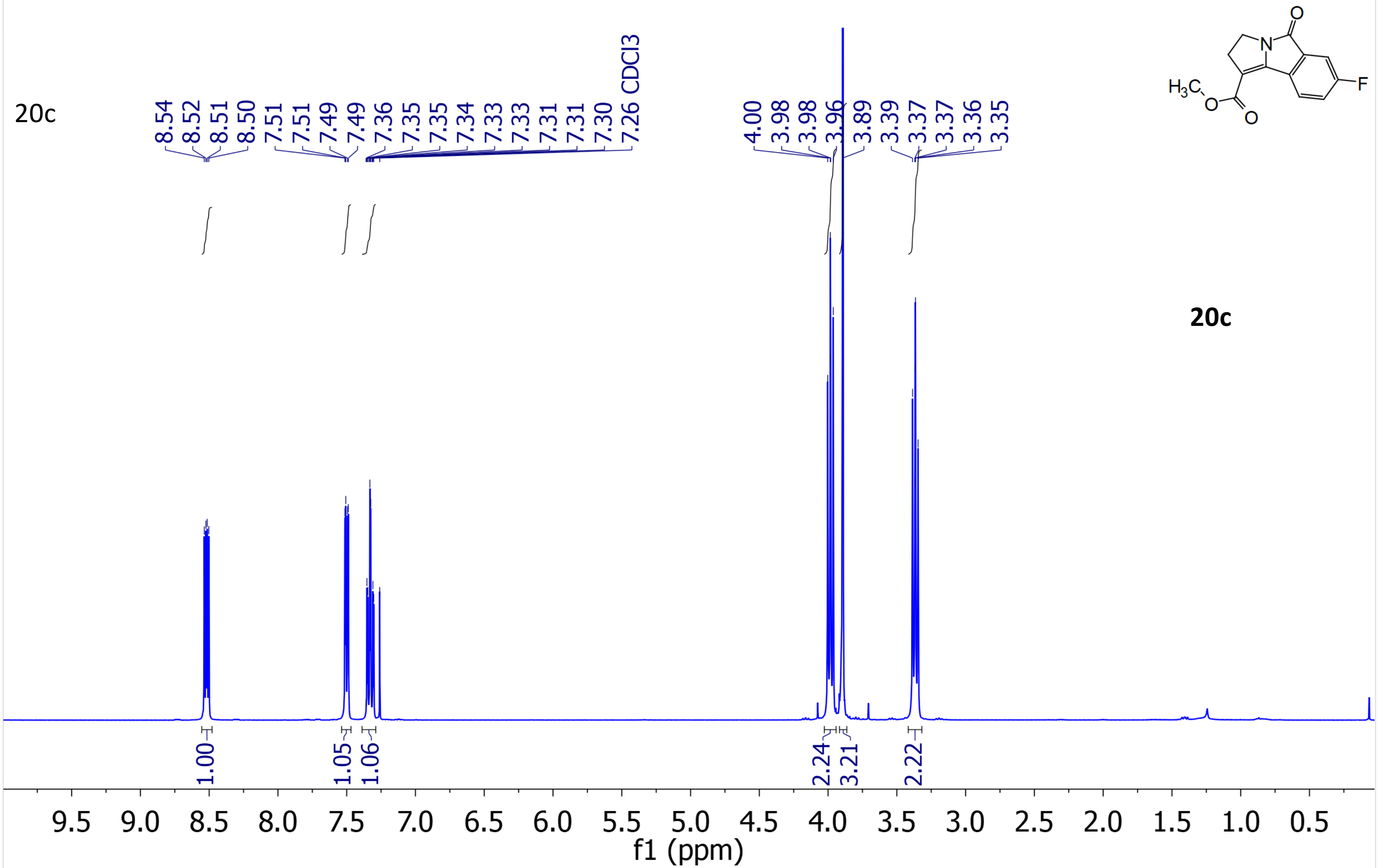

^{1}H NMR (400 MHz, Chloroform-*d*) δ 8.52 (dd, *J* = 8.5, 4.7 Hz, 1H), 7.50 (dd, *J* = 7.5, 2.4 Hz, 1H), 7.33 (td, *J* = 8.7, 2.5 Hz, 1H), 3.98 (dd, *J* = 8.7, 7.4 Hz, 2H), 3.89 (s, 3H), 3.37 (dd, *J* = 8.6, 7.5 Hz, 2H).

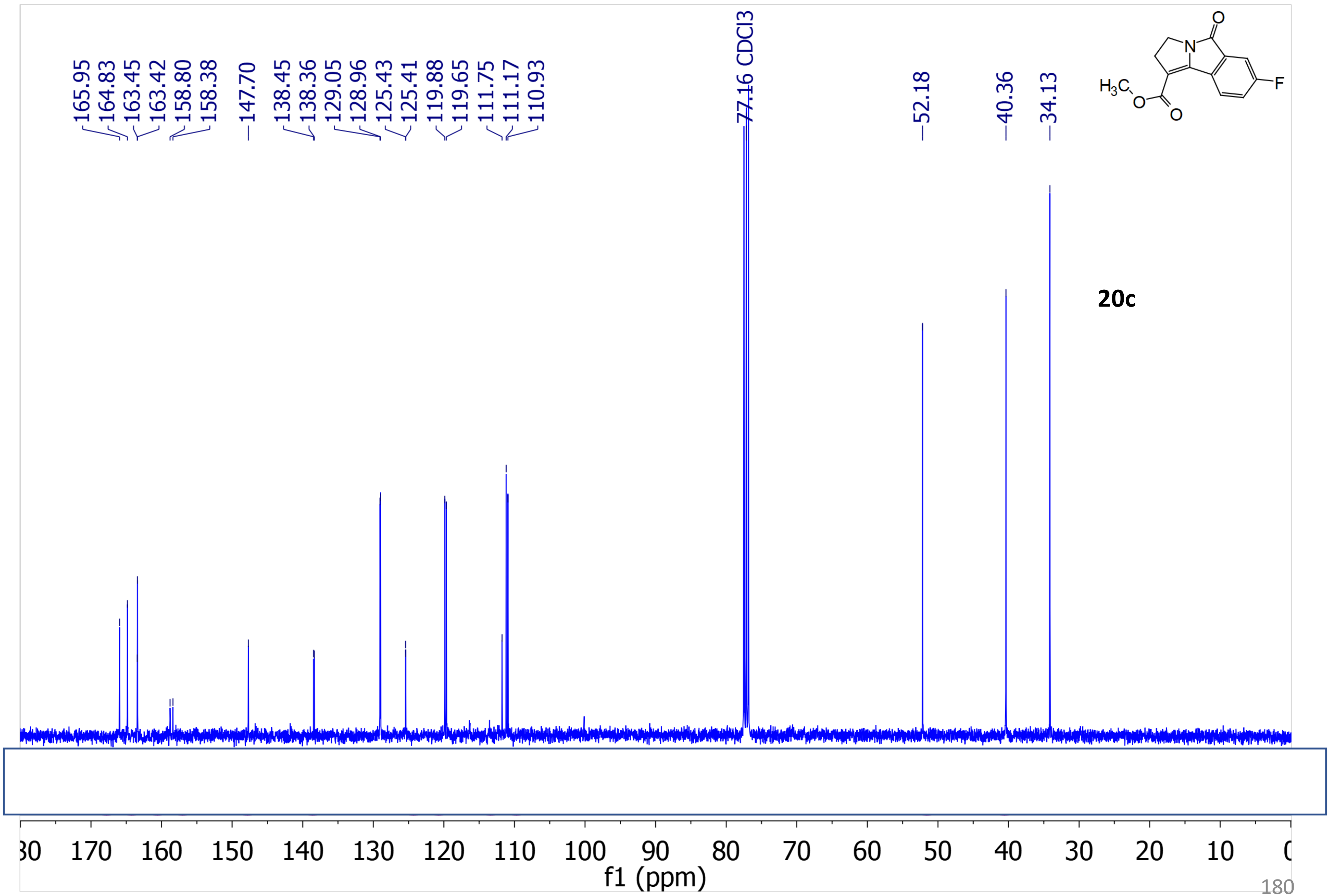
165.95
164.83
163.45
163.42
158.80
158.38
147.70
138.45
138.36
129.05
128.96
125.43
125.41
119.88
119.65
111.75
111.17
110.93
77.16 CDCl3
52.18
40.36
34.13
20c
H3C
O
O
N
O
F
f1 (ppm)
180
170
160
150
140
130
120
110
100
90
80
70
60
50
40
30
20
10
0

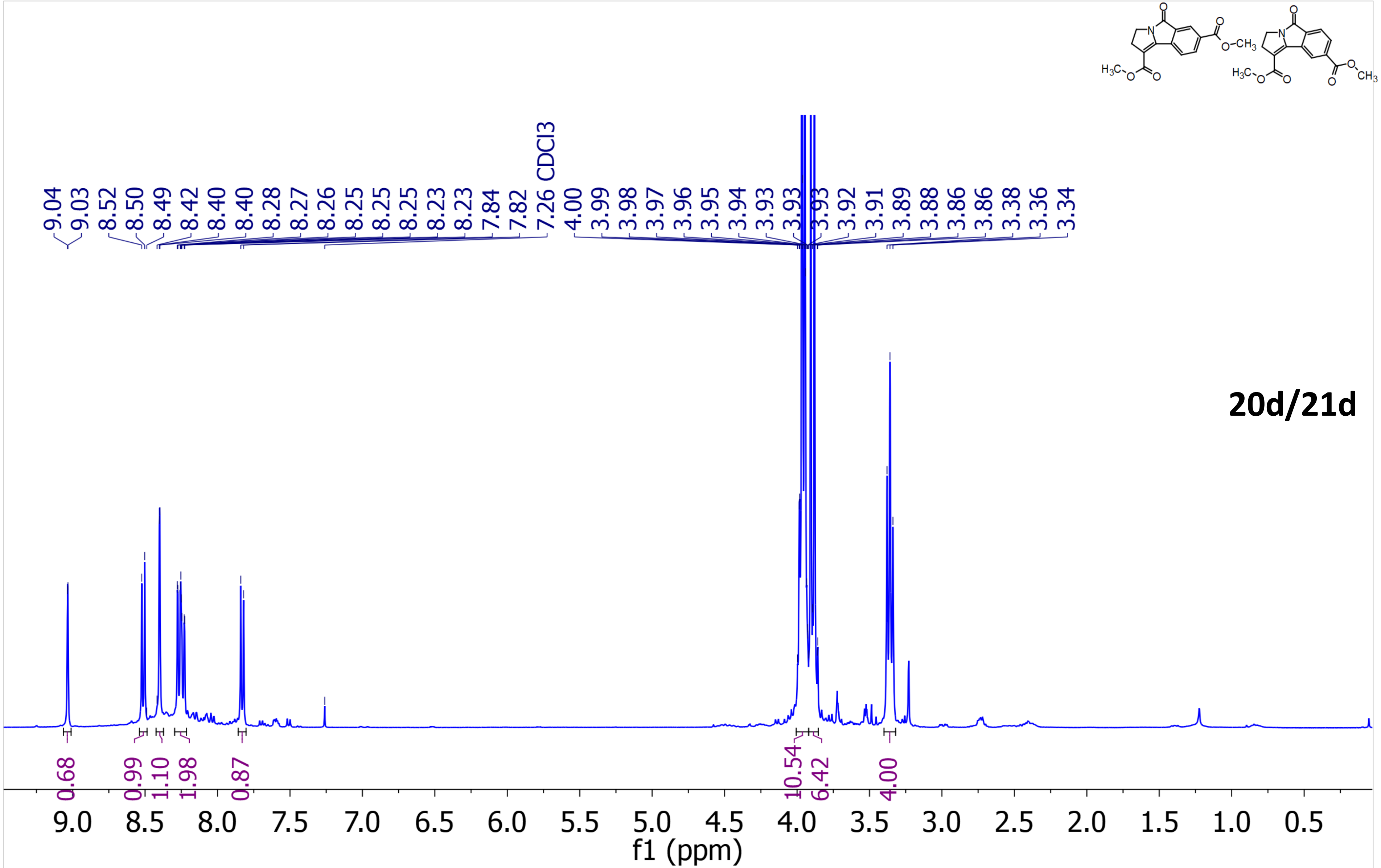

^{1}H NMR (400 MHz, Chloroform-*d*) δ 9.03 (d, *J* = 1.4 Hz, 1H), 8.51 (d, *J* = 8.1 Hz, 1H), 8.40 (d, *J* = 1.5 Hz, 1H), 8.25 (ddd, *J* = 10.7, 8.0, 1.5 Hz, 2H), 7.83 (d, *J* = 7.9 Hz, 1H), 3.95 (dd, *J* = 8.0, 2.0 Hz, 10H), 3.89 (d, *J* = 10.2 Hz, 6H), 3.36 (t, *J* = 8.2 Hz, 4H).

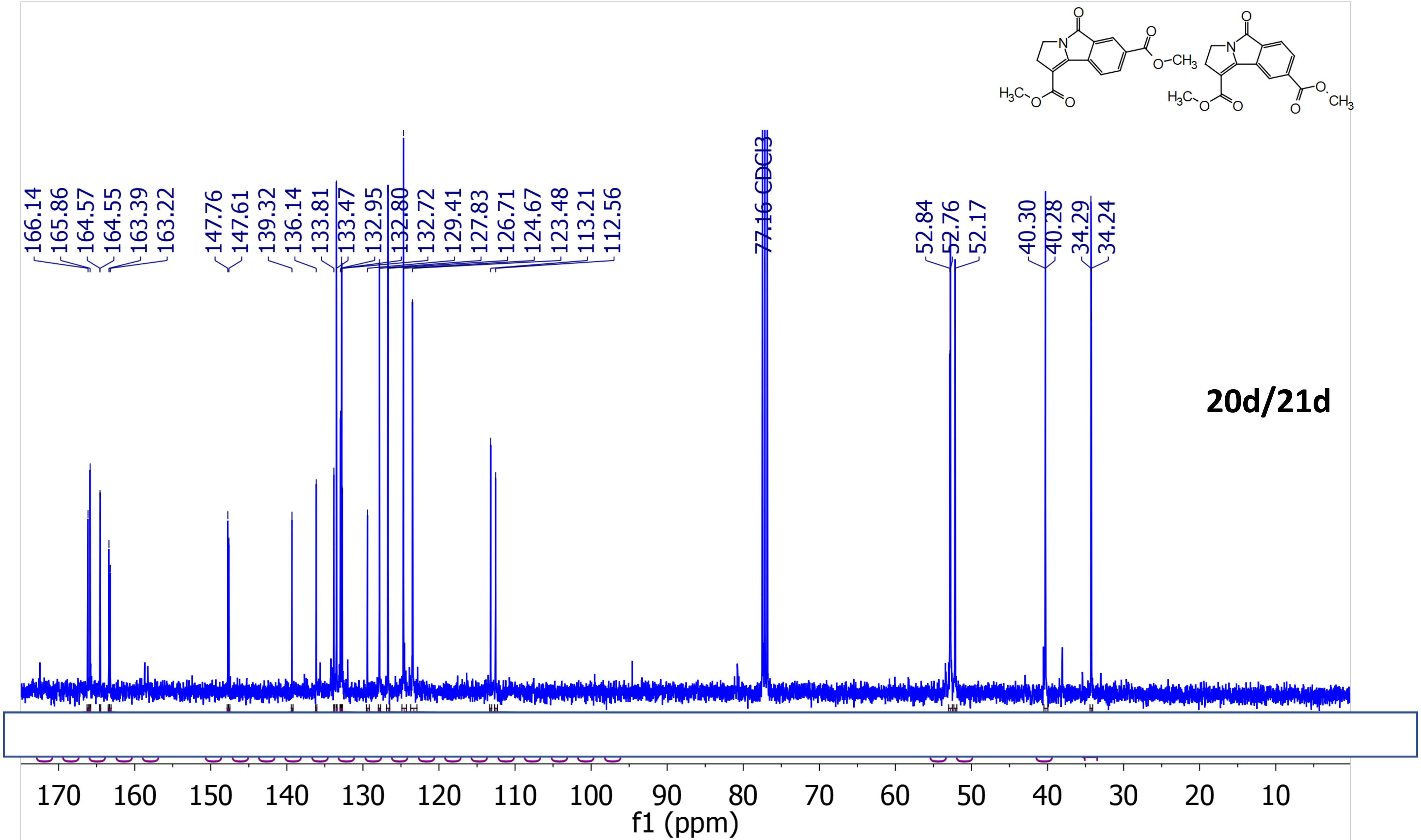

^{13}C NMR (101 MHz, $CDCl_3$) δ 166.14, 165.86, 164.57, 164.55, 163.39, 163.22, 147.76, 147.61, 139.32, 136.14, 133.81, 133.47, 132.95, 132.80, 132.72, 129.41, 127.83, 126.71, 124.67, 123.48, 113.21, 112.56, 52.84, 52.76, 52.17, 40.30, 40.28, 34.29, 34.24.

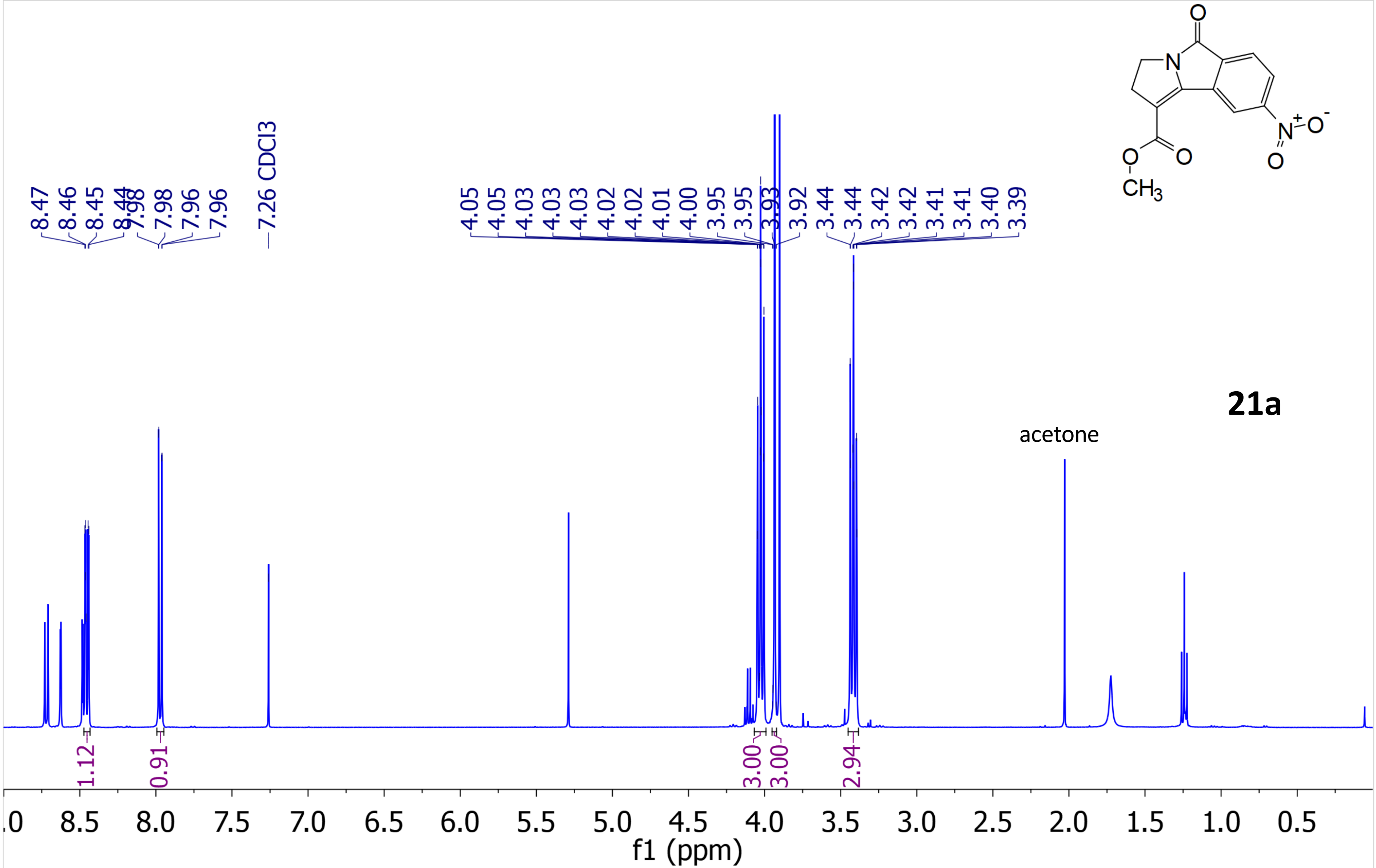

^{1}H NMR (400 MHz, Chloroform-*d*) δ 9.33 (dd, *J* = 2.1, 0.6 Hz, 1H), 8.47 – 8.43 (m, 1H), 7.97 (dd, *J* = 8.3, 0.6 Hz, 1H), 4.03 (t, *J* = 16.4, 7.9 Hz, 2H), 3.93 (s, 3H), 3.42 (t, *J* = 16.4, 8.7 Hz, 2H).

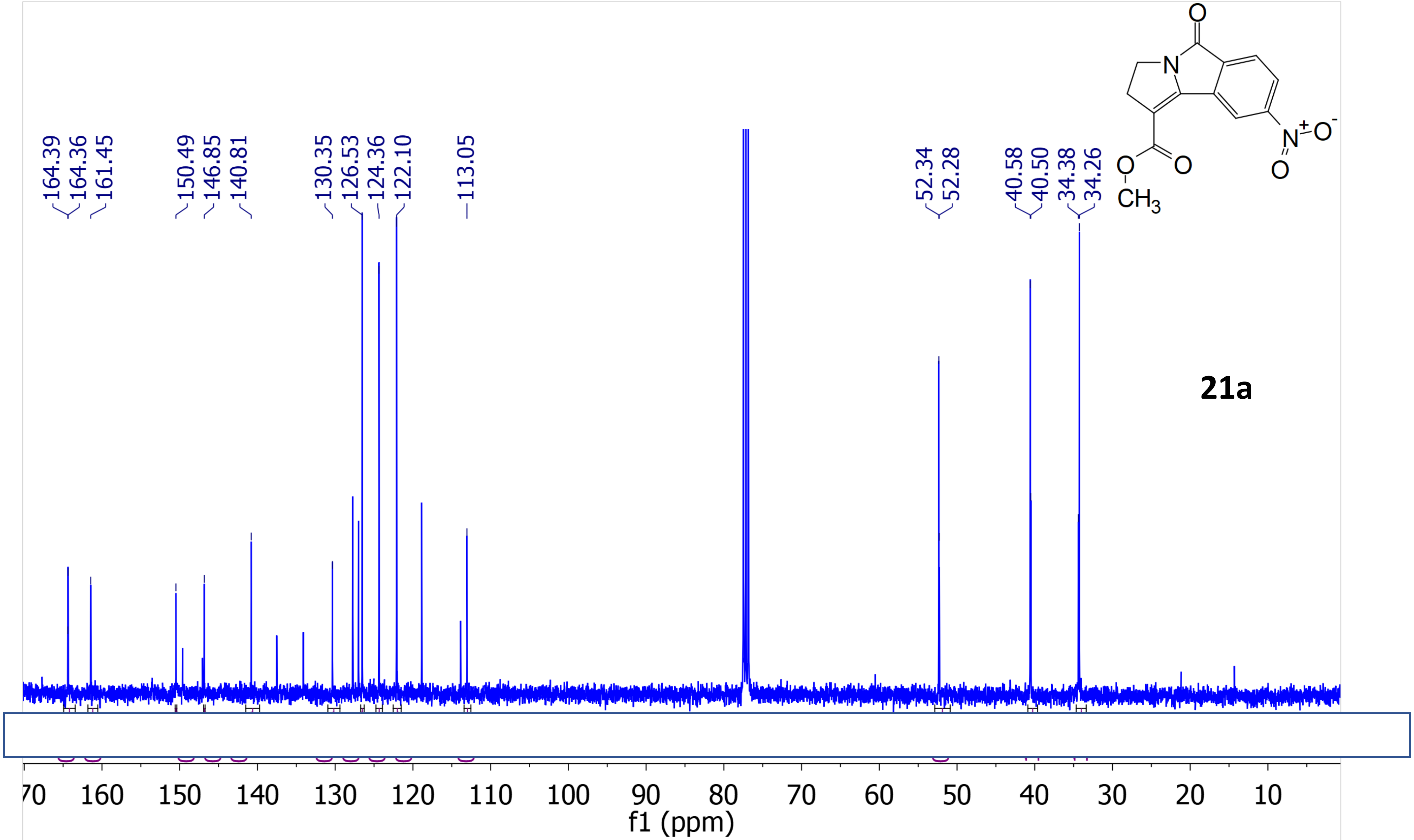

^{13}C NMR (101 MHz, Chloroform-*d*) δ 164.38, 161.45, 150.49, 146.85, 140.81, 130.35, 126.53, 124.36, 122.10, 113.05, 52.31, 40.54, 34.32.

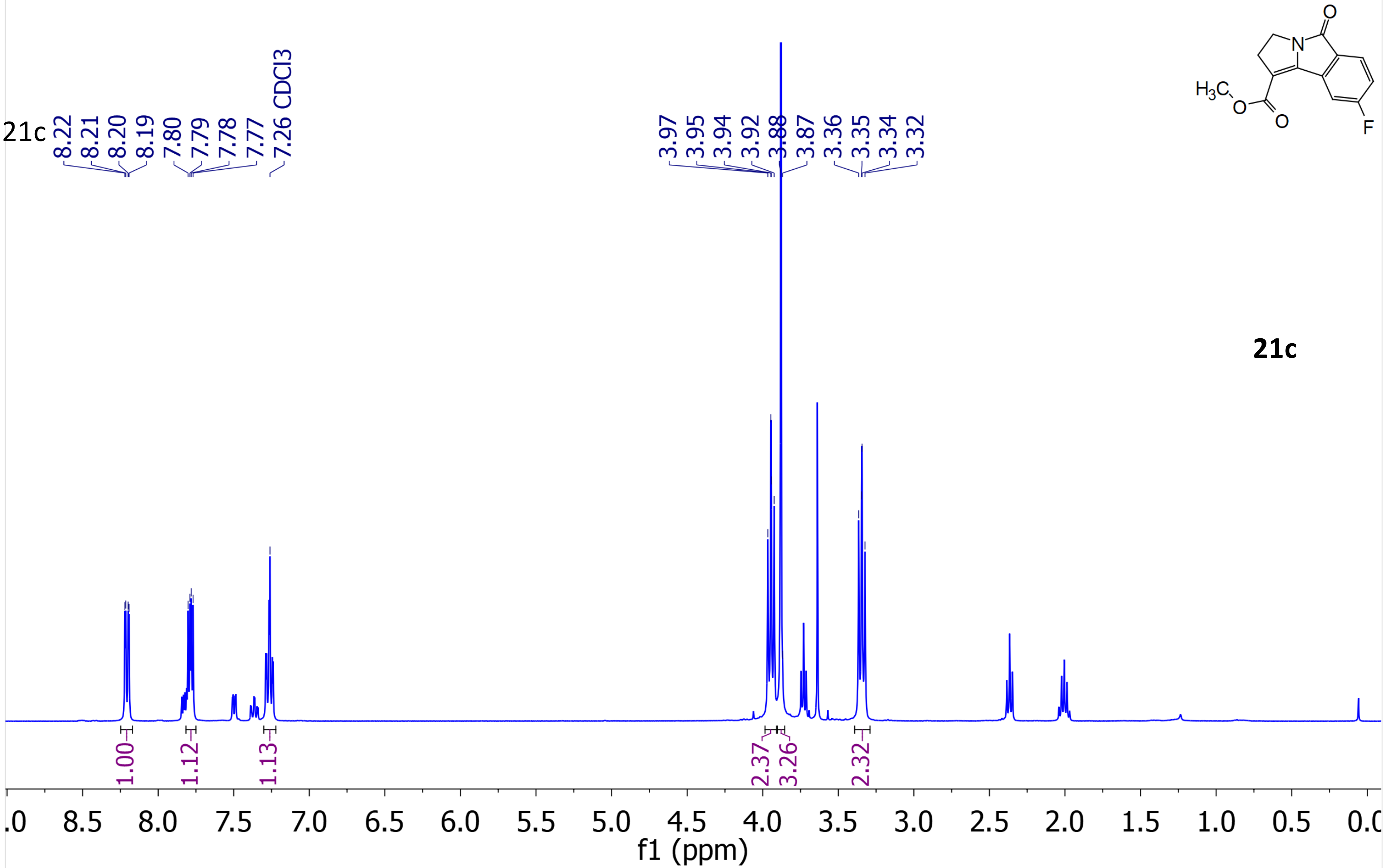

^{1}H NMR (400 MHz, Chloroform-*d*) δ 8.21 (dd, *J* = 8.7, 2.4 Hz, 1H), 7.79 (dd, *J* = 8.4, 4.9 Hz, 1H), 7.26 (s, 1H), 3.94 (dd, *J* = 9.0, 7.8 Hz, 2H), 3.88 (s, 3H), 3.34 (dd, *J* = 9.1, 7.7 Hz, 2H).

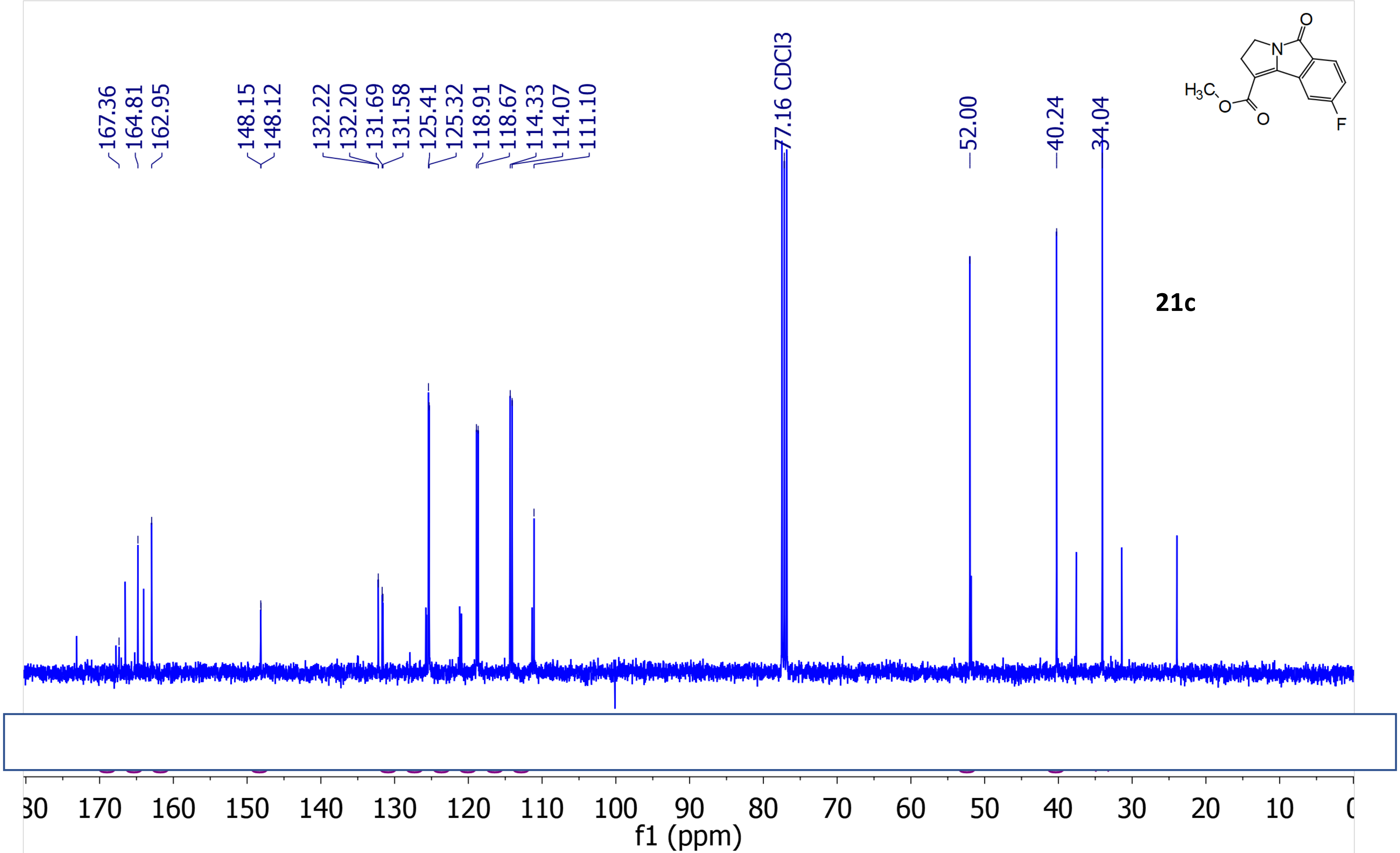

^{13}C NMR (101 MHz, $CDCl_3$) δ 167.36, 164.81, 162.95, 148.15, 148.12, 132.22, 132.20, 131.69, 131.58, 125.41, 125.32, 118.91, 118.67, 114.33, 114.07, 111.10, 77.16, 52.00, 40.24, 34.04.

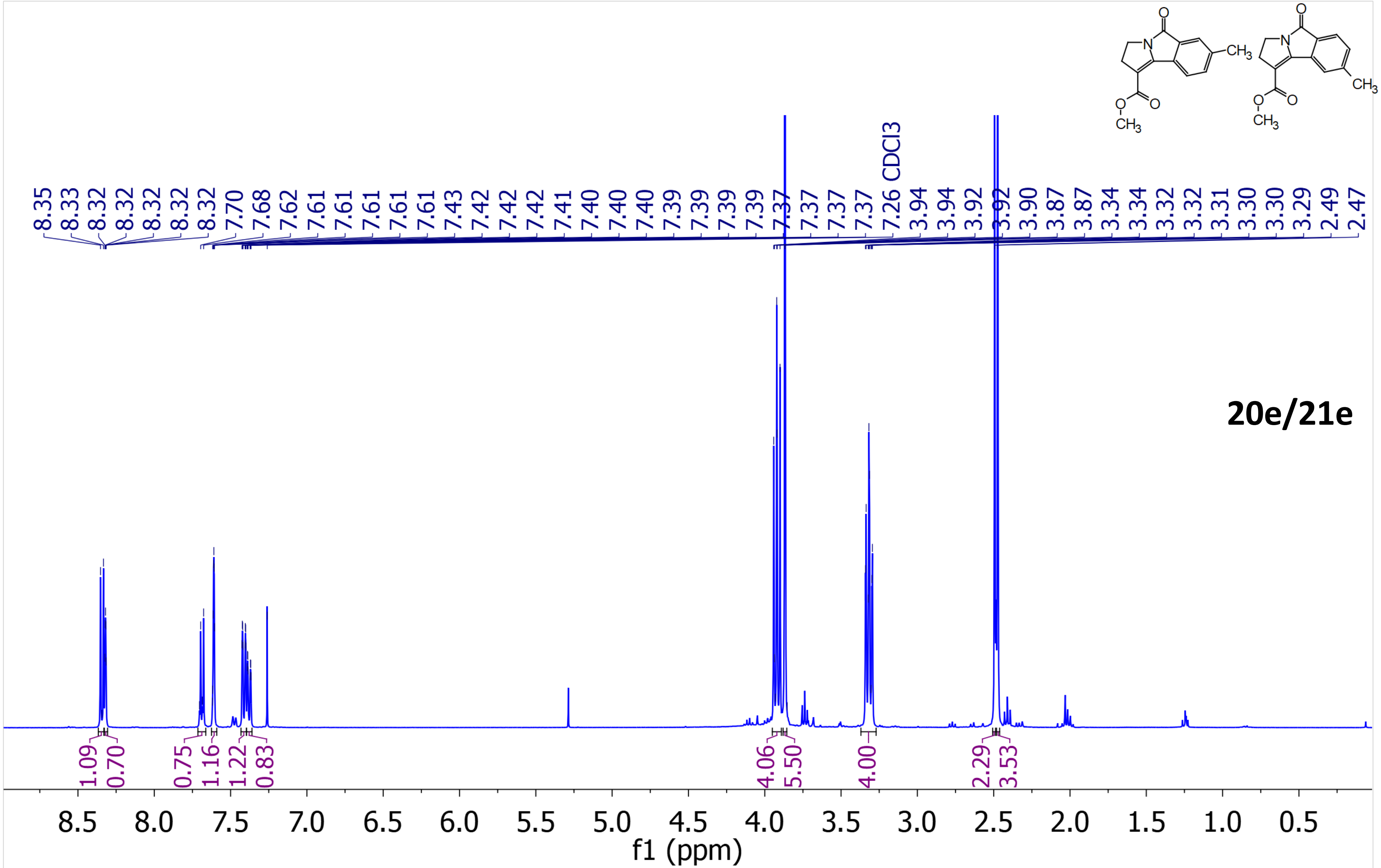

^{1}H NMR (400 MHz, Chloroform-*d*) δ 8.34 (d, *J* = 7.9 Hz, 1H), 8.32 (dt, *J* = 1.6, 0.8 Hz, 1H), 7.71 – 7.66 (m, 1H), 7.61 (dt, *J* = 1.6, 0.8 Hz, 1H), 7.41 (ddd, *J* = 7.9, 1.7, 0.8 Hz, 1H), 7.38 (ddd, *J* = 7.7, 1.5, 0.7 Hz, 1H), 3.92 (dd, *J* = 8.9, 7.7 Hz, 4H), 3.87 (d, *J* = 1.1 Hz, 6H), 3.32 (ddd, *J* = 9.4, 7.6, 1.7 Hz, 4H), 2.49 (s, 3H), 2.47 (s, 3H).

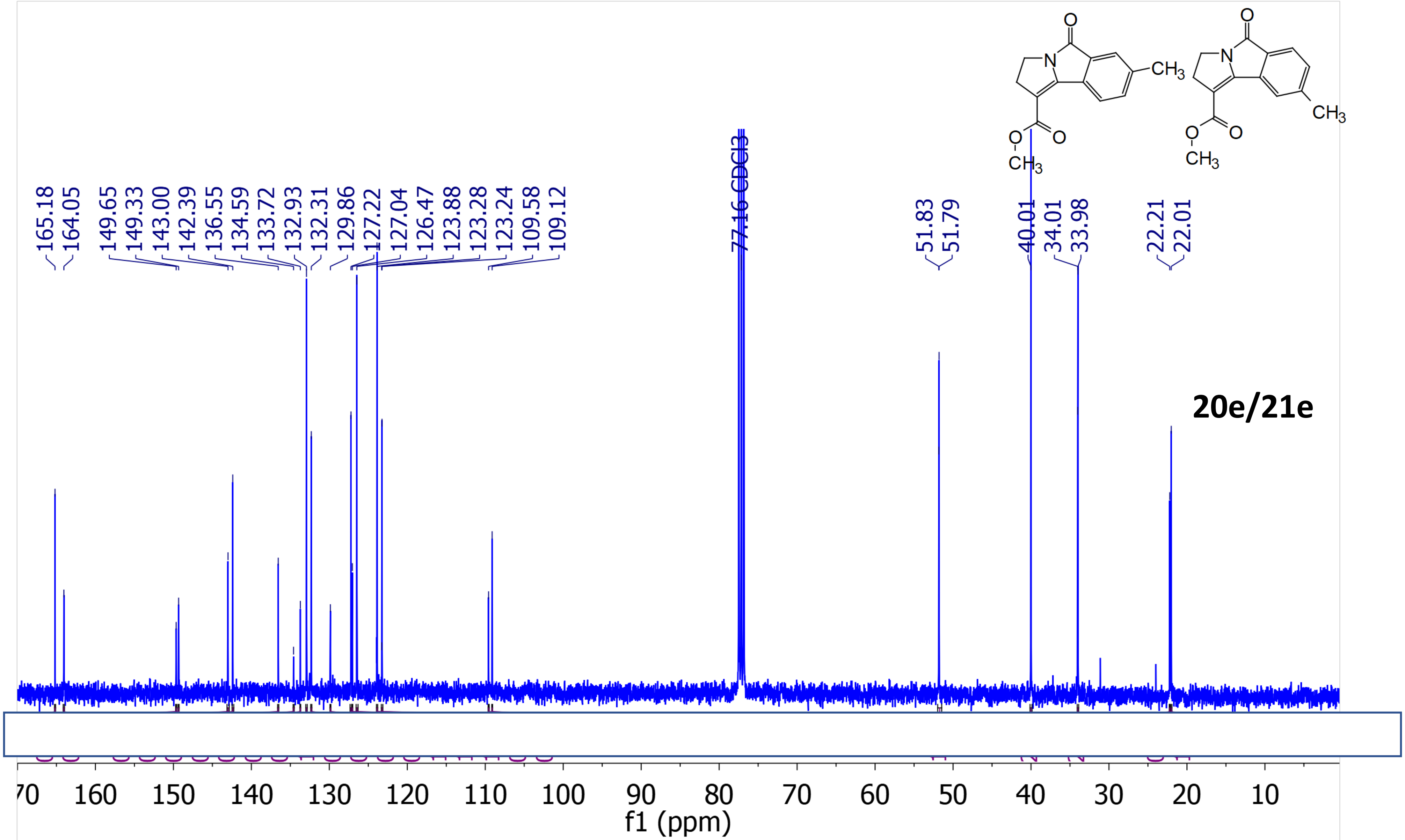

^{13}C NMR (101 MHz, $CDCl_3$) δ 165.18, 164.05, 149.65, 149.33, 143.00, 142.39, 136.55, 134.59, 133.72, 132.93, 132.31, 129.86, 127.22, 127.04, 126.47, 123.88, 123.28, 123.24, 109.58, 109.12, 51.83, 51.79, 40.01, 34.01, 33.98, 22.21, 22.01.

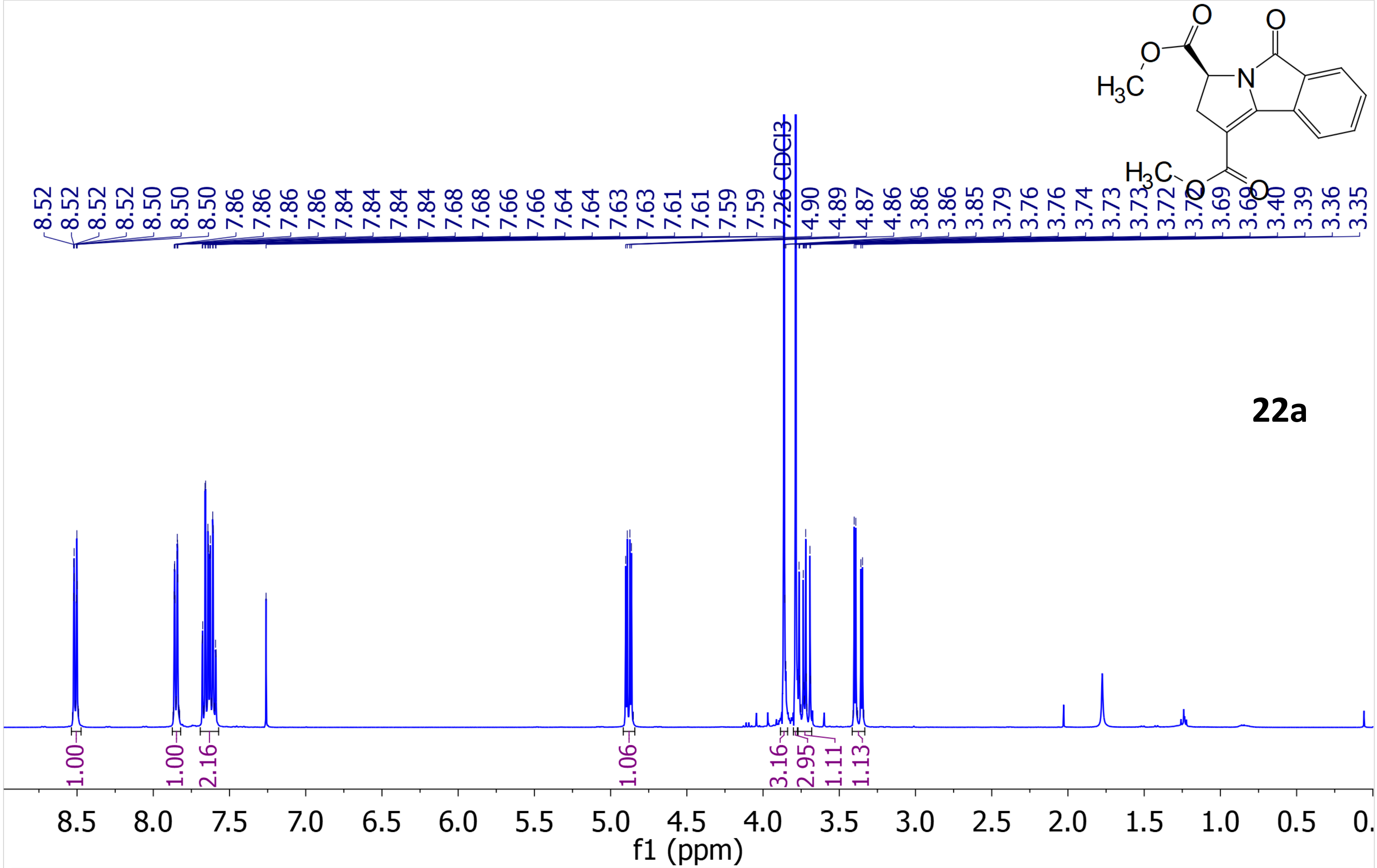

^{1}H NMR (400 MHz, Chloroform-*d*) δ 8.54 – 8.47 (m, 1H), 7.85 (ddd, *J* = 7.3, 1.4, 0.7 Hz, 1H), 7.69 – 7.57 (m, 2H), 4.88 (dd, *J* = 11.0, 4.3 Hz, 1H), 3.86 (s, 3H), 3.79 (s, 3H), 3.72 (dd, 1H), 3.37 (dd, *J* = 17.6, 4.3 Hz, 1H).

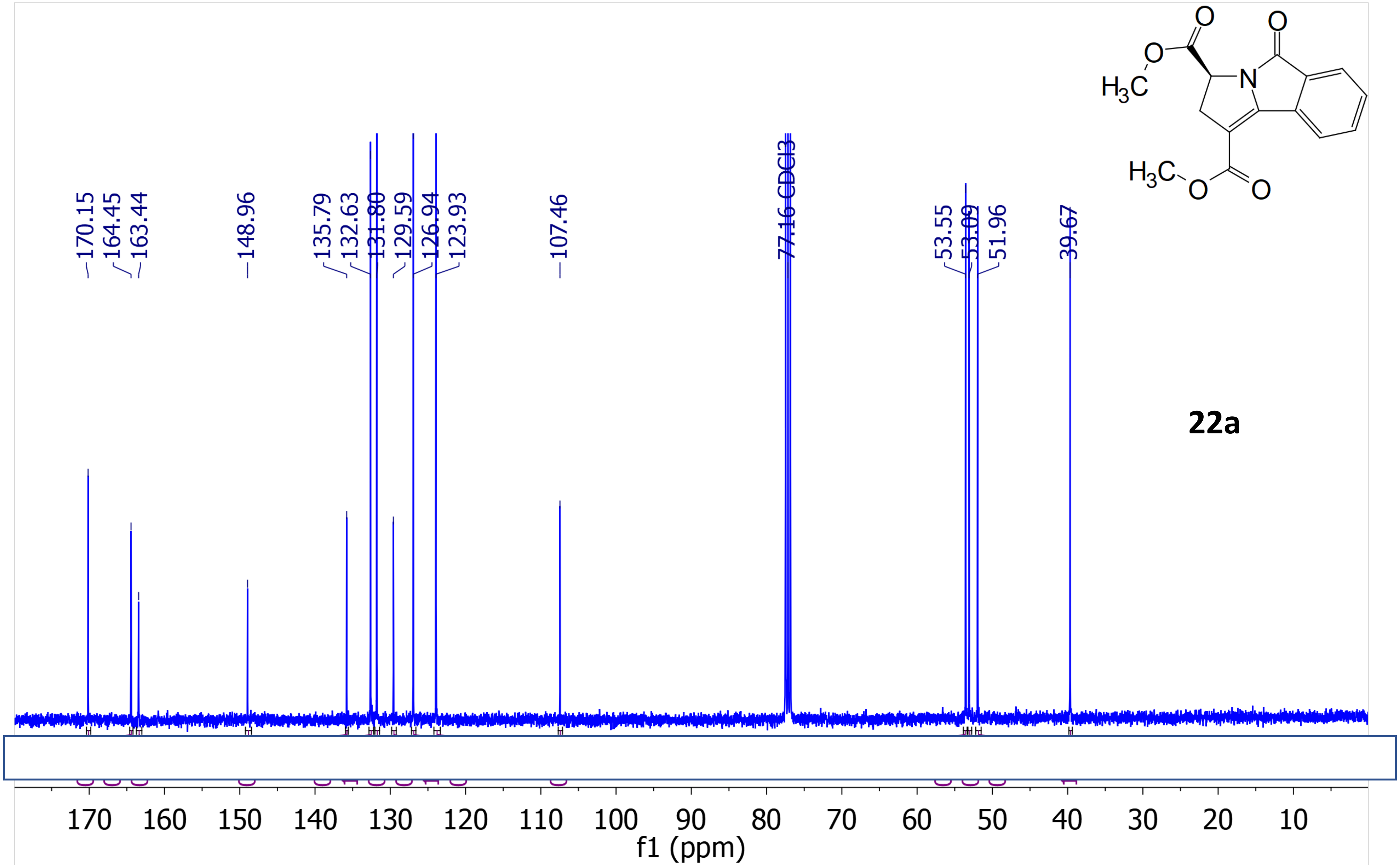

^{13}C NMR (101 MHz, $CDCl_3$) δ 170.15, 164.45, 163.44, 148.96, 135.79, 132.63, 131.80, 129.59, 126.94, 123.93, 107.46, 53.55, 53.09, 51.96, 39.67.

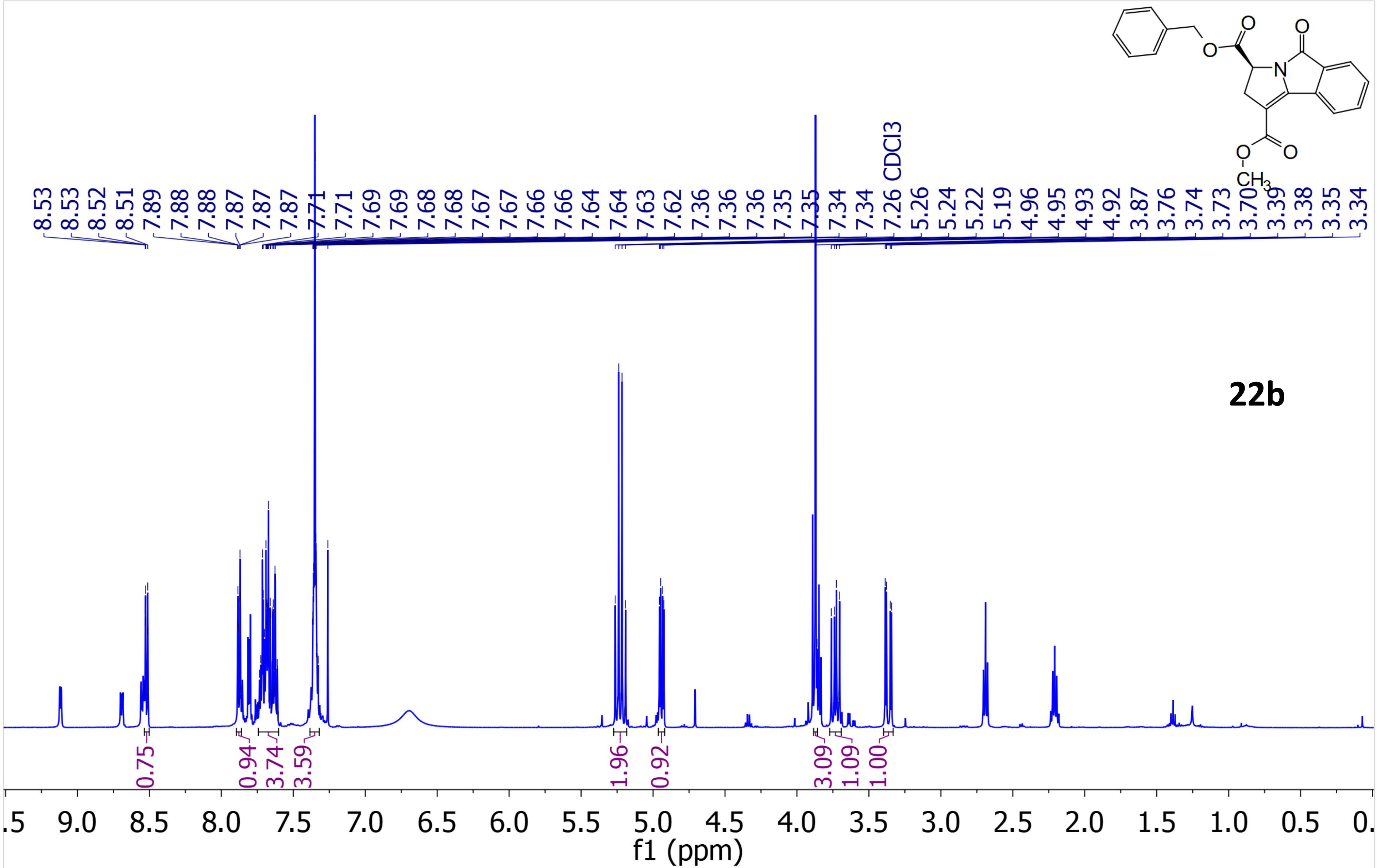

^{1}H NMR (500 MHz, Chloroform-*d*) δ 8.52 (dt, *J* = 7.7, 1.0 Hz, 1H), 7.88 (dt, *J* = 7.3, 1.1 Hz, 1H), 7.74 – 7.60 (m, 3H), 7.38 – 7.32 (m, 4H), 5.23 (q, *J* = 12.2 Hz, 2H), 4.94 (dd, *J* = 11.0, 4.3 Hz, 1H), 3.87 (s, 3H), 3.77 – 3.69 (m, 1H), 3.36 (dd, *J* = 17.6, 4.3 Hz, 1H).

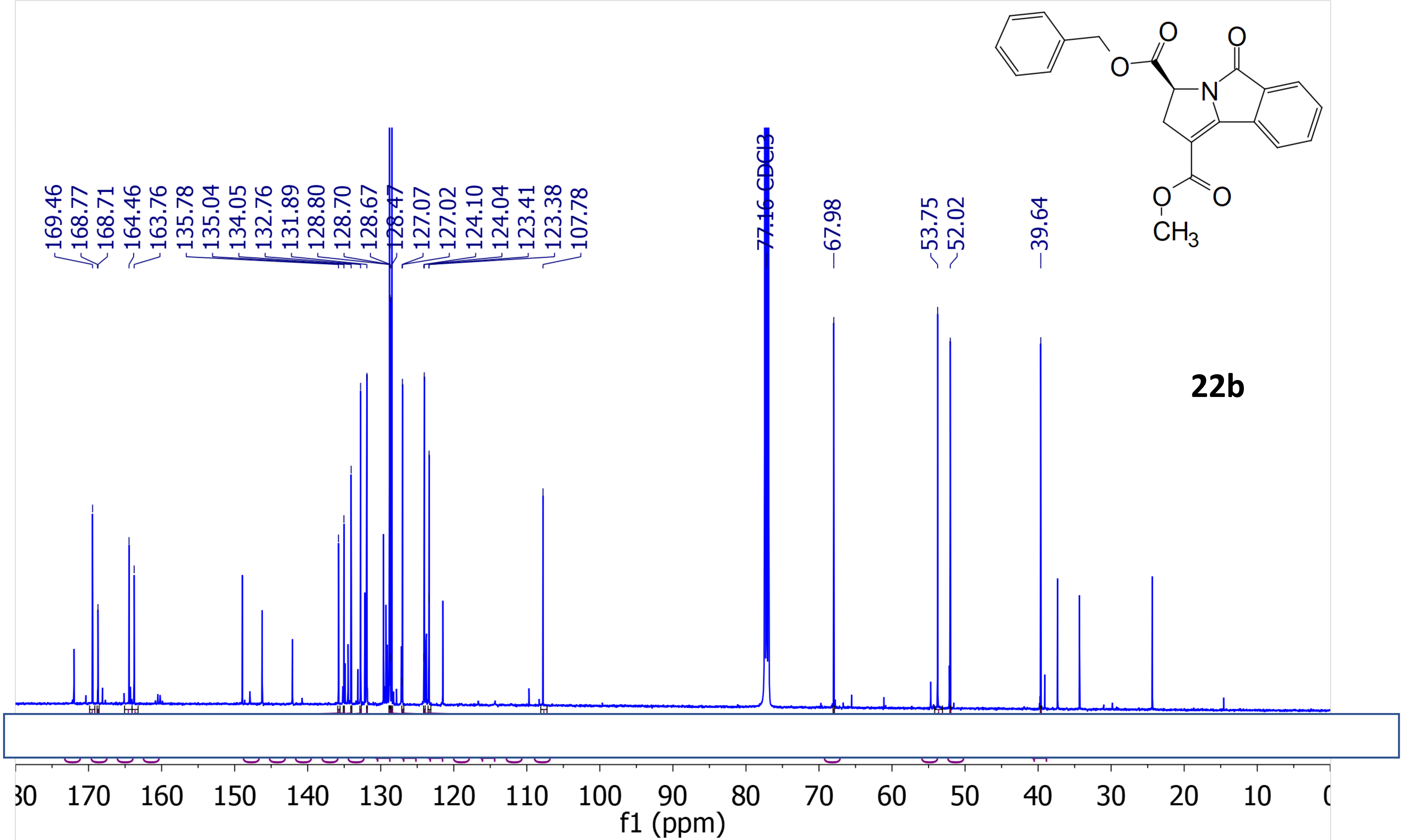

^{13}C NMR (126 MHz, Chloroform-*d*) δ 169.46, 168.77, 168.71, 164.46, 163.76, 135.78, 135.04, 134.05, 132.76, 131.89, 128.80, 128.70, 128.67, 128.47, 127.07, 127.02, 124.10, 124.04, 123.41, 123.38, 107.78, 67.98, 53.75, 52.02, 39.64.

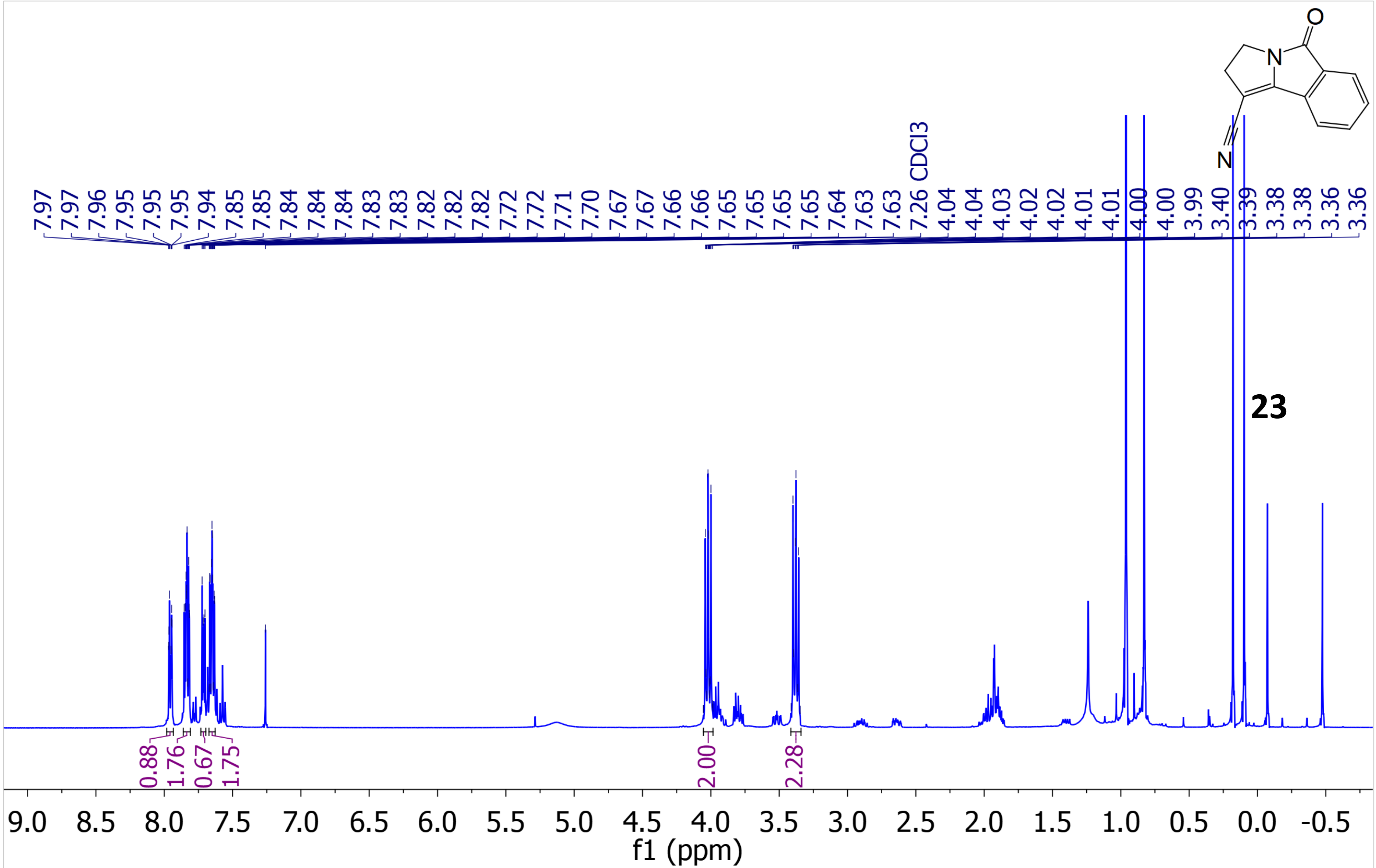

^{1}H NMR (400 MHz, Chloroform-*d*) δ 7.98 – 7.93 (m, 1H), 7.86 – 7.81 (m, 1H), 7.71 (dd, *J* = 5.4, 3.1 Hz, 1H), 7.65 (ddd, *J* = 7.6, 5.5, 1.4 Hz, 1H), 4.02 (dd, *J* = 8.9, 7.7 Hz, 2H), 3.38 (dd, *J* = 9.1, 7.6 Hz, 2H).

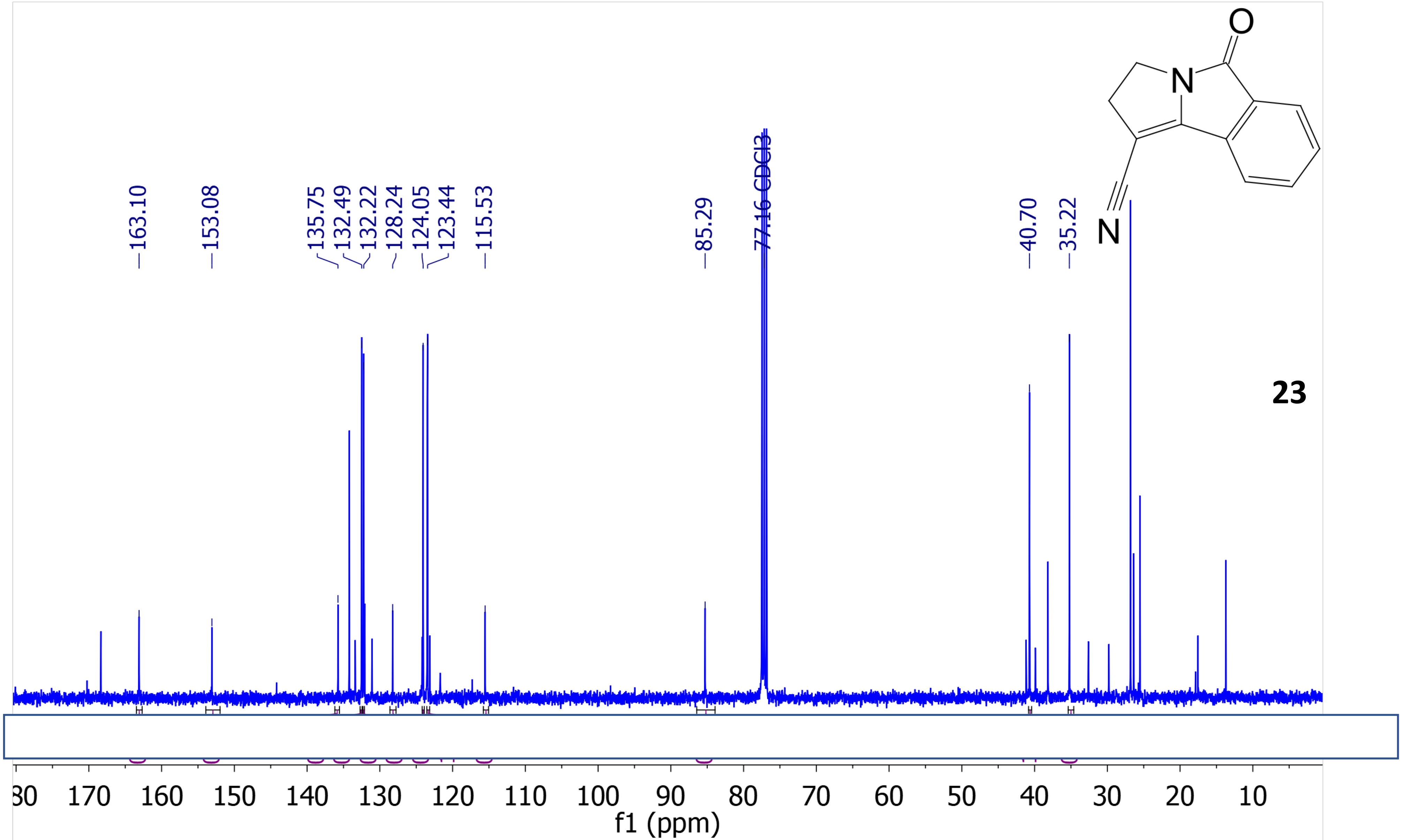

^{13}C NMR (101 MHz, $CDCl_3$) δ 163.10, 153.08, 135.75, 132.49, 132.22, 128.24, 124.05, 123.44, 115.53, 85.29, 40.70, 35.22.

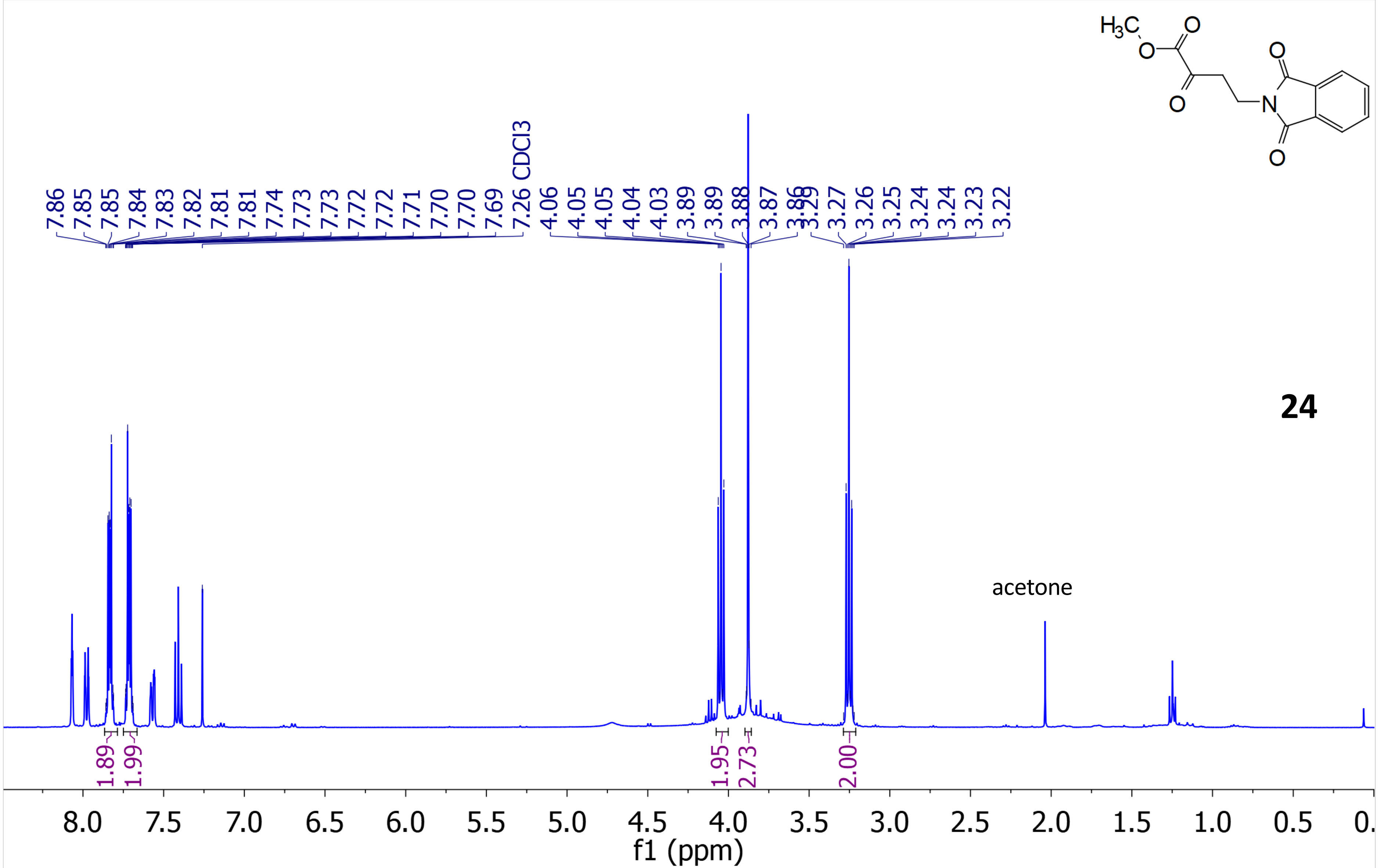

^{1}H NMR (400 MHz, Chloroform-*d*) δ 7.83 (dd, J = 5.4, 3.1 Hz, 2H), 7.72 (dd, 2H), 4.05 (t, J = 7.0 Hz, 2H), 3.88 (s, 3H), 3.25 (t, J = 6.9 Hz, 2H).

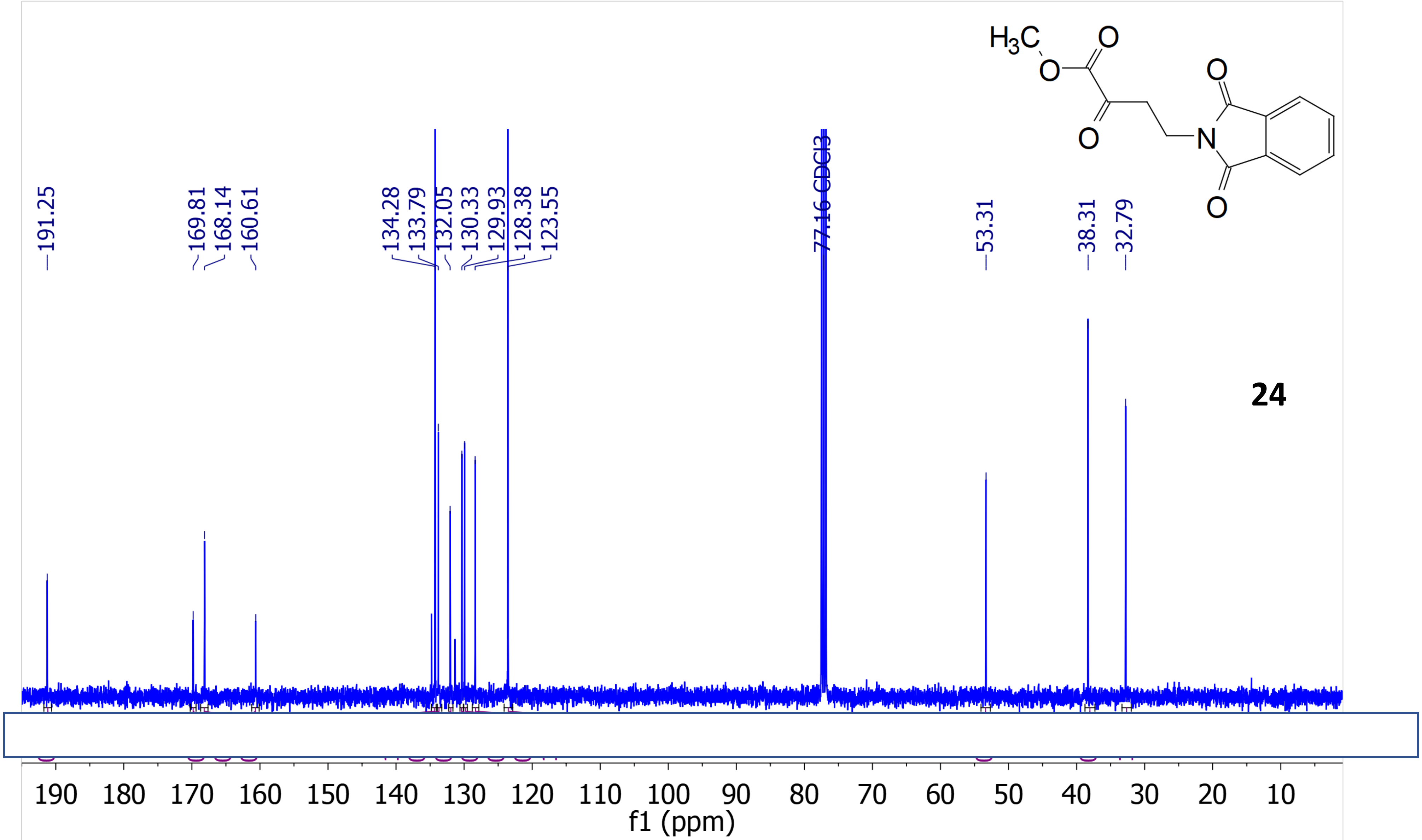

^{13}C NMR (101 MHz, $CDCl_3$) δ 191.25, 169.81, 168.14, 160.61, 134.28, 133.79, 132.05, 130.33, 129.93, 128.38, 123.55, 53.31, 38.31, 32.79.

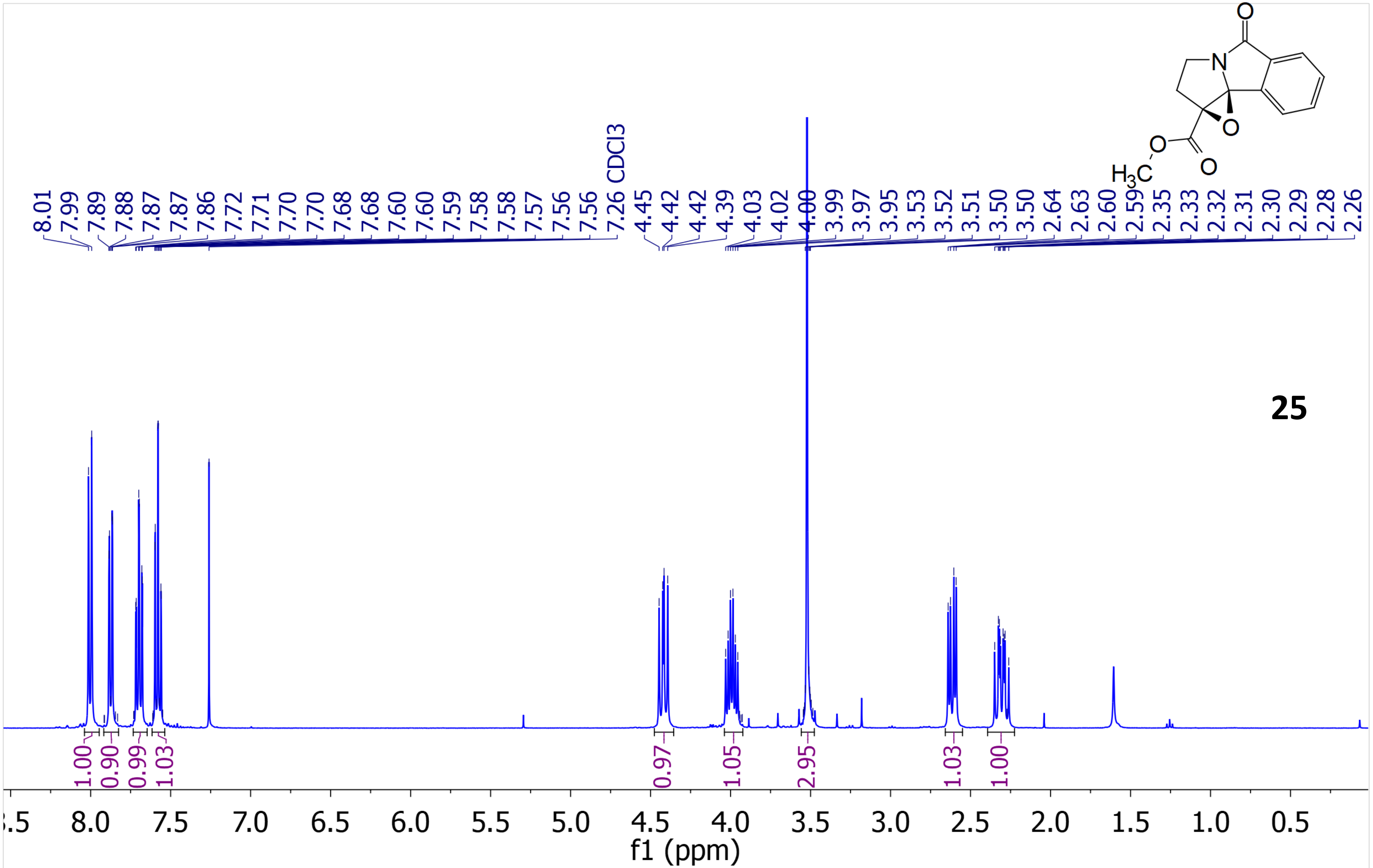

^{1}H NMR (400 MHz, Chloroform-*d*) δ 7.94 (d, *J* = 7.9 Hz, 1H), 7.81 (dd, *J* = 7.7, 1.3 Hz, 1H), 7.63 (td, *J* = 7.6, 1.3 Hz, 1H), 7.51 (td, *J* = 7.5, 0.8 Hz, 1H), 4.35 (dd, *J* = 12.5, 9.1 Hz, 1H), 3.93 (td, *J* = 12.0, 6.1 Hz, 1H), 3.46 (s, 3H), 2.55 (dd, *J* = 14.3, 6.0 Hz, 1H), 2.24 (ddd, *J* = 14.2, 11.7, 9.2 Hz, 1H).

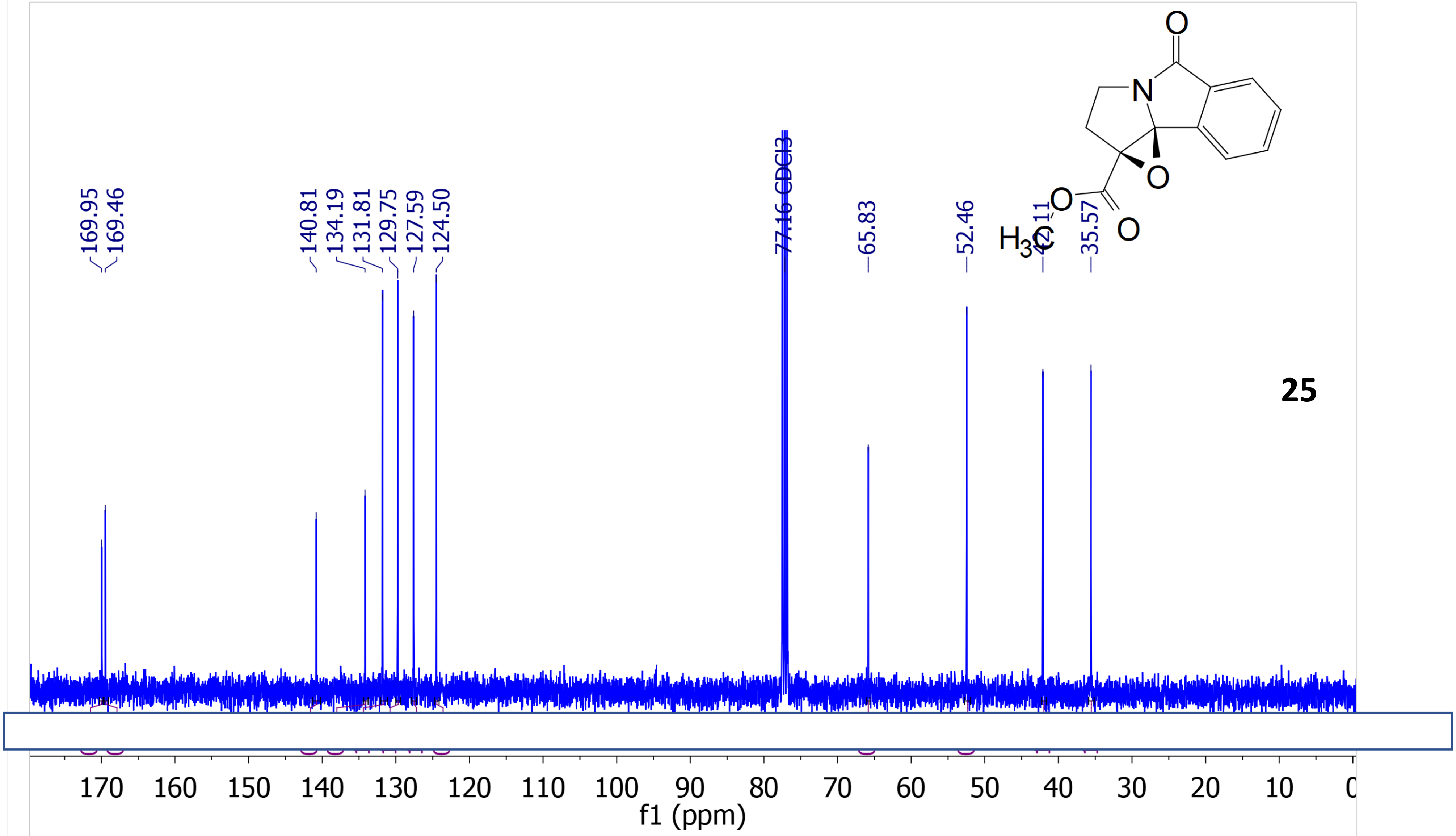

^{13}C NMR (101 MHz, $CDCl_3$) δ 169.95, 169.46, 140.81, 134.19, 131.81, 129.75, 127.59, 124.50, 65.83, 52.46, 42.11, 35.57.

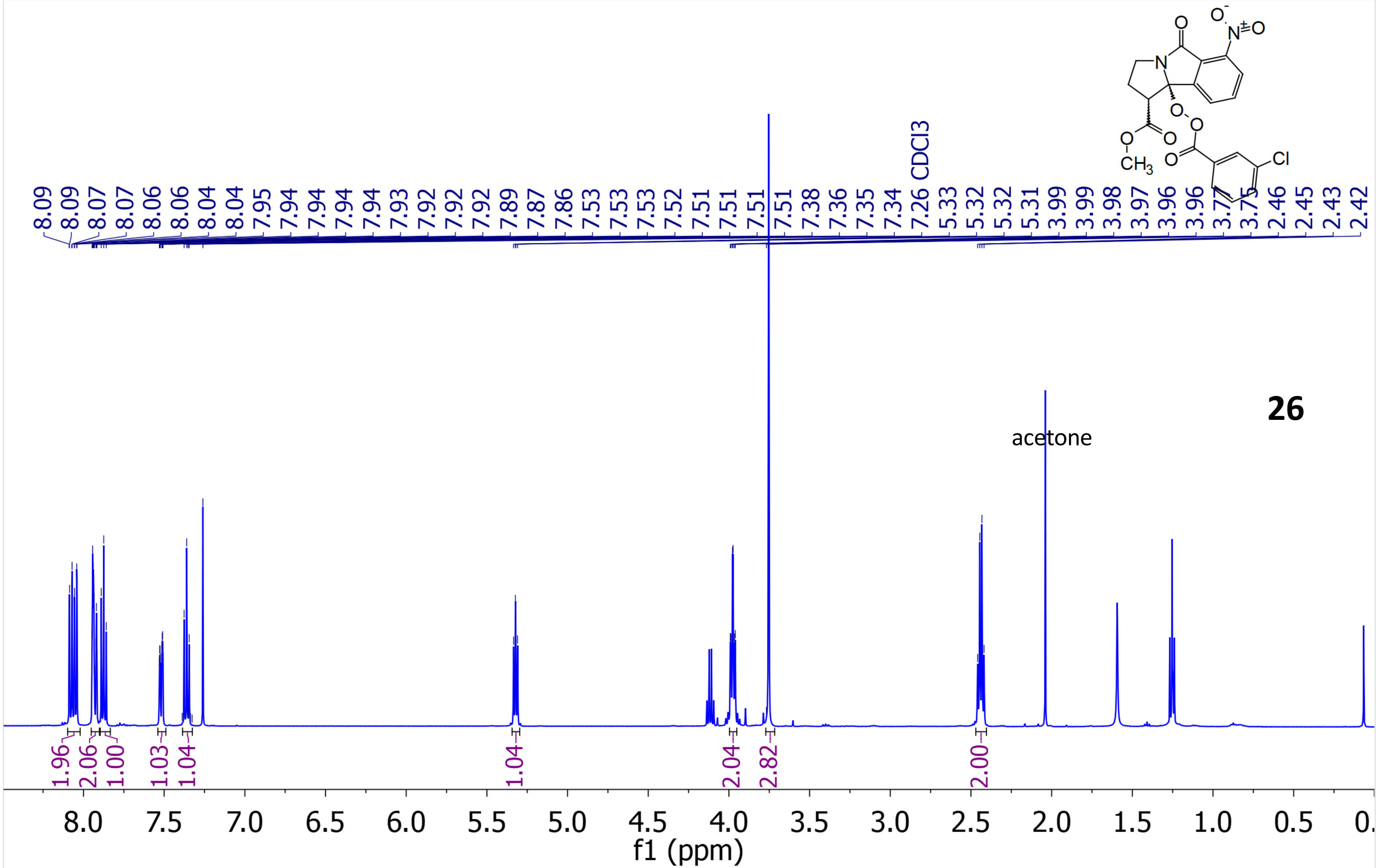

^{1}H NMR (500 MHz, Chloroform-*d*) δ 8.06 (ddd, *J* = 15.2, 7.8, 0.9 Hz, 2H), 7.95 – 7.90 (m, 2H), 7.87 (t, *J* = 7.8 Hz, 1H), 7.52 (ddd, *J* = 8.0, 2.2, 1.1 Hz, 1H), 7.36 (t, *J* = 7.9 Hz, 1H), 5.32 (dd, *J* = 6.8, 5.8 Hz, 1H), 3.98 (td, *J* = 6.7, 1.9 Hz, 2H), 3.75 (s, 3H), 2.44 (q, *J* = 6.7 Hz, 2H).

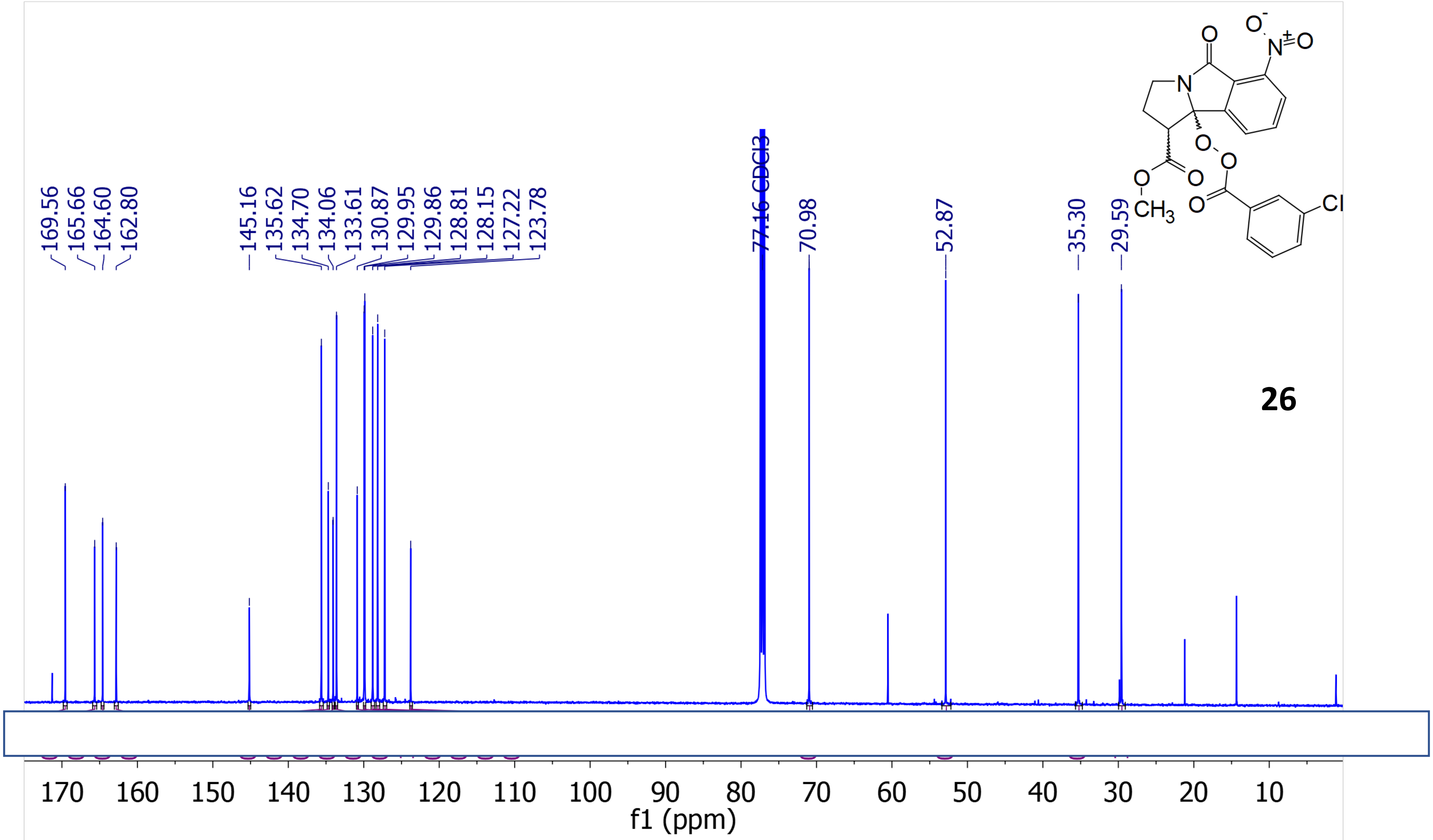

^{13}C NMR (126 MHz, $CDCl_3$) δ 169.56, 165.66, 164.60, 162.80, 145.16, 135.62, 134.70, 134.06, 133.61, 130.87, 129.95, 129.86, 128.81, 128.15, 127.22, 123.78, 70.98, 52.87, 35.30, 29.59.

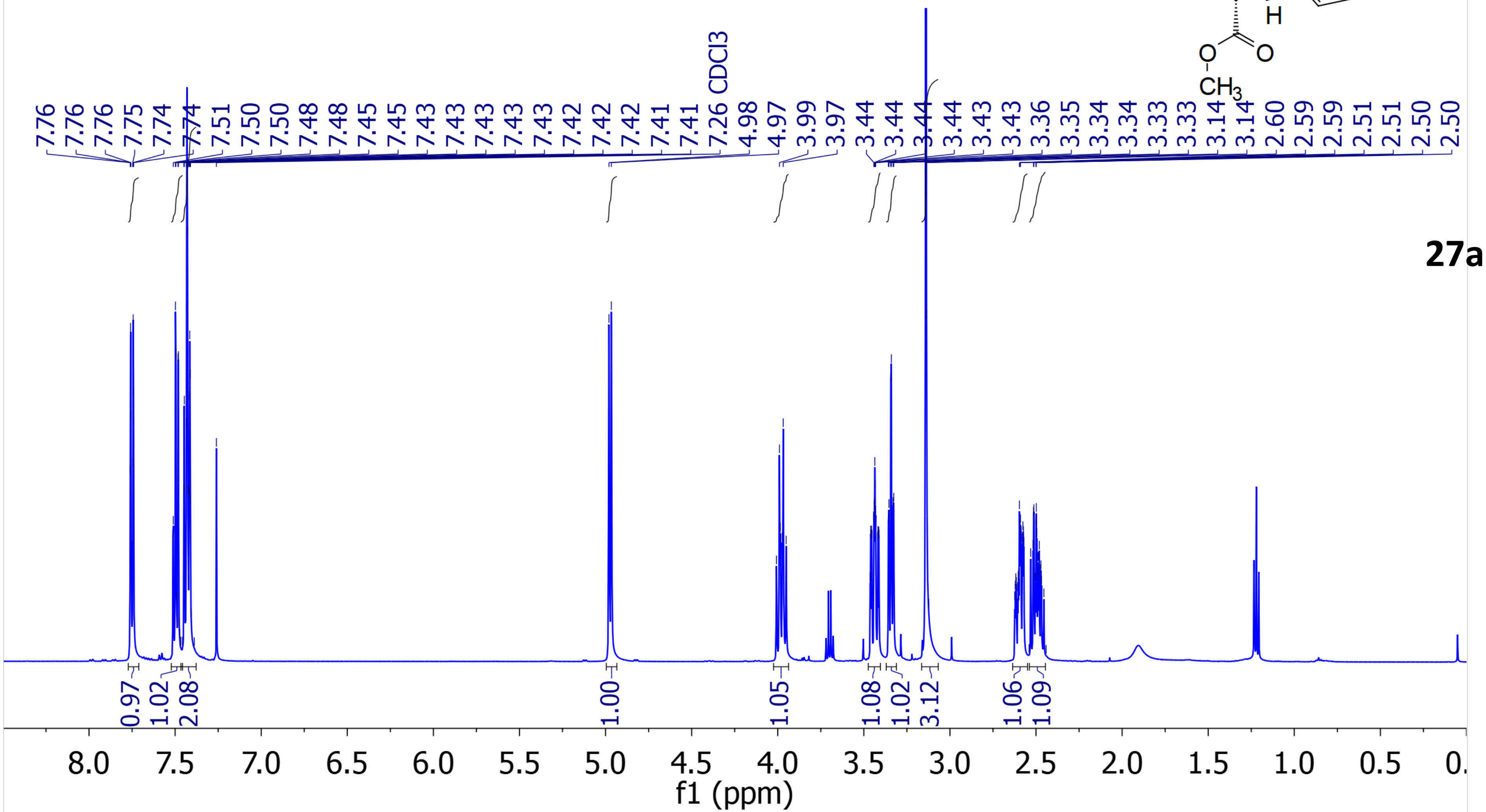

27a

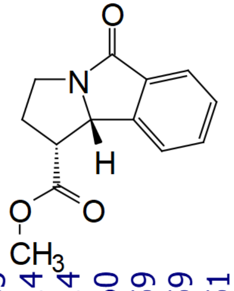

^{1}H NMR (500 MHz, Chloroform-*d*) δ 7.75 (dt, *J* = 7.5, 1.1 Hz, 1H), 7.50 (td, *J* = 7.4, 1.2 Hz, 1H), 7.47 – 7.38 (m, 2H), 4.97 (d, *J* = 6.9 Hz, 1H), 3.98 (dt, *J* = 11.2, 8.5 Hz, 1H), 3.44 (dtd, 1H), 3.34 (td, *J* = 7.0, 1.6 Hz, 1H), 3.14 (s, 4H), 2.60 (dddd, *J* = 13.2, 8.4, 3.0, 1.6 Hz, 1H), 2.49 (dddd, *J* = 13.5, 9.4, 8.6, 7.0 Hz, 1H).

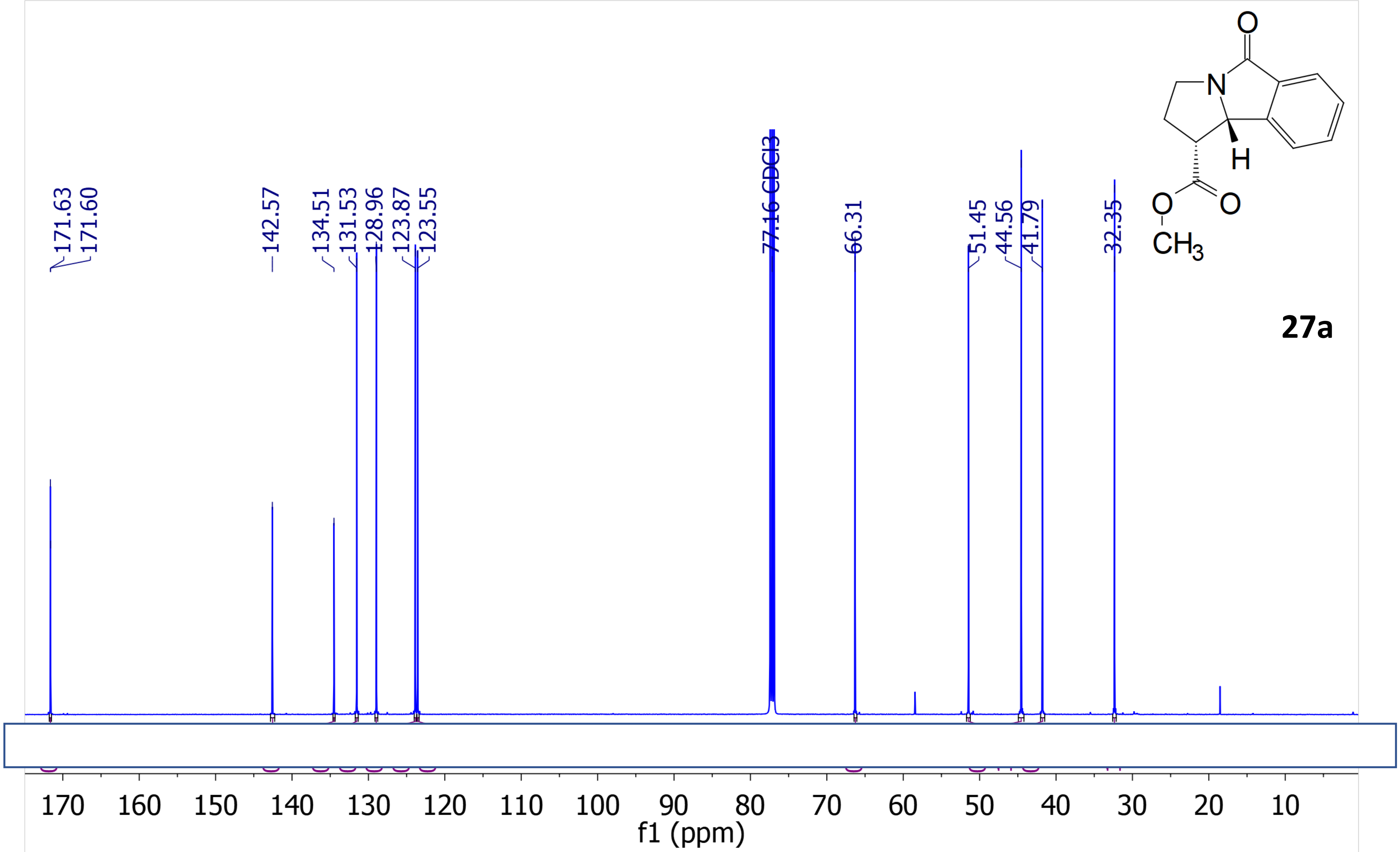

^{13}C NMR (126 MHz, $CDCl_3$) δ 171.63, 171.60, 142.57, 134.51, 131.53, 128.96, 123.87, 123.55, 66.31, 51.45, 44.56, 41.79, 32.35.

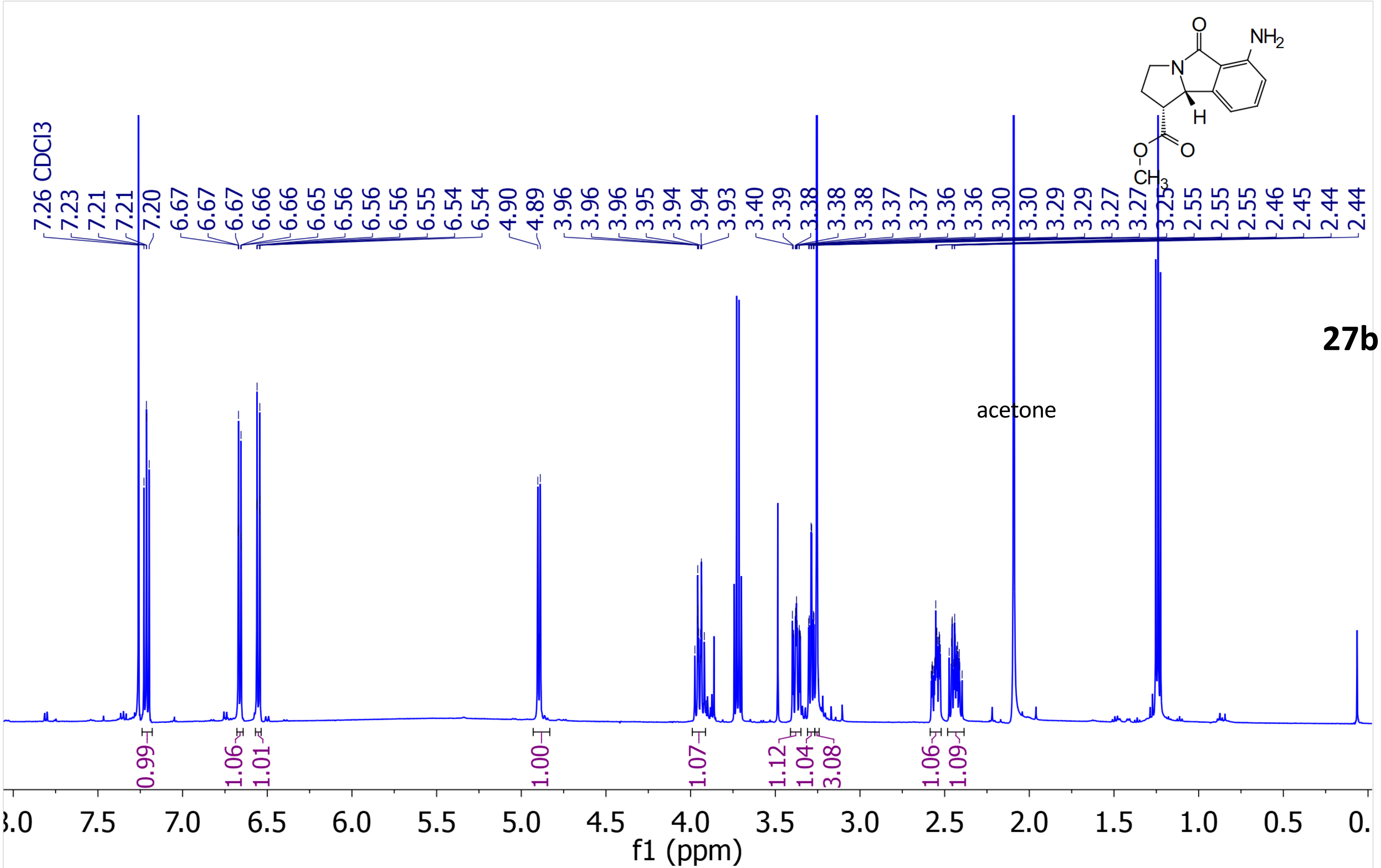

^{1}H NMR (500 MHz, Chloroform-*d*) δ 7.21 (dd, *J* = 8.1, 7.4 Hz, 1H), 6.66 (dd, *J* = 7.4, 0.9 Hz, 1H), 6.55 (dd, *J* = 8.2, 0.8 Hz, 1H), 4.90 (d, *J* = 7.2 Hz, 1H), 3.95 (dd, *J* = 11.2, 8.4 Hz, 1H), 3.37 (ddd, *J* = 11.2, 9.5, 3.2 Hz, 1H), 3.29 (dt, *J* = 7.0, 1.9 Hz, 1H), 3.25 (s, 3H), 2.55 (dddd, *J* = 13.5, 8.4, 3.2, 1.9 Hz, 1H), 2.44 (dddd, *J* = 13.5, 9.3, 8.4, 7.0 Hz, 1H).

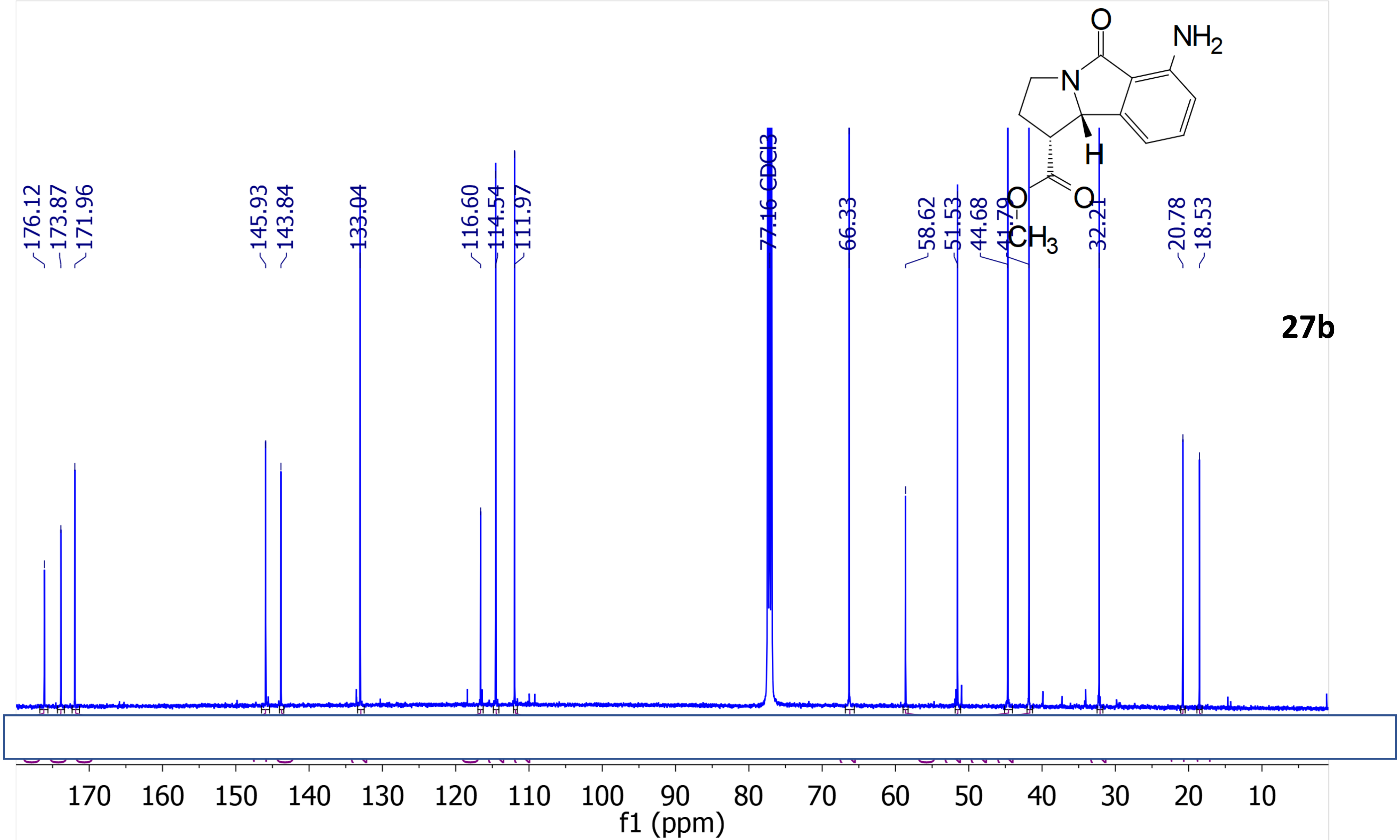

27b

^{13}C NMR (126 MHz, $CDCl_3$) δ 176.12, 173.87, 171.96, 145.93, 143.84, 133.04, 116.60, 114.54, 111.97, 66.33, 58.62, 51.53, 44.68, 41.79, 32.21, 20.78, 18.53.

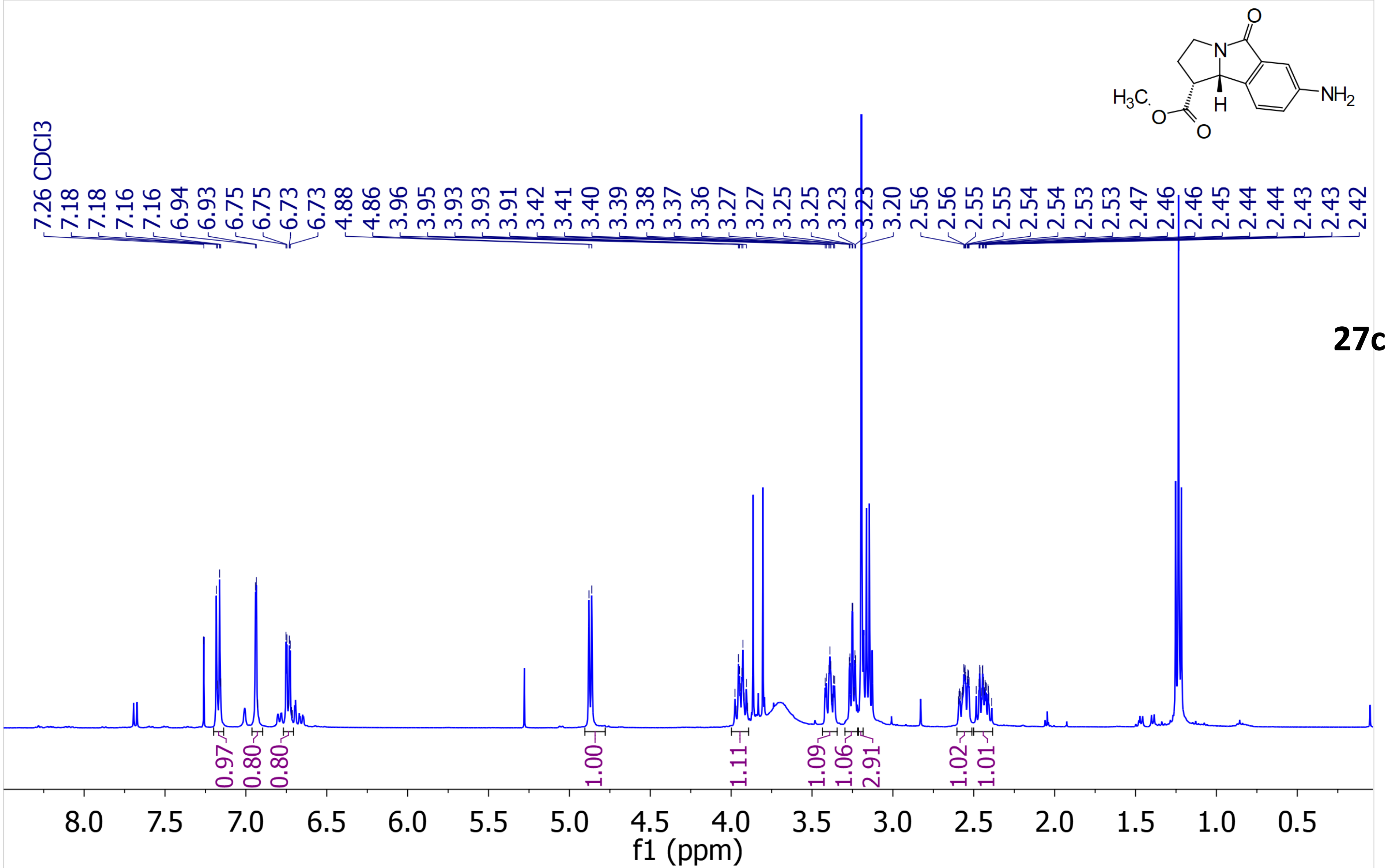

27c

^{1}H NMR (400 MHz, Chloroform-*d*) δ 7.17 (dd, *J* = 8.1, 2.7 Hz, 1H), 6.94 (d, *J* = 2.3 Hz, 1H), 6.74 (dd, *J* = 8.2, 2.3 Hz, 1H), 4.87 (d, *J* = 6.9 Hz, 1H), 3.94 (dtd, *J* = 11.4, 8.5, 2.7 Hz, 1H), 3.39 (ddd, *J* = 11.6, 9.3, 2.9 Hz, 1H), 3.25 (td, *J* = 6.9, 1.5 Hz, 1H), 3.20 (s, 3H), 2.56 (dddd, *J* = 13.1, 8.5, 3.0, 1.6 Hz, 1H), 2.50 – 2.38 (m, 1H).

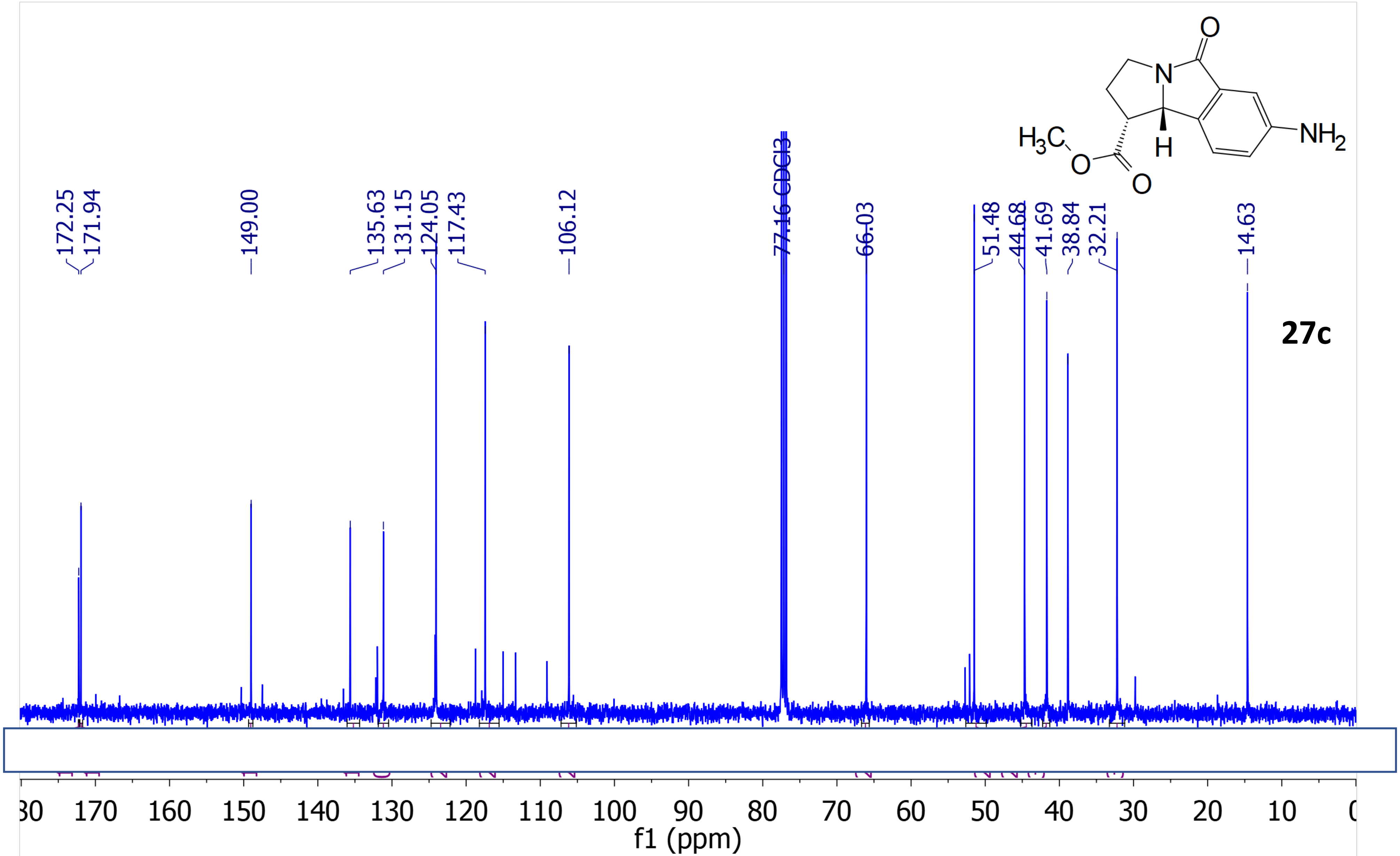

^{13}C NMR (101 MHz, $CDCl_3$) δ 172.25, 171.94, 149.00, 135.63, 131.15, 124.05, 117.43, 106.12, 77.16, 66.03, 51.48, 44.68, 41.69, 38.84, 32.21, 14.63.

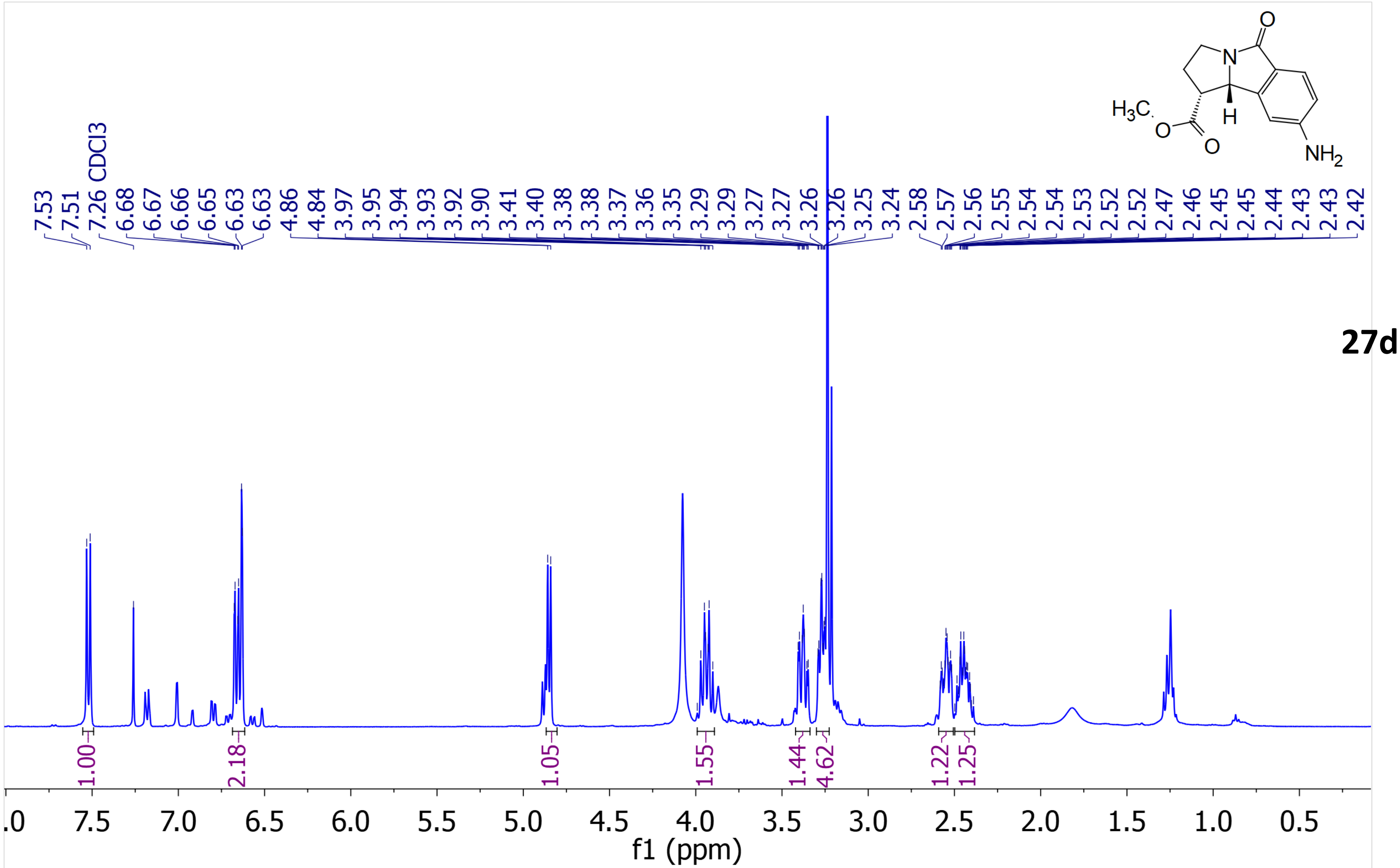

^{1}H NMR (400 MHz, Chloroform-*d*) δ 7.52 (d, *J* = 8.2 Hz, 1H), 6.69 – 6.62 (m, 2H), 4.85 (d, *J* = 7.0 Hz, 1H), 3.94 (dt, *J* = 11.0, 8.4 Hz, 1H), 3.38 (ddd, *J* = 11.6, 9.3, 3.0 Hz, 1H), 3.30 – 3.23 (m, 4H), 2.55 (ddt, *J* = 13.2, 8.3, 2.6 Hz, 1H), 2.44 (dtd, *J* = 13.5, 8.9, 7.1 Hz, 1H).

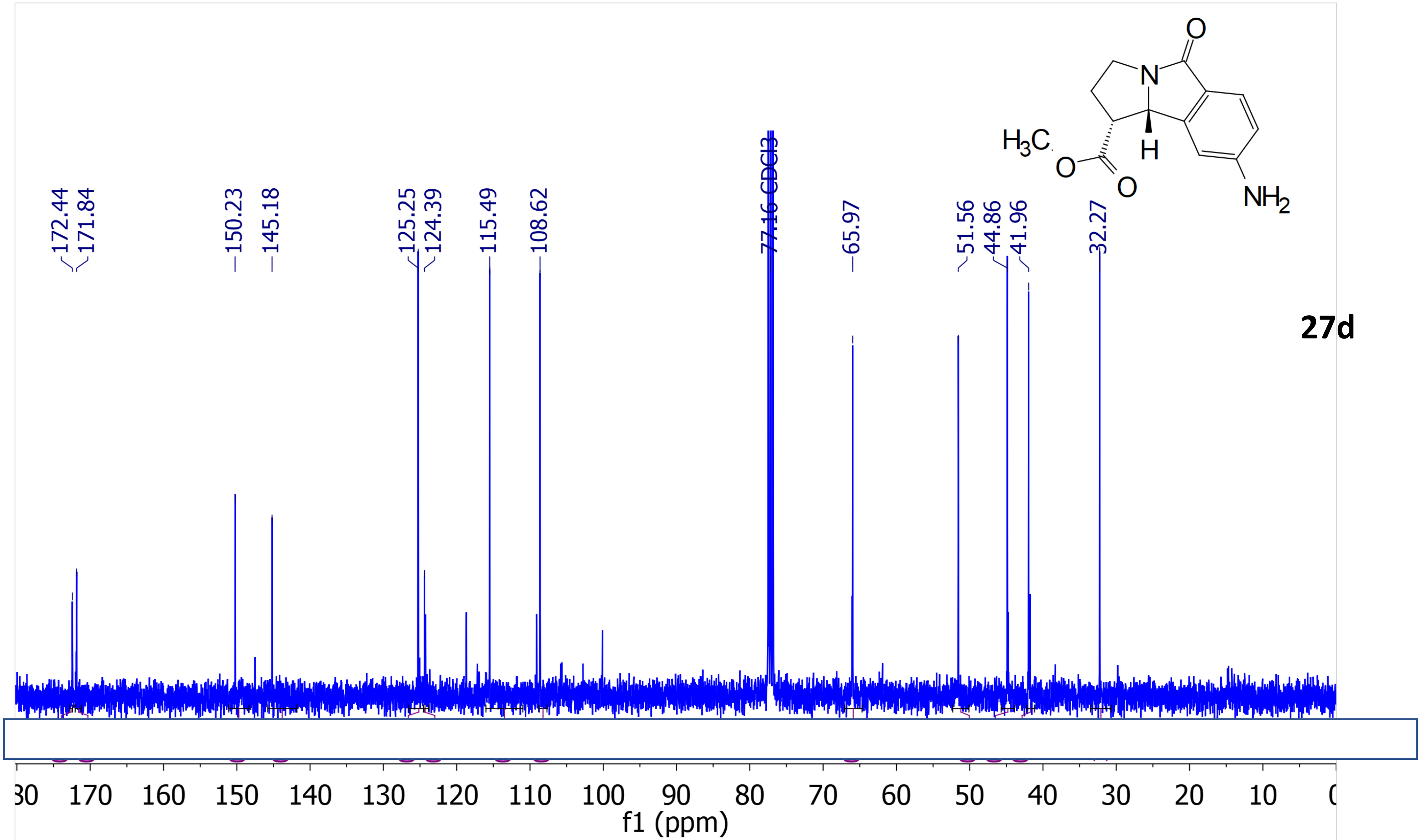

^{13}C NMR (101 MHz, $CDCl_3$) δ 172.44, 171.84, 150.23, 145.18, 125.25,124.39, 115.49, 108.62, 65.97, 51.56, 44.86, 41.96, 32.27.

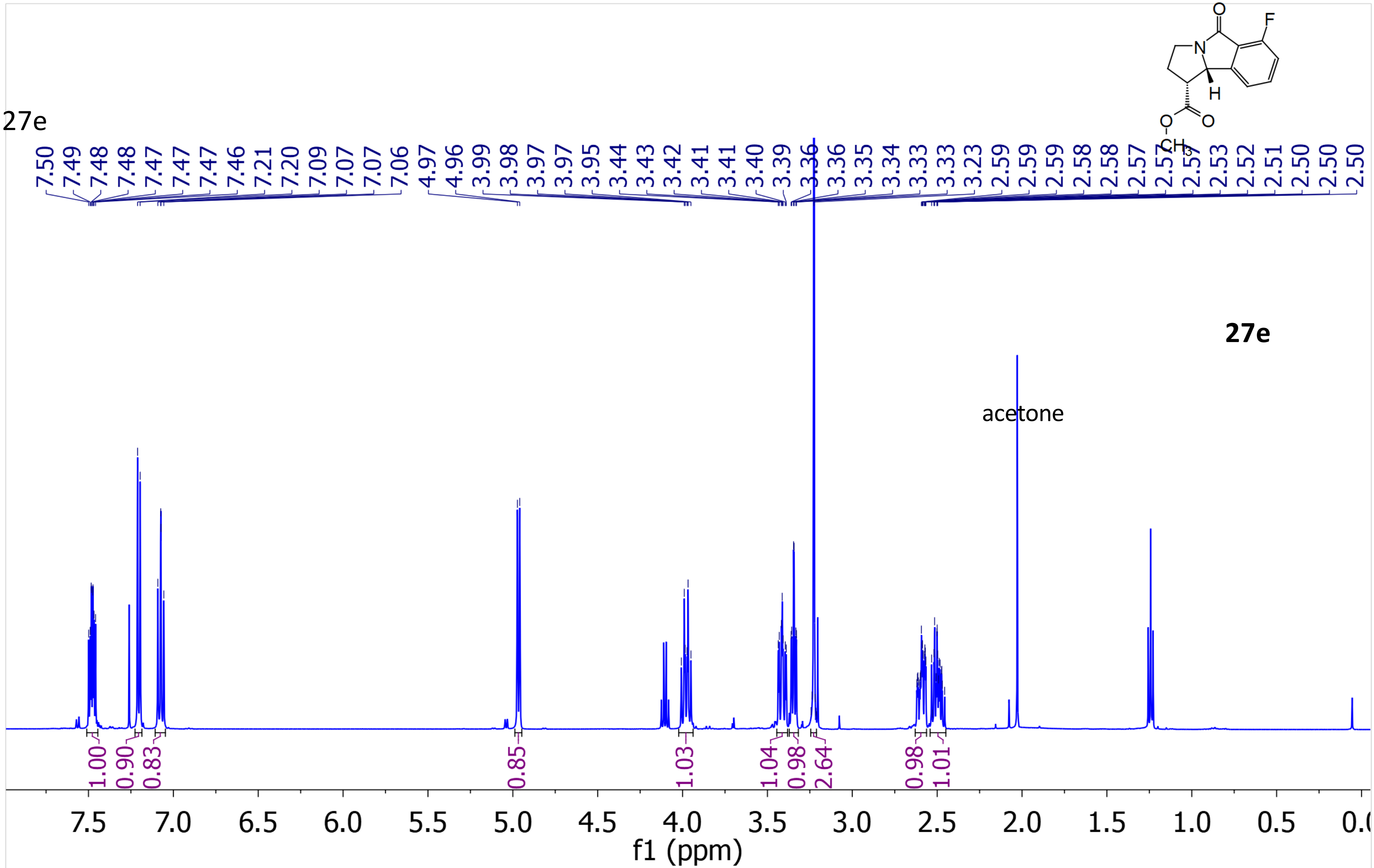

^{1}H NMR (500 MHz, Chloroform-*d*) δ 7.48 (ddd, *J* = 8.4, 7.6, 4.7 Hz, 1H), 7.20 (d, *J* = 7.5 Hz, 1H), 7.07 (t, *J* = 17.6, 8.8 Hz, 1H), 4.97 (d, *J* = 6.9 Hz, 1H), 3.98 (dt, *J* = 11.3, 8.5 Hz, 1H), 3.41 (ddd, *J* = 11.9, 9.3, 3.0 Hz, 1H), 3.34 (td, *J* = 7.0, 1.6 Hz, 1H), 3.23 (s, 3H), 2.59 (dddd, *J* = 13.2, 8.5, 3.0, 1.6 Hz, 1H), 2.54 – 2.45 (m, 1H).

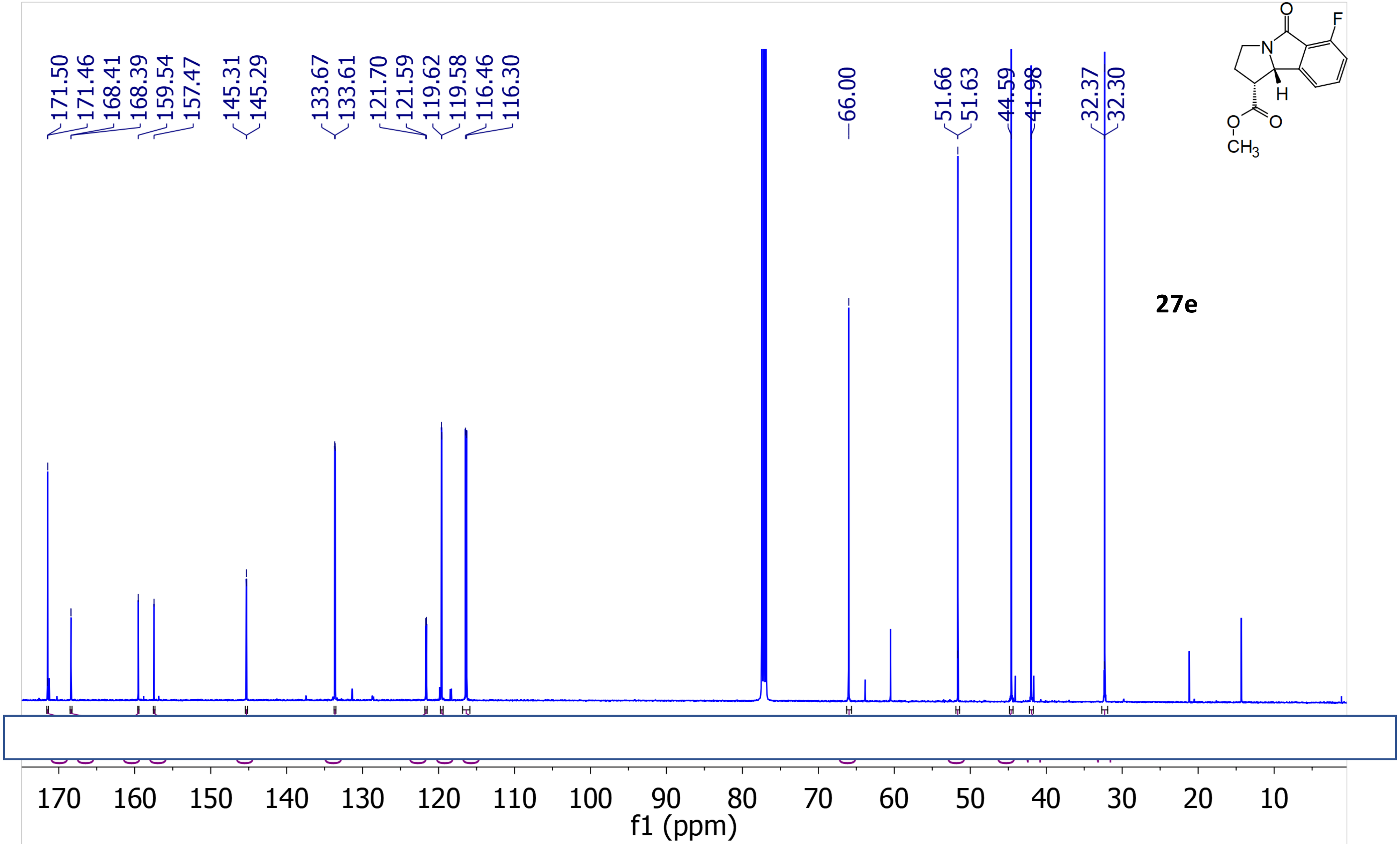

^{13}C NMR (126 MHz, $CDCl_3$) δ 171.50, 171.46, 168.41, 168.39, 159.54, 157.47, 145.31, 145.29, 133.67, 133.61, 121.70, 121.59, 119.62, 119.58, 116.46, 116.30, 66.00, 51.66, 51.63, 44.59, 41.98, 32.37, 32.30.

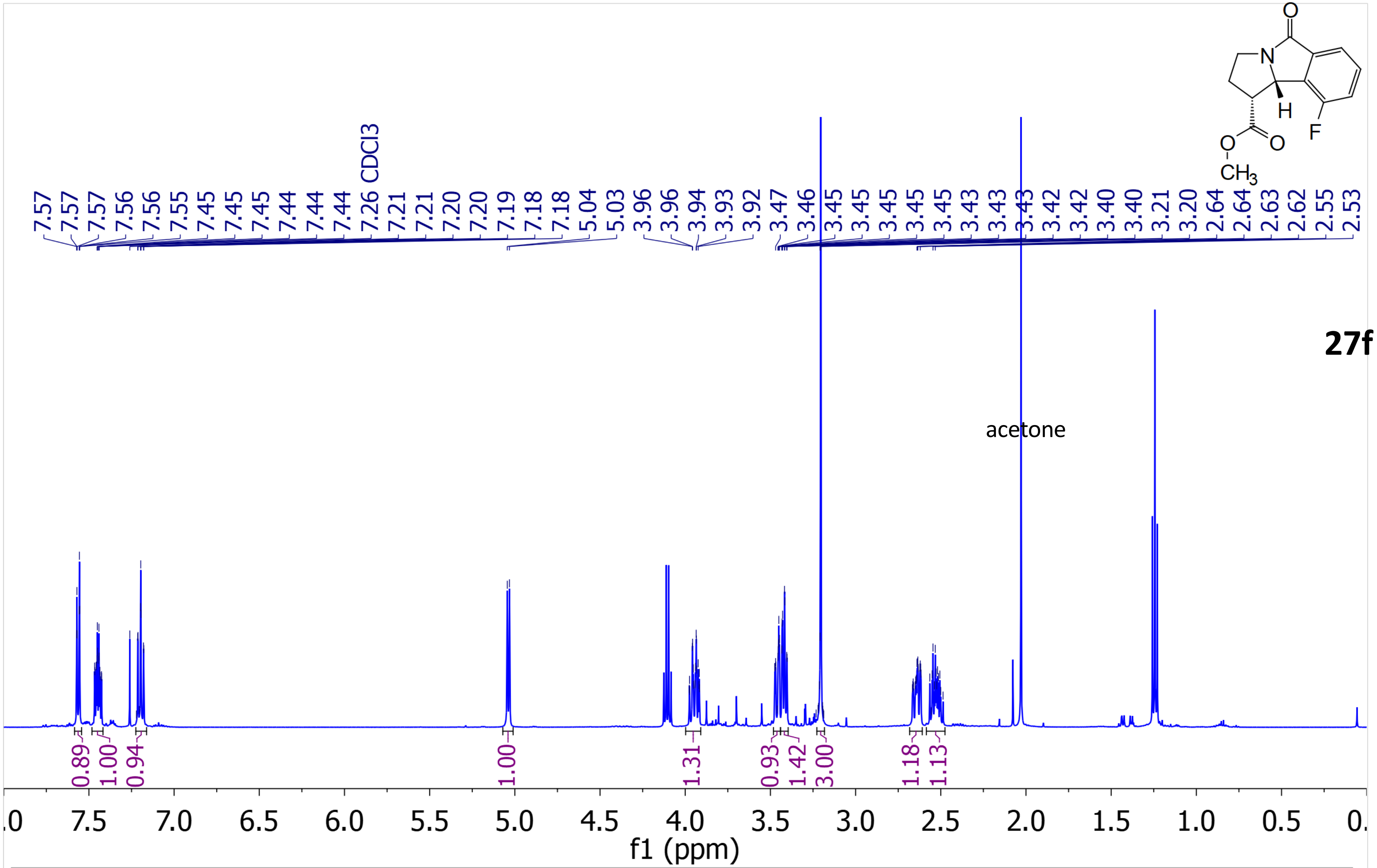

^{1}H NMR (500 MHz, Chloroform-*d*) δ 7.56 (dt, *J* = 7.5, 0.8 Hz, 1H), 7.45 (dddd, *J* = 8.1, 7.4, 4.6, 0.6 Hz, 1H), 7.20 (ddd, *J* = 8.8, 8.2, 0.8 Hz, 1H), 5.04 (d, *J* = 6.4 Hz, 1H), 4.00 – 3.91 (m, 1H), 3.48 – 3.44 (m, 1H), 3.44 – 3.40 (m, 1H), 3.20 (s, 3H), 2.64 (dddd, *J* = 13.6, 8.4, 2.4, 1.1 Hz, 1H), 2.53 (dtd, *J* = 13.6, 9.3, 6.8 Hz, 1H).
Product contains EtOAc at 13.4% by weight

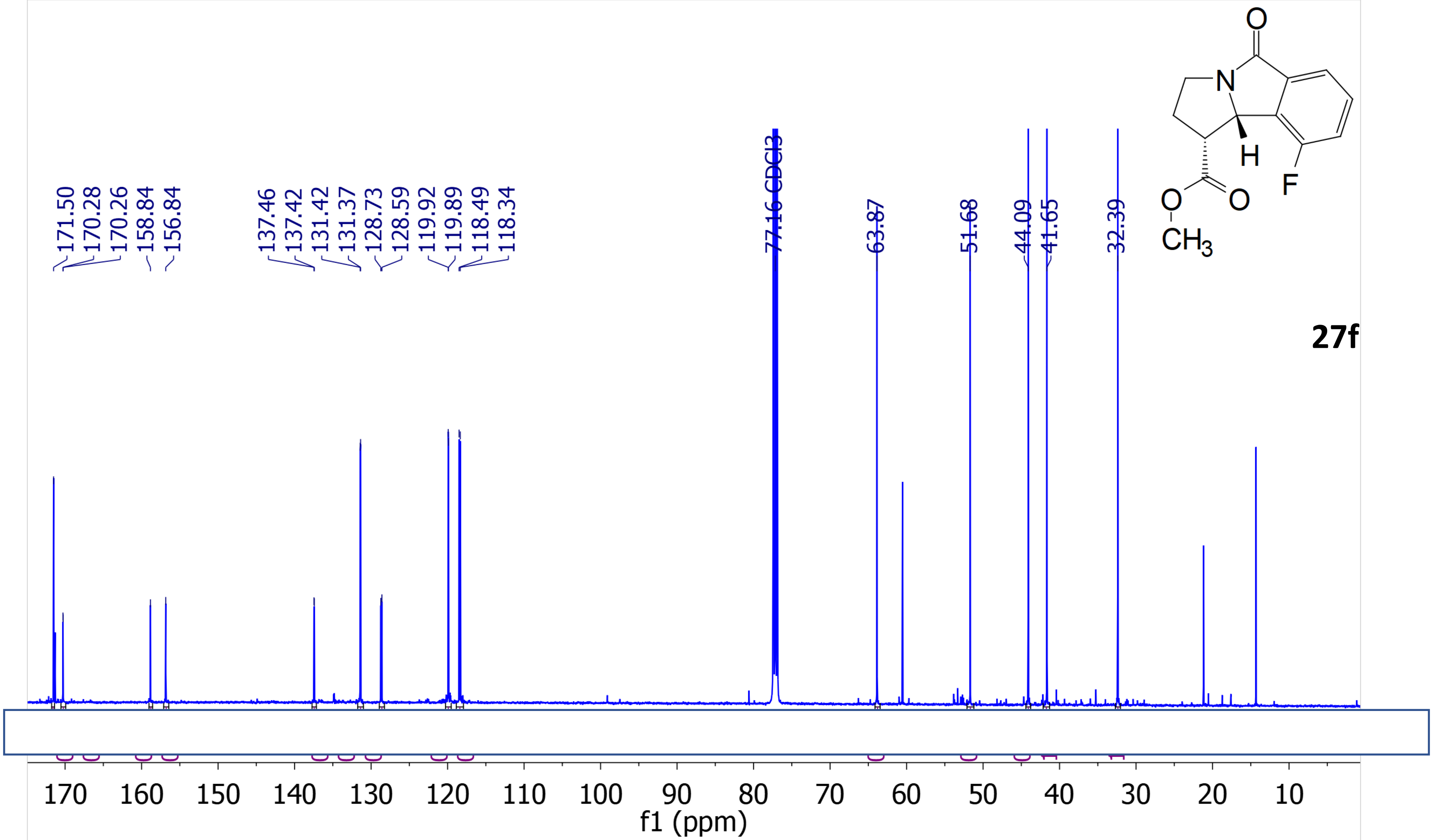

^{13}C NMR (126 MHz, $CDCl_3$) δ 171.50, 170.28, 170.26, 158.84, 156.84, 137.46, 137.42, 131.42, 131.37, 128.73, 128.59, 119.92, 119.89, 118.49, 118.34, 63.87, 51.68, 44.09, 41.65, 32.39.

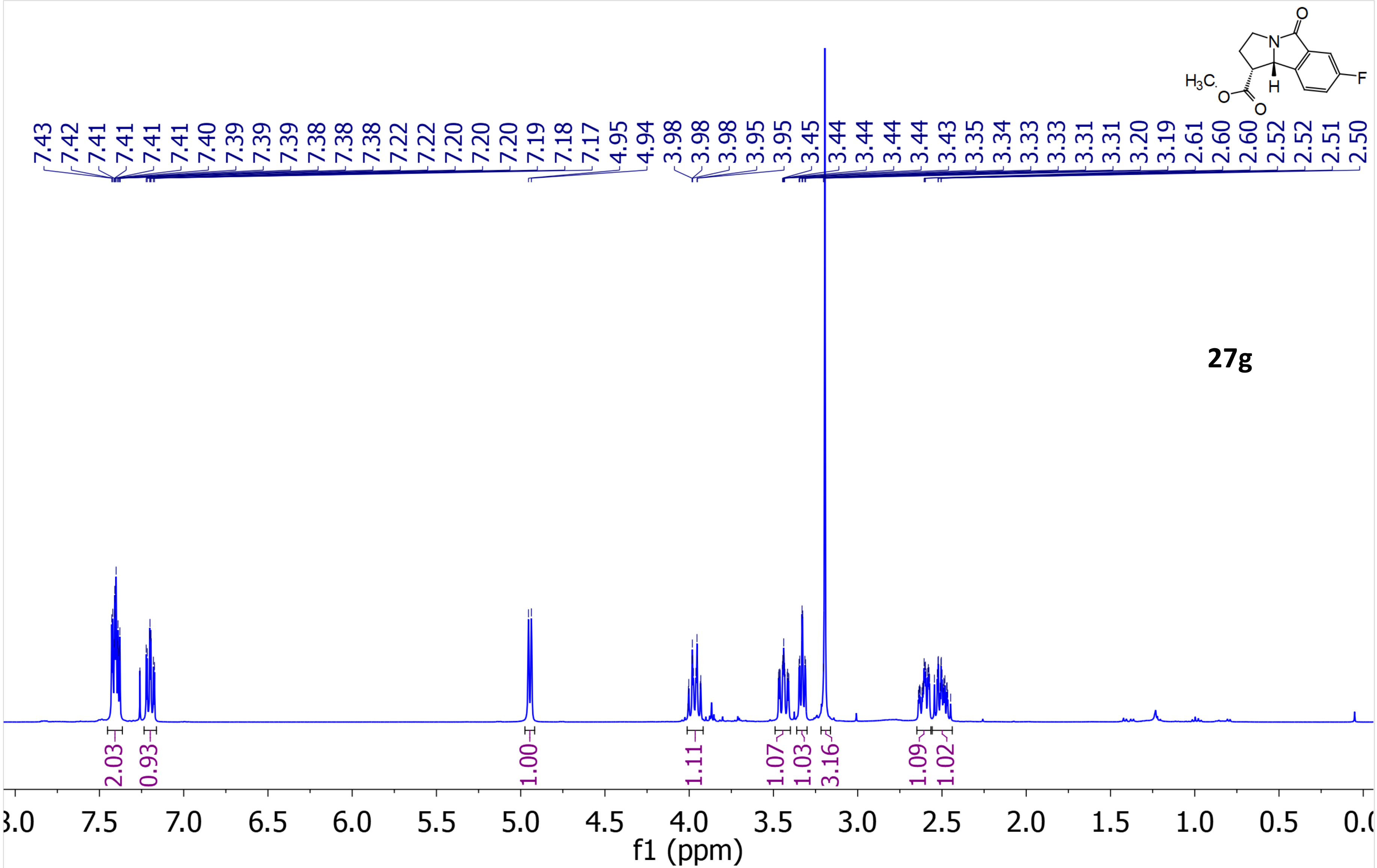

^{1}H NMR (400 MHz, Chloroform-*d*) δ 7.45 – 7.36 (m, 2H), 7.20 (ddd, *J* = 9.0, 8.3, 2.5 Hz, 1H), 4.95 (d, *J* = 6.9 Hz, 1H), 3.97 (dt, *J* = 11.4, 8.5 Hz, 1H), 3.44 (dddd, *J* = 11.3, 9.4, 2.9, 0.7 Hz, 1H), 3.33 (td, *J* = 6.9, 1.6 Hz, 1H), 3.19 (s, 3H), 2.61 (dddd, *J* = 13.0, 8.4, 2.9, 1.5 Hz, 1H), 2.50 (dddd, *J* = 13.6, 9.4, 8.6, 6.9 Hz, 1H).

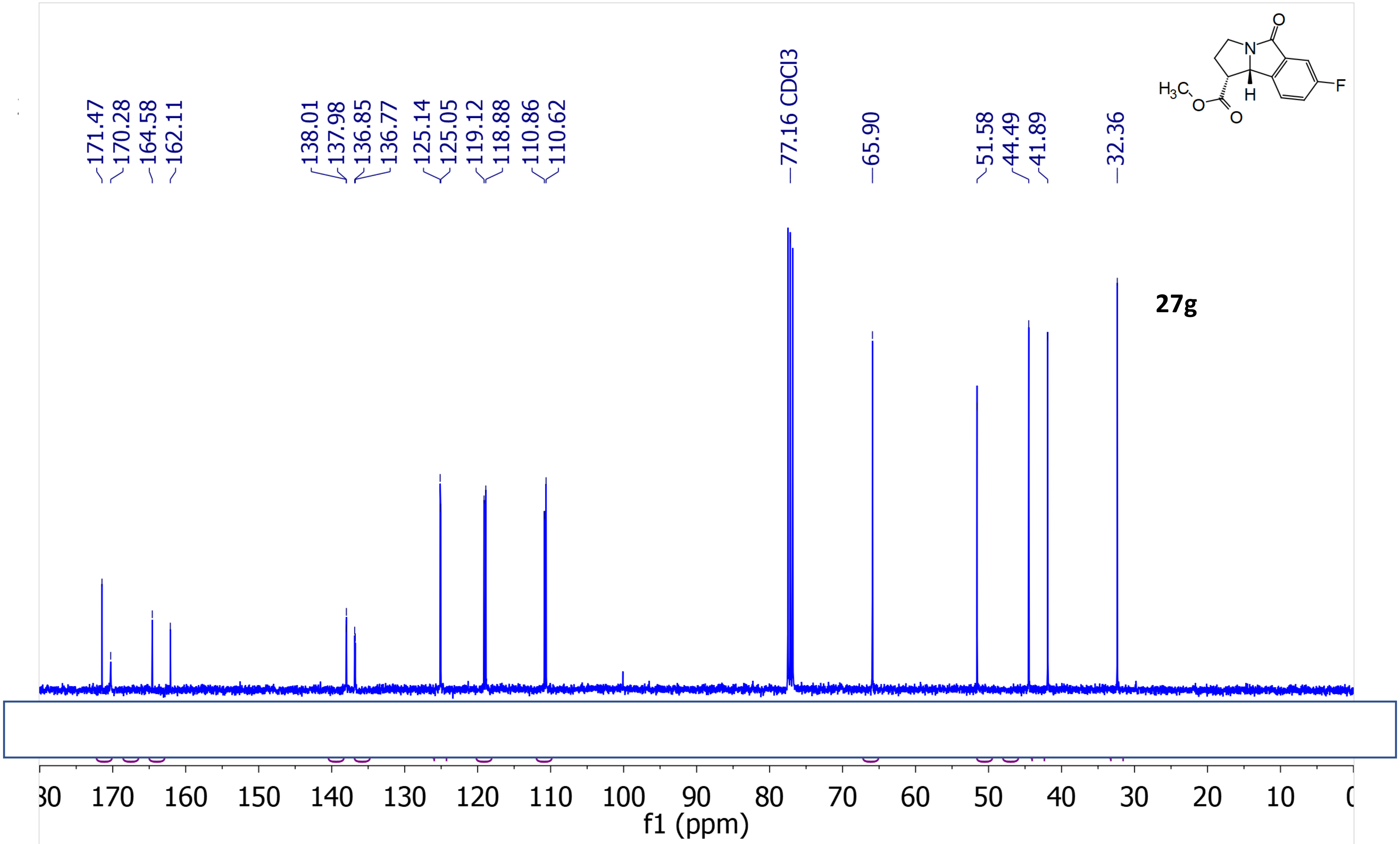

^{13}C NMR (101 MHz, $CDCl_3$) δ 171.47, 170.28, 164.58, 162.11, 138.01, 137.98, 136.85, 136.77, 125.14, 125.05, 119.12, 118.88, 110.86, 110.62, 77.16, 65.90, 51.58, 44.49, 41.89, 32.36.

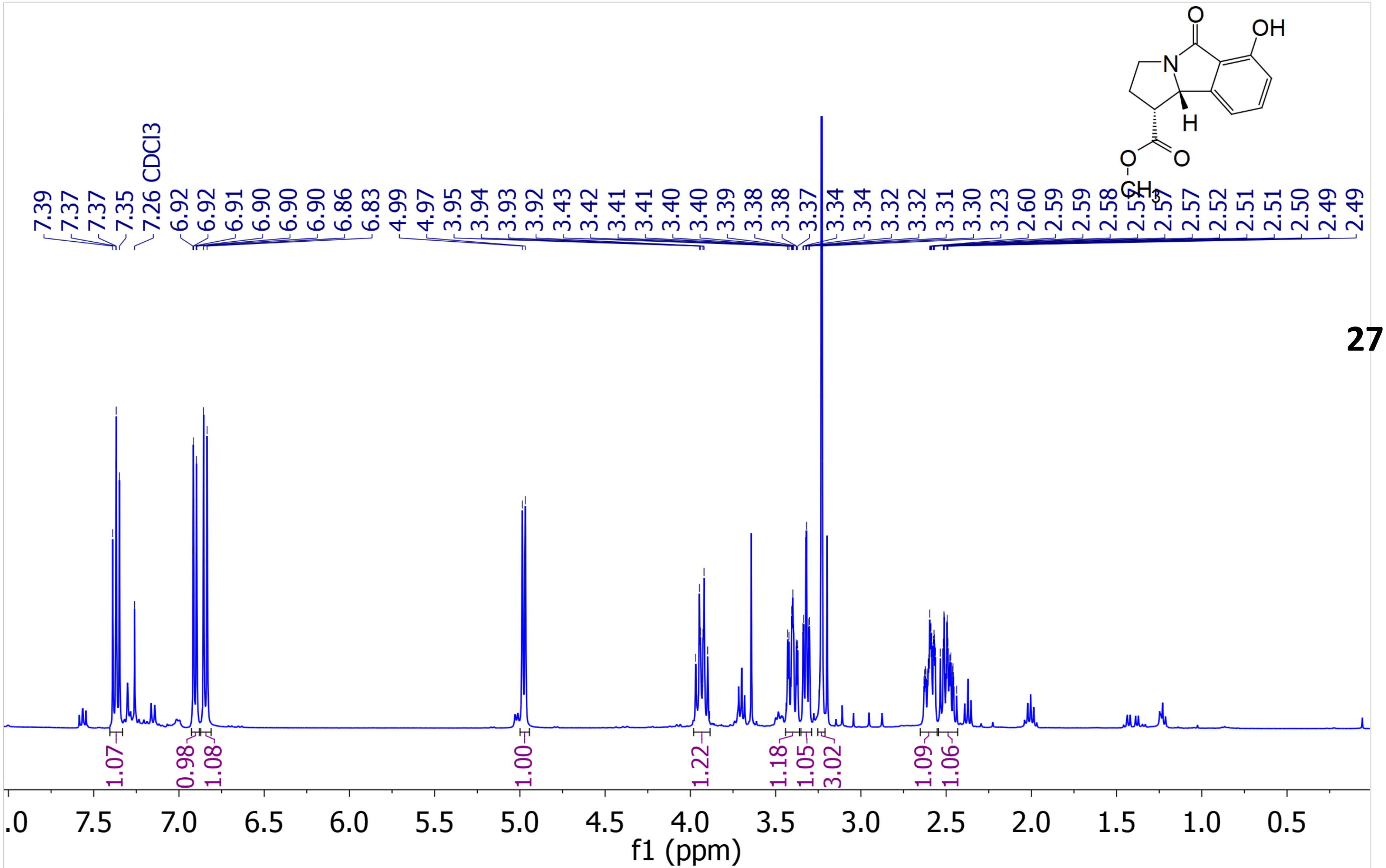

27h

^{1}H NMR (400 MHz, Chloroform-*d*) δ 7.37 (dd, *J* = 8.3, 7.4 Hz, 1H), 6.91 (dt, *J* = 7.4, 0.8 Hz, 1H), 6.85 (d, *J* = 8.2 Hz, 1H), 4.98 (d, *J* = 6.9 Hz, 1H), 3.93 (dt, *J* = 11.1, 8.5 Hz, 1H), 3.44 – 3.36 (m, 1H), 3.32 (td, *J* = 6.9, 1.6 Hz, 1H), 3.23 (s, 3H), 2.60 (dddd, *J* = 13.2, 8.4, 3.0, 1.6 Hz, 1H), 2.49 (dddd, *J* = 13.6, 9.4, 8.6, 6.9 Hz, 1H).

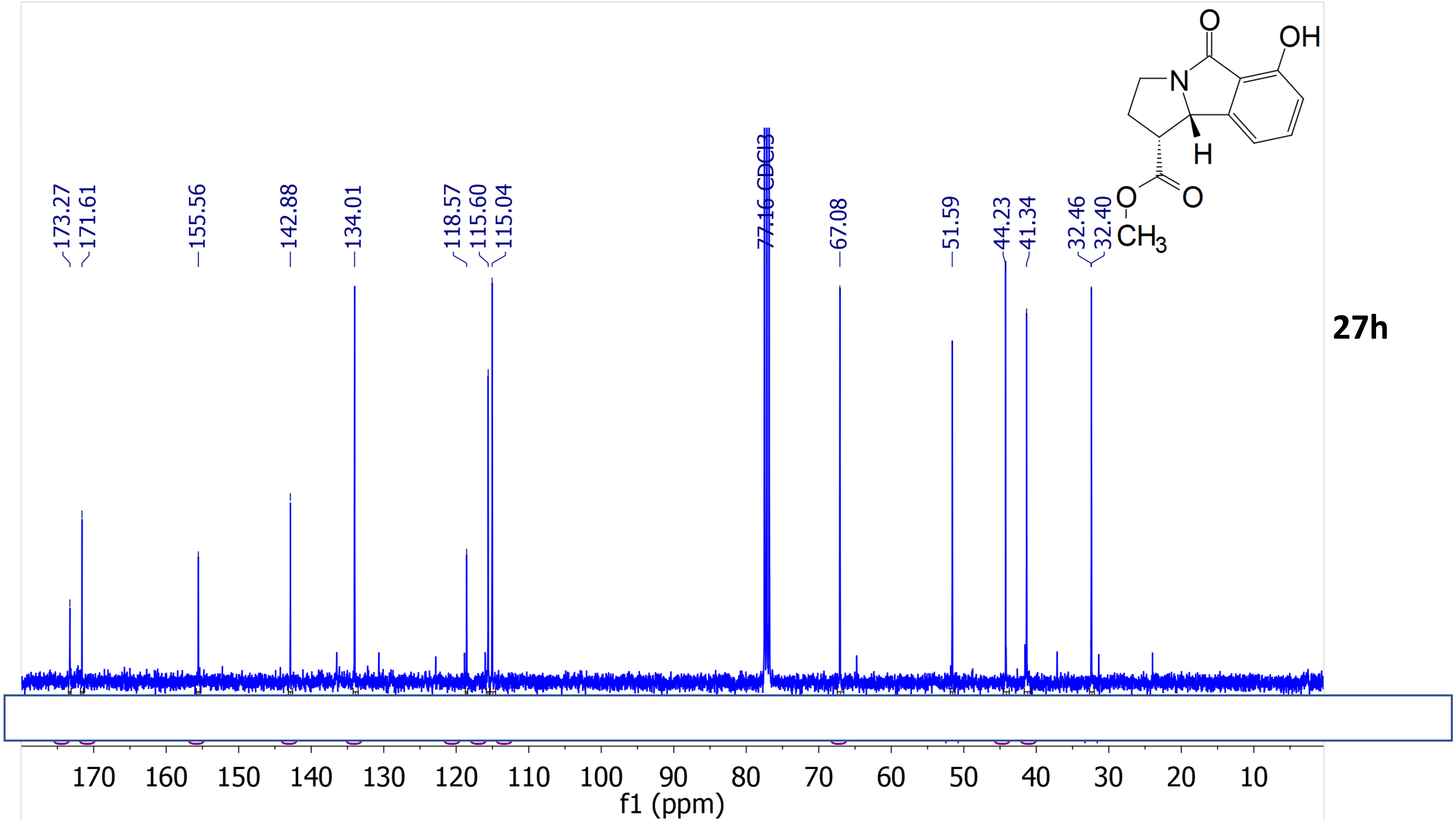

27h

^{13}C NMR (101 MHz, $CDCl_3$) δ 173.27, 171.61, 155.56, 142.88, 134.01, 118.57, 115.60, 115.04, 67.08, 51.59, 44.23, 41.34, 32.46, 32.40.

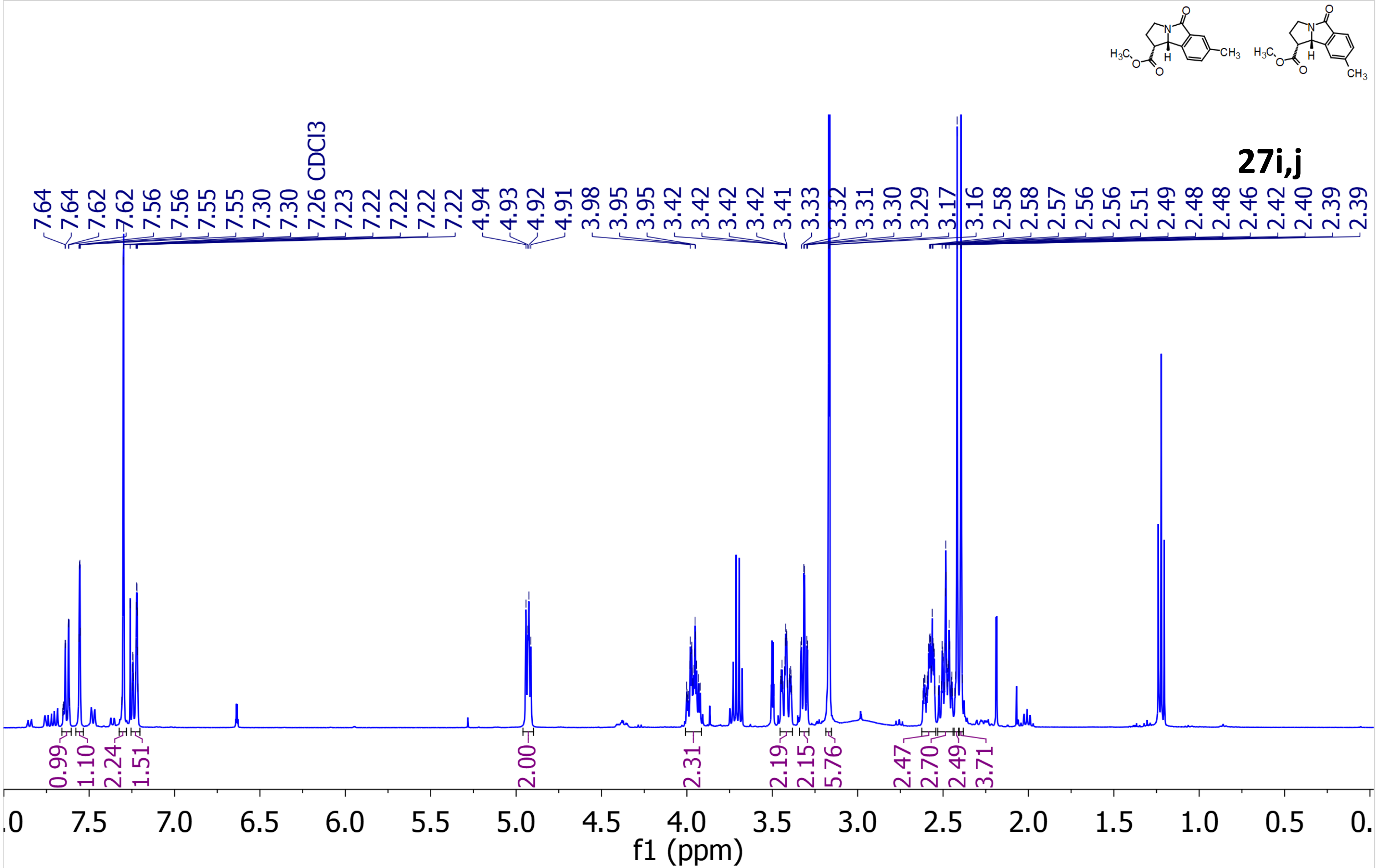

^1H NMR (400 MHz, Chloroform-*d*) δ 7.66 – 7.61 (m, 1H), 7.56 (q, *J* = 1.0 Hz, 1H), 7.30 (d, *J* = 1.2 Hz, 2H), 7.26 – 7.20 (m, 2H), 4.93 (dd, *J* = 6.9, 4.4 Hz, 2H), 4.01 – 3.91 (m, 2H), 3.45 – 3.38 (m, 2H), 3.31 (td, *J* = 7.0, 1.6 Hz, 2H), 3.17 (d, *J* = 2.0 Hz, 6H), 2.58 (dddd, *J* = 13.2, 8.4, 3.0, 1.6 Hz, 2H), 2.53 – 2.44 (m, 2H), 2.42 (s, 3H), 2.39 (d, *J* = 0.7 Hz, 3H).

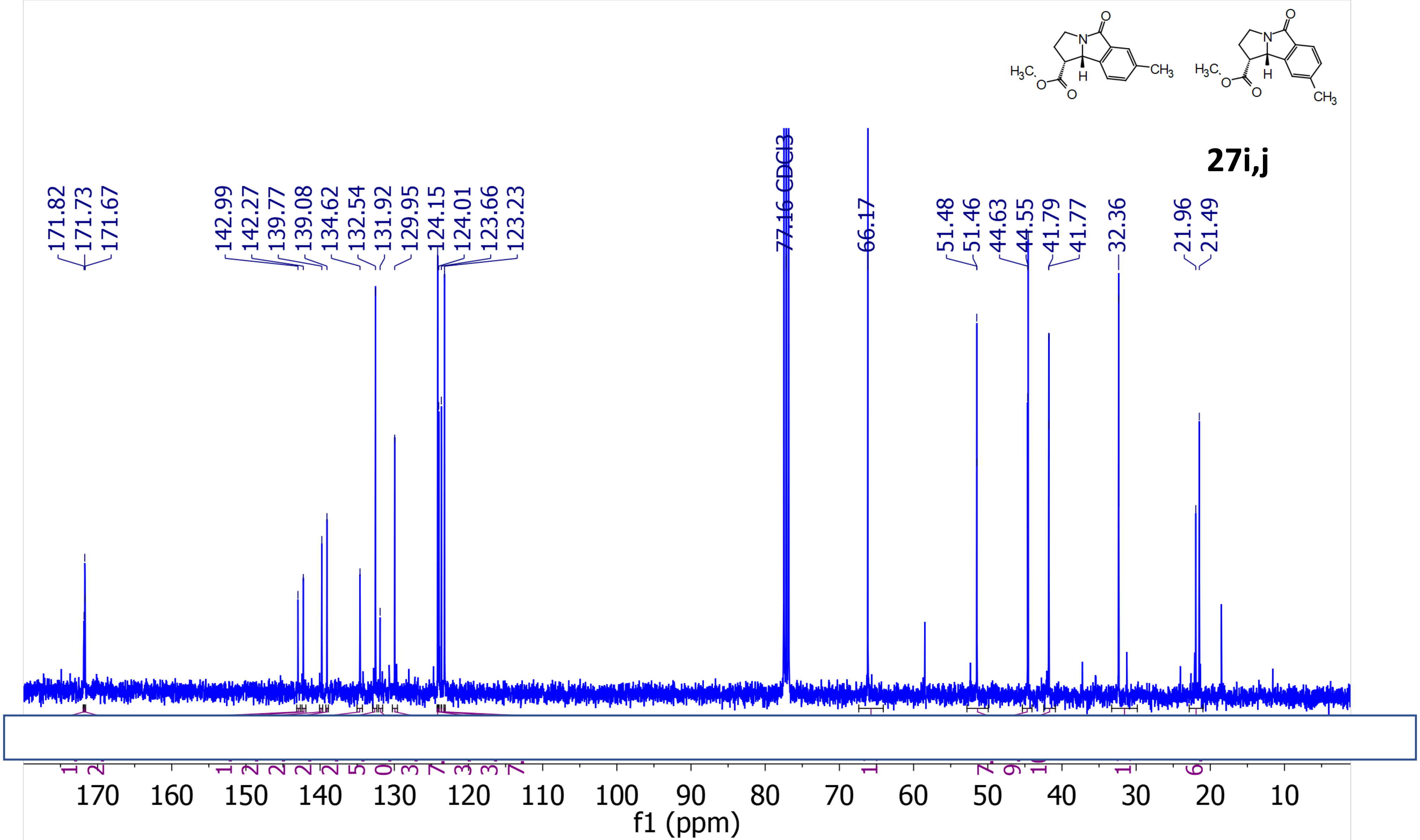

^{13}C NMR (101 MHz, $CDCl_3$) δ 171.82, 171.73, 171.67, 142.99, 142.27, 139.77, 139.08, 134.62, 132.54, 131.92, 129.95, 124.15, 124.01, 123.66, 123.23, 77.16, 66.17, 51.48, 51.46, 44.63, 44.55, 41.79, 41.77, 32.36, 21.96, 21.49.

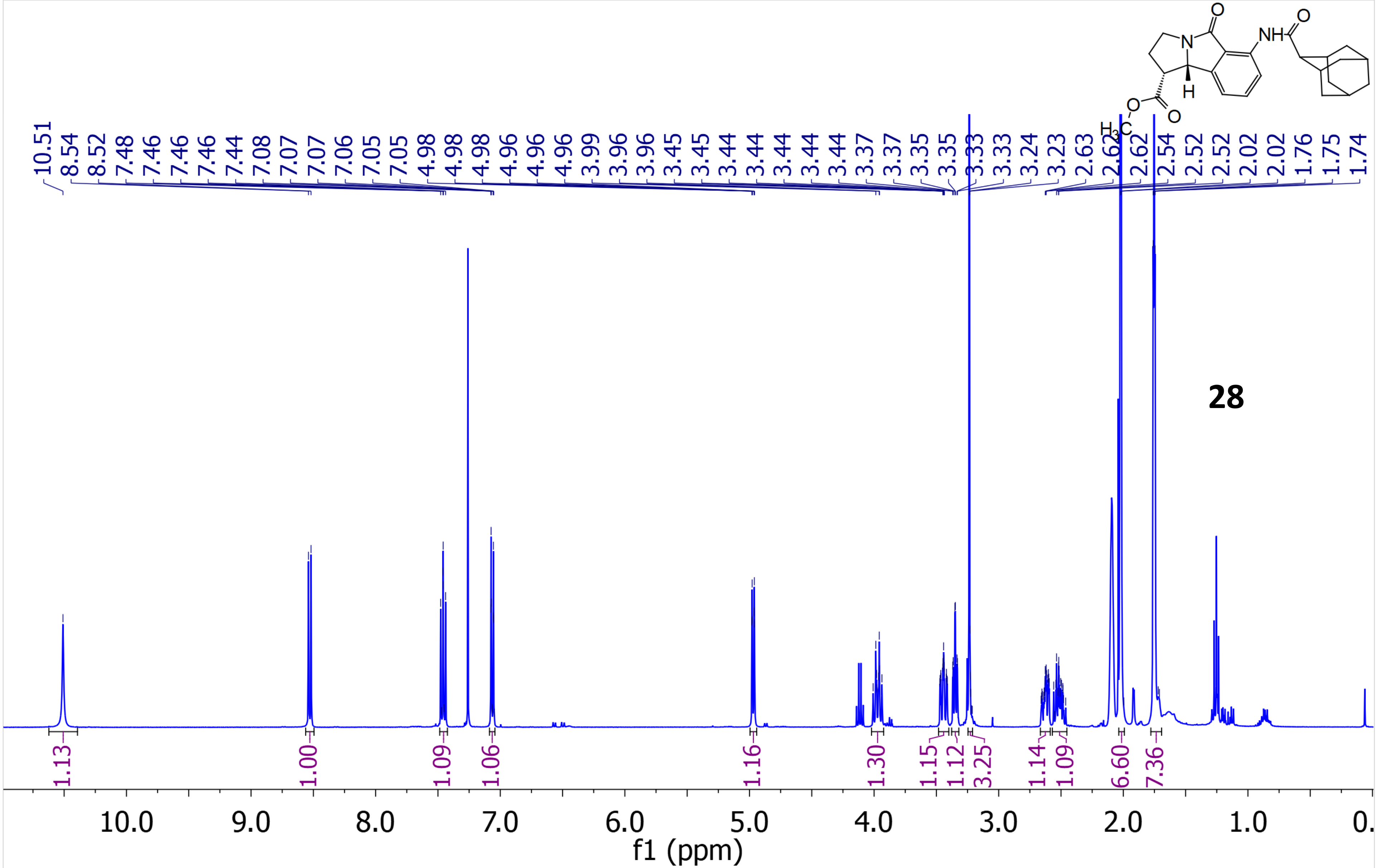

^{1}H NMR (400 MHz, Chloroform-*d*) δ 10.51 (s, 1H), 8.53 (d, *J* = 8.3 Hz, 1H), 7.49 – 7.42 (m, 1H), 7.06 (dt, *J* = 7.5, 0.8 Hz, 1H), 4.97 (dt, *J* = 7.0, 0.8 Hz, 1H), 3.97 (dt, *J* = 11.1, 8.5 Hz, 1H), 3.44 (dddd, *J* = 11.1, 9.4, 2.8, 0.7 Hz, 1H), 3.35 (td, *J* = 6.8, 1.5 Hz, 1H), 3.24 (s, 3H), 2.63 (dddd, *J* = 13.6, 8.4, 2.8, 1.5 Hz, 1H), 2.57 – 2.45 (m, 1H), 2.02 (d, *J* = 3.1 Hz, 7H), 1.75 (t, *J* = 3.2 Hz, 7H).

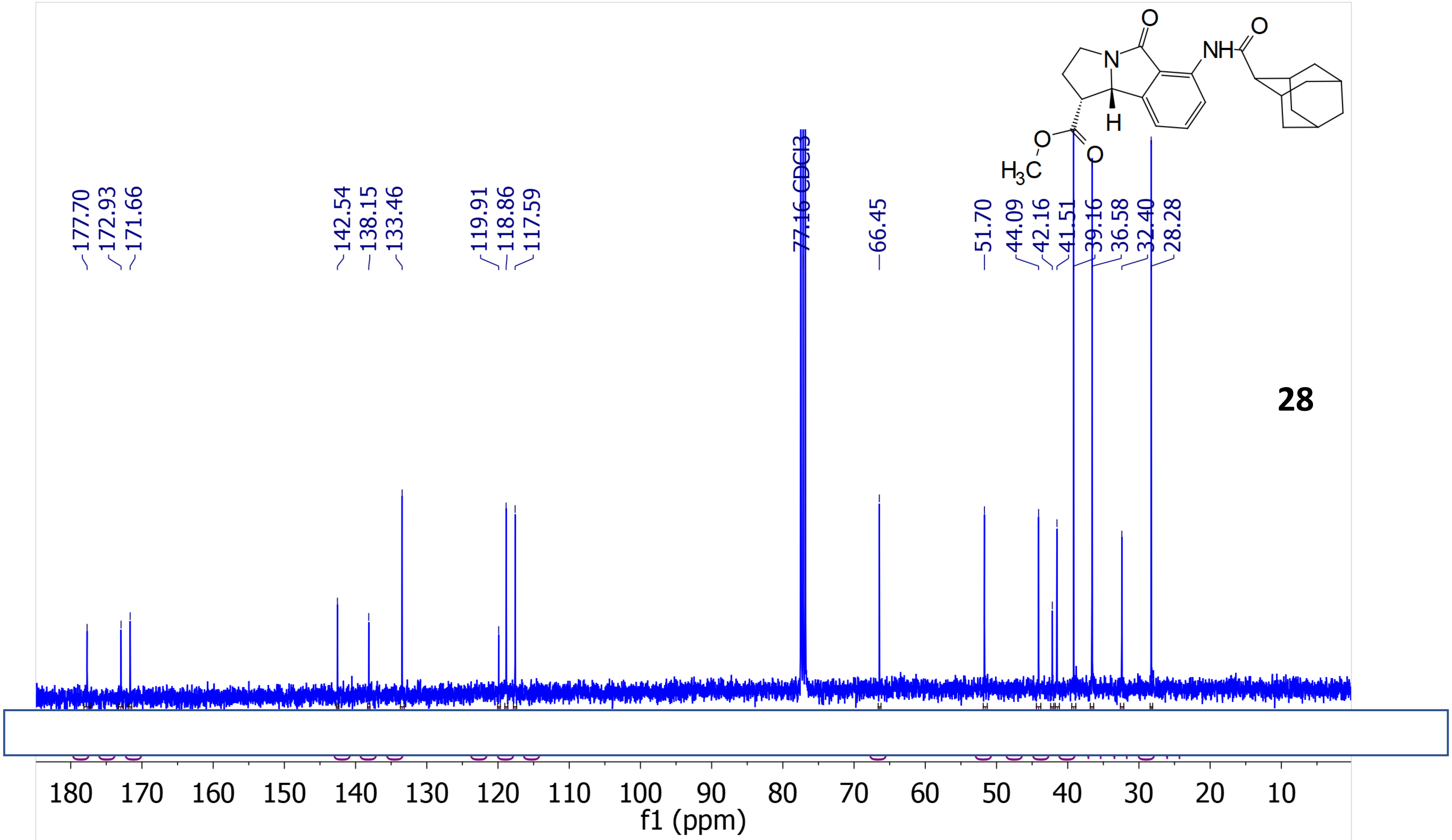

^{13}C NMR (101 MHz, $CDCl_3$) δ 177.70, 172.93, 171.66, 142.54, 138.15, 133.46, 119.91, 118.86, 117.59, 66.45, 51.70, 44.09, 42.16, 41.51, 39.16, 36.58, 32.40, 28.28.

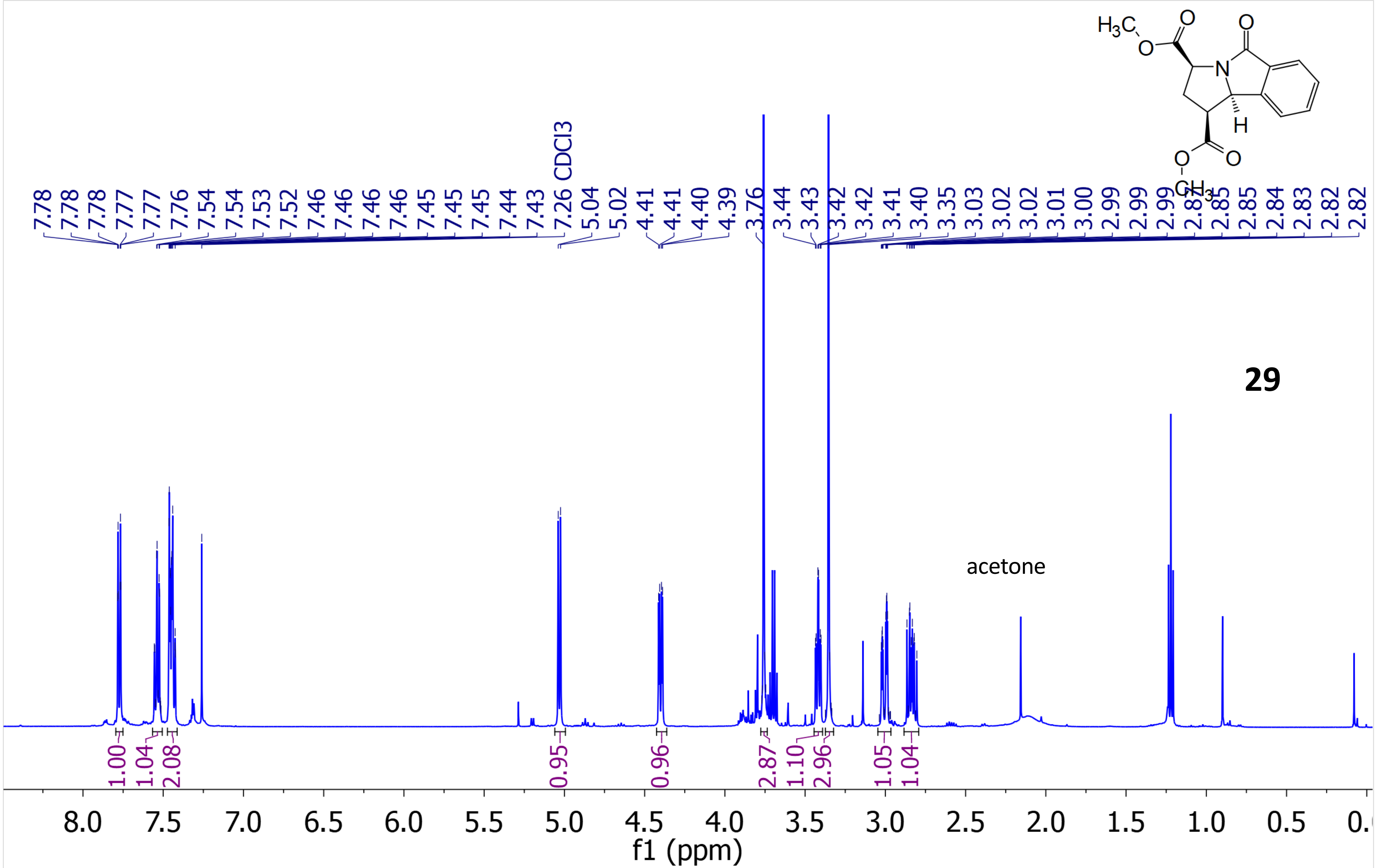

^{1}H NMR (500 MHz, Chloroform-*d*) δ 7.77 (d, *J* = 7.6, 1.1 Hz, 1H), 7.54 (td, *J* = 7.5, 1.3 Hz, 1H), 7.47 – 7.41 (m, 2H), 5.03 (d, *J* = 7.2 Hz, 1H), 4.40 (dd, *J* = 9.0, 3.2 Hz, 1H), 3.76 (s, 3H), 3.42 (td, *J* = 7.6, 2.2 Hz, 1H), 3.35 (s, 3H), 3.01 (ddd, *J* = 14.0, 3.2, 2.2 Hz, 1H), 2.83 (ddd, *J* = 13.9, 9.0, 7.8 Hz, 1H).

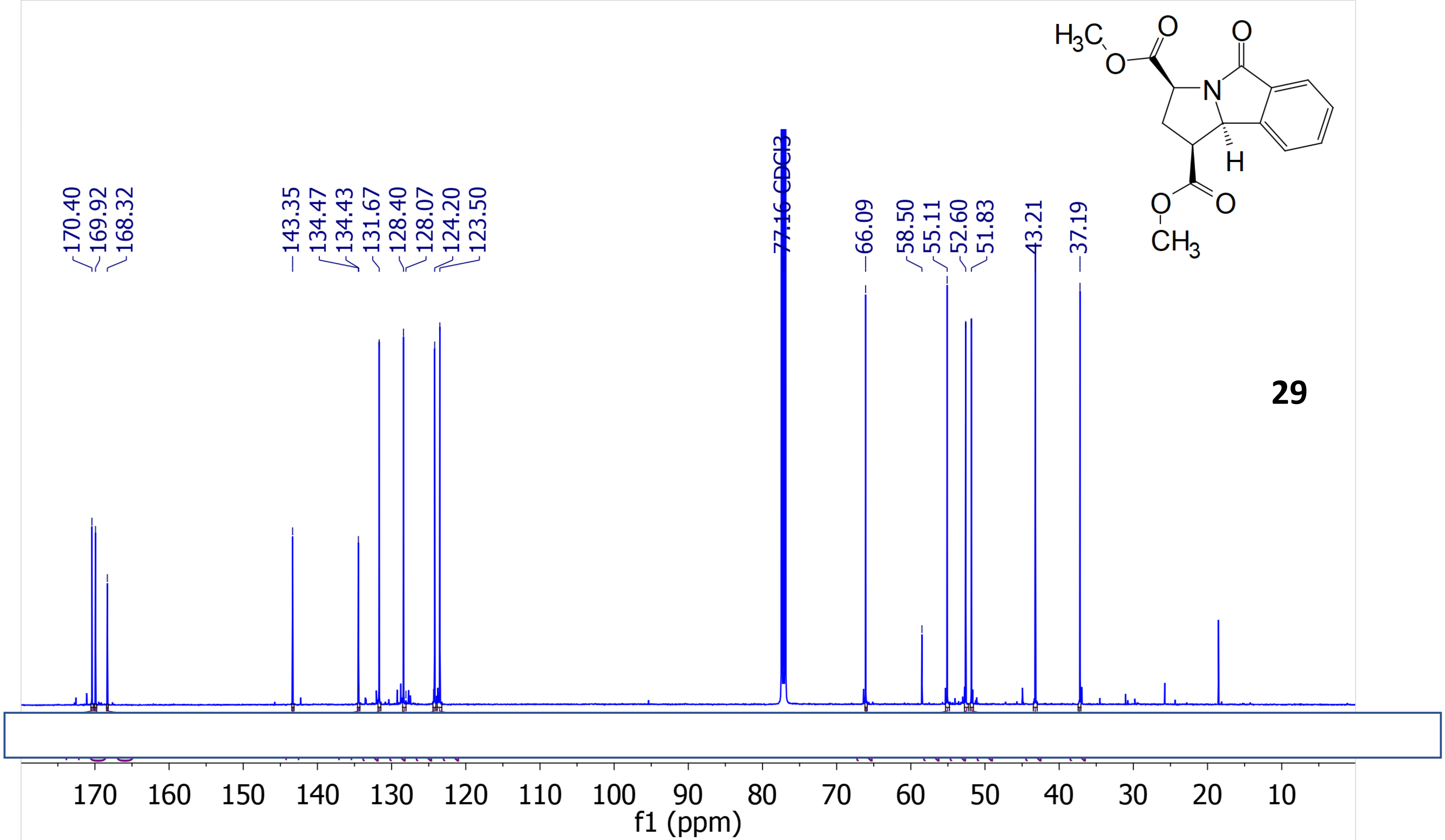

29

^{13}C NMR (126 MHz, $CDCl_3$) δ 170.40, 169.92, 168.32, 143.35, 134.47, 134.43, 131.67, 128.40, 128.07, 124.20, 123.50, 66.09, 58.50, 55.11, 52.60, 51.83, 43.21, 37.19.

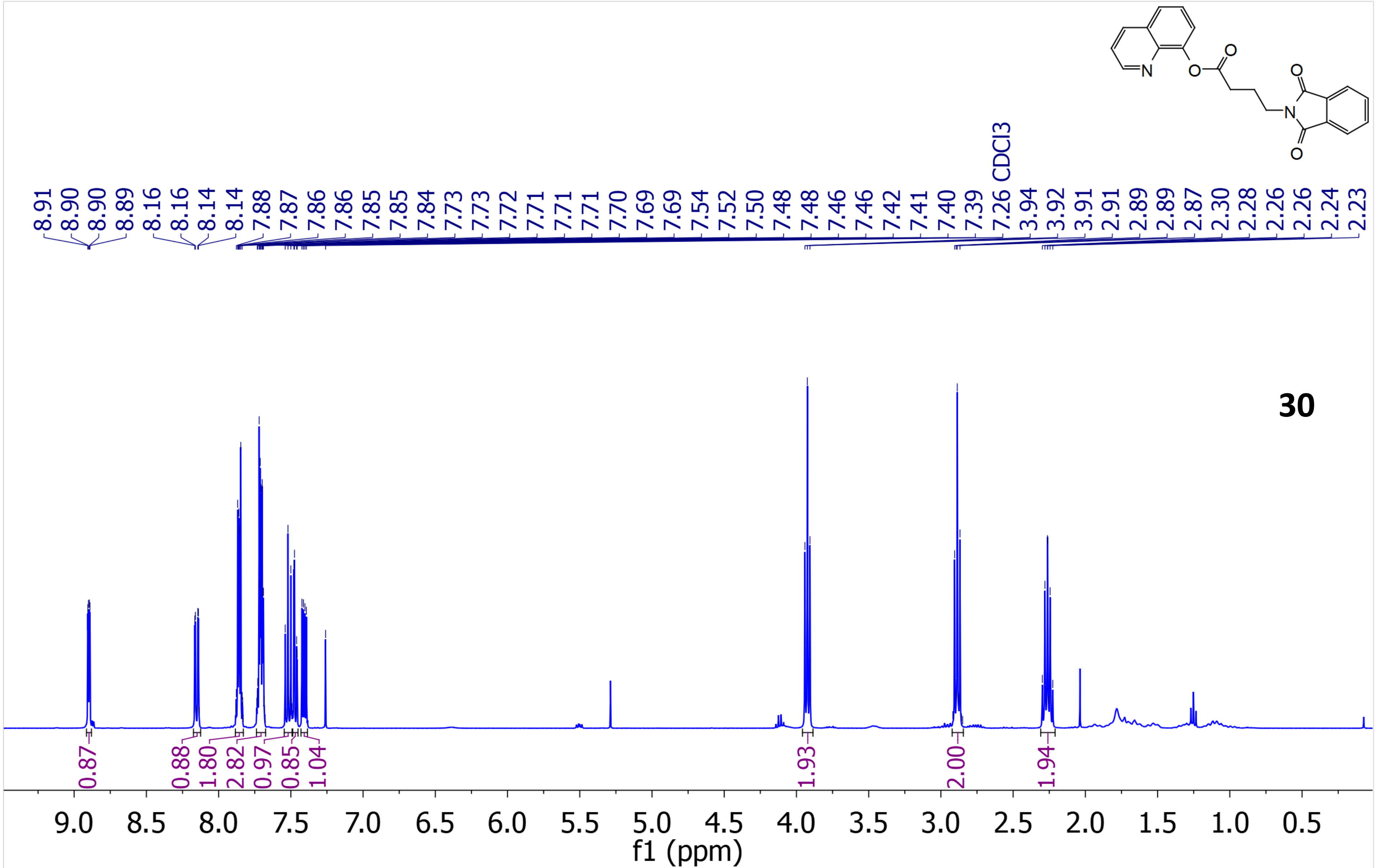

^{1}H NMR (400 MHz, Chloroform-*d*) δ 8.90 (dd, *J* = 4.2, 1.7 Hz, 1H), 8.15 (dd, *J* = 8.3, 1.7 Hz, 1H), 7.88 – 7.83 (m, 2H), 7.74 – 7.67 (m, 3H), 7.52 (t, *J* = 7.8 Hz, 1H), 7.47 (dd, *J* = 7.5, 1.6 Hz, 1H), 7.41 (dd, *J* = 8.3, 4.2 Hz, 1H), 3.92 (t, *J* = 6.9 Hz, 2H), 2.89 (t, *J* = 7.5 Hz, 2H), 2.27 (p, 2H).

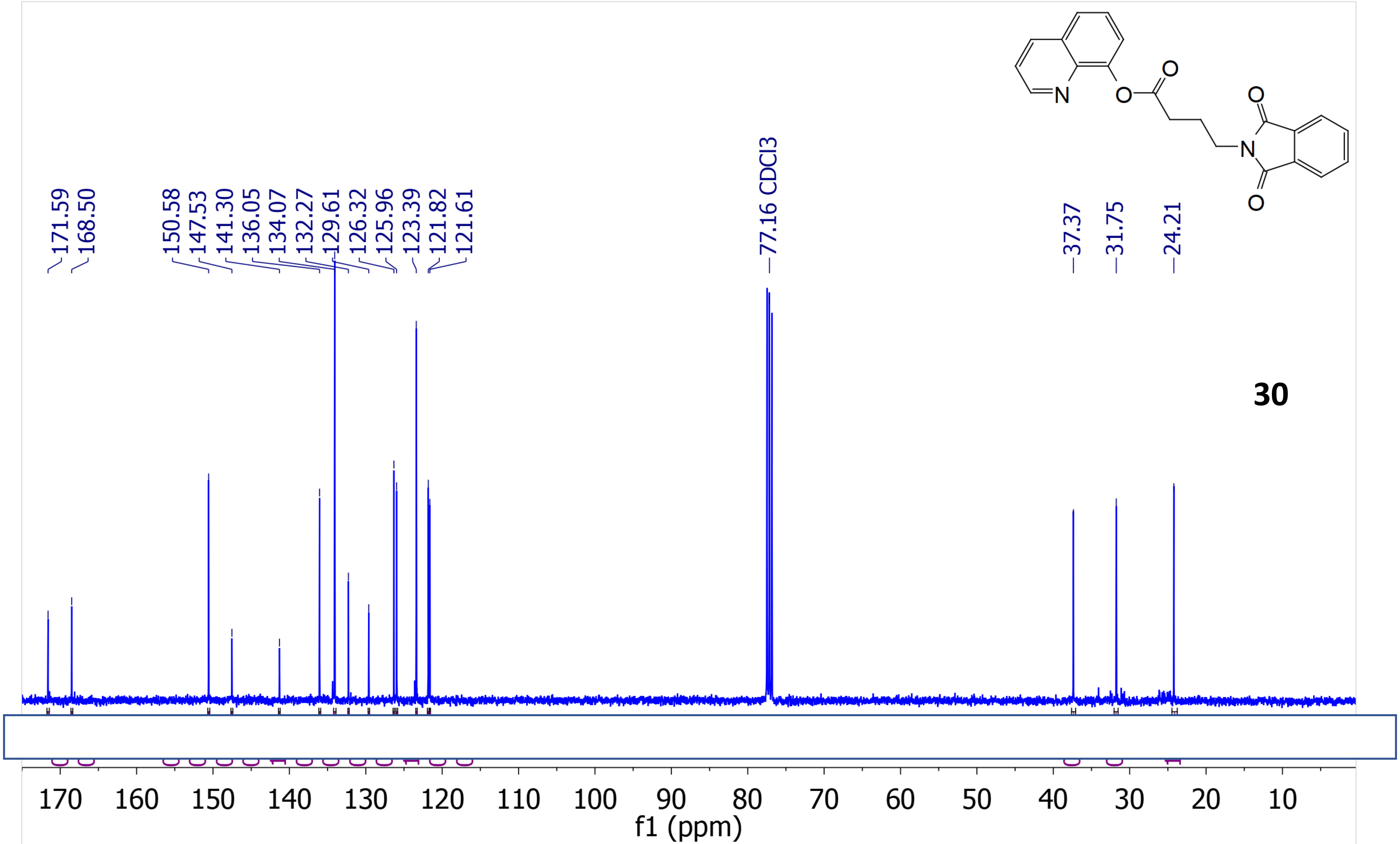

^{13}C NMR (101 MHz, Chloroform-*d*) δ 171.59, 168.50, 150.58, 147.53, 141.30, 136.05, 134.07, 132.27, 129.61, 126.32, 125.96, 123.39, 121.82, 121.61, 37.37, 31.75, 24.21.

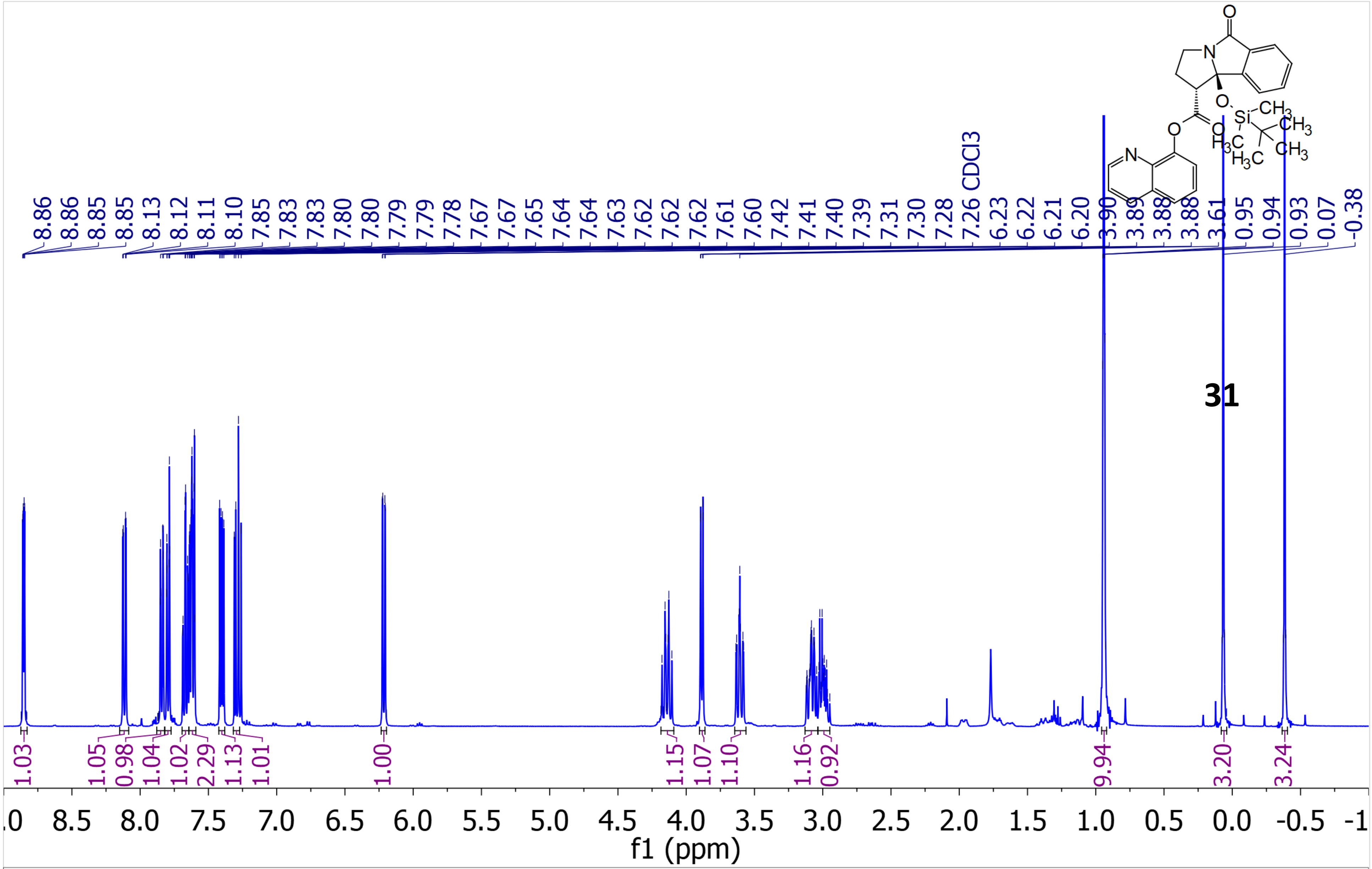

^{1}H NMR (400 MHz, Chloroform-*d*) δ 8.85 (dd, *J* = 4.2, 1.7 Hz, 1H), 8.12 (dd, *J* = 8.3, 1.7 Hz, 1H), 7.88 – 7.82 (m, 1H), 7.80 (dt, *J* = 7.5, 1.0 Hz, 1H), 7.67 (td, *J* = 7.4, 1.4 Hz, 1H), 7.62 (ddd, *J* = 8.4, 5.6, 1.4 Hz, 2H), 7.40 (dd, *J* = 8.3, 4.2 Hz, 1H), 7.32 – 7.27 (m, 1H), 6.22 (dd, *J* = 7.5, 1.3 Hz, 1H), 4.14 (dt, *J* = 11.0, 8.9 Hz, 1H), 3.89 (dd, *J* = 6.7, 0.9 Hz, 1H), 3.61 (ddd, *J* = 11.3, 9.3, 2.1 Hz, 1H), 3.13 – 3.04 (m, 1H), 3.03 – 2.95 (m, 1H), 0.94 (s, 9H), 0.07 (s, 3H), -0.38 (s, 3H).

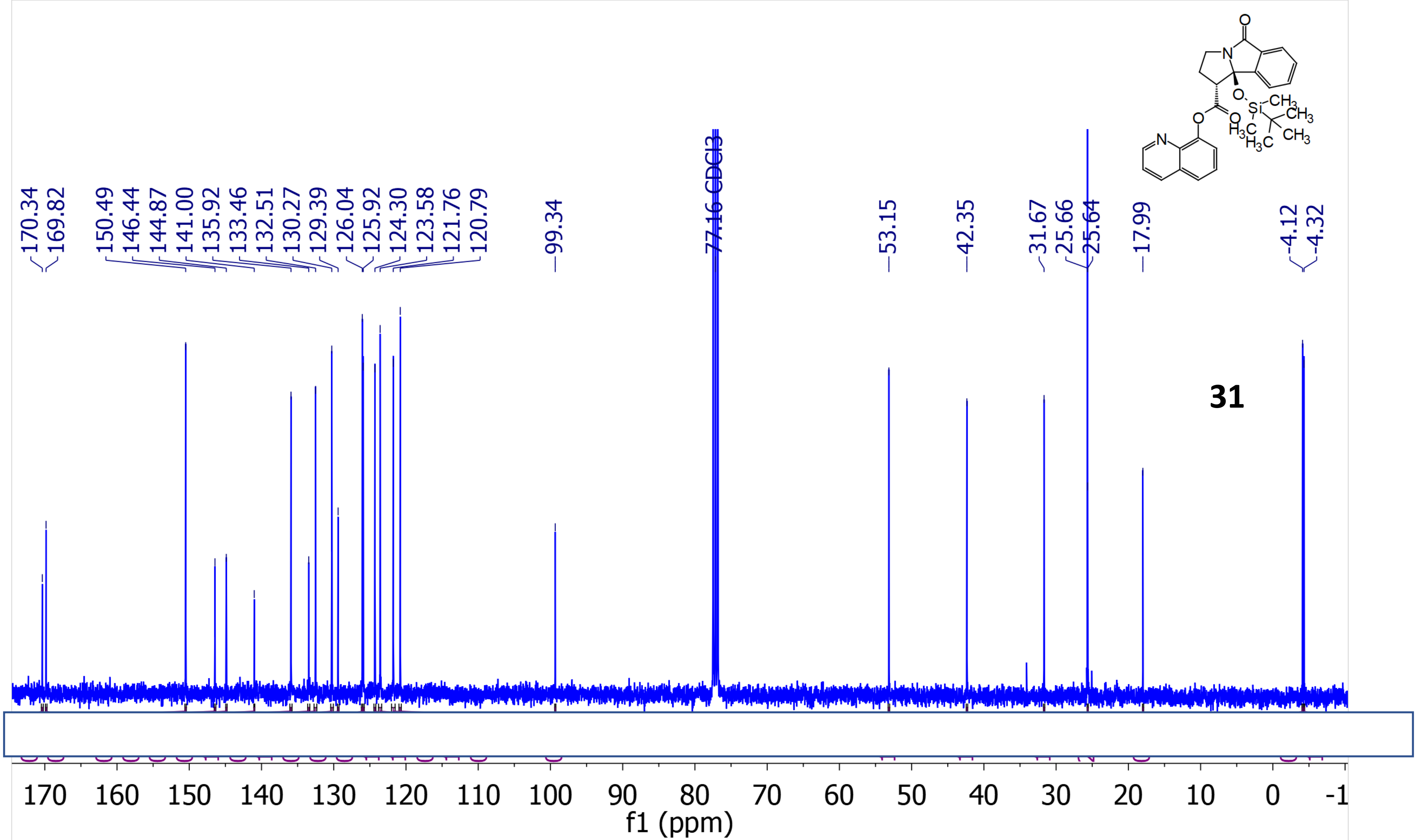

^{13}C NMR (101 MHz, Chloroform-*d*) δ 170.34, 169.82, 150.49, 146.44, 144.87, 141.00, 135.92, 133.46, 132.51, 130.27, 129.39, 126.04, 125.92, 124.30, 123.58, 121.76, 120.79, 99.34, 53.15, 42.35, 31.67, 25.66, 25.64, 17.99, -4.12, -4.32

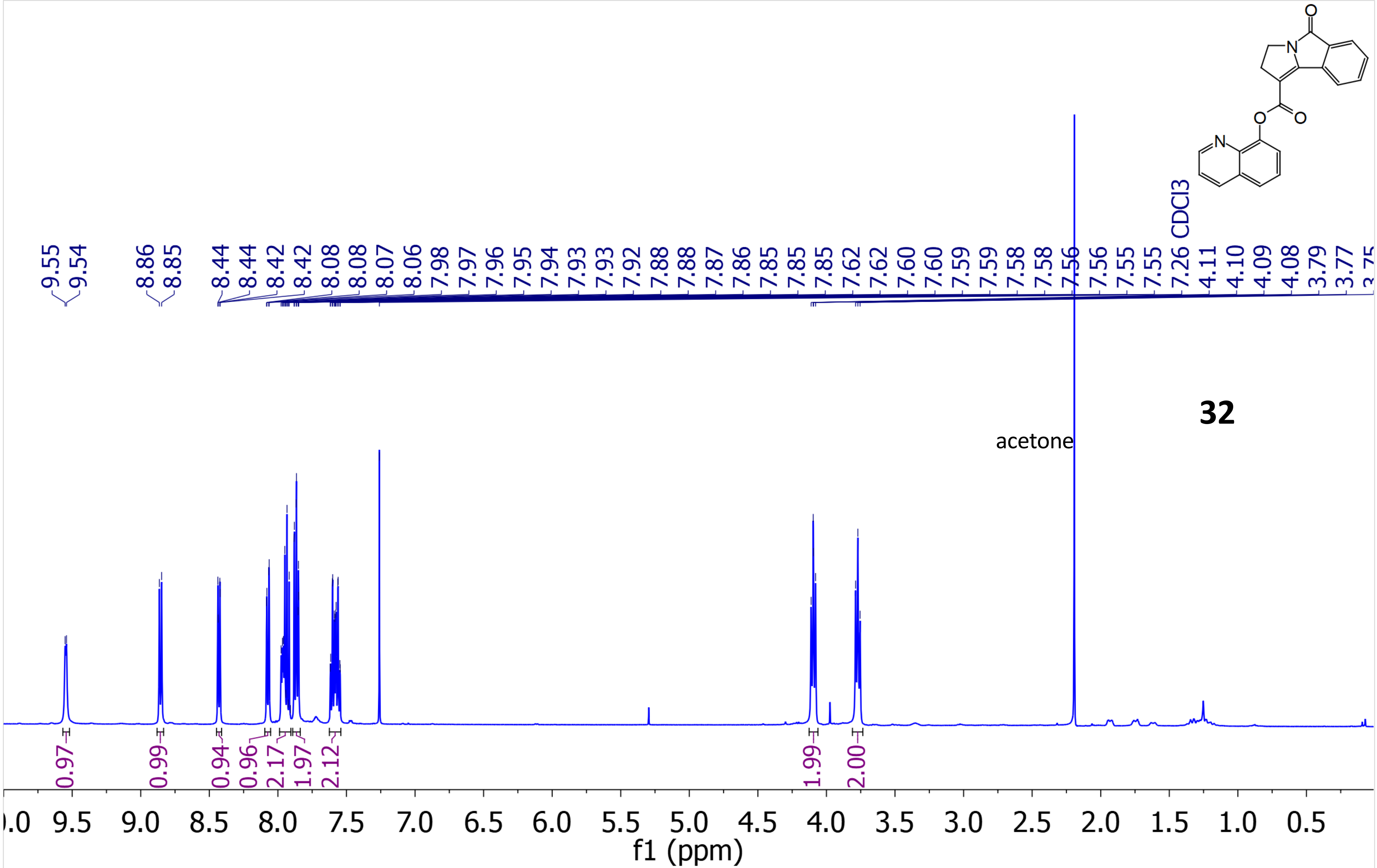

32

^{1}H NMR (500 MHz, Chloroform-*d*) δ 9.55 (d, *J* = 5.0 Hz, 1H), 8.86 (d, *J* = 8.3 Hz, 1H), 8.43 (d, *J* = 7.7 Hz, 1H), 8.07 (dd, *J* = 8.2, 1.3 Hz, 1H), 7.99 – 7.91 (m, 2H), 7.87 (t, *J* = 7.9, 1.1 Hz, 2H), 7.58 (dtd, *J* = 20.0, 7.5, 1.2 Hz, 2H), 4.10 (t, *J* = 16.5, 8.0 Hz, 2H), 3.77 (t, *J* = 16.0, 8.3 Hz, 2H).

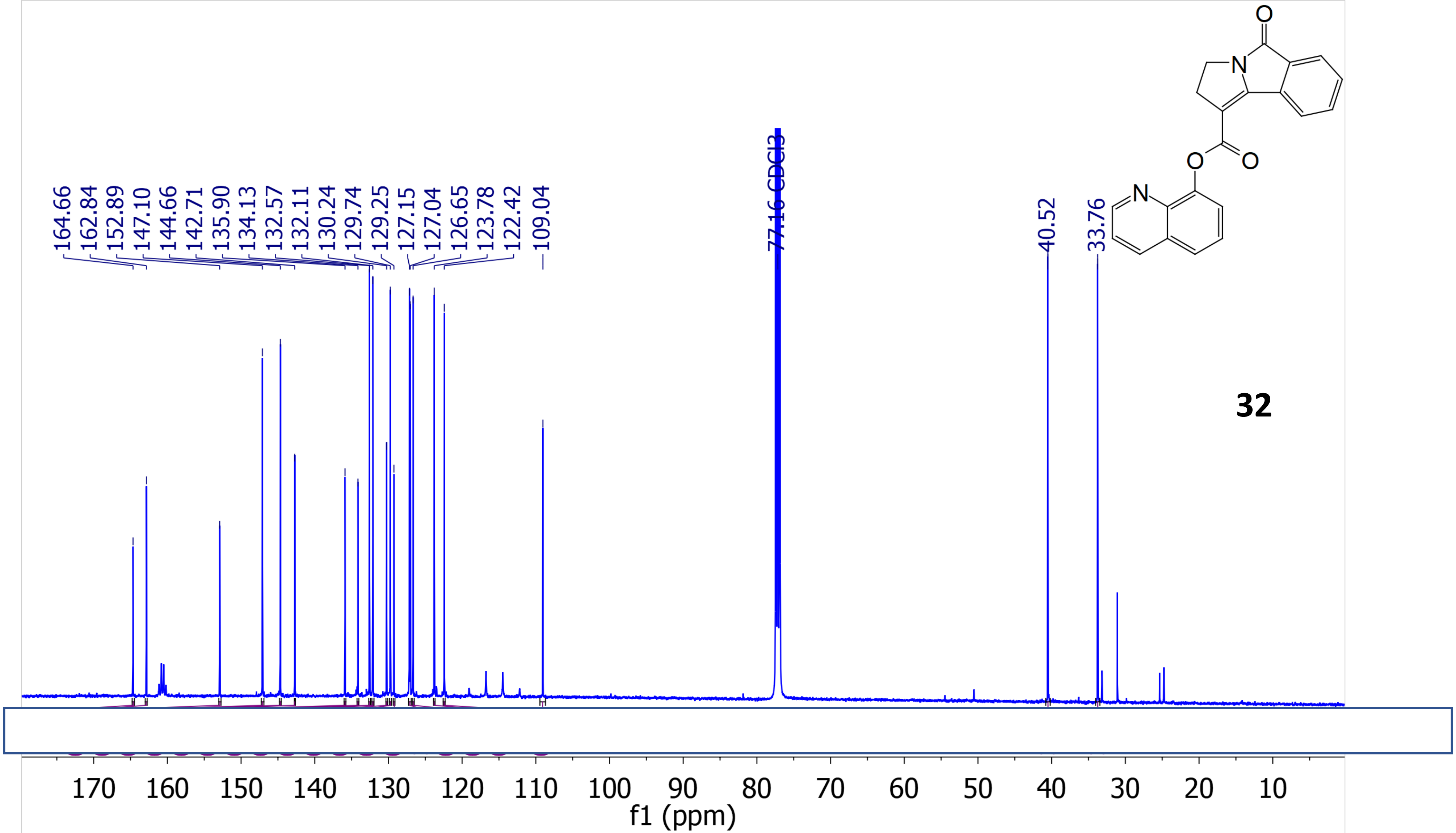

^{13}C NMR (126 MHz, Chloroform-*d*) δ 164.66, 162.84, 152.89, 147.10, 144.66, 142.71, 135.90, 134.13, 132.57, 132.11, 130.24, 129.74, 129.25, 127.15, 127.04, 126.65, 123.78, 122.42, 109.04, 40.52, 33.76.

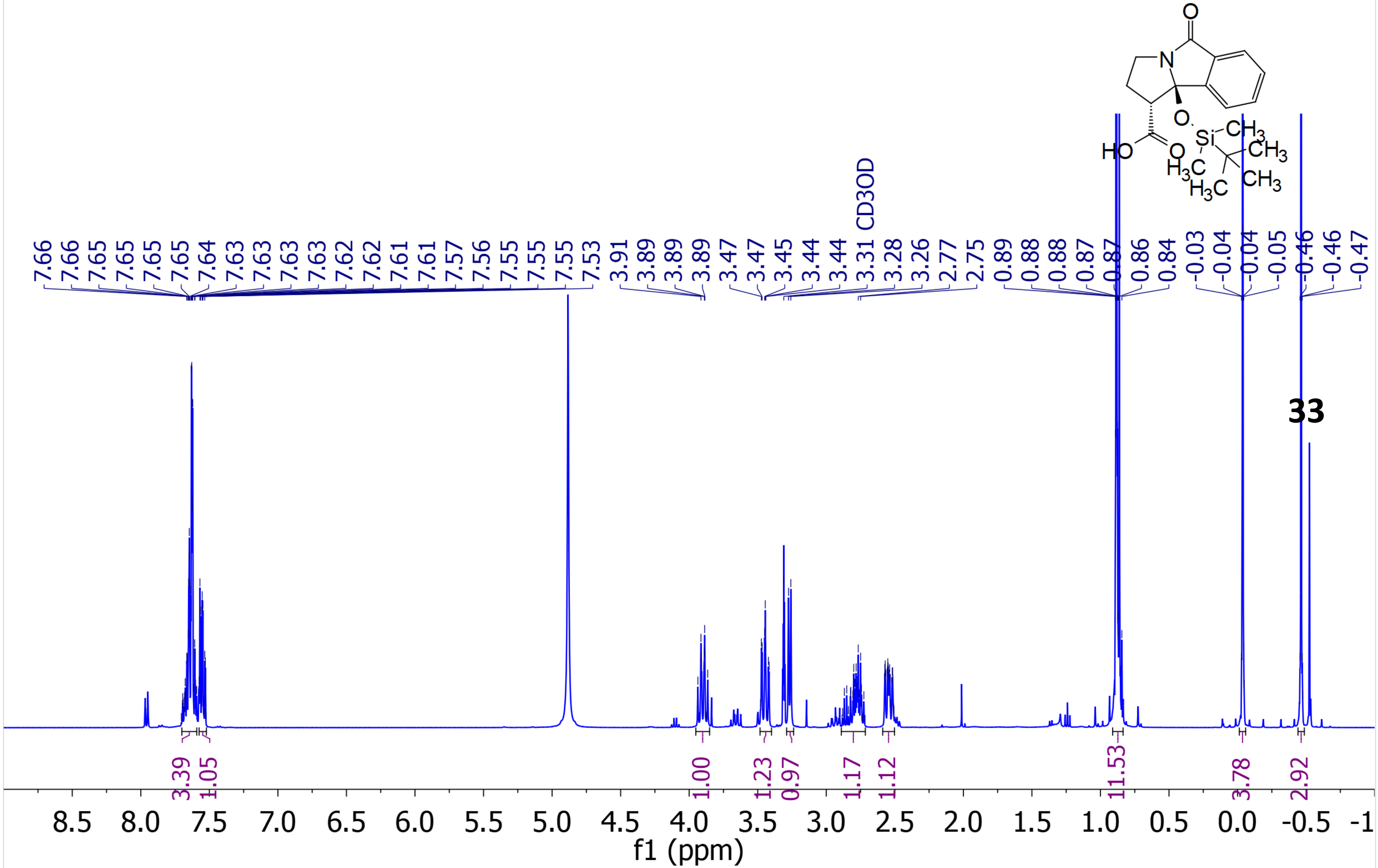

^{1}H NMR (400 MHz, Methanol-d_4) δ 7.70 – 7.59 (m, 3H), 7.58 – 7.52 (m, 1H), 3.90 (ddd, J = 11.0, 9.4, 8.5 Hz, 1H), 3.45 (ddd, J = 11.2, 9.5, 2.0 Hz, 1H), 3.27 (d, J = 6.8 Hz, 1H), 2.89 – 2.72 (m, 1H), 2.59 – 2.50 (m, 1H), 0.87 (d, J = 8.5 Hz, 9H), -0.04 (d, J = 1.3 Hz, 3H), -0.46 (s, 3H).

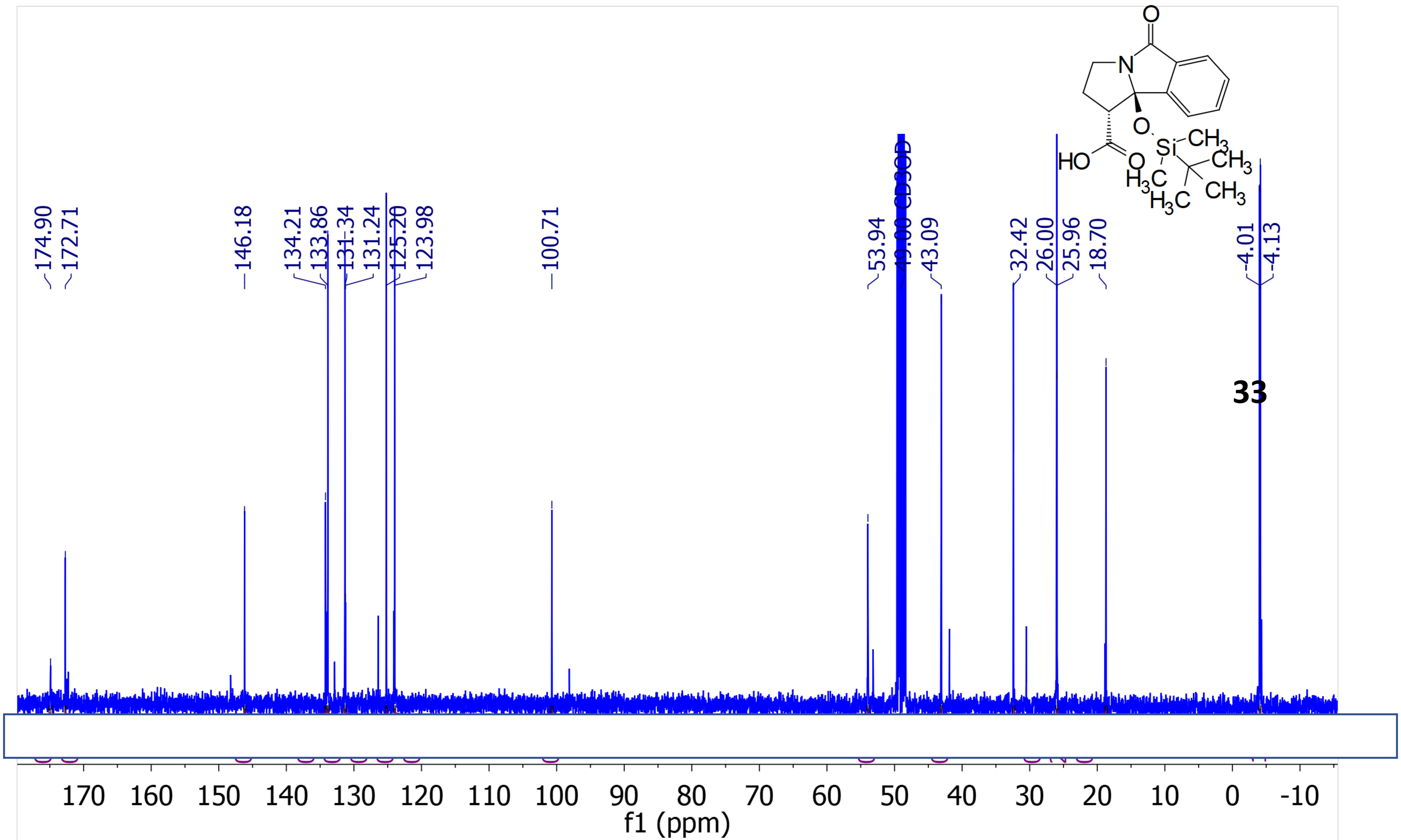

^{13}C NMR (101 MHz, MeOD) δ 172.71, 146.18, 134.21, 133.86, 131.34, 131.24, 125.20, 123.98, 100.71, 53.94, 43.09, 32.42, 26.00, 25.96, 18.70, -4.01, -4.13.

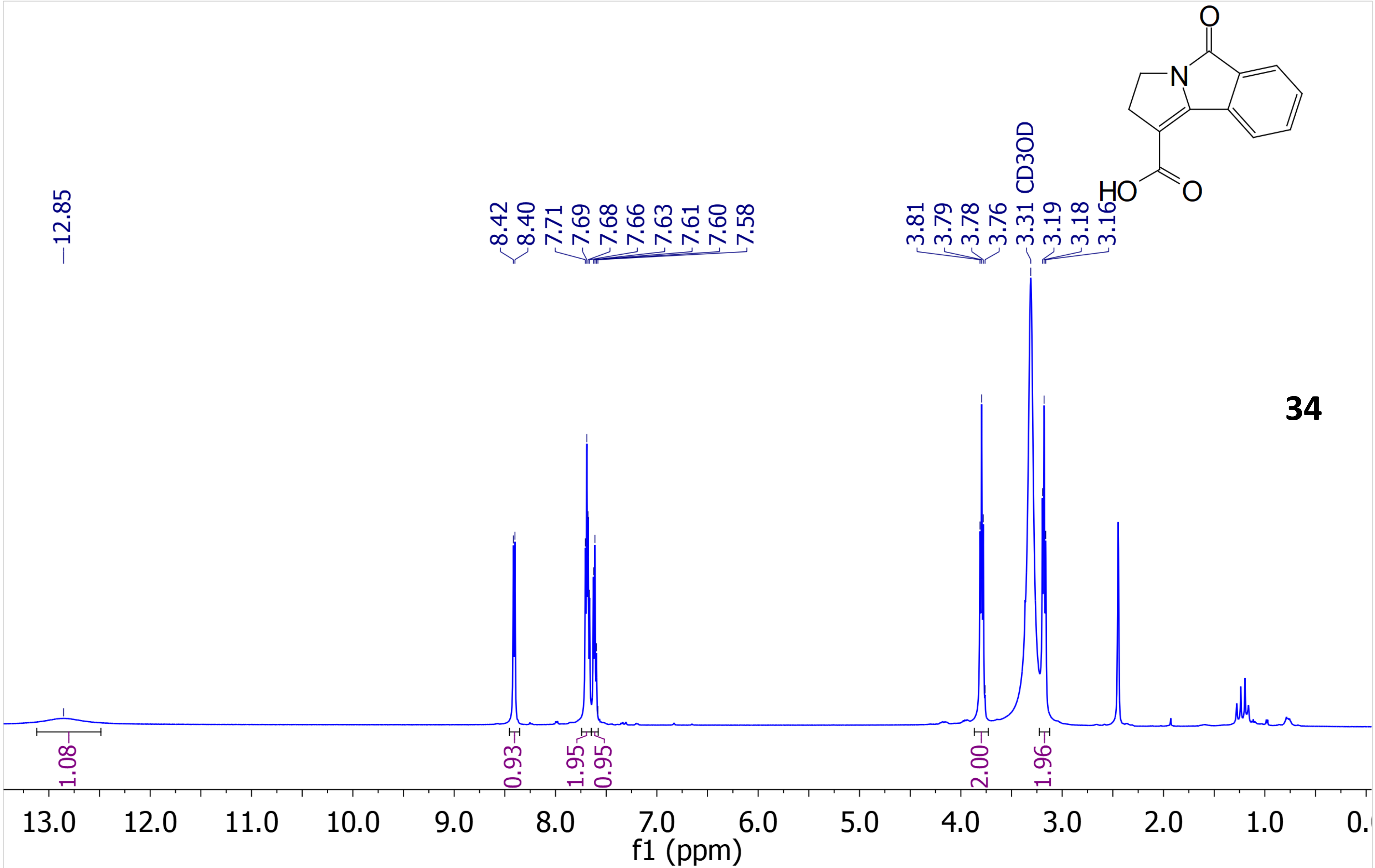

^1H NMR (500 MHz, DMSO-d_6) δ 12.91 (s, 1H), 8.46 (d, J = 7.7 Hz, 1H), 7.80 – 7.70 (m, 2H), 7.66 (t, J = 7.4 Hz, 1H), 3.85 (t, J = 8.2 Hz, 2H), 3.23 (t, J = 8.2 Hz, 2H).

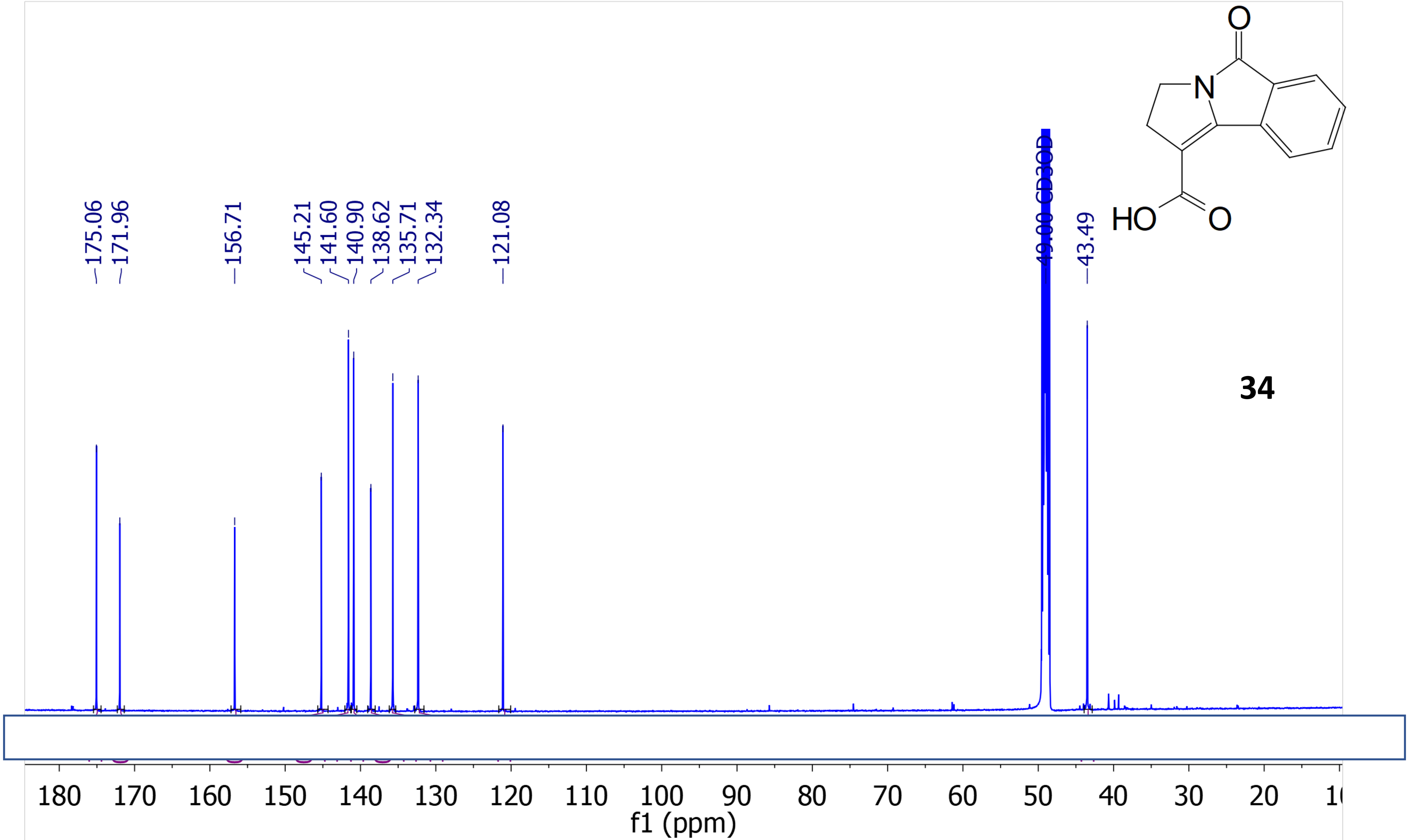

^{13}C NMR (126 MHz, DMSO) δ 165.58, 162.48, 147.23, 135.73, 132.12, 131.42, 129.14, 126.23, 122.86, 111.60, 34.01.

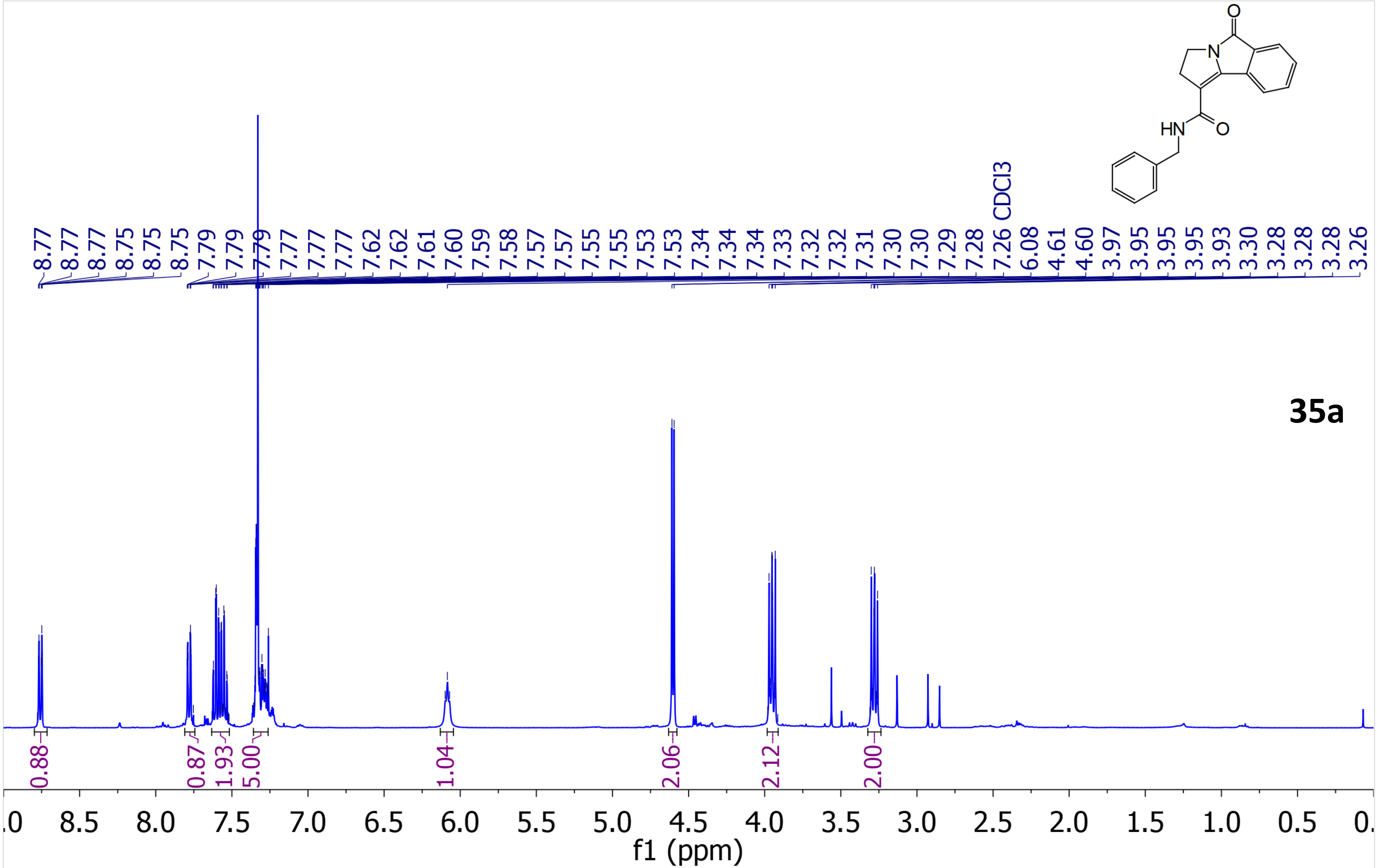

35a

^{1}H NMR (400 MHz, Chloroform-*d*) δ 8.76 (dt, *J* = 7.8, 1.0 Hz, 1H), 7.78 (td, 1H), 7.58 (dtd, 2H), 7.36 – 7.26 (m, 5H), 6.08 (t, *J* = 5.8 Hz, 1H), 4.60 (d, *J* = 5.8 Hz, 2H), 3.95 (t, 2H), 3.28 (t, 2H).

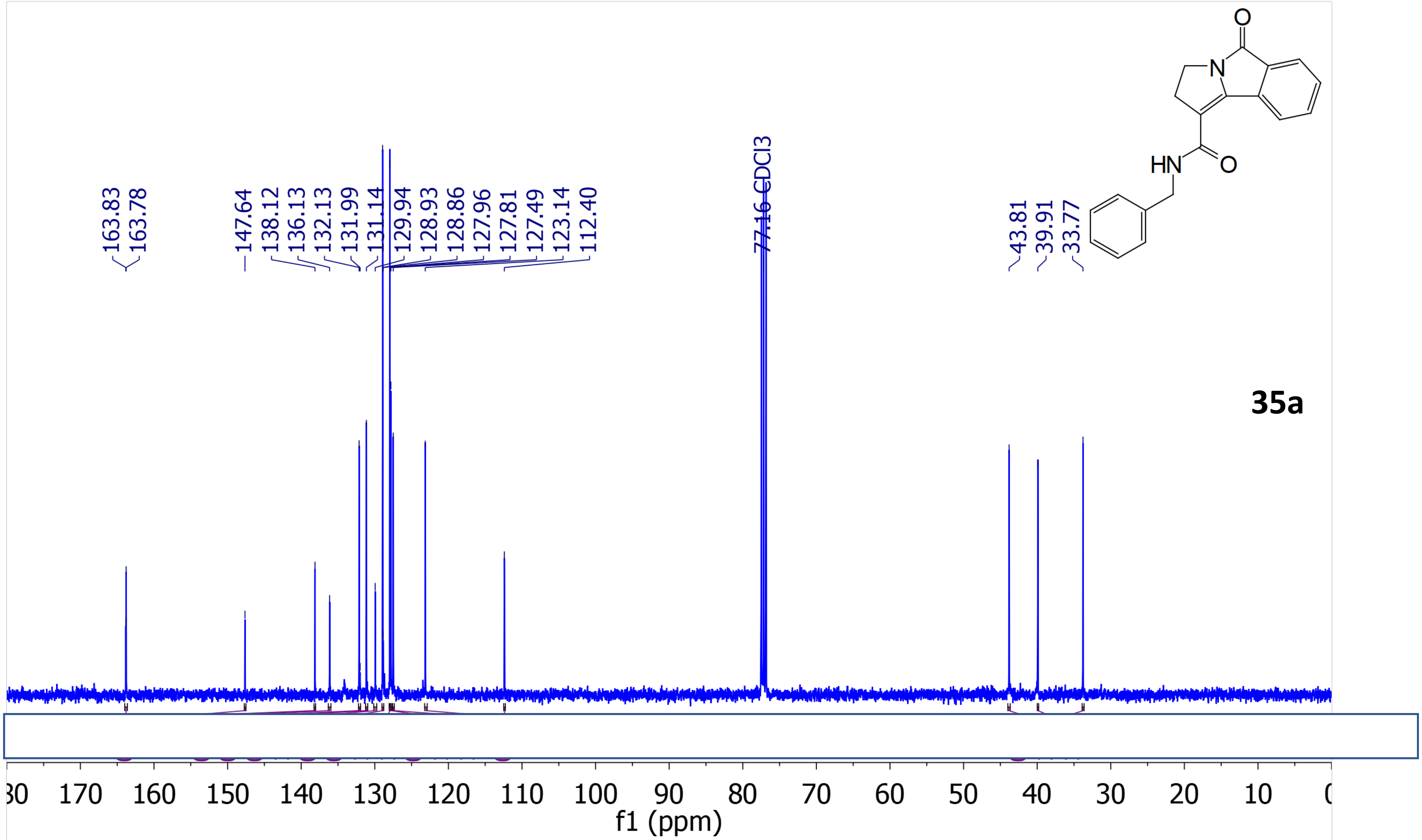

^{13}C NMR (101 MHz, $CDCl_3$) δ 163.83, 163.78, 147.64, 138.12, 136.13, 132.13, 131.99, 131.14, 129.94, 128.93, 128.86, 127.96, 127.81, 127.49, 123.14, 112.40, 43.81, 39.91, 33.77.

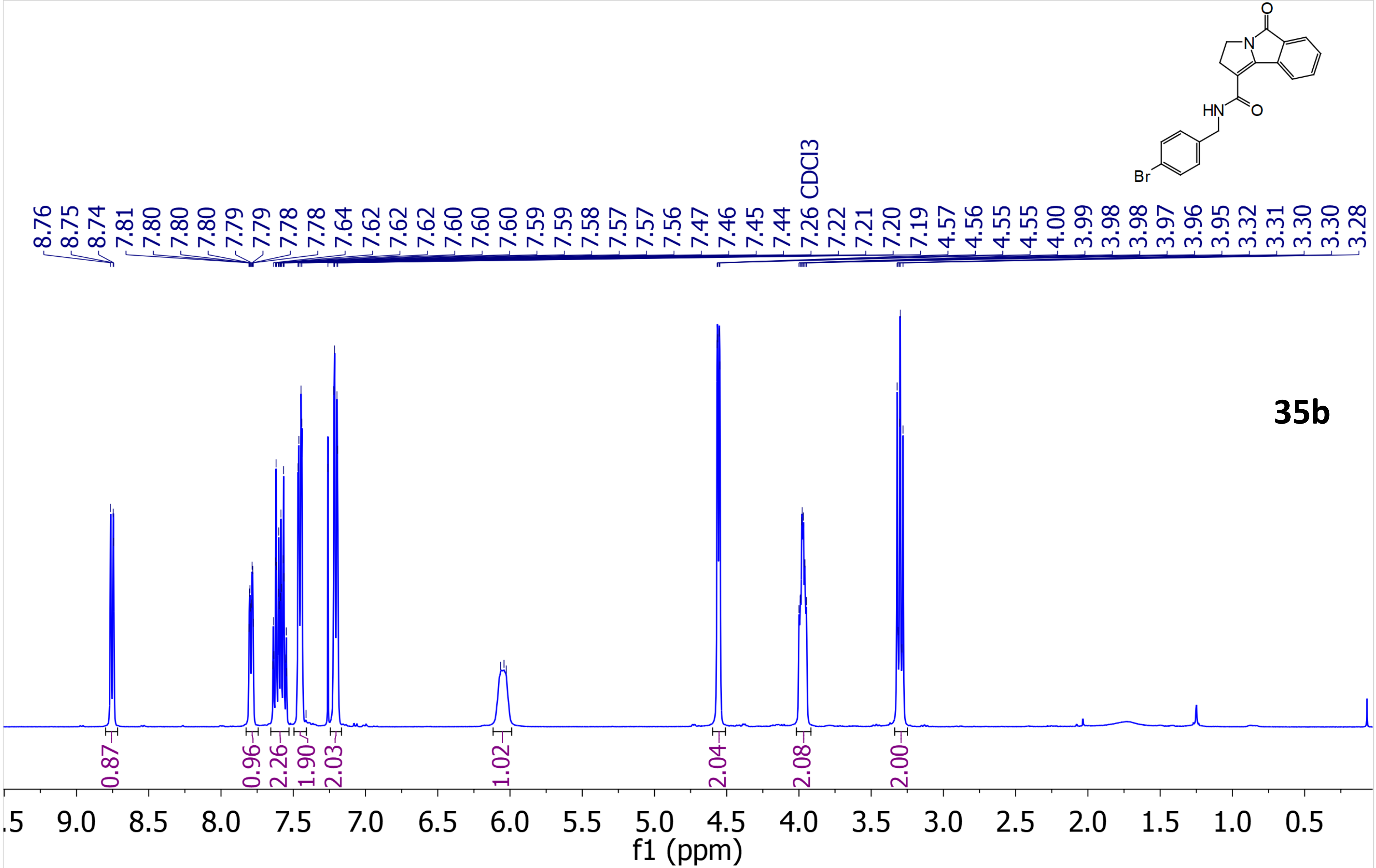

^{1}H NMR (400 MHz, Chloroform-*d*) δ 8.80 – 8.72 (m, 1H), 7.79 (d, *J* = 8.0, 1.4 Hz, 1H), 7.60 (dt, 2H), 7.45 (dd, *J* = 8.4, 2.5 Hz, 2H), 7.21 (dd, *J* = 8.4, 2.0 Hz, 2H), 6.04 (s, 1H), 4.56 (d, *J* = 5.9, 1.7 Hz, 2H), 3.97 (td, *J* = 8.5, 4.0 Hz, 2H), 3.30 (t, *J* = 9.0, 7.4 Hz, 2H).

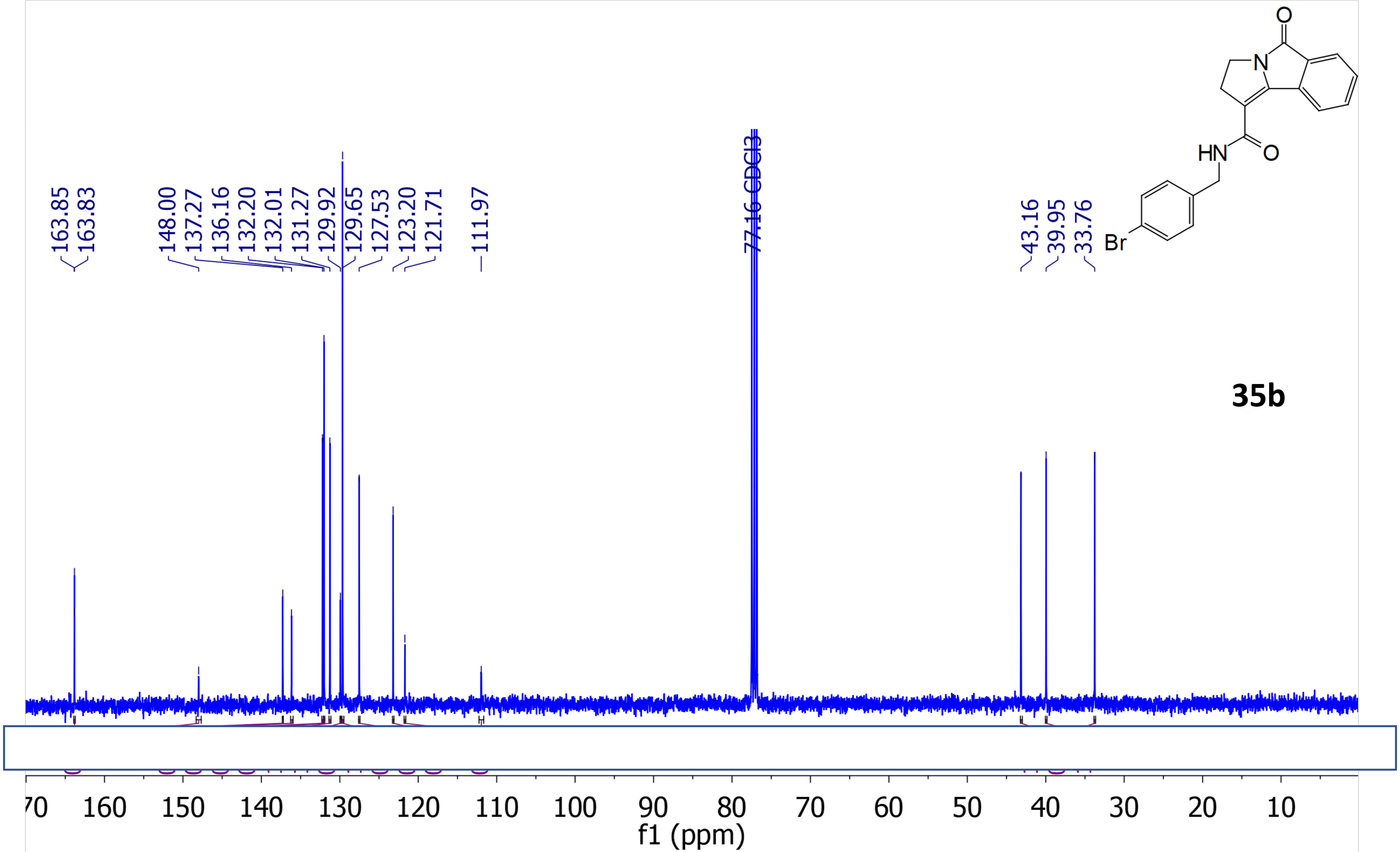

35b

^{13}C NMR (101 MHz, Chloroform-*d*) δ 163.85, 163.83, 148.00, 137.27, 136.16, 132.20, 132.01, 131.27, 129.92, 129.65, 127.53, 123.20, 121.71, 111.97, 43.16, 39.95, 33.76.

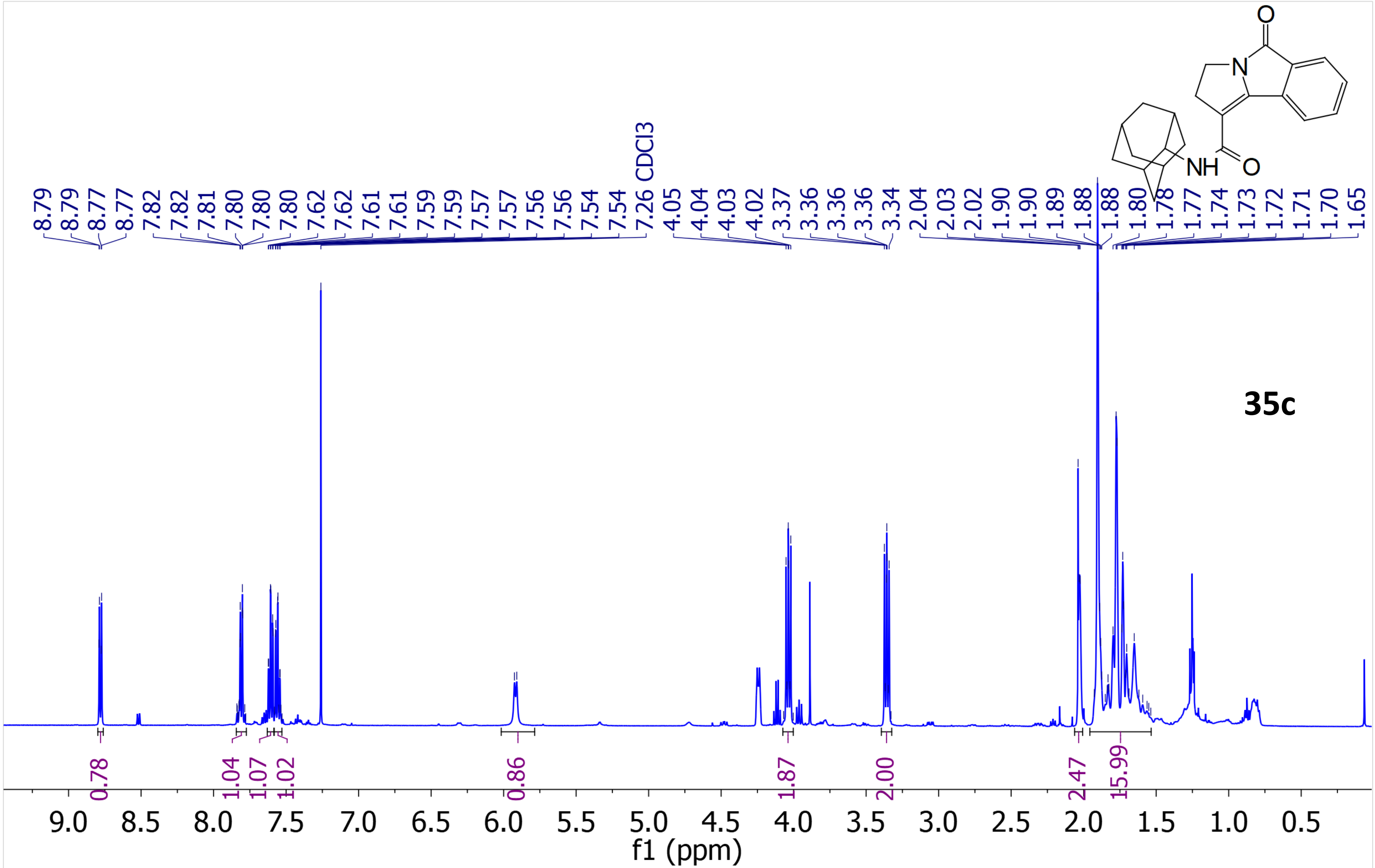

^{1}H NMR (500 MHz, Chloroform-*d*) δ 8.81 (dd, J = 7.5, 1.2 Hz, 1H), 7.84 (dt, J = 7.3, 0.9 Hz, 1H), 7.63 (td, J = 7.5, 1.3 Hz, 1H), 7.58 (td, J = 7.4, 1.2 Hz, 1H), 4.06 (t, J = 8.9, 7.5 Hz, 2H), 3.38 (t, 2H), 2.09 – 2.03 (m, 2H), 1.98 – 1.56 (m, 15H).

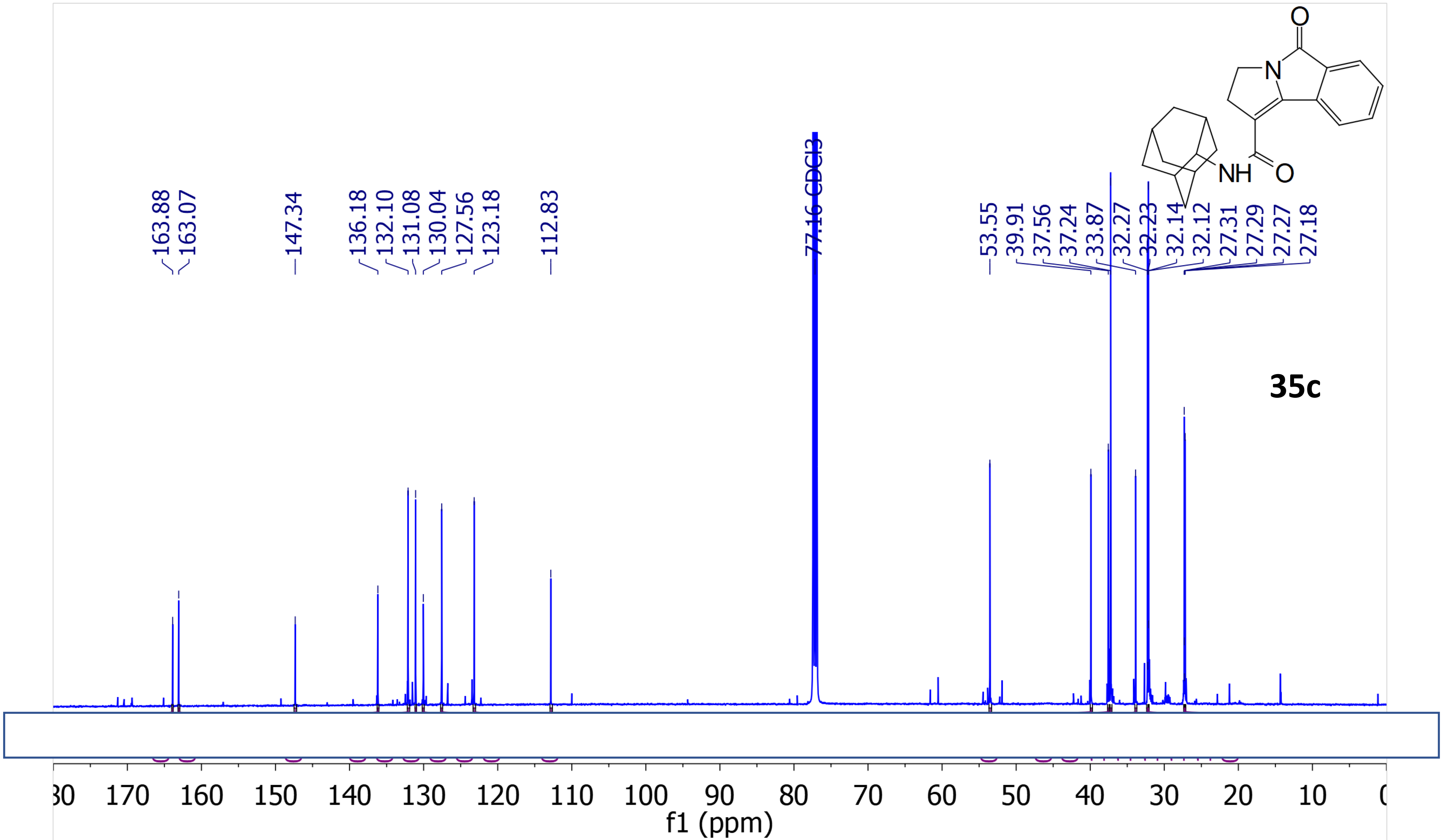

^{13}C NMR (126 MHz, $CDCl_3$) δ 163.88, 163.07, 147.34, 136.18, 132.10, 131.08, 130.04, 127.56, 123.18, 112.83, 53.55, 39.91, 37.56, 37.24, 33.87, 32.27, 32.23, 32.14, 32.12, 27.31, 27.29, 27.27, 27.18.

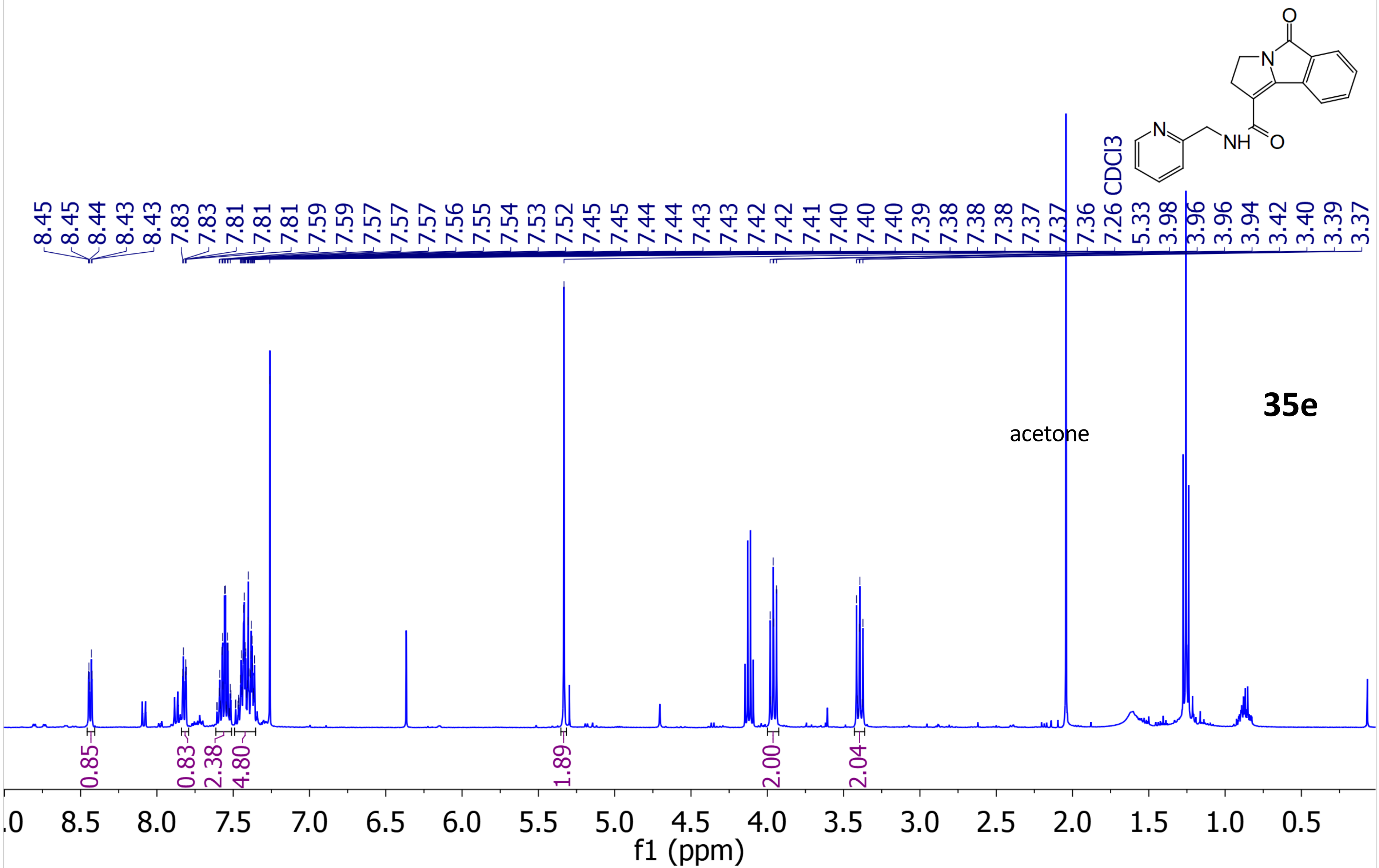

^1H NMR (400 MHz, Chloroform-*d*) δ 8.46 – 8.41 (m, 1H), 7.84 – 7.79 (m, 1H), 7.61 – 7.51 (m, 2H), 7.49 – 7.35 (m, 5H), 5.33 (s, 2H), 3.96 (dd, J = 9.1, 7.7 Hz, 2H), 3.39 (dd, J = 9.1, 7.7 Hz, 2H).

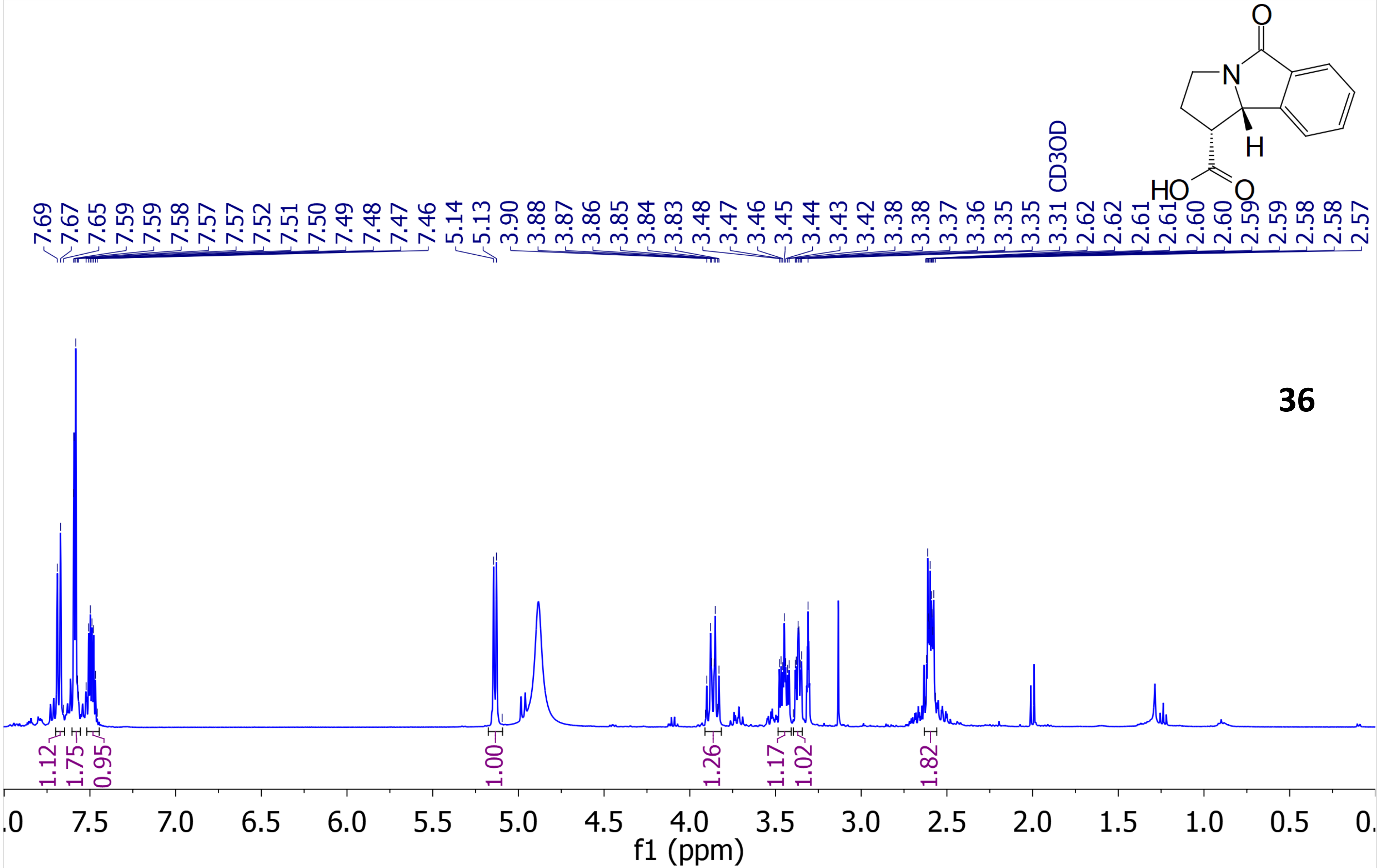

^{1}H NMR (400 MHz, Methanol-d_4) δ 7.68 (d, J = 7.5 Hz, 1H), 7.59 (d, 2H), 7.49 (dt, J = 8.1, 4.1 Hz, 1H), 5.14 (d, J = 6.7 Hz, 1H), 3.87 (dt, J = 11.2, 8.7 Hz, 1H), 3.45 (ddd, J = 11.4, 8.1, 3.9 Hz, 1H), 3.37 (td, J = 6.3, 2.3 Hz, 1H), 2.63 – 2.56 (m, 2H).

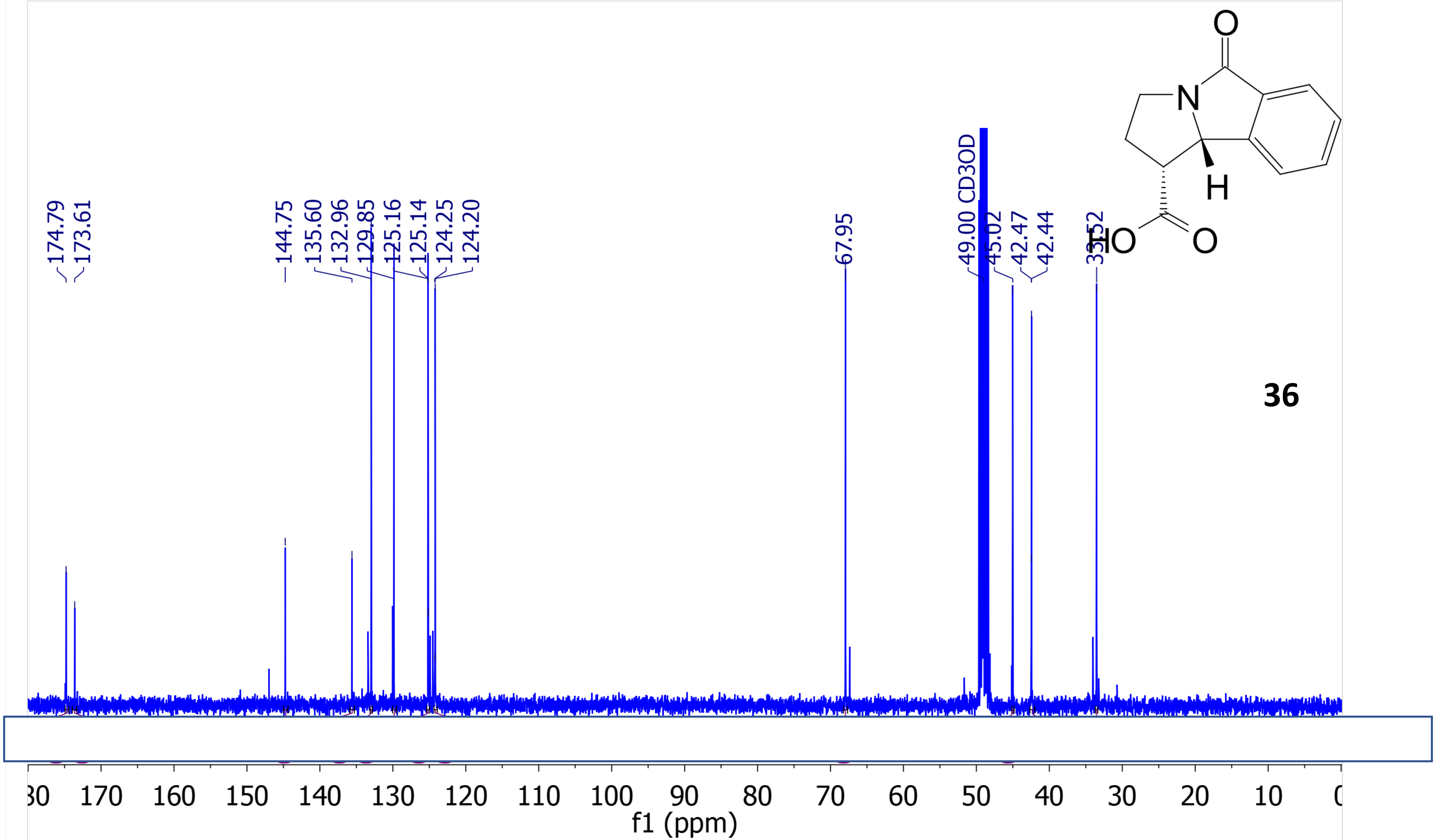

^{13}C NMR (101 MHz, MeOD) δ 174.79, 173.61, 144.75, 135.60, 132.96, 129.85, 125.16, 125.14, 124.25, 124.20, 67.95, 45.02, 42.47, 42.44, 33.52.

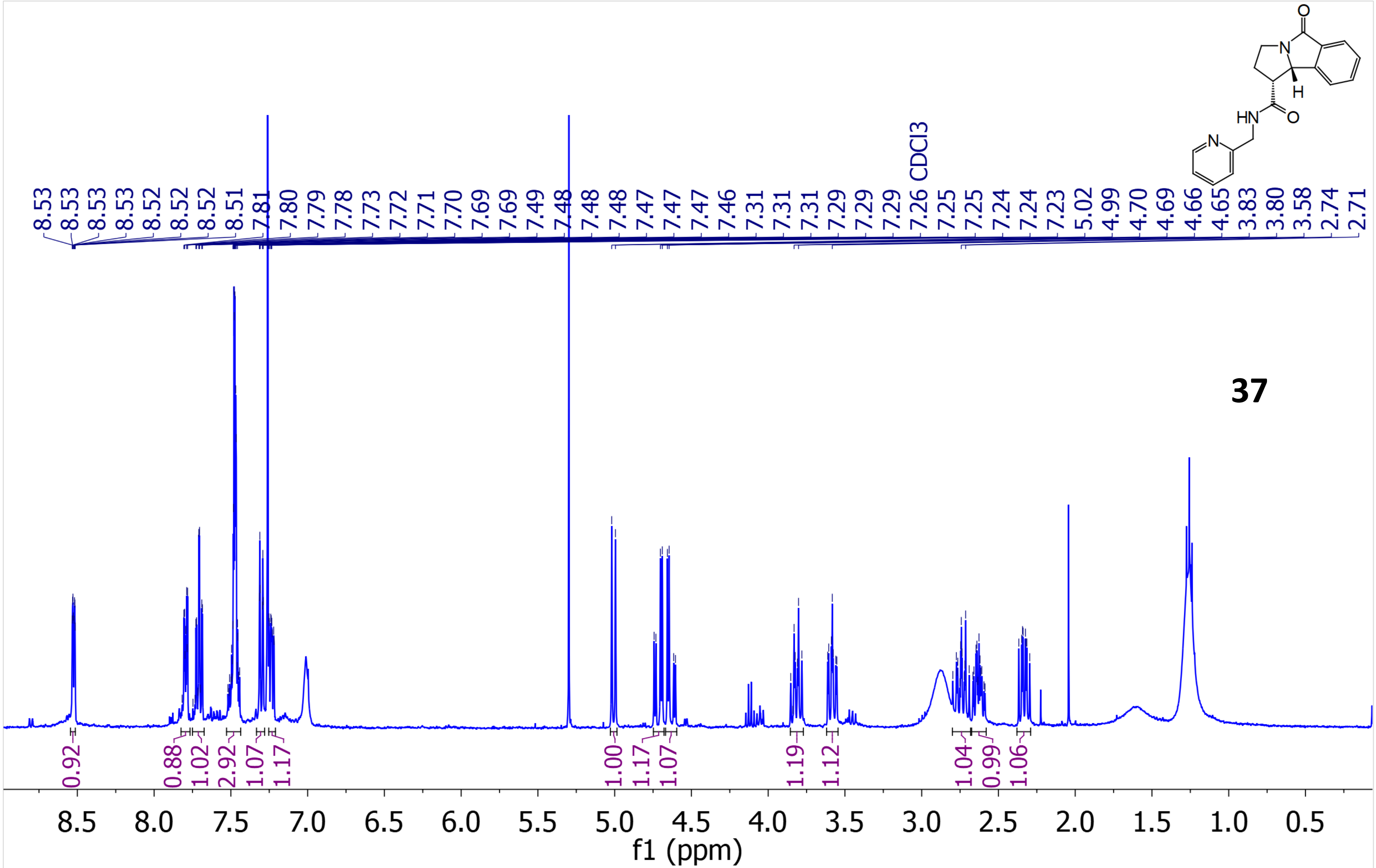

^{1}H NMR (400 MHz, Chloroform-*d*) δ 8.52 (ddd, J = 4.9, 1.9, 1.0 Hz, 1H), 7.82 – 7.77 (m, 1H), 7.71 (td, J = 7.7, 1.8 Hz, 1H), 7.53 – 7.44 (m, 3H), 7.30 (dt, J = 7.9, 1.0 Hz, 1H), 7.24 (ddd, J = 7.6, 5.0, 1.1 Hz, 1H), 5.01 (d, J = 9.8 Hz, 1H), 4.72 (dd, J = 16.5, 4.8 Hz, 1H), 4.63 (dd, J = 16.5, 4.6 Hz, 1H), 3.82 (dt, J = 11.6, 8.7 Hz, 1H), 3.58 (ddd, J = 11.7, 9.5, 2.6 Hz, 1H), 2.74 (ddt, J = 12.9, 11.3, 9.3 Hz, 1H), 2.67 – 2.58 (m, 1H), 2.33 (ddd, J = 11.2, 9.7, 7.5 Hz, 1H).

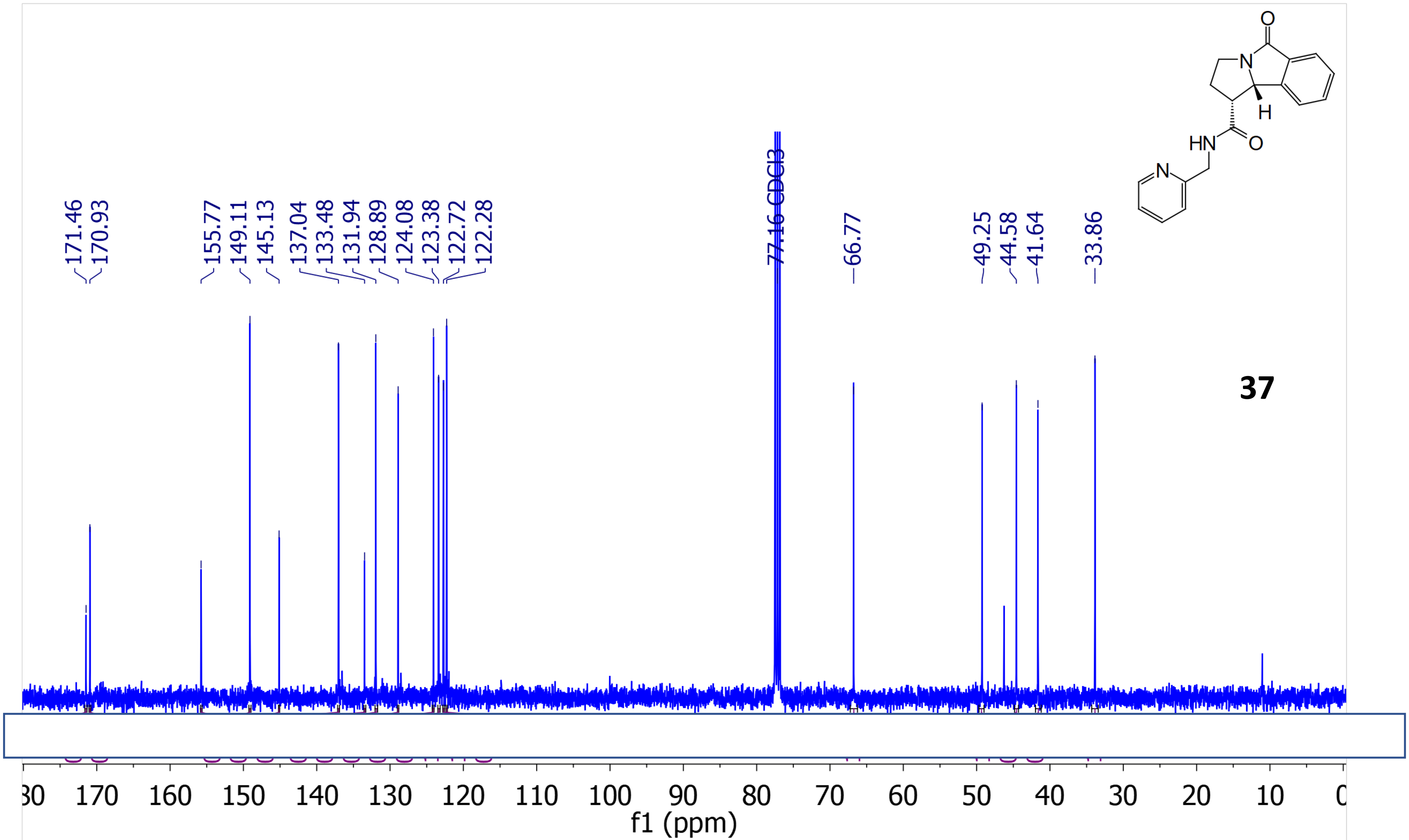

^{13}C NMR (101 MHz, $CDCl_3$) δ 171.46, 170.93, 155.77, 149.11, 145.13, 137.04, 133.48, 131.94, 128.89, 124.08, 123.38, 122.72, 122.28, 66.77, 49.25, 44.58, 41.64, 33.86.
